# Forecasting the effects of smoking prevalence scenarios on years of life lost and life expectancy from 2022 to 2050: a systematic analysis for the Global Burden of Disease Study 2021

**DOI:** 10.1016/S2468-2667(24)00166-X

**Published:** 2024-10-02

**Authors:** GBD 2021 Tobacco Forecasting Collaborators, Dana Bryazka, Marissa B Reitsma, Yohannes Habtegiorgis Abate, Abdallah H A Abd Al Magied, Atef Abdelkader, Arash Abdollahi, Meriem Abdoun, Rizwan Suliankatchi Abdulkader, Roberto Ariel Abeldaño Zuñiga, E S Abhilash, Olugbenga Olusola Abiodun, Olumide Abiodun, Richard Gyan Aboagye, Lucas Guimarães Abreu, Dariush Abtahi, Hasan Abualruz, Bilyaminu Abubakar, Niveen ME Abu-Rmeileh, Salahdein Aburuz, Ahmed Abu-Zaid, Mesafint Molla Adane, Akindele Olupelumi Adebiyi, Oyelola A Adegboye, Victor Adekanmbi, Habeeb Omoponle Adewuyi, Qorinah Estiningtyas Sakilah Adnani, Leticia Akua Adzigbli, Siamak Afaghi, Aanuoluwapo Adeyimika Afolabi, Muhammad Sohail Afzal, Saira Afzal, Antonella Agodi, Williams Agyemang-Duah, Bright Opoku Ahinkorah, Austin J Ahlstrom, Aqeel Ahmad, Danish Ahmad, Muayyad M Ahmad, Sajjad Ahmad, Shahzaib Ahmad, Ali Ahmadi, Anisuddin Ahmed, Ayman Ahmed, Haroon Ahmed, Muktar Beshir Ahmed, Safoora Ahmed, Marjan Ajami, Mohammed Ahmed Akkaif, Ema Akter, Salah Al Awaidy, Syed Mahfuz Al Hasan, Yazan Al-Ajlouni, Ziyad Al-Aly, Khurshid Alam, Zufishan Alam, Wafa A Aldhaleei, Abdelazeem M Algammal, Adel Ali Saeed Al-Gheethi, Khalid F Alhabib, Fadwa Naji Alhalaiqa, Mohammed Khaled Al-Hanawi, Abid Ali, Mohammed Usman Ali, Rafat Ali, Syed Shujait Ali, Waad Ali, Sheikh Mohammad Alif, Syed Mohamed Aljunid, François Alla, Peter Allebeck, Wael Almahmeed, Sabah Al-Marwani, Sadeq Al-Maweri, Mahmoud A Alomari, Jaber S Alqahtani, Ahmed Yaseen Alqutaibi, Rajaa M Mohammad Al-Raddadi, Sahel Majed Alrousan, Saqr Alsakarneh, Najim Z Alshahrani, Zaid Altaany, Awais Altaf, Nelson Alvis-Guzman, Mohammad Al-Wardat, Yaser Mohammed Al-Worafi, Hany Aly, Safwat Aly, Mohammad Sharif Ibrahim Alyahya, Karem H Alzoubi, Walid Adnan Al-Zyoud, Reza Amani, Tarek Tawfik Amin, Sohrab Amiri, Hubert Amu, Gianna Gayle Herrera Amul, Ganiyu Adeniyi Amusa, Tanu Anand, Deanna Anderlini, David B Anderson, Jason A Anderson, Catalina Liliana Andrei, Tudorel Andrei, Mohammed Tahir Ansari, Iyadunni Adesola Anuoluwa, Saeid Anvari, Sumadi Lukman Anwar, Anayochukwu Edward Anyasodor, Jalal Arabloo, Elshaimaa A Arafa, Aleksandr Y Aravkin, Demelash Areda, Brhane Berhe Aregawi, Olatunde Aremu, Anton A Artamonov, Akeza Awealom Asgedom, Mohammad Asghari-Jafarabadi, Mubarek Yesse Ashemo, Tahira Ashraf, Thomas Astell-Burt, Seyyed Shamsadin Athari, Prince Atorkey, Alok Atreya, Avinash Aujayeb, Adedapo Wasiu Awotidebe, Getinet Ayano, Setognal Birara Aychiluhm, Sina Azadnajafabad, Ahmed Y Azzam, Giridhara Rathnaiah Babu, Pegah Bahrami Taghanaki, Saeed Bahramian, Ruhai Bai, Shankar M Bakkannavar, Senthilkumar Balakrishnan, Kiran Bam, Maciej Banach, Soham Bandyopadhyay, Mehmet Firat Baran, Martina Barchitta, Mainak Bardhan, Suzanne Lyn Barker-Collo, Amadou Barrow, Hameed Akande Bashiru, Afisu Basiru, Mohammad-Mahdi Bastan, Sanjay Basu, Saurav Basu, Kavita Batra, Mohsen Bayati, Amir Hossein Behnoush, Shelly L Bell, Luis Belo, Alice A Beneke, Derrick A Bennett, Isabela M Bensenor, Azizullah Beran, Amiel Nazer C Bermudez, Habtamu B Beyene, Devidas S Bhagat, Akshaya Srikanth Bhagavathula, Neeraj Bhala, Nikha Bhardwaj, Pankaj Bhardwaj, Sonu Bhaskar, Ajay Nagesh Bhat, Natalia V Bhattacharjee, Priyadarshini Bhattacharjee, Jasvinder Singh Bhatti, Cem Bilgin, Atanu Biswas, Bijit Biswas, Micheal Kofi Boachie, Eyob Ketema Bogale, Berrak Bora Basara, Hamed Borhany, Samuel Adolf Bosoka, Souad Bouaoud, Edward J Boyko, Hermann Brenner, Andre R Brunoni, Raffaele Bugiardini, Norma B Bulamu, Yasser Bustanji, Zahid A Butt, Florentino Luciano Caetano dos Santos, Daniela Calina, Chao Cao, Fan Cao, Angelo Capodici, Rosario Cárdenas, Giulia Carreras, Joao Mauricio Castaldelli-Maia, Maria Sofia Cattaruzza, Arthur Caye, Luca Cegolon, Edina Cenko, Sonia Cerrai, Sandip Chakraborty, Rama Mohan Chandika, Eeshwar K Chandrasekar, Vijay Kumar Chattu, Anis Ahmad Chaudhary, Akhilanand Chaurasia, An-Tian Chen, Guangjin Chen, Haowei Chen, Meng Xuan Chen, Simiao Chen, Kent Jason Go Cheng, Gerald Chi, Fatemeh Chichagi, Ritesh Chimoriya, Jesus Lorenzo Chirinos-Caceres, Abdulaal Chitheer, Bryan Chong, Chean Lin Chong, Yuen Yu Chong, Hitesh Chopra, Sonali Gajanan Choudhari, Dinh-Toi Chu, Isaac Sunday Chukwu, Sheng-Chia Chung, Muhammad Chutiyami, Joao Conde, Alexandru Corlateanu, Michael H Criqui, Natalia Cruz-Martins, Alanna Gomes da Silva, Omid Dadras, Siyu Dai, Xiaochen Dai, Giovanni Damiani, Lalit Dandona, Rakhi Dandona, Samuel D Darcho, Reza Darvishi Cheshmeh Soltani, Saswati Das, Nihar Ranjan Dash, Kairat Davletov, Aklilu Tamire Debele, Shayom Debopadhaya, Daniel Demant, Hardik Dineshbhai Desai, Devananda Devegowda, Syed Masudur Rahman Dewan, Arkadeep Dhali, Amol S Dhane, Vishal R Dhulipala, Thanh Chi Do, Milad Dodangeh, Phidelia Theresa Doegah, Sushil Dohare, Deepa Dongarwar, Mario D'Oria, Ojas Prakashbhai Doshi, Rajkumar Prakashbhai Doshi, Robert Kokou Dowou, Ashel Chelsea Dsouza, Haneil Larson Dsouza, Viola Savy Dsouza, Bruce B Duncan, Andre Rodrigues Duraes, Arkadiusz Marian Dziedzic, Abdel Rahman E'mar, Alireza Ebrahimi, Negar Ebrahimi, Mohammad Ebrahimi Kalan, David Edvardsson, Kristina Edvardsson, Ferry Efendi, Diyan Ermawan Effendi, Foolad Eghbali, Michael Ekholuenetale, Rabie Adel El Arab, Ibrahim Farahat El Bayoumy, Iman El Sayed, Iffat Elbarazi, Muhammed Elhadi, Waseem El-Huneidi, Mohamed A Elmonem, Gihan ELNahas, Ibrahim Elsohaby, Chadi Eltaha, Mohd Elmagzoub Eltahir, Mehdi Emamverdi, Theophilus I Emeto, Daniel Asfaw Erku, Farshid Etaee, Elochukwu Fortune Ezenwankwo, Natalia Fabin, Adeniyi Francis Fagbamigbe, Omotayo Francis Fagbule, Shahriar Faghani, Ayesha Fahim, Ildar Ravisovich Fakhradiyev, Luca Falzone, Umar Farooque, Ali Fatehizadeh, Zareen Fatima, Nelsensius Klau Fauk, Timur Fazylov, Alireza Feizkhah, Ginenus Fekadu, Xiaoqi Feng, Pietro Ferrara, Nuno Ferreira, Bikila Regassa Feyisa, Filippos T Filippidis, Florian Fischer, Luisa S Flor, Nataliya A Foigt, Celia Fortuna Rodrigues, Matteo Foschi, Sridevi G, Peter Andras Gaal, Muktar A Gadanya, Abhay Motiramji Gaidhane, Márió Gajdács, Silvano Gallus, Aravind P Gandhi, Balasankar Ganesan, Prem Gautam, Rupesh K Gautam, Miglas Welay Gebregergis, Mesfin Gebrehiwot, Teferi Gebru Gebremeskel, Lemma Getacher, Fataneh Ghadirian, Ramy Mohamed Ghazy, Ali Gholamrezanezhad, Mahsa Ghorbani, Sherief Ghozy, Artyom Urievich Gil, Gabriela Fernanda Gil, Elena V Gnedovskaya, Sonu Goel, Salime Goharinezhad, Mohamad Goldust, Mahaveer Golechha, Pouya Goleij, Davide Golinelli, Giuseppe Gorini, Mahdi Gouravani, Ayman Grada, Michal Grivna, Shekhar Grover, Shi-Yang Guan, Mohammed Ibrahim Mohialdeen Gubari, Avirup Guha, Stefano Guicciardi, Snigdha Gulati, Damitha Asanga Gunawardane, Sasidhar Gunturu, Zhifeng Guo, Anish Kumar Gupta, Bhawna Gupta, Ishita Gupta, Mohak Gupta, Rajeev Gupta, Sapna Gupta, Veer Bala Gupta, Vipin Gupta, Vivek Kumar Gupta, Mostafa Hadei, Najah R Hadi, Ali Hajj Ali, Esam S Halboub, Nadia M Hamdy, Samer Hamidi, Ahmad Hammoud, Graeme J Hankey, Arief Hargono, Josep Maria Haro, Ahmed I Hasaballah, Faizul Hasan, Md Kamrul Hasan, Md Saquib Hasnain, Amr Hassan, Ikrama Ibrahim Hassan, Shoaib Hassan, Simon I Hay, Behzad Heibati, Mohammad Heidari, Mehdi Hemmati, Delia Hendrie, Claudiu Herteliu, Demisu Zenbaba Heyi, Kamal Hezam, Yuta Hiraike, Nguyen Quoc Hoan, Ramesh Holla, Nobuyuki Horita, Md Mahbub Hossain, Sahadat Hossain, Hassan Hosseinzadeh, Mihaela Hostiuc, Sorin Hostiuc, Junjie Huang, Ayesha Humayun, Javid Hussain, Bing-Fang Hwang, Segun Emmanuel Ibitoye, Nayu Ikeda, Adalia Ikiroma, Olayinka Stephen Ilesanmi, Irena M Ilic, Milena D Ilic, Mustapha Immurana, Leeberk Raja Inbaraj, Muhammad Iqhrammullah, Lalu Muhammad Irham, Md Rabiul Islam, Sheikh Mohammed Shariful Islam, Farhad Islami, Gaetano Isola, Ramaiah Itumalla, Masao Iwagami, Mahalaxmi Iyer, Vinothini J, Jalil Jaafari, Louis Jacob, Abdollah Jafarzadeh, Khushleen Jaggi, Nader Jahanmehr, Akhil Jain, Nityanand Jain, Ammar Abdulrahman Jairoun, Sanobar Jaka, Mihajlo Jakovljevic, Reza Jalilzadeh Yengejeh, Elham Jamshidi, Manthan Dilipkumar Janodia, Talha Jawaid, Sathish Kumar Jayapal, Shubha Jayaram, Ruwan Duminda Jayasinghe, Rime Jebai, Sun Ha Jee, Bijay Mukesh Jeswani, Heng Jiang, Mohammad Jokar, Jost B Jonas, Tamas Joo, Nitin Joseph, Charity Ehimwenma Joshua, Jacek Jerzy Jozwiak, Mikk Jürisson, Vaishali K, Ali Kabir, Zubair Kabir, Vidya Kadashetti, Sivesh Kathir Kamarajah, Mona Kanaan, Kehinde Kazeem Kanmodi, Surya Kant, Rami S Kantar, Paschalis Karakasis, Ibraheem M Karaye, Salah Eddin Karimi, Yeganeh Karimi, Arman Karimi Behnagh, Samad Karkhah, Prabin Karki, Faizan Zaffar Kashoo, Srinivasa Vittal Katikireddi, Harkiran Kaur, Navjot Kaur, Sina Kazemian, Tahseen Haider Kazmi, Peter Njenga Keiyoro, Emmanuelle Kesse-Guyot, Yousef Saleh Khader, Himanshu Khajuria, Amirmohammad Khalaji, Alireza Khalilian, Ajmal Khan, Maseer Khan, Mohammad Jobair Khan, Moien AB Khan, Shaghayegh Khanmohammadi, Khaled Khatab, Haitham Khatatbeh, Moawiah Mohammad Khatatbeh, Amir M Khater, Khalid A Kheirallah, Manoj Khokhar, Moein Khormali, Atulya Aman Khosla, Sepehr Khosravi, Kwanghyun Kim, Min Seo Kim, Yun Jin Kim, Adnan Kisa, Ali-Asghar Kolahi, Somayeh Komaki, Shivakumar KM Marulasiddaiah Kondlahalli, Miikka Korja, Oleksii Korzh, Soewarta Kosen, Karel Kostev, Kewal Krishan, Barthelemy Kuate Defo, Mohammed Kuddus, Omar Kujan, Mukhtar Kulimbet, Ashish Kumar, G Anil Kumar, Nithin Kumar, Rakesh Kumar, Vijay Kumar, Amartya Kundu, Satyajit Kundu, Setor K Kunutsor, Om P Kurmi, Dian Kusuma, Frank Kyei-Arthur, Ville Kytö, Carlo La Vecchia, Chandrakant Lahariya, Daphne Teck Ching Lai, Hanpeng Lai, Ratilal Lalloo, Tea Lallukka, Bagher Larijani, Savita Lasrado, Jerrald Lau, Paolo Lauriola, Thao Thi Thu Le, Janet L Leasher, Munjae Lee, Seung Won Lee, Wei-Chen Lee, Yo Han Lee, Elvynna Leong, Temesgen Leka Lerango, An Li, Wei Li, Virendra S Ligade, Stephen S Lim, Jialing Lin, Paulina A Lindstedt, Gang Liu, Erand Llanaj, José Francisco López-Gil, Paulo A Lotufo, Giancarlo Lucchetti, Alessandra Lugo, Jay B Lusk, Hawraz Ibrahim M Amin, Zheng Feei Ma, Monika Machoy, Farzan Madadizadeh, Elham Mahmoudi, Abdelrahman M Makram, Omar M Makram, Kashish Malhotra, Ahmad Azam Malik, Deborah Carvalho Malta, Abdullah A Mamun, Pejman Mansouri, Mohammad Ali Mansournia, Emmanuel Manu, Hamid Reza Marateb, Jose Martinez-Raga, Miquel Martorell, Roy Rillera Marzo, Yasith Mathangasinghe, Elezebeth Mathews, Medha Mathur, Navgeet Mathur, Rita Mattiello, Andrea Maugeri, Martin McKee, Enkeleint A Mechili, Ravi Mehrotra, Tesfahun Mekene Meto, Birye Dessalegn Mekonnen, Hadush Negash Meles, Walter Mendoza, Ritesh G Menezes, Sultan Ayoub Meo, Atte Meretoja, Tuomo J Meretoja, Tomislav Mestrovic, Caine C A Meyers, Irmina Maria Michalek, Ted R Miller, Giuseppe Minervini, Mojgan Mirghafourvand, Erkin M Mirrakhimov, Vinaytosh Mishra, Sanjeev Misra, Prasanna Mithra, Ahmed Ismail Mohamed, Jama Mohamed, Mouhand F H Mohamed, Nouh Saad Mohamed, Ameen Mosa Mohammad, Sakineh Mohammad-Alizadeh-Charandabi, Ibrahim Mohammadzadeh, Hussen Mohammed, Shafiu Mohammed, Syam Mohan, Ali H Mokdad, Hossein Molavi Vardanjani, Sabrina Molinaro, Shaher Momani, Himel Mondal, Ute Mons, AmirAli Moodi Ghalibaf, Maryam Moradi, Rafael Silveira Moreira, Negar Morovatdar, Shane Douglas Morrison, Vincent Mougin, George Duke Mukoro, Francesk Mulita, Erin C Mullany, Malaisamy Muniyandi, Yanjinlkham Munkhsaikhan, Efren Murillo-Zamora, Christopher J L Murray, Woojae Myung, Pirouz Naghavi, Ganesh R Naik, Soroush Najdaghi, Hastyar Hama Rashid Najmuldeen, Luigi Naldi, Gopal Nambi, Vinay Nangia, Jobert Richie Nansseu, Shumaila Nargus, Gustavo G Nascimento, Abdulqadir J Nashwan, Zuhair S Natto, Javaid Nauman, Muhammad Naveed, Nawsherwan, Biswa Prakash Nayak, Vinod C Nayak, Athare Nazri-Panjaki, Sabina Onyinye Nduaguba, Ruxandra Irina Negoi, Reza Nejad Shahrokh Abadi, Seyed Aria Nejadghaderi, Chakib Nejjari, Subas Neupane, Marie Ng, Josephine W Ngunjiri, Duc Hoang Nguyen, Hau Thi Hien Nguyen, Hien Quang Nguyen, Phat Tuan Nguyen, Phuong The Nguyen, Van Thanh Nguyen, Yeshambel T Nigatu, Taxiarchis Konstantinos Nikolouzakis, Ali Nikoobar, Nasrin Nikravangolsefid, Vikram Niranjan, Chukwudi A Nnaji, Lawrence Achilles Nnyanzi, Efaq Ali Noman, Shuhei Nomura, Syed Toukir Ahmed Noor, Mamoona Noreen, Majid Nozari, Fred Nugen, Chimezie Igwegbe Nzoputam, Ogochukwu Janet Nzoputam, Bogdan Oancea, Kehinde O Obamiro, Ismail A Odetokun, Daniel Bogale Odo Odo, Oluwakemi Ololade Odukoya, Michael Safo Oduro, James Odhiambo Oguta, In-Hwan Oh, Hassan Okati-Aliabad, Sylvester Reuben Okeke, Akinkunmi Paul Okekunle, Osaretin Christabel Okonji, Andrew T Olagunju, Omotola O Olasupo, Matthew Idowu Olatubi, Gláucia Maria Moraes Oliveira, Abdulhakeem Abayomi Olorukooba, Goran Latif Omer, Sok King Ong, Abdulahi Opejin Opejin, Michal Ordak, Verner N Orish, Esteban Ortiz-Prado, Uchechukwu Levi Osuagwu, Stanislav S Otstavnov, Amel Ouyahia, Mahesh Padukudru P A, Alicia Padron-Monedero, Jagadish Rao Padubidri, Anton Pak, Raul Felipe Palma-Alvarez, Hai-Feng Pan, Demosthenes Panagiotakos, Songhomitra Panda-Jonas, Anamika Pandey, Leonidas D Panos, Ioannis Pantazopoulos, Anca Pantea Stoian, Paraskevi Papadopoulou, Shahina Pardhan, Pragyan Paramita Parija, Romil R Parikh, Eun-Kee Park, Seoyeon Park, Nicholas Parsons, Roberto Passera, Jay Patel, Sangram Kishor Patel, Shankargouda Patil, Hridoy Patwary, Shrikant Pawar, Prince Peprah, Gavin Pereira, Arokiasamy Perianayagam, Richard G Pestell, Fanny Emily Petermann-Rocha, Tom Pham, Anil K Philip, Michael R Phillips, Dimitri Poddighe, Roman V Polibin, Ramesh Poluru, Fabio Porru, Akram Pourshams, Jalandhar Pradhan, Pranil Man Singh Pradhan, Manya Prasad, Akila Prashant, Elton Junio Sady Prates, Dimas Ria Angga Pribadi, Bharathi M Purohit, Jagadeesh Puvvula, Ibrahim Qattea, Venkatraman Radhakrishnan, Catalina Raggi, Pankaja Raghav, Fakher Rahim, Afarin Rahimi-Movaghar, Md Mosfequr Rahman, Mosiur Rahman, Muhammad Aziz Rahman, Shayan Rahmani, Mohammad Rahmanian, Nazanin Rahmanian, Vinoth Rajendran, Pushp Lata Rajpoot, Prashant Rajput, Pradhum Ram, Mahmoud Mohammed Ramadan, Majed Ramadan, Kritika Rana, Rishabh Kumar Rana, Chhabi Lal Ranabhat, Sowmya J Rao, Sina Rashedi, Ahmed Mustafa Rashid, Mohammad-Mahdi Rashidi, Ashkan Rasouli-Saravani, Devarajan Rathish, Santosh Kumar Rauniyar, Ilari Rautalin, Nakul Ravikumar, Salman Rawaf, Murali Mohan Rama Krishna Reddy, Elrashdy Moustafa Mohamed Redwan, Negar Rezaei, Mohsen Rezaeian, Abanoub Riad, Monica Rodrigues, Thales Philipe R Rodrigues da Silva, Jefferson Antonio Buendia Rodriguez, Leonardo Roever, Kevin T Root, Gholamreza Roshandel, Allen Guy Ross, Himanshu Sekhar Rout, Bedanta Roy, Nitai Roy, Simanta Roy, Guilherme de Andrade Ruela, Chandan S N, Cameron John Sabet, Siamak Sabour, Kabir P Sadarangani, Basema Ahmad Saddik, Masoumeh Sadeghi, Mohammad Reza Saeb, Umar Saeed, Pooya Saeedi, Sher Zaman Safi, Dominic Sagoe, Fatemeh Saheb Sharif-Askari, Amirhossein Sahebkar, Soumya Swaroop Sahoo, Md Refat Uz Zaman Sajib, Mirza Rizwan Sajid, Luciane B Salaroli, Mohamed A Saleh, Mohammed Z Y Salem, Dauda Salihu, Yoseph Leonardo Samodra, Abdallah M Samy, Juan Sanabria, Milena M Santric-Milicevic, Bruno Piassi Sao Jose, Muhammad Arif Nadeem Saqib, Made Ary Sarasmita, Aswini Saravanan, Babak Saravi, Yaser Sarikhani, Tanmay Sarkar, Gargi Sachin Sarode, Sachin C Sarode, Benn Sartorius, Brijesh Sathian, Anudeep Sathyanarayan, Maheswar Satpathy, Monika Sawhney, Mete Saylan, Benedikt Michael Schaarschmidt, Michael P Schaub, Markus P Schlaich, Maria Inês Schmidt, Art Schuermans, Austin E Schumacher, Siddharthan Selvaraj, Mohammad H Semreen, Subramanian Senthilkumaran, Sadaf G Sepanlou, Yashendra Sethi, Seyed Arsalan Seyedi, Allen Seylani, Mahan Shafie, Arman Shafiee, Ataollah Shahbandi, Samiah Shahid, Hamid R Shahsavari, Moyad Jamal Shahwan, Ahmed Shaikh, Masood Ali Shaikh, Ali S Shalash, Muhammad Aaqib Shamim, Anas Shamsi, Alfiya Shamsutdinova, Mohd Shanawaz, Abhishek Shankar, Mohammed Shannawaz, Medha Sharath, Amin Sharifan, Manoj Sharma, Ujjawal Sharma, Vishal Sharma, Aziz Sheikh, Ali Sheikhy, Mahabalesh Shetty, Pavanchand H Shetty, Premalatha K Shetty, Desalegn Shiferaw, Tariku Shimels, Rahman Shiri, Aminu Shittu, Ivy Shiue, Velizar Shivarov, Seyed Afshin Shorofi, Sunil Shrestha, Emmanuel Edwar Siddig, João Pedro Silva, Abhinav Singh, Baljinder Singh, Harmanjit Singh, Jasvinder A Singh, Paramdeep Singh, Puneetpal Singh, Surjit Singh, Virendra Singh, Freddy Sitas, Amanda E Smith, Matiwos Soboka, Ranjan Solanki, Marco Solmi, Soroush Soraneh, Joan B Soriano, Ireneous N Soyiri, Michael Spartalis, Chandrashekhar T Sreeramareddy, Panagiotis Stachteas, Dan J Stein, Paschalis Steiropoulos, Aleksandar Stevanović, Kurt Straif, Muhammad Suleman, Gerhard Sulo, Zhong Sun, Vinay Suresh, Chandan Kumar Swain, Lukasz Szarpak, Sree Sudha T Y, Payam Tabaee Damavandi, Ozra Tabatabaei Malazy, Seyed-Amir Tabatabaeizadeh, Celine Tabche, Jyothi Tadakamadla, Santosh Kumar Tadakamadla, Jabeen Taiba, Iman M Talaat, Ashis Talukder, Mircea Tampa, Jacques Lukenze JL Tamuzi, Ker-Kan Tan, Minale Tareke, Ingan Ukur Tarigan, Mojtaba Teimoori, Mohamad-Hani Temsah, Reem Mohamad Hani Temsah, Masayuki Teramoto, Dufera Rikitu Terefa, Pugazhenthan Thangaraju, Kavumpurathu Raman Thankappan, Rekha Thapar, Rasiah Thayakaran, Nikhil Kenny Thomas, Jansje Henny Vera Ticoalu, Krishna Tiwari, Roman Topor-Madry, Marcos Roberto Tovani-Palone, Khaled Trabelsi, An Thien Tran, Ngoc Ha Tran, Thang Huu Tran, Nguyen Tran Minh Duc, Indang Trihandini, Jaya Prasad Tripathy, Thien Tan Tri Tai Truyen, Evangelia Eirini Tsermpini, Abdul Rohim Tualeka, Aniefiok John Udoakang, Arit Udoh, Atta Ullah, Saeed Ullah, Muhammad Umair, Brigid Unim, Bhaskaran Unnikrishnan, Jibrin Sammani Usman, Sanaz Vahdati, Asokan Govindaraj Vaithinathan, Jef Van den Eynde, Constantine Vardavas, Tommi Juhani Vasankari, Siavash Vaziri, Balachandar Vellingiri, Narayanaswamy Venketasubramanian, Madhur Verma, Paul J Villeneuve, Manish Vinayak, Francesco S Violante, Sergey Konstantinovitch Vladimirov, Simona Ruxandra Volovat, Abdul Wadood, Yasir Waheed, Mandaras Tariku Walde, Shu Wang, Yanzhong Wang, Muhammad Waqas, Nuwan Darshana Wickramasinghe, Peter Willeit, Marcin W Wojewodzic, Asrat Arja Wolde, Tewodros Eshete Wonde, Hong Xiao, Suowen Xu, Mukesh Kumar Yadav, Kazumasa Yamagishi, Danting Yang, Lin Yang, Yuichiro Yano, Amir Yarahmadi, Renjulal Yesodharan, Saber Yezli, Xinglin Yi, Arzu Yiğit, Dehui Yin, Dong Keon Yon, Naohiro Yonemoto, Seok-Jun Yoon, Chuanhua Yu, Chun-Wei Yuan, Fathiah Zakham, Mohammed G M Zeariya, Haijun Zhang, Jianrong Zhang, Liqun Zhang, Claire Chenwen Zhong, Shang Cheng Zhou, Bin Zhu, Magdalena Zielińska, Ghazal Zoghi, Sa'ed H Zyoud, Stein Emil Vollset, Emmanuela Gakidou

**Affiliations:** a Institute for Health Metrics and Evaluation, University of Washington, Seattle, WA, USA; b Institute for Health Metrics and Evaluation, University of Washington, Seattle, WA, USA; c Department of Clinical Governance and Quality Improvement, Aleta Wondo General Hospital, Aleta Wondo, Ethiopia; d College of Pharmacy, Ajman University, Ajman, United Arab Emirates; e Department of Mathematics and Sciences, Ajman University, Ajman, United Arab Emirates; f Minimally Invasive Surgery Research Center, Iran University of Medical Sciences, Tehran, Iran; g Department of Medicine, University of Setif Algeria, Sétif, Algeria; h Department of Health, Sétif, Algeria; i National Institute of Epidemiology, Indian Council of Medical Research, Chennai, India; j Postgraduate Department, University of Sierra Sur, Miahuatlan de Porfirio Diaz, Mexico; k Yhteiskuntadatatieteen keskus (Centre for Social Data Science), University of Helsinki, Helsinki, Finland; l Department of Botany, Sree Narayana Guru College Chelannur, Kozhikode, India; m Department of Internal Medicine, Federal Medical Centre, Abuja, Nigeria; n Department of Community Medicine, Babcock University, Ilishan-Remo, Nigeria; o Department of Family and Community Health, University of Health and Allied Sciences, Ho, Ghana; p Department of Pediatric Dentistry of the School of Dentistry, Federal University of Minas Gerais, Belo Horizonte, Brazil; q Department of Anesthesiology, Shahid Beheshti University of Medical Sciences, Tehran, Iran; r Department of Nursing, Al Zaytoonah University of Jordan, Amman, Jordan; s Department of Pharmacology and Toxicology, Usmanu Danfodiyo University, Sokoto, Sokoto, Nigeria; t Nigerian Institute of Medical Research, Lagos, Nigeria; u Institute of Community and Public Health, Birzeit University, Ramallah, Palestine; v Department of Therapeutics, United Arab Emirates University, Al Ain, United Arab Emirates; w College of Pharmacy, University of Jordan, Amman, Jordan; x Department of Biochemistry and Molecular Medicine, Alfaisal University, Riyadh, Saudi Arabia; y College of Graduate Health Sciences, University of Tennessee, Memphis, TN, USA; z College of Medicine and Health Sciences, Bahir Dar University, Bahir Dar, Ethiopia; aa Department of Community Medicine, University of Ibadan, Ibadan, Nigeria; ab Department of Community Medicine, University College Hospital, Ibadan, Ibadan, Nigeria; ac Menzies School of Health Research, Charles Darwin University, Darwin, NT, Australia; ad Department of Obstetrics and Gynecology, University of Texas Medical Branch, Galveston, TX, USA; ae Department of Educational Counselling and Developmental Psychology, University of Ibadan, Ibadan, Nigeria; af Department of Educational Psychology, University of Johannesburg, Johannesburg, South Africa; ag Department of Public Health, Universitas Padjadjaran (Padjadjaran University), Bandung, Indonesia; ah Department of Epidemiology and Biostatistics, University of Health and Allied Sciences, Ho, Ghana; ai Department of Internal Medicine, Shahid Beheshti University of Medical Sciences, Tehran, Iran; aj Technical Services Directorate, MSI Nigeria Reproductive Choices, Abuja, Nigeria; ak Department of Life Sciences, University of Management and Technology, Lahore, Pakistan; al Department of Community Medicine, King Edward Memorial Hospital, Lahore, Pakistan; am Department of Public Health, Public Health Institute, Lahore, Pakistan; an Department of Medical and Surgical Sciences and Advanced Technologies “GF Ingrassia”, University of Catania, Catania, Italy; ao Department of Geography and Planning, Queen's University, Kingston, ON, Canada; ap School of Public Health, University of Technology Sydney, Sydney, NSW, Australia; aq Institute for Health Metrics and Evaluation, University of Washington, Seattle, WA, USA; ar Department of Applied Mathematics, University of Washington, Seattle, WA, USA; as Department of Medical Biochemistry, Shaqra University, Shaqra, Saudi Arabia; at School of Medicine and Psychology, Australian National University, Canberra, ACT, Australia; au Public Health Foundation of India, Gandhinagar, India; av Clinical Department, University of Jordan, Amman, Jordan; aw Department of Health and Biological Sciences, Abasyn University, Peshawar, Pakistan; ax Department of Natural Sciences, Lebanese American University, Beirut, Lebanon; ay Department of Medical Oncology, Miami Cancer Institute, Miami, FL, USA; az Department of Community Medicine and Preventive Health, King Edward Medical University Lahore, Lahore, Pakistan; ba Department of Epidemiology and Biostatistics, Shahrekord University of Medical Sciences, Shahrekord, Iran; bb Department of Epidemiology, Shahid Beheshti University of Medical Sciences, Tehran, Iran; bc Maternal and Child Health Division, International Centre for Diarrhoeal Disease Research, Bangladesh, Dhaka, Bangladesh; bd Department of Women's and Children's Health, Uppsala University, Uppsala, Sweden; be Institute of Endemic Diseases, University of Khartoum, Khartoum, Sudan; bf Swiss Tropical and Public Health Institute, University of Basel, Basel, Switzerland; bg Department of Biosciences, COMSATS Institute of Information Technology, Islamabad, Pakistan; bh Department of Epidemiology, Jimma University, Jimma, Ethiopia; bi College of Medicine and Public Health, Flinders University, Adelaide, SA, Australia; bj Department of Biochemistry, Jamia Hamdard, Delhi, India; bk National Nutrition and Food Technology Research Institute, Shahid Beheshti University of Medical Sciences, Tehran, Iran; bl Department of Cardiology, Fudan University, Shanghai, China; bm Maternal and Child Health Division, International Centre for Diarrhoeal Disease Research, Bangladesh, Dhaka, Bangladesh; bn Department of Communicable Diseases, Ministry of Health, Muscat, Oman; bo Middle East, Eurasia, and Africa Influenza Stakeholders Network, Muscat, Oman; bp Division of Public Health Sciences, Washington University in St. Louis, St. Louis, MO, USA; bq School of Medicine, New York Medical College, Valhalla, NY, USA; br Department of Epidemiology, Columbia University, New York, NY, USA; bs Department of Research and Development, Washington University in St. Louis, St. Louis, MO, USA; bt Clinical Epidemiology Center, US Department of Veterans Affairs (VA), St Louis, MO, USA; bu Murdoch Business School, Murdoch University, Perth, WA, Australia; bv School of Health and Environmental Studies, Hamdan Bin Mohammed Smart University, Dubai, United Arab Emirates; bw Division of Gastroenterology and Hepatology, Mayo Clinic, Jacksonville, FL, USA; bx Department of Bacteriology, Immunology, and Mycology, Suez Canal University, Ismailia, Egypt; by Global Centre for Environmental Remediation, University of Newcastle, Newcastle, NSW, Australia; bz Cooperative Research Centre for Contamination Assessment and Remediation of the Environment, Newcastle, NSW, Australia; ca Department of Cardiac Sciences, King Saud University, Riyadh, Saudi Arabia; cb College of Nursing, Qatar University, Doha, Qatar; cc Department of Health Services and Hospital Administration, King Abdulaziz University, Jeddah, Saudi Arabia; cd Health Economics Research Group, King Abdulaziz University, Jeddah, Saudi Arabia; ce Department of Zoology, Abdul Wali Khan University Mardan, Mardan, Pakistan; cf Department of Medical Rehabilitation (Physiotherapy), University of Maiduguri, Maiduguri, Nigeria; cg Department of Rehabilitation Sciences, Hong Kong Polytechnic University, Hong Kong, China; ch Department of Biosciences, Jamia Millia Islamia, New Delhi, India; ci Center for Biotechnology and Microbiology, University of Swat, Swat, Pakistan; cj Department of Geography, Sultan Qaboos University, Muscat, Oman; ck Institute of Health and Wellbeing, Federation University Australia, Melbourne, VIC, Australia; cl School of Public Health and Preventive Medicine, Monash University, Melbourne, VIC, Australia; cm Department of Public Health and Community Medicine, International Medical University, Kuala Lumpur, Malaysia; cn International Centre for Casemix and Clinical Coding, National University of Malaysia, Bandar Tun Razak, Malaysia; co Bordeaux School of Public Health, University of Bordeaux, Bordeaux, France; cp Department of Global Public Health, Karolinska Institutet, Stockholm, Sweden; cq Department of Cardiology, Heart, Vascular, and Thoracic Institute, Cleveland Clinic Abu Dhabi, Abu Dhabi, United Arab Emirates; cr College of Medicine and Health Sciences Academic Programs, Khalifa University, Abu Dhabi, United Arab Emirates; cs Department of Dentistry, Sana'a University, Sana'a, Yemen; ct Independent Consultant, Irbid, Jordan; cu College of Dental Medicine, Qatar University, Doha, Qatar; cv Department of Physical Therapy and Rehabilitation Sciences, Jordan University of Science and Technology, Irbid, Jordan; cw Department of Rehabilitation Sciences and Physical Therapy, Jordan University of Science and Technology, Irbid, Jordan; cx Department of Respiratory Care, Prince Sultan Military College of Health Sciences, Dammam, Saudi Arabia; cy Department of Prosthodontics and Implant Dentistry, Taibah University, Medinah, Saudi Arabia; cz Department of Prosthodontics and Implant Dentistry, Ibb University, Ibb, Yemen; da Department of Community Medicine, King Abdulaziz University, Jeddah, Saudi Arabia; db Macro-Fiscal Policy Department, Ministry of Finance, Dubai, United Arab Emirates; dc Internal Medicine Department, University of Missouri, Kansas, MO, USA; dd Department of Internal Medicine, Saint Luke's Mid America Heart Institute, Kansas, MO, USA; de Department of Family and Community Medicine, University of Jeddah, Jeddah, Saudi Arabia; df Department of Basic Sciences, Yarmouk University, Irbid, Jordan; dg Institute of Molecular Biology and Biotechnology, The University of Lahore, Lahore, Pakistan; dh Research Group in Health Economics, Universidad de Cartagena (University of Cartagena), Cartagena, Colombia; di Research Group in Hospital Management and Health Policies, Universidad de la Costa (University of the Coast), Barranquilla, Colombia; dj Department of Rehabilitation Sciences, Jordan University of Science and Technology, Irbid, Jordan; dk Department of Medical Sciences, Azal University for Human Development, Sana'a, Yemen; dl Department of Clinical Sciences, University of Science and Technology of Fujairah, Fujairah, United Arab Emirates; dm Department of Pediatrics, Cleveland Clinic, Cleveland, OH, USA; dn Department of Pediatric Cardiology, Boston Children's Hospital, Boston, MA, USA; do Department of Pediatrics, Harvard University, Boston, MA, USA; dp Faculty of Medicine, Jordan University of Science and Technology, Irbid, Jordan; dq Department of Pharmacy Practice and Pharmacotherapeutics, University of Sharjah, Sharjah, United Arab Emirates; dr Department of Clinical Pharmacy, Jordan University of Science and Technology, Irbid, Jordan; ds Department of Biomedical Engineering, German Jordanian University, Amman, Jordan; dt Interdisciplinary Graduate Program in Human Toxicology, University of Iowa, Iowa City, IA, USA; du Health Policy Research Center, Shiraz University of Medical Sciences, Shiraz, Iran; dv Public Health and Community Medicine Department, Cairo University, Cairo, Egypt; dw Quran and Hadith Research Center, Baqiyatallah University of Medical Sciences, Tehran, Iran; dx Department of Population and Behavioural Sciences, University of Health and Allied Sciences, Ho, Ghana; dy School of Government, Ateneo De Manila University, Quezon City, Philippines; dz Research for Impact, Singapore, Singapore; ea Department of Medicine, University of Jos, Jos, Nigeria; eb Department of Internal Medicine, Jos University Teaching Hospital, Jos, Nigeria; ec Clinical Studies & Trials Unit, Indian Council of Medical Research, Delhi, India; ed Centre for Sensorimotor Performance, The University of Queensland, Brisbane, QLD, Australia; ee Neurology Department, Royal Brisbane and Women's Hospital, Brisbane, QLD, Australia; ef Faculty of Medicine and Health, University of Sydney, Sydney, NSW, Australia; eg Institute for Health Metrics and Evaluation, University of Washington, Seattle, WA, USA; eh Department of Cardiology, Carol Davila University of Medicine and Pharmacy, Bucharest, Romania; ei Department of Statistics and Econometrics, Bucharest University of Economic Studies, Bucharest, Romania; ej School of Pharmacy, University of Nottingham Malaysia, Semenyih, Malaysia; ek Department of Microbiology, University of Medical Sciences, Ondo, Ondo, Nigeria; el Regenerative Medicine, Organ Procurement and Transplantation Multi-disciplinary Center, Guilan University of Medical Sciences, Rasht, Iran; em Department of Surgery, Gadjah Mada University, Yogyakarta, Indonesia; en Rural Health Research Institute, Charles Sturt University, Orange, NSW, Australia; eo Health Management and Economics Research Center, Iran University of Medical Sciences, Tehran, Iran; ep Department of Clinical Sciences, Ajman University, Ajman, United Arab Emirates; eq Department of Pharmacology and Toxicology, Beni-Suef University, Beni-Suef, Egypt; er Department of Applied Mathematics, University of Washington, Seattle, WA, USA; es Institute for Health Metrics and Evaluation, University of Washington, Seattle, WA, USA; et Department of Health Metrics Sciences, School of Medicine, University of Washington, Seattle, WA, USA; eu College of Art and Science, Ottawa University, Surprise, AZ, USA; ev School of Life Sciences, Arizona State University, Tempe, AZ, USA; ew College of Medicine and Health Sciences, Adigrat University, Adigrat, Ethiopia; ex Department of Public Health, Birmingham City University, Birmingham, UK; ey Institute for Biomedical Problems, Russian Academy of Sciences, Moscow, Russia; ez Department of Environmental Health, Mekelle University, Mekelle, Ethiopia; fa Cabrini Research, Cabrini Health, Malvern, VIC, Australia; fb School of Public Health and Preventative Medicine, Monash University, Melbourne, VIC, Australia; fc Department of Public Health, Jimma University, Jimma, Ethiopia; fd Department of Public Health, Wachemo University, Hossana, Ethiopia; fe University Institute of Radiological Sciences and Medical Imaging Technology, The University of Lahore, Lahore, Pakistan; ff School of Architecture, Design, and Planning, University of Sydney, Sydney, NSW, Australia; fg Department of Immunology, Zanjan University of Medical Sciences, Zanjan, Iran; fh School of Medicine and Public Health, University of Newcastle, Newcastle, NSW, Australia; fi Australian College of Applied Professions, Australian College of Applied Professions, Sydney, NSW, Australia; fj Department of Forensic Medicine, Lumbini Medical College, Palpa, Nepal; fk Northumbria HealthCare NHS Foundation Trust, Newcastle upon Tyne, UK; fl Department of Physiotherapy, Bayero University Kano, Kano, Nigeria; fm School of Nursing and Public Health, University of KwaZulu-Natal, Durban, South Africa; fn School of Indigenous Studies, The University of Western Australia, Perth, WA, Australia; fo School of Public Health, Curtin University, Perth, WA, Australia; fp Institute of Public Health, University of Gondar, Gondar, Ethiopia; fq Rural Health Research Institute, Charles Sturt University, Orange, NSW, Australia; fr Department of Surgery, Washington University in St. Louis, St. Louis, MO, USA; fs Leeds Institute of Rheumatic and Musculoskeletal Medicine, University of Leeds, Leeds, UK; ft Montefiore-Einstein Cerebrovascular Research Lab, Albert Einstein College of Medicine, Bronx, NY, USA; fu Faculty of Medicine, October 6 University, 6th of October City, Egypt; fv Department of Population Medicine, Qatar University, Doha, Qatar; fw Department of Biostatistics, Mashhad University of Medical Sciences, Mashhad, Iran; fx School of Medicine, Isfahan University of Medical Sciences, Isfahan, Iran; fy School of Public Affairs, Nanjing University of Information Science and Technology, Nanjing, China; fz Department of Forensic Medicine and Toxicology, Manipal Academy of Higher Education, Manipal, India; ga Division of Biological Sciences, Tamil Nadu State Council for Science and Technology, Chennai, India; gb Department of Medicine, Monash University, Clayton, VIC, Australia; gc Department of Hypertension, Medical University of Lodz, Lodz, Poland; gd Polish Mothers' Memorial Hospital Research Institute, Lodz, Poland; ge Nuffield Department of Surgical Sciences, University of Oxford, Oxford, NA, UK; gf Department of Neurosurgery, University of Southampton, Southampton, UK; gg Vocational School of Technical Sciences, Batman University, Batman, Turkiye; gh Department of Medical and Surgical Sciences and Advanced Technologies “GF Ingrassia”, University of Catania, Catania, Italy; gi Miller School of Medicine, University of Miami, Miami, FL, USA; gj School of Psychology, University of Auckland, Auckland, New Zealand; gk Department of Public and Environmental Health, University of The Gambia, Banjul, The Gambia; gl Department of Epidemiology, University of Florida, Gainesville, FL, USA; gm Department of Animal Sciences, Obafemi Awolowo University, Ile-Ife, Nigeria; gn Department of Veterinary Physiology and Biochemistry, University of Ilorin, Ilorin, Nigeria; go Non-communicable Diseases Research Center, Tehran University of Medical Sciences, Tehran, Iran; gp School of Medicine, Iran University of Medical Sciences, Tehran, Iran; gq Center for Primary Care, Harvard University, Boston, MA, USA; gr School of Public Health, Imperial College London, London, UK; gs Department of Academics, Indian Institute of Public Health, Gurgaon, India; gt Department of Medical Education, University of Nevada Las Vegas, Las Vegas, NV, USA; gu Health Human Resources Research Center, Shiraz University of Medical Sciences, Shiraz, Iran; gv School of Medicine, Tehran University of Medical Sciences, Tehran, Iran; gw Non-Communicable Diseases Research Center (NCDRC), Tehran, Iran; gx School of the Environment, Yale University, New Haven, CT, USA; gy School of Health Policy and Management, Korea University, Seoul, South Korea; gz Faculty of Pharmacy, Department of Biological Sciences, University of Porto, Porto, Portugal; ha Research Unit on Applied Molecular Biosciences (UCIBIO), University of Porto, Porto, Portugal; hb College of Medicine, University of Florida, Gainesville, FL, USA; hc Nuffield Department of Population Health, University of Oxford, Oxford, UK; hd Department of Internal Medicine, University of São Paulo, São Paulo, Brazil; he School of Medicine, Indiana University, Indianapolis, IN, USA; hf Department of Epidemiology and Biostatistics, University of the Philippines Manila, Manila, Philippines; hg Department of Epidemiology, Brown University, Providence, RI, USA; hh Metabolomics Laboratory, Baker Heart and Diabetes Institute, Melbourne, VIC, Australia; hi Department of Microbiology, Addis Ababa University, Addis Ababa, Ethiopia; hj Department of Forensic Chemistry, Government Institute of Forensic Science, Aurangabad, Aurangabad, India; hk Department of Public Health, North Dakota State University, Fargo, ND, USA; hl Division of Gastroenterology and Hepatology, Mayo Clinic, Jacksonville, FL, USA; hm Institute of Applied Health Research, University of Nottingham, Nottingham, UK; hn Institute of Applied Health Research, University of Birmingham, Birmingham, UK; ho Department of Anatomy, All India Institute of Medical Sciences, Jodhpur, India; hp Department of Community Medicine and Family Medicine, All India Institute of Medical Sciences, Jodhpur, India; hq School of Public Health, All India Institute of Medical Sciences, Jodhpur, India; hr Global Health Neurology Lab, NSW Brain Clot Bank, Sydney, NSW, Australia; hs Division of Cerebrovascular Medicine and Neurology, National Cerebral and Cardiovascular Center, Suita, Japan; ht Department of General Medicine, Manipal Academy of Higher Education, Mangalore, India; hu Institute for Health Metrics and Evaluation, University of Washington, Seattle, WA, USA; hv Newcastle University, Newcastle upon Tyne, UK; hw Department of Clinical Medicine, Cambridge University Hospitals NHS Foundation Trust, Cambridge, UK; hx Department of Human Genetics and Molecular Medicine, Central University of Punjab, Bathinda, India; hy Department of Radiology, Mayo Clinic College of Medicine, Rochester, MN, USA; hz Neurovascular Research Laboratory, Mayo Clinic College of Medicine, Rochester, MN, USA; ia Department of Neurology, Institute of Post-Graduate Medical Education and Research and Seth Sukhlal Karnani Memorial Hospital, Kolkata, India; ib Department of Community Medicine and Family Medicine, All India Institute of Medical Sciences, Deoghar, India; ic SAMRC/Wits Centre for Health Economics and Decision Science - PRICELESS SA, University of the Witwatersrand, Johannesburg, South Africa; id SAMRC Centre for Health Economics and Decision Science, University of the Witwatersrand, Johannesburg, South Africa; ie Department of Health Promotion and Behavioural Science, Bahir Dar University, Bahir Dar, Ethiopia; if General Directorate of Health Information Systems, Ministry of Health, Ankara, Turkiye; ig Internal Medicine Department, Shahid Beheshti University of Medical Sciences, Tehran, Iran; ih Disease Surveillance Department, Ghana Health Service, Ho, Ghana; ii Department of Epidemiology and Biostatistics, University of Health and Allied Sciences, Ho, Ghana; ij Department of Medicine, University Ferhat Abbas of Setif, Setif, Algeria; ik Department of Epidemiology and Preventive Medicine, University Hospital Saadna Abdenour, Setif, Algeria; il School of Medicine, University of Washington, Seattle, WA, USA; im General Medicine Service, Department of Veterans Affairs, Seattle, WA, USA; in Division of Clinical Epidemiology and Aging Research, German Cancer Research Center, Heidelberg, Germany; io Department of Internal Medicine, University of São Paulo, São Paulo, Brazil; ip Department of Psychiatry, University of São Paulo, São Paulo, Brazil; iq Department of Medical and Surgical Sciences, University of Bologna, Bologna, Italy; ir Flinders Health and Medical Research Institute, Flinders University, Adelaide, SA, Australia; is School of Pharmacy, The University of Jordan, Amman, Jordan; it Department of Basic Biomedical Sciences, University of Sharjah, Sharjah, United Arab Emirates; iu School of Public Health Sciences, University of Waterloo, Waterloo, ON, Canada; iv Al Shifa School of Public Health, Al Shifa Trust Eye Hospital, Rawalpindi, Pakistan; iw Harvard Business School, Harvard University, Boston, MA, USA; ix Department of Clinical Pharmacy, University of Medicine and Pharmacy of Craiova, Romania, Craiova, Romania; iy Dana-Farber Cancer Institute, Harvard University, Boston, MA, USA; iz Department of Ophthalmology, Beijing Institute of Ophthalmology, Beijing, China; ja Department of Health Management (Direzione Sanitaria), IRCCS Istituto Ortopedico Rizzoli, Bologna, Italy; jb Interdisciplinary Research Center for Health Science, Sant'Anna School of Advanced Studies, Pisa, Italy; jc Department of Health Care, Metropolitan Autonomous University, Mexico City, Mexico; jd Institute for Cancer Research, Prevention and Clinical Network, Florence, Italy; je Department of Psychiatry, University of São Paulo, São Paulo, Brazil; jf Department of Public Health and Infectious Diseases, La Sapienza University, Rome, Italy; jg Department of Psychiatry, University of São Paulo, São Paulo, Brazil; jh Department of Psychiatry, Federal University of Rio Grande do Sul, Porto Alegre, Brazil; ji Department of Medical, Surgical, and Health Sciences, University of Trieste, Trieste, Italy; jj Public Health Unit, University Health Agency Giuliano-Isontina (ASUGI), Trieste, Italy; jk Department of Medical and Surgical Sciences, University of Bologna, Bologna, Italy; jl Institute of Clinical Physiology, Italian National Council of Research, Pisa, Italy; jm State Disease Investigation Laboratory, Animal Resources Development Department, Agartala, India; jn Department of Clinical Nutrition, Jazan University, Jazan, Saudi Arabia; jo Department of Anesthesiology and Perioperative Medicine, University of Rochester, Rochester, NY, USA; jp Temerty Faculty of Medicine, University of Toronto, Toronto, ON, Canada; jq Department of Community Medicine, Datta Meghe Institute of Medical Sciences, Sawangi, India; jr Department of Biology, Al-Imam Mohammad Ibn Saud Islamic University, Riyadh, Saudi Arabia; js Oral Medicine and Radiology, King George's Medical University, Lucknow, India; jt Fuwai Hospital, Chinese Academy of Medical Sciences & Peking Union Medical College, Beijing, China; ju Department of Computer Science, University of Texas at Austin, Austin, TX, USA; jv Department of Stomatology, Huazhong University of Science and Technology, Wuhan, China; jw Hubei Province Key Laboratory of Oral and Maxillofacial Development and Regeneration, Wuhan, China; jx Clinical Research Center, Zhujiang Hospital of Southern Medical University, Guangzhou, China; jy University of Michigan, Ann Arbor, MI, USA; jz Heidelberg Institute of Global Health (HIGH), Heidelberg University, Heidelberg, Germany; ka Center for Healthy Aging, Pennsylvania State University, University Park, PA, USA; kb Social Sciences Division, Syracuse University, Syracuse, NY, USA; kc Division of Cardiovascular Medicine, Harvard University, Boston, MA, USA; kd Department of Scientific Research, Tehran University of Medical Sciences, Tehran, Iran; ke Concord Institute of Academic Surgery, Sydney Local Health District, Sydney, NSW, Australia; kf Concord Clinical School, University of Sydney, Sydney, NSW, Australia; kg Department of Public Health, Administration, and Social Sciences, Cayetano Heredia University, Lima, Peru; kh Iraq Field Epidemiology Training Program (I-FETP), Ministry of Health, Baghdad, Iraq; ki Department of Medicine, National University of Singapore, Singapore, Singapore; kj RIPAS Hospital, University of Brunei Darussalam, BSB, Brunei; kk The Nethersole School of Nursing, The Chinese University of Hong Kong, Hong Kong, China; kl Centre for Research Impact & Outcome, Chitkara University, Rajpura, India; km Department of Community Medicine, Jawaharlal Nehru Medical College, Wardha, India; kn Center for Biomedicine and Community Health, International School, Vietnam National University Hanoi (VNUIS), Hanoi, Vietnam; ko Department of Paediatric Surgery, Federal Medical Centre, Umuahia, Nigeria; kp Department of Health Informatics, University College London, London, UK; kq Health Data Research UK, London, UK; kr School of Nursing and Midwifery, University of Technology Sydney, Sydney, NSW, Australia; ks Nova Medical School, Nova University of Lisbon, Lisbon, Portugal; kt Department of Respiratory Medicine and Allergology, Nicolae Testemitanu State University of Medicine and Pharmacy, Chisinau, Moldova; ku Department of Family Medicine and Public Health, University of California San Diego, La Jolla, CA, USA; kv Department of Diagnostic and Therapeutic Technologies, Cooperativa de Ensino Superior Politécnico e Universitário (Polytechnic and University Higher Education Cooperative), Vila Nova de Famalicão, Portugal; kw Institute for Research and Innovation in Health (i3S), University of Porto, Porto, Portugal; kx School of Nursing, Federal University of Minas Gerais, Belo Horizonte, Brazil; ky Department of Global Public Health and Primary Care, University of Bergen, Bergen, Norway; kz Iranian Research Center for HIV/AIDS (IRCHA), Tehran University of Medical Sciences, Tehran, Iran; la Department of Paediatrics, The Chinese University of Hong Kong, Hong Kong, China; lb School of Clinical Medicine, Hangzhou Normal University, Hangzhou, China; lc Institute for Health Metrics and Evaluation, University of Washington, Seattle, WA, USA; ld Department of Health Metrics Sciences, School of Medicine, University of Washington, Seattle, WA, USA; le IRCCS Istituto Ortopedico Galeazzi (Galeazzi Orthopedic Institute IRCCS), University of Milan, Milan, Italy; lf Department of Dermatology, Case Western Reserve University, Cleveland, OH, USA; lg Public Health Foundation of India, Gurugram, India; lh Institute for Health Metrics and Evaluation, University of Washington, Seattle, WA, USA; zzp Indian Council of Medical Research, New Delhi, India; li Public Health Foundation of India, Gurugram, India; lj Institute for Health Metrics and Evaluation, University of Washington, Seattle, WA, USA; zzq Department of Health Metrics Sciences, School of Medicine, University of Washington, Seattle, WA, USA; lk Department of Public Health, Haramaya University, Harar, Ethiopia; ll Environmental Health, Arak University of Medical Sciences, Arak, Iran; lm Department of Biochemistry, Ministry of Health and Welfare, New Delhi, India; ln Clinical Sciences Department, University of Sharjah, Sharjah, United Arab Emirates; lo Health Research Institute, Kazakh National Medical University, Almaty, Kazakhstan; lp Department of Health Policy and Management, Haramaya University, Harar, Ethiopia; lq Medical College, Albany Medical College, Albany, NY, USA; lr School of Public Health, University of Technology Sydney, Sydney, NSW, Australia; ls School of Public Health and Social Work, Queensland University of Technology, Brisbane, QLD, Australia; lt Department of Research, Gujarat Adani Institute of Medical Sciences and G.K. General Hospital, Bhuj, India; lu JSS Medical College Department of Biochemistry, Jagadguru Sri Shivarathreeswara University, Mysuru, India; lv Department of Pharmacy, United International University, Dhaka, Bangladesh; lw Pharmacology Division, Center for Life Sciences Research Bangladesh, Dhaka, Bangladesh; lx Sheffield Teaching Hospitals NHS Foundation Trust, Sheffield, UK; ly Research and Development Cell, Dr. D. Y. Patil University, Pune, India; lz The Zena and Michael A. Wiener Cardiovascular Institute, Icahn School of Medicine at Mount Sinai, New York, NY, USA; ma Department of Medicine, Pham Ngoc Thach University of Medicine, Ho Chi Minh City, Vietnam; mb School of Medicine, Iran University of Medical Sciences, Tehran, Iran; mc Institute of Health Research, University of Health and Allied Sciences, Ho, Ghana; md Department of Epidemiology, Jazan University, Jazan, Saudi Arabia; me Health Science Center, University of Texas, Houston, TX, USA; mf Department of Medical, Surgical, and Health Sciences, University of Trieste, Trieste, Italy; mg Cardio-Thoraco-Vascular Department, Azienda Sanitaria Universitaria Giuliano Isontina, Trieste, Italy; mh Independent Consultant, South Plainfield, NJ, USA; mi Department of Cardiology, Hackettstown Medical Center, Hackettstown, NJ, USA; mj Newton Medical Center, Sparta, NJ, USA; mk Department of Epidemiology and Biostatistics, University of Health and Allied Sciences, Ho, Ghana; ml Department of Medicine, Bangalore Medical College and Research Institute, Bangalore, India; mm Manipal Academy of Higher Education, Manipal, India; mn Department of Forensic Medicine and Toxicology, Kasturba Medical College Mangalore, Mangalore, India; mo Faculty of Health, Medicine and Life Sciences (FHML), Maastricht University, Maastricht, Netherlands; mp Postgraduate Program in Epidemiology, Federal University of Rio Grande do Sul, Porto Alegre, Brazil; mq School of Medicine, Federal University of Bahia, Salvador, Brazil; mr Department of Internal Medicine, Escola Bahiana de Medicina e Saúde Pública (Bahiana School of Medicine and Public Health), Salvador, Brazil; ms Department of Conservative Dentistry with Endodontics, Medical University of Silesia, Katowice, Poland; mt Department of Pediatrics, Cleveland Clinic, Cleveland, OH, USA; mu Department of Orthopaedic Surgery, Massachusetts General Hospital, Boston, MA, USA; mv Cardiovascular Department, Tehran University of Medical Sciences, Tehran, Iran; mw Department of Behavioral and Community Health, University of Maryland, College Park, MD, USA; mx School of Nursing and Midwifery, La Trobe University, Melbourne, VIC, Australia; my School of Nursing and Midwifery, La Trobe University, Bundoora, VIC, Australia; mz Advanced Nursing Department, Universitas Airlangga (Airlangga University), Surabaya, Indonesia; na Research Center for Public Health and Nutrition, National Research and Innovation Agency Republic of Indonesia (BRIN), Jakarta, Indonesia; nb Iran University of Medical Sciences, Iran University of Medical Sciences, Tehran, Iran; nc Faculty of Science and Health, University of Portsmouth, Hampshire, UK; nd Almoosa College of Health Sciences, Al Ahsa, Saudi Arabia; ne Department of Public Health and Community Medicine, Tanta University, Tanta city, Egypt; nf School of Public Health, Texila American University, Guyana, Guyana; ng Biomedical Informatics and Medical Statistics Department, Alexandria University, Alexandria, Egypt; nh Institute of Public Health, United Arab Emirates University, Al Ain, United Arab Emirates; ni Faculty of Medicine, University of Tripoli, Tripoli, Libya; nj Houston Methodist Hospital, Houston, TX, USA; nk Department of Basic Medical Sciences, University of Sharjah, Sharjah, United Arab Emirates; nl Egypt Center for Research and Regenerative Medicine (ECRRM), Cairo, Egypt; nm Department of Neuropsychiatry, Ain Shams University, Cairo, Egypt; nn Executive Committee, International Association for Women Mental Health, Potomac, MD, USA; no Department of Infectious Diseases and Public Health, City University of Hong Kong, Hong Kong, China; np Department of Animal Medicine, Zagazig University, Zagazig, Egypt; nq Department of Pediatrics, Texas A&M University, Dallas, TX, USA; nr Department of Education, Ajman University, Ajman, United Arab Emirates; ns National Eye Institute, National Institute of Health, Bethesda, MD, USA; nt Department of Public Health and Tropical Medicine, James Cook University, Townsville, QLD, Australia; nu Health Economics and Financing Practice Area, Management Sciences for Health, Arlington, VA, USA; nv Department of Internal Medicine, Yale University, New Haven, CT, USA; nw Drexel University Dornsife School of Public Health, Drexel University, Philadelphia, PA, USA; nx Independent Consultant, Bologna, Italy; ny Department of Epidemiology and Medical Statistics, University of Ibadan, Ibadan, Nigeria; nz Research Centre for Healthcare and Community, Coventry University, Coventry, UK; oa Department of Periodontology and Community Dentistry, University of Ibadan, Ibadan, Nigeria; ob Department of Periodontology and Community Dentistry, University College Hospital, Ibadan, Ibadan, Nigeria; oc Interdisciplinary Neuroscience Research Program, Tehran University of Medical Sciences, Tehran, Iran; od Department of Oral Biology, Riphah International University, Islamabad, Pakistan; oe Director of the Scientific and Technological Park, Kazakh National Medical University, Almaty, Kazakhstan; of Department of Biomedical and Biotechnological Sciences, University of Catania, Catania, Italy; og Epidemiology and Biostatistics Unit, IRCCS Pascale, Naples, Italy; oh Department of Family Medicine, Luton & Dunstable University Hospital, Luton, UK; oi School of Engineering, Edith Cowan University, Joondalup, Western Australia (WA), Australia; oj University Institute of Radiological Sciences and Medical Imaging Technology, The University of Lahore, Lahore, Pakistan; ok Centre for Public Health, Equity and Human Flourishing, Torrens University Australia, Adelaide, SA, Australia; ol Institute of Resource Governance and Social Change, Kupang, Indonesia; om Laboratory of Experimental Medicine, Kazakh National Medical University, Almaty, Kazakhstan; on Department of Social Medicine and Epidemiology, Guilan University of Medical Sciences, Rasht, Iran; oo Department of Public Health and Infectious Diseases, City University of Hong Kong, Hong Kong, China; op Department of Pharmacy, Wollega University, Nekemte, Ethiopia; oq School of Population Health, University of New South Wales, Sydney, NSW, Australia; or National Institute of Environmental Health, Chinese Center for Disease Control and Prevention, Beijing, China; os Center for Public Health Research, University of Milan Bicocca, Monza, Italy; ot Laboratory of Public Health, IRCCS Istituto Auxologico Italiano, Milan, Italy; ou Department of Social Sciences, University of Nicosia, Nicosia, Cyprus; ov Institute of Health Sciences, Wollega University, Nekemte, Ethiopia; ow Jimma University, Jimma, Ethiopia; ox School of Public Health, Imperial College London, London, UK; oy Institute of Public Health, Charité Universitätsmedizin Berlin (Charité Medical University Berlin), Berlin, Germany; oz Institute for Health Metrics and Evaluation, University of Washington, Seattle, WA, USA; pa Department of Health Metrics Sciences, School of Medicine, University of Washington, Seattle, WA, USA; pb Institute of Gerontology, National Academy of Medical Sciences of Ukraine, Kyiv, Ukraine; pc Associate Laboratory Institute for Health and Bioeconomy (i4HB), University of Porto, Porto, Portugal; pd Faculty of Engineering, University of Porto, Porto, Portugal; pe Department of Neuroscience, Multiple Sclerosis Research Center, Ravenna, Italy; pf Department of Biotechnological and Applied Clinical Sciences, University of L'Aquila, L'Aquila, Italy; pg Department of Community Medicine and Family Medicine, All India Institute of Medical Sciences, Gorakhpur, India; ph Health Services Management Training Centre, Semmelweis University, Budapest, Hungary; pi Department of Applied Social Sciences, Sapientia Hungarian University of Transylvania, Târgu-Mureş, Romania; pj Department of Community Medicine, Bayero University Kano, Kano, Nigeria; pk Department of Community Medicine, Aminu Kano Teaching Hospital, Kano, Nigeria; pl Department of Community Medicine, Datta Meghe Institute of Medical Sciences, Wardha, India; pm Department of Oral Biology and Experimental Dental Research, University of Szeged, Szeged, Hungary; pn Department of Medical Epidemiology, Mario Negri Institute for Pharmacological Research, Milan, Italy; po Department of Community Medicine and Family Medicine, All India Institute of Medical Sciences, Nagpur, India; pp Institute of Health and Wellbeing, Federation University Australia, Churchill, VIC, Australia; pq Professional Services Division, Texas State Board of Pharmacy, Austin, TX, USA; pr Department of Pharmacology, Indore Institute of Pharmacy, Indore, India; ps Department of Midwifery, Adigrat University, Adigrat, Ethiopia; pt Department of Environmental Health, Wollo University, Dessie, Ethiopia; pu Department of Reproductive and Family Health, Axum College of Health Science, Axum, Ethiopia; pv College of Medicine and Public Health, Flinders University, Adelaide, SA, Australia; pw Department of Public Health, Debre Berhan University, Debre Berhan, Ethiopia; px Psychiatric Nursing and Management Department, Shahid Beheshti University of Medical Sciences, Tehran, Iran; py Tropical Health Department, Alexandria University, Alexandria, Egypt; pz Family and Community Medicine Department, King Khalid University, Abha, Saudi Arabia; qa Department of Radiology, University of Southern California, Los Angeles, CA, USA; qb Orthodontics Department, Mashhad University of Medical Sciences, Mashhad, Iran; qc Departments of Radiology and Neurosurgery, Mayo Clinic, Rochester, MN, USA; qd Country Office, World Health Organization (WHO), Astana, Kazakhstan; qe Institute for Health Metrics and Evaluation, University of Washington, Seattle, WA, USA; qf Third Department of Neurology, Research Center of Neurology, Moscow, Russia; qg Department of Community Medicine & School of Public Health, Post Graduate Institute of Medical Education and Research, Chandigarh, India; qh College of Human and Health Sciences, Swansea University, Swansea, UK; qi Preventive Medicine and Public Health Research Center, Iran University of Medical Sciences, Tehran, Iran; qj Department of Dermatology, Yale University, New Haven, CT, USA; qk Department of Health Systems and Policy Research, Indian Institute of Public Health, Gandhinagar, India; ql Department of Genetics, Sana Institute of Higher Education, Sari, Iran; qm Universal Scientific Education and Research Network (USERN), Kermanshah University of Medical Sciences, Kermanshah, Iran; qn Department of Life Sciences, Health and Healthcare Professions, Link Campus University, Rome, Italy; qo Health Services Research, Evaluation and Policy Unit, AUSL della Romagna, Ravenna, Italy; qp Oncological Network, Prevention and Research Institute, Institute for Cancer Research, Prevention and Clinical Network, Florence, Italy; qq School of Medicine, Tehran University of Medical Sciences, Tehran, Iran; qr Department of Dermatology, Case Western Reserve University, Cleveland, OH, USA; qs Institute of Public Health, United Arab Emirates University, Al Ain, United Arab Emirates; qt Department of Public Health and Preventive Medicine, Charles University, Prague, Czech Republic; qu Department of Preventive Oncology, Centre for Health Innovation and Policy (CHIP) Foundation, Noida, India; qv School of Health System Studies, Tata Institute of Social Sciences, Mumbai, India; qw Department of Epidemiology and Biostatistics, Anhui Medical University, Hefei, China; qx Department of Clinical Science, University of Sulaimani, Sulaimani, Iraq; qy Harrington Heart and Vascular Institute, Case Western Reserve University, Cleveland, OH, USA; qz Division of Cardiovascular Medicine, Ohio State University, Columbus, OH, USA; ra Health Directorate, Local Health Authority of Bologna, Bologna, Italy; rb Department of Biomedical and Neuromotor Sciences, University of Bologna, Bologna, Italy; rc Department of Thoracic Surgery, Cleveland Clinic, Cleveland, OH, USA; rd Department of Community Medicine, University of Peradeniya, Kandy, Sri Lanka; re Department of Psychiatry, Bronxcare Health System, Bronx, NY, USA; rf Department of Psychiatry, Icahn School of Medicine at Mount Sinai, New York, NY, USA; rg Group Health Department, Nanyang Central Hospital, Nanyang, China; rh Department of Nephrology, Max Super Specialty Hospital, New Delhi, India; ri Non-communicable Diseases Division (NCD), Indian Council of Medical Research, New Delhi, India; rj Department of Public Health, Torrens University Australia, Melbourne, VIC, Australia; rk Independent Consultant, Bharatpur, India; rl Independent Consultant, Delhi, India; rm DeBakey Heart and Vascular Center, Houston Methodist Hospital, Houston, TX, USA; rn Department of Preventive Cardiology & Medicine, Eternal Heart Care Centre & Research Institute, Jaipur, India; ro Department of Medicine, Mahatma Gandhi University Medical Sciences, Jaipur, India; rp Department of Toxicology, Shriram Institute for Industrial Research, Delhi, India; rq School of Medicine, Deakin University, Geelong, VIC, Australia; rr Department of Anthropology, University of Delhi, Delhi, India; rs Faculty of Medicine Health and Human Sciences, Macquarie University, Sydney, NSW, Australia; rt Department of Health in Emergencies and Disasters, Tehran University of Medical Sciences, Tehran, Iran; ru Department of Clinical Pharmacology and Medicine, University of Kufa, Najaf, Iraq; rv American University of Beirut, Faculty of Medicine, American University of Beirut, Beirut, Lebanon; rw Department of Maxillofacial Surgery and Diagnostic Sciences, Jazan University, Jazan, Saudi Arabia; rx Biochemistry Department, Ain Shams University, Cairo, Egypt; ry School of Health and Environmental Studies, Hamdan Bin Mohammed Smart University, Dubai, United Arab Emirates; rz Department of Medical and Technical Information Technology, Bauman Moscow State Technical University, Moscow, Russia; sa Centre for Neuromuscular and Neurological Disorders, The University of Western Australia, Perth, WA, Australia; sb Stroke Research Centre, Perron Institute for Neurological and Translational Science, Perth, WA, Australia; sc Department of Epidemiology Population Biostatistics and Health Promotion, Universitas Airlangga (Airlangga University), Surabaya, Indonesia; sd Research Unit, Parc Sanitari Sant Joan de Deu, Barcelona, Spain; se Department of Mental Health, Biomedical Research Networking Center for Mental Health Network (CiberSAM), Madrid, Spain; sf Department of Zoology and Entomology, Al-Azhar University, Cairo, Egypt; sg Department of Nursing, Taipei Medical University, Taipei, Taiwan; sh Department of Health Research Methods, Evidence and Impact, McMaster University, Hamilton, ON, Canada; si Department of Biochemistry and Molecular Biology, Tejgaon College, Dhaka, Bangladesh; sj Department of Pharmacy, Palamau Institute of Pharmacy, Daltonganj, India; sk Department of Neurology, Cairo University, Cairo, Egypt; sl Public Health Department, Dalhatu Araf Specialist Hospital, Lafia, Nigeria; sm Department of Public Health, Federal University of Lafia, Lafia, Nigeria; sn Center for International Health (CIH), University of Bergen, Bergen, Norway; so Bergen Center for Ethics and Priority Setting (BCEPS), University of Bergen, Bergen, Norway; sp Institute for Health Metrics and Evaluation, University of Washington, Seattle, WA, USA; sq Department of Health Metrics Sciences, School of Medicine, University of Washington, Seattle, WA, USA; sr Alberta Respiratory Centre (ARC), University of Alberta, Edmonton, AB, Canada; ss Community-Oriented Nursing Midwifery Research Center, Shahrekord University of Medical Sciences, Shahrekord, Iran; st Department of Medicine, MedStar Health, Washington, DC, USA; su Department of Medicine, Georgetown University, Washington, DC, USA; sv School of Public Health, Curtin University, Perth, WA, Australia; sw Department of Statistics and Econometrics, Bucharest University of Economic Studies, Bucharest, Romania; sx Babes-Bolyai University, Cluj-Napoca, Romania; sy Department of Public Health, Madda Walabu University, Robe, Ethiopia; sz Department of Microbiology, Taiz University, Taiz, Yemen; ta School of Medicine, Nankai University, Tianjin, China; tb Graduate School of Medicine, University of Tokyo, Tokyo, Japan; tc School of Dentistry, Hanoi Medical University, Hanoi, Vietnam; td Kasturba Medical College, Mangalore, Manipal Academy of Higher Education, Manipal, India; te Department of Pulmonology, Yokohama City University, Yokohama, Japan; tf National Human Genome Research Institute (NHGRI), National Institutes of Health, Bethesda, MD, USA; tg Department of Decision and Information Sciences, University of Houston, Houston, TX, USA; th Public Health Research Group, Nature Study Society of Bangladesh, Khulna, Bangladesh; ti Department of Behavioural Science and Health, University College London, London, UK; tj Department of Public Health and Informatics, Jahangirnagar University, Dhaka, Bangladesh; tk School of Health and Society, University of Wollongong, Wollongong, NSW, Australia; tl Department of Internal Medicine, Carol Davila University of Medicine and Pharmacy, Bucharest, Romania; tm Department of Legal Medicine and Bioethics, Carol Davila University of Medicine and Pharmacy, Bucharest, Romania; tn Department of Clinical Legal Medicine, National Institute of Legal Medicine Mina Minovici, Bucharest, Romania; to Faculty of Medicine, The Chinese University of Hong Kong, Hong Kong, China; tp Department of Public Health and Community Medicine, Shaikh Zayed Postgraduate Medical Institute, Lahore, Pakistan; tq Department of Biological Sciences and Chemistry, University of Nizwa, Nizwa, Oman; tr Department of Occupational Safety and Health, China Medical University, Taiwan, Taichung, Taiwan; ts Department of Occupational Therapy, Asia University, Taiwan, Taichung, Taiwan; tt Department of Health Promotion and Education, University of Ibadan, Ibadan, Nigeria; tu International Center for Nutrition and Information, National Institutes of Biomedical Innovation, Health and Nutrition, Tokyo, Japan; tv The National Centre for Remote and Rural Health and Care, NHS National Services Scotland, Edinburgh, Scotland; tw West Africa RCC, Africa Centre for Disease Control and Prevention, Abuja, Nigeria; tx Department of Community Medicine, University College Hospital, Ibadan, Ibadan, Nigeria; ty Faculty of Medicine, University of Belgrade, Belgrade, Serbia; tz Faculty of Medical Sciences, University of Kragujevac, Kragujevac, Serbia; ua Institute of Health Research, University of Health and Allied Sciences, Ho, Ghana; ub Department of Health Research, ICMR National Institute for Research in Tuberculosis, Chennai, India; uc Faculty of Public Health, Universitas Muhammadiyah Aceh, Banda Aceh, Indonesia; ud Faculty of Pharmacy, Universitas Ahmad Dahlan, Yogyakarta, Indonesia; ue School of Pharmacy, BRAC University, Dhaka, Bangladesh; uf Institute for Physical Activity and Nutrition, Deakin University, Burwood, VIC, Australia; ug Sydney Medical School, University of Sydney, Sydney, NSW, Australia; uh Department of Surveillance and Health Equity Science, American Cancer Society, Atlanta, GA, USA; ui Department of General Surgery and Medical-Surgical Specialties, University of Catania, Catania, Italy; uj School of Management, The Apollo University, Chittoor, India; uk Department of Health Services Research, University of Tsukuba, Tsukuba, Japan; ul Department of Non-Communicable Disease Epidemiology, London School of Hygiene & Tropical Medicine, London, UK; um Department of Microbiology, Central University of Punjab, Bathinda, India; un Department of Community Medicine and Family Medicine, All India Institute of Medical Sciences, Gorakhpur, India; uo Department of Environmental Health Engineering, Guilan University of Medical Sciences, Rasht, Iran; up Department of Physical and Medicine, Université Paris Cité, Paris, France; uq Research and Development Unit, Biomedical Research Networking Center for Mental Health Network (CiberSAM), Barcelona, Spain; ur Department of Immunology, Kerman University of Medical Sciences, Kerman, Iran; us Department of Immunology, Rafsanjan University of Medical Sciences, Rafsanjan, Iran; ut Department of Nephrology, San Mateo Medical Center, San Mateo, CA, USA; uu Department of Nephrology, Mills Peninsula Medical Center, Burlingame, CA, USA; uv Department of Health Policy and Management, Shahid Beheshti University of Medical Sciences, Tehran, Iran; uw Safety Promotion and Injury Prevention Research Center, Shahid Beheshti University of Medical Sciences, Tehran, Iran; ux Department of Leukemia, The University of MD Anderson Cancer Center, Houston, TX, USA; uy Statistics Unit, Riga Stradins University, Riga, Latvia; uz Department of Health and Safety, Dubai Municipality, Dubai, United Arab Emirates; va Department of Population Health, New York University, New York, NY, USA; vb The World Academy of Sciences UNESCO, Trieste, Italy; vc Shaanxi University of Technology, Hanzhong, China; vd Department of Environmental Engineering, Islamic Azad University, Ahvaz, Iran; ve Johns Hopkins University, Baltimore, MD, USA; vf School of Pharmaceutical Management, IIHMR University, Jaipur, India; vg Department of Pharmacology, Imam Mohammad Ibn Saud Islamic University, Riyadh, Saudi Arabia; vh Centre of Studies and Research, Ministry of Health, Muscat, Oman; vi Department of Biochemistry, Government Medical College, Mysuru, India; vj Department of Oral Medicine and Periodontology, University of Peradeniya, Peradeniya, Sri Lanka; vk Department of Oral Medicine and Periodontology, Saveetha University, Chennai, India; vl Department of Epidemiology, Florida International University, Miami, FL, USA; vm Department of Epidemiology and Health Promotion, Yonsei University, Seoul, South Korea; vn Department of Internal Medicine, GCS Medical College, Hospital & Research Centre, Ahmedabad, India; vo Department of Public Health, La Trobe University, Melbourne, VIC, Australia; vp Melbourne School of Population and Global Health, University of Melbourne, Melbourne, VIC, Australia; vq Faculty of Veterinary Medicine, University of Calgary, Calgary, AB, Canada; vr Young Researchers and Elite Club, Islamic Azad University, Karaj, Iran; vs Rothschild Foundation Hospital, Institute of Molecular and Clinical Ophthalmology Basel, Paris, France; vt Singapore Eye Research Institute, Singapore, Singapore; vu Health Services Management Training Centre, Semmelweis University, Budapest, Hungary; vv Hungarian Health Management Association, Budapest, Hungary; vw Department of Community Medicine, Manipal Academy of Higher Education, Mangalore, India; vx Department of Economics, National Open University, Benin City, Nigeria; vy Department of Family Medicine and Public Health, University of Opole, Opole, Poland; vz Institute of Family Medicine and Public Health, University of Tartu, Tartu, Estonia; wa Department of Physiotherapy, Manipal Academy of Higher Education, Manipal, India; wb Minimally Invasive Surgery Research Center, Iran University of Medical Sciences, Tehran, Iran; wc School of Public Health, University College Cork, Cork, Ireland; wd Department of Oral and Maxillofacial Pathology, Krishna Vishwa Vidyapeeth (Deemed to be University), Karad, India; we Academic Department of Surgery, University of Birmingham, Birmingham, UK; wf Department of Health Sciences, University of York, York, UK; wg Faculty of Dentistry, University of Puthisastra, Phnom Penh, Cambodia; wh Office of the Executive Director, Cephas Health Research Initiative Inc, Ibadan, Nigeria; wi Department of Respiratory Medicine, King George's Medical University, Lucknow, India; wj The Hansjörg Wyss Department of Plastic and Reconstructive Surgery, NYU Langone Health, New York, NY, USA; wk Cleft Lip and Palate Surgery Division, Global Smile Foundation, Norwood, MA, USA; wl 2nd Cardiology Department, Aristotle University of Thessaloniki, Thessaloniki, Greece; wm School of Health Professions and Human Services, Hofstra University, Hempstead, NY, USA; wn Department of Anesthesiology, Montefiore Medical Center, Bronx, NY, USA; wo Social Determinants of Health Research Center, Tabriz University of Medical Sciences, Tabriz, Iran; wp Cardiovascular Diseases Research Institute, Tehran University of Medical Sciences, Tehran, Iran; wq Endocrine Research Center, Iran University of Medical Sciences, Tehran, Iran; wr Department of Echocardiography, Iran University of Medical Sciences, Tehran, Iran; ws Department of Medical-Surgical Nursing, Guilan University of Medical Sciences, Rasht, Iran; wt Central Department of Public Health, Tribhuvan University, Kathmandu, Nepal; wu Department of Physical Therapy and Health Rehabilitation, Majmaah University, Majmaah, Saudi Arabia; wv MRC/CSO Social and Public Health Sciences Unit, University of Glasgow, Glasgow, UK; ww Public Health Foundation of India, New Delhi, India; wx Department of ENT, Dr. B. R. Ambedkar State Institute of Medical Sciences (AIMS), Mohali, India; wy Cardiac Primary Prevention Research Center, Tehran University of Medical Sciences, Tehran, Iran; wz Department of Cardiac Electrophysiology, Tehran University of Medical Sciences, Tehran, Iran; xa Community Medicine Department, Central Park Medical College, Lahore, Pakistan; xb Community Medicine & Public Health, University of Health Sciences, Lahore, Pakistan; xc Open, Distance and eLearning Campus, University of Nairobi, Nairobi, Kenya; xd Department of Human Nutrition, National Research Institute for Agriculture, Food and Environment, Jouy-en-Josas, France; xe Sorbonne Paris Nord University, Bobigny, France; xf Department of Public Health, Jordan University of Science and Technology, Irbid, Jordan; xg Amity Institute of Forensic Sciences, Amity University, Noida, India; xh School of Medicine, Tehran University of Medical Sciences, Tehran, Iran; xi Endocrinology and Metabolism Research Institute, Non-Communicable Diseases Research Center (NCDRC), Tehran, Iran; xj Department of Biostatistics, Mazandaran University of Medical Sciences, Sari, Iran; xk Natural and Medical Sciences Research Center, University of Nizwa, Nizwa, Oman; xl Department of Epidemiology, Jazan University, Jazan, Saudi Arabia; xm Department of Rehabilitation Sciences, Hong Kong Polytechnic University, Hong Kong, China; xn Family Medicine Department, United Arab Emirates University, Al Ain, United Arab Emirates; xo Department of Primary Care, NHS North West London, London, UK; xp Department of Epidemiology, Non-Communicable Diseases Research Center (NCDRC), Tehran, Iran; xq School of Medicine, Tehran University of Medical Sciences, Tehran, Iran; xr College of Health, Wellbeing and Life Sciences, Sheffield Hallam University, Sheffield, UK; xs College of Arts and Sciences, Ohio University, Zanesville, OH, USA; xt Faculty of Nursing, Yarmouk University, Irbid, Jordan; xu Department of Basic Medical Sciences, Yarmouk University, Irbid, Jordan; xv National Hepatology and Tropical Medicine Research Institute, Cairo University, Cairo, Egypt; xw Department of Public Health, Jordan University of Science and Technology, Irbid, Jordan; xx Department of Biochemistry, All India Institute of Medical Sciences, Jodhpur, India; xy Sina Trauma and Surgery Research Center, Tehran University of Medical Sciences, Tehran, Iran; xz Department of Internal Medicine, Corewell Health East William Beaumont University Hospital, Royal Oak, MI, USA; ya Department of Medical Oncology, Miami Cancer Institute, Miami, FL, USA; yb Department of Epidemiology and Biostatistics, Non-Communicable Diseases Research Center (NCDRC), Tehran, Iran; yc Department of Clinical Research, Icahn School of Medicine at Mount Sinai, New York City, NY, USA; yd Graduate School of Public Health, Yonsei University, Busan, South Korea; ye Broad Institute of MIT and Harvard, Cambridge, MA, USA; yf Massachusetts General Hospital, Boston, MA, USA; yg School of Traditional Chinese Medicine, Xiamen University Malaysia, Sepang, Malaysia; yh School of Health Sciences, Kristiania University College, Oslo, Norway; yi Department of International Health and Sustainable Development, Tulane University, New Orleans, LA, USA; yj Social Determinants of Health Research Center, Shahid Beheshti University of Medical Sciences, Tehran, Iran; yk Department of Physiology, Hamedan University of Medical Sciences, Hamedan, Iran; yl Department of Public Health Dentistry, Krishna Vishwa Vidyapeeth (Deemed to be University), Karad, India; ym Department of Neurosurgery, Helsinki University Hospital, Helsinki, Finland; yn Department of General Practice and Family Medicine, Kharkiv National Medical University, Kharkiv, Ukraine; yo Independent Consultant, Jakarta, Indonesia; yp Department of Epidemiology, IQVIA, Frankfurt am Main, Germany; yq University Hospital Marburg, Marburg, Germany; yr Department of Anthropology, Panjab University, Chandigarh, India; ys Department of Demography, University of Montreal, Montreal, QC, Canada; yt Department of Social and Preventive Medicine, University of Montreal, Montreal, QC, Canada; yu Department of Biochemistry, University of Hail, Hail, Saudi Arabia; yv Dental School, The University of Western Australia, Perth, WA, Australia; yw Atchabarov Scientific-Research Institute of Fundamental and Applied Medicine, Kazakh National Medical University, Almaty, Kazakhstan; yx Center of Medicine and Public Health, Asfendiyarov Kazakh National Medical University, Almaty, Kazakhstan; yy Department of Cardiovascular Medicine, Cabrini Institute, Rochester, MN, USA; yz Public Health Foundation of India, Gurugram, India; za Department of Community Medicine, Manipal Academy of Higher Education, Mangalore, India; zb College of Public Health & Health Informatics, University of Hail, Hail, Saudi Arabia; zc Geospatial Information Science and Engineering Hub, Indian Institute of Technology, Mumbai, India; zd Centre for Studies in Economics and Planning, Central University of Gujarat, Gandhinagar, India; ze Division of Cardiovascular Medicine, University of Kentucky, Lexington, KY, USA; zf School of Medicine and Dentistry, Griffith University, Gold Coast, QLD, Australia; zg Department of Nutrition and Food Science, Patuakhali Science and Technology University, Patuakhali, Bangladesh; zh Section of Cardiology, University of Manitoba, Winnipeg, MB, Canada; zi Translational Health Sciences, Bristol Medical School, University of Bristol, Bristol, UK; zj Faculty of Health and Life Sciences, Coventry University, Coventry, UK; zk Department of Medicine, McMaster University, Hamilton, ON, Canada; zl Department of Health Services Research and Management, City University of London, London, UK; zm Faculty of Public Health, University of Indonesia, Depok, Indonesia; zn Department of Environment and Public Health, University of Environment and Sustainable Development, Somanya, Ghana; zo Clinical Research Center, Turku University Hospital, Turku, Finland; zp Heart Center, University of Turku, Turku, Finland; zq Department of Clinical Sciences and Community Health, University of Milan, Milan, Italy; zr Integrated Department of Epidemiology, Health Policy, Preventive Medicine and Pediatrics, Foundation for People-centric Health Systems, New Delhi, India; zs Centre for Health: The Specialty Practice, New Delhi, India; zt School of Digital Science, Universiti Brunei Darussalam (University of Brunei Darussalam), Bandar Seri Begawan, Brunei; zu Institute of Applied Data Analytics, Universiti Brunei Darussalam (University of Brunei Darussalam), Bandar Seri Begawan, Brunei; zv Department of Occupational and Environmental Health, Yangzhou University, Yangzhou, China; zw Department of Respiratory and Critical Care Medicine, Northern Jiangsu People's Hospital, Yangzhou, China; zx School of Dentistry, The University of Queensland, Brisbane, QLD, Australia; zy Department of Public Health, University of Helsinki, Helsinki, Finland; zz Endocrinology and Metabolism Research Institute, Tehran University of Medical Sciences, Tehran, Iran; aaa Department of Otorhinolaryngology, Father Muller Medical College, Mangalore, India; aab Department of Surgery, National University of Singapore, Singapore, Singapore; aac International Society Doctors for the Environment, Arezzo, Italy; aad University of Medicine and Pharmacy at Ho Chi Minh City, Ho Chi Minh City, Vietnam; aae College of Optometry, Nova Southeastern University, Fort Lauderdale, FL, USA; aaf Department of Medical Science, Ajou University School of Medicine, Suwon, South Korea; aag Department of Precision Medicine, Sungkyunkwan University, Suwon-si, South Korea; aah Department of Family Medicine, University of Texas Medical Branch, Galveston, TX, USA; aai Department of Preventive Medicine, Korea University, Seoul, South Korea; aaj Faculty of Science, Universiti Brunei Darussalam (University of Brunei Darussalam), Bandar Seri Begawan, Brunei; aak Department of Public Health, Dilla University, Dilla, Ethiopia; aal Center for Dentistry and Oral Hygiene, University of Groningen, Groningen, Netherlands; aam Stomatological Hospital, Southern Medical University, Guangzhou, China; aan Department of Psychiatry, Yale University, New Haven, CT, USA; aao Department of Pharmaceutical Regulatory Affairs and Management, Manipal Academy of Higher Education, Manipal, India; aap Institute for Health Metrics and Evaluation, University of Washington, Seattle, WA, USA; aaq Department of Health Metrics Sciences, School of Medicine, University of Washington, Seattle, WA, USA; aar International Centre for Future Health Systems, University of New South Wales, Sydney, NSW, Australia; aas Institute for Health Metrics and Evaluation, University of Washington, Seattle, WA, USA; aat School of Life Sciences, University of Technology Sydney, Sydney, NSW, Australia; aau Department of Molecular Epidemiology, German Institute of Human Nutrition Potsdam-Rehbrücke, Potsdam, Germany; aav German Center for Diabetes Research (DZD), München-Neuherberg, Germany; aaw One Health Research Group, Universidad de Las Américas, Quito, Ecuador; aax Department of Medicine, University of São Paulo, São Paulo, Brazil; aay School of Medicine, Federal University of Juiz de Fora, Juiz de Fora, Brazil; aaz Department of Environmental Health Sciences, Mario Negri Institute for Pharmacological Research, Milan, Italy; bba Department of Population Health Sciences, Duke University, Durham, NC, USA; bbb Department of Chemistry, Salahaddin University-Erbil, Erbil, Iraq; bbc Department of Medical Biochemical Analysis, Cihan University-Erbil, Erbil, Iraq; bbd Centre for Public Health and Wellbeing, University of the West of England, Bristol, UK; bbe Department of Periodontology, Pomeranian Medical University, Szczecin, Poland; bbf Department of Biostatistics and Epidemiology, Yazd University of Medical Sciences, Yazd, Iran; bbg Department of Cardiology, Tehran University of Medical Sciences, Tehran, Iran; bbh School of Public Health, Imperial College London, London, UK; bbi Department of Orthopaedics, October 6 University, 6th of October City, Egypt; bbj Department of Medicine, Medical College of Georgia at Augusta University, Augusta, GA, USA; bbk Department of Cardiology, October 6 University, 6th of October City, Egypt; bbl Rama Medical College Hospital and Research Centre, Uttar Pradesh, India; bbm Institute of Applied Health Research, University of Birmingham, Birmingham, UK; bbn Rabigh Faculty of Medicine, King Abdulaziz University, Jeddah, Saudi Arabia; bbo Department of Maternal-Child Nursing and Public Health, Federal University of Minas Gerais, Belo Horizonte, Brazil; bbp Poche Centre for Indigenous Health, The University of Queensland, Brisbane, QLD, Australia; bbq Department of Cardiology, Tehran University of Medical Sciences, Tehran, Iran; bbr Department of Epidemiology and Biostatistics, Tehran University of Medical Sciences, Tehran, Iran; bbs Department of Population and Behavioural Sciences, University of Health and Allied Sciences, Ho, Ghana; bbt Biomedical Engineering Research Center (CREB), Universitat Politècnica de Catalunya (Barcelona Tech - UPC), Barcelona, Spain; bbu Department of Biomedical Engineering, University of Isfahan, Isfahan, Iran; bbv Psychiatry Department, Hospital Universitario Doctor Peset, Valencia, Spain; bbw Department of Medicine, University of Valencia, Valencia, Spain; bbx Department of Nutrition and Dietetics, University of Concepción, Concepción, Chile; bby Centre for Healthy Living, University of Concepción, Concepción, Chile; bbz Faculty of Humanities and Health Sciences, Curtin University, Sarawak, Malaysia; cca Jeffrey Cheah School of Medicine and Health Sciences, Monash University, Subang Jaya, Malaysia; ccb Department of Anatomy and Developmental Biology, Monash University, Clayton, VIC, Australia; ccc Department of Anatomy, Genetics and Biomedical Informatics, University of Colombo, Colombo, Sri Lanka; ccd Department of Public Health and Community Medicine, Central University of Kerala, Kasaragod, India; cce Community Medicine, Geetanjali Medical College and Hospital, Udaipur, India; ccf Department of General Medicine, Geetanjali Medical College and Hospital, Udaipur, India; ccg Department of Social Medicine, Federal University of Rio Grande do Sul, Porto Alegre, Brazil; cch Department of Medical and Surgical Sciences and Advanced Technologies “GF Ingrassia”, University of Catania, Catania, Italy; cci Department of Health Services Research and Policy, London School of Hygiene & Tropical Medicine, London, UK; ccj Department of Healthcare, University of Vlora, Vlora City, Albania; cck Clinic of Social and Family Medicine, University of Crete, Heraklion, Greece; ccl Centre for Health Innovation and Policy, Noida, India; ccm Department of Public Health, Arba Minch University, Arba Minch, Ethiopia; ccn School of Nursing and Midwifery, Deakin University, Melbourne, VIC, Australia; cco Department of Medical Laboratory Sciences, Adigrat University, Adigrat, Ethiopia; ccp Universidad Nacional Mayor de San Marcos, Lima, Peru; ccq Division of Forensic Medicine, Imam Abdulrahman Bin Faisal University, Dammam, Saudi Arabia; ccr Department of Physiology, King Saud University, Riyadh, Saudi Arabia; ccs General Administration Department, Helsinki University Hospital, Helsinki, Finland; cct School of Health Sciences, University of Melbourne, Melbourne, VIC, Australia; ccu Comprehensive Cancer Center, Helsinki University Hospital, Helsinki, Finland; ccv University of Helsinki, Helsinki, Finland; ccw University Centre Varazdin, University North, Varazdin, Croatia; ccx Institute for Health Metrics and Evaluation, University of Washington, Seattle, WA, USA; ccy Icelandic Centre for Social Research and Analysis, Reykjavik, Iceland; ccz Icelandic Centre for Social Research and Analysis (ICSRA), Reykjavik University, Reykjavik, Iceland; dda National Cancer Registry, Maria Sklodowska-Curie National Research Institute of Oncology, Warsaw, Poland; ddb Department of Pathology, Maria Sklodowska-Curie National Research Institute of Oncology, Warsaw, Poland; ddc Pacific Institute for Research & Evaluation, Calverton, MD, USA; ddd School of Public Health, Curtin University, Perth, WA, Australia; dde Multidisciplinary Department of Medical-Surgical and Dental Specialties, University of Campania “Luigi Vanvitelli”, Naples, Italy; ddf Saveetha Dental College and Hospitals, Saveetha University, Chennai, India; ddg Faculty of Nursing and Midwifery, Tabriz University of Medical Sciences, Tabriz, Iran; ddh Internal Medicine Programme, Kyrgyz State Medical Academy, Bishkek, Kyrgyzstan; ddi Department of Atherosclerosis and Coronary Heart Disease, National Center of Cardiology and Internal Disease, Bishkek, Kyrgyzstan; ddj College of Healthcare Management and Economics, Gulf Medical University, Ajman, United Arab Emirates; ddk Research and Development Department, Panacea Institute of Interdisciplinary Research and Education, Varanasi, India; ddl Department of Surgical Oncology, All India Institute of Medical Sciences, Jodhpur, India; ddm Department of Community Medicine, Manipal Academy of Higher Education, Mangalore, India; ddn College of Health Science, University of Hargeisa, Hargeisa, Somalia; ddo Institute of Health Science, Jimma University, Jimma, Ethiopia; ddp College of Applied and Natural Science, University of Hargeisa, Hargeisa, Somalia; ddq Department of Internal Medicine, Brown University, Providence, RI, USA; ddr Molecular Biology Unit, Sirius Training and Research Centre, Khartoum, Sudan; dds Bio-Statistical and Molecular Biology Department, Sirius Training and Research Centre, Khartoum, Sudan; ddt College of Medicine, University of Duhok, Duhok, Iraq; ddu Social Determinants of Health Research Center, Tabriz University of Medical Sciences, Tabriz, Iran; ddv Department of Midwifery, Tabriz University of Medical Sciences, Tabriz, Iran; ddw Skull Base Research Center, Shahid Beheshti University of Medical Sciences, Tehran, Iran; ddx Department of Public Health, Dire Dawa University, Dire Dawa, Ethiopia; ddy Health Systems and Policy Research Unit, Ahmadu Bello University, Zaria, Nigeria; ddz Heidelberg Institute of Global Health (HIGH), Heidelberg University, Heidelberg, Germany; eea Substance Abuse and Toxicology Research Center, Jazan University, Jazan, Saudi Arabia; eeb School of Health Sciences, University of Petroleum and Energy Studies, Dehradun, India; eec Institute for Health Metrics and Evaluation, University of Washington, Seattle, WA, USA; eed Department of Health Metrics Sciences, School of Medicine, University of Washington, Seattle, WA, USA; eee Department of Biostatistics, Shiraz University of Medical Sciences, Shiraz, Iran; eef Institute of Clinical Physiology, National Research Council, Pisa, Italy; eeg Department of Mathematics, The University of Jordan, Amman, Jordan; eeh Nonlinear Dynamics Research Center (NDRC), Ajman University, Ajman, United Arab Emirates; eei Department of Physiology, All India Institute of Medical Sciences, Deoghar, India; eej Faculty of Medicine and University Hospital Cologne, German Cancer Research Center, Heidelberg, Germany; eek Faculty of Medicine and University Hospital Cologne, University of Cologne, Cologne, Germany; eel Faculty of Medicine, Birjand University of Medical Sciences, Birjand, Iran; eem Iran University of Medical Sciences, Tehran, Iran; een Department of Public Health, Oswaldo Cruz Foundation, Recife, Brazil; eeo Department of Public Health, Federal University of Pernambuco, Recife, Brazil; eep Clinical Research Development Unit, Mashhad University of Medical Sciences, Mashhad, Iran; eeq Division of Plastic and Reconstructive Surgery, University of Washington Medical Center, Seattle, WA, USA; eer Institute for Health Metrics and Evaluation, University of Washington, Seattle, WA, USA; ees Department of Surgery, Ahmadu Bello University Teaching Hospital, Zaria, Nigeria; eet Department of Surgery, General University Hospital of Patras, Patras, Greece; eeu Faculty of Medicine, University of Thessaly, Larissa, Greece; eev Institute for Health Metrics and Evaluation, University of Washington, Seattle, WA, USA; eew Department of Health Economics, National Institute for Research in Tuberculosis, Chennai, India; eex Department of Community and Global Health, The University of Tokyo, Tokyo, Japan; eey Clinical Epidemiology Research Unit, Mexican Institute of Social Security, Villa de Alvarez, Mexico; eez Postgraduate in Medical Sciences, Universidad de Colima, Colima, Mexico; ffa Institute for Health Metrics and Evaluation, University of Washington, Seattle, WA, USA; ffb Department of Health Metrics Sciences, School of Medicine, University of Washington, Seattle, WA, USA; ffc Department of Psychiatry, Seoul National University, Seoul, South Korea; ffd Department of Neuropsychiatry, Seoul National University Bundang Hospital, Seongnam, South Korea; ffe Department of Computer Science, University of Illinois Urbana-Champaign, Urbana, IL, USA; fff College of Medicine and Public Health, Flinders University, Adelaide, SA, Australia; ffg Department of Engineering, Western Sydney University, Sydney, NSW, Australia; ffh Heart Failure Research Center, Isfahan University of Medical Sciences, Isfahan, Iran; ffi Neuroscience Research Center, Isfahan University of Medical Sciences, Isfahan, Iran; ffj Department of Medical Laboratory Analysis, Cihan University Sulaymaniya, Sulaymaniyah, Iraq; ffk Department of Dermatology, San Bortolo Hospital, Vicenza, Italy; ffl GISED Study Center, Bergamo, Italy; ffm Department of Health and Rehabilitation Sciences, Prince Sattam bin Abdulaziz University, Al Kharj, Saudi Arabia; ffn Suraj Eye Institute, Nagpur, India; ffo Department for the Control of Disease, Epidemics, and Pandemics, Ministry of Public Health, Yaoundé, Cameroon; ffp Department of Public Heath, University of Yaoundé I, Yaoundé, Cameroon; ffq University Institute of Public Health, The University of Lahore, Lahore, Pakistan; ffr National Dental Research Institute Singapore, Duke-NUS Medical School, Singapore, Singapore; ffs Nursing & Midwifery Research Department (NMRD), Hamad Medical Corporation, Doha, Qatar; fft Department of Dental Public Health, King Abdulaziz University, Jeddah, Saudi Arabia; ffu Department of Health Policy and Oral Epidemiology, Harvard University, Boston, MA, USA; ffv College of Medicine and Health Sciences, United Arab Emirates University, Al Ain, United Arab Emirates; ffw Department of Circulation and Medical Imaging, Norwegian University of Science and Technology, Trondheim, Norway; ffx Department of Biotechnology, University of Central Punjab, Lahore, Pakistan; ffy School of Medicine, Xiamen University, Xiamen, China; ffz Amity Institute of Forensic Sciences, Amity University, Noida, India; gga Department of Forensic Medicine, Manipal Academy of Higher Education, Manipal, India; ggb Department of Health Promotion, Zahedan University of Medical Sciences, Zahedan, Iran; ggc School of Pharmacy, West Virginia University, Morgantown, WV, USA; ggd Department of Anatomy and Embryology, Carol Davila University of Medicine and Pharmacy, Bucharest, Romania; gge Department of Cardiology, Cardio-Aid, Bucharest, Romania; ggf Research Center, Mashhad University of Medical Sciences, Mashhad, Iran; ggg HIV/STI Surveillance Research Center, Kerman University of Medical Sciences, Kerman, Iran; ggh Department of Epidemiology, Non-Communicable Diseases Research Center (NCDRC), Tehran, Iran; ggi Faculty of Medicine, Euromed University of Fes, Fez, Morocco; ggj Faculty of Medicine, University Sidi Mohammed Ben Abdellah, Fez, Morocco; ggk Department of Health Sciences, University of Tampere, Tampere, Finland; ggl Yong Loo Lin School of Medicine, National University of Singapore, Singapore, Singapore; ggm Institute for Health Metrics and Evaluation, University of Washington, Seattle, WA, USA; ggn Department of Biological Sciences, University of Embu, Embu, Kenya; ggo Cardiovascular laboratory, Methodist Hospital, Merrillville, Merrillville, IN, USA; ggp Department of Allergy, Immunology and Dermatology, Hanoi Medical University, Hanoi, Vietnam; ggq Faculty of Medicine, Duy Tan University, Da Nang, Vietnam; ggr Institute for Research and Training in Medicine, Biology and Pharmacy, Duy Tan University, Da Nang, Vietnam; ggs Cardiovascular Research Department, Methodist Hospital, Merrillville, IL, USA; ggt Department of Surgery, Danang Family Hospital, Danang, Vietnam; ggu Hitotsubashi Institute for Advanced Study (HIAS), Hitotsubashi University, Tokyo, Japan; ggv Institute for Cancer Control, National Cancer Center, Chuo-ku, Japan; ggw Department of General Medicine, University of Medicine and Pharmacy at Ho Chi Minh City, Ho Chi Minh City, Vietnam; ggx Institute for Mental Health Policy Research, Centre for Addiction and Mental Health, Toronto, ON, Canada; ggy Department of General Surgery, University Hospital of Heraklion, Heraklion, Crete, Greece; ggz Laboratory of Toxicology, University of Crete, Heraklion, Greece; hha Social Determinants of Health Research Center, Shahid Beheshti University of Medical Sciences, Tehran, Iran; hhb Department of Nephrology and Hypertension, Mayo Clinic, Rochester, MN, USA; hhc Department of Public Health, HSE Ireland, Dublin, Ireland; hhd Department of Public Health, UNICAF, Larnaca, Cyprus; hhe Technical Department, University of Cape Town, Cape Town, South Africa; hhf School of Public Health and Family Medicine, University of Cape Town, Cape Town, South Africa; hhg Center for Public Health, Teesside University, Middlesbrough, UK; hhh School of Chemical & Biomolecular Engineering, University of Sydney, Sydney, NSW, Australia; hhi Faculty of Applied Sciences, Department of Microbiology, Taiz University, Taiz, Yemen; hhj Global Research Institute, Keio University, Tokyo, Japan; hhk Department of Global Health Policy, University of Tokyo, Tokyo, Japan; hhl Department of Maternal and Child Health, International Centre for Diarrhoeal Disease Research, Bangladesh, Dhaka, Bangladesh; hhm Department of Statistics, Shahjalal University of Science and Technology, Sylhet, Bangladesh; hhn Department of Microbiology and Molecular Genetics, The Women University Multan, Multan, Pakistan; hho School of Health, Bam University of Medical Sciences, Bam, Iran; hhp Department of Radiology, Mayo Clinic, Rochester, MN, USA; hhq School of Information, University of California Berkeley, Berkeley, CA, USA; hhr Center of Excellence in Reproductive Health Innovation (CERHI), University of Benin, Benin City, Nigeria; hhs Department of Physiology, University of Benin, Edo, Nigeria; hht Department of Physiology, Benson Idahosa University, Benin City, Nigeria; hhu Department of Applied Economics and Quantitative Analysis, University of Bucharest, Bucharest, Romania; hhv James Cook University, Townsville, QLD, Australia; hhw Department of Veterinary Public Health and Preventive Medicine, University of Ilorin, Ilorin, Nigeria; hhx Department of Public Health, Arsi University, Asella, Ethiopia; hhy Department of Community Health and Primary Care, University of Lagos, Idi Araba, Nigeria; hhz Department of Family and Preventive Medicine, University of Utah, Salt Lake City, UT, USA; iia PSSM Data Sciences, Pfizer Inc., Groton, CT, USA; iib Sheffield Centre for Health and Related Research, University of Sheffield, Sheffield, UK; iic Department of Preventive Medicine, Kyung Hee University, Seoul, South Korea; iid Health Promotion Research Center, Zahedan University of Medical Sciences, Zahedan, Iran; iie Centre for Social Research in Health, University of New South Wales, Sydney, NSW, Australia; iif University of Sydney, Sydney, NSW, Australia; iig Department of Food and Nutrition, Seoul National University, Seoul, South Korea; iih College of Medicine, University of Ibadan, Ibadan, Nigeria; iii School of Pharmacy, University of the Western Cape, Cape Town, South Africa; iij Department of Psychiatry and Behavioural Neurosciences, McMaster University, Hamilton, ON, Canada; iik Department of Psychiatry, University of Lagos, Lagos, Nigeria; iil Department of Health Research Methods, Evidence, and Impact, McMaster University, Hamilton, ON, Canada; iim Department of Nursing Science, Bowen University, Iwo, Nigeria; iin Cardiology Department, Federal University of Rio de Janeiro, Rio de Janeiro, Brazil; iio Department of Community Medicine, Ahmadu Bello University, Zaria, Nigeria; iip Surgery Department, Sulaimani University, Sulaimani, Iraq; iiq ENT Department, Tor Vergata University of Rome, Rome, Italy; iir Department of Public Health, Ministry of Health, Bandar Seri Begawan, Brunei; iis Institute of Health Sciences, Universiti Brunei Darussalam (University of Brunei Darussalam), Bandar Seri Begawan, Brunei; iit Department of Geography, East Carolina University, Greenville, NC, USA; iiu Department of Pharmacotherapy and Pharmaceutical Care, Medical University of Warsaw, Warsaw, Poland; iiv Department of Microbiology and Immunology, University of Health and Allied Sciences, Ho, Ghana; iiw Sickle Cell Unit, Ho Teaching Hospital, Ho, Ghana; iix One Health Global Research Group, Universidad de las Americas (University of the Americas), Quito, Ecuador; iiy School of Medicine, Western Sydney University, Bathurst, NSW, Australia; iiz Department of Optometry and Vision Science, University of KwaZulu-Natal, KwaZulu-Natal, South Africa; jja Laboratory of Public Health Indicators Analysis and Health Digitalization, Moscow Institute of Physics and Technology, Dolgoprudny, Russia; jjb Department of Project Management, National Research University Higher School of Economics, Moscow, Russia; jjc Faculty of Medicine, University Ferhat Abbas of Setif, Setif, Algeria; jjd Division of Infectious Diseases, University Hospital of Setif, Setif, Algeria; jje Department of Respiratory Medicine, Jagadguru Sri Shivarathreeswara University, Mysore, India; jjf National School of Public Health, Institute of Health Carlos III, Madrid, Spain; jjg Department of Forensic Medicine and Toxicology, Manipal Academy of Higher Education, Mangalore, India; jjh Centre for the Business and Economics of Health, The University of Queensland, Brisbane, QLD, Australia; jji Australian Institute of Tropical Health and Medicine, James Cook University, Townsville, QLD, Australia; jjj Department of Mental Health, Hospital Universitari Vall d'Hebron (Vall d'Hebron University Hospital), Barcelona, Spain; jjk Department of Psychiatry, Mental Health and Addictions, Vall d'Hebron Institut de Recerca (Vall d'Hebron Research Institute), Barcelona, Spain; jjl Department of Epidemiology and Biostatistics, Anhui Medical University, Hefei, China; jjm Department of Nutrition and Dietetics, Harokopio University, Athens, Greece; jjn Board of Directors, National Public Health Organization, Athens, Greece; jjo Department of Ophthalmology, Heidelberg University, Heidelberg, Germany; jjp Public Health Foundation of India, Gurugram, India; jjq Department of Neurology, University of Bern, Bern, Switzerland; jjr Department of Neurology, University of Cyprus, Nicosia, Cyprus; jjs Department of Emergency Medicine, University of Thessaly, Larissa, Greece; jjt Department of Emergency Medicine, University of Bern, Bern, Switzerland; jju Department of Diabetes, Nutrition and Metabolic Diseases, Carol Davila University of Medicine and Pharmacy, Bucharest, Romania; jjv Department of Science and Mathematics, Deree-The American College of Greece, Athens, Greece; jjw Department of Biophysics, University of Athens, Athens, Greece; jjx Vision and Eye Research Institute, Anglia Ruskin University, Cambridge, UK; jjy Department of Community Medicine, All India Institute of Medical Sciences, Jammu, India; jjz Department of Epidemiology and Community Health, University of Minnesota, Minneapolis, MN, USA; kka Department of Medical Humanities and Social Medicine, Kosin University, Busan, South Korea; kkb Department of Biomedical Data Science, Stanford University, Stanford, CA, USA; kkc School of Psychological Sciences, Monash University, Melbourne, VIC, Australia; kkd Department of Medical Sciences, University of Torino, Torino, Italy; kke Department of Imaging, AOU Città della Salute e della Scienza di Torino, Torino, Italy; kkf Global Health Governance Programme, University of Edinburgh, Edinburgh, UK; kkg School of Dentistry, University of Leeds, Leeds, UK; kkh Department of Research and Training, Population Council Institute, New Delhi, India; kki College of Dental Medicine, Roseman University of Health Sciences, South Jordan, UT, USA; kkj Centre of Molecular Medicine and Diagnostics (COMManD), Saveetha University, Chennai, India; kkk Maternal and Child Health Division, International Centre for Diarrhoeal Disease Research, Bangladesh, Dhaka, Bangladesh; kkl Department of Genetics, Yale University, New Haven, CT, USA; kkm Australian Institute of Health Innovation, Macquarie University, Sydney, NSW, Australia; kkn School of Population Health, Curtin University, Bentley, WA, Australia; kko Centre for Fertility and Health, Norwegian Institute of Public Health, Oslo, Norway; kkp Social and Economic Survey Research Institute, Qatar University, Doha, Qatar; kkq Pennsylvania Cancer and Regenerative Medicine Center, Baruch S Blumberg Institute, Doylestown, PA, USA; kkr Department of Medicine, Xavier University School of Medicine, Woodbury, NY, USA; kks Facultad de Medicina, Universidad Diego Portales (Diego Portales University), Santiago, Chile; kkt School of Cardiovascular and Metabolic Health, University of Glasgow, Glasgow, UK; kku Institute for Health Metrics and Evaluation, University of Washington, Seattle, WA, USA; kkv School of Pharmacy, University of Nizwa, Nizwa, Oman; kkw Shanghai Mental Health Center, Shanghai Jiao Tong University, Shanghai, China; kkx Departments of Psychiatry and Epidemiology, Columbia University, New York, NY, USA; kky Department of Medicine, Nazarbayev University, Astana, Kazakhstan; kkz Clinical Academic Department of Pediatrics, University Medical Center (UMC), Astana, Kazakhstan; lla Department of Epidemiology and Evidence-Based Medicine, I.M. Sechenov First Moscow State Medical University, Moscow, Russia; llb Department of Data Management and Analysis, The International Clinical Epidemiology Network (INCLEN) Trust International, New Delhi, India; llc Department of Public Health, Erasmus University Medical Center, Rotterdam, Netherlands; lld Digestive Diseases Research Institute, Tehran University of Medical Sciences, Tehran, Iran; lle Humanities and Social Sciences, National Institute of Technology Rourkela, Rourkela, India; llf Department of Community Medicine, Tribhuvan University, Kathmandu, Nepal; llg T.H. Chan School of Public Health, Harvard University, Boston, MA, USA; llh Department of Clinical Research and Epidemiology, Institute of Liver and Biliary Sciences, New Delhi, New Delhi, India; lli Department of Biochemistry, Jagadguru Sri Shivarathreeswara University, Mysuru, India; llj Department of Maternal-Child Nursing and Public Health, Federal University of Minas Gerais, Belo Horizonte, Brazil; llk Health Sciences Department, Muhammadiyah University of Surakarta, Sukoharjo, Indonesia; lll Centre for Dental Education and Research, All India Institute of Medical Sciences, New Delhi, India; llm Department of Biostatistics, Epidemiology, and Informatics, University of Pennsylvania, Philadelphia, PA, USA; lln Department of Neonatology, Case Western Reserve University, Cleveland, OH, USA; llo Department of Medical Oncology, Cancer Institute (W.I.A), Chennai, India; llp Institute for Health Metrics and Evaluation, University of Washington, Seattle, WA, USA; llq Department of Community Medicine and Family Medicine, All India Institute of Medical Sciences, Jodhpur, India; llr Department of Medical Laboratory Technologies, Alnoor University, Mousl, Iraq; lls Al-Noor Center of Research and Innovation, Alnoor University, Mousl, Iraq; llt Iranian National Center for Addiction Studies, Tehran University of Medical Sciences, Tehran, Iran; llu Department of Population Science and Human Resource Development, University of Rajshahi, Rajshahi, Bangladesh; llv Department of Population Science and Human Resource Development, University of Rajshahi, Rajshahi, Bangladesh; llw Institute of Health and Wellbeing, Federation University Australia, Berwick, VIC, Australia; llx School of Nursing and Midwifery, La Trobe University, Melbourne, VIC, Australia; lly School of Medicine, Shahid Beheshti University of Medical Sciences, Tehran, Iran; llz Non-communicable Diseases Research Center, Tehran University of Medical Sciences, Tehran, Iran; mma Student Research Committee, Shahid Beheshti University of Medical Sciences, Tehran, Iran; mmb Guilan Road Trauma Research Center, Guilan University of Medical Sciences, Rasht, Iran; mmc Department of Community Medicine and Family Medicine, All India Institute of Medical Sciences, Gorakhpur, India; mmd Department of Public Health, Jazan University, Jazan, Saudi Arabia; mme Centre for Chronic Disease Control, New Delhi, India; mmf Department of Cardiology, Emory University, Atlanta, GA, USA; mmg Department of Clinical Science, University of Sharjah, Sharjah, United Arab Emirates; mmh Department of Cardiology, Mansoura University, Mansoura, Egypt; mmi Department of Population Health, King Saud bin Abdulaziz University for Health Sciences, Jeddah, Saudi Arabia; mmj Translational Health Research Institute, Western Sydney University, Sydney, NSW, Australia; mmk Department of Community Medicine, Shaheed Nirmal Mahto Medical College and Hospital, Dhanbad, India; mml Department of Research, Eastern Scientific LLC, Richmond, KY, USA; mmm Department of Health Promotion and Administration, Eastern Kentucky University, Richmond, KY, USA; mmn Department of Oral Pathology, Microbiology and Forensic Odontology, Sharavathi Dental College and Hospital, Shimogga, India; mmo Thrombosis Research Group, Brigham and Women's Hospital, Harvard Medical School, Boston, MA, USA; mmp Department of Epidemiology, Non-Communicable Diseases Research Center (NCDRC), Tehran, Iran; mmq Department of Medicine, Jinnah Sindh Medical University, Karachi, Pakistan; mmr Baylor University, Dallas, TX, USA; mms Non-communicable Diseases Research Center, Tehran University of Medical Sciences, Tehran, Iran; mmt Social Determinants of Health Research Center, Shahid Beheshti University of Medical Sciences, Tehran, Iran; mmu Department of Immunology, Shahid Beheshti University of Medical Sciences, Tehran, Iran; mmv Department of Family Medicine, Rajarata University of Sri Lanka, Anuradhapura, Sri Lanka; mmw Department of Global Health Policy, University of Tokyo, Tokyo, Japan; mmx Department of Neurosurgery, Helsinki University Hospital, Helsinki, Finland; mmy The National Institute for Stroke and Applied Neurosciences, Auckland University of Technology, Auckland, New Zealand; mmz Section of Pulmonary and Critical Care Medicine, University of Chicago, Chicago, IL, USA; nna Department of Primary Care and Public Health, Imperial College London, London, UK; nnb Academic Public Health England, Public Health England, London, UK; nnc Department of Internal Medicine, Manipal Academy of Higher Education, Mangalore, India; nnd Department of Biological Sciences, King Abdulaziz University, Jeddah, Egypt; nne Department of Protein Research, Research and Academic Institution, Alexandria, Egypt; nnf Non-communicable Diseases Research Center, Tehran University of Medical Sciences, Tehran, Iran; nng Endocrinology and Metabolism Research Institute, Tehran University of Medical Sciences, Tehran, Iran; nnh Department of Epidemiology and Biostatistics, Rafsanjan University of Medical Sciences, Rafsanjan, Iran; nni Department of Public Health, Masaryk University, Brno, Czech Republic; nnj Czech National Centre for Evidence-based Healthcare and Knowledge Translation, Masaryk University, Brno, Czech Republic; nnk Department of Geography and Demography, University of Coimbra, Coimbra, Portugal; nnl Department of Nursing in Women's Health, Federal University of São Paulo, São Paulo, Brazil; nnm Vaccination Research Observatory, Federal University of Minas Gerais, Belo Horizonte, Brazil; nnn Department of Pharmacology and Toxicology, University of Antioquia, Medellin, Colombia; nno Warwick Medical School, University of Warwick, Coventry, UK; nnp Department of Clinical Research, University of Sao Paulo, Ribeirão Preto, Brazil; nnq Gilbert and Rose-Marie Chagoury School of Medicine, Lebanese American University, Beirut, Lebanon; nnr College of Medicine, University of Florida, Gainesville, FL, USA; nns Golestan Research Center of Gastroenterology and Hepatology, Golestan University of Medical Sciences, Gorgan, Iran; nnt Rural Health Research Institute, Charles Sturt University, Orange, NSW, Australia; nnu Department of Analytical and Applied Economics, Utkal University, Bhubaneswar, India; nnv RUSA Centre of Excellence in Public Policy and Governance, Utkal University, Bhubaneswar, India; nnw Faculty of Medicine, Quest International University Perak, Ipoh, Malaysia; nnx Department of Biochemistry and Food Analysis, Patuakhali Science and Technology University, Patuakhali, Bangladesh; nny Department of Epidemiology, Florida International University, Miami, FL, USA; nnz Advanced Campus Governador Valadares, Juiz de For a Federal University, Governador Valadares, Brazil; ooa Department of Nursing, Universidade Presidente Antônio Carlos (President Antônio Carlos University), Governador Valadares, Brazil; oob Department of Oral and Maxillofacial Surgery, Jagadguru Sri Shivarathreeswara University, Mysore, India; ooc Department of Medicine, Georgetown University, Washington, DC, USA; ood Department of Epidemiology, Shahid Beheshti University of Medical Sciences, Tehran, Iran; ooe Faculty of Health and Dentistry, Diego Portales University, Santiago de Chile, Chile; oof Subdirección de Desarrollo Académico e investigación, Instituto Teletón, Santiago de Chile, Chile; oog College of Medicine, University of Sharjah, Sharjah, United Arab Emirates; ooh School of Population Health, University of New South Wales, Sydney, NSW, Australia; ooi Cardiac Rehabilitation Research Center, Isfahan University of Medical Sciences, Isfahan, Iran; ooj Department of Pharmaceutical Chemistry, International Medical University, Gdańsk, Poland; ook Clinical and Biomedical Research Center, Foundation University Islamabad, Islamabad, Pakistan; ool International Center of Medical Sciences Research (ICMSR), Islamabad, Pakistan; oom Endodontics Department, Mashhad University of Medical Sciences, Mashhad, Iran; oon Faculty of Medicine, Bioscience and Nursing, MAHSA University, Selangor, Malaysia; ooo Interdisciplinary Research Centre in Biomedical Materials (IRCBM), COMSATS Institute of Information Technology, Lahore, Pakistan; oop Department of Psychosocial Science, University of Bergen, Bergen, Norway; ooq Sharjah Institute of Medical Sciences, University of Sharjah, Sharjah, United Arab Emirates; oor Center for Global Health Research, Saveetha University, Chennai, India; oos Biotechnology Research Center, Mashhad University of Medical Sciences, Mashhad, Iran; oot Department of Community Medicine and Family Medicine, All India Institute of Medical Sciences, Bathinda, India; oou Department of Health and Kinesiology, University of Illinois, Urbana-Champaign, IL, USA; oov Department of Statistics, University of Gujrat, Gujrat, Pakistan; oow Department of Integrated Health Education, Federal University of Espirito Santo, Vitória, Brazil; oox College of Medicine, University of Sharjah, Sharjah, United Arab Emirates; ooy Faculty of Pharmacy, Mansoura University, Mansoura, Egypt; ooz Technology Management Department, University College of Applied Sciences, Gaza, Palestine; ppa School of Economics and Management, University of Kassel, Kassel, Germany; ppb College of Nursing, Jouf University, Jouf, Saudi Arabia; ppc Institute of Epidemiology and Preventive Medicine, National Taiwan University, Taipei, Taiwan; ppd Benang Merah Research Center (BMRC), Minahasa Utara, Indonesia; ppe Department of Entomology, Ain Shams University, Cairo, Egypt; ppf Medical Ain Shams Research Institute (MASRI), Ain Shams University, Cairo, Egypt; ppg Department of Surgery, Marshall University, Huntington, WV, USA; pph Department of Nutrition and Preventive Medicine, Case Western Reserve University, Cleveland, OH, USA; ppi Faculty of Medicine, University of Belgrade, Belgrade, Serbia; ppj School of Public Health and Health Management, University of Belgrade, Belgrade, Serbia; ppk Department of Infectious Diseases and Tropical Medicine, Federal University of Minas Gerais, Belo Horizonte, Brazil; ppl Research Development Coordination Section, Pakistan Health Research Council, Islamabad, Pakistan; ppm School of Sciences, University of Management and Technology, Lahore, Pakistan; ppn Pharmacy Study Program, Udayana University, Badung, Indonesia; ppo Department of Clinical Pharmacy, Taipei Medical University, Taipei, Taiwan; ppp Department of Pharmacology and Research, All India Institute of Medical Sciences, Jodhpur, India; ppq Indira Gandhi Medical College and Research Institute, Puducherry, India; ppr Department of Orthopaedics and Trauma Surgery, University of Freiburg, Freiburg, Germany; pps Department of Orthopaedics, Loretto Hospital Freiburg, Freiburg, Germany; ppt Department of Public Health, Jahrom University of Medical Sciences, Jahrom, Iran; ppu Health Policy Research Center, Shiraz University of Medical Sciences, Shiraz, Iran; ppv Department of Food Processing Technology, West Bengal State Council of Technical Education, Malda, India; ppw Department of Oral Pathology and Microbiology, Dr D Y Patil Vidyapeeth, Pune, Pune, India; ppx Department of Oral Pathology and Microbiology, Dr D Y Patil Vidyapeeth, Pune, Pune, India; ppy Faculty of Medicine, The University of Queensland, Brisbane, QLD, Australia; ppz Nuffield Department of Medicine, University of Oxford, Oxford, UK; zzr Department of Health Metrics Sciences, School of Medicine, University of Washington, Seattle, WA, USA; qqa Department of Geriatric and Long Term Care, Hamad Medical Corporation, Doha, Qatar; qqb Faculty of Health & Social Sciences, Bournemouth University, Bournemouth, UK; qqc Department of Medicine, Bangalore Medical College and Research Institute, Bangalore, India; qqd UGC Centre of Advanced Study in Psychology, Utkal University, Bhubaneswar, India; qqe Udyam-Global Association for Sustainable Development, Bhubaneswar, India; qqf Department of Public Health Sciences, University of North Carolina at Charlotte, Charlotte, NC, USA; qqg Psychiatry Clinic, Holy Savior Armenian Hospital, Istanbul, Turkiye; qqh Department of Diagnostic and Interventional Radiology and Neuroradiology, University Hospital Essen, Essen, Germany; qqi Swiss Research Institute for Public Health and Addiction, University of Zürich, Zurich, Switzerland; qqj Dobney Hypertension Centre, The University of Western Australia, Perth, WA, Australia; qqk Hypertension and Kidney Disease Laboratory, Baker Heart and Diabetes Institute, Melbourne, VIC, Australia; qql Postgraduate Program in Epidemiology, Federal University of Rio Grande do Sul, Porto Alegre, Brazil; qqm Cardiovascular Research Center, Massachusetts General Hospital, Boston, MA, USA; qqn Department of Cardiovascular Sciences, Katholieke Universiteit Leuven, Leuven, Belgium; qqo Institute for Health Metrics and Evaluation, University of Washington, Seattle, WA, USA; qqp Department of Community Oral Health and Clinical Prevention, University of Malaya, Kuala Lumpur, Malaysia; qqq College of Pharmacy, University of Sharjah, Sharjah, United Arab Emirates; qqr Research Institute of Medical & Health Sciences, University of Sharjah, Sharjah, United Arab Emirates; qqs Emergency Department, Manian Medical Centre, Erode, India; qqt Digestive Diseases Research Institute, Tehran University of Medical Sciences, Tehran, Iran; qqu Non-communicable Disease Research Center, Shiraz University of Medical Sciences, Shiraz, Iran; qqv Department of Medicine and Surgery, Government Doon Medical College, Dehradun, India; qqw Endocrinology and Metabolism Research Institute, Tehran University of Medical Sciences, Tehran, Iran; qqx National Heart, Lung, and Blood Institute, National Institute of Health, Rockville, MD, USA; qqy Department of Neurology, Tehran University of Medical Sciences, Tehran, Iran; qqz Non-communicable Diseases Research Center, Alborz University of Medical Sciences, Karaj, Iran; rra School of Medicine, Tehran University of Medical Sciences, Tehran, Iran; rrb Institute of Molecular Biology and Biotechnology, The University of Lahore, Lahore, Pakistan; rrc Research Centre for Health Sciences (RCHS), The University of Lahore, Lahore, Pakistan; rrd Department of Chemistry, Institute for Advanced Studies in Basic Sciences (IASBS), Zanjan, Iran; rre Center for Medical and Bio-Allied Health Sciences Research, Ajman University, Ajman, United Arab Emirates; rrf Icahn School of Medicine at Mount Sinai, New York, NY, USA; rrg Institute for Critical Care Medicine, Mount Sinai Health System, New York, NY, USA; rrh Independent Consultant, Karachi, Pakistan; rri Neurology Department, Ain Shams University, Cairo, Egypt; rrj Department of Pharmacology, All India Institute of Medical Sciences, Jodhpur, India; rrk Center for Medical and Bio-Allied Health Sciences Research, Ajman University, Ajman, United Arab Emirates; rrl Centre for Interdisciplinary Research in Basic Sciences (CIRBSc), Jamia Millia Islamia, New Delhi, India; rrm Science Department, Kazakh National Medical University, Almaty, Kazakhstan; rrn College of Nursing and Health Sciences, Jazan University, Jazan, Saudi Arabia; rro Department of Radiation Oncology, All India Institute of Medical Sciences, New Delhi, India; rrp Amity Institute of Public Health, Amity University, Noida, India; rrq Department of Medicine, Bangalore Medical College and Research Institute, Bangalore, India; rrr Department for Evidence-based Medicine and Evaluation, University for Continuing Education Krems, Krems, Austria; rrs Research Center for Rational Use of Drugs, Tehran University of Medical Sciences, Tehran, Iran; rrt Department of Social and Behavioral Health, University of Nevada Las Vegas, Las Vegas, NV, USA; rru Department of Human Genetics and Molecular Medicine, Central University of Punjab, Bathinda, India; rrv Institute of Forensic Science & Criminology, Panjab University, Chandigarh, India; rrw Centre for Medical Informatics, University of Edinburgh, Edinburgh, UK; rrx Division of General Internal Medicine, Harvard University, Boston, MA, USA; rry Department of Endocrinology and Metabolism Population Sciences, Tehran University of Medical Sciences, Tehran, Iran; rrz School of Medicine, Non-Communicable Diseases Research Center (NCDRC), Tehran, Iran; ssa K S Hegde Medical Academy, Nitte University, Mangalore, India; ssb Department of Forensic Medicine and Toxicology, Manipal Academy of Higher Education, Mangalore, India; ssc Manipal College of Dental Sciences Mangalore, Manipal Academy of Higher Education, Mangalore, India; ssd Department of Public Health, Dambi Dollo University, Dembi Dollo, Ethiopia; sse Department of Epidemiology, Jimma University, Jimma, Ethiopia; ssf Department of Pharmacology, Saint Paul's Hospital Millennium Medical College, Addis Ababa, Ethiopia; ssg Finnish Institute of Occupational Health, Helsinki, Finland; ssh Department of Veterinary Public Health and Preventive Medicine, Usmanu Danfodiyo University, Sokoto, Sokoto, Nigeria; ssi Oulu Business School, University of Oulu, Oulu, Finland; ssj Martti Ahtisaari Institute, University of Oulu, Oulu, Finland; ssk Department of Experimental Research, Medical University Pleven, Sofia, Bulgaria; ssl Department of Genetics, Sofia University “St. Kliment Ohridiski”, Sofia, Bulgaria; ssm Department of Medical-Surgical Nursing, Mazandaran University of Medical Sciences, Sari, Iran; ssn Department of Nursing and Health Sciences, Flinders University, Adelaide, SA, Australia; sso Department of Research and Academics, Kathmandu Cancer Center, Bhaktapur, Nepal; ssp Unit of Basic Medical Sciences, University of Khartoum, Khartoum, Sudan; ssq Department of Medical Microbiology and Infectious Diseases, Erasmus University, Rotterdam, Netherlands; ssr Research Unit on Applied Molecular Biosciences (UCIBIO), University of Porto, Porto, Portugal; sss Department of Dentistry, All India Institute of Medical Sciences, Bhopal, India; sst Department of Biochemistry, Central University of Punjab, Bathinda, India; ssu Department of Pharmacology, Government Medical College and Hospital, Chandigarh, India; ssv School of Medicine, Baylor College of Medicine, Houston, TX, USA; ssw Department of Medicine Service, US Department of Veterans Affairs (VA), Houston, TX, USA; ssx Department of Radiodiagnosis, All India Institute of Medical Sciences, Bathinda, India; ssy Department of Human Genetics, Punjabi University, Patiala, India; ssz Department of Pharmacology, All India Institute of Medical Sciences, Jodhpur, India; tta Department of Pulmonary Medicine, Mahaveer Jaipuria Rajasthan Hospital, Jaipur, India; ttb Centre for Primary Health Care and Equity (CPHCE), University of New South Wales, Sydney, NSW, Australia; ttc Menzies Centre for Health Policy, University of Sydney, Sydney, NSW, Australia; ttd Institute for Health Metrics and Evaluation, University of Washington, Seattle, WA, USA; tte Department of Psychiatry, Jimma University, Jimma, Ethiopia; ttf Department of Systemic Pathology, Touro College of Osteopathic Medicine, Middletown, NY, USA; ttg Department of Pathology, American University of the Caribbean School of Medicine, Cupecoy, Saint Martin; tth Department of Neuroscience, University of Ottawa, Ottawa, ON, Canada; tti Student Research Committee, Urmia University of Medical Sciences, Urmia, Iran; ttj School of Medicine, Babol University of Medical Sciences, Babol, Iran; ttk Hospital Universitario de La Princesa, Universidad Autónoma de Madrid (Autonomous University of Madrid), Madrid, Spain; ttl Centro de Investigación Biomédica en Red Enfermedades Respiratorias (CIBERES) (Center for Biomedical Research in Respiratory Diseases Network), Madrid, Spain; ttm Hull York Medical School, University of Hull, Hull City, UK; ttn 3rd Department of Cardiology, University of Athens, Athens, Greece; tto Division of Community Medicine and Public Health, International Medical University, Kuala Lumpur, Malaysia; ttp 2nd Department of Cardiology, Aristotle University of Thessaloniki, Thessaloniki, Greece; ttq SAMRC Unit on Risk and Resilience in Mental Disorders, University of Cape Town, Cape Town, South Africa; ttr Department of Medicine, Democritus University of Thrace, Alexandroupolis, Greece; tts Faculty of Medicine, University of Belgrade, Belgrade, Serbia; ttt Global Observatory on Pollution and Health, Boston College, Chestnut Hill, MA, USA; ttu ISGlobal Instituto de Salud Global de Barcelona, Barcelona, Spain; ttv Center for Biotechnology and Microbiology, University of Swat, Swat, Pakistan; ttw School of Life Sciences, Xiamen University, Xiamen, China; ttx Department of Disease Burden, Norwegian Institute of Public Health, Bergen, Norway; tty Department of Biomedical Sciences, Universiti Putra Malaysia, Selangor, Malaysia; ttz Department of Neurology, King George's Medical University, Lucknow, India; uua Department of Analytical and Applied Economics, Utkal University, Bhubaneswar, India; uub Henry JN Taub Department of Emergency Medicine, Baylor College of Medicine, Houston, TX, USA; uuc Department of Clinical Research and Development, LUXMED Group, Warsaw, Poland; uud Department of Pharmacology, All India Institute of Medical Sciences, Deoghar, India; uue Department of Neurology, Neurocenter of Southern Switzerland (NSI), Lugano, Switzerland; uuf Endocrinology and Metabolism Research Institute, Tehran University of Medical Sciences, Tehran, Iran; uug Department of Basic Medical Sciences, Islamic Azad University, Mashhad, Iran; uuh Department of Internal Medicine, Islamic Azad University, Mashhad, Iran; uui Department of Primary Care and Public Health, Imperial College London, London, UK; uuj Dentistry and Oral Health, Rural Clinical Sciences, La Trobe University, Bendigo, VIC, Australia; uuk School of Dentistry and Oral Health, Griffith University, Gold Coast, QLD, Australia; uul Department of Environmental, Agricultural and Occupational Health, University of Nebraska Medical Center, Omaha, NE, USA; uum Sri Ramachandra Medical College and Research Institute, Chennai, India; uun Clinical Sciences Department, University of Sharjah, Sharjah, United Arab Emirates; uuo Department of Pathology, Alexandria University, Alexandria, Egypt; uup National Centre for Epidemiology and Population Health, Australian National University, Acton, ACT, Australia; uuq Statistics Discipline, Khulna University, Khulna, Bangladesh; uur Department of Dermatology, Carol Davila University of Medicine and Pharmacy, Bucharest, Romania; uus Department of Dermato-Venereology, Dr. Victor Babes Clinical Hospital of Infectious Diseases and Tropical Diseases, Bucharest, Romania; uut Department of Epidemiology, Stellenbosch University, Cape Town, South Africa; uuu Department of Medicine, Northlands Medical Group, Omuthiya, Namibia; uuv Department of Surgery, National University of Singapore, Singapore, Singapore; uuw Department of Psychiatry, Bahir Dar University, Bahir Dar, Ethiopia; uux National Research and Innovation Agency, Jakarta, Indonesia; uuy Department of Urology, Sabzevar University of Medical Sciences, Sabzevar, Iran; uuz Pediatric Intensive Care Unit, King Saud University, Riyadh, Saudi Arabia; vva College of Pharmacy, Alfaisal University, Riyadh, Saudi Arabia; vvb Department of Epidemiology and Biostatistics, University of California San Francisco, San Francisco, CA, USA; vvc Outpatient Department, Wollega University, Bedele town, Ethiopia; vvd Department of Public Health, Wollega University, Nekemte, Ethiopia; vve Department of Pharmacology, All India Institute of Medical Sciences, Raipur, India; vvf Public Health Department, Amrita Institute of Medical Sciences, Kochi, India; vvg Department of Community Medicine, Manipal Academy of Higher Education, Mangalore, India; vvh Institute of Applied Health Research, University of Birmingham, Birmingham, UK; vvi Faculty of Medicine, University of Southampton, Southampton, UK; vvj Department of Gastroenterology, St. Luke's Hospital, Patanamthitta, India; vvk Faculty of Public Health, Universitas Sam Ratulangi (Sam Ratulangi University), Manado, Indonesia; vvl Department of Pharmacology, All India Institute of Medical Sciences, Jodhpur, India; vvm Institute of Public Health, Jagiellonian University Medical College, Kraków, Poland; vvn Agency for Health Technology Assessment and Tariff System, Warsaw, Poland; vvo Saveetha Dental College and Hospitals, Saveetha University, Chennai, India; vvp High Institute of Sport and Physical Education of Sfax, University of Sfax, Sfax, Tunisia; vvq Department of Medicine, Pham Ngoc Thach University of Medicine, Ho Chi Minh City, Vietnam; vvr School of Biomedical Engineering, University of Technology Sydney, Sydney, NSW, Australia; vvs Department of Internal Medicine, University of Medicine and Pharmacy at Ho Chi Minh City, Ho Chi Minh City, Vietnam; vvt Department of Business Analytics, University of Massachusetts Dartmouth, Dartmouth, MA, USA; vvu Molecular Neuroscience Research Center, Shiga University of Medical Science, Shiga, Japan; vvv Faculty of Public Health, University of Indonesia, Depok, Indonesia; vvw Department of Community and Family Medicine, All India Institute of Medical Sciences, Nagpur, India; vvx Faculty of Medicine, Nam Can Tho University, Can Tho, Vietnam; vvy Department of Psychiatry, Dalhousie University, Halifax, NS, Canada; vvz Department of Occupational Health and Safety, University of Development, Surabaya, Indonesia; wwa Department of Biosciences and Biotechnology, University of Medical Sciences, Ondo, Ondo, Nigeria; wwb Faculty of Health and Life Sciences, University of Exeter, Exeter, UK; wwc Natural and Medical Sciences Research Center, University of Nizwa, Nizwa, Oman; wwd International Center for Chemical and Biological Sciences, University of Karachi, Karachi, Pakistan; wwe Medical Genomics Research Department, King Abdullah International Medical Research Center, Riyadh, Saudi Arabia; wwf Department of Life Sciences, University of Management and Technology, Lahore, Pakistan; wwg Department of Cardiovascular, Endocrine-metabolic Diseases and Aging, National Institute of Health, Rome, Italy; wwh Kasturba Medical College, Mangalore, Manipal Academy of Higher Education, Manipal, India; wwi Department of Physiotherapy, Bayero University Kano, Kano, Nigeria; wwj Department of Rehabilitation Sciences, Hong Kong Polytechnic University, Hong Kong, China; wwk Department of Informatics and Radiology, Mayo Clinic, Rochester, MN, USA; wwl College of Health and Sport Sciences, University of Bahrain, Zallaq, Bahrain; wwm Department of Cardiovascular Sciences, Katholieke Universiteit Leuven, Leuven, Belgium; wwn Laboratory of Toxicology, University of Crete, Heraklion, Greece; wwo UKK Institute, Tampere, Finland; wwp Faculty of Medicine and Health Technology, Tampere University, Tampere, Finland; wwq Department of Infectious Disease, Kermanshah University of Medical Sciences, Kermanshah, Iran; wwr Human Genetics and OMICS, Department of Zoology, Central University of Punjab, Bathinda, India; wws Department of Human Genetics & Molecular Biology, Bharathiar University, Coimbatore, India; wwt Raffles Neuroscience Centre, Raffles Hospital, Singapore, Singapore; wwu Yong Loo Lin School of Medicine, National University of Singapore, Singapore, Singapore; wwv Department of Community Medicine and Family Medicine, All India Institute of Medical Sciences, Bathinda, India; www School of Mathematics and Statistics, Carleton University, Ottawa, ON, Canada; wwx Department of Cardiology, Icahn School of Medicine at Mount Sinai, New York, NY, USA; wwy Department of Medical and Surgical Sciences, University of Bologna, Bologna, Italy; wwz Occupational Medicine Unit, Sant'Orsola Malpighi Hospital, Bologna, Italy; xxa Department of Molecular Epidemiology, Research Institute for Systems Biology and Medicine, Moscow, Russia; xxb Department of Information Technologies and Management, Moscow Institute of Physics and Technology, Dolgoprudny, Russia; xxc Department of Medical Oncology, University of Medicine and Pharmacy “Grigore T Popa” Iasi, Iaşi, Romania; xxd Department of Medical Oncology, Regional Institute of Oncology, Iaşi, Romania; xxe Department of Biochemistry, Abdul Wali Khan University Mardan, Mardan, Pakistan; xxf School of Health Sciences, National University of Sciences and Technology (NUST), Islamabad, Pakistan; xxg Lebanese American University, Byblos, Lebanon; xxh Department of Psychiatry, Haramaya University, Harar, Ethiopia; xxi Department of Neurosurgery, Capital Medical University, Beijing, China; xxj Department of Neurosurgery, Beijing Tiantan Hospital, Beijing, China; xxk School of Life Course and Population Sciences, King's College London, London, UK; xxl Key Laboratory of Computer-Aided Drug Design, Guangdong Medical University, Dongguan, China; xxm Department of Biotechnology and Genetic Engineering, Hazara University Mansehra, Mansehra, Pakistan; xxn Department of Community Medicine, Rajarata University of Sri Lanka, Anuradhapura, Sri Lanka; xxo Institute of Clinical Epidemiology, Public Health, Health Economics, Medical Statistics and Informatics, Medical University Innsbruck, Innsbruck, Austria; xxp Department of Public Health and Primary Care, University of Cambridge, Cambridge, UK; xxq Department of Research, Cancer Registry of Norway, Oslo, Norway; xxr Department of Chemical Toxicology, Norwegian Institute of Public Health, Oslo, Norway; xxs National Data Management Center for Health (NDMC), Ethiopian Public Health Institute, Addis Ababa, Ethiopia; xxt Institute for Health Metrics and Evaluation, University of Washington, Seattle, WA, USA; xxu Department of Public Health, Debre Markos University, Debre Markos, Ethiopia; xxv School of Public Health, Zhejiang University, Zhejiang, China; xxw Department of Public Health Science, Fred Hutchinson Cancer Research Center, Seattle, WA, USA; xxx Department of Endocrinology, University of Science and Technology of China, Hefei, China; xxy School of Medicine, University of Rochester, Rochester, NY, USA; xxz Department of Microbiology, Central University of Punjab, Bathinda, India; yya Department of Public Health, Juntendo University, Tokyo, Japan; yyb Department of Public Health Medicine, University of Tsukuba, Tsukuba, Japan; yyc Department of Epidemiology, University of Florida, Gainesville, FL, USA; yyd Department of Cancer Epidemiology and Prevention Research, Alberta Health Services, Calgary, AB, Canada; yye Department of Oncology, University of Calgary, Calgary, AB, Canada; yyf Faculty of Medicine, Juntendo University, Tokyo, Japan; yyg Department of Medicine, Shiraz University of Medical Sciences, Shiraz, Iran; yyh Department of Medicine, Mashhad University of Medical Sciences, Mashhad, Iran; yyi Manipal College of Nursing, Manipal Academy of Higher Education, Udupi, India; yyj Biostatics, Epidemiology, and Science Computing Department, King Faisal Specialist Hospital & Research Center, Riyadh, Saudi Arabia; yyk Department of Respiratory Medicine, Military Medical University, Chongqing, China; yyl Department of Health Management, Süleyman Demirel Üniversitesi (Süleyman Demirel University), Isparta, Turkiye; yym Department of Epidemiology, Xuzhou Medical University, Xuzhou, China; yyn Department of Pediatrics, Kyung Hee University, Seoul, South Korea; yyo Department of Biostatistics, University of Toyama, Toyama, Japan; yyp Department of Public Health, Juntendo University, Tokyo, Japan; yyq Department of Preventive Medicine, Korea University, Seoul, South Korea; yyr Department of Epidemiology and Biostatistics, Wuhan University, Wuhan, China; yys Institute for Health Metrics and Evaluation, University of Washington, Seattle, WA, USA; yyt Faculty of Medicine and Health Sciences, Hodeidah University, Hodeidah, Yemen; yyu Department of Virology, University of Helsinki, Helsinki, Finland; yyv Department of Public Health, University of Hail, Hail, Saudi Arabia; yyw Department of Zoology and Entomology, Al-Azhar University, Cairo, Egypt; yyx School of Public Health, Peking University, Beijing, China; yyy Department of International Health, Johns Hopkins University, Baltimore, MD, USA; yyz Melbourne Medical School, University of Melbourne, Melbourne, VIC, Australia; zza Victorian Comprehensive Cancer Centre, Melbourne, VIC, Australia; zzb Medical Oncology Department of Gastrointestinal Cancer, Cancer Hospital of Dalian University of Technology, Shenyang, China; zzc School of Biomedical Engineering, Dalian University of Technology, Dalian, China; zzd Jockey Club School of Public Health and Primary Care, The Chinese University of Hong Kong, Hong Kong, China; zze School of Humanities and Management, Zhejiang Chinese Medical University, Hangzhou, China; zzf School of Public Health and Emergency Management, Southern University of Science and Technology, Shenzhen, China; zzg Department of Biochemistry and Pharmacogenomics, Medical University of Warsaw, Warsaw, Poland; zzh Endocrinology and Metabolism Research Center, Hormozgan University of Medical Sciences, Bandar Abbas, Iran; zzi Department of Clinical and Community Pharmacy, An-Najah National University, Nablus, Palestine; zzj An-Najah National University Hospital, Clinical Research Centre, An-Najah National University, Nablus, Palestine; zzk Institute for Health Metrics and Evaluation, University of Washington, Seattle, WA, USA; zzl Department of Health Metrics Sciences, School of Medicine, University of Washington, Seattle, WA, USA; zzm GBD Collaborating Unit, Norwegian Institute of Public Health, Bergen, Norway; zzn Institute for Health Metrics and Evaluation, University of Washington, Seattle, WA, USA; zzo Department of Health Metrics Sciences, School of Medicine, University of Washington, Seattle, WA, USA

## Abstract

**Background:**

Smoking is the leading behavioural risk factor for mortality globally, accounting for more than 175 million deaths and nearly 4·30 billion years of life lost (YLLs) from 1990 to 2021. The pace of decline in smoking prevalence has slowed in recent years for many countries, and although strategies have recently been proposed to achieve tobacco-free generations, none have been implemented to date. Assessing what could happen if current trends in smoking prevalence persist, and what could happen if additional smoking prevalence reductions occur, is important for communicating the effect of potential smoking policies.

**Methods:**

In this analysis, we use the Institute for Health Metrics and Evaluation's Future Health Scenarios platform to forecast the effects of three smoking prevalence scenarios on all-cause and cause-specific YLLs and life expectancy at birth until 2050. YLLs were computed for each scenario using the Global Burden of Disease Study 2021 reference life table and forecasts of cause-specific mortality under each scenario. The reference scenario forecasts what could occur if past smoking prevalence and other risk factor trends continue, the Tobacco Smoking Elimination as of 2023 (Elimination-2023) scenario quantifies the maximum potential future health benefits from assuming zero percent smoking prevalence from 2023 onwards, whereas the Tobacco Smoking Elimination by 2050 (Elimination-2050) scenario provides estimates for countries considering policies to steadily reduce smoking prevalence to 5%. Together, these scenarios underscore the magnitude of health benefits that could be reached by 2050 if countries take decisive action to eliminate smoking. The 95% uncertainty interval (UI) of estimates is based on the 2·5th and 97·5th percentile of draws that were carried through the multistage computational framework.

**Findings:**

Global age-standardised smoking prevalence was estimated to be 28·5% (95% UI 27·9–29·1) among males and 5·96% (5·76–6·21) among females in 2022. In the reference scenario, smoking prevalence declined by 25·9% (25·2–26·6) among males, and 30·0% (26·1–32·1) among females from 2022 to 2050. Under this scenario, we forecast a cumulative 29·3 billion (95% UI 26·8–32·4) overall YLLs among males and 22·2 billion (20·1–24·6) YLLs among females over this period. Life expectancy at birth under this scenario would increase from 73·6 years (95% UI 72·8–74·4) in 2022 to 78·3 years (75·9–80·3) in 2050. Under our Elimination-2023 scenario, we forecast 2·04 billion (95% UI 1·90–2·21) fewer cumulative YLLs by 2050 compared with the reference scenario, and life expectancy at birth would increase to 77·6 years (95% UI 75·1–79·6) among males and 81·0 years (78·5–83·1) among females. Under our Elimination-2050 scenario, we forecast 735 million (675–808) and 141 million (131–154) cumulative YLLs would be avoided among males and females, respectively. Life expectancy in 2050 would increase to 77·1 years (95% UI 74·6–79·0) among males and 80·8 years (78·3–82·9) among females.

**Interpretation:**

Existing tobacco policies must be maintained if smoking prevalence is to continue to decline as forecast by the reference scenario. In addition, substantial smoking-attributable burden can be avoided by accelerating the pace of smoking elimination. Implementation of new tobacco control policies are crucial in avoiding additional smoking-attributable burden in the coming decades and to ensure that the gains won over the past three decades are not lost.

**Funding:**

Bloomberg Philanthropies and the Bill & Melinda Gates Foundation.

## Introduction

Smoking has accounted for more than 175 million deaths globally over the past three decades.^1^ Despite substantial progress in reducing smoking prevalence in many countries, smoking remains a leading risk factor for preventable morbidity and mortality.^2^ More than one in ten global deaths and nearly 142 million years of life lost (YLLs) were attributable to smoking in 2021.^1^ Smoking also has important effects on health-care costs, productivity, and health disparities.^3^, ^4^, ^5^ As a result, tobacco control is an enduring policy and public health priority, with enormous potential to improve population health.

**Research in context**

**Evidence before this study**

Smoking is widely recognised as a major global health risk with extensive supporting literature. The Global Burden of Diseases, Injuries, and Risk Factors Study serves as the most comprehensive source for smoking prevalence and all-cause and cause-specific attributable burden across 204 countries and territories, by age and sex. However, fewer studies have focused on forecasting tobacco use and burden. We searched PubMed on July 9, 2024, using the following search terms: ((“smok*”[All Fields] OR “tobacco”[All Fields]) AND (“forecast*”[All Fields] OR “scenario*”[All Fields] OR “projection*”[All Fields]) AND (“prevalence”[All Fields] OR “burden”[All Fields] OR “disease”[All Fields])). We restricted the search to articles published in the past 10 years. The search yielded 1098 studies. Many studies have forecasted smoking prevalence in the status quo, as well as under various policy scenarios, for specific countries. A 2024 WHO report forecasts global smoking prevalence will be 30·6% among males and 5·7% among females in 2030. Previous studies have also estimated future disease burden from smoking, although these studies typically focus on all causes combined or a small subset of smoking-related causes. Our search did not identify any studies that have estimated the future burden of smoking for all countries and all smoking-related health conditions.

**Added value of this study**

Our study contributes a comprehensive set of estimates of future health burden under three smoking prevalence scenarios for 204 countries and 365 diseases and injuries, disaggregated by 5-year age group and sex. Methodologically, we have developed a forecasting framework that incorporates smoking prevalence, intensity, duration, and risk reduction from cessation. Furthermore, by leveraging the Institute for Health Metrics and Evaluation's Future Health Scenarios platform, our forecasts integrate dynamic changes in demographics and other determinants of health.

**Implications of all the available evidence**

The current evidence underscores the substantial potential for health gains through more aggressive tobacco control policies, worldwide. Although a continuation of the existing decline in smoking prevalence will undoubtedly yield health benefits, our analysis quantifies the substantial increase in life expectancy and decrease in years of life lost that could result from accelerated efforts in tobacco control.

After a period of accelerated progress following the adoption of WHO's Framework Convention on Tobacco Control, progress has slowed in recent years.^6^, ^7^ Although global prevalence of smoking continues to decline, the pace of decline fluctuates and has slowed in many countries. Renewed efforts are required to overcome the tobacco industry's attempts to maintain a market. The concept of a tobacco endgame, in which focus shifts from controlling the tobacco epidemic to eliminating the tobacco epidemic, has been discussed for more than 10 years in the academic literature.^8^, ^9^ Countries and organisations around the world have set goals to reduce smoking prevalence to less than 5% in the coming years.^10^, ^11^, ^12^, ^13^

Strategies to reach a tobacco-free future work together to eliminate the initiation of tobacco use among youth while steadily phasing out tobacco use in the adult population. Despite promising policy proposals in some countries, including the UK,^14^ the political commitment to implement and enforce the measures necessary to realise a tobacco-free future largely remains elusive. New Zealand's transformational legislation that would have prohibited the sale of tobacco to anyone born on or after Jan 1, 2009, was recently repealed to fund other tax cuts. Similarly, Malaysia's generational smoking ban was dropped from their recent tobacco control bill.^15^, ^16^ Forecasts of the health effects of potential smoking policy scenarios provide quantitative evidence on the costs of inaction that can aid decision makers.

Previous studies have simulated the effects of reducing smoking prevalence in selected countries.^17^, ^18^, ^19^, ^20^, ^21^, ^22^, ^23^, ^24^, ^25^, ^26^, ^27^, ^28^, ^29^ These studies find large population health benefits that accrue over time, the potential for health-care cost savings, and reductions in smoking-attributable health disparities.^30^, ^31^ Although many prospective policy simulation studies exist for some countries, such analyses do not exist for all countries. Furthermore, previous studies have not systematically disaggregated impacts by disease, which can aid health system planning and prioritisation of medical innovation. Finally, health forecasting is a dynamic process that reflects simultaneous changes across many determinants of health and feedback between changes in health determinants and changes in demography. Our study adds to existing evidence by providing comprehensive country-specific and disease-specific estimates under three smoking prevalence scenarios using a dynamic forecasting framework.

With the use of the Institute for Health Metrics and Evaluation's Future Health Scenarios platform, we aim to forecast all-cause and cause-specific YLLs for all countries from 2022 to 2050, as well as life expectancy gains, under three smoking prevalence scenarios. The reference scenario, which reflects a continuation of current trends in smoking prevalence, and the Tobacco Smoking Elimination as of 2023 (Elimination-2023) scenario, in which smoking prevalence is immediately reduced to zero, quantify the bounds of potential future health benefits from smoking elimination. The third scenario, Tobacco Smoking Elimination by 2050 (Elimination-2050), which includes a generational ban beginning in 2023 and a steady reduction of smoking prevalence to 5% among older cohorts by 2050, provides insight into what gains could be realised by countries considering policies to reach endgame targets. Together, these scenarios underscore the magnitude of health benefits, benefits that will continue to grow beyond 2050, which could be achieved if countries take action to end the global tobacco epidemic. This manuscript was produced as part of the GBD Collaborator Network and in accordance with the GBD Protocol.

## Methods

### Overview

We used the Future Health Scenarios platform to forecast YLLs under three smoking prevalence scenarios, for 204 countries and territories as well as 365 diseases and injuries by 5-year age group and sex from 2022 to 2050. In the following sections, we focus primarily on methods specific to this analysis of the effects of smoking prevalence reductions. The methods used to forecast population, fertility, and all-cause mortality have been reported elsewhere.^32^, ^33^, ^34^, ^35^ Methods and accompanying estimates for smoking-specific inputs, including smoking prevalence, continuous measures of intensity of exposure, and dose-response relative risks for 32 health outcomes have also been previously published.^1^ In this analysis, we only report the direct effects from smoking tobacco, which do not include the health effects from second-hand smoke, smokeless tobacco use, or electronic nicotine delivery systems. This study adheres to the Guidelines for Accurate and Transparent Health Estimates Reporting statement (appendix 1 pp 16–17).^36^

### Forecasting framework

We obtained estimates of independent drivers of health, including over 70 risk factor summary exposure values from the Global Burden of Diseases, Injuries, and Risk Factors Study (GBD), interventions such as vaccines and antiretroviral therapy coverage, and covariates such as Socio-demographic Index (a composite measure of income, education, and fertility under age 25 years) from GBD 2021, for every location, age, and sex, from 1990 to 2019. These independent variables were then forecasted to 2050, mostly using a generalised ensemble model that includes past annual rates of change and relationship with Socio-demographic Index to predict future trends (appendix 1 p 13).

Once we have a complete time series from 1990 to 2050 for each of the independent variables, we use them to forecast cause-specific mortality rates up to 2050. Future mortality is estimated using three components: underlying (risk-deleted) mortality, a risk factor scalar, and latent trends predicted with an autoregressive integrated moving average (0,1,0) model.^35^ These cause-specific mortality estimates are aggregated to obtain all-cause mortality.^33^ We used the methods published in Vollset and colleagues’ study^33^ to obtain life tables and population estimates to 2050. Briefly, age-specific and sex-specific future population was computed for each location based on future all-cause mortality, fertility, and net migration. Importantly, population and life expectancy were computed independently for each scenario, enabling us to capture the effects of differing mortality rates on population age structure and size. YLLs were then computed for each scenario using demographically aggregated mortality forecasts and the GBD reference life table.^33^ Details on validation of our forecasting model can be found in previous studies.^32^, ^33^, ^35^

### Smoking scenario definitions

We forecast cause-specific and all-cause mortality under three scenarios, which we refer to as the reference, Elimination-2023, and Elimination-2050 scenarios. The reference scenario assumes that past independent health driver trends and relationships between drivers and health outcomes persist into the future. To estimate an upper bound on the effect of reducing smoking prevalence, we constructed the Elimination-2023 scenario, under which past trends persist across all inputs except current smoking prevalence, which is reduced to zero from 2023 onwards. Finally, we constructed the Elimination-2050 scenario, under which past trends persist across all inputs except smoking prevalence, which is linearly reduced to 5% between 2023 and 2050. The Elimination-2050 scenario provides a benchmark that falls between the reference scenario and Elimination-2023 scenario.

For all scenarios, we use GBD 2021 estimates of distributions of cigarette-equivalents smoked per day and pack-years among current smokers, distributions of years since quitting among former smokers, and cause-specific relative risk estimates for both current and former smokers. We assumed the 2022 age-specific distributions of cigarette-equivalents smoked per day and pack-years among current smokers remained constant into the future. In the reference scenario, we similarly assumed the 2022 age-specific distributions of years since quitting among former smokers remained constant into the future. In the alternative scenarios, we shifted the 2022 years since quitting distributions for former smokers who quit in 2022 or earlier forward with every future year and created uniform distributions to model the years since quitting for former smokers who quit in 2023 or later. We then used these inputs, and scenario-specific prevalence forecasts for current smokers, former smokers, and never smokers, to compute population attributable fractions for smoking between 2020 and 2050, which in turn were used to compute smoking summary exposure values. The population attributable fraction calculation was adapted from the GBD study, with additional terms added to capture the burden among recent quitters (appendix 1 p 12).

### Reference scenario forecast

The reference scenario is a probabilistic forecast that allows historical trends of drivers of health to continue into the future and holds the past relationship between drivers and health outcomes constant. To forecast smoking prevalence in this scenario, we first obtained current smoking and former smoking prevalence estimates for every location, sex, 5-year age group, and year from 1990 to 2019 from GBD 2021.^1^ We then used an ensemble model to forecast current and former smoking prevalence from 2020 to 2050.^35^ Briefly, the ensemble models consisted of six submodels that used annualised rates of change to forecast prevalence to 2050 based on past smoking trends. Each submodel had a recency-weighting parameter ranging from 0 to 2·5, in which larger values correspond to more weight given to the trend in recent years. Model performance was assessed using a 10-year holdout. The final forecast was a weighted average of these submodels with each weighted by the inverse of their root mean squared error.

### Alternative smoking prevalence scenarios

The Elimination-2023 scenario was constructed with the reference scenario's current and former smoking prevalence values from 2020 to 2022, setting current smoking prevalence to 0% from 2023 onwards. We considered the population of current smokers in 2022 to be former smokers from 2023 onwards and tracked their years since quitting for every year from 2023 to 2050. For the population of existing former smokers that quit smoking between 1990 and 2022, we held the distribution of years since quitting constant and extended it into the future (ie, if for a given cohort the mean years since quitting was 10 in 2022, the mean years since quitting for that cohort would be 15 in 2027).

The Elimination-2050 scenario was constructed similarly. We created the scenario by setting smoking prevalence to zero for birth cohorts of ages 0–19 years in 2023. For older cohorts, we linearly reduced current smoking prevalence starting in 2023 to 5% by 2050. For older cohorts with current smoking prevalence rates below 5% in 2022, we held that prevalence rate constant to 2050. As in the Elimination-2023 scenario, we held the distribution of years since quitting constant and logically extended it into the future for the population of existing former smokers that quit smoking between 1990 and 2022. We used a uniform distribution of years since quitting for the population of former smokers that quit during or after 2023, since the proportion of people quitting between 2023 and 2050 was constant.

To account for the effects of different mortality rates between people who currently, formerly, and never smoked, we estimated the all-cause relative risks of mortality for each smoking status. First, we computed exposure-weighted relative risks by location, age, sex, and cause in 2022. We then aggregated these cause-specific relative risks across all causes to generate an all-cause relative risk of mortality. Finally, we computed the mortality rate among people who never smoked and used each of the mortality rates to adjust our prevalence estimates in every future year. As a result, the share of current and former smokers in the population decreased over time in each of the alternative scenarios.

### Uncertainty estimation

Uncertainty intervals (UIs) were computed from distributions for each estimate generated by propagating 500 draws through the multistage computational pipeline. The 95% uncertainty interval of estimates is based on the 2·5th and 97·5th percentile of draws. We report results with the mean and UI for all three scenarios in the main text and tables, and only for the reference scenario in figures. We did all analyses using R (version 4.2.2) and Python (version 3.10.13).

### Role of the funding source

The funders of the study had no role in study design, data collection, data analysis, data interpretation, or writing of the report.

## Results

### Smoking prevalence forecasts

**Figure 1:**
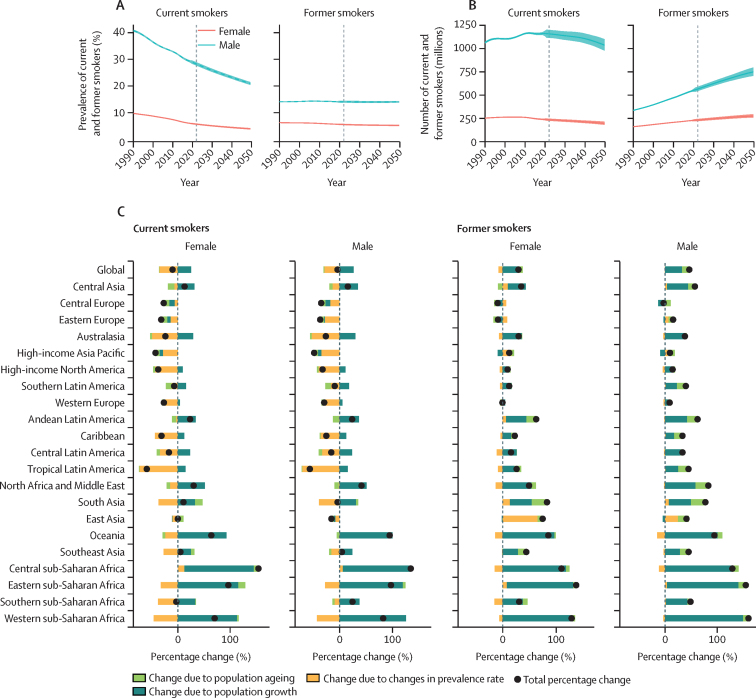
Annual change in global smoking (A) Changes in current and former smoking prevalence over time, age standardised. (B) Number of current and former smokers over time for all ages. Estimates for 1990–2019 were obtained from GBD 2021 for (A) and (B). The solid lines in (A) and (B) indicate the mean estimate, whereas the shaded areas reflect the 95% uncertainty interval. (C) Decomposition of forecasted change in number of smokers from 2023 to 2050 due to population ageing, population growth, and changes in the prevalence rate among current and former smokers. The grey dashed vertical line in (A) and (B) indicates 2022 (the first forecast year). The black dots in (C) indicate the overall percentage change in the number of smokers from 2023 to 2050.

The age-standardised prevalence of current smoking declined globally from 1990 to 2022, from 40·8% (95% UI 40·4–41·1) to 28·5% (27·9–29·1) among males aged 10 years and older, and from 9·94% (9·77–10·15) to 5·96% (5·76–6·21) among females aged 10 years and older (figure 1). We forecast that this decline will continue, albeit at a slower pace, with an age-standardised prevalence of 21·1% (20·6–21·6) among males and 4·18% (3·98–4·48) among females in 2050, a 25·9% (25·2–26·6) and 30·0% (26·1–32·1) decline relative to 2022, respectively. By 2050, the age-standardised prevalence among males is forecasted to range between 3·18% (2·95–3·47) in Brazil and 63·2% (60·6–65·6) in the Federated States of Micronesia, and among females between 0·50% (0·36–0·68) in Nigeria and 38·5% (35·2–41·8) in Serbia (appendix 2 pp 3–9). Despite a forecasted increase of the global population of 1·44 billion (95% UI 1·07–1·84) or 18·1% (95% UI 13·6–23·0) between 2022 and 2050, as well as population ageing, the number of current smokers is also forecast to decline. Globally, we forecast 1040 million (95% UI 980–1110) male smokers and 201 million (187–220) female smokers by 2050. The largest relative decline in the number of current smokers is forecasted to occur in Tropical Latin America and the largest increase in central sub-Saharan Africa for both sexes. Population growth is the largest factor responsible for increases in current smoking populations in most regions.

Global age-standardised prevalence of former smoking among those aged 20 years and older is forecast to stay relatively constant from 14·3% (95% UI 13·9–14·6) in 2022 to 14·2% (13·9–14·6) in 2050 among males and from 5·81% (5·60–6·05) to 5·50% (5·28–5·72) among females. The forecasted age-standardised prevalence of former smoking among males in 2050 ranges between 3·80% (3·44–4·17) in Benin and 56·9% (54·1–59·1) in Tanzania, and among females between 0·47% (0·39–0·56) in Libya and 46·9% (42·9–51·5) in Tanzania. Due to a combination of population growth, ageing, and increases in the prevalence of former smoking, the number of former smokers is forecasted to increase by 28·5% (23·0–34·6) by 2050. Compared with 800 million (95% UI 771–835) former smokers in 2022, we forecast 1030 million (973–1090) former smokers in 2050. The number of former smokers is forecast to increase in most GBD regions, apart from central Europe among males and eastern Europe, central Europe, and western Europe among females. In these regions, declines in the population between 2022 and 2050 offset any increases in former smoking prevalence. Population growth and ageing are the largest factors responsible for increases in the former smoking population in the regions with an increasing number of former smokers.

### YLLs under the reference scenario

**Figure 2:**
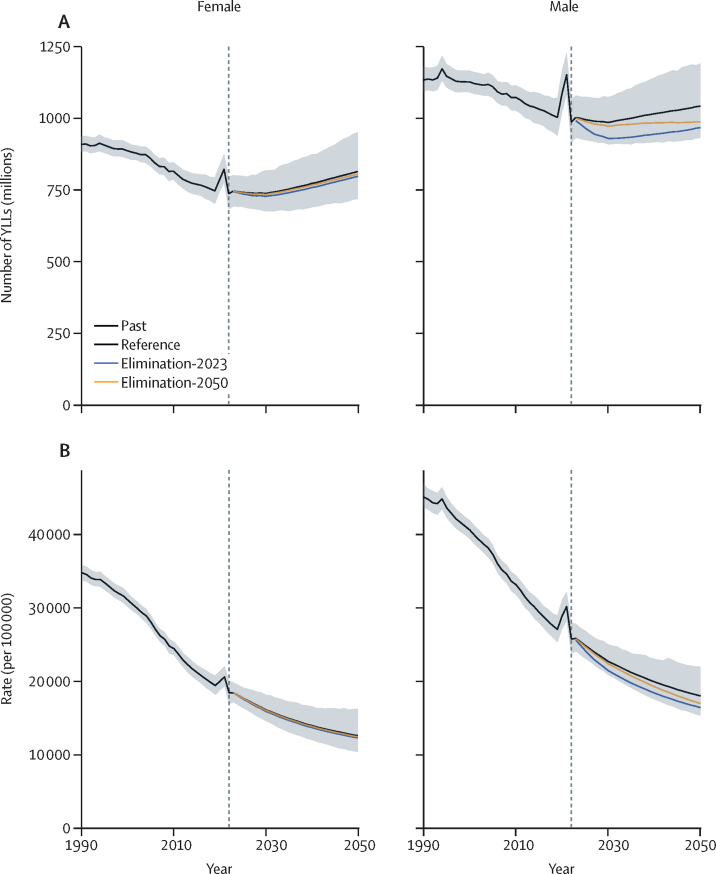
Global YLLs by scenario, all causes The solid lines indicate the mean estimates of the number of all-age YLLs (A) and the rate of age-standardised YLLs (B). The shaded area reflects the 95% uncertainty interval for the past and reference scenario. Estimates for 1990–2021 were obtained from GBD 2021. The grey dashed vertical line indicates 2022 (the first forecast year). YLLs=years of life lost.

**Figure 3:**
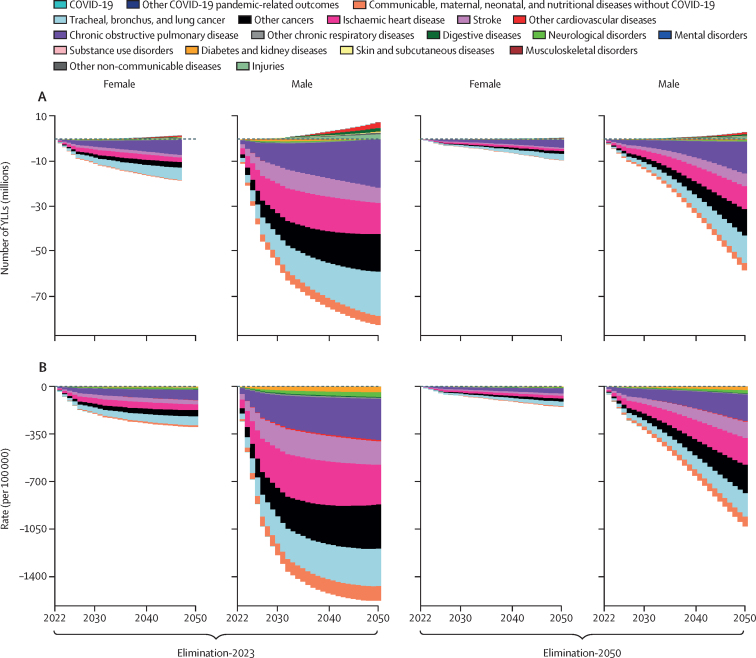
Global YLLs by GBD Level 2 causes of death under the reference scenario, Elimination-2050 scenario, and Elimination-2023 scenario (A) Difference in number of YLLs compared with the reference scenario. (B) Difference in rate of age-standardised YLLs compared with the reference scenario. Negative net differences in (A) and (B) indicate causes from which YLLs would be avoided under each custom scenario, whereas net positive differences indicate causes from which additional YLLs would occur. YLLs=years of life lost.

YLLs represent the number of life-years lost due to premature death. Globally, there were 1020 million (95% UI 942–1090) YLLs among males and 757 million (705–810) among females in 2022 (figure 2). Under our reference scenario, we project that there will be 1040 million (932–1190) YLLs among males and 816 million (720–953) YLLs among females in 2050, for a cumulative total of 51·5 billion (47·3–56·9) future YLLs between 2022 and 2050. Although large, these global increases are a product of population growth and ageing. The global age-standardised rate of YLLs is forecast to decrease from 26 530·9 (24 623·2–28 614·0) per 100 000 among males in 2022 to 17 113·2 (14 553·3–20 949·3) per 100 000 in 2050, and from 18 919·3 (17 608·9–20 322·8) per 100 000 to 12 448·6 (10 282·1–16 034·6) per 100 000 among females. In our reference scenario non-communicable diseases comprise the majority of future YLLs, accounting for 68·1% (95% UI 64·9–70·8) of cumulative YLLs among females and 64·3% (61·8–66·3) among males (figure 3). Non-communicable diseases are also responsible for the largest age-standardised YLL rates, accounting for 11 273·6 (10 328·7–12 498·2) YLLs per 100 000 between 2022 and 2050 among males and 7760·4 (7007·3–8602·8) per 100 000 among females. Cardiovascular diseases and cancers are the leading causes of non-communicable diseases YLLs in this scenario, accounting for 12·3 billion (95% UI 10·8–14·1) and 9·05 billion (8·13–9·92) cumulative future YLLs, respectively.

### YLLs under alternative scenarios

**Figure 4:**
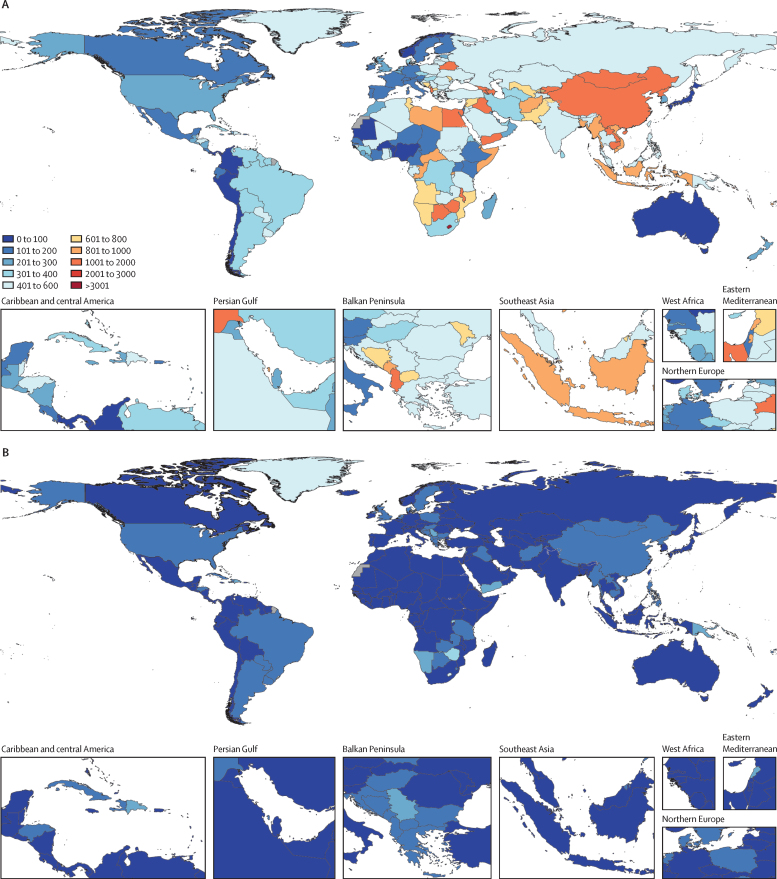
Difference in cumulative age-standardised rate of YLLs compared with the reference scenario 2022 to 2050 (per 100 000) (A) The difference in the cumulative age-standardised rate of YLLs between the reference scenario and the Elimination-2050 scenario among males. (B) The difference between the reference scenario and the Elimination-2050 scenario among females. YLLs=years of life lost.

The difference between the number of YLLs under the reference scenario and Elimination-2023 scenario approximates the maximum number of future smoking-attributable YLLs that can be avoided. Our forecasts suggest that a maximum of 2040 million (95% UI 1900–2210) cumulative smoking-attributable YLLs are theoretically avoidable between 2022 and 2050, with 1700 million (1570–1850) YLLs avoidable among males and 341 million (318–369) YLLs avoidable among females globally. The GBD regions with the greatest number of avoidable YLLs are East Asia and South Asia, with 708 million (620–809) and 385 million (320–455) cumulative YLLs avoidable, respectively. The greatest decreases in the cumulative age-standardised rate of YLLs between these scenarios can be seen in Oceania and East Asia, with decreases of 1342·0 (95% UI 1057·9–1746·4) per 100 000, and 1076·2 (911·8–1274·7) per 100 000, respectively (figure 4). The largest gains could be accomplished in China, India, and Indonesia, with a potential 1·08 billion (95% UI 0·98–1·21) YLLs avoidable with smoking prevalence elimination as of 2023.

Under the Elimination-2050 scenario, gains in health burden as measured with YLLs are delayed relative to the Elimination-2023 scenario but are similar by 2050. Under this scenario, we project 735 million (95% UI 675–808) fewer cumulative YLLs among males and 141 million (131–154) fewer cumulative YLLs among females relative to the reference scenario. Of the 1700 million (1570–1850) avoidable YLLs among males and 341 million (318–369) avoidable YLLs among females if smoking prevalence was reduced to 0% globally in 2023, 43·2% (42·7–43·9) and 41·5% (40·9–42·1) are avoidable if smoking prevalence elimination is delayed until 2050. The GBD regions with the largest reductions in cumulative YLLs under the Elimination-2050 scenario relative to the reference are East Asia, with 336 million (298–379) cumulative avoidable YLLs, and South Asia, with 172 million (142–206) cumulative avoidable YLLs. The Elimination-2050 scenario forecasts an age-standardised rate of 20 038·4 (18 092·9–22 666·7) cumulative YLLs per 100 000 among males and 14 466·7 (12 785·2–16 594·3) per 100 000 among females, a reduction of 560·0 (505·7–629·0) per 100 000 and 95·7 (85·2–108·4) per 100 000 compared with the reference scenario. The countries that would have the largest number of YLLs avoided between 2022 and 2050 under the Elimination-2050 scenario are China, India, and Indonesia, with reductions of 328 million (289–371), 124 million (100–151), and 38 million (31·9–45·7), respectively.

Cancers, ischaemic heart disease, and chronic obstructive pulmonary disease (COPD) account for 85·2% (95% UI 81·4–87·2) of avoidable YLLs. Cancers account for 692 million (595–782) avoidable YLLs among males and 140 million (121–159) among females, with lung cancer responsible for 51·8% (47·9–55·1) of avoidable cancer YLLs among males and 62·6% (59·3–65·7) of avoidable cancer YLLs among females. COPD and ischaemic heart disease account for 482 million (413–552) and 426 million (332–549) of all avoidable YLLs, respectively. Even if smoking is globally eliminated, premature death will still occur due to other drivers, such as air pollution or obesity. Under the Elimination-2050 scenario, 876 million (810–958) YLLs could be avoided relative to the reference scenario. COPD, ischaemic heart disease, and lung cancer are the leading causes of avoidable YLLs under this scenario, accounting for 220 million (190–253), 175 million (130–236), and 178 million (142–213) YLLs, respectively. However, unlike the Elimination-2023 scenario, under the Elimination-2050 scenario, the majority of avoidable YLLs occur after 2040 (59·4% [58·9–60·1], relative to 44·0% [43·4–44·8] under the Elimination-2023 scenario). Time series plots of the YLLs forecast under each of these scenarios for ischaemic heart disease, lung cancer, and COPD are available in appendix 2 (pp 27–30).

### Life expectancy at birth

**Figure 5:**
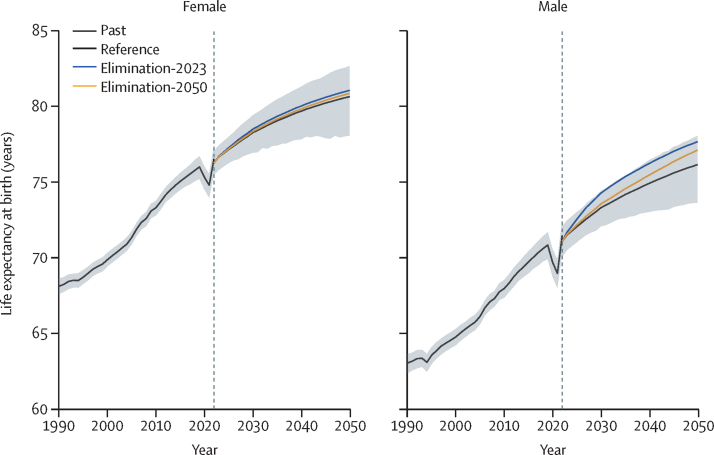
Global life expectancy at birth by sex and scenario The solid lines indicate the mean estimates of life expectancy at birth. The shaded area reflects the 95% uncertainty interval for the past and reference scenario. The grey dashed vertical line indicates 2022 (the first forecast year). Estimates for 1990 to 2021 were obtained from GBD 2021.

Global life expectancy at birth has increased between 1990 and 2022 by 7·95 years (95% UI 6·81–9·05) among males (63·1 years [62·4–63·7] to 71·1 years [70·1–72·0]) and 8·1 years (7·2–9·0) among females (68·1 years [67·6–68·6] to 76·2 years [75·5–77·0]; figure 5). Under the reference scenario, we forecast that the life expectancy at birth would continue to increase, climbing to 76·1 years (73·6–78·0) among males and 80·6 years (78·1–82·6) among females in 2050. If smoking were to be eliminated in 2023, the life expectancy would increase to 77·6 years (75·1–79·6) among males and 81·0 years (78·5– 83·1) among females in 2050, globally. This corresponds to a 31·0% (23·1–52·1) among males and 10·4% (6·8–22·2) among females larger gain in life expectancy under the Elimination-2023 scenario compared with the reference scenario. The GBD regions with the largest gains in life expectancy among males relative to the reference under the Elimination-2023 scenario would be East Asia, Eastern Europe, and Central Asia, with 2·6 (2·3–2·8), 2·0 (1·9–2·0), and 1·9 (1·9–2·0) additional years, respectively. Among females, the GBD regions with the largest gains in life expectancy by 2050 possible with elimination of smoking prevalence in 2023 are high-income North America, East Asia, and Oceania, with 0·9 (0·8–0·9), 0·7 (0·7–0·8), and 0·7 (0·6–0·9) additional years, respectively. Under the Elimination-2050 scenario, life expectancy at birth would increase by a further 1·0 years (0·9–1·0) among males and 0·2 years (0·2–0·2) among females globally, relative to the reference scenario. Similar to the Elimination-2023 scenario, the GBD regions with the largest gains in life expectancy among males under the Elimination-2050 scenario would be East Asia, Central Asia, and Southeast Asia, with 1·8 (1·6–2·0), 1·3 (1·2–1·4), and 1·2 (1·1–1·3) additional years of life gained. Among females, the GBD regions with the largest gains in life expectancy are East Asia, high-income North America, and Oceania, with 0·5 (0·4–0·6), 0·3 (0·3–0·4), and 0·3 (0·3–0·4) years gained.

## Discussion

In this Article, we present forecasts of smoking prevalence and resulting YLLs and life expectancy to 2050 for a reference scenario and two alternative scenarios. We forecast that 2040 million (95% UI 1900–2210) years of life will be lost to smoking between 2022 and 2050, of which 1·70 million (1·85–1·57), or 83·3% (95% UI 82·3–84·2) are among males. With our reference scenario, we forecast an additional gain in life expectancy of 4·8 years (95% UI 2·4–6·4) between 2022 and 2050. Life expectancy increased globally by an additional 1·5 years (1·5–1·6) among males and 0·4 years (0·4–0·4) among females between 2022 and 2050 in our Elimination-2023 scenario, and 1·0 years (0·9–1·0) and 0·2 years (0·2–0·2) in our Elimination-2050 scenario. The additional years of life expectancy forecast in the Elimination-2050 scenario represent an increase of 19·6% (95% UI 14·7–32·9) among males and 5·33% (3·62–11·09) among females, compared with our reference scenario. Our analysis shows that large population health gains can be achieved by accelerating progress towards smoking elimination.

This level of benefit is rare from a single, feasible intervention. In comparison, increased health-care spending per capita by 10% over a 20-year period has been estimated to have improved life expectancy in Organisation for Economic Co-operation and Development countries by 3·5 months.^37^ Increases of 10% in income per capita and primary educational attainment were estimated to improve life expectancy by 2·2 months and 3·2 months, respectively.^37^ Accelerated progress with regard to a single behavioural risk factor would result in life expectancy gains that are greater than these advancements. Importantly, these gains represent an average across the entire population, including people who have never smoked, and are therefore underestimates of the gains expected among smokers.

This analysis represents a substantial advancement of our ability to forecast smoking-attributable burden. To more accurately translate the effects of smoking prevalence to health burden, we built a novel framework for forecasting the health burden of tobacco use, based on the framework established in GBD. This framework allowed us to directly translate prevalence changes to population attributable fractions and summary exposure values and leverage the Future Health Scenarios platform to translate these measures into the health effects of these scenarios for all countries and causes measured by GBD. Furthermore, it allows us to quantify the maximum future health gains possible through smoking elimination, as well as the population dynamics that would result through such elimination. We are able to capture excess future risk among former smokers due to past smoking exposure. Our analysis shows that smoking intervention would result in longer lives lived by current and former smokers. This analysis also allows us to capture the important effect of competing risks. Rather than dying prematurely from ischaemic heart disease or lung cancer, individuals would gain years of life. However, they inevitably would die later, either from causes attributable to other risk factors, or from illnesses related to older age. The fluid, time-varying nature of the potential health gains were important in our decision to use YLLs rather than deaths as our measure of interest. We present cumulative YLLs rather than annual YLLs throughout this analysis for similar reasons. The question is not whether people will die, either due to smoking or another driving factor, but rather how much longer their lives could be in the absence of smoking. Further work is needed to understand these dynamic relationships.

Our analysis likely underestimates the total benefits of smoking elimination in two ways. First, since 62·4% (95% UI 61·1–63·6) of smoking-attributable burden in 2021 was among individuals aged 60 years and older, and our simulation's first smoke-free generation will be at most aged 57–61 years by 2050, the majority of health gains from the smoke-free generation component of our smoking elimination scenarios will not be observed until after 2050. As a result, health gains from smoking elimination will continue to grow well beyond 2050. Second, the full health gains possible under the alternative scenarios will not begin to be realised until enough time has passed that all residual risk among former smokers has dissipated. Under the Elimination-2050 scenario, this would not occur until approximately 2080, 30 years after the last smokers quit. These factors highlight the importance of using a package of policies that include efforts directed at helping current smokers reduce their consumption and quit. In future studies, we plan to extend the forecasts to at least 2100, so that we can follow the relevant cohorts to old age and thus capture the full effect of the smoking elimination scenarios.

Our analysis has several limitations. First, we analysed the direct effects of reductions in smoking prevalence on disease burden. Although we did not quantify them, we anticipate additional health benefits under these scenarios in terms of reductions in second-hand smoke exposure. Second, while our analysis considers additional years of life possible with smoking prevalence reduction, we did not quantify the benefits to quality of life lived free of disease. Further health benefits would likely occur under these scenarios in terms of more years lived without disability. Third, our forecast model assumes that the age-specific smoking initiation rates, distributions of cigarette-equivalents smoked per day by current smokers, pack-years among current smokers, and years since quitting among former smokers would remain constant into the future. This assumption is consistent with observed data that smoking intensity is largely stable over time, even as prevalence varies. Fourth, our alternative scenarios only act to reduce smoking prevalence, and do not include additional benefits that could be achieved by reducing smoking intensity. Fifth, we do not model substitution with electronic nicotine delivery systems and assume that recent quitters take on the risk profile of former tobacco smokers, not current users of electronic nicotine delivery systems. Sixth, the reference scenario that is used as the benchmark against which potential health gains are measured assumes that past trends in drivers of health persist. As a result of this assumption, our reference forecasts reflect historical progress in the reduction of many causes of health burden, such as communicable, maternal, neonatal, and nutritional diseases, that resulted from substantial investment. For the future forecast under this scenario to be realised, this investment will need to continue. Similarly, each of our scenarios assume that the relationship between drivers of health, such as smoking, and health outcomes will continue into the future. If improvements in lung cancer detection or treatment accelerate, the need for smoking elimination would be reduced. Seventh, the past data that we forecast comes from GBD, which carries its own limitations. For instance, data on tobacco use is self-reported, which is subject to under-reporting and social desirability bias, particularly in demographics among which smoking is not socially accepted. Finally, we do not quantify the health gains possible under a worse scenario in which smoking exposure intensifies relative to past trends, or other scenarios in which other risk factor exposure does not continue as it has in the past. Despite these limitations, our analysis represents an important quantification of the health gains possible under various smoking prevalence scenarios.

This study is an advancement of our ability to forecast smoking-attributable burden for every country and cause represented in the GBD. Although global smoking prevalence will continue to decrease in the reference scenario, substantial burden will still accumulate in the future due to smoking. Smoking elimination, even by 2050, would result in gains in life expectancy at birth at a rate not possible by many other interventions. Further research, with forecasts to 2100 encompassing avoidable secondhand smoke burden, is needed to generate a more complete understanding of the gains possible with smoking elimination. Accelerated adoption of anti-tobacco policies in all countries is needed, and equally, existing policies must be maintained to build upon the gains won since the adoption of the Framework Convention on Tobacco Control. Timely action is crucial to ensure the maximum amount of health burden is avoided in the coming decades.

### GBD 2021 Tobacco Forecasting Collaborators

### Affiliations

### Contributors

### Data sharing

To download the GBD data used in these analyses, please visit the Global Health Data Exchange GBD 2021 website (https://ghdx.healthdata.org/gbd-2021/sources). To download forecasted estimates used in these analyses, please visit the GBD tobacco forecasting visualisation tool (https://vizhub.healthdata.org/tobacco-forecasting/).

## Declaration of interests

S Afzal reports support for the present manuscript from HEC Digital Library Pakistan. S Afzal reports payment or honoraria for lectures, presentations, speakers bureaus, manuscript writing, or educational events from King Edward Medical University and collaborative partners including University of Johns Hopkins, University of California, and University of Massachusetts; participation on a Data Safety Monitoring Board or Advisory Board with National Bioethics Committee Pakistan, King Edward Medical University Institutional Ethical Review Board, and Ethical Review Board Fatima Jinnah Medical University and Sir Ganga Ram Hospital; leadership or fiduciary roles in board, society, committee or advocacy groups, paid or unpaid, with Pakistan Association of Medical Editors, Faculty of Public Health Royal Colleges UK (FFPH) as a fellow, Society of Prevention, Advocacy And Research, King Edward Medical University (SPARK), and Pakistan Society of Infectious Diseases as a member; and other financial or non-financial interests from Public Health and Preventive Medicine at King Edward Medical University as Dean, Annals of King Edward Medical University as Chief Editor, Quality Enhancement Cell at King Edward Medical University as director, Research and Publications Higher Education Commission Pakistan as a member; all outside the submitted work. A Biswas reports consulting fees from Lupin Pharmaceuticals, India, Alkem Laboratories. India, Intas Pharmaceuticals, India, Eisai Pharmaceuticals, India, and Torrent Pharmaceuticals, India, all outside the submitted work. R Bai reports support for the present manuscript from the Social Science Fund of Jiangsu Province (grant number 21GLD008) and the Fundamental Research Funds for the Central Universities (grant number 30923011101). M L Bell reports grants or contracts paid to their institution from US Environmental Protection Agency, National Institutes of Health (NIH; USA), High Tide Foundation, Health Effects Institute, Yale Women Faculty Forum, Environmental Defense Fund, Wellcome Trust Foundation, Yale Climate Change and Health Center, Robert Wood Johnson Foundation, and Hutchinson Postdoctoral Fellowship; consulting fees from Clinique, ToxiMap, and SciQuest; payment or honoraria for lectures, presentations, speakers bureaus, manuscript writing or educational events from Colorado School of Public Health, Duke University, University of Texas, Data4Justice, Korea University, Organization of Teratology Information Specialists, University of Pennsylvania, Boston University, IOP Publishing, NIH, Health Canada, EHS, PAC-10, UK Research and Innovation, AXA Research Fund Fellowship, Harvard University, University of Montana, and SciQuest; support for attending meetings and/or travel from Colorado School of Public Health, University of Texas, Duke University, Boston University, University of Pennsylvania, Harvard University, American Journal of Public Health, Columbia University, Community Modeling and Analysis System (CMAS) conference, Nature conference; leadership or fiduciary roles in other board, society, committee or advocacy groups, unpaid, from Fifth National Climate Assessment, Lancet Countdown, Johns Hopkins EHE Advisory Board, Harvard external advisory committee (for training grant), WHO Global Air Pollution and Health Technical Advisory group, National Academies Panels and Committees, and from US EPA Clean Air Scientific Advisory Committee (CASAC; paid); all outside the submitted work. L Belo reports other financial or non-financial support from FCT in the scope of the project UIDP/04378/2020 and UIDB/04378/2020 of UCIBIO and the project LA/P/0140/2020 of i4HB; outside the submitted work. S Bhaskar reports grants or contracts from Japan Society for the Promotion of Science (JSPS) and Japanese Ministry of Education, Culture, Sports, Science and Technology (MEXT) for a grant-in-aid for Scientific Research (KAKENHI; P23712), and from JSPS and the Australian Academy of Science for a JSPS International Fellowship (P23712); leadership or fiduciary roles in board, society, committee or advocacy groups, paid or unpaid with Rotary District 9675, Sydney, Australia (District Chair, Diversity, Equity & Inclusion), Global Health & Migration Hub Community, Global Health Hub Germany, Berlin, Germany (Chair, Founding Member and Manager), PLoS One, BMC Neurology, Frontiers in Neurology, Frontiers in Stroke, Frontiers in Public Health, Journal of Aging Research & BMC Medical Research Methodology (Editorial Board Member), College of Reviewers, Canadian Institutes of Health Research (CIHR), Government of Canada (Member), World Headache Society, Bengaluru, India (Director of Research), Cariplo Foundation, Milan, Italy (Expert Adviser/Reviewer), National Cerebral and Cardiovascular Center, Department of Neurology, Suita, Osaka, Japan (Visiting Director), and Cardiff University Biobank, Cardiff, UK (Member, Scientific Review Committee); all outside the submitted work. E J Boyko reports payment or honoraria for lectures, presentations, speakers bureaus, manuscript writing or educational events from Korean Diabetes Association, International Society for the Diabetic Foot, Diabetes Association of the R.O.C. (Taiwan), American Diabetes Association; support for attending meetings and/or travel from Korean Diabetes Association, Diabetes Association of the R.O.C. (Taiwan), International Society for the Diabetic Foot; all outside the submitted work. J Conde reports grants or contracts from European Research Council Starting Grant (ERC-StG-2019-848325. Funding €1.5M); patents planned, issued or pending Surfactant-Based Hydrogel, Methods and Uses Thereof with Universidade Nova de Lisboa; all outside the submitted work. G F Gil reports grants or contracts from Bill & Melinda Gates Foundation through role as researcher on the GEM team at Institute for Health Metrics and Evaluation (IHME); support for attending meetings and/or travel from University of Washington (Graduate and Professional Student Senate) paid through IHME; all outside the submitted work. A Guha reports grants or contracts from American Heart Association and Department of Defense (USA); consulting fees with Pfizer and Novartis; leadership or fiduciary roles in other board, society, committee or advocacy groups, paid or unpaid with ZERO Prostate Cancer (health equity task force); all outside the submitted work. A Hassan reports consulting fees from Novartis, Sanofi Genzyme, Biologix, Merck, Hikma Pharmaceuticals, Janssen, Inspire Pharma, Future Pharma, Elixir Pharma; payment or honoraria for lectures, presentations, speakers bureaus, manuscript writing or educational events from Novartis, Allergan, Merck, Biologix, Janssen, Roche, Sanofi Genzyme, Bayer, Hikma Pharmaceuticals, Al Andalus, Chemipharm, Lundbeck, Inspire Pharma, Future Pharma and Habib Scientific Office, and EVER Pharma; support for attending meetings and/or travel from Novartis, Allergan, Merck, Biologix, Roche, Sanofi Genzyme, Bayer, Hikma Pharmaceuticals, Chemipharm, and Al Andalus and Clavita Pharm; leadership or fiduciary roles in other board, society, committee or advocacy groups, paid or unpaid with the Egyptian Society of Neurology (board member of headache chapter), and International Headache Society (committee of education member, regional committee member); all outside the submitted work. C Herteliu reports grants or contracts from Romanian Ministry of Research, Innovation and Digitalization through UEFISCDI (Project “Analysis of the impact of Covid-19 on the main demographic indicators in Romania and the Republic of Moldova by using econometric modeling” code PN-IV-P8-8.3-ROMD-2023-0208), European Commision Horizon 4P-CAN (Personalised Cancer Primary Prevention Research through Citizen Participation and Digitally Enabled Social Innovation), European Union – NextgenerationEU and Romanian Government, under National Recovery and Resilience Plan for Romania (Project “Societal and Economic Resilience within multi-hazards environment in Romania,” contract number 760050/ 23.05.2023, cod PNRR-C9-I8-CF 267/ 29.11.2022, through the Romanian Ministry of Research, Innovation and Digitalization, within Component 9, Investment I8)(Project “A better understanding of socio-economic systems using quantitative methods from Physics,” contract number 760034/ 23.05.2023, cod PNRR-C9-I8-CF 255/ 29.11.2022, through the Romanian Ministry of Research, Innovation and Digitalization, within Component 9, Investment I8); all outside the submitted work. I M Ilic reports support for the present manuscript from Ministry of Education, Science and Technological development, Republic of Serbia (project No 175042, 2011-2023). M D Ilic reports support for the present manuscript from Ministry of Science, Technological Development and Innovation of the Republic of Serbia (number 451-03-47/2023-01/200111). T Joo reports support for the present manuscript from National Research, Development and Innovation Office in Hungary (RRF-2.3.1-21-2022-00006), Data-Driven Health Division of National Laboratory for Health Security. J Jozwiak reports payment or honoraria for lectures, presentations, speakers bureaus, manuscript writing or educational events from Novartis, Adamed, and Amgen outside the submitted work. S V Katikireddi reports support for the present manuscript, paid to their institution, from Medical Research Council (MC_UU_00022/2), Scottish Government Chief Scientist Office (SPHSU17), and European Research Council (949582). K Krishan reports non-financial support from the UGC Centre of Advanced Study, CAS II, awarded to the Department of Anthropology, Panjab University, Chandigarh, India, outside the submitted work. J L Leasher reports leadership or fiduciary roles in other board, society, committee or advocacy groups, unpaid, with Member Planning Group for National Eye Health Education Program, National Eye Institute, USA outside the submitted work. H R Marateb reports grants or contracts paid to their institution, Universitat Politècnica de Catalunya–Barcelona Tech (UPC), from The Beatriu de Pinós post-doctoral programme from the Office of the Secretary of Universities and Research from the Ministry of Business and Knowledge of the Government of Catalonia (programme: 2020 BP 00261) outside the submitted work. E Mathews reports grants and contracts from the DBT India Alliance/Wellcome Trust (Clinical and Public Health Early Career Fellowship, grant number IA/CPHE/17/1/503345) outside the submitted work. S A Meo reports grants or contracts from Researchers Supporting Project, King Saud University, Riyadh, Saudi Arabia (RSP-2024 R47) outside the submitted work. R S Moreira reports grants or contracts from the National Council for Scientific and Technological Development (CNPq) for a CNPq Research Productivity Scholarship (scholarship registration number: 316607/2021-5) outside the submitted work. S Nomura reports support for the present manuscript from the Ministry of Education, Culture, Sports, Science and Technology of Japan (24H00663) and from the Japan Science and Technology Agency for Precursory Research for Embryonic Science and Technology (JPMJPR22R8). O O Odukoya reports grants or contracts from the Northwestern/Nigeria Research Training Program in HIV and Malignancies (NN-HAM; 2D43TW009575-11) outside the submitted work. A P Okekunle reports support for the present manuscript from the National Research Foundation of Korea funded by the Ministry of Science and ICT (2020H1D3A1A04081265). A P Okekunle reports support for attending meetings and/or travel from National Research Foundation of Korea funded by the Ministry of Science and ICT (2020H1D3A1A04081265) outside the submitted work. R F Palma-Alvarez reports payment or honoraria for lectures, presentations, speakers bureaus, manuscript writing or educational events from Angelini, Casen-Recordati, Exeltis, Lundbeck, Takeda, and Neuraxpharm outside the submitted work. R Passera reports participation on a Data Safety Monitoring Board or Advisory Board (unpaid) for “Consolidation with ADCT-402 (loncastuximab tesirine) after immunochemotherapy: a phase II study in BTKi-treated/ineligible Relapse/Refractory Mantle Cell Lymphoma (MCL) patients” with Fondazione Italiana Linfomi (FIL), Alessandria; leadership or fiduciary roles in other board, society, committee or advocacy groups (unpaid) as Member of the EBMT Statistical Committee, European Society for Blood and Marrow Transplantation, Paris (FRANCE) and past member 2020-2023 (biostatistician) of the IRB/IEC Comitato Etico AO SS. Antonio e Biagio Alessandria-ASL AL-VC (ITALY); all outside the submitted work. Y L Samodra reports grants or contracts from Taipei Medical University; leadership or fiduciary roles in other board, society, committee or advocacy groups, paid or unpaid, with Benang Merah Research Center, Indonesia as co-founder; all outside the submitted work. J Sanabria reports support for attending meetings and/or travel from the University Medical School for Continuing Medical Education (CME); three patents granted and two pending, no royalties; all outside the submitted work. B M Schaarschmidt reports grants or contracts from Else Kröner-Fresenius Foundation, Deutsche Forschungsgemeinschaft, and PharmaCept GmbH; payment or honoraria for lectures, presentations, speakers bureaus, manuscript writing or educational events from AstraZeneca; support for travel from Bayer; all outside the submitted work. V Sharma reports other financial or non-financial support from DFSS (MHA)'s research project (DFSS28(1)2019/EMR/6) at Institute of Forensic Science & Criminology, Panjab University, Chandigarh, India, outside the submitted work. V Shivarov reports stock or stock options from ICON; other financial or non-financial interests CON/PRAHS via a salary; all outside the submitted work. S Shrestha reports other financial or non-financial support from the School of Pharmacy, Monash University Malaysia via the Graduate Research Merit Scholarship outside the submitted work. J P Silva reports support for the present manuscript from the Portuguese Foundation for Science and Technology via payment of a salary (contract with reference 2021.01789.CEECIND/CP1662/CT0014). J A Singh reports consulting fees from ROMTech, Atheneum, Clearview Healthcare Partners, American College of Rheumatology, Yale University, Hulio, Horizon Pharmaceuticals, DINORA, ANI/Exeltis, USA, Frictionless Solutions, Schipher, Crealta/Horizon, Medisys, Fidia, PK Med, Two Labs, Adept Field Solutions, Clinical Care Options, Putnam Associates, Focus Forward, Navigant Consulting, Spherix, MedIQ, Jupiter Life Science, UBM LLC, Trio Health, Medscape, WebMD, Practice Point Communications, and the National Institutes of Health (USA); payment or honoraria for speakers bureaus from Simply Speaking; past support for attending meetings and/or travel from OMERACT as a steering committee member; participation on a Data Safety Monitoring Board or Advisory Board (unpaid) with the Food and Drug Administration (USA) Arthritis Advisory Committee; leadership or fiduciary roles in other board, society, committee or advocacy groups with OMERACT as past steering committee member (paid), the Veterans Affairs Rheumatology Field Advisory Committee as Chair (unpaid), and the UAB Cochrane Musculoskeletal Group Satellite Center on Network Meta-analysis as Editor and Director (unpaid); stock or stock options in Atai Life Sciences, Kintara Therapeutics, Intelligent Biosolutions, Acumen Pharmaceutical, TPT Global Tech, Vaxart Pharmaceuticals, Atyu Biopharma, Adaptimmune Therapeutics, GeoVax Labs, Pieris Pharmaceuticals, Enzolytics, Seres Therapeutics, Tonix Pharmaceuticals Holding Corp., Aebona Pharmaceuticals, Charlotte's Web Holdings, and previously owned stock options in Amarin, Viking, and Moderna Pharmaceuticals; all outside the submitted work. M Solmi reports payment or honoraria for advisory boards or educational events from AbbVie, Lundbeck, and Otsuka, outside the submitted work. D J Stein reports personal fees from Discovery Vitality, Johnson & Johnson, Kanna, L’Oreal, Lundbeck, Orion, Sanofi, Servier, Takeda and Vistagen, outside the submitted work. J H V Ticoalu reports leadership or fiduciary roles in board, society, committee or advocacy groups, paid or unpaid with Benang Merah Research Center, Indonesia as co-founder, outside the submitted work. P Willeit reports consulting fees from Novartis Pharmaceuticals, outside the submitted work. M Zielińska reports other financial or non-financial support as an AstraZeneca employee, outside the submitted work.
