# Supplementary material for: Forecasting the effects of smoking prevalence scenarios on years of life lost and life expectancy from 2022 to 2050: a systematic analysis for the Global Burden of Disease Study 2021
Source: Lancet Public Health. 2024 Oct 2;9(10):e729–44. doi: 10.1016/S2468-2667(24)00166-X (PMC11447278; doi:10.1016/S2468-2667(24)00166-X)
Supplement: Supplementary appendix 1 [file mmc1.pdf]

THE LANCET
Public Health

Supplementary appendix 1

This appendix formed part of the original submission and has been peer reviewed. We post it as supplied by the authors.

Supplement to: GBD 2021 Tobacco Forecasting Collaborators. Forecasting the effects of smoking prevalence scenarios on years of life lost and life expectancy from 2022 to 2050: a systematic analysis for the Global Burden of Disease Study 2021. *Lancet Public Health* 2024; **9:** e729–44.

Appendix 1: methods appendix to “Forecasting the impacts of smoking prevalence scenarios on years of life lost and life expectancy from 2022 to 2050 - a systematic analysis for the Global Burden of Disease Study 2021”

This appendix provides further methodological detail for “Forecasting the impacts of smoking prevalence scenarios on years of life lost and life expectancy from 2022 to 2050 - a systematic analysis for the Global Burden of Disease Study 2021”.

Table of Contents

GEOGRAPHIES ESTIMATED 3

DEMOGRAPHICS 9

MODEL FLOWCHART 10

INDEPENDENT DRIVERS FORECASTING OVERVIEW 11

SMOKING FORECASTING OVERVIEW 11

DIFFERENTIAL MORTALITY 13

RISK REDUCTION CURVES 14

ILLUSTRATIVE COHORT 15

GATHER CHECKLIST 16

REFERENCES 17

Geographies Estimated

This study utilized the standard geographical hierarchy for the Global Burden of Diseases, Injuries, and Risk Factors Study 2021 (GBD 2021). The 204 countries and territories included in this analysis are nested in 21 aggregate regions and seven aggregate super-regions. The regions and super-regions in the GBD are defined based on a combination of epidemiologic patterns and spatial distance.

Appendix Table S1. GBD location hierarchy used for estimation

Location	Region	Super Region
Armenia	Central Asia	Central Europe, Eastern Europe, and Central Asia
Azerbaijan	Central Asia	Central Europe, Eastern Europe, and Central Asia
Georgia	Central Asia	Central Europe, Eastern Europe, and Central Asia
Kazakhstan	Central Asia	Central Europe, Eastern Europe, and Central Asia
Kyrgyzstan	Central Asia	Central Europe, Eastern Europe, and Central Asia
Mongolia	Central Asia	Central Europe, Eastern Europe, and Central Asia
Tajikistan	Central Asia	Central Europe, Eastern Europe, and Central Asia
Turkmenistan	Central Asia	Central Europe, Eastern Europe, and Central Asia
Uzbekistan	Central Asia	Central Europe, Eastern Europe, and Central Asia
Albania	Central Europe	Central Europe, Eastern Europe, and Central Asia
Bosnia and Herzegovina	Central Europe	Central Europe, Eastern Europe, and Central Asia
Bulgaria	Central Europe	Central Europe, Eastern Europe, and Central Asia
Croatia	Central Europe	Central Europe, Eastern Europe, and Central Asia
Czechia	Central Europe	Central Europe, Eastern Europe, and Central Asia
Hungary	Central Europe	Central Europe, Eastern Europe, and Central Asia
Montenegro	Central Europe	Central Europe, Eastern Europe, and Central Asia
North Macedonia	Central Europe	Central Europe, Eastern Europe, and Central Asia
Poland	Central Europe	Central Europe, Eastern Europe, and Central Asia
Romania	Central Europe	Central Europe, Eastern Europe, and Central Asia
Serbia	Central Europe	Central Europe, Eastern Europe, and Central Asia
Slovakia	Central Europe	Central Europe, Eastern Europe, and Central Asia
Slovenia	Central Europe	Central Europe, Eastern Europe, and Central Asia
Belarus	Eastern Europe	Central Europe, Eastern Europe, and Central Asia
Estonia	Eastern Europe	Central Europe, Eastern Europe, and Central Asia
Latvia	Eastern Europe	Central Europe, Eastern Europe, and Central Asia
Lithuania	Eastern Europe	Central Europe, Eastern Europe, and Central Asia
Republic of Moldova	Eastern Europe	Central Europe, Eastern Europe, and Central Asia
Russian Federation	Eastern Europe	Central Europe, Eastern Europe, and Central Asia
Ukraine	Eastern Europe	Central Europe, Eastern Europe, and Central Asia
Australia	Australasia	High-income

New Zealand	Australasia	High-income
Brunei Darussalam	High-income Asia Pacific	High-income
Japan	High-income Asia Pacific	High-income
Republic of Korea	High-income Asia Pacific	High-income
Singapore	High-income Asia Pacific	High-income
Canada	High-income North America	High-income
Greenland	High-income North America	High-income
United States of America	High-income North America	High-income
Argentina	Southern Latin America	High-income
Chile	Southern Latin America	High-income
Uruguay	Southern Latin America	High-income
Andorra	Western Europe	High-income
Austria	Western Europe	High-income
Belgium	Western Europe	High-income
Cyprus	Western Europe	High-income
Denmark	Western Europe	High-income
Finland	Western Europe	High-income
France	Western Europe	High-income
Germany	Western Europe	High-income
Greece	Western Europe	High-income
Iceland	Western Europe	High-income
Ireland	Western Europe	High-income
Israel	Western Europe	High-income
Italy	Western Europe	High-income
Luxembourg	Western Europe	High-income
Malta	Western Europe	High-income
Monaco	Western Europe	High-income
Netherlands	Western Europe	High-income
Norway	Western Europe	High-income
Portugal	Western Europe	High-income
San Marino	Western Europe	High-income
Spain	Western Europe	High-income
Sweden	Western Europe	High-income
Switzerland	Western Europe	High-income
United Kingdom	Western Europe	High-income
Bolivia (Plurinational State of)	Andean Latin America	Latin America and Caribbean
Ecuador	Andean Latin America	Latin America and Caribbean
Peru	Andean Latin America	Latin America and Caribbean
Antigua and Barbuda	Caribbean	Latin America and Caribbean

Bahamas	Caribbean	Latin America and Caribbean
Barbados	Caribbean	Latin America and Caribbean
Belize	Caribbean	Latin America and Caribbean
Bermuda	Caribbean	Latin America and Caribbean
Cuba	Caribbean	Latin America and Caribbean
Dominica	Caribbean	Latin America and Caribbean
Dominican Republic	Caribbean	Latin America and Caribbean
Grenada	Caribbean	Latin America and Caribbean
Guyana	Caribbean	Latin America and Caribbean
Haiti	Caribbean	Latin America and Caribbean
Jamaica	Caribbean	Latin America and Caribbean
Puerto Rico	Caribbean	Latin America and Caribbean
Saint Kitts and Nevis	Caribbean	Latin America and Caribbean
Saint Lucia	Caribbean	Latin America and Caribbean
Saint Vincent and the Grenadines	Caribbean	Latin America and Caribbean
Suriname	Caribbean	Latin America and Caribbean
Trinidad and Tobago	Caribbean	Latin America and Caribbean
United States Virgin Islands	Caribbean	Latin America and Caribbean
Colombia	Central Latin America	Latin America and Caribbean
Costa Rica	Central Latin America	Latin America and Caribbean
El Salvador	Central Latin America	Latin America and Caribbean
Guatemala	Central Latin America	Latin America and Caribbean
Honduras	Central Latin America	Latin America and Caribbean
Mexico	Central Latin America	Latin America and Caribbean
Nicaragua	Central Latin America	Latin America and Caribbean
Panama	Central Latin America	Latin America and Caribbean
Venezuela (Bolivarian Republic of)	Central Latin America	Latin America and Caribbean
Brazil	Tropical Latin America	Latin America and Caribbean
Paraguay	Tropical Latin America	Latin America and Caribbean
Afghanistan	North Africa and Middle East	North Africa and Middle East
Algeria	North Africa and Middle East	North Africa and Middle East
Bahrain	North Africa and Middle East	North Africa and Middle East
Egypt	North Africa and Middle East	North Africa and Middle East
Iran (Islamic Republic of)	North Africa and Middle East	North Africa and Middle East
Iraq	North Africa and Middle East	North Africa and Middle East
Jordan	North Africa and Middle East	North Africa and Middle East
Kuwait	North Africa and Middle East	North Africa and Middle East
Lebanon	North Africa and Middle East	North Africa and Middle East

Libya	North Africa and Middle East	North Africa and Middle East
Morocco	North Africa and Middle East	North Africa and Middle East
Oman	North Africa and Middle East	North Africa and Middle East
Palestine	North Africa and Middle East	North Africa and Middle East
Qatar	North Africa and Middle East	North Africa and Middle East
Saudi Arabia	North Africa and Middle East	North Africa and Middle East
Sudan	North Africa and Middle East	North Africa and Middle East
Syrian Arab Republic	North Africa and Middle East	North Africa and Middle East
Tunisia	North Africa and Middle East	North Africa and Middle East
Turkey	North Africa and Middle East	North Africa and Middle East
United Arab Emirates	North Africa and Middle East	North Africa and Middle East
Yemen	North Africa and Middle East	North Africa and Middle East
Bangladesh	South Asia	South Asia
Bhutan	South Asia	South Asia
India	South Asia	South Asia
Nepal	South Asia	South Asia
Pakistan	South Asia	South Asia
China	East Asia	Southeast Asia, East Asia, and Oceania
Democratic People's Republic of Korea	East Asia	Southeast Asia, East Asia, and Oceania
Taiwan (Province of China)	East Asia	Southeast Asia, East Asia, and Oceania
American Samoa	Oceania	Southeast Asia, East Asia, and Oceania
Cook Islands	Oceania	Southeast Asia, East Asia, and Oceania
Fiji	Oceania	Southeast Asia, East Asia, and Oceania
Guam	Oceania	Southeast Asia, East Asia, and Oceania
Kiribati	Oceania	Southeast Asia, East Asia, and Oceania
Marshall Islands	Oceania	Southeast Asia, East Asia, and Oceania
Micronesia (Federated States of)	Oceania	Southeast Asia, East Asia, and Oceania
Nauru	Oceania	Southeast Asia, East Asia, and Oceania
Niue	Oceania	Southeast Asia, East Asia, and Oceania
Northern Mariana Islands	Oceania	Southeast Asia, East Asia, and Oceania
Palau	Oceania	Southeast Asia, East Asia, and Oceania
Papua New Guinea	Oceania	Southeast Asia, East Asia, and Oceania
Samoa	Oceania	Southeast Asia, East Asia, and Oceania
Solomon Islands	Oceania	Southeast Asia, East Asia, and Oceania
Tokelau	Oceania	Southeast Asia, East Asia, and Oceania
Tonga	Oceania	Southeast Asia, East Asia, and Oceania
Tuvalu	Oceania	Southeast Asia, East Asia, and Oceania
Vanuatu	Oceania	Southeast Asia, East Asia, and Oceania

Cambodia	Southeast Asia	Southeast Asia, East Asia, and Oceania
Indonesia	Southeast Asia	Southeast Asia, East Asia, and Oceania
Lao People's Democratic Republic	Southeast Asia	Southeast Asia, East Asia, and Oceania
Malaysia	Southeast Asia	Southeast Asia, East Asia, and Oceania
Maldives	Southeast Asia	Southeast Asia, East Asia, and Oceania
Mauritius	Southeast Asia	Southeast Asia, East Asia, and Oceania
Myanmar	Southeast Asia	Southeast Asia, East Asia, and Oceania
Philippines	Southeast Asia	Southeast Asia, East Asia, and Oceania
Seychelles	Southeast Asia	Southeast Asia, East Asia, and Oceania
Sri Lanka	Southeast Asia	Southeast Asia, East Asia, and Oceania
Thailand	Southeast Asia	Southeast Asia, East Asia, and Oceania
Timor-Leste	Southeast Asia	Southeast Asia, East Asia, and Oceania
Viet Nam	Southeast Asia	Southeast Asia, East Asia, and Oceania
Angola	Central Sub-Saharan Africa	Sub-Saharan Africa
Central African Republic	Central Sub-Saharan Africa	Sub-Saharan Africa
Congo	Central Sub-Saharan Africa	Sub-Saharan Africa
Democratic Republic of the Congo	Central Sub-Saharan Africa	Sub-Saharan Africa
Equatorial Guinea	Central Sub-Saharan Africa	Sub-Saharan Africa
Gabon	Central Sub-Saharan Africa	Sub-Saharan Africa
Burundi	Eastern Sub-Saharan Africa	Sub-Saharan Africa
Comoros	Eastern Sub-Saharan Africa	Sub-Saharan Africa
Djibouti	Eastern Sub-Saharan Africa	Sub-Saharan Africa
Eritrea	Eastern Sub-Saharan Africa	Sub-Saharan Africa
Ethiopia	Eastern Sub-Saharan Africa	Sub-Saharan Africa
Kenya	Eastern Sub-Saharan Africa	Sub-Saharan Africa
Madagascar	Eastern Sub-Saharan Africa	Sub-Saharan Africa
Malawi	Eastern Sub-Saharan Africa	Sub-Saharan Africa
Mozambique	Eastern Sub-Saharan Africa	Sub-Saharan Africa
Rwanda	Eastern Sub-Saharan Africa	Sub-Saharan Africa
Somalia	Eastern Sub-Saharan Africa	Sub-Saharan Africa
South Sudan	Eastern Sub-Saharan Africa	Sub-Saharan Africa
Uganda	Eastern Sub-Saharan Africa	Sub-Saharan Africa
United Republic of Tanzania	Eastern Sub-Saharan Africa	Sub-Saharan Africa
Zambia	Eastern Sub-Saharan Africa	Sub-Saharan Africa
Botswana	Southern Sub-Saharan Africa	Sub-Saharan Africa
Eswatini	Southern Sub-Saharan Africa	Sub-Saharan Africa
Lesotho	Southern Sub-Saharan Africa	Sub-Saharan Africa
Namibia	Southern Sub-Saharan Africa	Sub-Saharan Africa

South Africa	Southern Sub-Saharan Africa	Sub-Saharan Africa
Zimbabwe	Southern Sub-Saharan Africa	Sub-Saharan Africa
Benin	Western Sub-Saharan Africa	Sub-Saharan Africa
Burkina Faso	Western Sub-Saharan Africa	Sub-Saharan Africa
Cabo Verde	Western Sub-Saharan Africa	Sub-Saharan Africa
Cameroon	Western Sub-Saharan Africa	Sub-Saharan Africa
Chad	Western Sub-Saharan Africa	Sub-Saharan Africa
Côte d'Ivoire	Western Sub-Saharan Africa	Sub-Saharan Africa
Gambia	Western Sub-Saharan Africa	Sub-Saharan Africa
Ghana	Western Sub-Saharan Africa	Sub-Saharan Africa
Guinea	Western Sub-Saharan Africa	Sub-Saharan Africa
Guinea-Bissau	Western Sub-Saharan Africa	Sub-Saharan Africa
Liberia	Western Sub-Saharan Africa	Sub-Saharan Africa
Mali	Western Sub-Saharan Africa	Sub-Saharan Africa
Mauritania	Western Sub-Saharan Africa	Sub-Saharan Africa
Niger	Western Sub-Saharan Africa	Sub-Saharan Africa
Nigeria	Western Sub-Saharan Africa	Sub-Saharan Africa
Sao Tome and Principe	Western Sub-Saharan Africa	Sub-Saharan Africa
Senegal	Western Sub-Saharan Africa	Sub-Saharan Africa
Sierra Leone	Western Sub-Saharan Africa	Sub-Saharan Africa
Togo	Western Sub-Saharan Africa	Sub-Saharan Africa

Demographics

We produced estimates by sex and five-year GBD age group from 2022 to 2050 for the 204 countries and territories included in this analysis. We also produced estimates aggregated by age:

- All-age estimates reflect population-weighted average estimates for ages 0 and above.
- Age-standardized estimates reflect weighting according to the GBD population standard.

Model Flowchart

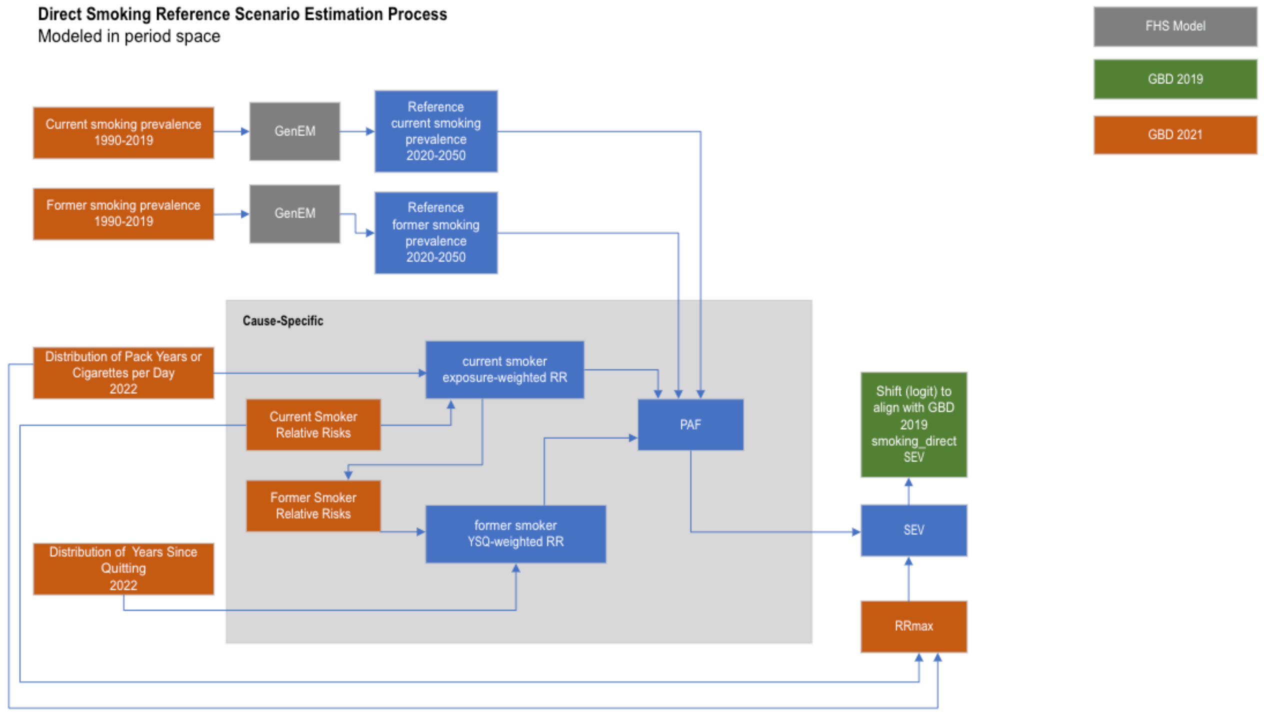

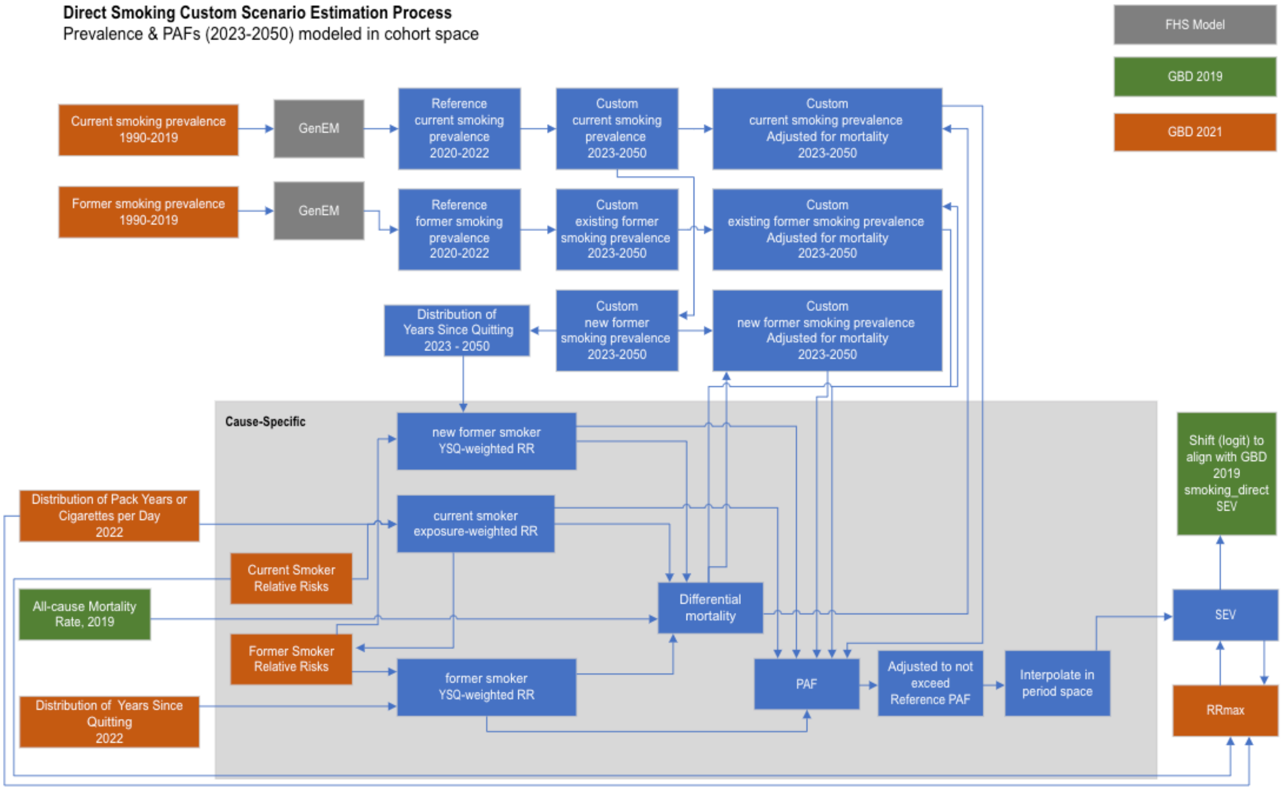

Independent Drivers Forecasting Overview

We obtained estimates of independent drivers of health, including over 70 GBD risk factor summary exposure values (SEVs), interventions such as vaccines and antiretroviral therapy (ART) coverage, and covariates such as sociodemographic index (SDI, a composite measure of income, education, and fertility under age 25) from the Global Burden of Disease (GBD) Study, for every location, age, and sex, from 1990 to 2019. These independent variables were then forecasted to 2050 mostly using a generalised ensemble model (GenEM) that includes two main modelling approaches: annualised rate of change (ARC) and a two-stage spline model based on Meta-Regression Bayesian Regularized Trimmed Tool (MR-BRT).[1] Each of these sub-models had 6 different recency-weighting parameters ranging from 0 to 2.5 (the higher the weight, the more weight is given to recent years).[2] The weight of each sub-model was determined by running out-of-sample predictive validity, training each sub-model on data from 1990-2009 and validated based on 2010-2019 GBD estimates. We measured each child model's performance using root mean-squared error (RMSE) based on which we determined sampling weights of each child model. Then we produced the sub-model forecasts based on the 1990-2019 training dataset with 500 draws for each sub-model and sampled the draws according to the RMSE in the training dataset to obtain the final ensemble forecasts.

Full description of framework for forecasting independent drivers can be found in Vollset et al. 2024 main paper and appendix.[3] Once we have a complete time series from 1990 to 2050 for each of the independent variables, we use them to forecast cause-specific mortality rates through 2050.

Smoking Forecasting Overview

While most risk factor summary exposure values (SEVs) are forecast using GenEM as described above, the complexity inherent in smoking exposure meant that forecasting smoking SEVs required a modified approach.[3] Smoking SEVs computed within the GBD are composite measures of current, former, and never smoking prevalence, distributions of cigarette-equivalents smoked per day and pack-years smoked among current smokers, distributions of years since smoking cessation among former smokers, as well as the relative risks of smoking and risk reduction as a function of years since cessation for each of 32 causes. To allow us to frame our reference and alternative smoking prevalence scenarios in interpretable units, we chose to forecast smoking prevalence rather than the smoking SEV. We then calculated future smoking cause-specific population attributable fractions (PAFs), from which we computed future SEVs.

We first obtained historical time series of current smoking prevalence, former smoking prevalence, distributions of cigarette equivalents smoked per day among current smokers, distributions of pack-years smoked among current smokers, and distributions of years since quitting among former smokers from GBD 2021. We also obtained cause-specific relative risk estimates for both current and former smokers, from GBD 2021. To construct the reference scenario, we forecast current and former smoking prevalence through 2050 and assumed exposure distributions remained constant. From these estimates, we computed reference-scenario PAFs through 2050. For the reference scenario, we forecast current and former smoking prevalence in period space to preserve observed age patterns. In contrast, we model the

alternative scenarios in cohort space to capture the mechanisms by which the alternative scenarios are implemented. To construct the alternative scenarios, which begin in 2023, we use the reference scenario current and former smoking prevalence forecasts through 2022. We then construct four smoking status groups: current smokers, former smokers who quit in 2022 or earlier, former smokers who quit in 2023 or later, and never smokers. We explicitly model the prevalence of each of these groups for every year from 2023 to 2050, treating every 5-year age group in 2022 as a single cohort.

When forecasting the prevalence of different smoking groups, it is important to consider that current smokers have a higher relative risk of mortality than former smokers, who in turn have a higher risk than never smokers. While the reference scenario incorporates these differing mortality rates across smoking statuses by forecasting smoking prevalence in period space, differential mortality must be explicitly added to the alternative scenario prevalence forecasts. To account for the effects of different mortality rates between current, former, and never smokers, we estimated the all-cause relative risks of mortality by smoking status. We computed exposure-weighted relative risks by location, age, sex, and cause in 2022 and aggregated these cause-specific relative risks across all causes to generate an all-cause relative risk of mortality. Finally, we computed the mortality rate among never smokers and used each of the mortality rates to adjust our prevalence estimates in every future year.

Using these inputs, we calculated smoking PAFs for every location, sex, 5-year age group, and cause at 5-year intervals between 2022 and 2052, using the formula below, adapted from the GBD study (Eq. 1).

$$PAF = \frac{p(n)+p(f)\int \exp(x)*rr(x)+ p(f\prime)\int \exp(z)*rr(x)+p(c)\int \exp(y)*rr(y)-1}{p(n)+p(f)\int \exp(x)*rr(x)+p(f\prime)\int \exp(z)*rr(x)+p(c)\int \exp(y)*rr(y)} \quad (1)$$

Where $p(n)$ is the prevalence of never smokers, $p(f)$ is the prevalence of former smokers who quit in 2022 or earlier, $p(f')$ is the prevalence of former smokers who quit in 2023 or later, $exp(x)$ is a distribution of years since quitting among former smokers who quit in 2022 or earlier, $exp(z)$ is a uniform distribution of years since quitting among former smokers who quit in 2023 or later, $rr(x)$ is the relative risk for years since quitting, $p(c)$ is the prevalence of current smokers, $exp(y)$ is a distribution of cigarettes per smoker per day or pack-years, and $rr(y)$ is the relative risk for cigarettes per smoker per day or pack-years.

These PAFs were then interpolated in period space to obtain a full time series from 2019 to 2050. Where the reference forecast PAFs exceed alternative scenario PAFs, we cap the alternative scenario PAFs by the reference scenario. Finally, these PAFs, a well as the GBD 2021 smoking RRmax values are used to compute all-cause SEVs using Eq. 2 below.[4]

$$SEV = \frac{\frac{PAF_c}{1-PAF_c}}{RR_{max}-1} \quad (2)$$

Where c is cause. The causes for which we forecast smoking-attributable burden include:

- tuberculosis
- lower respiratory infections
- oesophageal cancer

- stomach cancer
- liver cancer
- larynx cancer
- tracheal, bronchus, and lung cancer
- breast cancer
- cervical cancer
- prostate cancer
- colon and rectum cancer
- lip and oral cavity cancer
- nasopharynx cancer
- other pharynx cancer
- pancreatic cancer
- kidney cancer
- bladder cancer
- leukaemia
- ischemic heart disease
- stroke
- atrial fibrillation and flutter
- aortic aneurysm
- lower extremity peripheral arterial disease
- chronic obstructive pulmonary disease
- asthma
- peptic ulcer disease
- gallbladder and biliary diseases
- Alzheimer's disease and other dementias
- Parkinson's disease
- multiple sclerosis
- diabetes mellitus
- rheumatoid arthritis

Final forecasts were shifted to align with GBD 2021 to reflect the newest available estimates.[3] The shift was applied in log space to all causes at both the aggregate and most-detailed levels. As a result, the most-detailed level values will not exactly sum to their aggregates in years beyond 2021.

Differential Mortality

To account for the effects of different mortality rates between current, former, and never smokers, we adjust the prevalence of current, former, and never smokers in each year.

We start by estimating the all-cause relative risks of mortality by smoking status. For every location, age, sex, and cause, we compute the exposure-weighted relative risk of mortality in

2022 (the last year for which we have GBD estimates) among current smokers and former smokers. For instance, to estimate the relative risk of mortality from ischemic heart disease among all current smokers for a given location, age, and sex, we weighted the GBD relative risk curve for ischemic heart disease by the distribution of cigarette equivalents consumed per day in that location, age, and sex. We then aggregate these cause-specific relative risks to obtain the all-cause relative risk of mortality by smoking status, weighting by the cause-specific mortality rate by region, age, and sex in 2022 (Eq. 3).

$$\log(RR_z) = \sum_i^{\omega} \log(RR_{i,z}) * \frac{Mortality_{i,l,a,s}}{\sum_j^{y} Mortality_{j,l,a,s}} \tag{3}$$

Where Mortality is mortality rate, RR is the exposure-weighted relative risk of mortality, l is location, a is age group, s is sex, c is cause, z is smoking status group, w is the set of causes associated with smoking, and y is the set of causes in the GBD.

Finally, we computed the mortality rate among never smokers and used each of the all-cause mortality rates to adjust our prevalence estimates in every future year as shown in Eq. 4:

$$\sum_j^{y} Mortality_{j,l,a,s} = P_{N_{l,a,s}} * x + P_{F_{l,a,s}} * RR_{former_{l,a,s}} * x + P_{C_{l,a,s}} * RR_{current_{l,a,s}} * x \tag{4}$$

Where x is the mortality rate among never smokers, P_N, P_F, P_C are prevalence of never, former, and current smoking, RR_{former} is the exposure-weighted relative risk of mortality among former smokers, and $RR_{current}$ is the exposure-weighted relative risk of mortality among current smokers.

For every future year, we adjusted the prevalence of each smoking group to account for mortality in the previous year:

$$P_{N_{t+1}} = P_{N_t} - \left(P_{N_t} * x\right) \tag{5}$$

$$P_{F_{t+1}} = P_{F_t} - (P_{F_t} * x * RR_{former}) \tag{6}$$

$$P_{C_{t+1}} = P_{C_t} - (P_{C_t} * x * RR_{current}) \tag{7}$$

Finally, we rescale the prevalence of each smoking group to ensure they summed to 100%:

$$overall.prevalence = P_{C_{t+1}} + P_{F_{t+1}} + P_{N_{t+1}} \tag{8}$$

$$P_{C_{t+1}} = P_{C_{t+1}}/overall.prevalence \tag{9}$$

$$P_{F_{t+1}} = P_{F_{t+1}}/overall.prevalence \tag{10}$$

$$P_{N_{t+1}} = P_{N_{t+1}}/overall.prevalence \tag{11}$$

Risk Reduction Curves

Throughout this analysis, we use the dose-response relative risk (RR) curves from GBD 2021. For all non-cardiovascular disease (CVD) causes, one RR curve was used for all age groups. For

CVD causes, the GBD adjusted the RR curves to account for lowering relative risk of CVD outcomes with age.

Similarly, to generate burden estimates among former smokers, we used GBD 2021 dose-response risk reduction curves. The shape of these curves is generated using literature reporting the rate of harm reduction among former smokers. To ensure consistency between current and former smoker burden, the risk reduction curves are anchored to the corresponding relative risk curves.[4] For any given cause, the relative risk among former smokers at 0 years since quitting is equivalent to the age-specific exposure-weighted relative risk among current smokers. For instance, if the smoking exposure of 50-54 year old current smokers for a given location, year, and sex smoke follows a symmetrical distribution, with this group smoking a mean of 2 cigarette-equivalents per day, and the relative risk of dying of a given cause is 1.8 at 2 cigarette-equivalents per day, the relative risk of mortality of that cause among 50-54 year old former smokers with 0 years since quitting will also be 1.8, with relative risk declining as years since quitting increase.

When forecasting burden among former smokers, we used the exposure distribution for the age at which they quit to determine the anchor point. For CVD outcomes, we applied the exposure distribution to the relative risk curve for their current age to determine this anchor point. For example, for those who quit at age 50-54 observed at age 75-79, we used the distribution of cigarette equivalents consumed per day at age 50 to 54 and the IHD RR curve for those at age 75 to 79 to determine the anchor for the risk reduction curve. Once the risk reduction curve was anchored, we used the value at 25 years since quitting to compute their burden. We do this because using the IHD RR curve for those at age 50-54 in this instance would result in overestimating the relative risk of IHD.

Illustrative Cohort

Taking an illustrative cohort of individuals who were 40-44 years old in 2022, we first compute the current and former smoking prevalence without differential mortality under the Elimination - 2050 scenario. The current smoking prevalence in this cohort is 40% in 2022; under this scenario we set the smoking prevalence to decline linearly to 5% between 2023 and 2050. In this scenario, we assume that there are no new smokers, so we hold the prevalence of never smokers constant.

Using these prevalence values and the 2022 GBD exposure distributions, we then compute the cause-specific exposure-weighted relative risk of mortality in 2022. We then adjust the current, former, and never smoking prevalence to account for differential mortality.

Using these updated prevalence rates, as well as exposure distributions and relative risk (RR) curves from GBD 2021, we then compute the PAFs for every cause using Eq. 1.

GATHER Checklist

This study complies with the Guidelines for Accurate and Transparent Health Estimates Reporting (GATHER) recommendations. We have documented the steps involved in our analytical procedures and detailed the data sources in the GATHER checklist below. The GATHER recommendations can be found here: http://gather-statement.org/

Appendix Table S2. GATHER compliance

#	GATHER checklist item	Description of compliance	Reference
	Objectives and funding		
1	Define the indicator(s), populations (including age, sex, and geographic entities), and time period(s) for which estimates were made.	Narrative provided in the paper and appendix describing indicators, definitions, and populations	Main text (Methods - Overview) and appendix
2	List the funding sources for the work.	Funding sources listed in paper	Main text (Summary – Funding)
	Data Inputs		
	For all data inputs from multiple sources that are synthesized as part of the study:		
3	Describe how the data were identified and how the data were accessed.	Narrative provided in paper and appendix describing data seeking methods	Main text (Methods) and appendix
4	Specify the inclusion and exclusion criteria. Identify all ad-hoc exclusions.	Narrative provided in paper and appendix describing inclusion and exclusion criteria by data type	Main text (Methods) and appendix
5	Provide information on all included data sources and their main characteristics. For each data source used, report reference information or contact name/institution, population represented, data collection method, year(s) of data collection, sex and age range, diagnostic criteria or measurement method, and sample size, as relevant.	Narrative for data sources is provided in paper and appendix. Metadata for sources by geography are available through an online data source tool; information on metadata for UNPD data available in the appendix	Main text (Methods), appendix, and through the online data citation tool: https://ghdx.healthdata.org/gbd-2021/results
6	Identify and describe any categories of input data that have potentially important biases (e.g., based on characteristics listed in item 5).	Limitations of and biases in data included in paper	Main text (Discussion – Limitations)
	For data inputs that contribute to the analysis but were not synthesized as part of the study:		
7	Describe and give sources for any other data inputs.	Included in online data source tools	Online data citation tool: https://ghdx.healthdata.org/gbd-2021/results
	For all data inputs:		
8	Provide all data inputs in a file format from which data can be efficiently extracted (e.g., a spreadsheet rather than a PDF), including all relevant meta-data listed in item 5. For any data inputs that cannot be shared because of ethical or legal reasons, such as third-party ownership, provide a contact name or the name of the institution that retains the right to the data.	Downloads of input data are available through online data query tools: https://ghdx.healthdata.org/gbd-2021/results	Global Health Data Exchange: https://ghdx.healthdata.org/gbd-2021/results
	Data analysis		
9	Provide a conceptual overview of the data analysis method. A diagram may be helpful.	A brief overview of the overall methodological processes have been provided	Main text (Methods) and appendix
10	Provide a detailed description of all steps of the analysis, including mathematical formulae. This	Detailed descriptions of all steps of the analysis, as well as relevant	Main text (Methods) and appendix

	description should cover, as relevant, data cleaning, data pre-processing, data adjustments and weighting of data sources, and mathematical or statistical model(s).	mathematical formulae, have been provided	
**11**	Describe how candidate models were evaluated and how the final model(s) were selected.	Details on model evaluation and finalisation have been provided	Main text (Methods – Reference Scenario Forecast) and appendix
**12**	Provide the results of an evaluation of model performance, if done, as well as the results of any relevant sensitivity analysis.	Details on evaluation of model performance have been provided	Main text (Results – Reference Scenario Forecast) and appendix
**13**	Describe methods for calculating uncertainty of the estimates. State which sources of uncertainty were, and were not, accounted for in the uncertainty analysis.	Details on uncertainty calculations have been provided	Main text (Methods) and appendix
**14**	State how analytic or statistical source code used to generate estimates can be accessed.	Access statement provided	Code is provided in an online repository: https://github.com/ihmeuw/fhs_2021_smoking_paper
	Results and Discussion		
**15**	Provide published estimates in a file format from which data can be efficiently extracted.	Results are available for download through online data tool.	Online data tool: https://ghdx.healthdata.org/gbd-2021/results
**16**	Report a quantitative measure of the uncertainty of the estimates (e.g. uncertainty intervals).	Uncertainty intervals are provided with results	Main text (Results and Discussion) and online data tools: https://ghdx.healthdata.org/gbd-2021/results https://vizhub.healthdata.org/tobacco-forecasting/
**17**	Interpret results in light of existing evidence. If updating a previous set of estimates, describe the reasons for changes in estimates.	Discussion of methodological differences between this study and existing evidence	Main text (Research in Context, Introduction, Methods, Discussion) and appendix
**18**	Discuss limitations of the estimates. Include a discussion of any modelling assumptions or data limitations that affect interpretation of the estimates.	Discussion of limitations was provided	Main text (Discussion – Limitations)

References

1 Zheng P, Barber R, Sorensen RJD, Murray CJL, Aravkin AY. Trimmed Constrained Mixed Effects Models: Formulations and Algorithms. *Journal of Computational and Graphical Statistics* 2021; **30**: 544–56.

2 Foreman KJ, Marquez N, Dolgert A, *et al.* Forecasting life expectancy, years of life lost, and all-cause and cause-specific mortality for 250 causes of death: reference and alternative scenarios for 2016–40 for 195 countries and territories. *Lancet* 2018; **392**: 2052–90.

3 GBD 2021 Forecasting Collaborators. Burden of disease scenarios for 204 countries and territories, 2022–2050: a forecasting analysis for the Global Burden of Disease Study 2021. *The Lancet* 2024; published online May 18.

4 GBD 2021 Risk Factor Collaborators. Global burden and strength of evidence for 88 risk factors in 204 countries and 811 subnational locations, 1990-2020: a systematic analysis for the Global Burden of Disease Study 2021. *The Lancet* 2024; published online May 16.
