# Supplementary material for: Forecasting the effects of smoking prevalence scenarios on years of life lost and life expectancy from 2022 to 2050: a systematic analysis for the Global Burden of Disease Study 2021
Source: Lancet Public Health. 2024 Oct 2;9(10):e729–44. doi: 10.1016/S2468-2667(24)00166-X (PMC11447278; doi:10.1016/S2468-2667(24)00166-X)

THE LANCET
Public Health

Supplementary appendix 2

This appendix formed part of the original submission and has been peer reviewed. We post it as supplied by the authors.

Supplement to: GBD 2021 Tobacco Forecasting Collaborators. Forecasting the effects of smoking prevalence scenarios on years of life lost and life expectancy from 2022 to 2050: a systematic analysis for the Global Burden of Disease Study 2021. *Lancet Public Health* 2024; **9:** e729–44.

Appendix 2: supplementary results appendix to "Forecasting the impacts of smoking prevalence scenarios on years of life lost and life expectancy from 2022 to 2050 - a systematic analysis for the Global Burden of Disease Study 2021"

This supplement provides additional figures and tables containing more detailed results for "Forecasting the impacts of smoking prevalence scenarios on years of life lost and life expectancy from 2022 to 2050 - a systematic analysis for the Global Burden of Disease Study 2021".

Table of Contents:

Supplementary Results Table S1. Global all-age and age-standardised prevalence of current and former smokers by location, sex, and scenario, 2022 to 2050 ………………………..………. 3

Supplementary Results Table S2. Cumulative all-age counts and age-standardised rates of years of life lost (YLLs) between 2022 and 2050 by location, sex, and scenario ……………….…. 10

Supplementary Results Table S3. Life expectancy at birth by location, sex, and scenario ……. 17

Supplementary Results Figure S1. Years of Life Lost (YLLs) by scenario, all causes ………... 27

Supplementary Results Figure S2. Years of Life Lost (YLLs) by scenario, IHD ……………... 28

Supplementary Results Figure S3. Years of Life Lost (YLLs) by scenario, lung cancer …...…. 29

Supplementary Results Figure S4. Years of Life Lost (YLLs) by scenario, COPD …………... 30

Supplementary Results Figure S5. Life expectancy at birth by super-region, sex, and scenario…………………………………………………………………………………..…31

Supplementary Results Figure S6. Global YLLs under Reference scenario…………………... 32

Supplementary Results Figure S7. Difference in cumulative age-standardised rate of YLLs under Elimination-2023 scenario compared to Reference scenario, 2022 to 2050 (per 100,000), Males……………………………………………………………………………..……... 33

Supplementary Results Figure S8. Difference in cumulative age-standardised rate of YLLs under Elimination-2023 scenario compared to Reference scenario, 2022 to 2050 (per 100,000), Females……………………………………………………………………..…...…….34

Supplementary Results Table S1. Global all-age and age-standardised prevalence of current and former smokers by location, sex, and scenario, 2022 and 2050

Location	Sex	Current Smoking Prevalence 2022	Current Smoking Prevalence 2050	Former Smoking Prevalence 2022	Former Smoking Prevalence 2050	Current Smokers (millions) 2022	Current Smokers (millions) 2050	Former Smokers (millions) 2022	Former Smokers (millions) 2050
Global	Female	5.96% (5.76% - 6.21%)	4.18% (3.98% - 4.48%)	5.81% (5.6% - 6.05%)	5.5% (5.28% - 5.72%)	241 (229 - 252)	201 (187 - 220)	237 (225 - 249)	280 (264 - 299)
Global	Male	28.4% (27.9% - 29.1%)	21.1% (20.6% - 21.6%)	14.3% (13.9% - 14.6%)	14.2% (13.9% - 14.6%)	1160 (1120 - 1200)	1040 (983 - 1110)	569 (548 - 593)	754 (711 - 801)
Central Europe, Eastern Europe, and Central Asia	Female	13.9% (13.3% - 14.5%)	11.3% (9.91% - 13.1%)	8.59% (8.13% - 9.08%)	8.68% (8.17% - 9.32%)	28.2 (26.3 - 30.4)	19.9 (17.3 - 23.7)	17.7 (16.4 - 19.1)	15.8 (14.4 - 17.4)
Central Europe, Eastern Europe, and Central Asia	Male	34.4% (33.7% - 35.2%)	26% (24.1% - 29.3%)	19.4% (19% - 19.9%)	19.5% (18.9% - 20.1%)	71.7 (67.3 - 76.1)	50.4 (45.4 - 58.8)	40.8 (38.4 - 43.1)	44.1 (41 - 47.6)
Central Asia	Female	3.53% (3.21% - 3.86%)	3.34% (2.82% - 3.95%)	2.5% (2.27% - 2.73%)	2.72% (2.47% - 2.98%)	1.78 (1.52 - 2.02)	1.95 (1.57 - 2.37)	1.24 (1.08 - 1.42)	1.59 (1.36 - 1.81)
Central Asia	Male	29.8% (28.7% - 30.9%)	26.3% (24.2% - 28.8%)	12.8% (12.3% - 13.3%)	13.1% (12.6% - 13.6%)	14.7 (13.2 - 16.4)	16.3 (14 - 19.2)	5.73 (5.11 - 6.31)	8.21 (7.21 - 9.17)
Armenia	Female	2.97% (2.38% - 3.63%)	2.89% (2.28% - 3.58%)	1.27% (1.06% - 1.52%)	1.23% (1.03% - 1.49%)	0.0472 (0.0364 - 0.0601)	0.0308 (0.0218 - 0.0413)	0.0207 (0.0167 - 0.0254)	0.0176 (0.0131 - 0.0226)
Armenia	Male	48.9% (47.6% - 50.3%)	45.4% (43.8% - 47.3%)	13.9% (12.8% - 15%)	13.6% (12.6% - 14.7%)	0.736 (0.639 - 0.839)	0.538 (0.428 - 0.649)	0.212 (0.18 - 0.244)	0.227 (0.179 - 0.282)
Azerbaijan	Female	2.31% (1.75% - 2.98%)	2.17% (1.53% - 3.1%)	1.06% (0.85% - 1.29%)	1.05% (0.84% - 1.3%)	0.126 (0.0932 - 0.167)	0.107 (0.0726 - 0.152)	0.0555 (0.0416 - 0.0699)	0.0477 (0.0361 - 0.0608)
Azerbaijan	Male	39.6% (38.2% - 41.2%)	38.4% (35.8% - 41.6%)	12.4% (11.6% - 13.3%)	12.6% (11.7% - 13.5%)	2.21 (1.89 - 2.54)	2.35 (1.95 - 2.76)	0.625 (0.524 - 0.726)	0.811 (0.67 - 0.945)
Georgia	Female	7.4% (6.39% - 8.61%)	8.66% (7.4% - 10.2%)	1.94% (1.62% - 2.34%)	1.76% (1.48% - 2.14%)	0.126 (0.106 - 0.152)	0.123 (0.0984 - 0.15)	0.0344 (0.0283 - 0.0415)	0.0277 (0.022 - 0.0338)
Georgia	Male	47% (45.3% - 48.7%)	46.4% (43.6% - 49.1%)	21.3% (20% - 22.9%)	21.9% (20.6% - 23.5%)	0.818 (0.713 - 0.913)	0.717 (0.593 - 0.834)	0.405 (0.354 - 0.459)	0.428 (0.353 - 0.503)
Kazakhstan	Female	6.52% (5.52% - 7.68%)	5.09% (3.75% - 6.9%)	3.54% (2.97% - 4.17%)	3.57% (2.97% - 4.26%)	0.646 (0.533 - 0.78)	0.553 (0.398 - 0.773)	0.352 (0.291 - 0.425)	0.4 (0.325 - 0.49)
Kazakhstan	Male	35.8% (34.2% - 37.6%)	27.9% (24.8% - 32%)	7.21% (6.63% - 7.87%)	7.74% (7.1% - 8.44%)	3.51 (3.08 - 3.91)	3.19 (2.64 - 3.9)	0.65 (0.569 - 0.734)	0.908 (0.774 - 1.04)
Kyrgyzstan	Female	5.17% (4.21% - 6.23%)	6.35% (5.08% - 7.71%)	2.07% (1.69% - 2.5%)	2.43% (1.91% - 3.04%)	0.188 (0.144 - 0.239)	0.258 (0.195 - 0.33)	0.0756 (0.0591 - 0.0948)	0.0933 (0.0674 - 0.126)
Kyrgyzstan	Male	40.9% (39.1% - 42.7%)	44% (39.7% - 46.8%)	14.7% (13.6% - 15.8%)	17.4% (16% - 18.8%)	1.4 (1.2 - 1.6)	1.83 (1.45 - 2.22)	0.472 (0.394 - 0.55)	0.752 (0.604 - 0.929)
Mongolia	Female	8.16% (7.01% - 9.36%)	10.6% (9.15% - 12.1%)	1.93% (1.63% - 2.27%)	1.78% (1.52% - 2.09%)	0.148 (0.125 - 0.173)	0.301 (0.248 - 0.355)	0.0335 (0.0281 - 0.04)	0.0504 (0.0422 - 0.0605)
Mongolia	Male	47.4% (45.9% - 48.8%)	52% (50.3% - 53.5%)	11.8% (10.8% - 12.7%)	11.7% (10.8% - 12.7%)	0.833 (0.766 - 0.901)	1.42 (1.27 - 1.57)	0.189 (0.168 - 0.212)	0.303 (0.26 - 0.349)
Tajikistan	Female	1% (0.78% - 1.28%)	0.61% (0.47% - 0.77%)	2.66% (2.22% - 3.09%)	3.18% (2.61% - 3.74%)	0.0528 (0.0391 - 0.0691)	0.0465 (0.0342 - 0.0609)	0.14 (0.109 - 0.175)	0.241 (0.185 - 0.31)
Tajikistan	Male	13.2% (12.1% - 14.3%)	7.45% (6.43% - 8.36%)	14.3% (13.2% - 15.6%)	14.9% (13.8% - 16.2%)	0.684 (0.577 - 0.802)	0.586 (0.471 - 0.717)	0.676 (0.569 - 0.8)	1.16 (0.968 - 1.4)
Turkmenistan	Female	2.6% (2.03% - 3.31%)	1.51% (1.09% - 2.18%)	2.39% (2.01% - 2.83%)	2.73% (2.25% - 3.28%)	0.0684 (0.0522 - 0.0896)	0.0471 (0.0331 - 0.07)	0.0621 (0.0508 - 0.0771)	0.0867 (0.0688 - 0.108)
Turkmenistan	Male	17.4% (15.9% - 19.2%)	8.47% (6.83% - 10.8%)	14.4% (13.3% - 15.6%)	15.5% (14.3% - 16.9%)	0.488 (0.422 - 0.562)	0.282 (0.217 - 0.369)	0.363 (0.315 - 0.414)	0.546 (0.457 - 0.65)
Uzbekistan	Female	2.02% (1.59% - 2.58%)	2.21% (1.65% - 3%)	2.71% (2.28% - 3.18%)	2.89% (2.42% - 3.38%)	0.367 (0.244 - 0.513)	0.491 (0.301 - 0.707)	0.483 (0.339 - 0.662)	0.642 (0.432 - 0.884)
Uzbekistan	Male	22.2% (20.6% - 23.9%)	22.9% (19.7% - 26.9%)	13.4% (12.3% - 14.5%)	13.4% (12.3% - 14.5%)	3.92 (2.83 - 5.02)	5.41 (3.7 - 7.38)	2.12 (1.5 - 2.74)	3.07 (2.09 - 4)
Central Europe	Female	23.7% (23% - 24.5%)	22% (21.1% - 23%)	13.1% (12.4% - 13.8%)	14% (13.2% - 15.1%)	12.6 (11.9 - 13.3)	8.88 (8.22 - 9.48)	7.51 (7.01 - 7.99)	6.46 (5.9 - 7.09)
Central Europe	Male	31.9% (31.3% - 32.6%)	25.5% (24.8% - 26.1%)	20% (19.5% - 20.5%)	19.7% (19% - 20.4%)	17.7 (16.7 - 18.5)	11 (10.3 - 11.7)	12.6 (11.9 - 13.2)	11.6 (10.8 - 12.3)
Albania	Female	11.5% (9.61% - 13.7%)	13.5% (10.7% - 16.4%)	2.54% (2.14% - 3.08%)	2.83% (2.36% - 3.47%)	0.154 (0.122 - 0.188)	0.149 (0.109 - 0.193)	0.0345 (0.0281 - 0.0427)	0.0333 (0.0256 - 0.0423)
Albania	Male	48% (46.5% - 49.6%)	55.1% (52.7% - 56.9%)	11.8% (10.8% - 12.6%)	11.7% (10.8% - 12.6%)	0.665 (0.573 - 0.766)	0.672 (0.553 - 0.807)	0.176 (0.15 - 0.206)	0.182 (0.148 - 0.222)
Bosnia and Herzegovina	Female	27.7% (24.5% - 31.4%)	30% (25.1% - 35.5%)	10.5% (9% - 12.2%)	12.4% (10.2% - 14.7%)	0.439 (0.371 - 0.512)	0.266 (0.198 - 0.349)	0.169 (0.138 - 0.202)	0.123 (0.0926 - 0.156)
Bosnia and Herzegovina	Male	40% (37.4% - 42.1%)	38.5% (33% - 44.7%)	16.2% (15.1% - 17.5%)	16.8% (15.7% - 18.1%)	0.66 (0.577 - 0.742)	0.403 (0.306 - 0.521)	0.306 (0.262 - 0.353)	0.301 (0.238 - 0.375)
Bulgaria	Female	30.8% (28.6% - 33.3%)	27.3% (24.9% - 29.8%)	13.4% (11.8% - 15.2%)	16.8% (14.9% - 18.9%)	0.885 (0.772 - 0.991)	0.517 (0.44 - 0.595)	0.416 (0.359 - 0.478)	0.354 (0.301 - 0.407)
Bulgaria	Male	37.4% (35.5% - 39.4%)	28.7% (26.5% - 31.3%)	18.4% (17.3% - 19.6%)	17.9% (16.7% - 19.1%)	1.19 (1.05 - 1.32)	0.609 (0.511 - 0.7)	0.692 (0.614 - 0.778)	0.527 (0.455 - 0.601)
Croatia	Female	29.6% (27.3% - 32%)	28.7% (25.9% - 31.3%)	13.3% (11.8% - 15%)	14.8% (13% - 16.6%)	0.576 (0.491 - 0.661)	0.35 (0.29 - 0.417)	0.29 (0.24 - 0.341)	0.219 (0.176 - 0.262)
Croatia	Male	34.5% (32.6% - 36.2%)	29.7% (27.7% - 31.7%)	19.5% (18.3% - 20.6%)	19.2% (18% - 20.4%)	0.676 (0.582 - 0.775)	0.376 (0.306 - 0.436)	0.477 (0.405 - 0.549)	0.4 (0.326 - 0.468)
Czechia	Female	21.6% (19.9% - 23.5%)	17.8% (16% - 19.9%)	15.3% (13.6% - 17.2%)	14.8% (13% - 16.7%)	1.06 (0.933 - 1.2)	0.727 (0.632 - 0.833)	0.832 (0.72 - 0.959)	0.705 (0.607 - 0.82)
Czechia	Male	31% (29.6% - 32.7%)	25.8% (24.3% - 27.3%)	21.4% (20.1% - 22.9%)	20.7% (19.4% - 22.1%)	1.56 (1.4 - 1.72)	1.08 (0.958 - 1.2)	1.3 (1.15 - 1.46)	1.23 (1.07 - 1.39)
Hungary	Female	23.4% (21% - 25.7%)	17.2% (14% - 21.6%)	10.8% (9.63% - 12.2%)	10.6% (9.01% - 12.4%)	1.04 (0.87 - 1.19)	0.686 (0.533 - 0.877)	0.554 (0.466 - 0.65)	0.497 (0.41 - 0.609)
Hungary	Male	30% (28.2% - 31.8%)	21.2% (18.4% - 24.9%)	18.3% (17.1% - 19.6%)	17.1% (16% - 18.4%)	1.35 (1.15 - 1.56)	0.893 (0.736 - 1.07)	0.961 (0.826 - 1.1)	0.941 (0.812 - 1.09)
Montenegro	Female	29.4% (26.9% - 31.8%)	25.6% (21.8% - 30.3%)	13.3% (11.3% - 15.6%)	13.5% (11.4% - 15.8%)	0.0942 (0.0796 - 0.11)	0.063 (0.0496 - 0.0796)	0.0419 (0.0339 - 0.0511)	0.0332 (0.0268 - 0.0407)
Montenegro	Male	32.7% (30.9% - 34.4%)	26.1% (23.1% - 30.9%)	19.8% (18.4% - 21.2%)	19.8% (18.4% - 21.2%)	0.106 (0.0921 - 0.121)	0.0705 (0.0565 - 0.0878)	0.0659 (0.0564 - 0.077)	0.0628 (0.0528 - 0.0748)
North Macedonia	Female	28.1% (25.6% - 30.4%)	27.8% (25.3% - 30.2%)	9.73% (8.16% - 11.5%)	10.4% (8.72% - 12.2%)	0.308 (0.249 - 0.373)	0.224 (0.178 - 0.273)	0.104 (0.0813 - 0.136)	0.0833 (0.0635 - 0.108)
North Macedonia	Male	42.3% (40.4% - 44.1%)	42.3% (40.5% - 44.2%)	15.7% (14.6% - 17%)	15.7% (14.6% - 17%)	0.502 (0.411 - 0.596)	0.384 (0.311 - 0.466)	0.187 (0.15 - 0.226)	0.173 (0.136 - 0.212)
Poland	Female	22.2% (20.5% - 23.9%)	19.4% (17.9% - 21%)	14.5% (12.9% - 16.2%)	14.3% (11.7% - 17.4%)	4.08 (3.59 - 4.57)	2.72 (2.39 - 3.06)	2.9 (2.48 - 3.3)	2.4 (1.91 - 2.96)
Poland	Male	28.2% (26.8% - 29.6%)	19.6% (16.1% - 22.1%)	21.5% (20.1% - 22.9%)	21% (19.2% - 22.7%)	5.35 (4.72 - 5.95)	2.89 (2.29 - 3.41)	4.41 (3.9 - 4.92)	4.16 (3.65 - 4.68)
Romania	Female	22.4% (20.7% - 24.1%)	23.8% (22% - 25.6%)	9.78% (8.63% - 11%)	12.2% (10.7% - 13.8%)	1.91 (1.63 - 2.22)	1.44 (1.21 - 1.68)	0.89 (0.737 - 1.05)	0.79 (0.638 - 0.961)
Romania	Male	34.7% (33.2% - 36.2%)	28.9% (27.4% - 30.4%)	19.4% (18.3% - 20.7%)	19.7% (18.6% - 21%)	3.1 (2.67 - 3.57)	1.84 (1.54 - 2.15)	2.06 (1.77 - 2.4)	1.78 (1.49 - 2.08)
Serbia	Female	33.4% (30.6% - 36.6%)	38.5% (35.2% - 41.8%)	16.5% (14.4% - 19.2%)	20.7% (16.9% - 24.6%)	1.41 (1.19 - 1.66)	1.23 (1.04 - 1.44)	0.704 (0.582 - 0.836)	0.681 (0.542 - 0.821)
Serbia	Male	33.7% (31.8% - 35.5%)	30% (26.7% - 34.5%)	19.3% (17.9% - 20.8%)	19% (17.6% - 20.6%)	1.5 (1.29 - 1.73)	1.06 (0.873 - 1.31)	0.973 (0.825 - 1.14)	0.826 (0.693 - 0.963)
Slovakia	Female	19.6% (17.7% - 21.8%)	18% (16.1% - 20%)	14.3% (12.8% - 16%)	14.1% (12.3% - 15.9%)	0.482 (0.418 - 0.554)	0.355 (0.308 - 0.408)	0.388 (0.335 - 0.455)	0.321 (0.274 - 0.375)
Slovakia	Male	30.2% (28.3% - 32%)	25% (22.8% - 26.9%)	22.6% (21.1% - 24.1%)	22.9% (21.3% - 24.5%)	0.804 (0.711 - 0.9)	0.57 (0.497 - 0.64)	0.658 (0.588 - 0.738)	0.664 (0.585 - 0.751)
Slovenia	Female	22% (20% - 24.2%)	20.7% (17.5% - 23.5%)	17.7% (15.6% - 19.7%)	19.5% (17.1% - 22%)	0.203 (0.178 - 0.232)	0.177 (0.147 - 0.209)	0.185 (0.16 - 0.215)	0.198 (0.17 - 0.229)
Slovenia	Male	25.7% (24.1% - 27.2%)	22.5% (20% - 24.6%)	23.3% (21.8% - 24.9%)	22.2% (20.8% - 23.8%)	0.246 (0.22 - 0.273)	0.2 (0.17 - 0.226)	0.287 (0.257 - 0.32)	0.294 (0.266 - 0.328)
Eastern Europe	Female	14% (12.8% - 15.2%)	11.8% (9.32% - 15.4%)	9.02% (8.27% - 9.91%)	9.9% (8.97% - 10.9%)	13.4 (11.7 - 15.3)	8.65 (6.68 - 11.6)	8.42 (7.35 - 9.57)	7.19 (6.23 - 8.18)
Eastern Europe	Male	38.8% (37.6% - 40%)	26.8% (23.6% - 32.8%)	21.5% (20.6% - 22.4%)	22.9% (21.7% - 23.9%)	39 (34.9 - 43.3)	23.1 (19.4 - 29.3)	21.7 (19.5 - 24.1)	23.2 (20.7 - 26)
Belarus	Female	13.6% (12.1% - 15.6%)	10.9% (9.41% - 12.8%)	6.7% (5.79% - 7.87%)	5.75% (4.87% - 6.89%)	0.568 (0.462 - 0.699)	0.348 (0.285 - 0.431)	0.301 (0.247 - 0.366)	0.209 (0.168 - 0.258)
Belarus	Male	42.7% (40.9% - 44.4%)	38.2% (36.3% - 39.9%)	22.8% (21.2% - 24.5%)	23.8% (22.1% - 25.5%)	1.94 (1.69 - 2.22)	1.5 (1.28 - 1.74)	1.05 (0.891 - 1.22)	1.05 (0.893 - 1.24)
Estonia	Female	16.7% (15.2% - 18.1%)	11.6% (8.85% - 15.7%)	18.1% (16.2% - 20.2%)	21.1% (18.9% - 23.5%)	0.104 (0.0899 - 0.118)	0.0622 (0.0453 - 0.0851)	0.119 (0.105 - 0.136)	0.123 (0.108 - 0.142)
Estonia	Male	27.2% (25.8% - 28.7%)	16.1% (12.8% - 21.3%)	27.7% (26.2% - 29.3%)	30.5% (28.9% - 32.2%)	0.171 (0.152 - 0.189)	0.0904 (0.0686 - 0.122)	0.188 (0.168 - 0.21)	0.211 (0.187 - 0.235)
Latvia	Female	20.1% (18.2% - 22.1%)	18.9% (16% - 21.6%)	14.4% (13% - 16%)	18.2% (16.3% - 20.3%)	0.164 (0.144 - 0.187)	0.0917 (0.0732 - 0.112)	0.121 (0.105 - 0.138)	0.0975 (0.0802 - 0.115)
Latvia	Male	41.6% (39.9% - 43.4%)	36.2% (34.1% - 38.4%)	21.4% (20.1% - 22.8%)	21.9% (20.5% - 23.4%)	0.355 (0.32 - 0.392)	0.2 (0.17 - 0.232)	0.206 (0.185 - 0.231)	0.192 (0.162 - 0.225)
Lithuania	Female	20.1% (18.1% - 22.1%)	22.4% (19.3% - 25.3%)	11.3% (10% - 12.7%)	13.7% (11.8% - 15.6%)	0.232 (0.2 - 0.265)	0.137 (0.11 - 0.165)	0.152 (0.13 - 0.177)	0.117 (0.0946 - 0.144)
Lithuania	Male	34.7% (32.7% - 36.5%)	28.9% (26.7% - 30.9%)	20.8% (19.4% - 22.1%)	22% (20.7% - 23.4%)	0.434 (0.388 - 0.483)	0.228 (0.192 - 0.27)	0.287 (0.254 - 0.318)	0.267 (0.224 - 0.309)
Republic of Moldova	Female	6.97% (5.82% - 8.22%)	6.95% (5.67% - 8.37%)	3.32% (2.84% - 3.82%)	3.51% (3.02% - 4.05%)	0.112 (0.0863 - 0.137)	0.0331 (0.022 - 0.0473)	0.065 (0.0493 - 0.0808)	0.048 (0.0331 - 0.0633)
Republic of Moldova	Male	37.2% (35% - 39.4%)	34.7% (31.4% - 39.2%)	29.7% (28% - 31.6%)	30.8% (29% - 32.6%)	0.644 (0.531 - 0.756)	0.248 (0.174 - 0.33)	0.511 (0.411 - 0.605)	0.409 (0.285 - 0.528)
Russian Federation	Female	14.2% (12.7% - 15.8%)	11.9% (9.06% - 16.1%)	9.01% (7.96% - 10.2%)	9.98% (8.78% - 11.3%)	9.71 (8.14 - 11.3)	6.74 (4.95 - 9.55)	5.92 (4.96 - 7.02)	5.49 (4.61 - 6.46)
Russian Federation	Male	39.5% (38% - 41%)	27.1% (23.4% - 33.8%)	20.6% (19.3% - 21.8%)	22.2% (20.7% - 23.6%)	28.1 (24.4 - 32.3)	17.7 (14.3 - 23.2)	14.6 (12.6 - 16.7)	16.8 (14.6 - 19.2)
Ukraine	Female	13.4% (11.9% - 15.2%)	11.2% (8.94% - 14%)	9.43% (8.24% - 10.6%)	9.96% (8.77% - 11.3%)	2.48 (2 - 3.08)	1.22 (0.889 - 1.65)	1.74 (1.37 - 2.17)	1.11 (0.838 - 1.38)
Ukraine	Male	36.2% (34.6% - 37.9%)	22.3% (19.5% - 27.2%)	23.5% (22.2% - 24.8%)	25.2% (23.8% - 26.6%)	7.36 (5.87 - 8.81)	3.14 (2.28 - 4.2)	4.91 (3.94 - 5.96)	4.32 (3.33 - 5.38)
High-income	Female	15.6% (15.1% - 16%)	11% (10.5% - 11.7%)	16.7% (16.2% - 17.3%)	16.6% (16% - 17.3%)	81.1 (77.9 - 84.5)	55.7 (52.7 - 59.2)	99.8 (96.1 - 104)	103 (98.2 - 108)
High-income	Male	23% (22.5% - 23.5%)	14.9% (14.4% - 15.7%)	23.8% (23.4% - 24.3%)	23.6% (23.1% - 24.1%)	121 (117 - 125)	78.1 (74.6 - 82.4)	148 (143 - 153)	161 (155 - 168)
Australasia	Female	12.6% (11% - 14.2%)	7.2% (6.15% - 8.3%)	22.4% (20.5% - 24.4%)	21% (19.4% - 23.4%)	1.92 (1.66 - 2.18)	1.42 (1.22 - 1.63)	3.63 (3.3 - 4.04)	4.6 (4.12 - 5.15)
Australasia	Male	14.2% (13.1% - 15.3%)	7.69% (7.07% - 8.36%)	26.2% (24.8% - 27.6%)	25.4% (23.9% - 26.8%)	2.11 (1.93 - 2.32)	1.51 (1.38 - 1.66)	4.27 (3.93 - 4.62)	5.66 (5.2 - 6.14)
Australia	Female	12.2% (10.3% - 14.1%)	7.05% (5.76% - 8.3%)	21.7% (19.6% - 24%)	20.3% (18.3% - 22.9%)	1.56 (1.32 - 1.82)	1.17 (0.974 - 1.38)	2.9 (2.57 - 3.3)	3.65 (3.2 - 4.18)

Supplementary Results Table S1. Global all-age and age-standardised prevalence of current and former smokers by location, sex, and scenario, 2022 and 2050

Location	Sex	Current Smoking Prevalence 2022	Current Smoking Prevalence 2050	Former Smoking Prevalence 2022	Former Smoking Prevalence 2050	Current Smokers (millions) 2022	Current Smokers (millions) 2050	Former Smokers (millions) 2022	Former Smokers (millions) 2050
Australia	Male	13.4% (12.2% - 14.7%)	6.71% (6.02% - 7.46%)	25.4% (23.8% - 27%)	24.5% (22.8% - 26%)	1.67 (1.5 - 1.88)	1.11 (0.995 - 1.25)	3.4 (3.07 - 3.72)	4.48 (4.08 - 4.89)
New Zealand	Female	14.4% (13.3% - 15.8%)	8% (7.16% - 9.2%)	25.8% (24.3% - 27.5%)	25.2% (23.3% - 27.3%)	0.358 (0.317 - 0.401)	0.256 (0.222 - 0.298)	0.736 (0.659 - 0.813)	0.953 (0.848 - 1.05)
New Zealand	Male	18.2% (17.3% - 19.2%)	12.8% (12.1% - 13.6%)	30.4% (29.1% - 31.6%)	31% (28.9% - 32.8%)	0.446 (0.402 - 0.49)	0.412 (0.376 - 0.453)	0.893 (0.804 - 0.972)	1.2 (1.08 - 1.33)
High-income Asia Pacific	Female	7.45% (6.54% - 8.46%)	5.03% (4.26% - 6.03%)	6.48% (5.7% - 7.28%)	7.63% (6.73% - 8.54%)	6.36 (5.53 - 7.2)	3.55 (3 - 4.28)	6.8 (5.94 - 7.72)	7.44 (6.53 - 8.57)
High-income Asia Pacific	Male	29.9% (28.6% - 31.1%)	18.2% (17.1% - 19.3%)	16.5% (15.6% - 17.5%)	17.6% (16.5% - 18.6%)	27.2 (25.2 - 29.1)	13.6 (12.4 - 14.7)	18.8 (17.3 - 20.3)	19.9 (18.2 - 21.5)
Brunei Darussalam	Female	4.66% (3.6% - 5.88%)	4.05% (2.9% - 5.24%)	6.16% (5.22% - 7.11%)	7.04% (6.03% - 8.16%)	0.0102 (0.0075 - 0.0135)	0.0094 (0.0065 - 0.013)	0.0127 (0.0102 - 0.0154)	0.0173 (0.0137 - 0.0214)
Brunei Darussalam	Male	24.3% (21.9% - 26.4%)	20.3% (15.1% - 23.4%)	17.3% (16.1% - 18.6%)	18% (16.8% - 19.4%)	0.063 (0.0539 - 0.0741)	0.0469 (0.0333 - 0.0586)	0.0372 (0.0313 - 0.0433)	0.0518 (0.0426 - 0.0613)
Japan	Female	8.86% (7.5% - 10.4%)	5.77% (4.62% - 7.3%)	7.09% (6.04% - 8.24%)	8.32% (7.04% - 9.67%)	5.04 (4.22 - 5.88)	2.6 (2.07 - 3.33)	5.14 (4.29 - 6.02)	5.23 (4.36 - 6.22)
Japan	Male	27.6% (25.9% - 29.4%)	15.2% (13.7% - 16.6%)	16.8% (15.6% - 18.1%)	18.1% (16.8% - 19.6%)	16.7 (15.1 - 18.3)	7.31 (6.48 - 8.24)	13.7 (12.2 - 15)	13.5 (11.8 - 15)
Republic of Korea	Female	4.98% (3.98% - 6.07%)	3.69% (2.94% - 4.66%)	5.57% (4.73% - 6.48%)	6.78% (5.75% - 7.92%)	1.16 (0.929 - 1.4)	0.815 (0.629 - 1.05)	1.56 (1.31 - 1.83)	2.06 (1.69 - 2.53)
Republic of Korea	Male	35.8% (33.9% - 38.1%)	25.8% (24% - 28%)	16.3% (15.1% - 17.5%)	17.2% (15.8% - 18.6%)	9.72 (8.84 - 10.6)	5.57 (4.96 - 6.15)	4.8 (4.31 - 5.27)	5.83 (5.25 - 6.38)
Singapore	Female	6.01% (4.84% - 7.34%)	4.1% (3.27% - 5.14%)	3.52% (2.98% - 4.12%)	3.74% (3.18% - 4.39%)	0.169 (0.133 - 0.21)	0.136 (0.108 - 0.169)	0.0981 (0.0829 - 0.115)	0.134 (0.111 - 0.16)
Singapore	Male	18.2% (16.6% - 20%)	13.4% (12.1% - 14.8%)	11.7% (10.8% - 12.7%)	12.3% (11.3% - 13.4%)	0.58 (0.511 - 0.653)	0.498 (0.438 - 0.56)	0.373 (0.332 - 0.422)	0.55 (0.489 - 0.618)
High-income North America	Female	12.9% (12% - 14%)	7.48% (6.72% - 8.58%)	19.2% (18% - 20.5%)	18.2% (17.1% - 19.4%)	24 (21.7 - 26.6)	14.5 (12.8 - 16.8)	41.4 (38.2 - 45.1)	43.6 (40.2 - 47.6)
High-income North America	Male	16.6% (15.7% - 17.3%)	10.1% (9.23% - 11.5%)	26% (24.8% - 27.1%)	24.9% (23.7% - 26%)	29.8 (27.5 - 32.1)	19.3 (17.2 - 22.2)	53.7 (49.7 - 57.7)	58.6 (54.5 - 63)
Canada	Female	13.4% (11.2% - 16%)	7.5% (5.99% - 9.31%)	21.4% (18.9% - 24%)	20.9% (18.4% - 23.4%)	2.51 (2.1 - 2.99)	1.76 (1.42 - 2.22)	4.58 (3.98 - 5.16)	5.7 (5 - 6.47)
Canada	Male	14.9% (13.4% - 16.3%)	7.59% (6.76% - 8.51%)	25.4% (23.9% - 27.1%)	25.8% (24.2% - 27.4%)	2.74 (2.45 - 3.05)	1.79 (1.57 - 2.05)	5.61 (5.11 - 6.14)	7.31 (6.65 - 8)
Greenland	Female	38.7% (34.2% - 44.3%)	34.5% (29.9% - 40.7%)	28.5% (25.3% - 31.8%)	28.7% (25% - 33.1%)	0.0103 (0.0086 - 0.0121)	0.0093 (0.0077 - 0.0112)	0.0079 (0.0068 - 0.0091)	0.0088 (0.0075 - 0.0104)
Greenland	Male	39.6% (36.8% - 42.4%)	34.4% (31.3% - 37.3%)	24.7% (23% - 26.4%)	24.9% (23.2% - 26.6%)	0.0117 (0.0105 - 0.0131)	0.0098 (0.0085 - 0.0111)	0.0079 (0.007 - 0.0089)	0.0088 (0.0076 - 0.01)
United States of America	Female	12.9% (11.8% - 14%)	7.47% (6.68% - 8.68%)	19% (17.7% - 20.4%)	17.8% (16.6% - 19.1%)	21.5 (19.2 - 24)	12.7 (11.1 - 14.9)	36.8 (33.6 - 40.6)	37.9 (34.7 - 41.7)
United States of America	Male	16.7% (15.9% - 17.5%)	10.4% (9.5% - 12%)	26.1% (24.8% - 27.3%)	24.8% (23.4% - 26%)	27 (24.8 - 29.3)	17.5 (15.5 - 20.3)	48.1 (44.2 - 51.9)	51.3 (47.2 - 55.6)
Southern Latin America	Female	22.8% (21.1% - 24.7%)	20.3% (18.4% - 22.2%)	19.3% (17.7% - 20.8%)	18.4% (16.8% - 19.8%)	7.65 (6.73 - 8.67)	6.51 (5.64 - 7.48)	6.72 (5.97 - 7.54)	6.78 (5.98 - 7.63)
Southern Latin America	Male	28.1% (26.8% - 29.5%)	23.5% (22% - 25.1%)	24.7% (23.8% - 25.8%)	24.9% (23.9% - 26%)	9.4 (8.43 - 10.5)	7.83 (6.89 - 8.96)	8.42 (7.55 - 9.39)	10.3 (9.24 - 11.5)
Argentina	Female	20% (17.8% - 22.5%)	18% (15.8% - 20.5%)	16.7% (15.1% - 18.5%)	15.5% (13.9% - 17.2%)	4.54 (3.76 - 5.43)	3.91 (3.19 - 4.72)	3.94 (3.29 - 4.66)	3.92 (3.25 - 4.68)
Argentina	Male	25.9% (24.3% - 27.6%)	21.9% (20.2% - 23.5%)	24.6% (23.1% - 26.1%)	24.7% (23.3% - 26.3%)	5.82 (4.94 - 6.86)	4.96 (4.13 - 5.91)	5.52 (4.74 - 6.44)	6.6 (5.62 - 7.72)
Chile	Female	30.7% (27.5% - 34.3%)	26.6% (23.4% - 30.2%)	25.4% (22% - 28.6%)	24.9% (21.6% - 28.2%)	2.79 (2.4 - 3.23)	2.42 (2.04 - 2.84)	2.4 (2.05 - 2.8)	2.54 (2.17 - 2.95)
Chile	Male	34.2% (32% - 36.3%)	28% (25.6% - 30.6%)	24.4% (22.8% - 26.1%)	24.6% (23% - 26.3%)	3.17 (2.82 - 3.54)	2.62 (2.31 - 2.97)	2.4 (2.12 - 2.71)	3.19 (2.83 - 3.56)
Uruguay	Female	19.2% (17.4% - 21.3%)	13.1% (11.2% - 16.6%)	20% (18.2% - 22.1%)	19.4% (17.7% - 21.6%)	0.318 (0.27 - 0.37)	0.193 (0.156 - 0.249)	0.351 (0.299 - 0.406)	0.319 (0.269 - 0.373)
Uruguay	Male	24.8% (23.2% - 26.3%)	17% (15.6% - 19.2%)	28.2% (26.8% - 29.6%)	29.3% (27.9% - 30.9%)	0.403 (0.346 - 0.463)	0.257 (0.218 - 0.302)	0.492 (0.432 - 0.561)	0.533 (0.461 - 0.619)
Western Europe	Female	20.2% (19.6% - 20.8%)	15.2% (14.5% - 16.1%)	18.4% (17.7% - 19.2%)	18% (17.1% - 18.9%)	41.5 (39.6 - 43.2)	29.8 (28.2 - 31.7)	42.2 (40.1 - 44.4)	41.1 (38.7 - 43.5)
Western Europe	Male	25.1% (24.6% - 25.6%)	17.6% (17.1% - 18.2%)	25.1% (24.6% - 25.6%)	24.3% (23.9% - 24.9%)	52.2 (50 - 54.2)	35.8 (34 - 37.6)	63.3 (61 - 65.6)	66.9 (64.1 - 69.6)
Andorra	Female	22% (17.8% - 26.2%)	18.8% (14.9% - 22.8%)	16.7% (14.4% - 19.1%)	16.8% (14.5% - 19.4%)	0.0087 (0.0069 - 0.0108)	0.0049 (0.0039 - 0.0062)	0.007 (0.0059 - 0.0085)	0.0057 (0.0048 - 0.0068)
Andorra	Male	28% (25.4% - 30.6%)	21.9% (19.5% - 24.2%)	24.6% (22.8% - 26.3%)	24.3% (22.6% - 26.1%)	0.0125 (0.0108 - 0.0144)	0.0068 (0.0058 - 0.0079)	0.0124 (0.0109 - 0.0139)	0.0125 (0.0109 - 0.0142)
Austria	Female	22.9% (20.6% - 25.3%)	17.2% (14.4% - 22%)	16.5% (14.8% - 18.3%)	16.1% (14.4% - 18.1%)	0.983 (0.861 - 1.11)	0.735 (0.604 - 0.953)	0.778 (0.679 - 0.893)	0.796 (0.698 - 0.913)
Austria	Male	31.4% (29.5% - 33.6%)	25.4% (23.2% - 29.1%)	21.3% (20.1% - 22.8%)	21.5% (20% - 23.2%)	1.38 (1.22 - 1.52)	1.14 (1.01 - 1.33)	1.05 (0.935 - 1.17)	1.21 (1.08 - 1.34)
Belgium	Female	18.7% (17% - 20.5%)	13.3% (12% - 14.8%)	18.4% (16.3% - 20.2%)	17.6% (15.5% - 19.8%)	1.03 (0.916 - 1.16)	0.792 (0.698 - 0.905)	1.06 (0.905 - 1.2)	1.12 (0.955 - 1.29)
Belgium	Male	20.9% (19.8% - 22.3%)	12.3% (11.6% - 13.4%)	24.2% (22.7% - 25.6%)	23.6% (22.1% - 25.2%)	1.17 (1.04 - 1.3)	0.758 (0.678 - 0.841)	1.58 (1.4 - 1.76)	1.77 (1.6 - 1.97)
Cyprus	Female	19.5% (17% - 22%)	17.1% (14.7% - 19.4%)	9.76% (8.55% - 11.2%)	9.74% (8.45% - 11.2%)	0.137 (0.114 - 0.162)	0.116 (0.0966 - 0.137)	0.0686 (0.0563 - 0.081)	0.0744 (0.0609 - 0.0877)
Cyprus	Male	40.8% (38.6% - 42.9%)	37.5% (34.3% - 40.8%)	22.4% (21.1% - 23.9%)	23% (21.5% - 24.5%)	0.284 (0.246 - 0.324)	0.274 (0.233 - 0.316)	0.166 (0.142 - 0.193)	0.226 (0.196 - 0.259)
Denmark	Female	19% (17.2% - 20.8%)	11% (8.8% - 13.8%)	26.5% (24.5% - 28.6%)	29.5% (27.3% - 31.9%)	0.561 (0.497 - 0.633)	0.351 (0.278 - 0.442)	0.866 (0.777 - 0.958)	1.06 (0.959 - 1.17)
Denmark	Male	18.8% (17.7% - 20%)	9.96% (9.03% - 11.2%)	27.7% (26.3% - 29.1%)	30% (28.4% - 31.6%)	0.569 (0.508 - 0.64)	0.329 (0.288 - 0.38)	0.931 (0.84 - 1.02)	1.14 (1.04 - 1.25)
Finland	Female	15.6% (14% - 17.2%)	10.8% (9.65% - 12.1%)	21.4% (19.5% - 23.8%)	23.2% (20.8% - 25.9%)	0.382 (0.334 - 0.435)	0.251 (0.219 - 0.288)	0.567 (0.495 - 0.642)	0.59 (0.512 - 0.671)
Finland	Male	21.1% (19.9% - 22.3%)	15% (14.1% - 16.1%)	27.3% (26% - 28.9%)	27.9% (26.5% - 29.5%)	0.541 (0.478 - 0.598)	0.366 (0.329 - 0.405)	0.858 (0.76 - 0.95)	0.889 (0.794 - 0.981)
France	Female	28.3% (26.3% - 30.2%)	27.8% (25.7% - 29.9%)	18.5% (16.6% - 20.6%)	18.6% (16.5% - 21%)	8.57 (7.55 - 9.57)	7.56 (6.58 - 8.47)	6.25 (5.38 - 7.22)	5.83 (4.92 - 6.81)
France	Male	33.3% (31.8% - 34.9%)	29.4% (27.5% - 31.2%)	25.3% (24% - 26.9%)	24.9% (23.5% - 26.5%)	9.91 (8.79 - 11)	8.11 (7.04 - 9.17)	9.9 (8.8 - 11)	10.1 (8.84 - 11.3)
Germany	Female	20% (18.4% - 21.7%)	15.4% (14.1% - 16.8%)	17.8% (16.1% - 19.5%)	16.7% (14.8% - 18.9%)	7.94 (6.94 - 8.95)	5.82 (5.1 - 6.56)	7.91 (6.8 - 9.03)	7.23 (6.24 - 8.42)
Germany	Male	26.7% (25.2% - 28.3%)	20.2% (19% - 21.6%)	22.9% (21.6% - 24.3%)	21.5% (20.2% - 23%)	11.1 (9.81 - 12.3)	8.09 (7.2 - 8.97)	11.7 (10.4 - 13)	11.7 (10.5 - 12.9)
Greece	Female	28% (25.6% - 30.3%)	24.2% (19.8% - 29.4%)	13.7% (12.4% - 15%)	16.2% (14% - 18.5%)	1.39 (1.19 - 1.62)	0.781 (0.601 - 1.01)	0.684 (0.572 - 0.796)	0.577 (0.454 - 0.711)
Greece	Male	37.1% (35.6% - 38.5%)	28.2% (26% - 30.6%)	22.3% (21.1% - 23.5%)	23.5% (22.2% - 24.8%)	1.79 (1.54 - 2.03)	0.924 (0.762 - 1.1)	1.38 (1.18 - 1.57)	1.39 (1.17 - 1.64)
Iceland	Female	13% (11.2% - 14.9%)	7.26% (6.21% - 8.68%)	14.4% (12.4% - 16.7%)	14.1% (12.1% - 16.2%)	0.0223 (0.0188 - 0.0264)	0.0166 (0.0138 - 0.0199)	0.026 (0.0219 - 0.0306)	0.0349 (0.0288 - 0.0405)
Iceland	Male	13.2% (12.2% - 14.2%)	6.86% (6.03% - 7.79%)	25.9% (24.2% - 27.5%)	25.7% (24% - 27.3%)	0.024 (0.0212 - 0.027)	0.0167 (0.0143 - 0.0195)	0.0508 (0.0455 - 0.0566)	0.0724 (0.0653 - 0.081)
Ireland	Female	18.8% (16.8% - 20.9%)	11.5% (9.85% - 13.4%)	17% (15.4% - 18.6%)	15.4% (13.5% - 17.4%)	0.454 (0.391 - 0.525)	0.327 (0.277 - 0.39)	0.457 (0.398 - 0.518)	0.532 (0.451 - 0.623)
Ireland	Male	18.1% (16.7% - 19.3%)	9.64% (8.55% - 11.1%)	21.6% (20.3% - 23%)	20.9% (19.6% - 22.2%)	0.436 (0.384 - 0.489)	0.283 (0.242 - 0.338)	0.59 (0.519 - 0.654)	0.763 (0.679 - 0.854)
Israel	Female	12.6% (10.8% - 14.4%)	8.86% (7.22% - 10.8%)	13.6% (11.8% - 15.6%)	13.4% (11.5% - 15.5%)	0.597 (0.488 - 0.72)	0.555 (0.422 - 0.703)	0.662 (0.53 - 0.803)	0.877 (0.704 - 1.08)
Israel	Male	22.6% (21% - 24.3%)	15.2% (14% - 16.6%)	22.5% (21.1% - 24.1%)	22.3% (20.9% - 23.9%)	1.07 (0.902 - 1.25)	0.976 (0.812 - 1.15)	1.13 (0.96 - 1.33)	1.63 (1.37 - 1.89)
Italy	Female	18.7% (17.1% - 20.4%)	16.3% (14.6% - 18%)	11.9% (10.8% - 13.2%)	10.5% (9.23% - 11.8%)	5.04 (4.42 - 5.69)	3.18 (2.75 - 3.62)	3.75 (3.27 - 4.29)	2.7 (2.26 - 3.19)
Italy	Male	25% (23.8% - 26.5%)	19.9% (18.6% - 21.2%)	20.6% (19.4% - 21.8%)	18.4% (17.2% - 19.5%)	6.79 (6.1 - 7.49)	4.05 (3.62 - 4.48)	7.31 (6.53 - 8.02)	6.22 (5.53 - 6.92)
Luxembourg	Female	18.9% (16.8% - 20.8%)	13.8% (12% - 15.6%)	17.8% (15.7% - 19.9%)	17.5% (15.4% - 19.6%)	0.0584 (0.0499 - 0.0662)	0.061 (0.052 - 0.0694)	0.0592 (0.0516 - 0.0681)	0.0879 (0.0769 - 0.0995)
Luxembourg	Male	23% (21.6% - 24.5%)	16.1% (14.9% - 17.3%)	24.8% (23.4% - 26.3%)	24.6% (23.2% - 26.2%)	0.0767 (0.069 - 0.0857)	0.076 (0.0689 - 0.0838)	0.0902 (0.0808 - 0.0993)	0.142 (0.129 - 0.154)
Malta	Female	19.6% (17.4% - 22.1%)	17.5% (14.4% - 20.2%)	16.4% (14.2% - 19%)	19.8% (17.1% - 22.8%)	0.0381 (0.032 - 0.0455)	0.0403 (0.0319 - 0.0477)	0.0334 (0.0277 - 0.0403)	0.0486 (0.0412 - 0.058)
Malta	Male	23.6% (21.8% - 25.5%)	17.2% (14.2% - 19.8%)	21.8% (20.3% - 23.2%)	21.8% (20.2% - 23.1%)	0.0517 (0.0443 - 0.0604)	0.0451 (0.0351 - 0.054)	0.0567 (0.0488 - 0.0648)	0.0713 (0.062 - 0.0798)
Monaco	Female	21.2% (16.7% - 26%)	17.8% (13.7% - 22%)	17.1% (14.7% - 19.6%)	17.2% (14.7% - 19.7%)	0.0036 (0.0028 - 0.0044)	0.0021 (0.0016 - 0.0027)	0.0033 (0.0027 - 0.0039)	0.0024 (0.002 - 0.0029)
Monaco	Male	25.7% (23.3% - 28.5%)	20.3% (17.9% - 22.8%)	24.6% (22.9% - 26.4%)	24.3% (22.5% - 26%)	0.0044 (0.0038 - 0.005)	0.0026 (0.0022 - 0.003)	0.0057 (0.0051 - 0.0063)	0.0045 (0.004 - 0.0051)
Netherlands	Female	17.4% (15.7% - 18.9%)	10.2% (9% - 11.5%)	26.9% (24.6% - 29.4%)	26.6% (24% - 29.5%)	1.49 (1.3 - 1.69)	0.886 (0.756 - 1.02)	2.59 (2.28 - 2.95)	2.7 (2.34 - 3.1)
Netherlands	Male	20.8% (19.5% - 22%)	12.2% (11.3% - 13.1%)	29.6% (28.1% - 31.4%)	29.4% (27.8% - 31.4%)	1.78 (1.59 - 1.98)	1.07 (0.966 - 1.19)	2.96 (2.67 - 3.31)	3.25 (2.93 - 3.62)
Norway	Female	15.7% (13.8% - 18%)	7.65% (6.35% - 9.22%)	15.3% (13% - 17.8%)	13.2% (11.1% - 15.6%)	0.413 (0.352 - 0.48)	0.224 (0.184 - 0.273)	0.392 (0.329 - 0.46)	0.378 (0.313 - 0.444)
Norway	Male	19% (17.6% - 20.6%)	9.82% (8.77% - 11.1%)	19.4% (17.9% - 21%)	18.8% (17.2% - 20.2%)	0.527 (0.467 - 0.593)	0.302 (0.261 - 0.348)	0.553 (0.484 - 0.612)	0.629 (0.557 - 0.696)
Portugal	Female	21.8% (20% - 23.9%)	25% (22.9% - 27.2%)	9.58% (8.34% - 11.1%)	9.69% (8.28% - 11.2%)	1.01 (0.857 - 1.17)	0.977 (0.839 - 1.13)	0.453 (0.38 - 0.534)	0.394 (0.331 - 0.462)
Portugal	Male	30.2% (28.7% - 31.7%)	25.1% (23.8% - 26.6%)	19.4% (18.1% - 20.6%)	18.2% (17% - 19.4%)	1.43 (1.24 - 1.62)	1.08 (0.947 - 1.21)	1.15 (0.994 - 1.31)	1.1 (0.965 - 1.25)
San Marino	Female	17% (14.2% - 20%)	12.5% (10.3% - 14.8%)	15.4% (13.3% - 17.7%)	15.2% (13.1% - 17.4%)	0.0027 (0.0021 - 0.0033)	0.0015 (0.0012 - 0.0019)	0.0027 (0.0022 - 0.0032)	0.0023 (0.0019 - 0.0028)
San Marino	Male	19.6% (18% - 21.4%)	13.6% (11.7% - 15.7%)	25.3% (23.5% - 27%)	25.2% (23.4% - 26.9%)	0.003 (0.0025 - 0.0035)	0.0018 (0.0014 - 0.0021)	0.0047 (0.004 - 0.0054)	0.0046 (0.0039 - 0.0054)
Spain	Female	21.8% (19.9% - 23.7%)	14.4% (11% - 18.9%)	16.1% (14.4% - 18%)	17% (14.8% - 19.7%)	4.54 (3.96 - 5.09)	2.97 (2.21 - 4)	3.56 (3.06 - 4.1)	3.77 (3.15 - 4.45)
Spain	Male	25.8% (24.6% - 27.2%)	13.4% (11.1% - 16.1%)	24.3% (22.8% - 25.8%)	24.3% (22.8% - 25.9%)	5.68 (5.01 - 6.32)	3.06 (2.5 - 3.78)	6.48 (5.78 - 7.19)	7.74 (7 - 8.48)

Supplementary Results Table S1. Global all-age and age-standardised prevalence of current and former smokers by location, sex, and scenario, 2022 and 2050

Location	Sex	Current Smoking Prevalence 2022	Current Smoking Prevalence 2050	Former Smoking Prevalence 2022	Former Smoking Prevalence 2050	Current Smokers (millions) 2022	Current Smokers (millions) 2050	Former Smokers (millions) 2022	Former Smokers (millions) 2050
Sweden	Female	12.8% (11.4% - 14.2%)	7.93% (6.83% - 9.17%)	27.3% (24.8% - 30.4%)	27.7% (25% - 30.7%)	0.66 (0.575 - 0.752)	0.523 (0.444 - 0.611)	1.53 (1.33 - 1.76)	1.97 (1.73 - 2.27)
Sweden	Male	11.4% (10.5% - 12.3%)	7.74% (6.66% - 8.92%)	28.4% (26.8% - 30%)	29.6% (27.9% - 31.3%)	0.608 (0.544 - 0.68)	0.525 (0.442 - 0.61)	1.69 (1.52 - 1.86)	2.24 (2.03 - 2.45)
Switzerland	Female	20.6% (18.1% - 23.2%)	16.8% (14.7% - 19.2%)	18.9% (16.6% - 21.4%)	20% (17.5% - 22.9%)	0.919 (0.791 - 1.06)	0.791 (0.681 - 0.914)	0.857 (0.727 - 1)	0.988 (0.842 - 1.17)
Switzerland	Male	25.4% (23.7% - 27.1%)	19.2% (17.5% - 20.9%)	23.2% (21.6% - 24.9%)	23% (21.4% - 24.7%)	1.15 (1.02 - 1.29)	0.932 (0.834 - 1.04)	1.19 (1.05 - 1.32)	1.4 (1.27 - 1.55)
United Kingdom	Female	15.2% (13.6% - 16.8%)	8.13% (6.89% - 9.71%)	26.2% (24% - 28.6%)	22.5% (20.3% - 25.1%)	5.14 (4.54 - 5.7)	2.85 (2.4 - 3.44)	9.9 (8.91 - 10.9)	9.47 (8.41 - 10.7)
United Kingdom	Male	17.7% (16.6% - 18.8%)	10% (8.94% - 11.4%)	33.6% (32% - 35.4%)	30.8% (29.2% - 32.7%)	5.72 (5.29 - 6.15)	3.39 (2.96 - 3.92)	12.5 (11.6 - 13.4)	13.2 (12 - 14.2)
Latin America and Caribbean	Female	7.31% (6.91% - 7.7%)	4.33% (4% - 4.7%)	12.8% (12.2% - 13.4%)	11.5% (11% - 12.1%)	22.5 (20.9 - 24.2)	14.2 (12.8 - 15.7)	39.4 (36.2 - 42.2)	44.3 (40.6 - 47.9)
Latin America and Caribbean	Male	15.8% (15.3% - 16.4%)	10.5% (9.78% - 11.3%)	25.4% (24.9% - 26%)	25.2% (24.5% - 26%)	47.3 (44.4 - 50.4)	34.2 (31 - 37.8)	73.6 (69.2 - 77.9)	91.8 (84.9 - 98.9)
Andean Latin America	Female	5.74% (5.22% - 6.36%)	5.64% (5.04% - 6.31%)	7.24% (6.6% - 7.94%)	7.59% (6.94% - 8.35%)	1.93 (1.71 - 2.19)	2.14 (1.85 - 2.45)	2.37 (2.1 - 2.66)	3.42 (3 - 3.9)
Andean Latin America	Male	21.4% (20.6% - 22.2%)	21.3% (20.2% - 22.4%)	22.1% (21.1% - 23%)	21.9% (20.9% - 22.8%)	7.3 (6.74 - 7.91)	8.36 (7.57 - 9.32)	7.05 (6.48 - 7.66)	9.86 (8.81 - 10.9)
Bolivia (Plurinational State of)	Female	8.13% (6.89% - 9.65%)	9.51% (7.44% - 11.5%)	9.8% (8.5% - 11.3%)	10.2% (8.82% - 11.8%)	0.482 (0.386 - 0.596)	0.747 (0.568 - 0.946)	0.548 (0.458 - 0.663)	0.835 (0.686 - 1.02)
Bolivia (Plurinational State of)	Male	18.4% (16.9% - 20%)	21.1% (16.5% - 23.6%)	22.6% (21% - 24.3%)	22.3% (20.8% - 24%)	1.1 (0.934 - 1.27)	1.71 (1.3 - 2.08)	1.23 (1.04 - 1.42)	1.8 (1.51 - 2.12)
Ecuador	Female	4.54% (3.78% - 5.49%)	3.55% (2.81% - 4.41%)	8.73% (7.48% - 10.1%)	8.88% (7.61% - 10.2%)	0.422 (0.326 - 0.529)	0.398 (0.305 - 0.519)	0.782 (0.63 - 0.939)	1.13 (0.884 - 1.37)
Ecuador	Male	22.5% (21% - 24%)	19.8% (17.6% - 21.7%)	21.9% (20.5% - 23.4%)	21.9% (20.4% - 23.6%)	2.06 (1.72 - 2.38)	2.29 (1.88 - 2.7)	1.88 (1.59 - 2.16)	2.72 (2.23 - 3.21)
Peru	Female	5.64% (4.87% - 6.46%)	5.27% (4.49% - 6.15%)	5.74% (4.95% - 6.65%)	5.85% (5.03% - 6.79%)	1.03 (0.864 - 1.2)	0.973 (0.808 - 1.17)	1.04 (0.876 - 1.23)	1.44 (1.18 - 1.75)
Peru	Male	22% (21% - 23.2%)	22.6% (21% - 24.9%)	22% (20.5% - 23.6%)	21.6% (20.1% - 23.1%)	4.15 (3.72 - 4.57)	4.36 (3.74 - 5.06)	3.94 (3.51 - 4.39)	5.35 (4.61 - 6.17)
Caribbean	Female	7.8% (7.02% - 8.65%)	4.62% (4.04% - 5.62%)	10.1% (9.43% - 10.7%)	9.68% (8.96% - 10.3%)	1.91 (1.69 - 2.15)	1.21 (0.994 - 1.5)	2.52 (2.28 - 2.76)	2.81 (2.48 - 3.16)
Caribbean	Male	16.9% (16% - 17.7%)	11% (10.1% - 12.4%)	21.2% (20.5% - 21.9%)	21.3% (20.5% - 22.1%)	4.06 (3.7 - 4.42)	2.81 (2.44 - 3.31)	5.06 (4.69 - 5.48)	6.09 (5.38 - 6.85)
Antigua and Barbuda	Female	5.5% (4.37% - 6.78%)	6.27% (5% - 7.78%)	9.87% (8.6% - 11.4%)	9.89% (8.6% - 11.4%)	0.0025 (0.0019 - 0.0032)	0.0026 (0.002 - 0.0033)	0.0047 (0.0039 - 0.0057)	0.0053 (0.0044 - 0.0065)
Antigua and Barbuda	Male	11.9% (10.5% - 13.4%)	13.7% (12% - 15.5%)	22% (20.5% - 23.7%)	21.7% (20.2% - 23.4%)	0.0054 (0.0045 - 0.0064)	0.006 (0.0049 - 0.0072)	0.0099 (0.0084 - 0.0113)	0.0117 (0.0101 - 0.0137)
Bahamas	Female	3.42% (2.66% - 4.33%)	2.97% (2.34% - 3.81%)	9.03% (7.67% - 10.4%)	9.05% (7.69% - 10.5%)	0.0067 (0.005 - 0.0087)	0.0057 (0.0043 - 0.0074)	0.0187 (0.0151 - 0.0229)	0.0238 (0.019 - 0.0291)
Bahamas	Male	11.6% (10.5% - 12.8%)	12.3% (11.2% - 13.6%)	21.3% (19.8% - 22.9%)	21.3% (19.8% - 22.9%)	0.0223 (0.0186 - 0.0261)	0.0238 (0.0198 - 0.0279)	0.0396 (0.0339 - 0.0459)	0.0513 (0.0435 - 0.0596)
Barbados	Female	4.14% (3.29% - 5.25%)	3.06% (2.37% - 3.94%)	6.15% (5.22% - 7.18%)	6.33% (5.35% - 7.41%)	0.0052 (0.004 - 0.0068)	0.0028 (0.0021 - 0.0037)	0.0093 (0.0076 - 0.0113)	0.0082 (0.0067 - 0.0101)
Barbados	Male	11.7% (10.4% - 13%)	8.13% (6.89% - 10.2%)	21% (19.4% - 22.6%)	21.3% (19.6% - 23%)	0.0161 (0.0136 - 0.0187)	0.0097 (0.0078 - 0.0121)	0.0328 (0.0281 - 0.0381)	0.0331 (0.0277 - 0.0392)
Belize	Female	4.5% (3.53% - 5.57%)	4.48% (3.41% - 5.62%)	9.22% (7.93% - 10.6%)	9.4% (8.08% - 10.8%)	0.0103 (0.0077 - 0.0131)	0.0142 (0.0106 - 0.0185)	0.0185 (0.0151 - 0.0221)	0.0333 (0.0274 - 0.0397)
Belize	Male	20.2% (18.3% - 22%)	19.7% (17.4% - 21.8%)	20.3% (18.9% - 21.7%)	20.2% (18.9% - 21.6%)	0.0437 (0.0368 - 0.051)	0.065 (0.0542 - 0.0767)	0.039 (0.0332 - 0.0452)	0.0664 (0.0558 - 0.0774)
Bermuda	Female	8.5% (6.82% - 10.5%)	8.25% (6.59% - 10.2%)	17.9% (15.9% - 20.2%)	18.8% (16.7% - 21.2%)	0.0026 (0.002 - 0.0032)	0.0018 (0.0014 - 0.0022)	0.007 (0.0059 - 0.0081)	0.0067 (0.0056 - 0.0079)
Bermuda	Male	17.7% (16% - 19.4%)	17.5% (15.8% - 19.3%)	23% (21.7% - 24.6%)	23.5% (22.1% - 25.1%)	0.0053 (0.0046 - 0.0061)	0.0037 (0.0032 - 0.0043)	0.0086 (0.0077 - 0.0097)	0.0082 (0.0071 - 0.0093)
Cuba	Female	13.6% (11.6% - 16.1%)	8.7% (7.22% - 10.9%)	9.74% (8.32% - 11.1%)	9.22% (7.84% - 10.6%)	0.8 (0.633 - 0.997)	0.379 (0.295 - 0.488)	0.637 (0.508 - 0.762)	0.53 (0.421 - 0.64)
Cuba	Male	27.5% (25.4% - 29.6%)	20.5% (18.7% - 22.6%)	20.2% (18.8% - 21.7%)	20.6% (19.2% - 22.1%)	1.64 (1.4 - 1.88)	0.948 (0.795 - 1.11)	1.29 (1.11 - 1.49)	1.29 (1.09 - 1.51)
Dominica	Female	6.14% (4.78% - 7.93%)	5.5% (4.3% - 7.07%)	6.98% (5.98% - 8.13%)	6.97% (5.98% - 8.09%)	0.0019 (0.0014 - 0.0025)	0.0015 (0.0011 - 0.0019)	0.0024 (0.0019 - 0.0029)	0.0025 (0.002 - 0.0031)
Dominica	Male	13.6% (12% - 15.2%)	12.7% (11% - 14.6%)	28.2% (26.3% - 29.9%)	28.1% (26.1% - 29.8%)	0.0045 (0.0038 - 0.0054)	0.0036 (0.0029 - 0.0045)	0.01 (0.0085 - 0.0117)	0.0098 (0.0083 - 0.0116)
Dominican Republic	Female	8.08% (6.6% - 9.51%)	5.43% (4.09% - 7.4%)	11.1% (9.82% - 12.8%)	10.5% (9.25% - 12%)	0.435 (0.339 - 0.535)	0.402 (0.278 - 0.568)	0.593 (0.48 - 0.711)	0.783 (0.619 - 0.964)
Dominican Republic	Male	11.6% (10.5% - 12.6%)	8.68% (7.24% - 11.1%)	21.1% (19.7% - 22.6%)	21.3% (19.8% - 22.8%)	0.633 (0.524 - 0.75)	0.598 (0.45 - 0.828)	1.11 (0.929 - 1.3)	1.49 (1.2 - 1.8)
Grenada	Female	5.15% (4.09% - 6.55%)	4.34% (3.41% - 5.54%)	9.68% (8.3% - 11.2%)	9.85% (8.45% - 11.4%)	0.0025 (0.0019 - 0.0033)	0.0018 (0.0013 - 0.0025)	0.005 (0.0041 - 0.0061)	0.0053 (0.0043 - 0.0066)
Grenada	Male	15.4% (13.9% - 17.1%)	13.4% (11.7% - 15.9%)	20.7% (19.1% - 22.2%)	20.8% (19.2% - 22.3%)	0.0084 (0.0071 - 0.0097)	0.0063 (0.005 - 0.0077)	0.0109 (0.0092 - 0.0126)	0.0117 (0.0094 - 0.0141)
Guyana	Female	4.2% (3.46% - 5.18%)	3.73% (3.06% - 4.63%)	8.93% (7.67% - 10.3%)	8.99% (7.71% - 10.4%)	0.0161 (0.0124 - 0.0206)	0.011 (0.0075 - 0.0151)	0.0333 (0.0273 - 0.0406)	0.0387 (0.0281 - 0.0517)
Guyana	Male	22.4% (21% - 24%)	21.9% (20.3% - 23.7%)	20.2% (18.8% - 21.9%)	20.2% (18.7% - 21.8%)	0.0849 (0.073 - 0.0973)	0.0466 (0.0334 - 0.0613)	0.07 (0.06 - 0.0811)	0.0926 (0.0654 - 0.122)
Haiti	Female	2.86% (2.22% - 3.61%)	1.82% (1.36% - 2.35%)	9.68% (8.41% - 11.2%)	9.62% (8.33% - 11.1%)	0.203 (0.144 - 0.272)	0.146 (0.1 - 0.2)	0.582 (0.453 - 0.739)	0.824 (0.611 - 1.05)
Haiti	Male	9.12% (8.3% - 10%)	5.89% (5% - 6.87%)	21.1% (19.8% - 22.6%)	21.4% (20.1% - 23%)	0.581 (0.466 - 0.705)	0.499 (0.372 - 0.659)	1.16 (0.93 - 1.38)	1.76 (1.31 - 2.18)
Jamaica	Female	7.42% (6.33% - 8.59%)	5.57% (4.66% - 6.65%)	8.84% (7.63% - 10.3%)	8.52% (7.35% - 9.99%)	0.1 (0.0826 - 0.122)	0.06 (0.0469 - 0.0782)	0.125 (0.101 - 0.153)	0.132 (0.104 - 0.165)
Jamaica	Male	18.3% (16.8% - 19.8%)	13.1% (11.8% - 15%)	21.5% (20% - 23.2%)	22% (20.3% - 23.7%)	0.258 (0.221 - 0.297)	0.16 (0.131 - 0.194)	0.294 (0.255 - 0.34)	0.334 (0.272 - 0.398)
Puerto Rico	Female	8.3% (7.14% - 9.86%)	6.71% (5.7% - 7.95%)	11.8% (10.7% - 12.9%)	12% (10.8% - 13.2%)	0.127 (0.107 - 0.147)	0.0592 (0.0484 - 0.0697)	0.232 (0.206 - 0.259)	0.199 (0.167 - 0.232)
Puerto Rico	Male	14.9% (13.9% - 16%)	12.3% (11.3% - 13.3%)	23.7% (22.5% - 24.7%)	23.2% (22% - 24.4%)	0.216 (0.196 - 0.239)	0.101 (0.0859 - 0.118)	0.442 (0.404 - 0.48)	0.395 (0.334 - 0.453)
Saint Kitts and Nevis	Female	2.8% (2.15% - 3.6%)	2.22% (1.67% - 2.9%)	4.88% (4.17% - 5.63%)	4.97% (4.25% - 5.76%)	0.0008 (0.0006 - 0.0011)	0.0006 (0.0004 - 0.0008)	0.0014 (0.0011 - 0.0018)	0.0018 (0.0014 - 0.0024)
Saint Kitts and Nevis	Male	8.8% (7.7% - 9.81%)	6.44% (5.32% - 8.39%)	18.1% (16.8% - 19.6%)	18.3% (17% - 19.8%)	0.0027 (0.0021 - 0.0033)	0.0016 (0.0012 - 0.0022)	0.0053 (0.0043 - 0.0064)	0.0056 (0.0045 - 0.0068)
Saint Lucia	Female	4.93% (3.95% - 6.05%)	3.61% (2.82% - 4.56%)	9.24% (7.98% - 10.6%)	9.5% (8.18% - 10.9%)	0.0042 (0.0031 - 0.0052)	0.0026 (0.0019 - 0.0034)	0.0087 (0.0071 - 0.0104)	0.0103 (0.0083 - 0.0125)
Saint Lucia	Male	16.5% (15% - 18.2%)	12.1% (10.7% - 14.6%)	20.7% (19.4% - 22.3%)	21% (19.6% - 22.6%)	0.0152 (0.0126 - 0.0177)	0.0099 (0.0079 - 0.0126)	0.0191 (0.0164 - 0.0218)	0.0222 (0.0186 - 0.0262)
Saint Vincent and the Grenadines	Female	4.69% (3.86% - 5.75%)	4.22% (3.46% - 5.22%)	8.94% (7.67% - 10.4%)	9.01% (7.74% - 10.4%)	0.0025 (0.0019 - 0.0031)	0.0016 (0.0013 - 0.0021)	0.0052 (0.0042 - 0.0063)	0.0055 (0.0044 - 0.0069)
Saint Vincent and the Grenadines	Male	18.7% (17.1% - 20.4%)	17.1% (15.6% - 18.7%)	20.7% (19.2% - 22.2%)	20.7% (19.2% - 22.2%)	0.0107 (0.0092 - 0.0124)	0.0074 (0.006 - 0.0088)	0.0127 (0.011 - 0.0146)	0.013 (0.0106 - 0.0157)
Suriname	Female	8.34% (6.75% - 10%)	5.83% (4.57% - 7.45%)	9% (7.77% - 10.4%)	9.5% (8.19% - 10.9%)	0.0246 (0.019 - 0.0308)	0.0181 (0.0134 - 0.0242)	0.0275 (0.0224 - 0.033)	0.0358 (0.0285 - 0.0439)
Suriname	Male	28.8% (26.6% - 31.3%)	21% (18.7% - 24.6%)	19.8% (18.4% - 21.3%)	20.1% (18.6% - 21.6%)	0.0844 (0.0719 - 0.0968)	0.0677 (0.0544 - 0.0829)	0.057 (0.0487 - 0.0656)	0.0737 (0.0611 - 0.0875)
Trinidad and Tobago	Female	8.23% (6.56% - 9.87%)	7.66% (6.1% - 9.21%)	11.1% (9.47% - 12.9%)	10.6% (9.01% - 12.2%)	0.0523 (0.041 - 0.0655)	0.0297 (0.0223 - 0.0384)	0.0774 (0.0633 - 0.0932)	0.0578 (0.0458 - 0.0729)
Trinidad and Tobago	Male	26.1% (24.1% - 28.3%)	24.2% (21.9% - 26.4%)	23.9% (22.3% - 25.6%)	22.8% (21.2% - 24.4%)	0.18 (0.152 - 0.208)	0.102 (0.0806 - 0.126)	0.177 (0.147 - 0.204)	0.147 (0.116 - 0.179)
United States Virgin Islands	Female	5.1% (4% - 6.35%)	3.93% (3.04% - 4.94%)	7.96% (6.94% - 8.95%)	7.2% (6.31% - 8.19%)	0.0022 (0.0017 - 0.0027)	0.0013 (0.001 - 0.0016)	0.0043 (0.0037 - 0.0049)	0.0032 (0.0026 - 0.0039)
United States Virgin Islands	Male	7.82% (7.04% - 8.72%)	5.98% (5.32% - 6.89%)	15.7% (14.6% - 16.8%)	15.2% (14.1% - 16.4%)	0.0033 (0.0028 - 0.0037)	0.0018 (0.0014 - 0.0022)	0.0079 (0.0072 - 0.0087)	0.0059 (0.0049 - 0.007)
Central Latin America	Female	8.29% (7.6% - 8.94%)	5.82% (5.23% - 6.37%)	8.82% (8.21% - 9.47%)	7.92% (7.36% - 8.51%)	10.9 (9.93 - 11.9)	7.91 (7.02 - 8.83)	11.5 (10.5 - 12.6)	11.7 (10.5 - 12.8)
Central Latin America	Male	19.4% (18.8% - 20.2%)	13.5% (12.3% - 14.6%)	24.7% (23.8% - 25.5%)	24.3% (23.3% - 25.4%)	24.5 (23 - 26)	18.4 (16.3 - 20.8)	30.4 (28.8 - 32.2)	34.9 (31.6 - 38.1)
Colombia	Female	7.67% (6.51% - 8.95%)	4.46% (3.58% - 5.7%)	8.08% (6.95% - 9.39%)	7.57% (6.5% - 8.81%)	1.97 (1.62 - 2.34)	1.24 (0.95 - 1.64)	2.04 (1.71 - 2.43)	2.17 (1.8 - 2.59)
Colombia	Male	12.4% (11.3% - 13.7%)	5.39% (4.68% - 6.68%)	23.2% (21.5% - 25%)	24% (22.3% - 25.9%)	3.06 (2.68 - 3.45)	1.42 (1.17 - 1.82)	5.61 (5.03 - 6.31)	6.93 (6.03 - 7.87)
Costa Rica	Female	6.42% (5.33% - 7.59%)	3.36% (2.68% - 4.22%)	11.1% (9.81% - 12.5%)	10.4% (9.13% - 11.7%)	0.157 (0.127 - 0.191)	0.0785 (0.063 - 0.0993)	0.277 (0.235 - 0.329)	0.287 (0.244 - 0.343)
Costa Rica	Male	13.1% (12% - 14.2%)	7.15% (6.5% - 7.84%)	27% (25.6% - 28.7%)	26.8% (25.3% - 28.5%)	0.308 (0.264 - 0.355)	0.173 (0.149 - 0.203)	0.627 (0.553 - 0.708)	0.748 (0.651 - 0.849)
El Salvador	Female	4.33% (3.47% - 5.36%)	4.82% (3.77% - 6.17%)	8.72% (7.52% - 10.1%)	8.51% (7.34% - 9.88%)	0.143 (0.109 - 0.183)	0.117 (0.0784 - 0.159)	0.298 (0.233 - 0.361)	0.241 (0.182 - 0.31)
El Salvador	Male	16.8% (15.2% - 18.4%)	20.1% (17.9% - 22.4%)	24.2% (22.3% - 26.2%)	23.2% (21.3% - 25.1%)	0.516 (0.418 - 0.605)	0.456 (0.328 - 0.576)	0.715 (0.586 - 0.832)	0.614 (0.457 - 0.778)
Guatemala	Female	4.66% (3.78% - 5.78%)	3.86% (3.09% - 4.84%)	7.87% (6.78% - 9.14%)	7.69% (6.61% - 8.98%)	0.398 (0.318 - 0.503)	0.341 (0.265 - 0.444)	0.631 (0.524 - 0.754)	0.786 (0.638 - 0.939)
Guatemala	Male	18.4% (17.1% - 19.8%)	15.4% (13.9% - 17.3%)	23.8% (22.1% - 25.6%)	23.2% (21.4% - 24.9%)	1.48 (1.31 - 1.67)	1.42 (1.2 - 1.7)	1.73 (1.53 - 1.95)	2.22 (1.89 - 2.6)
Honduras	Female	5.31% (4.3% - 6.45%)	5.02% (4.06% - 6.14%)	6.56% (5.59% - 7.75%)	6.49% (5.51% - 7.64%)	0.282 (0.219 - 0.348)	0.347 (0.269 - 0.44)	0.338 (0.274 - 0.416)	0.454 (0.365 - 0.56)
Honduras	Male	22.5% (20.9% - 24.2%)	19.9% (18.2% - 21.7%)	23.4% (21.6% - 25.2%)	22.7% (21% - 24.5%)	1.12 (0.964 - 1.28)	1.38 (1.17 - 1.62)	1.1 (0.942 - 1.26)	1.53 (1.29 - 1.78)
Mexico	Female	9.31% (8.28% - 10.4%)	6.69% (5.76% - 7.59%)	9.58% (8.5% - 10.7%)	8.24% (7.27% - 9.23%)	6.22 (5.37 - 7.11)	4.64 (3.92 - 5.38)	6.4 (5.59 - 7.34)	6.23 (5.38 - 7.08)
Mexico	Male	22.9% (21.8% - 24%)	15.5% (13.5% - 17.6%)	26.2% (24.5% - 27.8%)	25.4% (23.5% - 27.3%)	14.8 (13.4 - 16.1)	11.1 (9.33 - 13.1)	16.5 (15 - 18.1)	18.8 (16.8 - 20.9)
Nicaragua	Female	6.7% (5.23% - 8.34%)	6.73% (5.19% - 8.42%)	6.91% (5.91% - 8.08%)	6.76% (5.77% - 7.94%)	0.238 (0.177 - 0.316)	0.236 (0.165 - 0.316)	0.235 (0.184 - 0.3)	0.267 (0.205 - 0.336)
Nicaragua	Male	23.6% (21.4% - 25.9%)	23.3% (19.4% - 26.3%)	23.4% (21.7% - 25.1%)	22.6% (20.9% - 24.3%)	0.804 (0.65 - 0.962)	0.897 (0.66 - 1.13)	0.741 (0.611 - 0.891)	0.896 (0.708 - 1.1)
Panama	Female	4.33% (3.54% - 5.24%)	2.92% (2.36% - 3.6%)	5.51% (4.75% - 6.31%)	5.4% (4.66% - 6.19%)	0.093 (0.0712 - 0.116)	0.0814 (0.0631 - 0.101)	0.121 (0.1 - 0.145)	0.191 (0.156 - 0.229)

Supplementary Results Table S1. Global all-age and age-standardised prevalence of current and former smokers by location, sex, and scenario, 2022 and 2050

Location	Sex	Current Smoking Prevalence 2022	Current Smoking Prevalence 2050	Former Smoking Prevalence 2022	Former Smoking Prevalence 2050	Current Smokers (millions) 2022	Current Smokers (millions) 2050	Former Smokers (millions) 2022	Former Smokers (millions) 2050
Panama	Male	11.4% (10.3% - 12.5%)	7.57% (6.7% - 8.53%)	11.8% (10.9% - 12.7%)	11.5% (10.6% - 12.4%)	0.251 (0.21 - 0.295)	0.226 (0.187 - 0.268)	0.258 (0.216 - 0.3)	0.382 (0.32 - 0.443)
Venezuela (Bolivarian Republic of)	Female	9.73% (7.6% - 12.1%)	6.81% (5.29% - 8.59%)	8.6% (7.1% - 10%)	8.12% (6.68% - 9.5%)	1.37 (1.06 - 1.75)	0.836 (0.624 - 1.11)	1.19 (0.948 - 1.43)	1.02 (0.776 - 1.31)
Venezuela (Bolivarian Republic of)	Male	17.2% (15.5% - 19.2%)	12.2% (10.8% - 13.7%)	23.7% (22% - 25.5%)	23.4% (21.7% - 25.1%)	2.26 (1.89 - 2.63)	1.36 (1.01 - 1.72)	3.08 (2.67 - 3.56)	2.75 (2.01 - 3.51)
Tropical Latin America	Female	6.36% (5.82% - 6.99%)	2.09% (1.86% - 2.46%)	18.5% (17.3% - 19.7%)	17% (15.8% - 18.2%)	7.83 (6.59 - 9.13)	2.96 (2.45 - 3.64)	22.5 (19.1 - 26.1)	26.3 (22.3 - 30.2)
Tropical Latin America	Male	10% (9.54% - 10.5%)	3.46% (3.22% - 3.79%)	27.7% (26.6% - 28.7%)	27.7% (26.5% - 28.9%)	11.6 (9.88 - 13.4)	4.63 (3.89 - 5.36)	30.8 (26.8 - 35.1)	41 (35.2 - 46.9)
Brazil	Female	6.34% (5.78% - 6.98%)	2.03% (1.78% - 2.39%)	18.6% (17.4% - 19.8%)	17% (15.9% - 18.3%)	7.59 (6.34 - 8.85)	2.78 (2.28 - 3.44)	21.9 (18.6 - 25.5)	25.6 (21.6 - 29.6)
Brazil	Male	9.71% (9.21% - 10.2%)	3.18% (2.95% - 3.47%)	27.7% (26.6% - 28.8%)	27.8% (26.5% - 29%)	10.9 (9.26 - 12.6)	4.11 (3.47 - 4.75)	29.9 (25.9 - 34.3)	39.8 (33.9 - 45.6)
Paraguay	Female	6.98% (5.76% - 8.4%)	4.21% (3.44% - 5.15%)	16.2% (14.3% - 18.2%)	15.4% (13.6% - 17.4%)	0.245 (0.188 - 0.308)	0.18 (0.129 - 0.235)	0.549 (0.441 - 0.682)	0.707 (0.537 - 0.894)
Paraguay	Male	20.7% (19% - 22.4%)	12.1% (10.8% - 13.6%)	26.4% (24.6% - 28.3%)	27.2% (25.5% - 29.2%)	0.734 (0.595 - 0.886)	0.525 (0.404 - 0.665)	0.884 (0.717 - 1.06)	1.21 (0.94 - 1.53)
North Africa and Middle East	Female	5.29% (4.93% - 5.69%)	4.62% (4.32% - 4.94%)	2.54% (2.37% - 2.73%)	2.27% (2.1% - 2.54%)	16.5 (15.2 - 18)	18.9 (17.1 - 20.6)	7.53 (6.92 - 8.25)	9.92 (8.87 - 11.2)
North Africa and Middle East	Male	29.7% (29.2% - 30.3%)	29.3% (28.6% - 30%)	15.6% (15.3% - 16%)	15.4% (15.1% - 15.8%)	101 (96.3 - 105)	130 (121 - 140)	46.4 (44.3 - 48.5)	75.5 (70.1 - 81.2)
North Africa and Middle East	Female	5.29% (4.93% - 5.69%)	4.62% (4.32% - 4.94%)	2.54% (2.37% - 2.73%)	2.27% (2.1% - 2.54%)	16.5 (15.2 - 18)	18.9 (17.1 - 20.6)	7.53 (6.92 - 8.25)	9.92 (8.87 - 11.2)
North Africa and Middle East	Male	29.7% (29.2% - 30.3%)	29.3% (28.6% - 30%)	15.6% (15.3% - 16%)	15.4% (15.1% - 15.8%)	101 (96.3 - 105)	130 (121 - 140)	46.4 (44.3 - 48.5)	75.5 (70.1 - 81.2)
Afghanistan	Female	2.83% (2.27% - 3.62%)	3.54% (2.64% - 4.81%)	2.51% (2.14% - 2.96%)	2.35% (2% - 2.77%)	0.455 (0.281 - 0.67)	1.21 (0.724 - 1.84)	0.356 (0.236 - 0.482)	0.732 (0.477 - 1)
Afghanistan	Male	16.2% (14.9% - 17.6%)	19.7% (17% - 23.2%)	14.5% (13.5% - 15.6%)	14.7% (13.6% - 15.8%)	2.62 (1.73 - 3.44)	7.11 (4.5 - 9.78)	1.75 (1.2 - 2.31)	4.39 (2.87 - 5.89)
Algeria	Female	1.32% (1.02% - 1.72%)	1.08% (0.83% - 1.41%)	0.82% (0.7% - 0.96%)	0.69% (0.58% - 0.84%)	0.289 (0.212 - 0.393)	0.284 (0.204 - 0.384)	0.176 (0.138 - 0.217)	0.204 (0.155 - 0.259)
Algeria	Male	29.4% (27.8% - 31%)	30.3% (26.6% - 33%)	26.6% (25% - 28.3%)	28.5% (26.8% - 30.3%)	6.8 (5.79 - 7.86)	8.53 (6.83 - 10.2)	5.9 (4.87 - 6.82)	9.16 (7.49 - 10.8)
Bahrain	Female	5.4% (4.43% - 6.54%)	5.64% (4.43% - 7.04%)	2.09% (1.75% - 2.44%)	1.97% (1.65% - 2.31%)	0.0308 (0.0242 - 0.0384)	0.0469 (0.0358 - 0.0585)	0.012 (0.0099 - 0.0145)	0.0177 (0.0146 - 0.0211)
Bahrain	Male	23.9% (21.6% - 26%)	28.2% (23.4% - 31.5%)	15.8% (14.6% - 17%)	15.7% (14.5% - 16.9%)	0.237 (0.207 - 0.27)	0.361 (0.292 - 0.418)	0.152 (0.135 - 0.17)	0.274 (0.245 - 0.304)
Egypt	Female	1.38% (1.02% - 1.87%)	1.22% (0.89% - 1.68%)	0.57% (0.48% - 0.67%)	0.56% (0.47% - 0.65%)	0.758 (0.542 - 1.06)	0.948 (0.68 - 1.33)	0.258 (0.212 - 0.307)	0.444 (0.36 - 0.545)
Egypt	Male	40.6% (39.3% - 41.8%)	45% (43.7% - 46.3%)	12.2% (11.5% - 13.1%)	12.2% (11.4% - 13%)	22.2 (20.2 - 24.3)	37.6 (33.3 - 42.7)	6.03 (5.27 - 6.81)	10 (8.52 - 11.7)
Iran (Islamic Republic of)	Female	4.22% (3.41% - 5.19%)	4.63% (3.75% - 5.67%)	3.19% (2.71% - 3.8%)	3.02% (2.54% - 3.62%)	1.86 (1.46 - 2.36)	2.34 (1.82 - 2.96)	1.36 (1.1 - 1.63)	1.46 (1.19 - 1.75)
Iran (Islamic Republic of)	Male	24.1% (22.5% - 25.7%)	26.5% (24.6% - 28.3%)	13.8% (12.8% - 14.9%)	13.9% (12.9% - 15%)	11.4 (10 - 12.8)	13.5 (11.7 - 15.4)	5.78 (5.04 - 6.64)	8.65 (7.49 - 9.92)
Iraq	Female	3.15% (2.59% - 3.74%)	2.4% (1.97% - 2.87%)	4% (3.48% - 4.59%)	4.21% (3.63% - 4.95%)	0.583 (0.398 - 0.816)	0.758 (0.518 - 1.03)	0.672 (0.457 - 0.885)	1.35 (0.933 - 1.82)
Iraq	Male	33.4% (31.8% - 35.2%)	31.2% (27.8% - 34.3%)	17.3% (16.3% - 18.5%)	18% (17% - 19.2%)	7.28 (5.16 - 9.22)	10 (7.1 - 13.2)	2.82 (1.98 - 3.58)	5.79 (4.12 - 7.41)
Jordan	Female	11.6% (9.87% - 13.3%)	12.6% (10.8% - 14.5%)	3.36% (2.86% - 3.97%)	3.29% (2.74% - 4.01%)	0.706 (0.573 - 0.841)	1.38 (1.14 - 1.65)	0.17 (0.139 - 0.205)	0.38 (0.308 - 0.471)
Jordan	Male	49.6% (47.9% - 51.3%)	55.9% (52.2% - 58.2%)	15.6% (14.5% - 17%)	15.1% (14% - 16.4%)	3.44 (3.07 - 3.83)	6.77 (5.96 - 7.52)	0.88 (0.766 - 1)	1.91 (1.65 - 2.16)
Kuwait	Female	5.52% (4.58% - 6.57%)	5% (4.13% - 6%)	1.51% (1.29% - 1.79%)	1.58% (1.35% - 1.87%)	0.116 (0.0902 - 0.145)	0.138 (0.109 - 0.169)	0.0321 (0.0254 - 0.0403)	0.0589 (0.0493 - 0.071)
Kuwait	Male	33.1% (31.3% - 34.9%)	31.1% (29.2% - 33%)	16.4% (15.2% - 17.6%)	16.6% (15.4% - 17.8%)	0.899 (0.766 - 1.04)	1.02 (0.895 - 1.16)	0.372 (0.312 - 0.433)	0.745 (0.648 - 0.853)
Lebanon	Female	23.7% (21.6% - 26%)	26.3% (23.2% - 30.4%)	6.05% (5.26% - 6.88%)	6.12% (5.32% - 6.98%)	0.682 (0.566 - 0.812)	0.794 (0.626 - 1)	0.173 (0.138 - 0.211)	0.224 (0.175 - 0.278)
Lebanon	Male	42.2% (40.3% - 44.2%)	45.6% (43% - 49.2%)	10.9% (10.1% - 11.7%)	10.8% (10% - 11.7%)	1.19 (0.996 - 1.38)	1.38 (1.1 - 1.65)	0.286 (0.24 - 0.337)	0.385 (0.314 - 0.465)
Libya	Female	1.66% (1.27% - 2.15%)	1.84% (1.38% - 2.45%)	0.47% (0.39% - 0.55%)	0.47% (0.39% - 0.56%)	0.0549 (0.04 - 0.075)	0.0535 (0.0377 - 0.0736)	0.0153 (0.0117 - 0.0192)	0.0171 (0.0132 - 0.0219)
Libya	Male	35.4% (32.8% - 37.9%)	38.5% (34.4% - 41.6%)	10.3% (9.44% - 11.2%)	10.6% (9.72% - 11.6%)	1.35 (1.12 - 1.57)	1.38 (1.1 - 1.68)	0.343 (0.281 - 0.408)	0.479 (0.384 - 0.584)
Morocco	Female	1.21% (0.9% - 1.57%)	0.96% (0.7% - 1.25%)	0.67% (0.57% - 0.8%)	0.6% (0.49% - 0.73%)	0.222 (0.163 - 0.299)	0.16 (0.116 - 0.207)	0.127 (0.101 - 0.157)	0.118 (0.0914 - 0.15)
Morocco	Male	20% (18.9% - 21.2%)	15.6% (13.9% - 17.4%)	16.9% (15.9% - 18%)	17.8% (16.7% - 19%)	3.9 (3.39 - 4.43)	3.21 (2.67 - 3.82)	3.16 (2.73 - 3.53)	4.01 (3.41 - 4.61)
Oman	Female	2.04% (1.58% - 2.54%)	1.81% (1.39% - 2.31%)	2.37% (2.05% - 2.75%)	2.27% (1.95% - 2.67%)	0.0369 (0.0279 - 0.0481)	0.0553 (0.0413 - 0.0715)	0.0412 (0.0345 - 0.0493)	0.0692 (0.0575 - 0.083)
Oman	Male	14% (13% - 15.3%)	12.3% (9.47% - 14.4%)	11.3% (10.4% - 12.2%)	11.6% (10.6% - 12.5%)	0.483 (0.424 - 0.551)	0.516 (0.393 - 0.624)	0.276 (0.244 - 0.312)	0.59 (0.522 - 0.668)
Palestine	Female	3.53% (2.75% - 4.41%)	3.27% (2.55% - 4.21%)	1.04% (0.89% - 1.21%)	1.03% (0.88% - 1.2%)	0.0856 (0.066 - 0.11)	0.119 (0.089 - 0.158)	0.023 (0.0192 - 0.0276)	0.0376 (0.0302 - 0.0463)
Palestine	Male	36.4% (34.5% - 38.7%)	33.5% (31.2% - 36.3%)	10.9% (10.1% - 11.9%)	11.2% (10.4% - 12.2%)	0.928 (0.827 - 1.03)	1.24 (1.06 - 1.44)	0.238 (0.208 - 0.273)	0.42 (0.349 - 0.493)
Qatar	Female	4.08% (3.12% - 5.1%)	3.91% (2.99% - 4.89%)	1.17% (0.96% - 1.4%)	1.15% (0.94% - 1.38%)	0.0363 (0.0276 - 0.0453)	0.0779 (0.0593 - 0.0977)	0.0111 (0.0087 - 0.0138)	0.0285 (0.0235 - 0.0344)
Qatar	Male	23.2% (21.5% - 24.6%)	23.2% (21.4% - 24.7%)	11.9% (11.1% - 12.9%)	12% (11.1% - 13%)	0.551 (0.496 - 0.608)	1.01 (0.909 - 1.11)	0.202 (0.178 - 0.226)	0.661 (0.596 - 0.734)
Saudi Arabia	Female	2.44% (1.88% - 3.1%)	2.64% (1.98% - 3.37%)	1.05% (0.89% - 1.24%)	0.88% (0.74% - 1.05%)	0.379 (0.275 - 0.5)	0.498 (0.371 - 0.663)	0.166 (0.132 - 0.206)	0.188 (0.149 - 0.232)
Saudi Arabia	Male	21.5% (20% - 23%)	23.4% (20.8% - 26.9%)	9.13% (8.37% - 9.91%)	9.9% (9.1% - 10.7%)	5.47 (4.66 - 6.31)	5.87 (4.78 - 7.06)	1.87 (1.55 - 2.2)	3.08 (2.64 - 3.59)
Sudan	Female	2.09% (1.61% - 2.78%)	1.54% (1.17% - 2.03%)	0.97% (0.82% - 1.13%)	0.89% (0.75% - 1.05%)	0.492 (0.349 - 0.677)	0.505 (0.357 - 0.671)	0.179 (0.14 - 0.225)	0.294 (0.229 - 0.37)
Sudan	Male	16.7% (15.5% - 18.2%)	12.8% (11.8% - 14.1%)	14.4% (13.4% - 15.6%)	14.9% (13.8% - 16.1%)	3.71 (3.11 - 4.39)	4.49 (3.71 - 5.45)	2.83 (2.33 - 3.37)	4.96 (4.04 - 6.06)
Syrian Arab Republic	Female	5.66% (4.49% - 7.05%)	4.34% (3.42% - 5.44%)	2.12% (1.81% - 2.48%)	2.1% (1.79% - 2.46%)	0.427 (0.322 - 0.559)	0.362 (0.263 - 0.491)	0.153 (0.12 - 0.192)	0.195 (0.15 - 0.248)
Syrian Arab Republic	Male	36.2% (33.6% - 38.9%)	29.6% (27.1% - 32.2%)	13.4% (12.4% - 14.4%)	14.1% (13.1% - 15.2%)	2.36 (1.92 - 2.8)	2.29 (1.79 - 2.85)	0.911 (0.758 - 1.08)	1.27 (1.01 - 1.59)
Tunisia	Female	2.62% (2.08% - 3.24%)	2.15% (1.71% - 2.68%)	1.67% (1.42% - 1.96%)	1.68% (1.4% - 1.98%)	0.158 (0.123 - 0.2)	0.128 (0.0986 - 0.165)	0.103 (0.084 - 0.125)	0.115 (0.0927 - 0.143)
Tunisia	Male	40.9% (39.4% - 42.4%)	36% (34.4% - 37.7%)	13.2% (12.2% - 14.3%)	13.8% (12.7% - 15%)	2.48 (2.21 - 2.77)	2.17 (1.86 - 2.49)	0.811 (0.708 - 0.932)	1.02 (0.85 - 1.21)
Turkey	Female	17.8% (15.9% - 19.8%)	19% (17% - 21.1%)	6.26% (5.41% - 7.32%)	6.91% (5.57% - 8.64%)	7.36 (6.36 - 8.42)	6.29 (5.45 - 7.25)	2.64 (2.25 - 3.1)	2.81 (2.25 - 3.61)
Turkey	Male	39.5% (37.8% - 41.5%)	34.5% (31.8% - 37.4%)	20% (18.7% - 21.3%)	18.9% (17.5% - 20.4%)	17.4 (15.8 - 18.9)	13.2 (11.6 - 15)	8.36 (7.5 - 9.29)	10.4 (9.19 - 11.8)
United Arab Emirates	Female	3.94% (3.17% - 4.9%)	3.5% (2.61% - 4.74%)	2.03% (1.76% - 2.38%)	1.97% (1.7% - 2.31%)	0.118 (0.0892 - 0.159)	0.219 (0.157 - 0.304)	0.0542 (0.0415 - 0.0702)	0.135 (0.111 - 0.166)
United Arab Emirates	Male	17.4% (16.1% - 18.9%)	14.1% (11.8% - 17.2%)	15.2% (14.1% - 16.5%)	16.1% (14.8% - 17.4%)	1.37 (1.1 - 1.64)	1.7 (1.36 - 2.15)	0.995 (0.815 - 1.19)	2.62 (2.24 - 3)
Yemen	Female	8.13% (6.72% - 9.69%)	8.23% (6.79% - 9.87%)	2.39% (2.03% - 2.79%)	2.32% (1.97% - 2.73%)	1.3 (1 - 1.62)	2.28 (1.76 - 2.92)	0.377 (0.289 - 0.478)	0.625 (0.478 - 0.803)
Yemen	Male	28.4% (26.5% - 30.1%)	28.7% (26.6% - 30.9%)	13.9% (12.8% - 15%)	14.2% (13.2% - 15.4%)	4.54 (3.76 - 5.44)	8.15 (6.54 - 9.97)	1.91 (1.58 - 2.27)	3.63 (2.89 - 4.54)
South Asia	Female	3.03% (2.64% - 3.51%)	2.12% (1.83% - 2.45%)	1.57% (1.4% - 1.75%)	1.74% (1.52% - 1.97%)	26.5 (22.3 - 31.7)	25.3 (20.9 - 30.5)	13.4 (11.4 - 15.6)	20.7 (16.8 - 24.6)
South Asia	Male	21.3% (20.3% - 22.2%)	14.1% (13.4% - 14.9%)	8.47% (7.93% - 9.05%)	8.92% (8.37% - 9.55%)	196 (175 - 220)	168 (149 - 191)	72.8 (64.6 - 81.7)	107 (93.2 - 122)
South Asia	Female	3.03% (2.64% - 3.51%)	2.12% (1.83% - 2.45%)	1.57% (1.4% - 1.75%)	1.74% (1.52% - 1.97%)	26.5 (22.3 - 31.7)	25.3 (20.9 - 30.5)	13.4 (11.4 - 15.6)	20.7 (16.8 - 24.6)
South Asia	Male	21.3% (20.3% - 22.2%)	14.1% (13.4% - 14.9%)	8.47% (7.93% - 9.05%)	8.92% (8.37% - 9.55%)	196 (175 - 220)	168 (149 - 191)	72.8 (64.6 - 81.7)	107 (93.2 - 122)
Bangladesh	Female	3.11% (2.53% - 3.83%)	3.41% (2.66% - 4.3%)	4.94% (4.18% - 5.7%)	6.99% (5.73% - 8.32%)	2.53 (1.94 - 3.22)	3.52 (2.61 - 4.67)	3.94 (3.19 - 4.77)	7.32 (5.48 - 9.41)
Bangladesh	Male	41.9% (40.2% - 43.6%)	41.7% (38.9% - 43.9%)	24.8% (23.4% - 26.3%)	27.3% (25.5% - 29%)	33.9 (28.6 - 38.6)	39.3 (32.1 - 46.3)	19.4 (16.5 - 22.1)	27.4 (22 - 32.9)
Bhutan	Female	5.85% (4.71% - 7.12%)	5.73% (4.54% - 7.08%)	1.92% (1.62% - 2.28%)	2.11% (1.75% - 2.52%)	0.0215 (0.0171 - 0.0273)	0.0226 (0.0177 - 0.0292)	0.0066 (0.0055 - 0.008)	0.01 (0.0079 - 0.0127)
Bhutan	Male	14.3% (13% - 15.7%)	13.6% (11.8% - 15.4%)	26.3% (24.6% - 28%)	27% (25.3% - 28.8%)	0.0582 (0.0504 - 0.0662)	0.0477 (0.0391 - 0.0572)	0.0952 (0.0834 - 0.106)	0.138 (0.119 - 0.158)
India	Female	2.78% (2.3% - 3.36%)	1.87% (1.52% - 2.29%)	1.15% (0.98% - 1.35%)	1.14% (0.96% - 1.35%)	18.8 (15 - 23.5)	17.2 (13.6 - 22.4)	7.51 (6.15 - 9.18)	10.6 (8.24 - 13.1)
India	Male	19% (17.9% - 20%)	11.4% (10.6% - 12.1%)	6.86% (6.28% - 7.52%)	7.35% (6.74% - 8.02%)	136 (117 - 155)	105 (90.5 - 122)	45.8 (38.9 - 53.1)	67.8 (57.1 - 80.4)
Nepal	Female	11.5% (9.88% - 13.1%)	4.97% (3.93% - 6.94%)	5.59% (4.8% - 6.49%)	4.45% (3.76% - 5.31%)	1.67 (1.37 - 2.01)	1.1 (0.802 - 1.54)	0.856 (0.703 - 1.04)	0.845 (0.67 - 1.07)
Nepal	Male	26.9% (25.4% - 28.4%)	16.7% (15.3% - 18.1%)	14.1% (13% - 15.3%)	15.5% (14.3% - 16.9%)	3.76 (3.26 - 4.31)	2.93 (2.37 - 3.48)	1.98 (1.71 - 2.27)	2.56 (2.07 - 3.05)
Pakistan	Female	3.48% (2.83% - 4.31%)	2.24% (1.73% - 2.92%)	1.11% (0.94% - 1.3%)	1.14% (0.96% - 1.35%)	3.54 (2.79 - 4.57)	3.41 (2.5 - 4.55)	1.02 (0.85 - 1.22)	1.62 (1.3 - 2.01)
Pakistan	Male	21.7% (20.2% - 23.1%)	13.2% (11.7% - 15.4%)	4.76% (4.38% - 5.21%)	4.97% (4.55% - 5.43%)	22.6 (20.1 - 25.1)	20.9 (16.7 - 25.4)	4.91 (4.34 - 5.51)	7.51 (6.32 - 8.91)
Southeast Asia, East Asia, and Oceania	Female	3.63% (3.28% - 4.02%)	3.27% (2.91% - 3.67%)	2.02% (1.83% - 2.24%)	2.85% (2.56% - 3.18%)	43 (38 - 49)	41.9 (36.7 - 48.4)	22.5 (20.3 - 25.2)	34.9 (30.5 - 39.6)
Southeast Asia, East Asia, and Oceania	Male	43.9% (42.9% - 44.8%)	39% (38% - 39.9%)	9.56% (9.12% - 10%)	11.3% (10.8% - 11.9%)	526 (494 - 558)	444 (414 - 478)	112 (104 - 120)	148 (137 - 160)
East Asia	Female	3.15% (2.63% - 3.73%)	2.84% (2.38% - 3.36%)	1.92% (1.66% - 2.24%)	3.21% (2.77% - 3.77%)	27 (22.6 - 32.7)	25.9 (21.1 - 31.4)	14.4 (12.3 - 16.9)	23.6 (19.8 - 27.7)
East Asia	Male	43.5% (42.1% - 44.8%)	39.5% (36.6% - 41.5%)	7.78% (7.21% - 8.4%)	9.64% (8.89% - 10.5%)	365 (334 - 395)	286 (253 - 320)	64.7 (58.3 - 71.7)	84.4 (74.5 - 95.1)

Supplementary Results Table S1. Global all-age and age-standardised prevalence of current and former smokers by location, sex, and scenario, 2022 and 2050

Location	Sex	Current Smoking Prevalence 2022	Current Smoking Prevalence 2050	Former Smoking Prevalence 2022	Former Smoking Prevalence 2050	Current Smokers (millions) 2022	Current Smokers (millions) 2050	Former Smokers (millions) 2022	Former Smokers (millions) 2050
China	Female	3.11% (2.57% - 3.7%)	2.79% (2.31% - 3.33%)	1.94% (1.67% - 2.27%)	3.25% (2.8% - 3.84%)	25.9 (21.5 - 31.5)	25 (20.3 - 30.6)	14.1 (11.9 - 16.5)	23.1 (19.3 - 27.3)
China	Male	43.7% (42.3% - 45.1%)	39.8% (36.7% - 41.8%)	7.83% (7.25% - 8.47%)	9.7% (8.93% - 10.6%)	355 (324 - 386)	279 (245 - 312)	63 (56.4 - 69.9)	82.3 (72.2 - 93.1)
Democratic People's Republic of Korea	Female	4.49% (3.59% - 5.63%)	4.64% (3.72% - 5.82%)	1.42% (1.2% - 1.65%)	1.98% (1.59% - 2.37%)	0.631 (0.479 - 0.825)	0.613 (0.46 - 0.811)	0.195 (0.153 - 0.241)	0.265 (0.202 - 0.335)
Democratic People's Republic of Korea	Male	38.3% (36.3% - 40.3%)	32.9% (30.8% - 35%)	6.27% (5.72% - 6.83%)	7.83% (7.14% - 8.56%)	5.56 (4.7 - 6.46)	4.37 (3.6 - 5.14)	0.818 (0.675 - 0.977)	1.12 (0.91 - 1.33)
Taiwan (Province of China)	Female	4.52% (3.71% - 5.59%)	3.18% (2.57% - 3.92%)	1.51% (1.27% - 1.78%)	2.19% (1.82% - 2.56%)	0.497 (0.394 - 0.624)	0.237 (0.187 - 0.3)	0.185 (0.154 - 0.223)	0.25 (0.204 - 0.304)
Taiwan (Province of China)	Male	35.4% (33.5% - 37.4%)	30.4% (28.1% - 32.8%)	6.36% (5.79% - 6.93%)	7.7% (6.95% - 8.4%)	4.51 (4.05 - 5.01)	3.16 (2.8 - 3.55)	0.834 (0.723 - 0.947)	0.989 (0.854 - 1.14)
Oceania	Female	18.8% (17.3% - 20.3%)	15.8% (14.2% - 17.9%)	8.74% (7.81% - 9.74%)	7.93% (6.94% - 8.99%)	1.36 (1.2 - 1.54)	2.08 (1.77 - 2.46)	0.589 (0.503 - 0.679)	1.01 (0.836 - 1.21)
Oceania	Male	45.1% (43.8% - 46.4%)	44.9% (43% - 46.8%)	14.4% (13.7% - 15.2%)	13.1% (12.2% - 13.9%)	3.49 (3.09 - 3.88)	6.41 (5.64 - 7.3)	0.923 (0.825 - 1.02)	1.62 (1.41 - 1.85)
American Samoa	Female	21.8% (18% - 26.2%)	20.6% (17.1% - 24.9%)	9.11% (7.55% - 10.7%)	8.87% (7.33% - 10.4%)	0.0054 (0.0044 - 0.0065)	0.0061 (0.005 - 0.0075)	0.0022 (0.0018 - 0.0027)	0.0027 (0.0022 - 0.0033)
American Samoa	Male	39.4% (36.4% - 42.3%)	38.8% (35.8% - 41.7%)	18.2% (17% - 19.5%)	18.4% (17% - 19.6%)	0.0101 (0.009 - 0.0112)	0.0122 (0.0108 - 0.0137)	0.0045 (0.004 - 0.0049)	0.0058 (0.0051 - 0.0066)
Cook Islands	Female	22.5% (20.2% - 24.8%)	14.6% (12.4% - 18.3%)	13.3% (11.5% - 15.2%)	11.7% (10% - 13.7%)	0.002 (0.0017 - 0.0023)	0.0012 (0.001 - 0.0016)	0.0012 (0.001 - 0.0015)	0.0011 (0.0009 - 0.0013)
Cook Islands	Male	34.1% (32.2% - 36.1%)	29.7% (27.7% - 31.6%)	19.8% (18.4% - 21.4%)	20.2% (18.8% - 21.7%)	0.0028 (0.0025 - 0.0031)	0.0024 (0.0021 - 0.0027)	0.0019 (0.0017 - 0.0021)	0.0019 (0.0016 - 0.0021)
Fiji	Female	14.8% (12.3% - 17.3%)	14.1% (11.3% - 16.7%)	16% (14.1% - 18.3%)	15.8% (13.8% - 18%)	0.0682 (0.0545 - 0.0824)	0.0591 (0.0454 - 0.0753)	0.074 (0.0631 - 0.0856)	0.0694 (0.0564 - 0.0841)
Fiji	Male	34.5% (32.1% - 36.8%)	29.2% (26.5% - 32%)	26.2% (24.4% - 28.1%)	27.1% (25.2% - 29.1%)	0.165 (0.146 - 0.186)	0.125 (0.105 - 0.15)	0.119 (0.105 - 0.135)	0.144 (0.12 - 0.176)
Guam	Female	19.4% (17.6% - 21.6%)	17.2% (15.2% - 19.4%)	11.8% (10.5% - 13.2%)	12.3% (11% - 13.8%)	0.0148 (0.0131 - 0.017)	0.0104 (0.0084 - 0.0127)	0.0093 (0.0081 - 0.0104)	0.0092 (0.0076 - 0.0108)
Guam	Male	28.6% (27.2% - 30.2%)	24.2% (22.6% - 26.3%)	22.4% (21.1% - 23.6%)	23.4% (22.1% - 24.7%)	0.0229 (0.0207 - 0.0252)	0.0152 (0.0127 - 0.0179)	0.0186 (0.0169 - 0.0204)	0.0227 (0.0189 - 0.0264)
Kiribati	Female	32.9% (30.2% - 35.6%)	31.3% (27.7% - 36.5%)	10.4% (8.82% - 11.8%)	8.6% (7.19% - 10.2%)	0.0204 (0.0179 - 0.0231)	0.0233 (0.0188 - 0.0292)	0.0064 (0.0054 - 0.0078)	0.0063 (0.005 - 0.008)
Kiribati	Male	59.3% (57.6% - 60.9%)	60.1% (57.1% - 64.4%)	16.4% (15.3% - 17.7%)	16.1% (15% - 17.4%)	0.0355 (0.0316 - 0.0398)	0.0434 (0.036 - 0.0513)	0.0086 (0.0074 - 0.0097)	0.0107 (0.009 - 0.0128)
Marshall Islands	Female	10.8% (8.92% - 13.2%)	13.5% (11.1% - 16.3%)	6.57% (5.58% - 7.75%)	6.83% (5.77% - 8.06%)	0.0032 (0.0025 - 0.0041)	0.0049 (0.0037 - 0.0062)	0.0018 (0.0014 - 0.0022)	0.0025 (0.0019 - 0.0031)
Marshall Islands	Male	34.3% (31.9% - 36.9%)	36.6% (34.1% - 39.2%)	18.8% (17.4% - 20.2%)	18.9% (17.5% - 20.3%)	0.0106 (0.0088 - 0.0122)	0.0137 (0.0114 - 0.0161)	0.0049 (0.0042 - 0.0057)	0.0069 (0.0057 - 0.0083)
Micronesia (Federated States of)	Female	35.2% (30.8% - 40%)	38.1% (33.5% - 43%)	13.4% (11.4% - 15.6%)	13.8% (11.7% - 16%)	0.0182 (0.0149 - 0.0216)	0.0191 (0.0143 - 0.0242)	0.0068 (0.0056 - 0.0083)	0.0071 (0.0053 - 0.0091)
Micronesia (Federated States of)	Male	60.7% (58.1% - 63.2%)	63.2% (60.6% - 65.6%)	16.6% (15.3% - 17.8%)	16.7% (15.4% - 17.8%)	0.0325 (0.0283 - 0.0374)	0.0319 (0.0247 - 0.0399)	0.0077 (0.0066 - 0.009)	0.0094 (0.0072 - 0.0119)
Nauru	Female	37.6% (33.6% - 41.6%)	31.8% (27.4% - 37.7%)	16.4% (14.2% - 18.7%)	14.7% (12.4% - 17.5%)	0.0022 (0.0018 - 0.0026)	0.0027 (0.0022 - 0.0033)	0.001 (0.0008 - 0.0012)	0.0012 (0.001 - 0.0015)
Nauru	Male	41.2% (39.2% - 43.4%)	39.2% (36.9% - 41.7%)	18.2% (17% - 19.6%)	18.4% (17.1% - 19.7%)	0.0024 (0.0021 - 0.0028)	0.0034 (0.0028 - 0.0041)	0.0008 (0.0007 - 0.001)	0.0014 (0.0011 - 0.0017)
Niue	Female	14.4% (12% - 17.1%)	14.1% (11.7% - 16.8%)	8.51% (7.2% - 9.89%)	8.55% (7.23% - 9.96%)	0.0001 (0.0001 - 0.0001)	0.0001 (0.0001 - 0.0001)	0.0001 (0.0001 - 0.0001)	0.0001 (0.0001 - 0.0001)
Niue	Male	25.7% (23.6% - 27.6%)	25% (22.8% - 27%)	19.2% (17.8% - 20.7%)	19.4% (17.9% - 20.9%)	0.0002 (0.0002 - 0.0002)	0.0002 (0.0002 - 0.0002)	0.0002 (0.0001 - 0.0002)	0.0002 (0.0001 - 0.0002)
Northern Mariana Islands	Female	17.2% (13.8% - 20.9%)	14.2% (11.3% - 17.4%)	8.05% (6.94% - 9.42%)	8.04% (6.92% - 9.4%)	0.004 (0.0032 - 0.005)	0.0031 (0.0025 - 0.004)	0.0019 (0.0016 - 0.0023)	0.002 (0.0017 - 0.0024)
Northern Mariana Islands	Male	37.6% (34.6% - 40.3%)	33.1% (30.3% - 35.9%)	18.3% (16.9% - 19.8%)	18.6% (17.2% - 20%)	0.0098 (0.0088 - 0.0109)	0.0081 (0.0071 - 0.0092)	0.0049 (0.0043 - 0.0055)	0.0053 (0.0047 - 0.006)
Palau	Female	13.4% (11.8% - 15.1%)	12.7% (11.2% - 14.3%)	10.5% (9.03% - 12.1%)	10.4% (8.93% - 12%)	0.001 (0.0009 - 0.0012)	0.0007 (0.0006 - 0.0009)	0.001 (0.0008 - 0.0011)	0.0008 (0.0007 - 0.001)
Palau	Male	31.6% (29.7% - 33.4%)	31.6% (29.6% - 33.4%)	19.4% (18.1% - 20.7%)	19.5% (18.2% - 20.8%)	0.003 (0.0026 - 0.0034)	0.0024 (0.0021 - 0.0027)	0.002 (0.0017 - 0.0023)	0.002 (0.0017 - 0.0023)
Papua New Guinea	Female	19.4% (17.4% - 21.3%)	15.6% (13.8% - 18.1%)	8.21% (7.05% - 9.45%)	7.78% (6.65% - 8.97%)	1.06 (0.899 - 1.23)	1.7 (1.4 - 2.05)	0.41 (0.333 - 0.492)	0.81 (0.648 - 0.988)
Papua New Guinea	Male	46.4% (44.7% - 48.1%)	45.5% (43.4% - 47.6%)	12.4% (11.5% - 13.3%)	11.8% (10.8% - 12.8%)	2.76 (2.36 - 3.13)	5.4 (4.65 - 6.23)	0.587 (0.507 - 0.675)	1.19 (1 - 1.38)
Samoa	Female	13.9% (12% - 15.7%)	11.8% (9.73% - 14%)	3.93% (3.32% - 4.61%)	3.67% (3.1% - 4.31%)	0.0147 (0.0123 - 0.0175)	0.0177 (0.0138 - 0.0224)	0.0041 (0.0034 - 0.0049)	0.0056 (0.0044 - 0.007)
Samoa	Male	35.1% (33.4% - 37%)	30% (27.9% - 33.3%)	8.23% (7.56% - 8.93%)	8.2% (7.53% - 8.9%)	0.0383 (0.0341 - 0.0432)	0.0477 (0.0399 - 0.0565)	0.0084 (0.0074 - 0.0096)	0.0139 (0.0116 - 0.0165)
Solomon Islands	Female	19.4% (16.8% - 22.1%)	21.9% (18.8% - 24.9%)	8.09% (6.86% - 9.57%)	8.24% (6.98% - 9.78%)	0.0685 (0.0556 - 0.0818)	0.112 (0.0893 - 0.14)	0.0268 (0.0212 - 0.0332)	0.0402 (0.0311 - 0.0511)
Solomon Islands	Male	48.7% (46.6% - 50.7%)	53.6% (50% - 56.2%)	17.2% (15.9% - 18.6%)	17% (15.8% - 18.4%)	0.17 (0.145 - 0.196)	0.279 (0.226 - 0.34)	0.0523 (0.0429 - 0.0609)	0.0759 (0.0612 - 0.0934)
Tokelau	Female	23.3% (19.6% - 27.6%)	24.2% (20.4% - 28.6%)	8.09% (6.84% - 9.33%)	8.11% (6.86% - 9.36%)	0.0002 (0.0001 - 0.0002)	0.0002 (0.0001 - 0.0002)	0.0001 (0 - 0.0001)	0.0001 (0 - 0.0001)
Tokelau	Male	40.6% (37.8% - 43.8%)	39.9% (37.1% - 43.2%)	18.4% (16.9% - 19.8%)	18.5% (17.1% - 20%)	0.0003 (0.0003 - 0.0003)	0.0003 (0.0003 - 0.0003)	0.0001 (0.0001 - 0.0001)	0.0002 (0.0001 - 0.0002)
Tonga	Female	13.1% (11.2% - 15.4%)	12.5% (10.6% - 14.6%)	5.48% (4.6% - 6.41%)	5.35% (4.5% - 6.27%)	0.007 (0.0057 - 0.0086)	0.008 (0.0062 - 0.0102)	0.0029 (0.0024 - 0.0035)	0.0037 (0.0029 - 0.0047)
Tonga	Male	39.4% (37.1% - 41.7%)	32.7% (30.6% - 35%)	14.3% (13.1% - 15.5%)	15.6% (14.3% - 16.9%)	0.0202 (0.0178 - 0.0227)	0.0225 (0.0184 - 0.0268)	0.0073 (0.0063 - 0.0083)	0.0118 (0.0095 - 0.0145)
Tuvalu	Female	20.4% (17.4% - 23.7%)	23.1% (19.8% - 26.4%)	9.18% (7.8% - 10.8%)	9.39% (7.98% - 11%)	0.0012 (0.001 - 0.0015)	0.0019 (0.0015 - 0.0023)	0.0006 (0.0004 - 0.0007)	0.0008 (0.0006 - 0.0009)
Tuvalu	Male	43.6% (41.4% - 46.1%)	45.2% (43% - 47.6%)	17.6% (16.3% - 18.8%)	17.6% (16.3% - 18.8%)	0.0029 (0.0025 - 0.0033)	0.004 (0.0033 - 0.0046)	0.0011 (0.0009 - 0.0012)	0.0015 (0.0013 - 0.0018)
Vanuatu	Female	6.38% (5.34% - 7.62%)	4.9% (3.97% - 6.15%)	2.74% (2.32% - 3.16%)	2.69% (2.29% - 3.09%)	0.0114 (0.0095 - 0.0139)	0.0126 (0.0099 - 0.0163)	0.0038 (0.0032 - 0.0045)	0.0064 (0.0053 - 0.0076)
Vanuatu	Male	33.6% (31.4% - 35.8%)	26% (22.9% - 30.9%)	24.2% (22.6% - 25.9%)	24.8% (23.1% - 26.5%)	0.054 (0.0488 - 0.0592)	0.0678 (0.0578 - 0.0814)	0.0363 (0.0325 - 0.0403)	0.0593 (0.052 - 0.0675)
Southeast Asia	Female	4.18% (3.84% - 4.55%)	3.2% (2.76% - 3.88%)	2.22% (2.05% - 2.4%)	2.22% (1.99% - 2.47%)	14.9 (13.5 - 16.5)	14.1 (12 - 17.4)	7.69 (7.01 - 8.4)	10 (8.83 - 11.3)
Southeast Asia	Male	43.2% (42.3% - 44%)	36.6% (34.4% - 39.9%)	14.4% (13.9% - 15%)	14.1% (13.5% - 14.8%)	156 (149 - 163)	151 (137 - 168)	48.7 (45.8 - 51.6)	63.1 (58 - 68.5)
Cambodia	Female	5.31% (4.39% - 6.33%)	5.09% (4.01% - 6.63%)	1.35% (1.13% - 1.59%)	1.41% (1.18% - 1.67%)	0.455 (0.356 - 0.576)	0.591 (0.432 - 0.79)	0.115 (0.0896 - 0.146)	0.162 (0.126 - 0.207)
Cambodia	Male	35.4% (33.9% - 36.8%)	28.7% (26.8% - 32.5%)	10% (9.23% - 10.9%)	11.1% (10.2% - 12%)	2.76 (2.29 - 3.2)	3.32 (2.67 - 3.97)	0.772 (0.636 - 0.917)	1.22 (1 - 1.47)
Indonesia	Female	3.33% (2.79% - 4.06%)	3% (2.19% - 4.21%)	0.98% (0.84% - 1.14%)	0.79% (0.67% - 0.95%)	4.69 (3.78 - 5.84)	5.26 (3.71 - 7.42)	1.32 (1.11 - 1.56)	1.4 (1.14 - 1.69)
Indonesia	Male	52.6% (51.1% - 54.1%)	49.3% (44% - 56.3%)	7.55% (6.99% - 8.13%)	6.86% (6.29% - 7.45%)	76.9 (70.1 - 82.6)	81.5 (68.8 - 96.9)	9.93 (8.84 - 11)	12.6 (10.9 - 14.4)
Lao People's Democratic Republic	Female	6.5% (5.47% - 7.85%)	8.15% (6.63% - 9.84%)	1.97% (1.66% - 2.32%)	1.9% (1.59% - 2.25%)	0.232 (0.187 - 0.288)	0.414 (0.321 - 0.515)	0.0683 (0.0549 - 0.0836)	0.095 (0.0747 - 0.117)
Lao People's Democratic Republic	Male	44.6% (42.6% - 46.6%)	49.7% (45.5% - 52.5%)	12% (11.1% - 13%)	11.3% (10.4% - 12.4%)	1.63 (1.45 - 1.81)	2.48 (2.14 - 2.82)	0.403 (0.351 - 0.45)	0.556 (0.477 - 0.633)
Malaysia	Female	3.14% (2.55% - 3.83%)	2.22% (1.72% - 2.84%)	1.12% (0.95% - 1.34%)	1.04% (0.87% - 1.24%)	0.479 (0.371 - 0.609)	0.42 (0.318 - 0.554)	0.171 (0.136 - 0.209)	0.209 (0.166 - 0.257)
Malaysia	Male	37.1% (34.9% - 39.2%)	28.3% (25.8% - 31.7%)	9.89% (9.08% - 10.8%)	9.13% (8.34% - 10.1%)	6.4 (5.47 - 7.33)	5.59 (4.68 - 6.61)	1.58 (1.34 - 1.83)	1.96 (1.64 - 2.3)
Maldives	Female	7.52% (6.27% - 8.91%)	8.19% (6.44% - 9.92%)	1.62% (1.37% - 1.95%)	1.41% (1.16% - 1.76%)	0.015 (0.0121 - 0.0183)	0.0293 (0.0225 - 0.0363)	0.0031 (0.0025 - 0.0038)	0.0054 (0.0043 - 0.0068)
Maldives	Male	41.4% (39.7% - 43.2%)	45.1% (38.6% - 49.6%)	13% (12% - 14.1%)	12.5% (11.5% - 13.7%)	0.142 (0.125 - 0.16)	0.221 (0.182 - 0.255)	0.0374 (0.0324 - 0.043)	0.0678 (0.0592 - 0.0771)
Mauritius	Female	6.11% (5.07% - 7.36%)	6.6% (5.04% - 8.25%)	3.27% (2.79% - 3.85%)	3.04% (2.55% - 3.58%)	0.0357 (0.0285 - 0.0446)	0.0298 (0.0218 - 0.0384)	0.021 (0.0172 - 0.0254)	0.0177 (0.0141 - 0.0215)
Mauritius	Male	40.2% (38.1% - 42.5%)	42.6% (35.6% - 47.6%)	13.2% (12.1% - 14.3%)	13.1% (12% - 14.3%)	0.258 (0.222 - 0.293)	0.221 (0.172 - 0.267)	0.0875 (0.0739 - 0.103)	0.0898 (0.0751 - 0.107)
Myanmar	Female	7.26% (6.16% - 8.53%)	4.25% (3.53% - 5.05%)	3.06% (2.55% - 3.59%)	2.61% (2.13% - 3.12%)	2.16 (1.79 - 2.63)	1.81 (1.44 - 2.31)	0.896 (0.73 - 1.08)	1.05 (0.832 - 1.34)
Myanmar	Male	36.3% (34.6% - 38.2%)	28.4% (24.1% - 31.6%)	14.9% (13.8% - 15.8%)	15.2% (14.1% - 16.3%)	10 (8.86 - 11.2)	9.01 (7.24 - 10.8)	3.87 (3.37 - 4.41)	4.99 (4.27 - 5.76)
Philippines	Female	7.62% (6.65% - 8.71%)	4.43% (3.64% - 5.64%)	6.41% (5.56% - 7.23%)	6.18% (5.24% - 7.25%)	4.19 (3.5 - 4.97)	3.45 (2.75 - 4.46)	3.49 (2.89 - 4.08)	4.82 (3.9 - 5.9)
Philippines	Male	36% (34.4% - 37.3%)	23.7% (22.2% - 25.8%)	20.6% (19.5% - 21.7%)	21% (19.8% - 22.1%)	21.1 (18.6 - 23.4)	18.5 (15.9 - 21)	10.9 (9.55 - 12.2)	16.5 (14.3 - 18.9)
Seychelles	Female	7.35% (5.93% - 8.9%)	6.51% (5.11% - 7.95%)	2.88% (2.41% - 3.37%)	2.68% (2.25% - 3.14%)	0.0035 (0.0027 - 0.0044)	0.004 (0.0031 - 0.0051)	0.0015 (0.0012 - 0.0019)	0.0019 (0.0015 - 0.0023)
Seychelles	Male	36.4% (33.6% - 39.2%)	33.2% (30.3% - 35.9%)	16% (14.8% - 17.4%)	15.4% (14.1% - 16.7%)	0.021 (0.0178 - 0.0246)	0.0239 (0.0204 - 0.028)	0.0091 (0.0076 - 0.0107)	0.0118 (0.01 - 0.0138)
Sri Lanka	Female	1.8% (1.36% - 2.22%)	1.37% (1% - 1.77%)	2.04% (1.73% - 2.37%)	1.98% (1.68% - 2.32%)	0.212 (0.157 - 0.27)	0.156 (0.109 - 0.211)	0.249 (0.203 - 0.302)	0.254 (0.199 - 0.32)
Sri Lanka	Male	26.3% (24.6% - 28.1%)	22.5% (17.5% - 26.6%)	18.9% (17.7% - 20.3%)	19.5% (18.1% - 21%)	2.88 (2.46 - 3.3)	2.36 (1.72 - 3.03)	2.12 (1.81 - 2.41)	2.28 (1.86 - 2.74)
Thailand	Female	3.25% (2.7% - 3.91%)	2.09% (1.72% - 2.53%)	2.12% (1.8% - 2.43%)	1.97% (1.66% - 2.29%)	1.2 (0.917 - 1.53)	0.695 (0.522 - 0.884)	0.834 (0.659 - 1.01)	0.954 (0.741 - 1.19)
Thailand	Male	36.2% (34.6% - 37.8%)	30% (28.1% - 31.6%)	19.8% (18.6% - 21.1%)	19.1% (17.8% - 20.9%)	12.1 (10.3 - 13.9)	8.08 (6.74 - 9.48)	7.05 (5.89 - 8.17)	7.24 (5.94 - 8.58)
Timor-Leste	Female	5.73% (4.7% - 6.87%)	5.61% (4.6% - 6.75%)	1.84% (1.57% - 2.16%)	1.77% (1.51% - 2.1%)	0.0397 (0.0312 - 0.0505)	0.0559 (0.0425 - 0.0726)	0.0118 (0.0095 - 0.0142)	0.0174 (0.0134 - 0.0219)
Timor-Leste	Male	61.2% (59.1% - 63.2%)	61.4% (59.2% - 63.5%)	6.32% (5.75% - 6.88%)	5.97% (5.43% - 6.53%)	0.429 (0.382 - 0.483)	0.639 (0.528 - 0.762)	0.0407 (0.0352 - 0.0471)	0.0576 (0.046 - 0.0703)
Viet Nam	Female	2.38% (1.93% - 2.85%)	1.85% (1.46% - 2.31%)	1.23% (1.06% - 1.42%)	1.19% (1.03% - 1.39%)	1.29 (1.02 - 1.59)	1.22 (0.951 - 1.53)	0.618 (0.516 - 0.733)	0.934 (0.761 - 1.13)
Viet Nam	Male	40.9% (39.6% - 42.4%)	33.7% (31.8% - 36%)	21.8% (20.5% - 23.1%)	23% (21.5% - 24.5%)	22.2 (20.1 - 24.2)	19.8 (17.4 - 22.4)	10.7 (9.52 - 12)	14.5 (12.7 - 16.5)
Sub-Saharan Africa	Female	2.88% (2.73% - 3.06%)	2.09% (1.98% - 2.23%)	4.11% (3.78% - 4.48%)	4.22% (3.85% - 4.6%)	16.5 (15.3 - 18)	22 (20.1 - 24.4)	23.2 (20.8 - 25.7)	44.4 (39.2 - 49.8)

Supplementary Results Table S1. Global all-age and age-standardised prevalence of current and former smokers by location, sex, and scenario, 2022 and 2050

Location	Sex	Current Smoking Prevalence 2022	Current Smoking Prevalence 2050	Former Smoking Prevalence 2022	Former Smoking Prevalence 2050	Current Smokers (millions) 2022	Current Smokers (millions) 2050	Former Smokers (millions) 2022	Former Smokers (millions) 2050
Sub-Saharan Africa	Male	15.2% (14.8% - 15.6%)	12.4% (11.9% - 12.8%)	11.5% (11.1% - 11.8%)	11.5% (11.1% - 11.8%)	79.5 (76 - 83.6)	128 (116 - 139)	55.9 (53 - 58.9)	111 (101 - 121)
Central Sub-Saharan Africa	Female	2.07% (1.72% - 2.45%)	2.23% (1.75% - 2.72%)	2.51% (2.25% - 2.82%)	2.28% (2.04% - 2.56%)	1.49 (1.1 - 2)	2.8 (1.98 - 3.76)	1.64 (1.29 - 2.01)	2.76 (2.15 - 3.42)
Central Sub-Saharan Africa	Male	19.1% (18.1% - 20.1%)	19.8% (17% - 21.7%)	11.6% (11% - 12.3%)	10.8% (10.2% - 11.5%)	12.1 (9.61 - 14.6)	24.7 (18.2 - 30.5)	6.66 (5.27 - 8.16)	12 (9.42 - 14.8)
Angola	Female	3.36% (2.6% - 4.31%)	3.77% (2.7% - 4.89%)	2.46% (2.09% - 2.88%)	2.22% (1.88% - 2.63%)	0.59 (0.421 - 0.837)	1.27 (0.882 - 1.76)	0.395 (0.322 - 0.487)	0.727 (0.586 - 0.9)
Angola	Male	18.3% (16.9% - 20.1%)	20% (15.9% - 22.9%)	11.8% (10.9% - 12.7%)	11% (10.1% - 11.9%)	2.64 (2.27 - 3.04)	6.46 (4.94 - 7.86)	1.59 (1.38 - 1.83)	3.17 (2.68 - 3.73)
Central African Republic	Female	1.82% (1.34% - 2.33%)	1.7% (1.2% - 2.3%)	2.52% (2.14% - 2.97%)	2.29% (1.94% - 2.71%)	0.0537 (0.0364 - 0.0752)	0.0622 (0.0381 - 0.0915)	0.0662 (0.0498 - 0.0844)	0.0811 (0.0571 - 0.108)
Central African Republic	Male	14.1% (12.5% - 15.9%)	13% (10.1% - 15.6%)	12% (11.2% - 13%)	11.3% (10.4% - 12.3%)	0.36 (0.282 - 0.439)	0.447 (0.292 - 0.62)	0.27 (0.217 - 0.323)	0.337 (0.242 - 0.442)
Congo	Female	2.53% (1.78% - 3.52%)	2.94% (2.08% - 4.05%)	2.37% (2.01% - 2.79%)	2.09% (1.77% - 2.47%)	0.0787 (0.052 - 0.118)	0.112 (0.075 - 0.16)	0.0619 (0.0481 - 0.0766)	0.0822 (0.0624 - 0.105)
Congo	Male	21.3% (19.5% - 23.2%)	26.6% (24.2% - 29.1%)	11.7% (10.6% - 12.7%)	10.6% (9.69% - 11.5%)	0.573 (0.47 - 0.681)	1.07 (0.872 - 1.3)	0.279 (0.229 - 0.329)	0.401 (0.322 - 0.479)
Democratic Republic of the Congo	Female	1.56% (1.16% - 2.07%)	1.6% (1.16% - 2.18%)	2.54% (2.15% - 2.99%)	2.31% (1.97% - 2.73%)	0.715 (0.425 - 1.16)	1.29 (0.785 - 1.99)	1.08 (0.723 - 1.47)	1.8 (1.2 - 2.45)
Democratic Republic of the Congo	Male	19.3% (17.9% - 20.7%)	19.4% (16.6% - 21.6%)	11.6% (10.7% - 12.5%)	10.8% (9.92% - 11.7%)	8.07 (5.54 - 10.5)	15.8 (10.3 - 21.2)	4.36 (3.03 - 5.84)	7.82 (5.55 - 10.6)
Equatorial Guinea	Female	2.74% (2.02% - 3.58%)	3.03% (2.23% - 4.06%)	2.39% (2.01% - 2.79%)	2.14% (1.79% - 2.5%)	0.022 (0.0154 - 0.0304)	0.046 (0.0329 - 0.0641)	0.0156 (0.0126 - 0.019)	0.0321 (0.0254 - 0.0405)
Equatorial Guinea	Male	25.9% (23.9% - 27.9%)	28.6% (25.4% - 31.3%)	11.3% (10.4% - 12.2%)	10.4% (9.58% - 11.2%)	0.21 (0.183 - 0.241)	0.506 (0.422 - 0.595)	0.0684 (0.0589 - 0.0777)	0.157 (0.133 - 0.182)
Gabon	Female	3.78% (3.02% - 4.78%)	4.36% (3.46% - 5.53%)	2.49% (2.1% - 2.89%)	2.24% (1.88% - 2.63%)	0.0386 (0.0302 - 0.0511)	0.0621 (0.0473 - 0.081)	0.0228 (0.0182 - 0.0279)	0.0334 (0.0266 - 0.041)
Gabon	Male	20.7% (19.3% - 22.3%)	23.6% (21.9% - 25.6%)	11.8% (10.9% - 12.8%)	10.9% (10% - 11.9%)	0.189 (0.163 - 0.215)	0.339 (0.283 - 0.403)	0.0923 (0.0796 - 0.106)	0.145 (0.12 - 0.172)
Eastern Sub-Saharan Africa	Female	3.16% (2.95% - 3.39%)	2.51% (2.26% - 2.79%)	8.22% (7.39% - 9.1%)	8.53% (7.64% - 9.46%)	6.52 (5.9 - 7.18)	9.73 (8.48 - 11.1)	17.3 (15.2 - 19.5)	33.6 (29.3 - 38.5)
Eastern Sub-Saharan Africa	Male	15.5% (15% - 16%)	12.8% (12.2% - 13.4%)	15.8% (15% - 16.6%)	16% (15.1% - 16.8%)	29.2 (27.6 - 31)	49.2 (44 - 54.6)	29.5 (27.3 - 31.6)	59.4 (53.2 - 66.5)
Burundi	Female	3.94% (3.14% - 4.93%)	2.66% (2.08% - 3.35%)	2.88% (2.39% - 3.47%)	3.03% (2.51% - 3.63%)	0.246 (0.183 - 0.329)	0.335 (0.248 - 0.441)	0.188 (0.148 - 0.235)	0.38 (0.294 - 0.479)
Burundi	Male	14.5% (13.4% - 15.8%)	11.9% (8.11% - 14.9%)	17.3% (16.1% - 18.4%)	17.8% (16.6% - 19.1%)	0.862 (0.705 - 1.01)	1.39 (0.878 - 1.87)	1.01 (0.834 - 1.16)	1.99 (1.62 - 2.42)
Comoros	Female	3.19% (2.56% - 4.08%)	2.86% (2.28% - 3.65%)	2.57% (2.19% - 2.98%)	2.6% (2.23% - 3.03%)	0.0124 (0.0092 - 0.0166)	0.0122 (0.009 - 0.0165)	0.0092 (0.0071 - 0.0116)	0.0121 (0.0091 - 0.0161)
Comoros	Male	21.5% (19.9% - 23.2%)	18.7% (17.1% - 20.4%)	16.8% (15.6% - 18%)	16.8% (15.7% - 18.1%)	0.0784 (0.0646 - 0.094)	0.086 (0.0671 - 0.11)	0.0573 (0.0469 - 0.0697)	0.0804 (0.0629 - 0.101)
Djibouti	Female	5.6% (4.51% - 6.82%)	5.97% (4.75% - 7.34%)	3.14% (2.69% - 3.65%)	3.16% (2.7% - 3.65%)	0.0325 (0.024 - 0.0428)	0.0571 (0.0425 - 0.0735)	0.017 (0.0134 - 0.0207)	0.0308 (0.0244 - 0.0378)
Djibouti	Male	37.1% (34.6% - 39.3%)	38.6% (35.9% - 41%)	10.5% (9.67% - 11.4%)	10.4% (9.58% - 11.4%)	0.246 (0.208 - 0.288)	0.425 (0.356 - 0.499)	0.0663 (0.0552 - 0.0782)	0.107 (0.0895 - 0.127)
Eritrea	Female	0.74% (0.56% - 0.98%)	0.65% (0.47% - 0.88%)	0.74% (0.61% - 0.89%)	0.74% (0.61% - 0.89%)	0.0256 (0.0165 - 0.0388)	0.0304 (0.0179 - 0.0471)	0.026 (0.0168 - 0.0372)	0.0348 (0.0203 - 0.0508)
Eritrea	Male	11.2% (10.1% - 12.6%)	9.85% (8.32% - 11.4%)	13.2% (12.1% - 14.3%)	12.6% (11.5% - 13.7%)	0.366 (0.245 - 0.495)	0.477 (0.29 - 0.693)	0.45 (0.315 - 0.611)	0.604 (0.381 - 0.844)
Ethiopia	Female	1.59% (1.23% - 2.04%)	1.9% (1.3% - 2.54%)	1.2% (1% - 1.42%)	1.24% (1.03% - 1.46%)	0.856 (0.602 - 1.22)	2.01 (1.35 - 2.82)	0.616 (0.476 - 0.767)	1.29 (1.01 - 1.64)
Ethiopia	Male	9.78% (8.96% - 10.6%)	11.2% (8.3% - 12.9%)	6.72% (6.11% - 7.35%)	6.33% (5.71% - 6.96%)	4.6 (3.86 - 5.4)	11.6 (8.36 - 14.5)	3.19 (2.62 - 3.78)	6.3 (5.13 - 7.52)
Kenya	Female	2.55% (2% - 3.2%)	1.32% (0.9% - 1.98%)	2.29% (1.93% - 2.71%)	2.26% (1.89% - 2.68%)	0.642 (0.474 - 0.864)	0.48 (0.322 - 0.723)	0.446 (0.368 - 0.53)	0.855 (0.688 - 1.07)
Kenya	Male	16.9% (15.7% - 18%)	11% (9.89% - 12.2%)	11.1% (10.1% - 12.1%)	11.5% (10.5% - 12.4%)	3.8 (3.42 - 4.22)	4.2 (3.55 - 4.93)	2.44 (2.15 - 2.75)	4.13 (3.49 - 4.83)
Madagascar	Female	2.8% (2.22% - 3.54%)	2.06% (1.54% - 2.69%)	2.58% (2.18% - 3.04%)	2.88% (2.44% - 3.35%)	0.454 (0.334 - 0.595)	0.515 (0.375 - 0.665)	0.369 (0.296 - 0.446)	0.712 (0.573 - 0.871)
Madagascar	Male	22.4% (20.8% - 24.5%)	16.2% (13.4% - 18.9%)	11.8% (10.9% - 12.8%)	12.2% (11.3% - 13.3%)	3.35 (2.97 - 3.77)	4.19 (3.3 - 5.11)	1.46 (1.28 - 1.65)	2.9 (2.51 - 3.32)
Malawi	Female	3.42% (2.8% - 4.27%)	2.72% (1.92% - 3.99%)	2.3% (1.95% - 2.71%)	2.13% (1.79% - 2.52%)	0.313 (0.236 - 0.426)	0.446 (0.302 - 0.666)	0.182 (0.151 - 0.215)	0.327 (0.263 - 0.397)
Malawi	Male	22.5% (21.2% - 23.9%)	23.6% (22.3% - 25%)	12.1% (11.2% - 13%)	11.3% (10.4% - 12.3%)	1.74 (1.57 - 1.95)	3.68 (3.11 - 4.32)	0.82 (0.73 - 0.925)	1.56 (1.3 - 1.86)
Mozambique	Female	5.81% (4.66% - 7.24%)	4.9% (3.63% - 6.53%)	2.94% (2.45% - 3.49%)	3.12% (2.61% - 3.74%)	0.931 (0.722 - 1.22)	1.45 (1.05 - 1.98)	0.467 (0.374 - 0.574)	0.917 (0.727 - 1.15)
Mozambique	Male	20.6% (19% - 22.6%)	19% (17.3% - 20.8%)	11.5% (10.6% - 12.6%)	11.6% (10.6% - 12.7%)	2.58 (2.25 - 2.92)	5.09 (4.28 - 6.03)	1.5 (1.32 - 1.7)	2.93 (2.45 - 3.48)
Rwanda	Female	7.85% (6.69% - 9.26%)	7.15% (5.96% - 8.51%)	3.34% (2.86% - 3.91%)	3.56% (3.04% - 4.14%)	0.402 (0.325 - 0.508)	0.894 (0.71 - 1.12)	0.211 (0.17 - 0.262)	0.448 (0.362 - 0.552)
Rwanda	Male	20.4% (19% - 21.7%)	20.5% (19.1% - 21.9%)	11.5% (10.5% - 12.6%)	11.6% (10.5% - 12.6%)	1.06 (0.9 - 1.22)	2.48 (2.11 - 2.88)	0.677 (0.56 - 0.783)	1.42 (1.19 - 1.67)
Somalia	Female	3.02% (2.33% - 3.85%)	2.55% (1.94% - 3.27%)	2.61% (2.17% - 3.09%)	2.86% (2.33% - 3.4%)	0.33 (0.207 - 0.5)	0.568 (0.336 - 0.867)	0.271 (0.184 - 0.365)	0.613 (0.396 - 0.831)
Somalia	Male	18.3% (16.4% - 20.4%)	16.1% (14.2% - 18.2%)	11.8% (10.8% - 12.8%)	11.9% (11% - 12.9%)	1.79 (1.29 - 2.26)	3.33 (2.26 - 4.44)	1.11 (0.787 - 1.43)	2.4 (1.6 - 3.18)
South Sudan	Female	3.08% (2.34% - 3.92%)	2.59% (1.95% - 3.33%)	2.61% (2.19% - 3.06%)	2.88% (2.41% - 3.36%)	0.155 (0.108 - 0.218)	0.288 (0.199 - 0.403)	0.124 (0.0958 - 0.156)	0.311 (0.238 - 0.397)
South Sudan	Male	18.6% (16.8% - 20.5%)	15.8% (14.2% - 17.8%)	11.8% (10.8% - 12.8%)	12.1% (11% - 13.1%)	0.803 (0.66 - 0.958)	1.66 (1.32 - 2.05)	0.527 (0.427 - 0.629)	1.21 (0.95 - 1.48)
Uganda	Female	4.44% (3.76% - 5.18%)	3.12% (2.33% - 4.61%)	5.85% (5.12% - 6.92%)	5.86% (5.09% - 6.92%)	0.954 (0.739 - 1.24)	1.34 (0.973 - 2.03)	1.1 (0.903 - 1.35)	2.42 (2.01 - 2.95)
Uganda	Male	13.2% (12.2% - 14.4%)	7.78% (6.49% - 11.1%)	9.29% (8.56% - 10.1%)	9.86% (9.02% - 10.7%)	2.55 (2.17 - 2.97)	3.36 (2.61 - 4.91)	1.58 (1.35 - 1.82)	3.83 (3.23 - 4.52)
United Republic of Tanzania	Female	2.3% (1.84% - 2.87%)	1.2% (0.84% - 1.82%)	42.7% (38.7% - 46.8%)	46.9% (42.9% - 51.4%)	0.613 (0.457 - 0.808)	0.612 (0.41 - 0.97)	13.3 (11.2 - 15.5)	25 (21.2 - 29.7)
United Republic of Tanzania	Male	13.8% (12.9% - 14.8%)	7.11% (6.29% - 8.7%)	51.7% (49.4% - 53.6%)	56.9% (54.1% - 59.1%)	3.41 (2.94 - 3.89)	3.53 (2.87 - 4.52)	14 (12.1 - 15.8)	28.4 (24 - 33.2)
Zambia	Female	6.07% (4.9% - 7.66%)	4.12% (2.59% - 6.72%)	1.39% (1.17% - 1.62%)	1.14% (0.94% - 1.36%)	0.613 (0.459 - 0.859)	0.735 (0.441 - 1.24)	0.127 (0.0982 - 0.159)	0.205 (0.158 - 0.262)
Zambia	Male	23.1% (22% - 24.3%)	21.1% (18.8% - 23.4%)	10.3% (9.48% - 11.1%)	10.2% (9.35% - 10.9%)	2.09 (1.78 - 2.42)	3.82 (3.1 - 4.69)	0.853 (0.72 - 0.995)	1.69 (1.39 - 2.01)
Southern Sub-Saharan Africa	Female	8.88% (7.74% - 10.1%)	6.17% (5.26% - 7.14%)	3.1% (2.71% - 3.54%)	2.69% (2.33% - 3.05%)	3.68 (3.12 - 4.26)	3.07 (2.57 - 3.63)	1.24 (1.03 - 1.44)	1.43 (1.18 - 1.66)
Southern Sub-Saharan Africa	Male	32.1% (31% - 33.3%)	29.5% (26.2% - 31.8%)	9.05% (8.51% - 9.61%)	9.1% (8.51% - 9.71%)	12.9 (11.6 - 14.2)	14.5 (12.2 - 16.6)	3.41 (3.03 - 3.83)	4.43 (3.92 - 5)
Botswana	Female	8.27% (6.92% - 9.82%)	7.3% (6.07% - 8.68%)	4.73% (4.12% - 5.49%)	4.76% (4.15% - 5.52%)	0.099 (0.0786 - 0.124)	0.118 (0.0948 - 0.148)	0.0544 (0.0438 - 0.0654)	0.0795 (0.0643 - 0.0979)
Botswana	Male	33.9% (32% - 36%)	36.5% (31.6% - 39.9%)	16.8% (15.6% - 18%)	16.7% (15.5% - 18.1%)	0.414 (0.358 - 0.474)	0.578 (0.471 - 0.689)	0.184 (0.156 - 0.212)	0.256 (0.216 - 0.297)
Eswatini	Female	2.79% (2.13% - 3.48%)	2.11% (1.6% - 2.69%)	1.39% (1.2% - 1.59%)	1.31% (1.13% - 1.51%)	0.0141 (0.0104 - 0.0186)	0.0148 (0.0108 - 0.0196)	0.0071 (0.0059 - 0.0085)	0.0091 (0.0072 - 0.0112)
Eswatini	Male	13.4% (12.1% - 14.7%)	10.2% (8.97% - 11.7%)	9.43% (8.72% - 10.2%)	9.09% (8.36% - 9.92%)	0.0742 (0.0632 - 0.0857)	0.0673 (0.0528 - 0.0826)	0.0426 (0.0365 - 0.0482)	0.0543 (0.0428 - 0.0662)
Lesotho	Female	2.81% (1.97% - 3.8%)	3.24% (2.28% - 4.37%)	1.75% (1.5% - 2.03%)	1.48% (1.26% - 1.71%)	0.0253 (0.0171 - 0.037)	0.0339 (0.024 - 0.0463)	0.0152 (0.0126 - 0.0178)	0.0172 (0.0136 - 0.021)
Lesotho	Male	40.8% (38.8% - 42.9%)	49.9% (46% - 52.5%)	9.95% (9.16% - 10.8%)	9.33% (8.57% - 10.1%)	0.377 (0.338 - 0.419)	0.556 (0.462 - 0.662)	0.0807 (0.0699 - 0.0919)	0.0966 (0.0792 - 0.114)
Namibia	Female	10.4% (8.57% - 12.5%)	7.11% (5.64% - 9.08%)	3.36% (2.95% - 3.88%)	2.88% (2.5% - 3.36%)	0.122 (0.0956 - 0.154)	0.129 (0.0959 - 0.171)	0.0371 (0.0302 - 0.0441)	0.0532 (0.0424 - 0.0651)
Namibia	Male	22.1% (20.5% - 23.6%)	17.9% (16.6% - 19.2%)	10.8% (9.89% - 11.7%)	10.9% (9.98% - 11.8%)	0.251 (0.213 - 0.288)	0.298 (0.25 - 0.352)	0.12 (0.1 - 0.138)	0.181 (0.15 - 0.218)
South Africa	Female	10.4% (8.81% - 12.1%)	7.25% (6.02% - 8.57%)	3.46% (2.92% - 4.03%)	3.06% (2.58% - 3.62%)	3.03 (2.48 - 3.6)	2.35 (1.88 - 2.87)	0.984 (0.779 - 1.18)	1.08 (0.852 - 1.32)
South Africa	Male	32.7% (31.2% - 34.3%)	28% (24.2% - 30.8%)	7.56% (6.99% - 8.25%)	7.19% (6.59% - 7.87%)	9.49 (8.23 - 10.8)	9.19 (7.49 - 10.9)	2.03 (1.75 - 2.37)	2.38 (2.03 - 2.78)
Zimbabwe	Female	4.36% (3.46% - 5.45%)	3.7% (2.77% - 5.01%)	1.62% (1.36% - 1.91%)	1.53% (1.28% - 1.81%)	0.358 (0.268 - 0.477)	0.419 (0.289 - 0.596)	0.108 (0.0881 - 0.132)	0.165 (0.128 - 0.209)
Zimbabwe	Male	32.5% (30.8% - 34.3%)	36.3% (32.4% - 38.9%)	15.3% (14.2% - 16.6%)	15.1% (14% - 16.4%)	2.22 (1.95 - 2.54)	3.85 (3.11 - 4.6)	1.06 (0.91 - 1.23)	1.58 (1.29 - 1.87)
Western Sub-Saharan Africa	Female	1.68% (1.53% - 1.85%)	1.21% (1.07% - 1.42%)	1.16% (1.07% - 1.25%)	1.11% (1.03% - 1.21%)	4.44 (3.91 - 5.05)	6.01 (5.11 - 7.23)	2.76 (2.5 - 3.05)	5.3 (4.71 - 5.97)
Western Sub-Saharan Africa	Male	10.4% (9.95% - 10.8%)	7.75% (7.17% - 8.7%)	8.23% (7.94% - 8.52%)	8.04% (7.69% - 8.35%)	24.1 (22.3 - 26)	37.7 (32.8 - 43.8)	16.1 (15 - 17.2)	33.7 (30.1 - 37.2)
Benin	Female	1.52% (1.2% - 1.9%)	0.78% (0.58% - 1.11%)	1.39% (1.18% - 1.61%)	1.29% (1.09% - 1.51%)	0.104 (0.0771 - 0.142)	0.113 (0.0808 - 0.16)	0.0892 (0.0717 - 0.111)	0.181 (0.146 - 0.226)
Benin	Male	8.4% (7.77% - 9.1%)	4.42% (3.95% - 5.39%)	3.82% (3.46% - 4.18%)	3.8% (3.44% - 4.17%)	0.507 (0.429 - 0.59)	0.613 (0.497 - 0.787)	0.172 (0.147 - 0.198)	0.406 (0.336 - 0.487)
Burkina Faso	Female	1.52% (1.15% - 1.98%)	1.03% (0.75% - 1.38%)	1.42% (1.17% - 1.68%)	1.36% (1.13% - 1.61%)	0.176 (0.129 - 0.233)	0.266 (0.188 - 0.366)	0.164 (0.135 - 0.199)	0.35 (0.287 - 0.429)
Burkina Faso	Male	15.5% (14.2% - 16.9%)	12.3% (11.2% - 13.6%)	14% (13% - 15.1%)	14.2% (13.1% - 15.3%)	1.72 (1.52 - 1.95)	3.28 (2.81 - 3.79)	1.27 (1.12 - 1.42)	2.99 (2.53 - 3.43)
Cabo Verde	Female	2.73% (2.15% - 3.35%)	2.19% (1.64% - 2.75%)	3.5% (2.95% - 4.06%)	3.26% (2.74% - 3.79%)	0.0076 (0.0056 - 0.0096)	0.0075 (0.0054 - 0.0101)	0.0097 (0.0077 - 0.012)	0.011 (0.0085 - 0.014)
Cabo Verde	Male	8.6% (7.62% - 9.72%)	6.51% (5.13% - 8.06%)	9.93% (9.1% - 10.8%)	9.36% (8.52% - 10.2%)	0.0252 (0.0209 - 0.0305)	0.0215 (0.0159 - 0.0285)	0.0247 (0.0209 - 0.029)	0.0323 (0.0262 - 0.0397)
Cameroon	Female	1.37% (1.07% - 1.72%)	0.93% (0.71% - 1.21%)	0.94% (0.77% - 1.12%)	0.94% (0.77% - 1.12%)	0.223 (0.155 - 0.306)	0.259 (0.183 - 0.355)	0.154 (0.118 - 0.2)	0.27 (0.203 - 0.356)
Cameroon	Male	12.5% (11.4% - 13.6%)	8.21% (7.36% - 9.17%)	9.77% (8.99% - 10.5%)	9.96% (9.16% - 10.7%)	1.88 (1.56 - 2.26)	2.35 (1.85 - 2.94)	1.27 (1.04 - 1.52)	2.55 (2.02 - 3.12)
Chad	Female	2.74% (2.13% - 3.45%)	1.66% (1.23% - 2.29%)	1.37% (1.17% - 1.63%)	1.2% (1% - 1.42%)	0.262 (0.191 - 0.369)	0.441 (0.306 - 0.653)	0.12 (0.0916 - 0.153)	0.298 (0.227 - 0.38)
Chad	Male	14.2% (12.9% - 15.4%)	9.82% (8.8% - 10.8%)	9.63% (8.84% - 10.5%)	9.16% (8.36% - 10.1%)	1.15 (0.967 - 1.36)	2.4 (1.98 - 2.84)	0.702 (0.589 - 0.83)	1.8 (1.48 - 2.16)

Supplementary Results Table S1. Global all-age and age-standardised prevalence of current and former smokers by location, sex, and scenario, 2022 and 2050

Location	Sex	Current Smoking Prevalence 2022	Current Smoking Prevalence 2050	Former Smoking Prevalence 2022	Former Smoking Prevalence 2050	Current Smokers (millions) 2022	Current Smokers (millions) 2050	Former Smokers (millions) 2022	Former Smokers (millions) 2050
Côte d'Ivoire	Female	2.5% (1.91% - 3.18%)	1.77% (1.27% - 2.5%)	1.23% (1.01% - 1.44%)	1.12% (0.92% - 1.33%)	0.36 (0.265 - 0.493)	0.433 (0.3 - 0.655)	0.163 (0.128 - 0.202)	0.269 (0.206 - 0.336)
Côte d'Ivoire	Male	16.7% (15.3% - 18%)	12.6% (10.6% - 16.5%)	9.78% (8.99% - 10.7%)	9.17% (8.37% - 10%)	2.53 (2.17 - 2.9)	3.19 (2.5 - 4.32)	1.16 (1 - 1.34)	2.09 (1.73 - 2.47)
Gambia	Female	0.93% (0.71% - 1.19%)	0.63% (0.48% - 0.8%)	1.49% (1.25% - 1.78%)	1.51% (1.25% - 1.8%)	0.0124 (0.009 - 0.0172)	0.0119 (0.0089 - 0.0162)	0.0166 (0.0132 - 0.0207)	0.0282 (0.0223 - 0.036)
Gambia	Male	17.9% (16.7% - 19.3%)	12.7% (11.8% - 13.8%)	15.3% (14.2% - 16.6%)	15.4% (14.3% - 16.7%)	0.203 (0.175 - 0.231)	0.236 (0.196 - 0.279)	0.154 (0.132 - 0.176)	0.263 (0.217 - 0.312)
Ghana	Female	2.66% (2.1% - 3.35%)	3.06% (2.41% - 3.88%)	1.2% (1.02% - 1.39%)	1.19% (1.01% - 1.38%)	0.494 (0.373 - 0.653)	0.817 (0.601 - 1.07)	0.192 (0.152 - 0.235)	0.333 (0.253 - 0.415)
Ghana	Male	10.5% (9.56% - 11.6%)	12.3% (10.2% - 13.8%)	10.4% (9.67% - 11.2%)	10.2% (9.48% - 11.1%)	1.64 (1.39 - 1.91)	3.33 (2.54 - 4.04)	1.37 (1.17 - 1.57)	2.53 (2.05 - 3.03)
Guinea	Female	1.93% (1.42% - 2.6%)	1.67% (1.23% - 2.22%)	1.19% (0.99% - 1.41%)	1.06% (0.88% - 1.27%)	0.148 (0.0961 - 0.222)	0.23 (0.157 - 0.335)	0.0799 (0.0641 - 0.1)	0.14 (0.108 - 0.175)
Guinea	Male	15.2% (14% - 16.7%)	11.4% (10.2% - 13%)	9.15% (8.53% - 9.96%)	9.2% (8.56% - 9.97%)	0.952 (0.814 - 1.11)	1.49 (1.24 - 1.77)	0.5 (0.429 - 0.565)	0.996 (0.842 - 1.16)
Guinea-Bissau	Female	1.37% (1.07% - 1.83%)	1.86% (1.26% - 2.55%)	1.34% (1.12% - 1.6%)	1.18% (0.97% - 1.42%)	0.0153 (0.0109 - 0.022)	0.0359 (0.023 - 0.0513)	0.0138 (0.0107 - 0.0173)	0.0225 (0.0174 - 0.0289)
Guinea-Bissau	Male	9.92% (8.8% - 11.1%)	13.8% (10.3% - 16.2%)	9.86% (8.99% - 10.7%)	9.01% (8.18% - 9.83%)	0.0969 (0.0807 - 0.114)	0.262 (0.189 - 0.331)	0.0773 (0.0652 - 0.0907)	0.142 (0.113 - 0.171)
Liberia	Female	2.14% (1.68% - 2.67%)	1.78% (1.31% - 2.43%)	2.35% (1.93% - 2.82%)	2.28% (1.86% - 2.72%)	0.0583 (0.0417 - 0.0759)	0.0807 (0.0559 - 0.114)	0.0537 (0.041 - 0.0678)	0.101 (0.0745 - 0.129)
Liberia	Male	11.2% (10.2% - 12.3%)	9.28% (8.24% - 10.6%)	13.2% (12.2% - 14.3%)	13.1% (12.2% - 14.2%)	0.285 (0.232 - 0.335)	0.431 (0.335 - 0.535)	0.309 (0.257 - 0.372)	0.572 (0.459 - 0.7)
Mali	Female	2.82% (2.11% - 3.65%)	3.4% (2.51% - 4.5%)	1.33% (1.11% - 1.57%)	1.18% (0.97% - 1.39%)	0.335 (0.232 - 0.48)	0.968 (0.676 - 1.37)	0.156 (0.122 - 0.192)	0.331 (0.257 - 0.414)
Mali	Male	19.1% (17.8% - 20.6%)	19.1% (16.5% - 22.8%)	10.1% (9.29% - 11%)	10.1% (9.32% - 11%)	2.27 (1.89 - 2.68)	5.57 (4.41 - 7.01)	1.06 (0.892 - 1.23)	2.55 (2.12 - 3.04)
Mauritania	Female	8.86% (7.29% - 10.6%)	7.24% (5.9% - 8.86%)	1.44% (1.2% - 1.69%)	1.29% (1.07% - 1.56%)	0.232 (0.183 - 0.286)	0.288 (0.215 - 0.375)	0.0324 (0.0255 - 0.04)	0.0501 (0.039 - 0.0631)
Mauritania	Male	26.8% (24.6% - 29.4%)	21.9% (19.8% - 24.1%)	9.24% (8.52% - 10%)	8.64% (7.88% - 9.48%)	0.594 (0.504 - 0.694)	0.867 (0.703 - 1.05)	0.171 (0.148 - 0.198)	0.305 (0.254 - 0.37)
Niger	Female	1.44% (1.09% - 1.85%)	1.07% (0.77% - 1.54%)	1.32% (1.12% - 1.6%)	1.27% (1.07% - 1.53%)	0.212 (0.148 - 0.286)	0.433 (0.293 - 0.654)	0.166 (0.131 - 0.211)	0.448 (0.345 - 0.582)
Niger	Male	12.5% (11.3% - 13.6%)	9.78% (8.52% - 12%)	11% (10.2% - 11.9%)	10.9% (10.1% - 11.8%)	1.67 (1.39 - 2)	3.86 (3.09 - 4.86)	1.22 (1.03 - 1.42)	3.38 (2.78 - 4.06)
Nigeria	Female	1% (0.74% - 1.31%)	0.5% (0.36% - 0.68%)	1% (0.85% - 1.18%)	0.97% (0.82% - 1.15%)	1.22 (0.852 - 1.78)	1.11 (0.762 - 1.61)	1.12 (0.908 - 1.38)	2.11 (1.68 - 2.68)
Nigeria	Male	5.87% (5.19% - 6.52%)	3.38% (2.91% - 3.97%)	5.56% (5.14% - 6.03%)	5.25% (4.76% - 5.81%)	6.14 (5.18 - 7.19)	7.39 (5.71 - 9.36)	4.79 (4.21 - 5.52)	9.72 (8 - 11.6)
Sao Tome and Principe	Female	1.87% (1.36% - 2.51%)	1.61% (1.16% - 2.27%)	2.94% (2.5% - 3.46%)	2.87% (2.42% - 3.36%)	0.002 (0.0014 - 0.0029)	0.002 (0.0014 - 0.0028)	0.0029 (0.0023 - 0.0035)	0.0036 (0.0027 - 0.0047)
Sao Tome and Principe	Male	7.44% (6.6% - 8.23%)	6.85% (5.9% - 8.28%)	18.6% (17.3% - 20.2%)	20% (18.6% - 21.7%)	0.0078 (0.0064 - 0.0091)	0.0085 (0.0065 - 0.011)	0.0189 (0.0161 - 0.0217)	0.0251 (0.0193 - 0.0313)
Senegal	Female	1.5% (1.1% - 2.04%)	0.78% (0.54% - 1.17%)	0.65% (0.54% - 0.77%)	0.65% (0.54% - 0.78%)	0.138 (0.0941 - 0.195)	0.106 (0.0721 - 0.165)	0.0524 (0.0417 - 0.0648)	0.0879 (0.0694 - 0.109)
Senegal	Male	12.3% (11.4% - 13.3%)	5.75% (5.17% - 6.73%)	16% (14.8% - 17.1%)	16.9% (15.7% - 18.1%)	0.992 (0.844 - 1.13)	0.809 (0.666 - 0.985)	1.1 (0.954 - 1.25)	2.2 (1.84 - 2.57)
Sierra Leone	Female	7.08% (5.73% - 8.58%)	4.69% (3.53% - 6.33%)	2.74% (2.29% - 3.3%)	2.33% (1.87% - 2.93%)	0.351 (0.269 - 0.464)	0.375 (0.269 - 0.523)	0.104 (0.0855 - 0.13)	0.177 (0.137 - 0.23)
Sierra Leone	Male	24.1% (22.7% - 25.5%)	18.1% (16.2% - 20.5%)	6.87% (6.29% - 7.5%)	6.47% (5.88% - 7.18%)	1.02 (0.9 - 1.15)	1.4 (1.18 - 1.67)	0.213 (0.186 - 0.243)	0.43 (0.354 - 0.515)
Togo	Female	2.11% (1.62% - 2.74%)	1.37% (1.04% - 1.79%)	1.54% (1.26% - 1.85%)	1.52% (1.24% - 1.84%)	0.0881 (0.0635 - 0.124)	0.0899 (0.0661 - 0.126)	0.0667 (0.0521 - 0.0849)	0.101 (0.0786 - 0.128)
Togo	Male	12.4% (11.2% - 13.4%)	8.32% (7.47% - 9.25%)	11.8% (10.8% - 12.7%)	11.8% (10.8% - 12.8%)	0.481 (0.397 - 0.562)	0.545 (0.438 - 0.659)	0.429 (0.355 - 0.501)	0.733 (0.58 - 0.876)

Supplementary Results Table S2. Cumulative all-age counts and age-standardised rates of years of life lost (YLLs) between 2022 to 2050 by location, sex, and scenario

Location	Sex	All-Age Count (Millions)				Age-Standardized Rate (per 100,000)			
		Reference	Difference between Reference and Elimination - 2023	Difference between Reference and Elimination - 2050	Difference between Elimination - 2050 and Elimination - 2023	Reference	Difference between Reference and Elimination - 2023	Difference between Reference and Elimination - 2050	Difference between Elimination - 2050 and Elimination - 2023
Global	Female	22200 (20100–24600)	341 (318–369)	141 (131–154)	200 (186–216)	14600 (12900–16700)	230 (207–259)	95.7 (85.2–108)	135 (121–151)
Global	Male	29300 (26800–32400)	1700 (1570–1850)	735 (675–808)	967 (897–1050)	20600 (18600–23300)	1300 (1190–1450)	560 (506–629)	745 (675–828)
Central Europe, Eastern Europe, and Central Asia	Female	1250 (1140–1370)	18.8 (17.2–20.5)	4.66 (4.27–5.14)	14.1 (13–15.5)	11000 (9840–12400)	216 (194–241)	58.5 (52.6–65.8)	157 (143–175)
Central Europe, Eastern Europe, and Central Asia	Male	1660 (1530–1820)	122 (113–133)	42.9 (39.4–47.6)	79 (73.2–86)	20600 (19000–22600)	1720 (1580–1880)	597 (544–668)	1120 (1030–1220)
Central Asia	Female	239 (203–293)	1.79 (1.47–2.32)	0.439 (0.345–0.588)	1.36 (1.12–1.72)	12500 (10600–15200)	102 (82.6–128)	25.2 (19.7–33)	76.6 (62.9–95.6)
Central Asia	Male	337 (291–405)	26.2 (22.9–31.2)	11.6 (9.86–14.3)	14.5 (12.8–17)	20500 (17700–24300)	1860 (1600–2230)	842 (706–1030)	1020 (889–1200)
Armenia	Female	6.87 (5.83–7.9)	0.0393 (0.0322–0.0462)	0.00746 (0.00598–0.00891)	0.0318 (0.0262–0.0373)	8150 (7250–9220)	57.1 (49–66.2)	12 (10.1–14.3)	45.1 (38.8–51.9)
Armenia	Male	8.84 (7.47–10.2)	1.2 (1.02–1.38)	0.531 (0.449–0.611)	0.674 (0.577–0.775)	15100 (13500–16800)	2320 (2050–2620)	1000 (883–1130)	1320 (1160–1490)
Azerbaijan	Female	27.1 (22.3–32.4)	0.0823 (0.0619–0.111)	0.00744 (0.0054–0.0106)	0.0748 (0.0565–0.101)	12900 (10900–15400)	37.8 (29.2–51.1)	3.95 (2.87–5.68)	33.8 (26.4–45.5)
Azerbaijan	Male	36.5 (30.1–43.4)	4.25 (3.48–5.12)	2.2 (1.78–2.66)	2.05 (1.67–2.46)	19700 (16800–23200)	2420 (2020–2920)	1300 (1080–1590)	1120 (933–1330)
Georgia	Female	10.1 (8.75–11.5)	0.122 (0.104–0.14)	0.0203 (0.0166–0.0257)	0.101 (0.0868–0.116)	9550 (8640–10500)	156 (137–177)	29.5 (25.5–34.4)	127 (112–143)
Georgia	Male	15.1 (13.1–17.1)	1.67 (1.43–1.91)	0.748 (0.64–0.858)	0.922 (0.794–1.05)	22200 (20300–24100)	3040 (2760–3330)	1340 (1210–1480)	1700 (1550–1860)
Kazakhstan	Female	47.2 (40.7–55.1)	0.354 (0.295–0.428)	0.0383 (0.03–0.0493)	0.316 (0.264–0.38)	11500 (10100–13300)	103 (85.8–125)	12.7 (10.2–16.3)	90 (75.5–109)
Kazakhstan	Male	68.7 (59.9–79.3)	4.79 (4.09–5.52)	1.32 (1.1–1.59)	3.47 (3–3.97)	21900 (19400–24900)	1790 (1570–2080)	469 (407–556)	1320 (1160–1520)
Kyrgyzstan	Female	14.7 (11.7–18)	0.236 (0.179–0.306)	0.0534 (0.0396–0.0705)	0.182 (0.139–0.235)	11900 (9830–14300)	207 (162–260)	46.8 (36–60)	160 (126–201)
Kyrgyzstan	Male	22.4 (17.9–27.5)	2.49 (1.99–3.05)	1.32 (1.05–1.63)	1.17 (0.948–1.41)	21500 (17600–25500)	2990 (2400–3650)	1640 (1300–2030)	1350 (1090–1640)
Mongolia	Female	8.69 (7.45–10.2)	0.201 (0.171–0.238)	0.0888 (0.0751–0.107)	0.112 (0.0958–0.132)	12700 (10800–15300)	330 (275–401)	144 (119–176)	186 (155–226)
Mongolia	Male	16.2 (13.8–18.8)	1.54 (1.32–1.81)	0.826 (0.695–0.983)	0.715 (0.622–0.822)	27800 (23500–33300)	3340 (2740–4080)	1740 (1410–2160)	1600 (1330–1940)
Tajikistan	Female	24.8 (19.8–31)	0.148 (0.108–0.204)	0.0569 (0.0406–0.0808)	0.0908 (0.0675–0.123)	14100 (11300–18000)	92.5 (67.4–130)	34 (24.1–49.4)	58.6 (43.1–81.2)
Tajikistan	Male	32.6 (25.7–40.6)	1.25 (0.966–1.58)	0.521 (0.398–0.666)	0.732 (0.564–0.915)	18300 (14900–22100)	908 (701–1170)	402 (309–515)	506 (392–651)
Turkmenistan	Female	14.5 (11.4–18.7)	0.104 (0.0708–0.153)	0.0208 (0.0132–0.033)	0.0834 (0.0577–0.12)	15400 (11900–20800)	121 (80.7–185)	24.4 (15.2–39.1)	96.9 (65.4–145)
Turkmenistan	Male	22 (17.6–28.1)	0.994 (0.751–1.3)	0.283 (0.212–0.388)	0.711 (0.54–0.922)	25200 (19800–33000)	1310 (932–1840)	395 (278–558)	919 (655–1280)
Uzbekistan	Female	85.2 (59.6–120)	0.508 (0.339–0.807)	0.145 (0.0937–0.235)	0.363 (0.245–0.567)	13100 (10700–16800)	79.8 (59.7–111)	23.1 (16.7–32.6)	56.7 (43–78.8)
Uzbekistan	Male	114 (79.9–161)	7.96 (5.44–11.6)	3.86 (2.61–5.77)	4.09 (2.8–5.83)	19500 (15900–24400)	1520 (1200–2010)	761 (585–1030)	763 (602–988)
Central Europe	Female	318 (296–338)	10 (9.16–10.7)	2.97 (2.7–3.22)	7.05 (6.48–7.51)	8170 (7620–8770)	384 (356–412)	125 (114–135)	260 (242–278)
Central Europe	Male	419 (390–447)	29.5 (27.2–31.7)	10.3 (9.47–11.1)	19.2 (17.8–20.6)	15300 (14300–16300)	1330 (1240–1430)	462 (430–496)	868 (805–929)
Albania	Female	6.4 (5.38–7.45)	0.201 (0.165–0.237)	0.0685 (0.0562–0.0812)	0.132 (0.109–0.155)	8850 (7540–10300)	333 (274–391)	119 (97.4–140)	214 (177–251)
Albania	Male	7.9 (6.69–9.16)	0.963 (0.797–1.13)	0.545 (0.451–0.638)	0.418 (0.341–0.495)	13000 (11100–15100)	1930 (1620–2280)	1120 (945–1310)	817 (679–970)
Bosnia and Herzegovina	Female	8.9 (7.51–10.3)	0.419 (0.35–0.5)	0.0981 (0.0825–0.117)	0.321 (0.268–0.382)	8010 (6640–9270)	537 (435–642)	140 (110–169)	398 (325–473)
Bosnia and Herzegovina	Male	10.8 (8.96–12.8)	1.21 (0.998–1.43)	0.488 (0.409–0.574)	0.722 (0.586–0.862)	13000 (10500–15200)	1800 (1400–2160)	729 (573–869)	1070 (829–1290)
Bulgaria	Female	24.5 (21.4–27.5)	0.593 (0.495–0.706)	0.134 (0.109–0.161)	0.459 (0.386–0.545)	11900 (10500–13400)	470 (397–546)	123 (102–145)	347 (294–402)
Bulgaria	Male	31.5 (27.4–35.8)	1.93 (1.65–2.23)	0.502 (0.421–0.587)	1.43 (1.23–1.64)	21700 (19100–24500)	1680 (1450–1930)	434 (369–506)	1240 (1080–1430)
Croatia	Female	11.3 (9.78–12.8)	0.347 (0.293–0.408)	0.0998 (0.0829–0.122)	0.247 (0.209–0.29)	7190 (6500–7930)	367 (327–412)	122 (108–137)	246 (220–274)
Croatia	Male	13.9 (11.9–15.8)	1.12 (0.95–1.3)	0.428 (0.364–0.493)	0.695 (0.585–0.804)	12700 (11400–13900)	1370 (1220–1510)	547 (493–606)	818 (727–909)
Czechia	Female	26.4 (23.3–29.6)	0.758 (0.663–0.863)	0.19 (0.163–0.22)	0.568 (0.498–0.643)	6470 (5830–7170)	285 (252–317)	78.5 (68–88.4)	206 (183–229)
Czechia	Male	34 (29.9–38.1)	2.38 (2.05–2.67)	0.873 (0.751–0.982)	1.51 (1.3–1.69)	11600 (10500–12800)	999 (886–1110)	362 (321–407)	637 (565–707)
Hungary	Female	30.4 (26.3–34.4)	0.99 (0.826–1.15)	0.276 (0.229–0.326)	0.714 (0.594–0.821)	8720 (7870–9610)	441 (387–493)	134 (116–152)	307 (270–342)
Hungary	Male	36.1 (30.9–40.9)	2.4 (2.02–2.79)	0.74 (0.62–0.862)	1.66 (1.4–1.92)	15100 (13700–16600)	1270 (1120–1410)	380 (335–423)	889 (784–986)
Montenegro	Female	1.74 (1.51–2)	0.0769 (0.0649–0.0904)	0.0225 (0.0187–0.0268)	0.0544 (0.046–0.0637)	10000 (8990–11200)	602 (527–688)	190 (166–220)	412 (361–466)
Montenegro	Male	2.19 (1.88–2.53)	0.211 (0.174–0.249)	0.0898 (0.074–0.106)	0.121 (0.101–0.143)	16600 (14700–18600)	1960 (1680–2250)	824 (706–947)	1140 (980–1300)
North Macedonia	Female	6.51 (5.28–7.89)	0.245 (0.192–0.31)	0.0568 (0.0439–0.0731)	0.189 (0.149–0.239)	12300 (10500–14200)	641 (535–752)	200 (168–235)	441 (367–519)
North Macedonia	Male	8.1 (6.51–9.86)	0.755 (0.586–0.947)	0.3 (0.23–0.378)	0.455 (0.356–0.569)	16200 (14000–18800)	1810 (1490–2150)	744 (621–880)	1070 (875–1270)
Poland	Female	98.8 (86.9–109)	3.58 (3.07–4.09)	1.21 (1.02–1.39)	2.37 (2.04–2.72)	7200 (6530–7750)	378 (335–419)	138 (120–155)	240 (215–264)
Poland	Male	139 (123–155)	9.4 (8.19–10.6)	3.28 (2.81–3.71)	6.12 (5.36–6.89)	15200 (13800–16500)	1230 (1090–1360)	427 (379–472)	803 (715–886)
Romania	Female	56.8 (48.3–66.2)	1.22 (1–1.45)	0.28 (0.225–0.339)	0.936 (0.777–1.11)	9330 (8310–10400)	317 (276–362)	86 (74.2–100)	231 (202–261)
Romania	Male	77.4 (65.4–90.1)	4.83 (4.06–5.69)	1.48 (1.24–1.75)	3.36 (2.82–3.93)	18000 (16200–19900)	1420 (1270–1580)	431 (382–484)	989 (883–1100)
Serbia	Female	26.8 (23–30.7)	1.24 (1.02–1.46)	0.44 (0.362–0.523)	0.797 (0.657–0.936)	9830 (8800–10900)	662 (580–760)	252 (218–292)	410 (360–470)
Serbia	Male	32.6 (27.9–37.3)	2.61 (2.18–3.07)	0.868 (0.72–1.04)	1.74 (1.45–2.04)	15300 (13500–17500)	1570 (1350–1820)	519 (446–608)	1050 (905–1220)
Slovakia	Female	14.5 (12.9–16.2)	0.246 (0.207–0.291)	0.0696 (0.0568–0.0842)	0.176 (0.15–0.206)	8020 (7140–9030)	202 (174–236)	63.6 (54.1–75)	139 (120–161)
Slovakia	Male	19.3 (17–21.6)	1.41 (1.21–1.6)	0.622 (0.535–0.704)	0.79 (0.678–0.896)	14700 (13000–16300)	1290 (1130–1450)	551 (484–622)	735 (644–832)
Slovenia	Female	4.54 (4.03–5.08)	0.108 (0.088–0.128)	0.0285 (0.0227–0.0344)	0.0792 (0.065–0.0937)	4810 (4260–5340)	198 (168–230)	61.4 (51.2–72.4)	137 (117–158)
Slovenia	Male	6.13 (5.45–6.85)	0.294 (0.25–0.336)	0.0755 (0.0629–0.087)	0.219 (0.187–0.25)	9250 (8380–10100)	581 (506–654)	159 (136–179)	423 (370–476)
Eastern Europe	Female	691 (605–783)	6.96 (5.83–8.28)	1.25 (1.01–1.56)	5.71 (4.8–6.75)	11000 (9720–12500)	171 (145–207)	38.9 (31.3–48.5)	132 (112–158)
Eastern Europe	Male	907 (801–1030)	66.3 (58.7–75)	21 (18.6–24.1)	45.2 (40.2–51.3)	22700 (20400–25300)	1910 (1680–2170)	594 (523–685)	1320 (1160–1490)
Belarus	Female	30.1 (25.1–35.7)	0.258 (0.201–0.338)	0.0283 (0.0206–0.04)	0.229 (0.179–0.297)	9900 (8270–12000)	141 (111–186)	20.6 (15.7–29.2)	120 (94.7–156)
Belarus	Male	42.3 (35.1–49.9)	4.71 (3.97–5.46)	2.3 (1.95–2.66)	2.41 (2.03–2.8)	23300 (19600–27500)	3220 (2660–3850)	1550 (1280–1870)	1670 (1370–1980)
Estonia	Female	3.38 (3.01–3.76)	0.0479 (0.0414–0.0551)	0.0146 (0.0123–0.0169)	0.0333 (0.029–0.0384)	6330 (5680–6990)	151 (131–172)	49.7 (43.1–56.4)	102 (88.3–115)
Estonia	Male	4.24 (3.75–4.74)	0.211 (0.182–0.24)	0.0597 (0.0516–0.0683)	0.151 (0.131–0.172)	13500 (12100–14900)	823 (723–930)	229 (201–258)	594 (522–673)
Latvia	Female	5.87 (5.21–6.52)	0.0776 (0.0661–0.0886)	0.0165 (0.0138–0.0191)	0.0611 (0.0526–0.0695)	8780 (7840–9760)	192 (168–217)	47.3 (41.1–54.5)	145 (127–163)
Latvia	Male	7.26 (6.42–8.07)	0.458 (0.405–0.516)	0.14 (0.122–0.16)	0.318 (0.282–0.358)	19200 (17200–20900)	1490 (1310–1660)	450 (395–503)	1040 (913–1150)
Lithuania	Female	8.38 (7.41–9.3)	0.0852 (0.0725–0.0977)	0.0237 (0.0199–0.0279)	0.0614 (0.0526–0.0704)	8450 (7490–9350)	158 (137–179)	51 (43.7–58.5)	107 (92.9–121)
Lithuania	Male	10.8 (9.65–12)	0.706 (0.626–0.787)	0.249 (0.22–0.279)	0.457 (0.407–0.511)	19700 (17800–21500)	1610 (1440–1780)	580 (521–637)	1030 (919–1150)
Republic of Moldova	Female	10.3 (8.34–12.6)	0.0968 (0.0752–0.124)	0.00824 (0.00611–0.0112)	0.0886 (0.0684–0.113)	10100 (8780–11900)	119 (99.8–149)	12.6 (10.2–16.5)	106 (89.3–132)
Republic of Moldova	Male	14.3 (11.6–17.2)	1.39 (1.11–1.67)	0.504 (0.396–0.618)	0.891 (0.719–1.06)	20300 (17800–23200)	2200 (1960–2550)	787 (690–937)	1410 (1260–1600)
Russian Federation	Female	481 (408–558)	5.33 (4.33–6.45)	1.04 (0.825–1.3)	4.29 (3.51–5.15)	11000 (9810–12300)	187 (158–221)	45.3 (36.3–56.5)	142 (121–166)
Russian Federation	Male	632 (537–733)	46.3 (39.4–54)	14.5 (12.3–17.2)	31.8 (27.1–37)	22500 (20200–24700)	1890 (1670–2140)	575 (500–666)	1320 (1170–1480)
Ukraine	Female	152 (115–202)	1.07 (0.709–1.58)	0.122 (0.0833–0.183)	0.946 (0.62–1.39)	11800 (8320–16600)	129 (79.6–190)	20.7 (12.3–31.8)	108 (67.5–159)
Ukraine	Male	196 (141–258)	12.5 (9.17–15.9)	3.28 (2.47–4.13)	9.22 (6.74–11.8)	24600 (17300–33400)	1810 (1230–2520)	498 (342–674)	1310 (877–1840)
High-income	Female	2560 (2480–2650)	77.8 (74.5–81)	22.7 (21.7–23.7)	55.1 (52.8–57.4)	6560 (6390–6760)	280 (272–287)	87.1 (84.3–90)	193 (188–197)
High-income	Male	3140 (3050–3240)	150 (145–156)	50.3 (48.5–52.5)	99.7 (96.6–104)	10600 (10400–10800)	614 (602–630)	207 (202–214)	407 (400–417)
Australasia	Female	61.2 (57.7–64.9)	1.5 (1.4–1.59)	0.346 (0.324–0.369)	1.15 (1.08–1.22)	5410 (5250–5580)	185 (179–192)	47.7 (45.6–50)	137 (133–143)
Australasia	Male	75.3 (70.6–79.6)	1.99 (1.88–2.12)	0.634 (0.597–0.675)	1.36 (1.28–1.45)	8400 (8190–8650)	287 (276–297)	94.3 (90–98.8)	192 (186–199)

Supplementary Results Table S2. Cumulative all-age counts and age-standardised rates of years of life lost (YLLs) between 2022 to 2050 by location, sex, and scenario

Location	Sex	All-Age Count (Millions): Reference	All-Age Count (Millions): Difference between Reference and Elimination - 2023	All-Age Count (Millions): Difference between Reference and Elimination - 2050	All-Age Count (Millions): Difference between Elimination - 2050 and Elimination - 2023	Age-Standardized Rate (per 100,000): Reference	Age-Standardized Rate (per 100,000): Difference between Reference and Elimination - 2023	Age-Standardized Rate (per 100,000): Difference between Reference and Elimination - 2050	Age-Standardized Rate (per 100,000): Difference between Elimination - 2050 and Elimination - 2023
Australia	Female	50.3 (46.7–53.8)	1.14 (1.06–1.23)	0.249 (0.23–0.268)	0.89 (0.823–0.96)	5220 (5070–5400)	169 (163–176)	42.4 (40.1–44.8)	127 (122–132)
Australia	Male	62.7 (58.1–67.1)	1.46 (1.35–1.57)	0.397 (0.365–0.43)	1.06 (0.985–1.14)	8330 (8110–8570)	254 (245–265)	73.9 (70.1–78.2)	180 (173–188)
New Zealand	Female	11 (10–11.9)	0.357 (0.326–0.391)	0.0965 (0.0882–0.106)	0.26 (0.237–0.286)	6380 (6140–6640)	268 (254–282)	76.1 (71.8–80.3)	192 (182–202)
New Zealand	Male	12.7 (11.6–13.7)	0.534 (0.487–0.583)	0.237 (0.215–0.259)	0.297 (0.271–0.325)	8780 (8480–9080)	459 (439–479)	204 (195–213)	255 (244–266)
High-income Asia Pacific	Female	378 (355–401)	5.36 (5.02–5.73)	1.42 (1.31–1.56)	3.93 (3.67–4.2)	4070 (3990–4160)	87.9 (85.3–90.9)	19.9 (18.8–21.2)	68 (66–70.2)
High-income Asia Pacific	Male	484 (455–513)	23.7 (22.3–25.4)	5.54 (5.19–6)	18.1 (17–19.4)	7460 (7320–7610)	480 (464–501)	108 (103–114)	373 (361–388)
Brunei Darussalam	Female	0.931 (0.789–1.09)	0.0311 (0.0263–0.0365)	0.0141 (0.012–0.0167)	0.017 (0.0144–0.0199)	11100 (9890–12500)	409 (361–468)	190 (167–216)	220 (193–253)
Brunei Darussalam	Male	1.25 (1.05–1.47)	0.0954 (0.0805–0.113)	0.0419 (0.0354–0.0492)	0.0534 (0.0449–0.0632)	15100 (13400–17200)	1250 (1100–1410)	670 (595–761)	576 (503–664)
Japan	Female	278 (255–299)	3.26 (2.97–3.52)	0.335 (0.307–0.363)	2.92 (2.66–3.16)	4080 (4000–4160)	90.3 (87.5–93.4)	13.2 (12.6–13.8)	77.2 (74.6–80)
Japan	Male	343 (316–369)	14.1 (12.9–15.2)	2.4 (2.2–2.66)	11.7 (10.7–12.6)	7520 (7380–7680)	438 (425–455)	74.8 (71.9–78)	363 (352–377)
Republic of Korea	Female	91.3 (84.5–97.7)	1.97 (1.77–2.2)	1.06 (0.945–1.2)	0.907 (0.815–1.01)	4140 (3970–4320)	104 (96.4–114)	52.2 (47.8–57.5)	52.3 (48.6–56.7)
Republic of Korea	Male	129 (119–138)	8.93 (8.07–9.93)	2.95 (2.66–3.29)	5.98 (5.41–6.65)	7560 (7310–7820)	627 (589–676)	215 (200–235)	412 (387–444)
Singapore	Female	8.53 (7.85–9.23)	0.105 (0.0955–0.114)	0.0158 (0.0141–0.0179)	0.0889 (0.0809–0.0972)	3730 (3570–3900)	57.8 (55.2–60.7)	7.69 (7.32–8.09)	50.1 (47.9–52.8)
Singapore	Male	11.4 (10.5–12.4)	0.576 (0.526–0.631)	0.147 (0.134–0.161)	0.429 (0.392–0.47)	5510 (5310–5780)	322 (307–338)	82.7 (78.6–87.6)	239 (228–251)
High-income North America	Female	989 (926–1060)	39.9 (37.1–42.6)	12.4 (11.5–13.3)	27.4 (25.5–29.4)	8840 (8530–9180)	446 (432–464)	145 (140–152)	301 (292–313)
High-income North America	Male	1220 (1150–1300)	53.9 (50.1–57.9)	21.6 (20.1–23.3)	32.2 (30–34.6)	13900 (13500–14300)	708 (682–735)	281 (271–293)	426 (411–442)
Canada	Female	86.6 (81.1–92.6)	3.02 (2.8–3.28)	0.804 (0.741–0.868)	2.22 (2.06–2.42)	6210 (5970–6470)	282 (270–297)	81 (76.9–85.3)	201 (193–212)
Canada	Male	103 (96.5–110)	4.22 (3.9–4.6)	1.58 (1.46–1.73)	2.64 (2.44–2.88)	9430 (9110–9770)	464 (446–485)	175 (168–183)	289 (278–303)
Greenland	Female	0.141 (0.124–0.16)	0.0125 (0.011–0.0142)	0.00499 (0.00435–0.00569)	0.00752 (0.00658–0.00853)	11400 (10200–12900)	1090 (969–1260)	443 (393–514)	647 (577–749)
Greenland	Male	0.232 (0.202–0.263)	0.0171 (0.0148–0.0197)	0.006 (0.00516–0.00698)	0.0111 (0.0096–0.0127)	18500 (16600–21200)	1530 (1330–1780)	539 (471–626)	992 (864–1160)
United States of America	Female	903 (837–969)	36.8 (34–39.6)	11.6 (10.7–12.5)	25.2 (23.3–27.1)	9180 (8850–9540)	468 (452–487)	154 (147–161)	314 (304–327)
United States of America	Male	1120 (1040–1200)	49.6 (45.9–53.5)	20.1 (18.6–21.7)	29.6 (27.4–31.9)	14400 (14000–14900)	739 (709–770)	295 (283–308)	444 (427–461)
Southern Latin America	Female	150 (136–167)	5.12 (4.53–5.81)	2.15 (1.88–2.47)	2.96 (2.64–3.34)	8580 (8180–9030)	349 (329–371)	149 (140–160)	200 (190–211)
Southern Latin America	Male	189 (171–209)	9.2 (8.2–10.3)	3.78 (3.33–4.26)	5.43 (4.86–6.04)	13600 (13100–14200)	723 (687–758)	289 (272–306)	433 (413–453)
Argentina	Female	104 (90.6–121)	4.22 (3.64–4.9)	1.88 (1.63–2.2)	2.33 (2.02–2.71)	9460 (8990–9970)	442 (416–471)	198 (185–213)	245 (231–260)
Argentina	Male	131 (113–151)	7.35 (6.33–8.47)	3.2 (2.74–3.69)	4.16 (3.59–4.79)	14900 (14200–15600)	902 (860–945)	377 (357–397)	524 (501–548)
Chile	Female	37.5 (34–41.3)	0.691 (0.618–0.764)	0.197 (0.176–0.222)	0.493 (0.442–0.544)	6570 (6310–6850)	165 (156–174)	51.9 (48.3–56.1)	113 (107–118)
Chile	Male	47.1 (42.9–51.9)	1.17 (1.06–1.3)	0.295 (0.265–0.332)	0.879 (0.791–0.97)	10700 (10200–11100)	303 (287–317)	79 (73.2–84.9)	224 (213–234)
Uruguay	Female	8.73 (7.53–9.91)	0.208 (0.178–0.238)	0.0711 (0.0602–0.0816)	0.137 (0.117–0.156)	9080 (8690–9500)	298 (282–314)	109 (102–116)	189 (180–199)
Uruguay	Male	11.2 (9.67–12.7)	0.677 (0.578–0.77)	0.287 (0.246–0.328)	0.39 (0.333–0.444)	16600 (16000–17400)	1150 (1090–1210)	473 (448–499)	673 (639–705)
Western Europe	Female	983 (945–1020)	26 (24.8–27.1)	6.34 (6.07–6.61)	19.6 (18.8–20.5)	5470 (5370–5600)	230 (224–236)	61.7 (60–63.5)	168 (164–173)
Western Europe	Male	1160 (1120–1200)	61.3 (58.6–64)	18.7 (17.9–19.6)	42.6 (40.8–44.4)	8680 (8530–8840)	599 (584–618)	186 (181–193)	412 (402–425)
Andorra	Female	0.155 (0.127–0.189)	0.00368 (0.00279–0.00469)	0.000667 (0.000479–0.000887)	0.00302 (0.00231–0.0038)	4590 (3420–5910)	155 (110–207)	34.5 (23.7–47.1)	120 (86.3–160)
Andorra	Male	0.229 (0.178–0.284)	0.0164 (0.0124–0.0204)	0.00533 (0.00413–0.0065)	0.0111 (0.00821–0.0139)	7760 (5570–10100)	651 (459–866)	215 (153–282)	437 (306–587)
Austria	Female	19.2 (17.3–21)	0.475 (0.427–0.522)	0.111 (0.0987–0.123)	0.364 (0.327–0.401)	5070 (4890–5250)	204 (194–215)	54.8 (51.8–58.1)	149 (142–158)
Austria	Male	23 (20.7–25.1)	1.26 (1.13–1.38)	0.312 (0.28–0.343)	0.944 (0.853–1.04)	8220 (7930–8510)	571 (545–600)	147 (139–155)	424 (405–445)
Belgium	Female	25.9 (23.5–28.5)	0.696 (0.627–0.78)	0.139 (0.125–0.156)	0.558 (0.501–0.624)	5870 (5670–6110)	246 (234–259)	56.6 (53.4–60.3)	189 (179–199)
Belgium	Male	31 (28.1–34)	1.72 (1.54–1.92)	0.558 (0.498–0.624)	1.16 (1.04–1.3)	9120 (8830–9420)	643 (614–675)	211 (201–222)	432 (412–454)
Cyprus	Female	2.6 (2.24–2.94)	0.0516 (0.0434–0.06)	0.00859 (0.00727–0.0102)	0.043 (0.0362–0.0499)	5640 (5150–6180)	143 (126–160)	26.6 (23.2–30.3)	116 (103–130)
Cyprus	Male	3.19 (2.74–3.68)	0.239 (0.201–0.281)	0.0879 (0.0742–0.103)	0.151 (0.127–0.177)	8680 (7750–9770)	826 (717–940)	321 (280–363)	506 (436–579)
Denmark	Female	13.7 (12.5–15)	0.663 (0.602–0.727)	0.224 (0.203–0.244)	0.439 (0.398–0.483)	6150 (5930–6390)	428 (404–451)	145 (137–153)	282 (268–298)
Denmark	Male	15.8 (14.5–17.3)	0.913 (0.822–1.01)	0.42 (0.377–0.467)	0.493 (0.443–0.546)	8840 (8550–9150)	687 (654–728)	315 (298–336)	372 (355–392)
Finland	Female	12.6 (11.3–13.9)	0.192 (0.17–0.212)	0.0384 (0.0338–0.0424)	0.154 (0.136–0.17)	5420 (5230–5620)	145 (137–154)	35.5 (33.3–37.8)	109 (103–116)
Finland	Male	15.6 (13.9–17)	0.549 (0.489–0.615)	0.137 (0.121–0.153)	0.413 (0.367–0.462)	9390 (9070–9720)	435 (412–460)	114 (108–121)	321 (304–339)
France	Female	138 (124–153)	3.22 (2.81–3.67)	0.804 (0.699–0.925)	2.42 (2.11–2.73)	5050 (4920–5200)	207 (193–223)	61.3 (56.6–67)	146 (137–157)
France	Male	170 (153–188)	9.18 (8.12–10.2)	2.47 (2.18–2.76)	6.71 (5.94–7.47)	9040 (8840–9250)	643 (599–682)	181 (168–194)	463 (432–490)
Germany	Female	214 (192–235)	5.49 (4.85–6.08)	1.06 (0.935–1.18)	4.43 (3.92–4.91)	6070 (5940–6200)	258 (247–268)	59.5 (56.1–62.8)	198 (190–207)
Germany	Male	255 (228–280)	12.8 (11.4–14.2)	3.38 (3.01–3.72)	9.46 (8.41–10.4)	9500 (9330–9700)	654 (628–683)	182 (174–191)	473 (454–493)
Greece	Female	26 (22.4–29.5)	0.859 (0.736–0.982)	0.253 (0.215–0.293)	0.606 (0.521–0.691)	6200 (6000–6420)	353 (336–370)	107 (101–114)	246 (234–258)
Greece	Male	30.9 (26.5–35)	2.75 (2.36–3.14)	0.93 (0.793–1.07)	1.82 (1.56–2.07)	10700 (10400–11000)	1210 (1160–1280)	413 (392–435)	801 (766–841)
Iceland	Female	0.644 (0.584–0.712)	0.0206 (0.0182–0.0232)	0.00335 (0.00294–0.00382)	0.0173 (0.0153–0.0194)	5140 (4790–5560)	232 (213–253)	41.6 (37.8–46.1)	191 (175–208)
Iceland	Male	0.809 (0.73–0.89)	0.0345 (0.0304–0.0384)	0.0141 (0.0125–0.0157)	0.0203 (0.018–0.0226)	7650 (7130–8270)	399 (368–436)	162 (150–177)	237 (219–259)
Ireland	Female	8.95 (7.97–9.83)	0.357 (0.314–0.397)	0.0872 (0.0767–0.0974)	0.269 (0.238–0.3)	5050 (4860–5260)	270 (253–288)	67.3 (63.1–72)	203 (190–216)
Ireland	Male	10.4 (9.3–11.4)	0.486 (0.429–0.535)	0.147 (0.13–0.162)	0.339 (0.3–0.374)	7100 (6820–7400)	411 (391–433)	125 (118–132)	287 (272–302)
Israel	Female	13.8 (12–16)	0.249 (0.211–0.291)	0.0573 (0.0486–0.0671)	0.192 (0.162–0.225)	4640 (4460–4850)	125 (118–133)	30.4 (28.5–32.5)	94.6 (89.3–101)
Israel	Male	17.1 (14.7–19.7)	0.765 (0.644–0.895)	0.21 (0.175–0.244)	0.555 (0.467–0.649)	7240 (6910–7590)	410 (387–436)	111 (104–117)	299 (282–318)
Italy	Female	139 (126–151)	2.84 (2.56–3.13)	0.461 (0.412–0.512)	2.38 (2.14–2.62)	4860 (4750–4970)	172 (165–180)	36.4 (34.5–38.5)	136 (130–142)
Italy	Male	157 (142–171)	8.14 (7.32–9.05)	2.62 (2.36–2.91)	5.52 (4.97–6.12)	7680 (7520–7840)	529 (509–552)	173 (166–181)	356 (342–372)
Luxembourg	Female	1.28 (1.16–1.41)	0.0424 (0.0375–0.047)	0.00972 (0.00853–0.0108)	0.0327 (0.029–0.0362)	5200 (4830–5630)	224 (204–245)	52.7 (47.7–57.9)	171 (156–187)
Luxembourg	Male	1.64 (1.47–1.82)	0.107 (0.0937–0.121)	0.0402 (0.0352–0.0454)	0.0669 (0.0585–0.0755)	7990 (7320–8700)	621 (562–685)	226 (205–250)	395 (357–434)
Malta	Female	1.02 (0.882–1.16)	0.0199 (0.017–0.0229)	0.00378 (0.00322–0.00435)	0.0161 (0.0137–0.0186)	5650 (5160–6190)	179 (162–197)	40.6 (36.5–45)	139 (126–153)
Malta	Male	1.16 (1–1.32)	0.0548 (0.0474–0.0632)	0.0163 (0.0142–0.0189)	0.0385 (0.0333–0.0442)	8050 (7360–8870)	497 (455–543)	156 (143–171)	340 (313–371)
Monaco	Female	0.113 (0.0973–0.129)	0.0037 (0.00302–0.00445)	0.00088 (0.000704–0.00107)	0.00282 (0.00232–0.00339)	7800 (6470–9240)	418 (336–514)	120 (94.6–152)	298 (241–365)
Monaco	Male	0.129 (0.114–0.146)	0.00819 (0.0069–0.00957)	0.00318 (0.00269–0.0037)	0.00501 (0.0042–0.00587)	11700 (10200–13500)	999 (854–1170)	393 (334–462)	606 (519–708)
Netherlands	Female	41.2 (37.2–45.1)	1.43 (1.28–1.6)	0.448 (0.4–0.502)	0.985 (0.88–1.1)	6320 (6140–6490)	325 (308–341)	106 (99.6–112)	219 (208–229)
Netherlands	Male	45.2 (40.8–49.5)	2.23 (1.99–2.46)	0.858 (0.765–0.945)	1.38 (1.22–1.52)	8320 (8070–8560)	559 (534–582)	221 (211–233)	337 (323–352)
Norway	Female	10.9 (10–12)	0.13 (0.118–0.144)	0.0173 (0.0154–0.0193)	0.113 (0.102–0.125)	5380 (5230–5510)	101 (96.4–106)	15.5 (14.6–16.6)	85.7 (81.6–90)
Norway	Male	12.8 (11.6–13.9)	0.262 (0.237–0.288)	0.042 (0.0379–0.0465)	0.22 (0.199–0.242)	7390 (7210–7590)	210 (201–221)	38.9 (36.9–41.3)	172 (164–180)
Portugal	Female	24.5 (21.6–27.6)	0.257 (0.225–0.29)	0.0643 (0.0562–0.0726)	0.193 (0.167–0.218)	4880 (4720–5040)	109 (104–115)	33.7 (31.7–36)	75.6 (71.9–79.6)
Portugal	Male	29.7 (26–33.5)	1.34 (1.16–1.52)	0.308 (0.269–0.346)	1.03 (0.893–1.17)	9130 (8870–9370)	576 (549–603)	146 (138–154)	430 (411–450)
San Marino	Female	0.06 (0.0458–0.0745)	0.00108 (0.000746–0.00144)	0.000162 (0.000109–0.000223)	0.000923 (0.000636–0.00122)	3770 (2520–5280)	111 (71–158)	22.3 (14.1–32.5)	88.7 (56.8–126)
San Marino	Male	0.0677 (0.0524–0.0846)	0.00354 (0.00253–0.0046)	0.00133 (0.001–0.00167)	0.00221 (0.00152–0.00293)	5610 (3850–7690)	374 (249–540)	134 (90.4–192)	240 (158–348)

Supplementary Results Table S2. Cumulative all-age counts and age-standardised rates of years of life lost (YLLs) between 2022 to 2050 by location, sex, and scenario

		All-Age Count (Millions)				Age-Standardized Rate (per 100,000)			
Location	Sex	Reference	Difference between Reference and Elimination - 2023	Difference between Reference and Elimination - 2050	Difference between Elimination - 2050 and Elimination - 2023	Reference	Difference between Reference and Elimination - 2023	Difference between Reference and Elimination - 2050	Difference between Elimination - 2050 and Elimination - 2023
Spain	Female	95.4 (85.1–104)	1.34 (1.19–1.47)	0.27 (0.239–0.299)	1.07 (0.95–1.18)	4520 (4400–4640)	122 (115–129)	29.2 (27.3–31.2)	92.3 (87.1–98.5)
Spain	Male	120 (107–132)	7.27 (6.4–8.06)	1.74 (1.55–1.93)	5.53 (4.86–6.13)	7970 (7810–8140)	618 (594–645)	154 (148–161)	464 (446–484)
Sweden	Female	21.4 (19.2–23.9)	0.736 (0.645–0.833)	0.272 (0.238–0.311)	0.463 (0.407–0.521)	5070 (4590–5600)	261 (234–292)	102 (90.4–115)	160 (143–178)
Sweden	Male	24.4 (21.6–27.6)	0.913 (0.796–1.05)	0.458 (0.399–0.526)	0.455 (0.398–0.52)	7020 (6230–7820)	346 (303–393)	171 (150–194)	174 (153–198)
Switzerland	Female	17.2 (15.6–18.9)	0.54 (0.487–0.597)	0.126 (0.113–0.141)	0.414 (0.371–0.46)	4480 (4330–4640)	206 (195–217)	53.1 (49.8–57)	153 (145–161)
Switzerland	Male	19.9 (18.1–21.8)	1.06 (0.952–1.18)	0.362 (0.323–0.404)	0.7 (0.627–0.775)	6510 (6290–6740)	436 (414–462)	146 (138–156)	290 (276–307)
United Kingdom	Female	155 (146–164)	6.35 (5.96–6.72)	1.87 (1.76–1.99)	4.47 (4.2–4.74)	6480 (6300–6660)	362 (349–374)	108 (104–112)	254 (245–263)
United Kingdom	Male	178 (169–187)	9.13 (8.51–9.71)	3.57 (3.32–3.8)	5.56 (5.18–5.92)	9300 (9070–9540)	603 (581–623)	236 (227–245)	367 (354–380)
Latin America and Caribbean	Female	1450 (1280–1660)	24 (21–28.3)	8.86 (7.62–10.6)	15.1 (13.4–17.6)	11300 (9910–13300)	198 (174–234)	72.5 (63.5–86.8)	126 (110–148)
Latin America and Caribbean	Male	2010 (1820–2240)	57.6 (51.6–65)	26.1 (23.1–29.9)	31.5 (28.5–35.1)	18600 (17000–20700)	579 (524–660)	255 (227–293)	324 (296–367)
Andean Latin America	Female	140 (119–162)	0.973 (0.82–1.12)	0.332 (0.272–0.394)	0.641 (0.549–0.737)	10800 (9130–12900)	81 (67.9–95.8)	26.9 (22–32.5)	54.1 (45.8–63.3)
Andean Latin America	Male	176 (149–203)	3.65 (3.14–4.17)	1.55 (1.32–1.81)	2.09 (1.81–2.38)	14600 (12300–17400)	339 (286–403)	140 (116–167)	200 (170–236)
Bolivia (Plurinational State of)	Female	34.6 (27.6–42.8)	0.287 (0.221–0.365)	0.158 (0.119–0.206)	0.129 (0.102–0.159)	17200 (13800–21900)	155 (117–203)	77.3 (57.4–104)	77.5 (59.5–100)
Bolivia (Plurinational State of)	Male	38.9 (30.8–48.1)	1.07 (0.831–1.34)	0.598 (0.452–0.763)	0.47 (0.377–0.577)	20000 (16000–25400)	656 (505–853)	333 (254–435)	324 (254–418)
Ecuador	Female	37.3 (30.5–44.1)	0.318 (0.245–0.393)	0.102 (0.0767–0.127)	0.216 (0.168–0.265)	10200 (8470–12200)	97.6 (77.9–122)	31.1 (24.7–39.2)	66.5 (53.2–82.5)
Ecuador	Male	50.8 (39.8–61.6)	1.13 (0.876–1.41)	0.445 (0.34–0.562)	0.687 (0.534–0.845)	15500 (12600–18900)	395 (307–504)	149 (115–191)	246 (191–313)
Peru	Female	68.2 (55.7–82.7)	0.368 (0.291–0.455)	0.072 (0.0567–0.0907)	0.296 (0.235–0.364)	9240 (7500–11400)	53.9 (42.3–67.9)	10.7 (8.32–13.6)	43.2 (34–54.1)
Peru	Male	86 (70–104)	1.44 (1.19–1.76)	0.511 (0.424–0.614)	0.934 (0.764–1.14)	12700 (10200–15600)	237 (191–294)	84.5 (68.1–105)	153 (123–190)
Caribbean	Female	155 (131–186)	2.7 (2.35–3.08)	1.09 (0.93–1.28)	1.61 (1.42–1.81)	18000 (14900–21800)	305 (264–364)	123 (104–149)	181 (159–214)
Caribbean	Male	192 (164–226)	6.69 (5.93–7.49)	3.05 (2.65–3.45)	3.64 (3.25–4.02)	24000 (20100–28600)	867 (758–1000)	382 (331–447)	485 (426–551)
Antigua and Barbuda	Female	0.248 (0.215–0.283)	0.00369 (0.00318–0.00426)	0.00135 (0.00115–0.00156)	0.00235 (0.00203–0.00271)	11600 (10800–12800)	191 (176–213)	70.8 (64.6–79.4)	120 (111–133)
Antigua and Barbuda	Male	0.272 (0.236–0.309)	0.00893 (0.0077–0.0103)	0.00442 (0.00378–0.00511)	0.00451 (0.0039–0.00515)	14800 (13900–16100)	545 (504–598)	265 (245–291)	280 (261–306)
Bahamas	Female	1.15 (0.922–1.41)	0.0122 (0.00967–0.0149)	0.00531 (0.00423–0.00648)	0.00685 (0.00544–0.00838)	13100 (10600–16100)	148 (118–183)	65.4 (52.2–81)	82.4 (66–102)
Bahamas	Male	1.57 (1.24–1.93)	0.0478 (0.0384–0.0576)	0.0263 (0.0211–0.0319)	0.0215 (0.0173–0.0256)	21800 (17600–26700)	720 (575–898)	392 (313–489)	328 (262–410)
Barbados	Female	0.979 (0.799–1.18)	0.00371 (0.00292–0.00472)	0.000606 (0.000481–0.000765)	0.0031 (0.00243–0.00395)	12200 (9800–15200)	62.9 (49.1–80.5)	11.7 (9.19–15)	51.2 (39.9–65.6)
Barbados	Male	0.985 (0.79–1.21)	0.0242 (0.0196–0.0292)	0.0101 (0.00845–0.0118)	0.0142 (0.0113–0.0174)	14800 (11500–18700)	436 (345–539)	185 (151–225)	251 (195–315)
Belize	Female	1.03 (0.845–1.23)	0.0145 (0.012–0.0176)	0.0064 (0.00523–0.0078)	0.00813 (0.00672–0.0098)	12300 (10700–14500)	185 (157–222)	78.9 (66.3–95)	106 (90.5–127)
Belize	Male	1.57 (1.32–1.85)	0.058 (0.0473–0.0688)	0.0307 (0.0248–0.0369)	0.0273 (0.0225–0.0321)	19600 (17100–22500)	862 (728–1010)	430 (362–508)	432 (366–499)
Bermuda	Female	0.137 (0.117–0.161)	0.00255 (0.00213–0.00307)	0.000904 (0.000752–0.00109)	0.00165 (0.00138–0.00199)	5210 (4480–6350)	128 (108–161)	51.2 (43–64.2)	77.2 (65.4–96.9)
Bermuda	Male	0.195 (0.168–0.225)	0.0086 (0.00733–0.00994)	0.00364 (0.0031–0.00421)	0.00496 (0.00423–0.00575)	10800 (9270–12700)	580 (498–685)	250 (214–295)	330 (283–391)
Cuba	Female	29.7 (25.9–33.9)	0.927 (0.792–1.08)	0.267 (0.225–0.316)	0.66 (0.566–0.762)	8340 (7420–9470)	346 (303–402)	108 (93–127)	237 (209–275)
Cuba	Male	38.8 (33–44.5)	2.64 (2.24–3.06)	0.945 (0.793–1.12)	1.69 (1.45–1.95)	13800 (12000–15600)	1120 (957–1290)	399 (340–468)	718 (619–823)
Dominica	Female	0.204 (0.168–0.249)	0.00197 (0.00161–0.00239)	0.000453 (0.000368–0.00055)	0.00152 (0.00124–0.00184)	16700 (13900–20700)	165 (137–201)	39.2 (32.5–47.9)	126 (104–153)
Dominica	Male	0.282 (0.233–0.341)	0.00824 (0.00686–0.00985)	0.00404 (0.00335–0.00484)	0.00421 (0.00351–0.00502)	24100 (20000–29500)	741 (632–885)	358 (305–426)	383 (328–459)
Dominican Republic	Female	25.1 (20.2–30.9)	0.871 (0.697–1.07)	0.488 (0.389–0.603)	0.382 (0.307–0.47)	12200 (10200–14900)	458 (377–569)	254 (209–318)	204 (168–252)
Dominican Republic	Male	37.8 (30.6–46.3)	1.67 (1.34–2.06)	1.06 (0.848–1.31)	0.612 (0.491–0.755)	19600 (16500–23400)	956 (762–1220)	591 (473–753)	364 (291–464)
Grenada	Female	0.295 (0.249–0.341)	0.00306 (0.0026–0.00358)	0.001 (0.00084–0.0012)	0.00205 (0.00174–0.00238)	14500 (12600–16700)	170 (146–197)	56.8 (48.6–67.4)	113 (98.2–130)
Grenada	Male	0.396 (0.333–0.457)	0.0115 (0.00968–0.0134)	0.00481 (0.00396–0.00576)	0.00668 (0.00571–0.00772)	21400 (18600–24900)	707 (608–827)	282 (240–335)	424 (366–492)
Guyana	Female	2.15 (1.65–2.69)	0.0202 (0.0156–0.0251)	0.00899 (0.0068–0.0114)	0.0112 (0.00879–0.0136)	18100 (14000–23000)	189 (144–243)	83.7 (63–108)	106 (80.8–135)
Guyana	Male	3.13 (2.39–3.93)	0.082 (0.0637–0.101)	0.0298 (0.0216–0.0385)	0.0522 (0.0416–0.0635)	30300 (23300–38100)	925 (681–1200)	329 (244–429)	596 (441–769)
Haiti	Female	68.1 (48–91.7)	0.431 (0.297–0.599)	0.202 (0.137–0.287)	0.229 (0.159–0.315)	33200 (24900–43800)	254 (177–363)	118 (81.8–170)	136 (94.8–193)
Haiti	Male	73.3 (51.4–100)	0.925 (0.644–1.25)	0.455 (0.307–0.634)	0.47 (0.338–0.62)	36700 (27600–49400)	602 (412–830)	280 (191–392)	322 (223–440)
Jamaica	Female	6.96 (5.46–8.61)	0.106 (0.0825–0.133)	0.0261 (0.0199–0.0331)	0.0795 (0.0626–0.099)	12500 (9580–16000)	199 (152–255)	49 (37–63.7)	150 (114–191)
Jamaica	Male	8.16 (6.41–10.1)	0.326 (0.256–0.406)	0.12 (0.0925–0.151)	0.207 (0.164–0.255)	16000 (12300–20500)	693 (517–899)	246 (184–321)	447 (333–583)
Puerto Rico	Female	7.6 (6.66–8.71)	0.125 (0.109–0.144)	0.0251 (0.0215–0.0292)	0.1 (0.0874–0.115)	6620 (5730–7660)	135 (116–158)	32.3 (27.4–38.5)	103 (88.6–119)
Puerto Rico	Male	9.74 (8.43–11.1)	0.314 (0.271–0.357)	0.131 (0.113–0.15)	0.182 (0.158–0.208)	13500 (11500–15700)	454 (381–531)	189 (158–222)	265 (223–310)
Saint Kitts and Nevis	Female	0.16 (0.125–0.194)	0.00143 (0.00113–0.00174)	0.00052 (0.000416–0.000634)	0.000906 (0.000717–0.00111)	12400 (10200–14800)	117 (96.7–140)	44.1 (36.6–52.4)	72.9 (60–87.4)
Saint Kitts and Nevis	Male	0.236 (0.186–0.287)	0.00495 (0.00391–0.00603)	0.00192 (0.00152–0.00231)	0.00304 (0.00238–0.00372)	21000 (17800–24300)	495 (421–575)	210 (179–241)	285 (239–335)
Saint Lucia	Female	0.457 (0.378–0.552)	0.00523 (0.0043–0.00634)	0.00167 (0.00137–0.00203)	0.00356 (0.00294–0.0043)	11100 (8910–13900)	127 (104–157)	41.7 (34.1–51.6)	85.7 (70.2–105)
Saint Lucia	Male	0.667 (0.55–0.799)	0.021 (0.0171–0.025)	0.00731 (0.00595–0.0088)	0.0136 (0.0112–0.0162)	18800 (15200–23200)	602 (477–741)	215 (170–266)	387 (306–476)
Saint Vincent and the Grenadines	Female	0.315 (0.262–0.372)	0.0029 (0.00238–0.00347)	0.00117 (0.000966–0.0014)	0.00173 (0.00141–0.00208)	14300 (12100–16900)	146 (123–173)	60.1 (50.7–71.1)	86 (72–102)
Saint Vincent and the Grenadines	Male	0.458 (0.391–0.533)	0.0126 (0.0105–0.0148)	0.00504 (0.00412–0.00603)	0.00758 (0.00637–0.00885)	21900 (19000–25100)	675 (582–781)	268 (227–314)	407 (356–468)
Suriname	Female	1.72 (1.38–2.09)	0.0286 (0.0229–0.0355)	0.00733 (0.00568–0.0094)	0.0213 (0.0172–0.0262)	14600 (11800–18000)	252 (200–319)	66.9 (52–86.4)	185 (147–233)
Suriname	Male	2.33 (1.84–2.88)	0.0939 (0.0746–0.116)	0.0305 (0.0234–0.0393)	0.0633 (0.0513–0.077)	22300 (17500–27900)	978 (738–1260)	306 (230–401)	672 (513–857)
Trinidad and Tobago	Female	3.61 (2.87–4.43)	0.0459 (0.0358–0.0576)	0.0106 (0.00807–0.0135)	0.0353 (0.0277–0.0442)	11900 (9430–14700)	168 (128–213)	41.6 (31.2–53.3)	127 (97.2–160)
Trinidad and Tobago	Male	5.26 (4.08–6.44)	0.202 (0.155–0.251)	0.0768 (0.0573–0.0983)	0.125 (0.0978–0.153)	20700 (16500–25600)	811 (612–1030)	307 (228–396)	504 (382–635)
United States Virgin Islands	Female	0.22 (0.192–0.253)	0.00284 (0.00234–0.00345)	0.000792 (0.000599–0.00116)	0.00205 (0.00173–0.00246)	7360 (6080–8880)	127 (101–158)	39 (30.7–50.4)	87.6 (70.3–109)
United States Virgin Islands	Male	0.33 (0.284–0.384)	0.00747 (0.0063–0.00893)	0.00386 (0.00327–0.00459)	0.00361 (0.00303–0.00435)	19400 (16200–23500)	438 (349–556)	226 (180–285)	212 (168–271)
Central Latin America	Female	609 (526–724)	6.83 (5.62–8.85)	1.51 (1.15–2.15)	5.32 (4.46–6.72)	11400 (9620–14100)	138 (110–185)	30.4 (22.7–43.7)	108 (87–141)
Central Latin America	Male	868 (775–996)	19.6 (17.2–23.6)	6.92 (5.88–8.71)	12.7 (11.3–14.8)	19400 (17000–22700)	490 (412–609)	166 (136–214)	324 (275–395)
Colombia	Female	91.9 (77.8–107)	1.12 (0.935–1.33)	0.221 (0.179–0.27)	0.895 (0.753–1.05)	7780 (6600–9220)	101 (85.3–120)	21.4 (17.6–25.9)	79.8 (67.4–94.9)
Colombia	Male	125 (106–145)	2.42 (2.06–2.86)	0.553 (0.465–0.661)	1.87 (1.59–2.2)	13100 (11100–15300)	261 (218–318)	59.5 (49.3–72.9)	201 (167–245)
Costa Rica	Female	9.15 (7.87–10.6)	0.114 (0.0974–0.132)	0.019 (0.0162–0.0224)	0.0946 (0.0812–0.11)	7740 (6940–8630)	101 (89.4–114)	17.6 (15.4–20.2)	83.4 (73.9–94.1)
Costa Rica	Male	13.2 (11.3–15.2)	0.382 (0.327–0.446)	0.168 (0.144–0.196)	0.215 (0.183–0.25)	14100 (12600–15700)	432 (382–486)	187 (165–212)	244 (215–275)
El Salvador	Female	14.1 (10.9–17.5)	0.167 (0.125–0.207)	0.075 (0.0561–0.0949)	0.0916 (0.07–0.113)	10800 (8900–13200)	143 (113–178)	64 (50.2–79.9)	78.6 (62.6–98)
El Salvador	Male	20.4 (15.7–25.2)	0.417 (0.312–0.529)	0.195 (0.143–0.252)	0.222 (0.17–0.278)	22200 (18600–26400)	521 (418–643)	240 (191–301)	281 (228–346)
Guatemala	Female	37.4 (31.6–44.1)	0.312 (0.258–0.374)	0.123 (0.102–0.148)	0.188 (0.156–0.225)	13500 (11400–16000)	127 (105–154)	49.9 (41.3–60.4)	77.2 (63.9–93.1)
Guatemala	Male	51.5 (43.5–60.2)	1.01 (0.845–1.2)	0.446 (0.371–0.538)	0.563 (0.471–0.661)	20800 (17700–24300)	490 (409–583)	214 (178–257)	276 (231–328)
Honduras	Female	28 (21.6–35.4)	0.418 (0.313–0.536)	0.175 (0.127–0.23)	0.243 (0.186–0.307)	16800 (13400–21100)	280 (215–366)	107 (79.8–143)	173 (135–225)
Honduras	Male	30.8 (24.3–38)	1.13 (0.889–1.41)	0.574 (0.45–0.721)	0.556 (0.439–0.69)	20600 (17400–25100)	1010 (833–1250)	505 (415–625)	508 (418–625)
Mexico	Female	321 (275–370)	2.98 (2.51–3.41)	0.43 (0.351–0.529)	2.55 (2.16–2.9)	11700 (10200–13700)	124 (103–145)	17.9 (14.5–21.6)	106 (88.9–124)
Mexico	Male	469 (405–537)	9.87 (8.5–11.3)	3.23 (2.72–3.82)	6.63 (5.76–7.53)	20200 (17600–23100)	491 (403–585)	151 (122–183)	339 (279–401)

Supplementary Results Table S2. Cumulative all-age counts and age-standardised rates of years of life lost (YLLs) between 2022 to 2050 by location, sex, and scenario

Location	Sex	All-Age Count (Millions)				Age-Standardized Rate (per 100,000)			
		Reference	Difference between Reference and Elimination - 2023	Difference between Reference and Elimination - 2050	Difference between Elimination - 2050 and Elimination - 2023	Reference	Difference between Reference and Elimination - 2023	Difference between Reference and Elimination - 2050	Difference between Elimination - 2050 and Elimination - 2023
Nicaragua	Female	11.6 (9–14.4)	0.0916 (0.0676–0.12)	0.0253 (0.018–0.0342)	0.0663 (0.0491–0.0868)	8970 (7470–10600)	75.2 (59.4–95.3)	20.4 (15.8–26.4)	54.8 (43.4–68.9)
Nicaragua	Male	15.9 (12.3–20.1)	0.456 (0.354–0.589)	0.215 (0.167–0.278)	0.241 (0.187–0.308)	14100 (12000–17000)	476 (389–589)	228 (186–281)	248 (203–310)
Panama	Female	8.25 (6.72–9.9)	0.0921 (0.0758–0.11)	0.0303 (0.025–0.036)	0.0618 (0.0508–0.0734)	8700 (7270–10300)	102 (84.9–121)	33.1 (27.6–39)	69.1 (57.3–82.3)
Panama	Male	11.7 (9.34–14.2)	0.24 (0.191–0.289)	0.0746 (0.0594–0.0916)	0.165 (0.132–0.198)	13400 (11000–16000)	304 (242–369)	91.8 (72.8–112)	212 (169–257)
Venezuela (Bolivarian Republic of)	Female	87.6 (56.8–149)	1.55 (0.868–3.06)	0.414 (0.213–0.88)	1.14 (0.656–2.18)	17600 (10300–34000)	328 (159–733)	90 (41.2–210)	238 (119–522)
Venezuela (Bolivarian Republic of)	Male	131 (93.5–198)	3.7 (2.42–5.97)	1.46 (0.908–2.54)	2.24 (1.51–3.46)	31900 (21200–54100)	1020 (567–1980)	396 (214–788)	619 (353–1180)
Tropical Latin America	Female	541 (461–631)	13.5 (11.4–15.8)	5.92 (4.96–7.03)	7.55 (6.42–8.83)	10100 (9290–11100)	268 (244–302)	118 (106–133)	150 (138–168)
Tropical Latin America	Male	771 (665–884)	27.7 (23.7–31.9)	14.6 (12.4–16.9)	13.1 (11.2–15.1)	17800 (16900–18900)	676 (633–738)	348 (326–383)	327 (307–356)
Brazil	Female	526 (445–615)	13.1 (11.1–15.3)	5.74 (4.79–6.83)	7.35 (6.22–8.62)	10000 (9240–11000)	267 (243–301)	117 (106–133)	150 (137–167)
Brazil	Male	749 (646–861)	26.6 (22.7–30.8)	14 (12–16.3)	12.6 (10.7–14.6)	17700 (16900–18900)	665 (624–728)	343 (321–378)	322 (303–350)
Paraguay	Female	14.6 (10.9–18.7)	0.38 (0.289–0.476)	0.184 (0.14–0.23)	0.197 (0.149–0.247)	11300 (9030–14200)	339 (269–424)	163 (130–205)	176 (139–219)
Paraguay	Male	22.5 (17–29)	1.1 (0.839–1.4)	0.562 (0.425–0.72)	0.54 (0.412–0.686)	18800 (14900–24000)	1110 (867–1420)	545 (426–695)	563 (440–724)
North Africa and Middle East	Female	1490 (1300–1740)	16.9 (14–20.9)	5.57 (4.51–7.01)	11.3 (9.41–14)	13900 (11800–16500)	168 (136–216)	54.2 (43.5–69.8)	114 (92.5–145)
North Africa and Middle East	Male	2070 (1830–2370)	159 (139–183)	73.8 (63.4–87.2)	84.9 (75–95.2)	18000 (15600–20900)	1490 (1270–1750)	688 (579–821)	804 (696–927)
North Africa and Middle East	Female	1490 (1300–1740)	16.9 (14–20.9)	5.57 (4.51–7.01)	11.3 (9.41–14)	13900 (11800–16500)	168 (136–216)	54.2 (43.5–69.8)	114 (92.5–145)
North Africa and Middle East	Male	2070 (1830–2370)	159 (139–183)	73.8 (63.4–87.2)	84.9 (75–95.2)	18000 (15600–20900)	1490 (1270–1750)	688 (579–821)	804 (696–927)
Afghanistan	Female	161 (107–226)	1.09 (0.658–1.71)	0.445 (0.258–0.725)	0.646 (0.4–0.977)	30300 (24100–38400)	332 (229–475)	114 (76–167)	218 (153–307)
Afghanistan	Male	176 (119–245)	5.76 (3.66–8.48)	3.18 (2.01–4.75)	2.57 (1.67–3.73)	29500 (23600–36800)	1790 (1240–2380)	976 (680–1300)	810 (560–1080)
Algeria	Female	85.6 (68.6–105)	0.546 (0.409–0.841)	0.142 (0.106–0.288)	0.404 (0.299–0.548)	12000 (10100–14600)	99.2 (76.4–136)	44.7 (34.6–63.1)	54.4 (41.1–72.8)
Algeria	Male	100 (79.3–123)	7.44 (5.71–9.23)	4.17 (3.22–5.16)	3.27 (2.48–4.11)	12400 (10300–15100)	1020 (797–1270)	598 (470–745)	421 (328–531)
Bahrain	Female	2.37 (2.07–2.78)	0.0498 (0.0424–0.0585)	0.0169 (0.0144–0.0199)	0.0329 (0.028–0.0385)	11200 (9520–13600)	286 (240–350)	104 (87.7–128)	182 (152–221)
Bahrain	Male	4.75 (4.1–5.58)	0.492 (0.426–0.57)	0.306 (0.267–0.354)	0.186 (0.16–0.215)	12900 (11000–15500)	1500 (1290–1750)	993 (854–1160)	506 (428–595)
Egypt	Female	269 (225–321)	1.08 (0.835–1.39)	0.398 (0.298–0.641)	0.683 (0.534–0.853)	21100 (17100–25900)	127 (98.3–159)	65.7 (50.6–81.6)	61.4 (46.6–78.8)
Egypt	Male	375 (310–454)	45.1 (36.8–55.2)	24.7 (19.9–30.8)	20.4 (16.9–24.3)	21800 (17900–26900)	3220 (2560–4050)	1690 (1330–2140)	1530 (1230–1910)
Iran (Islamic Republic of)	Female	144 (126–166)	2.26 (1.89–2.7)	0.837 (0.698–1)	1.42 (1.19–1.7)	7780 (6900–9000)	117 (100–139)	42.5 (36.5–50.4)	74.9 (63.7–89.1)
Iran (Islamic Republic of)	Male	207 (182–236)	15.5 (13.5–17.7)	6.64 (5.76–7.68)	8.87 (7.79–10)	11600 (10500–13400)	861 (776–986)	362 (322–419)	500 (452–563)
Iraq	Female	94.4 (63.8–125)	1.66 (1.09–2.28)	0.916 (0.595–1.28)	0.745 (0.499–1)	13800 (11100–17100)	287 (207–389)	149 (106–205)	138 (101–183)
Iraq	Male	152 (105–200)	10.9 (7.32–14.6)	5.55 (3.77–7.43)	5.32 (3.54–7.15)	21900 (17600–26600)	2030 (1480–2570)	1090 (806–1390)	932 (667–1190)
Jordan	Female	17.5 (14.6–21)	0.416 (0.342–0.524)	0.133 (0.112–0.165)	0.283 (0.23–0.359)	7750 (6420–9510)	218 (170–276)	81.3 (63.1–103)	136 (106–175)
Jordan	Male	25.7 (21.4–31)	2.79 (2.27–3.47)	1.36 (1.1–1.71)	1.44 (1.17–1.78)	9720 (8140–11900)	1110 (900–1420)	525 (424–674)	590 (476–758)
Kuwait	Female	4.49 (3.73–5.38)	0.0531 (0.0436–0.064)	0.00808 (0.00677–0.00956)	0.0451 (0.0368–0.0546)	4800 (4220–5600)	50.5 (43–60.8)	9.42 (7.85–11.5)	41.1 (35–49.5)
Kuwait	Male	10.4 (8.45–12.9)	0.91 (0.735–1.14)	0.297 (0.242–0.371)	0.614 (0.491–0.772)	9640 (7950–11600)	797 (646–976)	294 (240–360)	503 (402–624)
Lebanon	Female	9.72 (7.96–11.8)	0.67 (0.543–0.813)	0.298 (0.24–0.361)	0.372 (0.302–0.448)	7570 (6470–8900)	593 (500–712)	265 (222–318)	328 (277–395)
Lebanon	Male	12.7 (10.3–15.4)	1.58 (1.28–1.91)	0.825 (0.668–0.999)	0.753 (0.61–0.915)	12200 (10500–14400)	1690 (1430–2000)	876 (743–1040)	812 (681–969)
Libya	Female	19.1 (14.5–24.3)	0.0646 (0.0471–0.0859)	0.0128 (0.00932–0.017)	0.0518 (0.0376–0.069)	16200 (13200–20800)	53.9 (39.5–74.3)	10.8 (7.93–14.9)	43.1 (31.5–59.4)
Libya	Male	26.5 (20.1–33.5)	2.66 (2.03–3.42)	1.21 (0.911–1.55)	1.45 (1.11–1.86)	22100 (17700–28000)	2130 (1630–2830)	962 (739–1250)	1170 (898–1570)
Morocco	Female	95.5 (75–117)	0.434 (0.302–0.6)	0.0584 (0.041–0.0824)	0.376 (0.261–0.518)	13900 (10700–17800)	69.4 (46.5–99.2)	9.58 (6.47–13.6)	59.9 (40.1–85.7)
Morocco	Male	110 (87.3–132)	5.6 (4.25–7.04)	1.93 (1.4–2.54)	3.66 (2.8–4.52)	16700 (13000–20500)	908 (666–1160)	298 (217–398)	610 (450–760)
Oman	Female	5.74 (4.64–7.1)	0.0392 (0.03–0.0516)	0.0141 (0.0108–0.0187)	0.0251 (0.0192–0.0329)	9250 (7330–11700)	68.4 (49.6–93.6)	23.7 (17.3–32.3)	44.6 (32.4–61.2)
Oman	Male	14.1 (11.4–17.3)	0.629 (0.498–0.796)	0.227 (0.179–0.288)	0.402 (0.315–0.507)	14900 (11700–18900)	635 (491–818)	277 (217–350)	358 (271–458)
Palestine	Female	8.55 (7.29–9.99)	0.0821 (0.0675–0.0998)	0.0194 (0.0161–0.0236)	0.0627 (0.0515–0.0761)	11500 (9860–13500)	150 (122–187)	42.6 (34.6–52.6)	108 (87–134)
Palestine	Male	12.5 (10.5–14.5)	1.04 (0.864–1.23)	0.466 (0.385–0.556)	0.574 (0.479–0.673)	16900 (14400–19200)	1730 (1440–2050)	815 (685–962)	917 (759–1090)
Qatar	Female	3.1 (2.58–3.71)	0.0132 (0.0106–0.0163)	0.00177 (0.00152–0.00209)	0.0114 (0.0091–0.0142)	8680 (7110–10700)	37.7 (30.2–47.4)	10.1 (8.18–12.6)	27.7 (21.7–34.9)
Qatar	Male	9.37 (7.49–11.6)	0.64 (0.504–0.797)	0.242 (0.189–0.307)	0.397 (0.314–0.494)	10100 (7890–12500)	632 (492–806)	247 (193–314)	385 (298–492)
Saudi Arabia	Female	83 (64.8–105)	0.902 (0.656–1.22)	0.325 (0.232–0.445)	0.577 (0.424–0.777)	13700 (10900–17600)	139 (102–191)	45.6 (33.2–63)	93.9 (68.9–128)
Saudi Arabia	Male	169 (132–209)	12.2 (9.52–15.6)	4.9 (3.72–6.41)	7.34 (5.76–9.17)	18400 (15000–22800)	1260 (995–1590)	477 (368–628)	780 (626–975)
Sudan	Female	135 (92.6–191)	0.906 (0.587–1.37)	0.097 (0.0663–0.142)	0.809 (0.519–1.23)	19600 (14000–26700)	161 (105–243)	22.2 (15.1–32.1)	139 (89–211)
Sudan	Male	165 (122–230)	7.05 (4.83–10.1)	2.83 (1.92–3.98)	4.22 (2.93–6.02)	23200 (17300–32200)	1310 (919–1880)	546 (378–779)	763 (527–1090)
Syrian Arab Republic	Female	45.2 (35.6–57)	0.54 (0.364–0.759)	0.0886 (0.0583–0.132)	0.451 (0.306–0.639)	18700 (14500–23700)	230 (155–335)	47.9 (32.5–67.1)	182 (122–268)
Syrian Arab Republic	Male	66.3 (52.2–83.8)	3.44 (2.53–4.56)	1.18 (0.827–1.62)	2.26 (1.67–2.97)	27800 (21800–34500)	1730 (1200–2410)	605 (416–855)	1120 (789–1550)
Tunisia	Female	23.7 (19–28.9)	0.198 (0.147–0.259)	0.0458 (0.0336–0.061)	0.152 (0.114–0.198)	8600 (6660–11000)	79.9 (56.1–109)	18.5 (12.9–25.7)	61.3 (42.9–83.6)
Tunisia	Male	32.7 (25.9–40.2)	3.11 (2.39–3.84)	1.38 (1.08–1.69)	1.72 (1.31–2.16)	13900 (10600–18100)	1510 (1110–2020)	693 (513–906)	821 (593–1110)
Turkey	Female	160 (139–184)	2.93 (2.39–3.55)	0.628 (0.494–0.782)	2.3 (1.89–2.76)	8370 (7130–9730)	177 (142–217)	40.4 (31.9–50.4)	136 (110–167)
Turkey	Male	224 (192–256)	20.2 (16.9–23.8)	6.37 (5.37–7.43)	13.8 (11.6–16.4)	13300 (11300–15600)	1340 (1090–1630)	439 (359–523)	901 (727–1100)
United Arab Emirates	Female	19.4 (16.1–23.2)	0.204 (0.163–0.25)	0.0246 (0.019–0.0304)	0.179 (0.143–0.219)	24600 (20800–29100)	283 (235–349)	30.4 (25.4–36.8)	253 (209–312)
United Arab Emirates	Male	38.3 (30.2–47.8)	2.63 (2.06–3.27)	1.15 (0.915–1.41)	1.48 (1.15–1.86)	9890 (8180–11700)	635 (510–767)	336 (270–407)	300 (240–364)
Yemen	Female	107 (79.5–147)	2.75 (1.8–4.21)	1.05 (0.667–1.72)	1.7 (1.14–2.51)	20100 (15200–27000)	659 (423–1020)	226 (141–364)	434 (282–659)
Yemen	Male	142 (105–189)	8.87 (6.29–12.2)	4.82 (3.36–6.85)	4.05 (2.97–5.42)	26700 (20600–35100)	2360 (1630–3260)	1220 (840–1700)	1140 (793–1550)
South Asia	Female	5110 (4350–5980)	54.1 (43.8–66.8)	22.6 (18.3–27.9)	31.5 (25.5–39.1)	17100 (14700–20000)	205 (155–271)	87.6 (65.6–116)	118 (89.6–155)
South Asia	Male	6510 (5490–7700)	330 (275–391)	149 (123–178)	181 (151–213)	21500 (18700–25400)	1230 (1000–1500)	550 (444–686)	677 (562–820)
South Asia	Female	5110 (4350–5980)	54.1 (43.8–66.8)	22.6 (18.3–27.9)	31.5 (25.5–39.1)	17100 (14700–20000)	205 (155–271)	87.6 (65.6–116)	118 (89.6–155)
South Asia	Male	6510 (5490–7700)	330 (275–391)	149 (123–178)	181 (151–213)	21500 (18700–25400)	1230 (1000–1500)	550 (444–686)	677 (562–820)
Bangladesh	Female	371 (294–464)	3.46 (2.56–4.61)	1.75 (1.26–2.41)	1.7 (1.27–2.21)	13500 (10900–16700)	128 (92.7–182)	60.2 (43.7–83.7)	68.2 (49.5–95.9)
Bangladesh	Male	443 (351–550)	38.5 (29.6–48.7)	21.3 (16.3–27.4)	17.2 (13.3–21.5)	16500 (13400–20600)	1520 (1150–2030)	818 (613–1110)	698 (533–921)
Bhutan	Female	1.7 (1.34–2.12)	0.021 (0.0154–0.0291)	0.00511 (0.00371–0.00709)	0.0159 (0.0117–0.0219)	14100 (11000–18200)	182 (130–267)	44.9 (31.8–65.6)	137 (98.6–200)
Bhutan	Male	2.22 (1.76–2.74)	0.0912 (0.0704–0.115)	0.0572 (0.0456–0.0702)	0.0339 (0.0252–0.0454)	17400 (13800–22400)	805 (616–1060)	553 (429–721)	252 (186–343)
India	Female	3870 (3240–4590)	42.7 (34.1–53.1)	18.5 (14.7–22.9)	24.2 (19.2–30.2)	16500 (14000–19700)	204 (153–270)	90.2 (66.7–119)	114 (86.4–150)
India	Male	5080 (4180–6160)	247 (199–300)	106 (84.3–129)	142 (115–171)	21400 (18200–25400)	1170 (954–1460)	502 (403–628)	669 (548–826)
Nepal	Female	76.5 (61.4–95)	1.87 (1.45–2.5)	0.761 (0.583–1.03)	1.11 (0.86–1.48)	14700 (12100–18400)	445 (328–621)	180 (130–255)	265 (197–367)
Nepal	Male	87.6 (70.8–108)	4.15 (3.29–5.12)	2 (1.58–2.5)	2.15 (1.71–2.63)	19500 (16000–24300)	1210 (939–1570)	585 (449–764)	624 (488–793)
Pakistan	Female	794 (633–984)	6.09 (4.48–8.37)	1.66 (1.17–2.33)	4.42 (3.3–6.01)	23000 (18700–28200)	217 (154–314)	52.3 (36–76.9)	165 (118–236)
Pakistan	Male	896 (721–1110)	40.2 (30.2–52.5)	19.8 (14.7–26.1)	20.3 (15.5–26.1)	24500 (19600–30500)	1410 (1000–1940)	675 (475–932)	733 (525–1000)

Supplementary Results Table S2. Cumulative all-age counts and age-standardised rates of years of life lost (YLLs) between 2022 to 2050 by location, sex, and scenario

		All-Age Count (Millions)				Age-Standardized Rate (per 100,000)			
Location	Sex	Reference	Difference between Reference and Elimination - 2023	Difference between Reference and Elimination - 2050	Difference between Elimination - 2050 and Elimination - 2023	Reference	Difference between Reference and Elimination - 2023	Difference between Reference and Elimination - 2050	Difference between Elimination - 2050 and Elimination - 2023
Southeast Asia, East Asia, and Oceania	Female	5160 (4590–5770)	136 (123–151)	71.5 (65.1–79.2)	64.8 (58.1–72.1)	9860 (8550–11300)	283 (238–332)	151 (126–177)	132 (111–155)
Southeast Asia, East Asia, and Oceania	Male	7540 (6740–8450)	799 (710–902)	353 (316–398)	446 (394–504)	16800 (14700–19400)	2000 (1710–2340)	926 (806–1070)	1070 (902–1270)
East Asia	Female	3240 (2770–3770)	111 (99.2–126)	63 (56.9–70.6)	48.2 (41.8–55.1)	7430 (5970–9060)	300 (243–365)	172 (139–209)	128 (103–155)
East Asia	Male	4830 (4160–5660)	597 (512–693)	273 (237–314)	324 (275–379)	14100 (11500–17100)	2070 (1670–2550)	1020 (847–1230)	1050 (825–1320)
China	Female	3110 (2640–3640)	109 (97–124)	62.3 (56.2–70)	46.7 (40.3–53.7)	7350 (5840–9000)	304 (244–373)	176 (142–216)	128 (102–157)
China	Male	4650 (3990–5470)	579 (494–674)	265 (229–307)	313 (264–368)	14000 (11300–17100)	2090 (1680–2600)	1040 (856–1260)	1050 (817–1330)
Democratic People's Republic of Korea	Female	81.2 (64.5–103)	1.93 (1.44–2.53)	0.647 (0.471–0.873)	1.28 (0.965–1.66)	13200 (10400–17400)	370 (275–498)	133 (98.4–180)	237 (177–317)
Democratic People's Republic of Korea	Male	107 (85.4–134)	12.3 (9.68–15.1)	4.95 (3.84–6.25)	7.35 (5.83–8.96)	21000 (17100–26300)	2670 (2150–3280)	1110 (888–1410)	1560 (1260–1890)
Taiwan (Province of China)	Female	51.2 (46.6–56.4)	0.221 (0.198–0.25)	0.00937 (0.00827–0.0107)	0.211 (0.189–0.239)	5990 (5610–6310)	37.2 (34.2–39.8)	2.61 (2.34–2.92)	34.6 (31.7–37.1)
Taiwan (Province of China)	Male	76.1 (69.1–83.9)	5.71 (5.18–6.32)	2.21 (2–2.44)	3.5 (3.16–3.89)	11800 (11100–12400)	1080 (1020–1140)	440 (414–463)	643 (602–677)
Oceania	Female	62.5 (50.2–77.9)	1.65 (1.24–2.15)	0.605 (0.446–0.813)	1.04 (0.79–1.34)	24800 (20300–30800)	840 (631–1130)	290 (215–398)	550 (415–732)
Oceania	Male	77.5 (63.4–95.1)	3.57 (2.87–4.56)	1.28 (1–1.7)	2.29 (1.85–2.88)	29600 (24800–36500)	1860 (1460–2380)	602 (462–781)	1260 (995–1590)
American Samoa	Female	0.174 (0.147–0.206)	0.00518 (0.00422–0.00636)	0.00187 (0.00146–0.00241)	0.00331 (0.00275–0.00396)	19200 (15500–23800)	655 (513–838)	230 (174–304)	425 (337–532)
American Samoa	Male	0.222 (0.191–0.261)	0.0151 (0.0129–0.0178)	0.00644 (0.00535–0.00787)	0.00866 (0.00753–0.00999)	24500 (20500–29300)	1990 (1660–2410)	808 (658–1010)	1180 (1010–1410)
Cook Islands	Female	0.0438 (0.037–0.0519)	0.000658 (0.000515–0.00084)	0.000121 (0.0000907–0.00016)	0.000537 (0.000425–0.00068)	9150 (7500–11200)	184 (140–239)	36.6 (26.9–49.2)	147 (113–190)
Cook Islands	Male	0.0664 (0.0573–0.0769)	0.00353 (0.00302–0.00415)	0.00117 (0.000974–0.00141)	0.00236 (0.00204–0.00273)	18000 (15100–21400)	1230 (1020–1490)	407 (330–503)	824 (690–989)
Fiji	Female	3.01 (2.42–3.8)	0.0615 (0.0467–0.0803)	0.0254 (0.0186–0.0341)	0.0361 (0.028–0.0464)	20800 (15900–27000)	470 (341–643)	188 (134–263)	282 (207–380)
Fiji	Male	3.93 (3.15–4.9)	0.257 (0.204–0.324)	0.115 (0.0887–0.15)	0.142 (0.114–0.173)	29600 (22800–37600)	2150 (1570–2790)	893 (647–1180)	1250 (923–1610)
Guam	Female	0.313 (0.27–0.358)	0.011 (0.00928–0.0131)	0.00306 (0.00244–0.00381)	0.00792 (0.00679–0.00932)	10100 (8900–11600)	401 (343–475)	130 (108–157)	272 (233–317)
Guam	Male	0.515 (0.444–0.581)	0.0304 (0.0261–0.0351)	0.0108 (0.00907–0.0127)	0.0196 (0.017–0.0222)	17400 (15500–19700)	1140 (994–1320)	427 (364–500)	715 (628–816)
Kiribati	Female	0.491 (0.383–0.617)	0.0288 (0.0219–0.0373)	0.0159 (0.0118–0.0213)	0.0128 (0.0101–0.0161)	27200 (21400–34400)	1810 (1340–2390)	902 (641–1220)	904 (694–1170)
Kiribati	Male	0.645 (0.505–0.804)	0.0706 (0.0553–0.0876)	0.0366 (0.028–0.0465)	0.034 (0.0272–0.041)	39000 (31400–48400)	5490 (4330–7150)	2740 (2140–3570)	2750 (2190–3530)
Marshall Islands	Female	0.251 (0.194–0.327)	0.00715 (0.00509–0.00986)	0.00297 (0.00204–0.00419)	0.00418 (0.00305–0.00567)	29100 (22100–38800)	919 (654–1260)	356 (247–499)	563 (405–762)
Marshall Islands	Male	0.321 (0.246–0.409)	0.0269 (0.0202–0.0344)	0.012 (0.00891–0.0159)	0.0148 (0.0113–0.0186)	35600 (27700–46100)	3460 (2540–4600)	1540 (1110–2060)	1930 (1420–2540)
Micronesia (Federated States of)	Female	0.356 (0.273–0.456)	0.0182 (0.0131–0.0241)	0.00859 (0.00589–0.0118)	0.00959 (0.00725–0.0123)	24000 (18600–30600)	1340 (975–1820)	593 (420–828)	745 (554–996)
Micronesia (Federated States of)	Male	0.502 (0.387–0.631)	0.0497 (0.0372–0.0625)	0.0232 (0.0167–0.0305)	0.0265 (0.0206–0.0323)	34300 (27400–43600)	4020 (3040–5270)	1730 (1270–2310)	2290 (1770–2980)
Nauru	Female	0.0499 (0.0373–0.0645)	0.00302 (0.00212–0.0042)	0.00134 (0.000897–0.00197)	0.00168 (0.00122–0.00222)	30100 (22800–40200)	1890 (1300–2700)	752 (494–1130)	1130 (814–1580)
Nauru	Male	0.075 (0.0575–0.0944)	0.00616 (0.00467–0.00789)	0.00314 (0.00234–0.00411)	0.00301 (0.00234–0.00379)	47700 (37000–61700)	5220 (3850–6940)	2520 (1840–3390)	2710 (2010–3590)
Niue	Female	0.00802 (0.00673–0.00949)	0.000144 (0.000108–0.000186)	0.0000401 (0.0000288–0.0000543)	0.000103 (0.0000795–0.000132)	24500 (21000–28800)	497 (364–642)	143 (103–191)	354 (261–452)
Niue	Male	0.00966 (0.00816–0.0113)	0.000512 (0.000415–0.000621)	0.000263 (0.000214–0.000321)	0.000249 (0.000203–0.000301)	31600 (27300–37000)	2060 (1670–2520)	1010 (809–1240)	1050 (853–1280)
Northern Mariana Islands	Female	0.138 (0.121–0.158)	0.00325 (0.00275–0.0038)	0.000876 (0.000715–0.00106)	0.00238 (0.00203–0.00274)	13700 (11600–15900)	360 (299–424)	99.9 (80.7–122)	260 (219–304)
Northern Mariana Islands	Male	0.218 (0.194–0.242)	0.0156 (0.0141–0.0173)	0.0055 (0.00488–0.00623)	0.0101 (0.00914–0.0111)	21700 (19300–24100)	1810 (1580–2020)	625 (542–711)	1180 (1040–1310)
Palau	Female	0.0752 (0.0629–0.091)	0.00177 (0.00146–0.00212)	0.000666 (0.000545–0.000816)	0.0011 (0.000922–0.00131)	24400 (20300–30000)	582 (467–722)	209 (167–260)	374 (301–461)
Palau	Male	0.106 (0.0872–0.129)	0.00912 (0.00751–0.011)	0.00466 (0.00387–0.00559)	0.00445 (0.00364–0.00544)	27500 (22800–33700)	2490 (2000–3110)	1230 (993–1530)	1260 (1010–1590)
Papua New Guinea	Female	50 (39.1–63.5)	1.28 (0.925–1.73)	0.448 (0.31–0.625)	0.836 (0.616–1.1)	25800 (20600–32600)	886 (644–1240)	287 (206–415)	599 (438–825)
Papua New Guinea	Male	60.8 (48.4–76.8)	2.4 (1.81–3.27)	0.726 (0.504–1.07)	1.68 (1.3–2.17)	29500 (24200–36900)	1640 (1230–2190)	427 (306–596)	1210 (916–1580)
Samoa	Female	0.703 (0.571–0.877)	0.0208 (0.0155–0.0271)	0.00613 (0.00436–0.00842)	0.0147 (0.0112–0.0188)	21800 (17800–27200)	836 (612–1110)	228 (160–313)	608 (449–792)
Samoa	Male	0.846 (0.688–1.07)	0.0632 (0.0508–0.0774)	0.0257 (0.0201–0.0324)	0.0375 (0.0309–0.0456)	24500 (20100–30400)	2460 (2000–3050)	967 (769–1220)	1500 (1230–1850)
Solomon Islands	Female	2.61 (1.97–3.42)	0.108 (0.0762–0.146)	0.0578 (0.0401–0.0793)	0.0502 (0.0361–0.0671)	26800 (21100–35300)	1310 (945–1810)	678 (484–947)	635 (459–867)
Solomon Islands	Male	3.61 (2.8–4.57)	0.324 (0.252–0.412)	0.196 (0.152–0.25)	0.128 (0.0993–0.161)	36200 (29100–46200)	4340 (3230–5700)	2530 (1870–3340)	1810 (1370–2370)
Tokelau	Female	0.00649 (0.00556–0.00768)	0.000138 (0.000105–0.000177)	0.0000486 (0.0000364–0.0000647)	0.0000889 (0.0000694–0.000113)	24900 (21000–29600)	619 (459–830)	214 (155–293)	405 (303–538)
Tokelau	Male	0.00685 (0.00591–0.008)	0.000371 (0.000296–0.000463)	0.000152 (0.000117–0.000196)	0.000219 (0.000178–0.000266)	26300 (22900–30800)	1710 (1330–2190)	661 (495–869)	1050 (831–1320)
Tonga	Female	0.241 (0.189–0.299)	0.00539 (0.00422–0.00674)	0.00153 (0.00118–0.00192)	0.00387 (0.00304–0.00482)	14100 (10900–18000)	407 (310–527)	113 (85.8–147)	294 (224–380)
Tonga	Male	0.318 (0.252–0.393)	0.0262 (0.0211–0.0313)	0.0133 (0.0107–0.0159)	0.0129 (0.0105–0.0154)	21100 (16800–26100)	2440 (1970–2960)	1220 (984–1480)	1220 (981–1480)
Tuvalu	Female	0.0429 (0.0346–0.0541)	0.00193 (0.00143–0.00256)	0.000898 (0.000643–0.00124)	0.00103 (0.000793–0.00133)	20900 (16600–26600)	1090 (803–1480)	480 (341–666)	613 (460–809)
Tuvalu	Male	0.0635 (0.0518–0.0783)	0.00605 (0.00481–0.0076)	0.003 (0.00236–0.00387)	0.00304 (0.00246–0.00374)	30300 (24500–38200)	3430 (2670–4470)	1590 (1220–2100)	1830 (1450–2350)
Vanuatu	Female	1.05 (0.871–1.28)	0.00929 (0.00719–0.0118)	0.00196 (0.00153–0.00249)	0.00733 (0.00558–0.00936)	21300 (17300–25900)	250 (189–331)	60.4 (45.7–81.5)	190 (143–252)
Vanuatu	Male	1.57 (1.3–1.86)	0.0946 (0.0744–0.115)	0.0388 (0.0299–0.0483)	0.0559 (0.0445–0.0671)	32500 (26900–38500)	2500 (1930–3110)	987 (761–1240)	1510 (1180–1860)
Southeast Asia	Female	1860 (1660–2080)	23.4 (20.8–26.2)	7.86 (7–8.91)	15.5 (13.9–17.3)	13500 (11800–15400)	192 (165–222)	64.1 (54.9–75)	127 (110–147)
Southeast Asia	Male	2620 (2380–2930)	199 (181–221)	79.1 (71.6–88.9)	120 (109–132)	21100 (18900–23600)	1810 (1590–2040)	708 (625–804)	1100 (968–1240)
Cambodia	Female	51.6 (39.8–64.6)	0.829 (0.653–1.02)	0.34 (0.264–0.425)	0.489 (0.388–0.601)	17500 (13700–21900)	359 (273–450)	141 (107–178)	218 (167–272)
Cambodia	Male	65.5 (50.5–81.9)	5.5 (4.44–6.69)	3.32 (2.68–4.02)	2.18 (1.75–2.65)	25700 (20500–31400)	3020 (2400–3620)	1870 (1490–2220)	1150 (910–1400)
Indonesia	Female	793 (659–952)	9.01 (7.54–10.6)	2.7 (2.28–3.17)	6.31 (5.26–7.44)	15400 (12500–18900)	214 (169–260)	72 (56.8–88)	142 (112–173)
Indonesia	Male	1020 (860–1200)	92.7 (77.6–110)	35.3 (29.5–42.7)	57.4 (47.9–67.7)	21000 (17600–25200)	2160 (1680–2700)	836 (657–1050)	1320 (1030–1650)
Lao People's Democratic Republic	Female	21.5 (17.3–26.8)	0.393 (0.311–0.483)	0.191 (0.15–0.237)	0.202 (0.161–0.246)	17900 (14400–22900)	397 (307–506)	187 (144–239)	210 (163–266)
Lao People's Democratic Republic	Male	28.5 (22.6–35.6)	2.58 (2.11–3.12)	1.49 (1.22–1.8)	1.09 (0.888–1.31)	24700 (19600–31000)	2880 (2290–3550)	1690 (1350–2080)	1190 (927–1480)
Malaysia	Female	72 (61.8–82.6)	0.495 (0.422–0.571)	0.116 (0.0978–0.142)	0.379 (0.32–0.442)	11700 (10800–12800)	118 (106–134)	40.4 (35.9–46.4)	77.3 (69.5–87.9)
Malaysia	Male	106 (91–122)	7.38 (6.32–8.43)	2.07 (1.76–2.38)	5.31 (4.54–6.07)	16800 (15700–18200)	1380 (1270–1500)	403 (369–443)	975 (900–1060)
Maldives	Female	0.567 (0.466–0.689)	0.0122 (0.00975–0.0153)	0.00433 (0.00344–0.00546)	0.00786 (0.00629–0.00977)	6630 (5470–8070)	146 (116–190)	49.5 (38.7–65)	96.3 (76.4–125)
Maldives	Male	1.06 (0.846–1.29)	0.128 (0.101–0.159)	0.0769 (0.0612–0.0954)	0.0512 (0.0395–0.0645)	8890 (7380–10800)	1020 (808–1280)	637 (511–804)	379 (299–477)
Mauritius	Female	3.64 (3.13–4.24)	0.0288 (0.0247–0.0338)	0.00565 (0.00486–0.00669)	0.0232 (0.0198–0.0271)	11400 (10500–12500)	104 (93.6–115)	20.8 (18.6–23.2)	83 (74.8–91.6)
Mauritius	Male	4.99 (4.34–5.8)	0.332 (0.287–0.386)	0.16 (0.137–0.185)	0.172 (0.149–0.2)	19200 (17700–20600)	1410 (1300–1520)	660 (608–716)	750 (693–802)
Myanmar	Female	176 (145–214)	3.67 (2.97–4.48)	1.83 (1.45–2.28)	1.85 (1.51–2.22)	17200 (14300–21400)	412 (322–540)	186 (142–243)	226 (175–296)
Myanmar	Male	245 (202–297)	12.1 (10.1–14.5)	5.13 (4.28–6.09)	6.96 (5.78–8.38)	28600 (23700–35000)	1930 (1580–2350)	893 (740–1090)	1040 (835–1280)
Philippines	Female	290 (243–342)	5.03 (4.17–5.93)	1.82 (1.53–2.13)	3.21 (2.63–3.81)	14600 (12400–17100)	324 (266–392)	127 (106–152)	197 (161–240)
Philippines	Male	436 (362–509)	26 (21.3–30.5)	9.25 (7.41–11.1)	16.7 (13.8–19.5)	22600 (18800–26700)	1590 (1250–1970)	522 (407–645)	1070 (844–1320)
Seychelles	Female	0.283 (0.242–0.325)	0.00277 (0.00235–0.00321)	0.000517 (0.000441–0.0006)	0.00225 (0.00191–0.00261)	12200 (11000–13800)	141 (124–161)	28.2 (24.8–32.2)	113 (99.2–129)
Seychelles	Male	0.425 (0.366–0.485)	0.0317 (0.0275–0.0365)	0.0166 (0.0145–0.0191)	0.0151 (0.0131–0.0175)	18300 (16600–20300)	1650 (1480–1840)	884 (805–984)	761 (681–857)
Sri Lanka	Female	46.8 (36.2–58.9)	0.279 (0.219–0.352)	0.0755 (0.0601–0.0938)	0.204 (0.16–0.258)	8240 (6020–10600)	58.7 (41.4–79.3)	16 (11.3–21.5)	42.7 (30–57.5)
Sri Lanka	Male	63.2 (46.9–80.6)	2.86 (2.18–3.61)	1.18 (0.922–1.46)	1.68 (1.24–2.14)	14900 (10700–19500)	806 (545–1090)	341 (235–457)	464 (309–635)
Thailand	Female	172 (137–210)	1.76 (1.41–2.12)	0.312 (0.246–0.38)	1.45 (1.16–1.74)	8700 (7120–10600)	88.9 (69.8–110)	16.1 (12.6–20.2)	72.8 (57.2–89.4)
Thailand	Male	242 (193–306)	16.2 (12.9–19.9)	6.43 (5.16–7.83)	9.77 (7.68–12.1)	17300 (13900–21300)	1140 (881–1460)	446 (350–565)	691 (532–895)

Supplementary Results Table S2. Cumulative all-age counts and age-standardised rates of years of life lost (YLLs) between 2022 to 2050 by location, sex, and scenario

Location	Sex	All-Age Count (Millions)				Age-Standardized Rate (per 100,000)			
		Reference	Difference between Reference and Elimination - 2023	Difference between Reference and Elimination - 2050	Difference between Elimination - 2050 and Elimination - 2023	Reference	Difference between Reference and Elimination - 2023	Difference between Reference and Elimination - 2050	Difference between Elimination - 2050 and Elimination - 2023
Timor-Leste	Female	3.61 (2.98–4.45)	0.026 (0.0203–0.0327)	0.00761 (0.00589–0.00986)	0.0184 (0.0145–0.0228)	16600 (13700–20700)	187 (144–245)	53.6 (41–71.7)	133 (103–174)
Timor-Leste	Male	4.79 (3.97–5.87)	0.313 (0.249–0.398)	0.132 (0.102–0.175)	0.181 (0.146–0.225)	22100 (18100–27300)	2140 (1650–2780)	848 (651–1120)	1290 (999–1670)
Viet Nam	Female	226 (189–266)	1.83 (1.53–2.17)	0.449 (0.368–0.549)	1.38 (1.16–1.63)	9940 (8240–12000)	94.5 (76.1–117)	22.4 (17.9–28.3)	72 (58–89.3)
Viet Nam	Male	403 (337–470)	32.5 (26.8–37.5)	14.4 (11.7–17.1)	18.1 (15.2–20.5)	22500 (19000–26200)	2140 (1740–2500)	909 (731–1080)	1230 (1000–1420)
Sub-Saharan Africa	Female	5200 (4360–6390)	13.1 (11.1–15.2)	5.5 (4.61–6.63)	7.59 (6.56–8.74)	24200 (20600–29000)	107 (88.9–129)	42.6 (35.4–52.1)	64.4 (54.1–76.8)
Sub-Saharan Africa	Male	6390 (5350–7830)	84.4 (74.1–95.8)	39.7 (34.2–46.3)	44.7 (39.7–49.7)	31300 (26800–37100)	708 (615–814)	299 (257–350)	410 (355–465)
Central Sub-Saharan Africa	Female	598 (437–830)	1.43 (1.06–1.89)	0.624 (0.452–0.846)	0.801 (0.606–1.05)	25300 (20200–33100)	90.8 (68.1–120)	33.8 (24.9–45.2)	57 (43–74.8)
Central Sub-Saharan Africa	Male	748 (558–1000)	14.5 (11.2–18.1)	7.61 (5.9–9.6)	6.89 (5.32–8.54)	33500 (27400–42600)	1060 (837–1310)	482 (376–607)	574 (462–706)
Angola	Female	150 (115–195)	0.539 (0.394–0.715)	0.238 (0.167–0.325)	0.301 (0.228–0.39)	24100 (19100–31400)	142 (104–192)	53.2 (37.9–74.1)	88.9 (66.2–118)
Angola	Male	177 (136–235)	4.2 (3.32–5.21)	2.51 (1.96–3.19)	1.68 (1.36–2.03)	31900 (25400–40500)	1440 (1130–1800)	779 (605–976)	664 (528–825)
Central African Republic	Female	36.4 (25.7–51.8)	0.0623 (0.0424–0.0882)	0.0259 (0.0168–0.0378)	0.0364 (0.025–0.0507)	43100 (31900–59100)	129 (86.5–181)	46.4 (30–67.6)	82.2 (57.3–114)
Central African Republic	Male	48.2 (34.7–65.3)	0.628 (0.441–0.827)	0.329 (0.225–0.442)	0.299 (0.215–0.386)	63000 (48600–81800)	1640 (1220–2090)	810 (593–1030)	834 (624–1060)
Congo	Female	23.3 (17.9–30.5)	0.0703 (0.0495–0.0945)	0.0291 (0.0195–0.0399)	0.0411 (0.0294–0.0543)	27700 (22000–35300)	94.9 (65.6–128)	36.3 (24.7–50.7)	58.7 (41–78.2)
Congo	Male	26.2 (20.6–34.3)	0.905 (0.707–1.13)	0.498 (0.391–0.618)	0.407 (0.315–0.509)	31500 (25400–39100)	1530 (1190–1880)	854 (680–1050)	671 (517–836)
Democratic Republic of the Congo	Female	376 (232–590)	0.721 (0.457–1.05)	0.323 (0.202–0.482)	0.397 (0.255–0.581)	24800 (19100–35000)	70.1 (51.2–96)	26.5 (19.3–36)	43.6 (32–60.3)
Democratic Republic of the Congo	Male	480 (321–700)	8.3 (5.48–11.2)	4.05 (2.62–5.6)	4.26 (2.84–5.69)	32800 (26200–42100)	862 (675–1110)	340 (259–451)	522 (412–658)
Equatorial Guinea	Female	6.47 (4.44–9.2)	0.0129 (0.00825–0.019)	0.00391 (0.00236–0.00602)	0.00903 (0.00586–0.0131)	24300 (16400–34000)	70.5 (43.9–107)	18.9 (11.3–29.3)	51.6 (32.7–77.6)
Equatorial Guinea	Male	8.46 (6.22–11.8)	0.224 (0.155–0.302)	0.118 (0.0794–0.165)	0.106 (0.0763–0.139)	32100 (24300–43100)	1250 (896–1690)	533 (373–735)	716 (525–948)
Gabon	Female	6.19 (4.61–8.24)	0.0206 (0.0145–0.0277)	0.00459 (0.00308–0.00633)	0.016 (0.0114–0.0213)	20300 (15400–26700)	80.1 (55.8–112)	16.3 (11.2–23.1)	63.8 (44.7–88)
Gabon	Male	8.74 (6.6–11.4)	0.242 (0.189–0.298)	0.106 (0.0815–0.134)	0.136 (0.108–0.166)	31800 (25200–40100)	1180 (945–1470)	481 (385–605)	699 (559–868)
Eastern Sub-Saharan Africa	Female	1650 (1400–1960)	5.63 (4.69–6.77)	3.09 (2.53–3.78)	2.54 (2.15–3.02)	21800 (18800–25900)	132 (109–163)	68.3 (55.8–85.6)	64 (53.7–78.4)
Eastern Sub-Saharan Africa	Male	2120 (1780–2530)	33.2 (28.6–39.2)	17.8 (15.1–21.7)	15.4 (13.6–17.6)	29200 (25400–34300)	754 (652–884)	360 (308–430)	394 (343–456)
Burundi	Female	53.1 (40.6–72)	0.15 (0.109–0.198)	0.0518 (0.0362–0.0716)	0.0978 (0.0728–0.128)	23600 (18800–30300)	101 (74.2–135)	25.4 (18.1–35.2)	75.5 (56.1–100)
Burundi	Male	70 (55–91.4)	1.11 (0.84–1.38)	0.658 (0.495–0.838)	0.448 (0.354–0.55)	31700 (25600–39600)	811 (632–1030)	410 (314–527)	402 (317–502)
Comoros	Female	2.55 (1.94–3.19)	0.00994 (0.00742–0.0128)	0.00286 (0.00209–0.00373)	0.00708 (0.00532–0.00906)	21800 (18000–27000)	104 (80–138)	29.2 (22.2–38.9)	75.1 (57.6–99.2)
Comoros	Male	2.89 (2.2–3.6)	0.0801 (0.0616–0.099)	0.0424 (0.0326–0.0524)	0.0377 (0.029–0.0466)	25400 (21000–30900)	916 (719–1170)	490 (388–625)	426 (331–552)
Djibouti	Female	3.98 (2.98–5.27)	0.0297 (0.022–0.0394)	0.012 (0.00877–0.016)	0.0177 (0.0132–0.0233)	19700 (15100–25900)	184 (131–259)	70.2 (49.4–99)	114 (81.7–160)
Djibouti	Male	5.81 (4.45–7.53)	0.304 (0.231–0.391)	0.157 (0.117–0.206)	0.146 (0.113–0.185)	26500 (20500–34400)	1730 (1320–2340)	860 (655–1160)	872 (659–1180)
Eritrea	Female	23.8 (15.4–34)	0.0138 (0.00907–0.0199)	0.00339 (0.00221–0.00506)	0.0104 (0.00686–0.0149)	24600 (18900–31700)	20.1 (14.6–26.3)	4.72 (3.43–6.24)	15.3 (11.2–20.1)
Eritrea	Male	33.9 (22–48.5)	0.66 (0.412–0.951)	0.292 (0.174–0.444)	0.368 (0.239–0.515)	35500 (28000–44600)	862 (648–1150)	325 (232–455)	537 (414–699)
Ethiopia	Female	353 (274–448)	0.264 (0.192–0.35)	0.107 (0.0755–0.144)	0.158 (0.117–0.206)	18100 (15000–21700)	18.3 (13.6–23.7)	6.12 (4.43–8.15)	12.1 (9.27–15.5)
Ethiopia	Male	470 (372–590)	4.41 (3.51–5.61)	2.29 (1.81–2.97)	2.11 (1.69–2.64)	23700 (20100–28300)	371 (300–466)	169 (136–214)	202 (165–248)
Kenya	Female	140 (118–166)	0.353 (0.275–0.483)	0.106 (0.0812–0.147)	0.247 (0.192–0.337)	18100 (15300–22000)	67.8 (49.3–104)	18.4 (13.4–27.4)	49.4 (35.7–76.5)
Kenya	Male	185 (156–219)	3.39 (2.69–4.12)	1.3 (0.996–1.67)	2.08 (1.7–2.49)	25300 (21200–29900)	615 (479–755)	196 (152–247)	418 (327–511)
Madagascar	Female	125 (103–155)	0.256 (0.19–0.331)	0.0532 (0.0384–0.0701)	0.203 (0.151–0.261)	26500 (21800–32400)	84.6 (61.5–112)	21.1 (15.2–27.9)	63.5 (46.4–84.7)
Madagascar	Male	142 (116–175)	2.26 (1.7–2.88)	0.734 (0.538–0.981)	1.52 (1.16–1.93)	30600 (24700–37900)	710 (514–930)	210 (153–276)	500 (362–656)
Malawi	Female	72.8 (55.3–96.5)	0.279 (0.207–0.365)	0.153 (0.112–0.203)	0.126 (0.0951–0.162)	23300 (18000–30200)	138 (97.7–186)	72.6 (50.9–99.6)	65.6 (47–87.1)
Malawi	Male	98.6 (79.3–124)	2.67 (2.18–3.29)	1.7 (1.39–2.11)	0.972 (0.806–1.17)	35300 (29200–44300)	1770 (1440–2200)	1120 (917–1390)	649 (525–808)
Mozambique	Female	139 (109–174)	0.437 (0.299–0.583)	0.124 (0.08–0.172)	0.313 (0.218–0.411)	24900 (19700–31500)	138 (93.7–190)	34 (22.2–48.3)	104 (72.3–142)
Mozambique	Male	181 (145–228)	3.23 (2.52–3.94)	1.81 (1.38–2.28)	1.42 (1.15–1.68)	38700 (32100–47700)	1370 (1100–1700)	685 (544–859)	687 (556–839)
Rwanda	Female	46 (35.4–64.2)	0.812 (0.632–1.01)	0.558 (0.432–0.701)	0.254 (0.199–0.31)	18800 (14900–25100)	547 (424–703)	355 (274–457)	192 (150–245)
Rwanda	Male	56 (42.6–91.5)	1.82 (1.43–2.23)	1.28 (1–1.57)	0.541 (0.427–0.657)	25300 (20000–37900)	1400 (1120–1750)	938 (748–1170)	460 (366–574)
Somalia	Female	156 (103–236)	0.28 (0.177–0.411)	0.121 (0.0745–0.18)	0.159 (0.102–0.232)	37900 (27900–52200)	189 (133–268)	71.9 (49.4–105)	117 (83.3–165)
Somalia	Male	213 (139–316)	2.78 (1.84–4.05)	1.58 (1.02–2.41)	1.2 (0.827–1.68)	52300 (39200–71300)	1780 (1280–2400)	895 (641–1220)	881 (647–1170)
South Sudan	Female	79.5 (53.8–124)	0.11 (0.0755–0.154)	0.0428 (0.0285–0.0623)	0.067 (0.0472–0.0922)	34800 (25000–51600)	121 (83–180)	40.1 (26.7–61.5)	80.4 (55.8–119)
South Sudan	Male	98.9 (68.5–143)	0.894 (0.625–1.24)	0.441 (0.296–0.652)	0.453 (0.329–0.588)	45400 (33000–64300)	1060 (740–1490)	443 (301–649)	618 (438–852)
Uganda	Female	158 (123–203)	0.482 (0.376–0.606)	0.229 (0.176–0.298)	0.253 (0.202–0.309)	19100 (15600–24200)	124 (94.9–164)	51.9 (39–69.6)	72.2 (56.1–94.4)
Uganda	Male	212 (169–271)	1.94 (1.53–2.47)	0.768 (0.587–1)	1.17 (0.944–1.45)	28600 (24200–34700)	495 (396–624)	155 (122–201)	340 (274–425)
United Republic of Tanzania	Female	216 (174–268)	1.8 (1.41–2.31)	1.36 (1.06–1.77)	0.437 (0.349–0.536)	20800 (17300–25500)	273 (216–353)	195 (153–252)	78.5 (62.4–102)
United Republic of Tanzania	Male	249 (201–306)	5.59 (4.35–7.09)	3.65 (2.79–4.71)	1.94 (1.55–2.37)	25200 (21200–30300)	837 (661–1080)	486 (378–631)	352 (283–443)
Zambia	Female	79 (59.2–104)	0.354 (0.277–0.453)	0.166 (0.13–0.211)	0.188 (0.146–0.242)	24000 (18100–31200)	263 (195–345)	125 (92.4–164)	138 (102–183)
Zambia	Male	101 (78.3–133)	2.04 (1.57–2.55)	1.07 (0.813–1.37)	0.966 (0.761–1.18)	33200 (25600–42100)	1110 (868–1390)	488 (373–623)	623 (492–769)
Southern Sub-Saharan Africa	Female	336 (295–382)	3.27 (2.91–3.66)	0.938 (0.83–1.05)	2.33 (2.05–2.63)	24500 (22100–27400)	280 (257–306)	78.3 (69.8–88.8)	202 (185–218)
Southern Sub-Saharan Africa	Male	445 (389–505)	15.1 (13.5–16.8)	6.78 (5.97–7.61)	8.35 (7.44–9.28)	36100 (32700–40200)	1480 (1340–1620)	606 (539–691)	869 (798–943)
Botswana	Female	8.42 (6.97–10.1)	0.105 (0.0829–0.129)	0.0439 (0.0343–0.0541)	0.0616 (0.0486–0.0747)	20900 (17600–24800)	311 (248–390)	121 (94.9–153)	190 (152–237)
Botswana	Male	10.6 (8.78–12.6)	0.542 (0.437–0.645)	0.289 (0.233–0.345)	0.253 (0.204–0.3)	29000 (24800–33900)	1830 (1510–2230)	1000 (837–1210)	826 (679–1010)
Eswatini	Female	5.25 (3.95–6.62)	0.0216 (0.0168–0.0263)	0.0127 (0.01–0.0152)	0.00892 (0.00681–0.0112)	30800 (23000–40200)	253 (178–340)	147 (104–198)	105 (74–144)
Eswatini	Male	7.12 (5.52–8.67)	0.106 (0.0841–0.128)	0.0433 (0.0339–0.0527)	0.0624 (0.0501–0.0749)	48800 (39200–61600)	1190 (951–1530)	467 (371–591)	727 (578–948)
Lesotho	Female	10.7 (8.85–12.9)	0.0668 (0.0555–0.0785)	0.0532 (0.0442–0.0622)	0.0136 (0.0109–0.0169)	38500 (31500–47600)	526 (411–654)	431 (340–532)	94.5 (70.5–123)
Lesotho	Male	15.1 (12.8–17.9)	0.854 (0.7–1.01)	0.512 (0.417–0.614)	0.342 (0.282–0.407)	62600 (53200–74400)	5590 (4630–6820)	3280 (2740–3960)	2300 (1870–2850)
Namibia	Female	8.17 (6.32–10.6)	0.108 (0.0802–0.138)	0.0563 (0.0418–0.0718)	0.052 (0.0385–0.0667)	20000 (14900–26500)	402 (275–540)	206 (141–275)	196 (134–266)
Namibia	Male	12 (9.33–15.3)	0.279 (0.221–0.337)	0.144 (0.114–0.173)	0.135 (0.106–0.164)	34100 (27100–43100)	1320 (1090–1610)	727 (604–875)	597 (483–741)
South Africa	Female	228 (197–263)	2.41 (2.08–2.77)	0.417 (0.354–0.487)	1.99 (1.73–2.28)	23200 (21500–25100)	257 (234–279)	42.1 (37.9–46.6)	214 (196–233)
South Africa	Male	309 (269–357)	9.46 (8.15–10.9)	3.21 (2.75–3.7)	6.24 (5.4–7.18)	34600 (31900–37500)	1170 (1080–1280)	357 (325–391)	816 (750–886)
Zimbabwe	Female	74.9 (58.2–92.9)	0.556 (0.452–0.668)	0.355 (0.288–0.427)	0.201 (0.163–0.241)	31700 (25100–39600)	488 (383–603)	309 (244–380)	180 (140–223)
Zimbabwe	Male	91.6 (71–115)	3.89 (3.14–4.72)	2.58 (2.05–3.14)	1.32 (1.07–1.58)	43800 (35300–53200)	3170 (2550–3880)	1950 (1560–2400)	1220 (997–1480)
Western Sub-Saharan Africa	Female	2620 (2080–3340)	2.76 (2.08–3.47)	0.849 (0.626–1.09)	1.92 (1.46–2.39)	25400 (20600–31900)	49.4 (37.3–63.8)	14.8 (11.1–19.2)	34.6 (26.1–44.3)
Western Sub-Saharan Africa	Male	3080 (2490–3910)	21.6 (17.3–26)	7.53 (5.99–9.24)	14 (11.2–16.8)	31200 (25500–38200)	424 (346–522)	135 (109–167)	289 (235–355)
Benin	Female	70.4 (50.1–107)	0.0816 (0.0543–0.108)	0.0203 (0.0133–0.0267)	0.0612 (0.0409–0.0811)	23700 (18400–35900)	54.3 (38.3–72.3)	13.3 (9.19–17.9)	41 (29.2–54.4)
Benin	Male	91.1 (66.1–151)	0.495 (0.346–0.633)	0.103 (0.0694–0.134)	0.392 (0.271–0.503)	32300 (25300–45600)	392 (292–498)	74.2 (53.6–95.7)	317 (239–403)
Burkina Faso	Female	156 (107–253)	0.0447 (0.0188–0.0639)	0.00897 (0.00294–0.0135)	0.0357 (0.015–0.0502)	28500 (20900–43300)	17.2 (9.17–24.7)	2.86 (0.952–4.23)	14.3 (7.88–20.5)
Burkina Faso	Male	187 (137–316)	0.919 (0.344–1.24)	0.289 (0.075–0.419)	0.63 (0.313–0.825)	37500 (29000–58700)	396 (196–530)	99.1 (27.8–140)	297 (175–393)

Supplementary Results Table S2. Cumulative all-age counts and age-standardised rates of years of life lost (YLLs) between 2022 to 2050 by location, sex, and scenario

Location	Sex	All-Age Count (Millions): Reference	All-Age Count (Millions): Difference between Reference and Elimination - 2023	All-Age Count (Millions): Difference between Reference and Elimination - 2050	All-Age Count (Millions): Difference between Elimination - 2050 and Elimination - 2023	Age-Standardized Rate (per 100,000): Reference	Age-Standardized Rate (per 100,000): Difference between Reference and Elimination - 2023	Age-Standardized Rate (per 100,000): Difference between Reference and Elimination - 2050	Age-Standardized Rate (per 100,000): Difference between Elimination - 2050 and Elimination - 2023
Cabo Verde	Female	1.08 (0.849–1.32)	0.00625 (0.00478–0.00795)	0.00194 (0.00146–0.00253)	0.00431 (0.00332–0.0054)	10300 (8260–12600)	60.7 (47.6–75.7)	17.8 (13.6–22.7)	42.9 (33.9–53.2)
Cabo Verde	Male	1.91 (1.49–2.35)	0.0506 (0.0395–0.0614)	0.0177 (0.0136–0.0223)	0.0329 (0.0261–0.0392)	21100 (17200–25200)	573 (457–686)	177 (140–216)	396 (318–469)
Cameroon	Female	136 (99.5–199)	0.117 (0.0789–0.16)	0.0247 (0.0183–0.0326)	0.0919 (0.0601–0.128)	24200 (18600–34800)	40.7 (29.3–55.6)	13.7 (10.8–17.2)	27.1 (18.7–38.1)
Cameroon	Male	173 (128–239)	1.92 (1.25–2.69)	0.747 (0.459–1.06)	1.17 (0.779–1.6)	31900 (24900–42500)	521 (340–719)	170 (104–241)	352 (238–479)
Chad	Female	153 (113–201)	0.138 (0.0987–0.186)	0.0172 (0.0123–0.0234)	0.121 (0.0863–0.162)	30900 (24400–39700)	94.1 (68.3–126)	12 (8.68–16)	82.1 (59.5–110)
Chad	Male	187 (134–248)	1.02 (0.733–1.31)	0.358 (0.248–0.475)	0.661 (0.477–0.837)	38100 (29200–48900)	657 (475–861)	188 (136–246)	469 (342–616)
Côte d'Ivoire	Female	112 (79.8–185)	0.279 (0.18–0.398)	0.0732 (0.045–0.105)	0.206 (0.134–0.293)	22500 (16800–35900)	87.5 (57–126)	21.8 (13.7–31.8)	65.8 (42.9–94)
Côte d'Ivoire	Male	153 (116–202)	2.3 (1.47–3.09)	0.643 (0.406–0.897)	1.66 (1.12–2.2)	30300 (23400–40600)	669 (447–925)	177 (119–245)	491 (327–679)
Gambia	Female	8.21 (6.46–10.2)	0.00894 (0.00646–0.0121)	0.00384 (0.00267–0.00524)	0.00511 (0.00378–0.00677)	21900 (17700–26700)	38.3 (27.9–50.5)	14.2 (10.1–19.2)	24.1 (17.7–31.3)
Gambia	Male	10.5 (8.41–13)	0.193 (0.148–0.251)	0.0694 (0.0508–0.0942)	0.123 (0.0966–0.157)	29500 (24300–35400)	797 (606–1010)	235 (178–305)	562 (431–705)
Ghana	Female	128 (99.4–186)	0.347 (0.249–0.461)	0.0878 (0.0624–0.118)	0.259 (0.181–0.346)	21800 (17100–30000)	82.4 (60–110)	22.2 (16.3–29.4)	60.2 (43.6–81.2)
Ghana	Male	165 (126–225)	2.42 (1.83–3.03)	1.4 (1.04–1.79)	1.02 (0.786–1.25)	31700 (25200–40300)	716 (570–897)	409 (321–512)	307 (242–386)
Guinea	Female	79.9 (56.9–134)	0.145 (0.106–0.191)	0.0464 (0.0334–0.0625)	0.0985 (0.0724–0.129)	29300 (21500–44900)	108 (78.7–145)	32.8 (23.7–44.7)	74.8 (55.1–101)
Guinea	Male	90.2 (65.8–159)	1.25 (0.927–1.6)	0.466 (0.334–0.612)	0.786 (0.586–0.987)	33900 (25300–51800)	988 (728–1290)	329 (241–435)	659 (486–860)
Guinea-Bissau	Female	10.4 (8.14–13.7)	0.0228 (0.0163–0.0302)	0.011 (0.00771–0.0146)	0.0118 (0.00848–0.0156)	29800 (23900–39400)	101 (74.3–135)	43.2 (31.1–59.2)	57.6 (43.1–76.4)
Guinea-Bissau	Male	13.7 (10.8–17.9)	0.199 (0.15–0.259)	0.118 (0.0868–0.156)	0.0813 (0.0601–0.102)	42900 (34800–57500)	1070 (830–1400)	561 (432–756)	505 (397–649)
Liberia	Female	23.7 (16.2–33.5)	0.0637 (0.0403–0.0883)	0.0257 (0.016–0.0364)	0.038 (0.0244–0.0522)	26800 (19200–38600)	95.1 (64.2–136)	34.5 (22.6–50.4)	60.6 (41.3–85.7)
Liberia	Male	27.5 (18.7–42)	0.364 (0.242–0.484)	0.18 (0.111–0.244)	0.185 (0.123–0.24)	29500 (21200–43200)	518 (362–707)	220 (142–310)	297 (211–404)
Mali	Female	161 (115–247)	0.273 (0.194–0.366)	0.142 (0.0981–0.197)	0.131 (0.093–0.174)	28300 (22500–37400)	123 (88.3–167)	55.2 (38.8–75.2)	67.8 (49.1–91.4)
Mali	Male	195 (144–260)	1.7 (1.31–2.12)	0.84 (0.649–1.06)	0.857 (0.667–1.06)	31800 (25600–39700)	847 (674–1080)	417 (332–533)	430 (339–549)
Mauritania	Female	13.6 (10.8–17.5)	0.0565 (0.0413–0.0787)	0.00987 (0.00697–0.014)	0.0466 (0.0343–0.0647)	18700 (15000–23800)	88.5 (63.8–123)	14 (9.87–20.1)	74.5 (53.9–104)
Mauritania	Male	15.4 (12.2–19.5)	0.232 (0.171–0.307)	0.062 (0.0432–0.087)	0.17 (0.128–0.222)	20900 (16600–26500)	436 (321–588)	94.5 (67.5–132)	341 (253–458)
Niger	Female	203 (141–347)	0.094 (0.0642–0.13)	0.0217 (0.0142–0.0307)	0.0723 (0.0505–0.0998)	30400 (22200–45800)	38.6 (26.4–54.3)	8.59 (5.82–12)	30 (20.8–42.4)
Niger	Male	227 (153–394)	0.784 (0.509–1.08)	0.207 (0.121–0.293)	0.577 (0.355–0.778)	33500 (24000–48800)	379 (273–500)	111 (82.9–141)	268 (188–360)
Nigeria	Female	1230 (880–1800)	0.755 (0.514–1.04)	0.244 (0.16–0.342)	0.511 (0.35–0.696)	25200 (19000–36100)	27.7 (18.5–39.4)	8.57 (5.66–12.4)	19.1 (12.8–27)
Nigeria	Male	1380 (1020–2120)	5.48 (3.71–7.36)	1.11 (0.727–1.53)	4.37 (3–5.82)	29800 (22700–40100)	224 (151–310)	36.1 (24–50.1)	188 (130–260)
Sao Tome and Principe	Female	0.468 (0.373–0.594)	0.00213 (0.00166–0.00275)	0.00118 (0.000908–0.00154)	0.000958 (0.00075–0.00124)	15600 (12600–19000)	79.7 (62.5–99.7)	40.9 (31.9–51.7)	38.8 (30.7–48.5)
Sao Tome and Principe	Male	0.591 (0.477–0.737)	0.0158 (0.0129–0.0192)	0.00807 (0.00647–0.0101)	0.00773 (0.00648–0.00922)	19900 (16800–23600)	605 (516–705)	276 (226–331)	329 (287–380)
Senegal	Female	48.7 (39.4–60.2)	0.0496 (0.0362–0.0652)	0.00533 (0.00384–0.00714)	0.0443 (0.0324–0.0581)	18100 (14900–22300)	23.9 (17.4–32.3)	2.45 (1.76–3.35)	21.4 (15.6–29)
Senegal	Male	60.3 (49.2–74.2)	0.788 (0.6–1)	0.253 (0.186–0.331)	0.535 (0.415–0.67)	23200 (19000–28600)	398 (300–512)	106 (78.9–138)	293 (222–373)
Sierra Leone	Female	48 (34.5–69.2)	0.149 (0.0843–0.215)	0.0285 (0.0163–0.0415)	0.12 (0.07–0.174)	29500 (22100–42700)	126 (76.5–186)	22.7 (13.6–34.2)	103 (62.9–151)
Sierra Leone	Male	55 (39.3–75.5)	0.736 (0.5–0.965)	0.319 (0.205–0.438)	0.417 (0.297–0.533)	33100 (24300–47100)	684 (476–929)	247 (163–343)	437 (307–585)
Togo	Female	32.7 (24.2–46.9)	0.132 (0.094–0.175)	0.0753 (0.0531–0.101)	0.0566 (0.0406–0.0726)	22800 (16800–32600)	124 (85.5–170)	64.9 (43.9–90.4)	59 (41.7–81)
Togo	Male	41.3 (30.4–59.8)	0.712 (0.479–0.925)	0.347 (0.221–0.46)	0.365 (0.255–0.465)	32100 (23800–45300)	849 (561–1150)	378 (242–517)	471 (318–632)

Supplementary Results Table S3. Life expectancy at birth by location, sex, and scenario

Location	Sex	2050			Change between 2022 and 2050		
		Reference	Difference between Elimination-2023 and Reference	Difference between Elimination-2050 and Reference	Reference	Elimination - 2023	Elimination - 2050
Global	Male	76.1 (73.6–78)	1.51 (1.45–1.58)	0.957 (0.912–1)	5.07 (2.79–6.67)	6.58 (4.27–8.23)	6.03 (3.73–7.66)
Global	Female	80.6 (78.1–82.6)	0.414 (0.395–0.435)	0.213 (0.197–0.23)	4.39 (1.82–6.16)	4.81 (2.22–6.58)	4.61 (2.02–6.38)
Central Europe, Eastern Europe, and Central Asia	Male	75.6 (73.5–77.2)	1.87 (1.81–1.95)	1.08 (1.04–1.12)	6.06 (4.04–7.53)	7.93 (5.92–9.42)	7.13 (5.14–8.61)
Central Europe, Eastern Europe, and Central Asia	Female	82.1 (79.9–84.1)	0.326 (0.309–0.343)	0.118 (0.11–0.127)	4.72 (2.61–6.4)	5.04 (2.94–6.73)	4.84 (2.73–6.52)
Central Asia	Male	75.9 (72–78.8)	1.94 (1.85–2.04)	1.28 (1.22–1.35)	6.95 (3.23–9.75)	8.88 (5.16–11.7)	8.23 (4.5–11.1)
Central Asia	Female	81.6 (77.7–84.8)	0.144 (0.126–0.166)	0.0389 (0.0298–0.0476)	6.25 (2.56–9.4)	6.4 (2.72–9.54)	6.29 (2.6–9.44)
Armenia	Male	79.8 (77.6–81.7)	3.38 (3.06–3.76)	2.34 (2.12–2.6)	7.05 (4.59–10.1)	10.4 (7.81–13.6)	9.39 (6.8–12.5)
Armenia	Female	84.8 (82.9–86.6)	0.104 (0.094–0.114)	0.0198 (0.0175–0.0223)	5.34 (3.14–8.14)	5.45 (3.24–8.24)	5.36 (3.16–8.16)
Azerbaijan	Male	76.7 (73.2–79.6)	2.75 (2.52–3.04)	2.06 (1.87–2.29)	6.95 (3.78–9.66)	9.7 (6.36–12.5)	9.01 (5.67–11.8)
Azerbaijan	Female	81.8 (78–85.2)	0.0492 (0.0415–0.0614)	0.00714 (0.00599–0.00901)	6.52 (3.01–9.64)	6.57 (3.06–9.68)	6.52 (3.02–9.64)
Georgia	Male	73.3 (71.7–74.8)	3.1 (2.87–3.4)	2.11 (1.96–2.29)	4 (2.43–5.24)	7.1 (5.4–8.53)	6.11 (4.47–7.45)
Georgia	Female	84.4 (82.4–86.3)	0.303 (0.285–0.319)	0.0632 (0.0583–0.0693)	5.81 (3.84–7.49)	6.11 (4.14–7.79)	5.87 (3.91–7.55)
Kazakhstan	Male	75.1 (72.4–77)	1.59 (1.47–1.71)	0.785 (0.725–0.847)	9.05 (6.43–11.6)	10.6 (8.05–13.1)	9.83 (7.24–12.3)
Kazakhstan	Female	81.7 (79.1–83.9)	0.124 (0.11–0.146)	0.0197 (0.0167–0.0243)	7.68 (5.05–10.3)	7.8 (5.19–10.5)	7.69 (5.08–10.4)
Kyrgyzstan	Male	74.9 (71.3–78.2)	3.12 (2.92–3.34)	2.27 (2.12–2.44)	7.48 (3.74–10.6)	10.6 (6.82–13.8)	9.75 (5.99–12.9)
Kyrgyzstan	Female	81.6 (78.3–84.9)	0.3 (0.258–0.349)	0.0668 (0.0555–0.0816)	6.72 (3.11–10)	7.02 (3.45–10.3)	6.78 (3.19–10.1)
Mongolia	Male	70.3 (66.3–73.8)	2.86 (2.63–3.11)	2.07 (1.91–2.25)	3.18 (-0.527–5.97)	6.04 (2.28–8.89)	5.25 (1.54–8.09)
Mongolia	Female	80.4 (76.9–83.3)	0.456 (0.421–0.494)	0.219 (0.199–0.241)	4.39 (1.08–6.93)	4.85 (1.55–7.37)	4.61 (1.31–7.14)
Tajikistan	Male	77.8 (74–81.4)	1.04 (0.926–1.16)	0.632 (0.56–0.709)	6.46 (2.44–10.1)	7.5 (3.56–11.1)	7.1 (3.09–10.8)
Tajikistan	Female	80.3 (75.3–84.4)	0.133 (0.11–0.166)	0.0461 (0.0366–0.0613)	5.49 (1.22–9.63)	5.62 (1.38–9.74)	5.53 (1.28–9.66)
Turkmenistan	Male	73.9 (67.8–78.9)	1.08 (0.988–1.21)	0.545 (0.506–0.606)	7.21 (2.26–11.3)	8.29 (3.4–12.4)	7.75 (2.82–11.9)
Turkmenistan	Female	80.8 (74.3–86.3)	0.143 (0.11–0.189)	0.03 (0.0206–0.0464)	7.29 (2.09–12.4)	7.43 (2.26–12.5)	7.32 (2.13–12.4)
Uzbekistan	Male	76.5 (71.3–80.5)	1.64 (1.51–1.83)	1.12 (1.04–1.25)	5.61 (1.3–9.18)	7.25 (3.01–10.8)	6.73 (2.48–10.3)
Uzbekistan	Female	81.3 (76.6–85.6)	0.111 (0.0937–0.141)	0.0317 (0.0262–0.0414)	5.48 (1.03–9.34)	5.59 (1.16–9.44)	5.51 (1.07–9.36)
Central Europe	Male	77.7 (76.7–78.5)	1.69 (1.59–1.82)	0.95 (0.893–1.02)	4.52 (3.83–5.09)	6.21 (5.41–6.86)	5.47 (4.73–6.08)
Central Europe	Female	83.7 (82.6–84.6)	0.701 (0.654–0.758)	0.295 (0.274–0.32)	3.81 (2.84–4.58)	4.51 (3.51–5.32)	4.1 (3.13–4.89)
Albania	Male	79.7 (77.6–81.4)	2.88 (2.56–3.29)	2.21 (1.96–2.53)	4.29 (2.39–5.79)	7.16 (5.15–8.78)	6.49 (4.48–8.06)
Albania	Female	83.1 (81.2–84.9)	0.666 (0.587–0.756)	0.283 (0.248–0.327)	3.39 (1.55–4.94)	4.06 (2.19–5.69)	3.68 (1.82–5.26)
Bosnia and Herzegovina	Male	80 (77.7–82.8)	2.93 (2.52–3.34)	1.91 (1.63–2.18)	5.28 (3.42–7.19)	8.21 (6.1–10.2)	7.19 (5.16–9.19)
Bosnia and Herzegovina	Female	84.4 (81.7–87)	1.15 (1.02–1.31)	0.477 (0.414–0.555)	4.63 (2.22–6.43)	5.79 (3.25–7.62)	5.11 (2.63–6.93)
Bulgaria	Male	74 (72.2–75.6)	1.52 (1.39–1.67)	0.78 (0.701–0.869)	5.08 (3.92–6.18)	6.59 (5.49–7.71)	5.86 (4.76–6.95)
Bulgaria	Female	80.4 (78.9–81.9)	0.619 (0.547–0.695)	0.275 (0.239–0.318)	4.41 (3.31–5.63)	5.03 (3.94–6.25)	4.69 (3.59–5.9)
Croatia	Male	79.3 (78.3–80.4)	1.92 (1.72–2.15)	1.16 (1.05–1.29)	4.5 (3.73–5.29)	6.43 (5.56–7.24)	5.67 (4.83–6.47)
Croatia	Female	84.1 (83–85.1)	0.711 (0.636–0.805)	0.294 (0.256–0.34)	3.35 (2.47–4.22)	4.06 (3.14–4.97)	3.65 (2.74–4.54)
Czechia	Male	80.4 (79.2–81.6)	1.5 (1.37–1.64)	0.817 (0.752–0.894)	3.66 (2.89–4.32)	5.16 (4.3–5.87)	4.48 (3.66–5.13)
Czechia	Female	85.4 (84.1–86.8)	0.596 (0.539–0.663)	0.204 (0.182–0.231)	2.92 (1.88–3.89)	3.51 (2.44–4.53)	3.12 (2.07–4.11)
Hungary	Male	78 (76.7–79.2)	1.54 (1.39–1.7)	0.76 (0.689–0.84)	4.82 (4–5.53)	6.36 (5.49–7.08)	5.58 (4.73–6.29)
Hungary	Female	82.9 (81.5–84.2)	0.716 (0.632–0.799)	0.255 (0.223–0.288)	3.21 (2.13–4.09)	3.92 (2.83–4.83)	3.46 (2.38–4.35)
Montenegro	Male	76.7 (75.4–78)	2.01 (1.76–2.28)	1.29 (1.13–1.46)	4.4 (3.6–5.34)	6.41 (5.52–7.34)	5.69 (4.84–6.61)
Montenegro	Female	81.1 (80–82.3)	0.807 (0.721–0.898)	0.406 (0.363–0.463)	3.34 (2.36–4.43)	4.15 (3.13–5.23)	3.75 (2.76–4.81)
North Macedonia	Male	77.3 (75.7–78.9)	1.9 (1.67–2.18)	1.15 (1.01–1.31)	6.34 (4.71–8.71)	8.24 (6.55–10.6)	7.49 (5.8–9.83)
North Macedonia	Female	80 (78.5–81.4)	0.729 (0.646–0.82)	0.301 (0.264–0.343)	5.19 (3.66–7.08)	5.92 (4.41–7.76)	5.49 (3.97–7.36)
Poland	Male	77.5 (76.4–78.7)	1.63 (1.49–1.82)	0.883 (0.805–0.978)	4.07 (3.44–4.85)	5.7 (5.02–6.48)	4.95 (4.32–5.73)
Poland	Female	84.8 (83.9–85.9)	0.744 (0.663–0.837)	0.309 (0.272–0.353)	3.68 (2.9–4.47)	4.43 (3.6–5.21)	3.99 (3.19–4.78)
Romania	Male	76.2 (74.6–77.8)	1.64 (1.51–1.79)	0.877 (0.807–0.953)	4.49 (3.21–5.45)	6.13 (4.76–7.1)	5.37 (4.05–6.31)
Romania	Female	83 (81.2–84.6)	0.541 (0.491–0.59)	0.221 (0.196–0.246)	4.02 (2.55–5.29)	4.56 (3.1–5.82)	4.24 (2.79–5.51)
Serbia	Male	77.3 (75.8–78.7)	1.8 (1.6–2.03)	1.01 (0.889–1.13)	4.27 (3.42–5.2)	6.07 (5.13–7.01)	5.28 (4.4–6.23)
Serbia	Female	81.5 (80.2–82.7)	1.09 (0.977–1.22)	0.549 (0.489–0.625)	3.68 (2.55–4.78)	4.76 (3.65–5.95)	4.23 (3.13–5.38)
Slovakia	Male	78.2 (76.8–79.6)	1.66 (1.48–1.84)	1.06 (0.946–1.17)	4.49 (3.4–5.4)	6.15 (4.97–7.15)	5.55 (4.38–6.51)
Slovakia	Female	83.8 (82.3–85.2)	0.321 (0.282–0.364)	0.127 (0.11–0.147)	3.56 (2.24–4.73)	3.88 (2.55–5.06)	3.68 (2.37–4.87)

Supplementary Results Table S3. Life expectancy at birth by location, sex, and scenario

Location	Sex	2050			Change between 2022 and 2050		
		Reference	Difference between Elimination-2023 and Reference	Difference between Elimination-2050 and Reference	Reference	Elimination - 2023	Elimination - 2050
Slovenia	Male	82.2 (81.3–83.1)	0.99 (0.88–1.1)	0.395 (0.348–0.446)	3.66 (3.24–4.07)	4.65 (4.15–5.07)	4.05 (3.6–4.46)
Slovenia	Female	87.5 (86.4–88.5)	0.461 (0.394–0.532)	0.17 (0.14–0.203)	2.7 (2.1–3.23)	3.16 (2.53–3.69)	2.87 (2.26–3.4)
Eastern Europe	Male	74.7 (72.4–76.7)	1.95 (1.86–2.05)	1.06 (1.01–1.12)	6.48 (4.65–7.97)	8.43 (6.62–9.98)	7.54 (5.76–9.05)
Eastern Europe	Female	81.9 (79.6–84)	0.226 (0.202–0.262)	0.0736 (0.062–0.09)	4.74 (2.81–6.5)	4.97 (3.07–6.7)	4.82 (2.9–6.56)
Belarus	Male	73.6 (70.5–76.3)	3.01 (2.8–3.26)	2.16 (2–2.33)	6.57 (4.14–8.52)	9.58 (7.08–11.6)	8.72 (6.24–10.7)
Belarus	Female	82.9 (80–85.4)	0.175 (0.148–0.222)	0.0425 (0.0337–0.059)	6.24 (3.65–8.66)	6.42 (3.88–8.82)	6.29 (3.72–8.7)
Estonia	Male	79.4 (78.2–80.7)	1.09 (1–1.21)	0.497 (0.457–0.547)	5.78 (5.03–6.44)	6.88 (6.09–7.57)	6.28 (5.52–6.95)
Estonia	Female	86 (84.9–87.1)	0.285 (0.255–0.322)	0.103 (0.0904–0.117)	4.26 (3.33–5.15)	4.54 (3.62–5.45)	4.36 (3.43–5.25)
Latvia	Male	75.9 (74.5–77.4)	1.69 (1.57–1.82)	0.885 (0.819–0.949)	6.3 (5.05–7.74)	7.98 (6.74–9.45)	7.18 (5.95–8.65)
Latvia	Female	83.8 (82.4–85.1)	0.323 (0.294–0.354)	0.113 (0.102–0.127)	5.28 (3.81–6.92)	5.6 (4.13–7.24)	5.39 (3.93–7.03)
Lithuania	Male	75 (73.6–76.4)	1.82 (1.69–1.96)	1.08 (0.998–1.16)	4.74 (3.6–5.81)	6.56 (5.37–7.7)	5.82 (4.66–6.93)
Lithuania	Female	83.6 (82.2–85.1)	0.279 (0.249–0.309)	0.111 (0.0964–0.124)	3.96 (2.69–5.23)	4.24 (2.97–5.52)	4.07 (2.81–5.34)
Republic of Moldova	Male	76.6 (73–79.3)	2.67 (2.53–2.82)	1.6 (1.51–1.69)	6.12 (2.82–8.14)	8.8 (5.49–10.9)	7.72 (4.46–9.77)
Republic of Moldova	Female	84.9 (80.9–87.9)	0.218 (0.198–0.251)	0.0242 (0.0198–0.033)	6.08 (2.38–8.7)	6.3 (2.61–8.9)	6.1 (2.41–8.72)
Russian Federation	Male	75.1 (73.1–76.9)	1.98 (1.87–2.12)	1.04 (0.977–1.12)	6.94 (5.43–8.3)	8.92 (7.42–10.3)	7.98 (6.51–9.35)
Russian Federation	Female	82 (80–83.9)	0.242 (0.212–0.277)	0.0826 (0.0681–0.102)	4.97 (3.27–6.5)	5.21 (3.53–6.73)	5.05 (3.37–6.58)
Ukraine	Male	73.3 (68.2–78.3)	1.64 (1.46–1.8)	0.863 (0.785–0.936)	4.77 (2.04–7.2)	6.41 (3.67–8.93)	5.63 (2.91–8.11)
Ukraine	Female	81 (76.6–85.6)	0.168 (0.128–0.223)	0.0421 (0.0313–0.0597)	3.56 (0.723–6.28)	3.73 (0.916–6.42)	3.6 (0.775–6.32)
High-income	Male	81.5 (81.1–81.7)	1.05 (1.01–1.1)	0.524 (0.505–0.547)	2.64 (2.31–2.92)	3.69 (3.33–3.98)	3.17 (2.81–3.45)
High-income	Female	85.4 (85–85.8)	0.594 (0.573–0.614)	0.232 (0.224–0.239)	1.57 (1.17–1.91)	2.16 (1.75–2.51)	1.8 (1.4–2.14)
Australasia	Male	83.4 (83–83.7)	0.504 (0.481–0.529)	0.194 (0.184–0.204)	2.35 (1.98–2.62)	2.85 (2.47–3.13)	2.54 (2.17–2.82)
Australasia	Female	86.6 (86.2–87)	0.41 (0.39–0.434)	0.105 (0.1–0.111)	1.43 (0.974–1.75)	1.84 (1.38–2.17)	1.54 (1.08–1.86)
Australia	Male	83.5 (83.1–83.8)	0.434 (0.413–0.458)	0.133 (0.126–0.14)	2.3 (1.92–2.6)	2.74 (2.35–3.03)	2.43 (2.06–2.73)
Australia	Female	86.9 (86.4–87.3)	0.374 (0.353–0.398)	0.0856 (0.0806–0.0911)	1.42 (0.951–1.74)	1.79 (1.31–2.12)	1.5 (1.04–1.82)
New Zealand	Male	83 (82.6–83.4)	0.858 (0.813–0.906)	0.5 (0.474–0.527)	2.59 (2.21–2.91)	3.45 (3.05–3.78)	3.09 (2.7–3.42)
New Zealand	Female	85.4 (84.8–85.8)	0.593 (0.558–0.629)	0.205 (0.193–0.219)	1.5 (1–1.88)	2.1 (1.6–2.49)	1.71 (1.21–2.09)
High-income Asia Pacific	Male	84.5 (84.1–84.8)	1.05 (0.977–1.12)	0.422 (0.39–0.457)	2.63 (2.28–2.99)	3.68 (3.28–4.06)	3.05 (2.68–3.42)
High-income Asia Pacific	Female	89.3 (88.9–89.6)	0.299 (0.276–0.323)	0.0996 (0.09–0.112)	1.53 (1.18–1.91)	1.83 (1.45–2.22)	1.63 (1.27–2.01)
Brunei Darussalam	Male	78.1 (76.5–79.3)	1.26 (1.18–1.35)	0.849 (0.796–0.908)	3.13 (1.94–4)	4.39 (3.2–5.3)	3.97 (2.78–4.89)
Brunei Darussalam	Female	80.6 (79.1–82)	0.637 (0.581–0.697)	0.317 (0.289–0.35)	2.31 (1.07–3.12)	2.94 (1.72–3.79)	2.62 (1.39–3.46)
Japan	Male	84.3 (84–84.6)	0.938 (0.875–0.995)	0.291 (0.274–0.308)	2.13 (1.79–2.42)	3.06 (2.7–3.38)	2.42 (2.07–2.71)
Japan	Female	89.4 (89.1–89.7)	0.253 (0.238–0.271)	0.0316 (0.03–0.0333)	1.28 (0.965–1.59)	1.54 (1.21–1.84)	1.31 (0.996–1.62)
Republic of Korea	Male	84.5 (84.1–85)	1.3 (1.19–1.45)	0.701 (0.632–0.786)	4.17 (3.68–5.01)	5.48 (4.91–6.35)	4.87 (4.33–5.72)
Republic of Korea	Female	89 (88.5–89.5)	0.424 (0.373–0.487)	0.265 (0.229–0.308)	3.03 (2.5–3.91)	3.46 (2.89–4.35)	3.3 (2.74–4.19)
Singapore	Male	87 (86.4–87.6)	0.713 (0.677–0.758)	0.239 (0.228–0.253)	2.93 (2.3–3.47)	3.64 (3–4.2)	3.17 (2.54–3.72)
Singapore	Female	89.8 (89.1–90.4)	0.187 (0.166–0.21)	0.0374 (0.0306–0.0457)	1.82 (1.18–2.38)	2.01 (1.35–2.59)	1.86 (1.21–2.43)
High-income North America	Male	79.1 (78.5–79.5)	1.04 (0.996–1.09)	0.589 (0.562–0.617)	2.91 (2.4–3.36)	3.95 (3.43–4.44)	3.5 (2.98–3.97)
High-income North America	Female	83 (82.5–83.5)	0.848 (0.813–0.88)	0.341 (0.329–0.353)	1.77 (1.19–2.25)	2.62 (2.02–3.1)	2.11 (1.53–2.58)
Canada	Male	82.8 (82.3–83.2)	0.885 (0.835–0.94)	0.458 (0.432–0.485)	2.27 (1.84–2.65)	3.16 (2.7–3.54)	2.73 (2.28–3.12)
Canada	Female	85.9 (85.4–86.3)	0.675 (0.632–0.725)	0.22 (0.206–0.237)	1.31 (0.808–1.71)	1.98 (1.47–2.39)	1.53 (1.03–1.93)
Greenland	Male	76 (74.3–77.4)	1.94 (1.74–2.15)	1.08 (0.971–1.19)	4.45 (3.57–5.08)	6.39 (5.44–7.1)	5.53 (4.62–6.2)
Greenland	Female	81.8 (80.1–82.9)	1.82 (1.64–2.01)	0.993 (0.901–1.11)	4.64 (3.51–5.37)	6.45 (5.24–7.26)	5.63 (4.47–6.39)
United States of America	Male	78.6 (78–79.1)	1.06 (1.01–1.11)	0.604 (0.575–0.634)	2.91 (2.36–3.41)	3.98 (3.42–4.49)	3.52 (2.97–4.03)
United States of America	Female	82.7 (82.1–83.2)	0.868 (0.832–0.902)	0.356 (0.342–0.369)	1.77 (1.17–2.27)	2.64 (2.02–3.14)	2.13 (1.52–2.62)
Southern Latin America	Male	79.1 (78.5–79.7)	1.01 (0.963–1.06)	0.524 (0.497–0.552)	3.51 (2.96–3.99)	4.52 (3.95–5.04)	4.03 (3.47–4.53)
Southern Latin America	Female	83.3 (82.7–83.9)	0.578 (0.549–0.611)	0.281 (0.264–0.299)	2.28 (1.69–2.84)	2.86 (2.28–3.41)	2.56 (1.98–3.12)
Argentina	Male	77.9 (77.3–78.5)	1.18 (1.12–1.24)	0.643 (0.611–0.674)	3.09 (2.49–3.66)	4.27 (3.65–4.85)	3.73 (3.12–4.3)
Argentina	Female	82.3 (81.6–83)	0.716 (0.684–0.755)	0.361 (0.341–0.383)	2.01 (1.36–2.65)	2.72 (2.08–3.38)	2.37 (1.73–3.02)
Chile	Male	82.1 (81.6–82.7)	0.503 (0.48–0.528)	0.151 (0.142–0.161)	4.16 (3.6–4.66)	4.66 (4.11–5.18)	4.31 (3.76–4.81)
Chile	Female	85.6 (85.1–86.1)	0.267 (0.253–0.281)	0.0997 (0.0933–0.107)	2.68 (2.16–3.13)	2.95 (2.42–3.39)	2.78 (2.26–3.22)

Supplementary Results Table S3. Life expectancy at birth by location, sex, and scenario

		2050			Change between 2022 and 2050		
Location	Sex	Reference	Difference between Elimination-2023 and Reference	Difference between Elimination-2050 and Reference	Reference	Elimination - 2023	Elimination - 2050
Uruguay	Male	76.7 (76.1–77.2)	1.44 (1.36–1.53)	0.844 (0.8–0.892)	3.27 (2.8–3.66)	4.71 (4.21–5.11)	4.12 (3.62–4.51)
Uruguay	Female	82.7 (82.1–83.2)	0.449 (0.426–0.472)	0.168 (0.159–0.179)	2.36 (1.84–2.79)	2.81 (2.28–3.25)	2.53 (2.01–2.96)
Western Europe	Male	82.7 (82.5–82.9)	1.08 (1.03–1.13)	0.522 (0.5–0.546)	2.64 (2.42–2.8)	3.72 (3.48–3.89)	3.16 (2.94–3.33)
Western Europe	Female	86.2 (85.9–86.5)	0.497 (0.476–0.52)	0.177 (0.17–0.185)	1.63 (1.35–1.86)	2.13 (1.84–2.37)	1.81 (1.52–2.05)
Andorra	Male	83.9 (81.1–87)	1.37 (1.2–1.54)	0.727 (0.655–0.811)	2.27 (1.8–2.66)	3.64 (3.13–4.04)	3 (2.52–3.37)
Andorra	Female	87.8 (85.6–90.1)	0.364 (0.31–0.423)	0.0947 (0.0743–0.118)	1.58 (0.736–2.28)	1.95 (1.06–2.64)	1.68 (0.823–2.37)
Austria	Male	83.4 (83–83.8)	1.04 (0.982–1.11)	0.437 (0.412–0.467)	3.14 (2.7–3.49)	4.18 (3.74–4.55)	3.58 (3.14–3.93)
Austria	Female	86.9 (86.5–87.4)	0.428 (0.4–0.462)	0.136 (0.126–0.148)	2.2 (1.76–2.61)	2.63 (2.19–3.04)	2.34 (1.9–2.75)
Belgium	Male	82.5 (82.1–82.9)	1.17 (1.08–1.25)	0.593 (0.551–0.637)	2.65 (2.3–2.96)	3.81 (3.45–4.13)	3.24 (2.88–3.56)
Belgium	Female	86 (85.5–86.4)	0.503 (0.469–0.542)	0.142 (0.133–0.155)	1.56 (1.1–1.96)	2.06 (1.6–2.46)	1.7 (1.24–2.1)
Cyprus	Male	82.6 (81.6–83.6)	1.22 (1.11–1.35)	0.647 (0.589–0.717)	3.04 (2.46–3.5)	4.26 (3.63–4.75)	3.69 (3.09–4.15)
Cyprus	Female	85.7 (84.9–86.3)	0.248 (0.222–0.275)	0.0586 (0.0521–0.0665)	2.22 (1.64–2.67)	2.47 (1.89–2.93)	2.28 (1.7–2.74)
Denmark	Male	82.3 (82–82.6)	1.23 (1.15–1.32)	0.804 (0.751–0.867)	3.02 (2.71–3.38)	4.25 (3.92–4.63)	3.83 (3.5–4.19)
Denmark	Female	84.8 (84.4–85.2)	1.02 (0.956–1.08)	0.49 (0.46–0.521)	1.62 (1.21–2.01)	2.63 (2.22–3.02)	2.11 (1.7–2.5)
Finland	Male	82.6 (82.2–83.1)	0.841 (0.794–0.899)	0.321 (0.304–0.342)	4.56 (3.91–5.26)	5.4 (4.74–6.12)	4.88 (4.23–5.59)
Finland	Female	86.6 (86.2–87.1)	0.325 (0.303–0.349)	0.0894 (0.0831–0.0964)	2.98 (2.37–3.63)	3.31 (2.7–3.97)	3.07 (2.46–3.73)
France	Male	82.6 (82.3–82.8)	1.19 (1.09–1.29)	0.545 (0.498–0.592)	2.31 (2.03–2.59)	3.49 (3.22–3.77)	2.85 (2.58–3.13)
France	Female	87.3 (87–87.6)	0.465 (0.423–0.514)	0.183 (0.164–0.204)	1.43 (1.13–1.7)	1.9 (1.58–2.18)	1.62 (1.31–1.89)
Germany	Male	81.7 (81.5–82)	1.08 (1.02–1.14)	0.477 (0.453–0.505)	2.5 (2.2–2.78)	3.58 (3.26–3.87)	2.98 (2.67–3.26)
Germany	Female	85.2 (84.9–85.5)	0.52 (0.49–0.553)	0.165 (0.155–0.177)	1.38 (1.07–1.65)	1.9 (1.58–2.17)	1.54 (1.23–1.82)
Greece	Male	81.3 (80.8–81.7)	2.03 (1.92–2.17)	1.15 (1.09–1.22)	3.6 (3.18–4.01)	5.64 (5.18–6.1)	4.76 (4.33–5.19)
Greece	Female	85.1 (84.8–85.4)	0.667 (0.631–0.711)	0.265 (0.251–0.284)	2.19 (1.84–2.55)	2.86 (2.5–3.24)	2.46 (2.1–2.83)
Iceland	Male	84.3 (83.6–85.1)	0.845 (0.787–0.914)	0.45 (0.42–0.485)	2.47 (2.01–2.95)	3.32 (2.84–3.79)	2.92 (2.46–3.4)
Iceland	Female	87.1 (86.4–87.8)	0.534 (0.494–0.584)	0.1 (0.0916–0.111)	2.4 (1.89–2.83)	2.94 (2.38–3.4)	2.5 (1.98–2.94)
Ireland	Male	85 (84.6–85.4)	0.86 (0.817–0.917)	0.417 (0.394–0.447)	3.44 (3.13–3.89)	4.3 (4–4.74)	3.86 (3.55–4.3)
Ireland	Female	87.1 (86.7–87.5)	0.645 (0.601–0.687)	0.205 (0.19–0.219)	2.14 (1.82–2.48)	2.79 (2.46–3.13)	2.35 (2.02–2.7)
Israel	Male	84.5 (83.5–85.2)	0.802 (0.734–0.88)	0.321 (0.295–0.351)	2.78 (1.73–3.73)	3.58 (2.52–4.58)	3.1 (2.04–4.07)
Israel	Female	87.3 (86.6–87.7)	0.287 (0.262–0.316)	0.0644 (0.0583–0.0716)	1.92 (1.28–2.43)	2.21 (1.54–2.73)	1.98 (1.34–2.49)
Italy	Male	83.5 (83.3–83.8)	1.01 (0.956–1.09)	0.5 (0.471–0.535)	2.76 (2.43–3.08)	3.77 (3.46–4.12)	3.26 (2.94–3.59)
Italy	Female	87 (86.6–87.2)	0.409 (0.383–0.438)	0.108 (0.1–0.117)	1.87 (1.5–2.2)	2.28 (1.91–2.62)	1.98 (1.61–2.31)
Luxembourg	Male	83.1 (82.2–83.8)	1.14 (1.06–1.22)	0.584 (0.545–0.626)	2.05 (1.64–2.4)	3.19 (2.76–3.51)	2.64 (2.21–2.95)
Luxembourg	Female	86.5 (85.7–87.2)	0.507 (0.471–0.546)	0.126 (0.115–0.137)	1.22 (0.622–1.62)	1.73 (1.09–2.13)	1.35 (0.74–1.75)
Malta	Male	84.5 (83.7–85.4)	0.98 (0.907–1.06)	0.391 (0.362–0.425)	3.81 (3.09–4.46)	4.79 (4.04–5.45)	4.2 (3.47–4.85)
Malta	Female	86.8 (85.9–87.6)	0.377 (0.349–0.415)	0.0944 (0.0867–0.103)	2.87 (2.21–3.45)	3.25 (2.57–3.84)	2.97 (2.3–3.55)
Monaco	Male	80 (78.6–81.1)	1.6 (1.41–1.82)	0.96 (0.843–1.09)	2.3 (1.96–2.65)	3.9 (3.53–4.29)	3.26 (2.92–3.64)
Monaco	Female	83.5 (81.9–85.2)	0.829 (0.707–0.957)	0.293 (0.243–0.347)	1.22 (0.823–1.56)	2.04 (1.66–2.38)	1.51 (1.13–1.86)
Netherlands	Male	82.7 (82.4–82.9)	0.99 (0.932–1.05)	0.571 (0.539–0.607)	1.94 (1.73–2.13)	2.93 (2.71–3.12)	2.51 (2.29–2.7)
Netherlands	Female	84.7 (84.4–85)	0.68 (0.636–0.729)	0.289 (0.27–0.31)	0.872 (0.593–1.1)	1.55 (1.27–1.78)	1.16 (0.876–1.39)
Norway	Male	83.8 (83.5–84)	0.346 (0.327–0.368)	0.0837 (0.0786–0.0894)	2.38 (2.08–3.22)	2.72 (2.43–3.58)	2.46 (2.16–3.31)
Norway	Female	85.9 (85.6–86.2)	0.183 (0.173–0.194)	0.027 (0.025–0.0291)	1.33 (1.01–2.06)	1.51 (1.19–2.24)	1.36 (1.04–2.08)
Portugal	Male	82.6 (82.3–82.9)	0.976 (0.92–1.04)	0.378 (0.355–0.401)	3.44 (3.09–3.83)	4.42 (4.04–4.81)	3.82 (3.46–4.22)
Portugal	Female	87.5 (87–87.9)	0.212 (0.199–0.228)	0.0848 (0.0795–0.0907)	2.69 (2.21–3.08)	2.91 (2.42–3.31)	2.78 (2.3–3.17)
San Marino	Male	87.9 (85–90.7)	1.09 (0.887–1.3)	0.608 (0.51–0.714)	3.14 (2.55–3.79)	4.23 (3.56–4.9)	3.75 (3.12–4.39)
San Marino	Female	90.1 (87.1–93.2)	0.309 (0.243–0.381)	0.0653 (0.0474–0.085)	1.89 (1.15–2.57)	2.2 (1.47–2.87)	1.96 (1.22–2.63)
Spain	Male	83.5 (83.2–83.7)	1.15 (1.09–1.22)	0.492 (0.465–0.521)	2.98 (2.68–3.25)	4.13 (3.79–4.41)	3.48 (3.16–3.75)
Spain	Female	87.7 (87.3–88.1)	0.215 (0.198–0.236)	0.0698 (0.0642–0.0767)	1.77 (1.37–2.19)	1.99 (1.58–2.41)	1.84 (1.44–2.26)
Sweden	Male	84.5 (83.4–85.6)	0.777 (0.72–0.854)	0.475 (0.439–0.518)	2.24 (1.93–2.53)	3.01 (2.71–3.32)	2.71 (2.4–3.01)
Sweden	Female	86.5 (85.6–87.4)	0.673 (0.618–0.724)	0.299 (0.274–0.325)	1.37 (0.997–1.71)	2.04 (1.64–2.4)	1.66 (1.28–2.02)
Switzerland	Male	85.3 (84.9–85.6)	0.961 (0.9–1.04)	0.51 (0.476–0.553)	2.13 (1.84–2.43)	3.09 (2.78–3.39)	2.64 (2.34–2.94)
Switzerland	Female	87.8 (87.5–88.1)	0.551 (0.515–0.589)	0.174 (0.162–0.189)	1.12 (0.822–1.37)	1.67 (1.35–1.93)	1.3 (0.983–1.55)

Supplementary Results Table S3. Life expectancy at birth by location, sex, and scenario

Location	Sex	2050			Change between 2022 and 2050		
		Reference	Difference between Elimination-2023 and Reference	Difference between Elimination-2050 and Reference	Reference	Elimination - 2023	Elimination - 2050
United Kingdom	Male	82.2 (81.9–82.5)	1.08 (1.03–1.13)	0.604 (0.577–0.632)	2.3 (1.98–2.56)	3.38 (3.06–3.64)	2.9 (2.59–3.16)
United Kingdom	Female	84.9 (84.6–85.3)	0.771 (0.731–0.812)	0.304 (0.289–0.319)	1.47 (1.12–1.78)	2.24 (1.89–2.56)	1.78 (1.42–2.09)
Latin America and Caribbean	Male	77.2 (74.8–78.8)	0.757 (0.724–0.792)	0.441 (0.421–0.46)	4.64 (2.41–6.14)	5.39 (3.14–6.91)	5.08 (2.84–6.59)
Latin America and Caribbean	Female	82.4 (79.8–84.5)	0.312 (0.296–0.331)	0.116 (0.108–0.129)	3.7 (1.22–5.68)	4.01 (1.54–5.99)	3.81 (1.35–5.79)
Andean Latin America	Male	79.6 (77.1–81.9)	0.505 (0.468–0.545)	0.283 (0.262–0.306)	5.63 (3.91–7.13)	6.14 (4.4–7.66)	5.92 (4.19–7.42)
Andean Latin America	Female	82.6 (80.2–84.7)	0.136 (0.123–0.149)	0.0451 (0.0399–0.0507)	4.56 (2.75–6)	4.69 (2.88–6.14)	4.6 (2.79–6.05)
Bolivia (Plurinational State of)	Male	76.1 (72.7–79.1)	0.788 (0.697–0.898)	0.514 (0.454–0.587)	9.02 (6.16–12)	9.8 (6.92–12.8)	9.53 (6.66–12.5)
Bolivia (Plurinational State of)	Female	77.4 (74.1–80.3)	0.182 (0.159–0.207)	0.101 (0.0851–0.118)	6.93 (4.43–9.34)	7.11 (4.59–9.53)	7.03 (4.52–9.44)
Ecuador	Male	78.6 (75.9–81.2)	0.512 (0.46–0.571)	0.262 (0.234–0.294)	3.82 (1.96–5.24)	4.33 (2.45–5.75)	4.08 (2.22–5.49)
Ecuador	Female	82.6 (80.3–84.8)	0.165 (0.147–0.183)	0.0495 (0.0434–0.0561)	2.99 (1.34–4.19)	3.15 (1.5–4.36)	3.04 (1.39–4.24)
Peru	Male	81.3 (78.4–84.1)	0.397 (0.356–0.445)	0.203 (0.183–0.225)	5.08 (3.45–6.4)	5.48 (3.82–6.82)	5.29 (3.65–6.61)
Peru	Female	84.4 (81.8–86.9)	0.1 (0.0895–0.113)	0.0194 (0.0173–0.0217)	4.19 (2.45–5.59)	4.29 (2.55–5.69)	4.21 (2.47–5.61)
Caribbean	Male	74.5 (59.9–77.5)	1.03 (0.667–1.11)	0.62 (0.405–0.666)	4.72 (-11.4–18.7)	5.76 (-10.8–19.8)	5.34 (-11–19.3)
Caribbean	Female	78.8 (68.2–82.1)	0.457 (0.341–0.493)	0.208 (0.155–0.228)	4.13 (-7.52–14.7)	4.58 (-7.18–15.1)	4.34 (-7.37–14.9)
Antigua and Barbuda	Male	77.8 (76.3–78.9)	0.681 (0.626–0.739)	0.416 (0.383–0.451)	3.26 (1.63–4.29)	3.94 (2.31–5.02)	3.68 (2.05–4.73)
Antigua and Barbuda	Female	80.2 (78.6–81.4)	0.283 (0.267–0.301)	0.111 (0.104–0.119)	2.22 (0.541–3.34)	2.5 (0.807–3.63)	2.33 (0.647–3.45)
Bahamas	Male	74.3 (71.2–77.1)	0.789 (0.734–0.842)	0.518 (0.481–0.553)	3.82 (2.46–5.29)	4.61 (3.24–6.11)	4.34 (2.98–5.83)
Bahamas	Female	80.1 (77.4–82.6)	0.222 (0.208–0.237)	0.102 (0.0955–0.109)	3.04 (1.43–4.28)	3.26 (1.65–4.51)	3.14 (1.53–4.39)
Barbados	Male	78.5 (75.7–81.2)	0.575 (0.523–0.626)	0.291 (0.263–0.318)	4.61 (2.79–5.96)	5.19 (3.31–6.55)	4.9 (3.05–6.26)
Barbados	Female	80 (77.3–82.2)	0.0857 (0.0749–0.0986)	0.0154 (0.0136–0.0182)	2.73 (0.966–3.96)	2.82 (1.06–4.05)	2.75 (0.984–3.98)
Belize	Male	76.2 (73.4–78.6)	1.09 (1.03–1.16)	0.702 (0.663–0.744)	4.3 (1.3–7.43)	5.39 (2.35–8.54)	5.01 (1.98–8.15)
Belize	Female	81.5 (78.5–84.1)	0.274 (0.26–0.29)	0.118 (0.11–0.129)	4.51 (1.26–7.3)	4.78 (1.53–7.57)	4.63 (1.38–7.42)
Bermuda	Male	81.4 (79.6–82.7)	0.979 (0.888–1.09)	0.587 (0.533–0.649)	3.84 (3.1–4.52)	4.82 (4.06–5.54)	4.42 (3.69–5.13)
Bermuda	Female	88.4 (86.4–89.8)	0.388 (0.341–0.441)	0.156 (0.137–0.178)	3.35 (2.46–4.09)	3.74 (2.82–4.49)	3.51 (2.61–4.25)
Cuba	Male	78.5 (76.7–80.2)	1.57 (1.45–1.7)	0.884 (0.813–0.955)	2.35 (1.29–3.05)	3.92 (2.79–4.64)	3.23 (2.15–3.93)
Cuba	Female	83.7 (81.9–85.1)	0.625 (0.573–0.677)	0.205 (0.185–0.227)	2.54 (1.27–3.45)	3.16 (1.87–4.09)	2.74 (1.47–3.66)
Dominica	Male	73.1 (70.3–75.4)	0.77 (0.698–0.852)	0.458 (0.416–0.505)	4.55 (2.55–6.18)	5.32 (3.25–7.01)	5 (2.97–6.68)
Dominica	Female	77.7 (74.8–79.9)	0.218 (0.199–0.241)	0.0526 (0.0476–0.0578)	3.84 (1.65–5.58)	4.06 (1.85–5.82)	3.89 (1.7–5.63)
Dominican Republic	Male	76.9 (73.3–79.8)	1.21 (1.11–1.33)	0.909 (0.837–0.996)	4.37 (1.9–5.99)	5.58 (3.07–7.24)	5.28 (2.77–6.93)
Dominican Republic	Female	82.5 (79.1–85.2)	0.804 (0.725–0.893)	0.53 (0.474–0.596)	3.83 (1.38–5.62)	4.63 (2.13–6.52)	4.36 (1.86–6.22)
Grenada	Male	73.9 (71.5–75.7)	0.658 (0.617–0.704)	0.361 (0.333–0.394)	3.58 (1.74–4.77)	4.23 (2.4–5.43)	3.94 (2.11–5.13)
Grenada	Female	78.5 (76–80.5)	0.227 (0.215–0.241)	0.0799 (0.0747–0.0868)	3.35 (1.25–4.76)	3.58 (1.48–4.99)	3.43 (1.33–4.84)
Guyana	Male	70.9 (66.8–75)	0.825 (0.748–0.901)	0.458 (0.406–0.517)	6.86 (4.35–10.2)	7.69 (5.24–11)	7.32 (4.86–10.6)
Guyana	Female	77.8 (74.3–81)	0.225 (0.203–0.247)	0.107 (0.0947–0.12)	6.9 (4.43–10)	7.13 (4.66–10.2)	7.01 (4.54–10.1)
Haiti	Male	69.5 (40.8–74.9)	0.49 (0.134–0.577)	0.272 (0.077–0.329)	7.71 (-21.4–36)	8.2 (-21.3–36.5)	7.98 (-21.3–36.2)
Haiti	Female	71 (50.1–76.8)	0.2 (0.0892–0.24)	0.105 (0.0473–0.126)	7.69 (-13.6–29.2)	7.89 (-13.5–29.5)	7.8 (-13.6–29.4)
Jamaica	Male	78 (74.8–81.4)	0.94 (0.861–1.03)	0.469 (0.429–0.511)	4.14 (2.58–5.73)	5.08 (3.49–6.68)	4.61 (3.05–6.21)
Jamaica	Female	81.4 (78–84.7)	0.323 (0.298–0.35)	0.0777 (0.0702–0.0862)	3.85 (1.45–5.57)	4.17 (1.76–5.89)	3.93 (1.53–5.65)
Puerto Rico	Male	81.4 (78.9–83.7)	0.885 (0.81–0.959)	0.484 (0.444–0.521)	4.61 (3.33–5.73)	5.49 (4.17–6.65)	5.09 (3.79–6.23)
Puerto Rico	Female	87.4 (85.4–89.3)	0.39 (0.362–0.419)	0.0884 (0.0812–0.0968)	3.03 (1.55–4.33)	3.42 (1.92–4.74)	3.11 (1.64–4.42)
Saint Kitts and Nevis	Male	74.8 (72.7–76.7)	0.464 (0.429–0.51)	0.232 (0.216–0.253)	4.96 (3.32–6.3)	5.42 (3.79–6.78)	5.19 (3.55–6.54)
Saint Kitts and Nevis	Female	80.5 (78.1–82.7)	0.172 (0.158–0.187)	0.0711 (0.0649–0.0781)	4.02 (2.04–5.59)	4.19 (2.2–5.77)	4.09 (2.11–5.66)
Saint Lucia	Male	76.4 (73.5–78.9)	0.708 (0.647–0.771)	0.351 (0.32–0.383)	4.73 (2.47–6.78)	5.44 (3.2–7.45)	5.08 (2.83–7.11)
Saint Lucia	Female	82.5 (79.6–85)	0.214 (0.196–0.233)	0.0718 (0.0657–0.0785)	4.52 (2.12–6.44)	4.73 (2.33–6.67)	4.59 (2.19–6.52)
Saint Vincent and the Grenadines	Male	74 (72–75.7)	0.687 (0.633–0.741)	0.383 (0.345–0.423)	3.88 (2.18–5.14)	4.56 (2.9–5.81)	4.26 (2.59–5.5)
Saint Vincent and the Grenadines	Female	79.3 (76.9–81.3)	0.192 (0.181–0.206)	0.0842 (0.0795–0.09)	3.93 (1.69–5.4)	4.12 (1.89–5.59)	4.01 (1.78–5.48)
Suriname	Male	75.5 (70.9–79.8)	1.1 (1.03–1.18)	0.525 (0.479–0.58)	5.44 (2.33–7.96)	6.54 (3.39–9.11)	5.96 (2.85–8.5)
Suriname	Female	80.9 (76.8–84.2)	0.373 (0.347–0.405)	0.102 (0.091–0.117)	4.82 (1.79–7.32)	5.2 (2.15–7.68)	4.92 (1.89–7.41)
Trinidad and Tobago	Male	76 (72.5–79.5)	0.951 (0.856–1.04)	0.519 (0.453–0.586)	6.62 (4.67–8.82)	7.57 (5.67–9.75)	7.14 (5.24–9.32)
Trinidad and Tobago	Female	82.8 (79.7–85.9)	0.277 (0.253–0.304)	0.0721 (0.0632–0.0816)	6.48 (4.37–8.76)	6.76 (4.66–9.05)	6.55 (4.45–8.83)

Supplementary Results Table S3. Life expectancy at birth by location, sex, and scenario

		2050			Change between 2022 and 2050		
**Location**	**Sex**	**Reference**	**Difference between Elimination-2023 and Reference**	**Difference between Elimination-2050 and Reference**	**Reference**	**Elimination - 2023**	**Elimination - 2050**
United States Virgin Islands	Male	77.2<br>(74.6–79.6)	0.615<br>(0.553–0.698)	0.395<br>(0.355–0.446)	6.3<br>(4.45–9.14)	6.92<br>(5.05–9.78)	6.7<br>(4.84–9.55)
United States Virgin Islands	Female	86<br>(84–87.8)	0.278<br>(0.243–0.317)	0.0819<br>(0.0701–0.0959)	4.59<br>(2.62–7.29)	4.87<br>(2.88–7.58)	4.67<br>(2.7–7.38)
Central Latin America	Male	76.8<br>(73.7–79.1)	0.613<br>(0.585–0.642)	0.3<br>(0.283–0.321)	4.7<br>(2.03–6.24)	5.32<br>(2.63–6.86)	5<br>(2.34–6.54)
Central Latin America	Female	82.1<br>(78.9–84.8)	0.214<br>(0.198–0.238)	0.0459<br>(0.0388–0.0608)	3.55<br>(0.609–5.71)	3.76<br>(0.844–5.9)	3.59<br>(0.667–5.75)
Colombia	Male	82.1<br>(79.5–84.2)	0.497<br>(0.457–0.543)	0.145<br>(0.134–0.158)	5.33<br>(3.78–6.4)	5.83<br>(4.24–6.92)	5.48<br>(3.92–6.55)
Colombia	Female	86.8<br>(84.2–89)	0.193<br>(0.177–0.211)	0.0354<br>(0.0309–0.0406)	3.85<br>(1.71–5.33)	4.05<br>(1.9–5.53)	3.89<br>(1.74–5.37)
Costa Rica	Male	80.4<br>(78.5–82)	0.738<br>(0.681–0.795)	0.418<br>(0.386–0.451)	3.8<br>(2.36–4.78)	4.54<br>(3.05–5.52)	4.22<br>(2.75–5.19)
Costa Rica	Female	86.2<br>(84.5–87.7)	0.251<br>(0.238–0.265)	0.0403<br>(0.0382–0.0427)	3.26<br>(1.65–4.42)	3.51<br>(1.9–4.67)	3.3<br>(1.69–4.46)
El Salvador	Male	75.3<br>(72.1–78.3)	0.635<br>(0.574–0.701)	0.338<br>(0.298–0.385)	5.15<br>(3.07–6.72)	5.79<br>(3.72–7.33)	5.49<br>(3.43–7.03)
El Salvador	Female	82.5<br>(79.4–85.6)	0.241<br>(0.216–0.265)	0.104<br>(0.09–0.118)	3.52<br>(0.991–5.66)	3.76<br>(1.22–5.92)	3.62<br>(1.09–5.77)
Guatemala	Male	76.7<br>(74–79)	0.55<br>(0.512–0.595)	0.309<br>(0.286–0.336)	5.77<br>(3.66–7.28)	6.32<br>(4.22–7.86)	6.08<br>(3.98–7.6)
Guatemala	Female	81.4<br>(78.7–83.8)	0.182<br>(0.17–0.195)	0.0691<br>(0.0637–0.0751)	5.45<br>(2.88–7.55)	5.63<br>(3.06–7.74)	5.52<br>(2.95–7.62)
Honduras	Male	74.9<br>(70.5–77.1)	0.975<br>(0.826–1.12)	0.636<br>(0.541–0.729)	5.03<br>(0.328–9.96)	6<br>(1.15–11)	5.66<br>(0.862–10.6)
Honduras	Female	77.2<br>(73.5–79.9)	0.327<br>(0.278–0.383)	0.119<br>(0.0982–0.144)	4.39<br>(0.809–7.54)	4.72<br>(1.11–7.86)	4.51<br>(0.916–7.66)
Mexico	Male	75.8<br>(73.2–78.3)	0.582<br>(0.54–0.626)	0.277<br>(0.258–0.296)	4.56<br>(2.63–5.93)	5.14<br>(3.21–6.56)	4.83<br>(2.92–6.23)
Mexico	Female	81.2<br>(78.8–83.5)	0.186<br>(0.173–0.199)	0.0244<br>(0.0218–0.0266)	3.6<br>(1.61–5.27)	3.79<br>(1.78–5.47)	3.63<br>(1.63–5.3)
Nicaragua	Male	79.7<br>(75.9–82.6)	0.716<br>(0.598–0.801)	0.464<br>(0.387–0.527)	2.62<br>(-0.74–5.16)	3.34<br>(-0.0674–5.95)	3.09<br>(-0.333–5.68)
Nicaragua	Female	84<br>(81.1–86.5)	0.116<br>(0.1–0.136)	0.0305<br>(0.0258–0.0369)	2<br>(-0.609–4.26)	2.11<br>(-0.479–4.37)	2.03<br>(-0.573–4.29)
Panama	Male	81.5<br>(78.9–84.1)	0.537<br>(0.487–0.59)	0.208<br>(0.191–0.228)	4.61<br>(3.12–5.77)	5.15<br>(3.61–6.33)	4.82<br>(3.31–5.99)
Panama	Female	85.8<br>(83.4–88.3)	0.272<br>(0.254–0.291)	0.0985<br>(0.0921–0.106)	3.49<br>(1.75–4.94)	3.76<br>(2.01–5.22)	3.58<br>(1.85–5.04)
Venezuela (Bolivarian Republic of)	Male	70.3<br>(58.4–77.5)	0.874<br>(0.78–1.03)	0.497<br>(0.429–0.634)	2.52<br>(-7.89–8.01)	3.4<br>(-6.87–8.86)	3.02<br>(-7.27–8.47)
Venezuela (Bolivarian Republic of)	Female	78.1<br>(65.5–85.9)	0.366<br>(0.27–0.562)	0.119<br>(0.0719–0.225)	1.46<br>(-9.22–8.31)	1.83<br>(-8.7–8.63)	1.58<br>(-9.02–8.4)
Tropical Latin America	Male	77.5<br>(75.6–78.6)	0.915<br>(0.866–0.961)	0.59<br>(0.559–0.62)	4.18<br>(2.27–5.09)	5.1<br>(3.16–6.01)	4.77<br>(2.85–5.69)
Tropical Latin America	Female	83.3<br>(81.2–84.8)	0.437<br>(0.417–0.459)	0.194<br>(0.184–0.21)	3.49<br>(1.41–4.9)	3.92<br>(1.86–5.34)	3.68<br>(1.62–5.09)
Brazil	Male	77.5<br>(75.6–78.6)	0.906<br>(0.858–0.951)	0.583<br>(0.552–0.613)	4.2<br>(2.31–5.1)	5.1<br>(3.19–6.02)	4.78<br>(2.88–5.7)
Brazil	Female	83.3<br>(81.2–84.8)	0.433<br>(0.413–0.456)	0.191<br>(0.18–0.208)	3.48<br>(1.42–4.91)	3.91<br>(1.86–5.34)	3.67<br>(1.62–5.09)
Paraguay	Male	76<br>(72.3–79.1)	1.21<br>(1.09–1.34)	0.807<br>(0.727–0.893)	3.62<br>(1.31–5.02)	4.83<br>(2.4–6.27)	4.42<br>(2.03–5.85)
Paraguay	Female	82.3<br>(78.9–85.3)	0.637<br>(0.577–0.697)	0.341<br>(0.306–0.374)	3.82<br>(1.37–5.48)	4.45<br>(1.96–6.11)	4.16<br>(1.68–5.82)
North Africa and Middle East	Male	77.2<br>(74.3–79.5)	1.71<br>(1.62–1.8)	1.13<br>(1.06–1.2)	5.03<br>(2.31–7.1)	6.74<br>(4.03–8.89)	6.16<br>(3.44–8.28)
North Africa and Middle East	Female	79.7<br>(76.8–82)	0.206<br>(0.186–0.234)	0.0756<br>(0.0679–0.0879)	4.19<br>(1.4–6.34)	4.4<br>(1.63–6.54)	4.27<br>(1.49–6.41)
North Africa and Middle East	Male	77.2<br>(74.3–79.5)	1.71<br>(1.62–1.8)	1.13<br>(1.06–1.2)	5.03<br>(2.31–7.1)	6.74<br>(4.03–8.89)	6.16<br>(3.44–8.28)
North Africa and Middle East	Female	79.7<br>(76.8–82)	0.206<br>(0.186–0.234)	0.0756<br>(0.0679–0.0879)	4.19<br>(1.4–6.34)	4.4<br>(1.63–6.54)	4.27<br>(1.49–6.41)
Afghanistan	Male	72.5<br>(68.2–76.7)	1.4<br>(1.2–1.58)	1<br>(0.87–1.12)	9.78<br>(5.73–13.5)	11.2<br>(7.15–14.9)	10.8<br>(6.73–14.5)
Afghanistan	Female	72<br>(67.5–76.3)	0.242<br>(0.196–0.306)	0.0957<br>(0.0748–0.125)	8.82<br>(4.63–12.6)	9.07<br>(4.88–12.8)	8.92<br>(4.73–12.7)
Algeria	Male	81.4<br>(78.6–83.8)	1.44<br>(1.26–1.62)	1.08<br>(0.948–1.23)	4.5<br>(2–6.48)	5.94<br>(3.35–7.97)	5.58<br>(2.99–7.63)
Algeria	Female	81.6<br>(79.3–83.5)	0.0908<br>(0.0779–0.105)	0.034<br>(0.0281–0.04)	3.79<br>(1.68–5.59)	3.89<br>(1.78–5.68)	3.83<br>(1.72–5.63)
Bahrain	Male	78.9<br>(76.9–80.5)	1.55<br>(1.36–1.79)	1.17<br>(1.02–1.37)	3.54<br>(1.92–4.83)	5.09<br>(3.3–6.58)	4.72<br>(2.96–6.16)
Bahrain	Female	80.1<br>(77.9–81.9)	0.334<br>(0.302–0.374)	0.122<br>(0.109–0.14)	3.24<br>(1.29–4.62)	3.57<br>(1.61–4.97)	3.36<br>(1.4–4.75)
Egypt	Male	73.8<br>(70.2–76.9)	2.9<br>(2.71–3.06)	2.1<br>(1.96–2.23)	4.06<br>(1.01–6.41)	6.96<br>(3.89–9.35)	6.16<br>(3.1–8.52)
Egypt	Female	75.5<br>(73–77.7)	0.066<br>(0.0584–0.0732)	0.031<br>(0.0275–0.0323)	4.12<br>(1.79–5.85)	4.18<br>(1.86–5.91)	4.15<br>(1.82–5.88)
Iran (Islamic Republic of)	Male	82.1<br>(79.1–84.1)	1.41<br>(1.31–1.51)	0.863<br>(0.802–0.921)	4.72<br>(1.82–6.86)	6.13<br>(3.19–8.35)	5.59<br>(2.66–7.77)
Iran (Islamic Republic of)	Female	84.5<br>(82–86.4)	0.19<br>(0.175–0.21)	0.0596<br>(0.0544–0.067)	4.07<br>(1.59–5.94)	4.26<br>(1.8–6.12)	4.13<br>(1.66–6)
Iraq	Male	74.9<br>(70.8–78.9)	1.86<br>(1.59–2.13)	1.35<br>(1.14–1.56)	5.87<br>(1.56–9.67)	7.72<br>(3.15–11.6)	7.22<br>(2.7–11.1)
Iraq	Female	80.7<br>(76.7–84)	0.34<br>(0.286–0.393)	0.207<br>(0.17–0.243)	5.96<br>(2.24–8.95)	6.3<br>(2.58–9.27)	6.17<br>(2.45–9.14)
Jordan	Male	84.3<br>(81.3–86.6)	2.17<br>(1.95–2.45)	1.47<br>(1.31–1.65)	5.4<br>(3.29–7.24)	7.58<br>(5.3–9.5)	6.87<br>(4.67–8.77)
Jordan	Female	84.9<br>(82.3–87)	0.393<br>(0.353–0.435)	0.189<br>(0.164–0.221)	4.92<br>(3.01–6.57)	5.31<br>(3.4–6.97)	5.11<br>(3.19–6.78)
Kuwait	Male	82.8<br>(80.3–84.9)	1.25<br>(1.13–1.38)	0.711<br>(0.645–0.793)	2.61<br>(0.599–4.1)	3.86<br>(1.77–5.4)	3.32<br>(1.26–4.84)
Kuwait	Female	89.4<br>(87.1–91.1)	0.167<br>(0.145–0.194)	0.0528<br>(0.0391–0.0673)	2.4<br>(0.571–3.81)	2.57<br>(0.736–3.99)	2.45<br>(0.619–3.87)
Lebanon	Male	79.9<br>(77.6–81.8)	2.39<br>(2.16–2.68)	1.68<br>(1.52–1.88)	4.2<br>(2.19–6.23)	6.6<br>(4.44–8.64)	5.89<br>(3.77–7.93)
Lebanon	Female	84.5<br>(82.2–86.4)	1.2<br>(1.08–1.35)	0.682<br>(0.612–0.772)	3.55<br>(1.47–5.39)	4.75<br>(2.68–6.64)	4.23<br>(2.14–6.12)

Supplementary Results Table S3. Life expectancy at birth by location, sex, and scenario

Location	Sex	2050			Change between 2022 and 2050		
		Reference	Difference between Elimination-2023 and Reference	Difference between Elimination-2050 and Reference	Reference	Elimination - 2023	Elimination - 2050
Libya	Male	74.1 (69.8–77.9)	2.3 (2.01–2.63)	1.52 (1.33–1.72)	4.36 (0.365–7.98)	6.66 (2.48–10.4)	5.88 (1.78–9.56)
Libya	Female	77.8 (73.5–81.6)	0.0653 (0.0564–0.0745)	0.0164 (0.0144–0.0184)	3.83 (0.352–6.62)	3.89 (0.419–6.68)	3.84 (0.368–6.64)
Morocco	Male	77.6 (73.9–81.1)	0.987 (0.851–1.13)	0.496 (0.42–0.588)	5.51 (2.07–8.37)	6.49 (3.08–9.31)	6 (2.6–8.84)
Morocco	Female	79.6 (75.5–83.3)	0.0708 (0.0545–0.0915)	0.0113 (0.00921–0.0144)	5.09 (1.26–8.15)	5.16 (1.36–8.2)	5.1 (1.28–8.16)
Oman	Male	77.8 (74.6–80.5)	0.568 (0.518–0.617)	0.304 (0.281–0.331)	3.17 (0.408–5.21)	3.74 (0.97–5.79)	3.47 (0.702–5.53)
Oman	Female	83.1 (79.2–86.5)	0.104 (0.0936–0.115)	0.0425 (0.0387–0.0468)	3.32 (-0.145–6.07)	3.43 (-0.0344–6.17)	3.36 (-0.102–6.12)
Palestine	Male	77.2 (72.7–79.7)	1.65 (1.45–1.82)	1.06 (0.936–1.17)	5.02 (0.538–9.64)	6.67 (1.96–11.3)	6.08 (1.45–10.7)
Palestine	Female	80.6 (77.7–82.7)	0.187 (0.172–0.202)	0.0542 (0.0473–0.0641)	4.1 (1.15–6.66)	4.28 (1.33–6.85)	4.15 (1.2–6.72)
Qatar	Male	81.1 (78.9–83.3)	0.782 (0.683–0.905)	0.389 (0.337–0.447)	3.26 (1.54–4.69)	4.04 (2.27–5.53)	3.65 (1.9–5.11)
Qatar	Female	81.5 (79.3–83.5)	0.0472 (0.0425–0.0533)	0.012 (0.00843–0.0165)	1.42 (-0.287–2.83)	1.47 (-0.241–2.88)	1.44 (-0.278–2.85)
Saudi Arabia	Male	75.5 (71.7–78.5)	1.18 (1.03–1.37)	0.678 (0.57–0.814)	2.49 (-0.82–4.88)	3.67 (0.476–5.97)	3.17 (-0.0102–5.48)
Saudi Arabia	Female	78.7 (74.9–81.6)	0.142 (0.119–0.177)	0.05 (0.0397–0.0647)	2.84 (-0.0445–5.13)	2.98 (0.113–5.27)	2.89 (0.0124–5.18)
Sudan	Male	74.3 (66.4–79.4)	1.21 (0.701–1.45)	0.699 (0.382–0.829)	5.84 (-1.38–10.1)	7.05 (-0.585–11.3)	6.54 (-0.926–10.8)
Sudan	Female	76.7 (67.8–82.5)	0.152 (0.089–0.193)	0.0249 (0.0132–0.031)	5.42 (-2.91–10.4)	5.57 (-2.79–10.5)	5.44 (-2.89–10.4)
Syrian Arab Republic	Male	72 (62.4–79.6)	1.49 (0.957–1.96)	0.846 (0.552–1.13)	4.05 (-9–17.4)	5.54 (-7.74–19.1)	4.89 (-8.28–18.3)
Syrian Arab Republic	Female	76.8 (70.8–81.5)	0.207 (0.146–0.283)	0.0364 (0.0253–0.0519)	3.25 (-3.71–10.2)	3.45 (-3.53–10.4)	3.28 (-3.68–10.3)
Tunisia	Male	79.5 (76.1–82.7)	1.87 (1.6–2.15)	1.27 (1.09–1.45)	5.49 (3.41–7.36)	7.37 (5.19–9.28)	6.76 (4.64–8.7)
Tunisia	Female	84.2 (81–87.2)	0.136 (0.117–0.157)	0.0334 (0.029–0.0391)	4.94 (2.57–7)	5.07 (2.72–7.13)	4.97 (2.61–7.03)
Turkey	Male	79.6 (77.6–81.7)	1.76 (1.56–1.99)	0.948 (0.841–1.05)	4.61 (3.43–5.56)	6.37 (5.06–7.44)	5.55 (4.29–6.58)
Turkey	Female	83.5 (81.5–85.3)	0.285 (0.255–0.32)	0.0774 (0.067–0.0899)	3.39 (1.94–4.52)	3.68 (2.24–4.82)	3.47 (2.02–4.6)
United Arab Emirates	Male	81.8 (79.6–84)	0.963 (0.881–1.05)	0.648 (0.584–0.715)	2.83 (1.04–4.17)	3.79 (1.91–5.19)	3.47 (1.61–4.87)
United Arab Emirates	Female	73.6 (72.3–74.8)	0.122 (0.111–0.136)	0.0126 (0.0109–0.0148)	1.72 (0.533–2.55)	1.84 (0.667–2.68)	1.73 (0.547–2.57)
Yemen	Male	72.9 (67.6–77.5)	1.99 (1.67–2.26)	1.41 (1.19–1.6)	6.52 (2.1–11.1)	8.51 (4.21–13)	7.93 (3.6–12.4)
Yemen	Female	77 (71.7–82)	0.648 (0.511–0.826)	0.273 (0.217–0.361)	6.79 (1.9–11.3)	7.44 (2.65–11.9)	7.06 (2.22–11.6)
South Asia	Male	76.4 (72.5–79.5)	1.23 (1.13–1.32)	0.797 (0.736–0.851)	6.93 (3.56–9.76)	8.15 (4.83–11)	7.72 (4.37–10.6)
South Asia	Female	79.5 (75.7–82.8)	0.301 (0.26–0.345)	0.157 (0.135–0.18)	6.74 (3.2–9.69)	7.05 (3.52–9.99)	6.9 (3.35–9.86)
South Asia	Male	76.4 (72.5–79.5)	1.23 (1.13–1.32)	0.797 (0.736–0.851)	6.93 (3.56–9.76)	8.15 (4.83–11)	7.72 (4.37–10.6)
South Asia	Female	79.5 (75.7–82.8)	0.301 (0.26–0.345)	0.157 (0.135–0.18)	6.74 (3.2–9.69)	7.05 (3.52–9.99)	6.9 (3.35–9.86)
Bangladesh	Male	79.3 (74.9–82.4)	1.96 (1.71–2.23)	1.43 (1.26–1.64)	6.25 (2.89–9.19)	8.21 (4.92–11.1)	7.69 (4.38–10.6)
Bangladesh	Female	81.7 (77.4–84.8)	0.235 (0.196–0.285)	0.116 (0.0952–0.14)	6.1 (2.7–8.99)	6.33 (2.97–9.22)	6.21 (2.84–9.11)
Bhutan	Male	78.5 (74.4–81.7)	1.07 (0.909–1.23)	0.784 (0.653–0.92)	7.03 (3.56–10.1)	8.09 (4.62–11.2)	7.81 (4.3–10.9)
Bhutan	Female	80.9 (77–84)	0.257 (0.21–0.335)	0.0699 (0.0576–0.0926)	6.76 (3.48–9.55)	7.02 (3.74–9.79)	6.83 (3.55–9.63)
India	Male	76.3 (72.3–79.6)	1.14 (1.05–1.25)	0.72 (0.658–0.783)	6.78 (3.33–9.42)	7.93 (4.52–10.6)	7.5 (4.06–10.2)
India	Female	79.9 (76.2–83.4)	0.314 (0.263–0.36)	0.171 (0.143–0.198)	6.65 (3.07–9.7)	6.97 (3.4–10)	6.82 (3.24–9.88)
Nepal	Male	77.2 (73.1–80.4)	1.25 (1.11–1.38)	0.866 (0.768–0.96)	7.08 (3.8–9.74)	8.33 (5.1–11)	7.94 (4.66–10.6)
Nepal	Female	80.7 (76.9–83.9)	0.513 (0.42–0.609)	0.276 (0.226–0.327)	7.24 (3.98–9.91)	7.75 (4.56–10.4)	7.52 (4.27–10.2)
Pakistan	Male	75.3 (70.6–79.6)	1.27 (1.1–1.43)	0.869 (0.754–0.969)	8.01 (4.23–10.9)	9.28 (5.46–12.1)	8.87 (5.04–11.7)
Pakistan	Female	75.9 (71.3–79.5)	0.197 (0.162–0.242)	0.047 (0.0357–0.0637)	7.45 (3.66–10.3)	7.65 (3.87–10.5)	7.5 (3.71–10.4)
Southeast Asia, East Asia, and Oceania	Male	77.2 (75–78.9)	2.35 (2.19–2.5)	1.57 (1.46–1.68)	3.82 (2.39–4.94)	6.16 (4.62–7.38)	5.39 (3.88–6.57)
Southeast Asia, East Asia, and Oceania	Female	82.9 (80.9–84.7)	0.61 (0.559–0.671)	0.383 (0.34–0.437)	3.7 (1.9–5.07)	4.31 (2.48–5.69)	4.08 (2.25–5.46)
East Asia	Male	78.7 (76.3–80.8)	2.56 (2.34–2.79)	1.75 (1.61–1.91)	3.53 (2.32–4.38)	6.08 (4.75–7.09)	5.28 (3.97–6.24)
East Asia	Female	84.7 (82.5–86.7)	0.741 (0.673–0.822)	0.492 (0.435–0.561)	3.63 (2.06–4.83)	4.37 (2.75–5.63)	4.12 (2.51–5.38)
China	Male	78.8 (76.3–80.9)	2.57 (2.34–2.8)	1.76 (1.61–1.93)	3.54 (2.36–4.37)	6.1 (4.78–7.11)	5.3 (4.01–6.25)
China	Female	84.7 (82.5–86.8)	0.756 (0.685–0.842)	0.506 (0.447–0.577)	3.67 (2.1–4.87)	4.42 (2.81–5.69)	4.17 (2.57–5.44)
Democratic People's Republic of Korea	Male	74.8 (71.1–77.7)	2.61 (2.35–2.87)	1.66 (1.48–1.83)	4.51 (1.76–6.68)	7.12 (4.4–9.36)	6.16 (3.44–8.39)
Democratic People's Republic of Korea	Female	79.6 (75.6–82.6)	0.542 (0.458–0.636)	0.207 (0.178–0.24)	3.35 (0.564–5.52)	3.89 (1.13–6.06)	3.56 (0.782–5.73)
Taiwan (Province of China)	Male	80.4 (79.6–81.2)	1.69 (1.59–1.78)	1.03 (0.973–1.09)	2.38 (1.82–3.04)	4.07 (3.43–4.76)	3.42 (2.81–4.09)
Taiwan (Province of China)	Female	86.5 (85.7–87.2)	0.072 (0.0656–0.0796)	0.00691 (0.00627–0.00768)	1.89 (1.24–2.55)	1.96 (1.31–2.62)	1.9 (1.25–2.56)
Oceania	Male	71.5 (67.1–74.9)	1.46 (1.27–1.66)	0.738 (0.63–0.867)	6.66 (2.6–9.91)	8.13 (4.17–11.4)	7.4 (3.4–10.7)
Oceania	Female	74 (69.3–77.7)	0.733 (0.61–0.876)	0.307 (0.247–0.385)	6.12 (1.71–10.2)	6.86 (2.6–10.8)	6.43 (2.1–10.5)

Supplementary Results Table S3. Life expectancy at birth by location, sex, and scenario

		2050			Change between 2022 and 2050		
Location	Sex	Reference	Difference between Elimination-2023 and Reference	Difference between Elimination-2050 and Reference	Reference	Elimination - 2023	Elimination - 2050
American Samoa	Male	72.4 (69.4–74.7)	1.73 (1.6–1.88)	1.06 (0.96–1.18)	3 (0.768–4.77)	4.73 (2.51–6.49)	4.06 (1.89–5.8)
American Samoa	Female	75.1 (71.8–77.8)	0.633 (0.55–0.735)	0.296 (0.246–0.361)	2.21 (-0.255–4.25)	2.85 (0.398–4.88)	2.51 (0.0611–4.53)
Cook Islands	Male	75.6 (73.1–77.8)	1.34 (1.21–1.46)	0.709 (0.631–0.792)	2.49 (0.496–3.95)	3.84 (1.85–5.34)	3.2 (1.23–4.71)
Cook Islands	Female	83 (80.5–85.3)	0.242 (0.205–0.289)	0.0599 (0.0475–0.0778)	3.12 (1.11–4.63)	3.36 (1.38–4.87)	3.17 (1.18–4.69)
Fiji	Male	70.9 (66.9–74.6)	1.5 (1.3–1.71)	0.957 (0.801–1.13)	4.87 (2.11–7.21)	6.38 (3.69–8.66)	5.83 (3.14–8.11)
Fiji	Female	75.4 (71.1–78.9)	0.428 (0.355–0.512)	0.217 (0.171–0.275)	4.97 (1.72–7.52)	5.39 (2.2–7.92)	5.18 (1.97–7.71)
Guam	Male	80.6 (77.8–83)	1.66 (1.53–1.77)	0.898 (0.818–0.986)	6.47 (3.66–9)	8.13 (5.37–10.7)	7.37 (4.6–9.88)
Guam	Female	89.7 (86.7–92.2)	0.756 (0.669–0.869)	0.315 (0.27–0.377)	6.15 (3.34–8.65)	6.91 (4.2–9.39)	6.47 (3.72–8.94)
Kiribati	Male	67 (62.6–70.9)	3.32 (2.96–3.85)	2.39 (2.11–2.75)	5.72 (2.36–8.38)	9.04 (5.83–11.6)	8.11 (4.9–10.7)
Kiribati	Female	72.2 (67.9–76.1)	1.38 (1.18–1.61)	0.945 (0.79–1.13)	5.1 (1.57–8.13)	6.49 (3.05–9.39)	6.05 (2.62–8.95)
Marshall Islands	Male	67.6 (62.6–71.9)	2.31 (1.99–2.66)	1.51 (1.3–1.76)	4.01 (0.301–6.91)	6.32 (2.76–9.07)	5.51 (1.95–8.28)
Marshall Islands	Female	70.4 (65.2–74.8)	0.693 (0.55–0.825)	0.333 (0.255–0.417)	3.45 (-0.601–6.83)	4.15 (0.121–7.43)	3.79 (-0.24–7.11)
Micronesia (Federated States of)	Male	67.6 (63.2–71.4)	2.75 (2.43–3.07)	1.83 (1.59–2.1)	2.9 (-0.0819–5.29)	5.65 (2.79–8.02)	4.73 (1.86–7.06)
Micronesia (Federated States of)	Female	72.3 (68.2–76)	1.15 (0.968–1.39)	0.705 (0.565–0.881)	2.49 (-0.64–5.16)	3.65 (0.668–6.17)	3.2 (0.194–5.75)
Nauru	Male	63.1 (57.4–67.9)	2.5 (2.28–2.84)	1.74 (1.58–1.98)	3.74 (-0.567–7.12)	6.24 (2.13–9.57)	5.48 (1.37–8.8)
Nauru	Female	70.3 (64.8–74.8)	1.24 (0.981–1.59)	0.714 (0.529–0.964)	4.44 (-0.31–7.66)	5.68 (1.25–8.81)	5.15 (0.649–8.34)
Niue	Male	69.8 (66.8–72.3)	1.69 (1.51–1.87)	1.16 (1.04–1.29)	4.16 (1.43–6.38)	5.84 (3.06–8.14)	5.32 (2.54–7.59)
Niue	Female	74 (70.6–76.9)	0.52 (0.419–0.615)	0.198 (0.154–0.249)	3.91 (0.885–6.39)	4.43 (1.42–6.87)	4.1 (1.11–6.55)
Northern Mariana Islands	Male	73.3 (71.1–75.2)	1.7 (1.59–1.8)	0.946 (0.876–1.01)	2.18 (0.0927–3.72)	3.89 (1.78–5.48)	3.13 (1.05–4.68)
Northern Mariana Islands	Female	78.6 (76.1–80.7)	0.432 (0.385–0.48)	0.148 (0.127–0.172)	2.34 (0.19–4.01)	2.78 (0.655–4.43)	2.49 (0.359–4.14)
Palau	Male	71.3 (67.4–74.3)	2.27 (2.06–2.47)	1.55 (1.43–1.67)	3.51 (0.717–5.67)	5.78 (3.06–7.97)	5.06 (2.31–7.25)
Palau	Female	72.7 (69.2–75.2)	0.567 (0.518–0.62)	0.234 (0.209–0.261)	2.24 (-0.249–4)	2.8 (0.361–4.58)	2.47 (0.011–4.24)
Papua New Guinea	Male	71.8 (67.1–75.5)	1.31 (1.11–1.52)	0.571 (0.471–0.701)	6.96 (2.76–10.8)	8.27 (4.15–12)	7.53 (3.42–11.3)
Papua New Guinea	Female	73.7 (68.8–77.6)	0.737 (0.607–0.883)	0.288 (0.226–0.368)	6.61 (2.24–10.8)	7.35 (3.14–11.5)	6.9 (2.62–11.1)
Samoa	Male	72.8 (69.3–75.8)	2.14 (1.9–2.4)	1.29 (1.13–1.47)	4 (1.05–6.61)	6.14 (3.33–8.59)	5.29 (2.47–7.79)
Samoa	Female	73.9 (70.1–77.3)	0.747 (0.606–0.913)	0.263 (0.199–0.337)	2.92 (-0.124–5.7)	3.67 (0.768–6.31)	3.19 (0.203–5.88)
Solomon Islands	Male	67.4 (62.6–71.4)	2.74 (2.45–2.99)	2.15 (1.91–2.36)	3.74 (0.36–6.9)	6.48 (3.03–9.46)	5.89 (2.41–8.9)
Solomon Islands	Female	71.6 (66.5–75.9)	1.01 (0.825–1.2)	0.657 (0.529–0.796)	3.3 (-1.19–6.84)	4.31 (0.0569–7.73)	3.96 (-0.362–7.41)
Tokelau	Male	73.2 (69.9–76)	1.71 (1.5–1.95)	0.973 (0.832–1.15)	4.98 (1.97–7.08)	6.69 (3.72–8.76)	5.95 (2.99–8)
Tokelau	Female	74.6 (70.5–78)	0.644 (0.54–0.752)	0.277 (0.225–0.34)	5.48 (1.85–8.01)	6.12 (2.5–8.62)	5.76 (2.15–8.26)
Tonga	Male	74.2 (71–77)	2.3 (2.15–2.45)	1.63 (1.53–1.73)	3.52 (1.33–5.32)	5.82 (3.59–7.7)	5.15 (2.93–7.01)
Tonga	Female	79 (75.6–81.9)	0.513 (0.461–0.569)	0.176 (0.155–0.2)	3.22 (0.849–5.06)	3.73 (1.36–5.58)	3.39 (1.02–5.24)
Tuvalu	Male	69.8 (65.4–73.2)	2.61 (2.31–2.91)	1.78 (1.54–2.02)	3.83 (0.671–6.37)	6.44 (3.46–8.85)	5.61 (2.6–8.02)
Tuvalu	Female	74.5 (70.4–78)	1.04 (0.862–1.23)	0.6 (0.48–0.748)	3.68 (0.541–6.2)	4.72 (1.72–7.14)	4.28 (1.26–6.74)
Vanuatu	Male	69.5 (65.7–73)	1.65 (1.42–1.85)	1.01 (0.867–1.15)	7.27 (3.37–10.8)	8.92 (5.13–12.5)	8.28 (4.46–11.8)
Vanuatu	Female	74.7 (71.1–78)	0.213 (0.178–0.252)	0.0571 (0.0475–0.0679)	5.79 (1.9–9.34)	6.01 (2.13–9.54)	5.85 (1.96–9.4)
Southeast Asia	Male	75.2 (73.1–77.1)	1.89 (1.8–2)	1.18 (1.12–1.25)	5.16 (3.31–6.82)	7.06 (5.14–8.74)	6.34 (4.44–8.02)
Southeast Asia	Female	80.3 (78.1–82.4)	0.271 (0.257–0.286)	0.105 (0.0996–0.112)	4.55 (2.65–6.17)	4.82 (2.91–6.44)	4.65 (2.75–6.28)
Cambodia	Male	72.9 (69.9–75.9)	2.4 (2.21–2.61)	1.92 (1.76–2.08)	5.88 (4.17–7.42)	8.29 (6.47–10)	7.8 (5.99–9.5)
Cambodia	Female	77.5 (74.4–80.6)	0.404 (0.372–0.442)	0.177 (0.163–0.195)	5.24 (3.2–6.93)	5.65 (3.62–7.35)	5.42 (3.39–7.11)
Indonesia	Male	75.7 (72.8–78.5)	2.08 (1.93–2.25)	1.27 (1.17–1.38)	6.22 (4.19–7.99)	8.3 (6.21–10.1)	7.49 (5.45–9.31)
Indonesia	Female	78.9 (76.3–81.6)	0.252 (0.228–0.276)	0.0976 (0.0867–0.113)	5.43 (3.51–7.18)	5.68 (3.75–7.44)	5.52 (3.6–7.29)
Lao People's Democratic Republic	Male	74.2 (70.8–77.2)	2.77 (2.51–3)	2.14 (1.94–2.31)	8.29 (6.2–10.1)	11.1 (8.92–13)	10.4 (8.28–12.3)
Lao People's Democratic Republic	Female	78.3 (74.6–81.1)	0.508 (0.451–0.58)	0.283 (0.252–0.322)	7.77 (5.49–9.83)	8.27 (5.99–10.4)	8.05 (5.77–10.1)
Malaysia	Male	77.8 (75.8–79.2)	1.65 (1.54–1.76)	0.857 (0.8–0.924)	5.2 (3.33–6.62)	6.85 (4.86–8.33)	6.06 (4.12–7.52)
Malaysia	Female	80.3 (78.8–81.5)	0.139 (0.131–0.15)	0.0464 (0.0398–0.054)	3.31 (1.8–4.42)	3.45 (1.94–4.57)	3.36 (1.84–4.47)
Maldives	Male	84.4 (81.7–86.7)	2.15 (1.98–2.33)	1.74 (1.58–1.89)	4.64 (2.65–6.36)	6.8 (4.66–8.63)	6.38 (4.25–8.19)
Maldives	Female	87 (84.1–89.3)	0.401 (0.364–0.445)	0.159 (0.143–0.178)	4.7 (2.44–6.67)	5.1 (2.83–7.07)	4.86 (2.59–6.84)
Mauritius	Male	76.7 (74.8–78.1)	1.8 (1.69–1.92)	1.28 (1.2–1.36)	4.81 (3.07–6.31)	6.62 (4.79–8.19)	6.09 (4.29–7.64)
Mauritius	Female	82.1 (80.2–83.6)	0.177 (0.168–0.185)	0.0384 (0.0362–0.0407)	3.94 (2.11–5.29)	4.12 (2.29–5.47)	3.98 (2.15–5.33)

Supplementary Results Table S3. Life expectancy at birth by location, sex, and scenario

		2050			Change between 2022 and 2050		
Location	Sex	Reference	Difference between Elimination-2023 and Reference	Difference between Elimination-2050 and Reference	Reference	Elimination - 2023	Elimination - 2050
Myanmar	Male	71.5 (67.1–74.6)	1.51 (1.3–1.67)	0.981 (0.84–1.09)	5.32 (1.95–9.2)	6.83 (3.31–10.7)	6.3 (2.84–10.1)
Myanmar	Female	79 (74.8–81.8)	0.509 (0.428–0.565)	0.272 (0.233–0.303)	6.19 (2.88–9.07)	6.7 (3.4–9.56)	6.47 (3.17–9.33)
Philippines	Male	74.9 (72.2–77.9)	1.53 (1.45–1.62)	0.831 (0.782–0.882)	5.87 (4.23–7.21)	7.4 (5.75–8.7)	6.7 (5.05–8.02)
Philippines	Female	79.5 (77.1–81.7)	0.381 (0.362–0.403)	0.175 (0.166–0.188)	4.2 (2.63–5.52)	4.58 (3.01–5.91)	4.37 (2.8–5.7)
Seychelles	Male	75.9 (74.3–77.3)	1.76 (1.65–1.87)	1.28 (1.2–1.38)	4.54 (3.37–5.49)	6.3 (5.13–7.31)	5.82 (4.65–6.82)
Seychelles	Female	80.4 (78.7–81.8)	0.196 (0.184–0.21)	0.0397 (0.037–0.0423)	3.18 (1.8–4.25)	3.38 (2–4.45)	3.22 (1.84–4.29)
Sri Lanka	Male	78.8 (74.1–82.9)	1.09 (0.957–1.19)	0.668 (0.583–0.727)	3.89 (-0.0564–7.91)	4.98 (0.909–9.05)	4.56 (0.548–8.6)
Sri Lanka	Female	84.4 (80.5–87.8)	0.13 (0.113–0.152)	0.0429 (0.0362–0.0505)	3.53 (0.619–6.48)	3.66 (0.733–6.61)	3.57 (0.657–6.52)
Thailand	Male	78.7 (75.1–82.2)	1.83 (1.69–2)	1.17 (1.08–1.27)	5.01 (3.4–6.18)	6.84 (5.08–8.19)	6.18 (4.45–7.46)
Thailand	Female	84.8 (82.3–87.5)	0.209 (0.187–0.234)	0.0389 (0.0349–0.0437)	3.58 (2.04–4.8)	3.79 (2.21–5.04)	3.62 (2.07–4.84)
Timor-Leste	Male	75.1 (71.1–78.3)	2.09 (1.85–2.3)	1.24 (1.09–1.37)	6.04 (3.18–8.41)	8.14 (5.21–10.5)	7.28 (4.38–9.67)
Timor-Leste	Female	79 (75.2–81.9)	0.226 (0.203–0.248)	0.0712 (0.0635–0.0786)	6.87 (4.04–9.13)	7.1 (4.26–9.35)	6.95 (4.11–9.2)
Viet Nam	Male	72.4 (69.8–74.7)	1.82 (1.63–1.98)	1.17 (1.04–1.29)	2.02 (0.102–3.46)	3.83 (1.91–5.3)	3.18 (1.29–4.61)
Viet Nam	Female	82.2 (79.7–84.3)	0.158 (0.142–0.175)	0.0417 (0.0375–0.0463)	3.39 (1.63–4.83)	3.55 (1.8–4.97)	3.43 (1.68–4.86)
Sub-Saharan Africa	Male	71.8 (68.1–74.4)	0.553 (0.513–0.585)	0.314 (0.291–0.338)	9.56 (6.42–11.7)	10.1 (6.96–12.3)	9.88 (6.74–12.1)
Sub-Saharan Africa	Female	75.8 (72.1–78.2)	0.106 (0.0965–0.116)	0.0488 (0.0437–0.0543)	9.19 (5.98–11.2)	9.3 (6.08–11.3)	9.24 (6.03–11.2)
Central Sub-Saharan Africa	Male	70.1 (65.8–73.4)	0.742 (0.666–0.819)	0.473 (0.418–0.53)	8.76 (5.22–11.5)	9.51 (5.98–12.3)	9.24 (5.72–12)
Central Sub-Saharan Africa	Female	74.4 (70.1–77.7)	0.0843 (0.0701–0.101)	0.0356 (0.0283–0.0432)	8.55 (5.5–10.9)	8.63 (5.58–11)	8.58 (5.54–11)
Angola	Male	70.8 (66.4–74.3)	1.08 (0.974–1.17)	0.771 (0.692–0.835)	8.58 (5.61–11)	9.66 (6.68–12.1)	9.35 (6.39–11.8)
Angola	Female	75.3 (70.7–78.8)	0.142 (0.122–0.165)	0.0599 (0.0482–0.0733)	8.76 (5.63–10.9)	8.9 (5.78–11.1)	8.82 (5.7–11)
Central African Republic	Male	61 (53.6–66.5)	0.68 (0.433–0.782)	0.461 (0.291–0.53)	11.7 (4.36–20)	12.4 (4.94–20.8)	12.2 (4.76–20.5)
Central African Republic	Female	67.9 (58.8–73.5)	0.0827 (0.0489–0.103)	0.0346 (0.0196–0.0445)	12 (3.12–20)	12 (3.17–20.1)	12 (3.14–20)
Congo	Male	70.1 (65.6–73.6)	1.15 (1.02–1.26)	0.822 (0.727–0.898)	6.55 (2.76–9.22)	7.7 (3.83–10.4)	7.38 (3.53–10.1)
Congo	Female	72.4 (68–75.9)	0.0788 (0.0636–0.0949)	0.0333 (0.025–0.042)	7.49 (4.29–9.81)	7.57 (4.38–9.88)	7.52 (4.33–9.84)
Democratic Republic of the Congo	Male	70.3 (65.6–73.8)	0.6 (0.524–0.687)	0.349 (0.298–0.412)	8.44 (4.42–11.4)	9.04 (4.99–12)	8.79 (4.76–11.7)
Democratic Republic of the Congo	Female	74.6 (70–78.1)	0.0642 (0.051–0.0827)	0.0279 (0.0214–0.0357)	8.04 (4.6–11)	8.11 (4.66–11)	8.07 (4.63–11)
Equatorial Guinea	Male	70 (64.8–74)	0.887 (0.731–1.01)	0.52 (0.428–0.608)	7.25 (2.93–10.7)	8.14 (3.78–11.7)	7.77 (3.43–11.3)
Equatorial Guinea	Female	75.1 (69.7–79.9)	0.069 (0.0558–0.081)	0.0208 (0.0159–0.0258)	8.72 (4.69–12)	8.79 (4.74–12.1)	8.74 (4.71–12.1)
Gabon	Male	69.8 (65.9–72.9)	0.854 (0.759–0.971)	0.499 (0.444–0.569)	5.94 (3.3–8.1)	6.8 (4.12–9.03)	6.44 (3.79–8.66)
Gabon	Female	76.1 (72.4–79.7)	0.0825 (0.0692–0.0964)	0.0169 (0.0133–0.021)	6.49 (4.32–8.2)	6.57 (4.41–8.28)	6.51 (4.34–8.21)
Eastern Sub-Saharan Africa	Male	72.5 (69.4–74.8)	0.596 (0.557–0.638)	0.374 (0.348–0.403)	9.76 (6.85–11.8)	10.4 (7.45–12.4)	10.1 (7.23–12.2)
Eastern Sub-Saharan Africa	Female	76.6 (73.3–78.8)	0.14 (0.125–0.155)	0.0822 (0.0732–0.0919)	9.3 (6.62–11.2)	9.44 (6.76–11.4)	9.38 (6.7–11.3)
Burundi	Male	71.4 (67.2–75)	0.587 (0.519–0.649)	0.375 (0.327–0.424)	11.8 (7.82–17.3)	12.4 (8.42–17.8)	12.2 (8.21–17.6)
Burundi	Female	75.6 (71.5–78.9)	0.0813 (0.0685–0.0951)	0.0249 (0.019–0.0319)	11 (7.43–15.1)	11.1 (7.51–15.2)	11.1 (7.45–15.1)
Comoros	Male	73.8 (70–76.7)	0.837 (0.775–0.896)	0.583 (0.54–0.625)	6.2 (3.33–8.16)	7.04 (4.16–9.03)	6.79 (3.9–8.77)
Comoros	Female	76 (72.4–78.8)	0.107 (0.0949–0.119)	0.0339 (0.0302–0.0381)	6.23 (3.48–8.24)	6.33 (3.59–8.35)	6.26 (3.52–8.27)
Djibouti	Male	72.7 (68.4–76.2)	1.46 (1.32–1.62)	1.01 (0.916–1.12)	6.57 (4.02–8.54)	8.03 (5.45–10)	7.58 (5.01–9.56)
Djibouti	Female	77.2 (72.9–80.6)	0.207 (0.183–0.231)	0.0857 (0.0751–0.0964)	7.4 (5.07–9.33)	7.61 (5.28–9.54)	7.49 (5.16–9.42)
Eritrea	Male	69.3 (64.5–72.6)	0.524 (0.428–0.619)	0.277 (0.218–0.345)	8.83 (5.44–11.4)	9.36 (5.98–12)	9.11 (5.74–11.7)
Eritrea	Female	74.4 (70.4–77.8)	0.015 (0.0128–0.0172)	0.00388 (0.00321–0.00458)	8.35 (5.63–10.5)	8.37 (5.65–10.5)	8.36 (5.64–10.5)
Ethiopia	Male	75.8 (72.2–78.4)	0.373 (0.338–0.408)	0.214 (0.193–0.236)	9.12 (6.6–11.1)	9.49 (6.98–11.5)	9.33 (6.82–11.3)
Ethiopia	Female	78.9 (75.8–81.4)	0.0188 (0.0155–0.022)	0.00688 (0.00529–0.00856)	8.52 (6.21–10.3)	8.54 (6.23–10.3)	8.53 (6.22–10.3)
Kenya	Male	73.6 (70.7–76.4)	0.467 (0.391–0.527)	0.22 (0.183–0.25)	8.2 (5.77–10)	8.67 (6.28–10.5)	8.42 (6.01–10.2)
Kenya	Female	77.8 (75.1–80.2)	0.0722 (0.0583–0.106)	0.0224 (0.0183–0.0319)	7.19 (5.11–8.93)	7.27 (5.17–9)	7.22 (5.13–8.95)
Madagascar	Male	72 (67.9–75.5)	0.5 (0.424–0.571)	0.205 (0.171–0.238)	11.3 (7.9–15.2)	11.8 (8.44–15.7)	11.5 (8.13–15.4)
Madagascar	Female	73.9 (70–77.2)	0.0674 (0.0582–0.0763)	0.017 (0.0145–0.0196)	10.1 (6.92–13.2)	10.2 (6.99–13.2)	10.1 (6.93–13.2)
Malawi	Male	69.5 (65–72.8)	1.15 (1.04–1.25)	0.904 (0.817–0.985)	10.3 (6.64–13.2)	11.5 (7.75–14.4)	11.2 (7.51–14.1)
Malawi	Female	76.1 (71.9–79.9)	0.121 (0.101–0.143)	0.0703 (0.0565–0.084)	11.2 (8–14)	11.4 (8.13–14.1)	11.3 (8.08–14)
Mozambique	Male	68.7 (64.7–71.8)	0.798 (0.717–0.864)	0.55 (0.492–0.598)	11.8 (8.82–14.4)	12.6 (9.55–15.2)	12.3 (9.33–15)
Mozambique	Female	76 (71.6–80)	0.125 (0.104–0.142)	0.0288 (0.0226–0.0359)	12.9 (9.69–15.4)	13 (9.79–15.6)	12.9 (9.72–15.5)

Supplementary Results Table S3. Life expectancy at birth by location, sex, and scenario

		2050			Change between 2022 and 2050		
Location	Sex	Reference	Difference between Elimination-2023 and Reference	Difference between Elimination-2050 and Reference	Reference	Elimination - 2023	Elimination - 2050
Rwanda	Male	74.4 (70.8–77.2)	1.24 (1.1–1.35)	1.02 (0.908–1.11)	8.32 (6.1–10.3)	9.55 (7.23–11.6)	9.34 (7.04–11.3)
Rwanda	Female	78.1 (75–80.8)	0.644 (0.571–0.734)	0.501 (0.445–0.569)	7.94 (5.74–9.52)	8.58 (6.39–10.2)	8.44 (6.25–10)
Somalia	Male	63.2 (55.8–68.6)	0.815 (0.698–0.939)	0.558 (0.478–0.644)	8.2 (1.96–12.5)	9.01 (2.75–13.4)	8.76 (2.51–13.1)
Somalia	Female	69.1 (61.2–74.4)	0.118 (0.1–0.136)	0.0533 (0.0458–0.0628)	8.87 (1.77–13.2)	8.99 (1.88–13.3)	8.92 (1.83–13.2)
South Sudan	Male	66.9 (59.6–72.6)	0.645 (0.539–0.718)	0.386 (0.321–0.435)	11.7 (5.79–15.7)	12.3 (6.44–16.3)	12.1 (6.2–16)
South Sudan	Female	72 (63.5–77.6)	0.0935 (0.074–0.109)	0.037 (0.0299–0.0445)	11.8 (4.47–16.7)	11.8 (4.55–16.8)	11.8 (4.5–16.7)
Uganda	Male	72.9 (69.3–75.3)	0.357 (0.316–0.391)	0.149 (0.129–0.168)	10.6 (7.93–13.5)	11 (8.28–13.8)	10.8 (8.08–13.6)
Uganda	Female	78.5 (74.9–81)	0.148 (0.128–0.165)	0.0676 (0.0581–0.0759)	9.75 (7.63–12.4)	9.9 (7.77–12.6)	9.82 (7.7–12.5)
United Republic of Tanzania	Male	74.6 (71.4–77.3)	0.767 (0.687–0.854)	0.526 (0.462–0.601)	8.64 (6.31–10.5)	9.41 (7.2–11.2)	9.16 (6.94–11)
United Republic of Tanzania	Female	76.8 (73.8–79.3)	0.322 (0.288–0.359)	0.244 (0.217–0.273)	7.77 (5.67–9.52)	8.09 (6.03–9.84)	8.01 (5.94–9.76)
Zambia	Male	69.8 (66.1–73.4)	0.758 (0.699–0.833)	0.461 (0.42–0.511)	9.04 (6.66–11.4)	9.79 (7.42–12.1)	9.5 (7.13–11.8)
Zambia	Female	75 (70.9–78.9)	0.237 (0.211–0.269)	0.135 (0.119–0.154)	9.73 (7.46–11.8)	9.97 (7.7–12.1)	9.87 (7.6–12)
Southern Sub-Saharan Africa	Male	68.9 (66.5–70.8)	1.08 (1.03–1.14)	0.649 (0.608–0.697)	9.04 (7.05–10.5)	10.1 (8.11–11.6)	9.69 (7.69–11.2)
Southern Sub-Saharan Africa	Female	75.1 (72.9–76.9)	0.274 (0.261–0.286)	0.0901 (0.0828–0.0967)	9 (7.28–10.4)	9.27 (7.55–10.7)	9.09 (7.37–10.5)
Botswana	Male	72.8 (69.9–75.1)	1.67 (1.49–1.84)	1.2 (1.07–1.32)	11.7 (9.43–14.2)	13.4 (11.1–15.8)	12.9 (10.6–15.4)
Botswana	Female	78.2 (75.4–80.5)	0.41 (0.377–0.444)	0.184 (0.167–0.2)	12.1 (9.86–14.5)	12.5 (10.3–14.9)	12.3 (10–14.7)
Eswatini	Male	64.4 (60.1–68.3)	0.576 (0.512–0.639)	0.32 (0.284–0.356)	10.9 (7.51–14.4)	11.4 (8.08–15)	11.2 (7.83–14.7)
Eswatini	Female	73.1 (68.2–78)	0.238 (0.198–0.278)	0.165 (0.136–0.193)	13.3 (10.6–17.6)	13.5 (10.8–17.8)	13.4 (10.7–17.8)
Lesotho	Male	60.6 (56.7–63.8)	2.43 (2.19–2.65)	1.91 (1.72–2.08)	10.9 (7.91–13.2)	13.3 (10.2–15.8)	12.8 (9.74–15.2)
Lesotho	Female	70.1 (65.9–73.7)	0.365 (0.297–0.431)	0.333 (0.268–0.4)	13.6 (10.8–15.9)	14 (11.2–16.3)	14 (11.1–16.2)
Namibia	Male	69.5 (65.6–72.8)	0.846 (0.741–0.934)	0.603 (0.522–0.671)	9.13 (6.62–11.3)	9.98 (7.41–12.1)	9.74 (7.17–11.9)
Namibia	Female	77.5 (73.4–81.2)	0.449 (0.406–0.498)	0.278 (0.25–0.309)	10.2 (8.11–12.1)	10.6 (8.54–12.5)	10.5 (8.37–12.4)
South Africa	Male	69.6 (67.7–71.2)	0.887 (0.843–0.94)	0.438 (0.413–0.464)	8.34 (6.61–9.63)	9.22 (7.51–10.5)	8.77 (7.06–10.1)
South Africa	Female	75.8 (74.3–77.1)	0.258 (0.244–0.271)	0.0461 (0.0427–0.0496)	7.99 (6.6–9.2)	8.25 (6.86–9.46)	8.03 (6.65–9.25)
Zimbabwe	Male	65.8 (61.8–69.2)	1.8 (1.64–1.94)	1.42 (1.3–1.53)	9.5 (6.13–12.2)	11.3 (7.87–14.1)	10.9 (7.52–13.7)
Zimbabwe	Female	71.3 (67.2–74.8)	0.334 (0.304–0.363)	0.249 (0.225–0.274)	10.2 (7.2–12.5)	10.5 (7.52–12.8)	10.4 (7.44–12.7)
Western Sub-Saharan Africa	Male	72.3 (68–75.6)	0.349 (0.287–0.384)	0.144 (0.119–0.162)	9.25 (5.15–11.9)	9.6 (5.52–12.2)	9.39 (5.3–12)
Western Sub-Saharan Africa	Female	75.7 (71–78.9)	0.0485 (0.0388–0.0565)	0.0155 (0.0124–0.0182)	9.15 (5–11.7)	9.2 (5.05–11.8)	9.16 (5.01–11.7)
Benin	Male	71.6 (62.1–75.7)	0.299 (0.159–0.345)	0.0662 (0.0325–0.0782)	8.28 (-0.327–12)	8.58 (-0.187–12.3)	8.34 (-0.292–12.1)
Benin	Female	76 (61.3–79.9)	0.0455 (0.0217–0.0566)	0.0121 (0.00585–0.0155)	7.73 (-6.46–11.8)	7.77 (-6.43–11.8)	7.74 (-6.45–11.8)
Burkina Faso	Male	69.4 (61–73.5)	0.288 (0.109–0.348)	0.107 (0.0313–0.14)	9.02 (0.275–13)	9.31 (0.546–13.3)	9.13 (0.381–13.1)
Burkina Faso	Female	74.2 (60.7–78.8)	0.0148 (0.00576–0.0189)	0.00249 (0.00025–0.00342)	9.09 (-3.82–13.4)	9.1 (-3.81–13.4)	9.09 (-3.82–13.4)
Cabo Verde	Male	74.8 (72.4–77.2)	0.533 (0.484–0.589)	0.214 (0.19–0.241)	5.16 (3.79–6.29)	5.7 (4.32–6.85)	5.38 (4.01–6.51)
Cabo Verde	Female	82.3 (80.2–84.7)	0.0943 (0.0821–0.108)	0.0242 (0.0199–0.0289)	4.05 (2.75–5.09)	4.15 (2.84–5.2)	4.08 (2.77–5.12)
Cameroon	Male	70.8 (64.9–74.9)	0.403 (0.282–0.487)	0.164 (0.111–0.21)	7.77 (2.38–10.7)	8.17 (2.71–11)	7.93 (2.52–10.8)
Cameroon	Female	75.3 (70.2–79.2)	0.0378 (0.03–0.0424)	0.0146 (0.0099–0.0188)	8.27 (4.57–11.1)	8.31 (4.59–11.1)	8.29 (4.58–11.1)
Chad	Male	69.8 (64.8–74.2)	0.433 (0.371–0.482)	0.184 (0.155–0.207)	10.7 (7.09–13.6)	11.1 (7.52–14)	10.8 (7.27–13.8)
Chad	Female	73.7 (68.8–77.7)	0.0684 (0.059–0.0773)	0.0104 (0.00898–0.0117)	11.4 (7.38–14.5)	11.5 (7.45–14.5)	11.4 (7.39–14.5)
Côte d'Ivoire	Male	72.6 (66.8–76.8)	0.515 (0.269–0.634)	0.203 (0.102–0.252)	9.82 (4.31–13)	10.3 (4.52–13.6)	10 (4.41–13.2)
Côte d'Ivoire	Female	77.4 (71.1–81.2)	0.088 (0.0489–0.11)	0.0194 (0.0109–0.0261)	9.57 (4–12.8)	9.66 (4.06–12.9)	9.59 (4.02–12.9)
Gambia	Male	72.2 (68.4–75.4)	0.591 (0.519–0.664)	0.275 (0.235–0.317)	8.49 (5.66–10.7)	9.08 (6.32–11.3)	8.76 (5.98–11)
Gambia	Female	76.3 (73–79.4)	0.036 (0.0302–0.0425)	0.0143 (0.0114–0.0177)	8.43 (6.15–10.5)	8.47 (6.19–10.6)	8.45 (6.17–10.5)
Ghana	Male	70.1 (63.7–73.7)	0.517 (0.388–0.575)	0.356 (0.259–0.396)	5.83 (-0.704–8.87)	6.35 (-0.243–9.37)	6.19 (-0.386–9.22)
Ghana	Female	75.8 (70.5–79.4)	0.0887 (0.0656–0.1)	0.0282 (0.0207–0.0314)	6.38 (0.57–9.15)	6.47 (0.62–9.24)	6.41 (0.587–9.18)
Guinea	Male	71.5 (61.9–76.3)	0.773 (0.507–0.895)	0.371 (0.248–0.429)	10.2 (1.93–14.1)	11 (2.63–14.8)	10.6 (2.26–14.5)
Guinea	Female	73.7 (65–78.1)	0.0951 (0.0591–0.113)	0.0312 (0.0198–0.0375)	9.46 (1.15–13.6)	9.55 (1.2–13.7)	9.49 (1.16–13.6)
Guinea-Bissau	Male	67.5 (63.4–70.6)	0.647 (0.574–0.717)	0.434 (0.381–0.489)	11.6 (7.01–27.7)	12.2 (7.71–28.3)	12 (7.48–28.1)
Guinea-Bissau	Female	73 (69–76.1)	0.0791 (0.0679–0.0918)	0.0372 (0.031–0.0447)	11.4 (7.18–27.9)	11.4 (7.26–28)	11.4 (7.22–27.9)
Liberia	Male	72.4 (64.4–77.4)	0.442 (0.285–0.516)	0.228 (0.145–0.281)	7.7 (1.11–11.7)	8.14 (1.57–12.2)	7.93 (1.37–12)
Liberia	Female	74 (66.6–78.6)	0.0816 (0.0512–0.102)	0.0292 (0.0195–0.0387)	7.84 (1.25–11.6)	7.92 (1.31–11.7)	7.86 (1.27–11.6)

Supplementary Results Table S3. Life expectancy at birth by location, sex, and scenario

Location	Sex	2050			Change between 2022 and 2050		
		Reference	Difference between Elimination-2023 and Reference	Difference between Elimination-2050 and Reference	Reference	Elimination - 2023	Elimination - 2050
Mali	Male	72.6 (67.9–76.1)	0.795 (0.693–0.875)	0.517 (0.443–0.57)	10.5 (6.53–13.6)	11.3 (7.26–14.5)	11 (7–14.2)
Mali	Female	74 (69.4–77.8)	0.121 (0.0955–0.147)	0.0564 (0.0434–0.0718)	9.94 (6.18–13.1)	10.1 (6.29–13.2)	9.99 (6.23–13.1)
Mauritania	Male	75.8 (72–79.3)	0.424 (0.36–0.497)	0.141 (0.113–0.178)	6.13 (3.12–12.1)	6.55 (3.53–12.5)	6.27 (3.26–12.2)
Mauritania	Female	77 (73.6–80.1)	0.0751 (0.0589–0.0959)	0.0126 (0.00918–0.0174)	6.35 (3.62–10)	6.42 (3.71–10.1)	6.36 (3.63–10)
Niger	Male	70.4 (64.3–75.2)	0.269 (0.174–0.318)	0.0821 (0.0431–0.0982)	7.58 (0.245–11.3)	7.85 (0.426–11.6)	7.67 (0.305–11.4)
Niger	Female	71.7 (64.1–76.3)	0.0307 (0.021–0.0384)	0.00613 (0.00397–0.00808)	6.67 (-0.409–10.7)	6.7 (-0.384–10.7)	6.68 (-0.404–10.7)
Nigeria	Male	73.8 (67–78.1)	0.2 (0.126–0.238)	0.0373 (0.0246–0.0475)	9.76 (3.11–13.2)	9.96 (3.27–13.4)	9.8 (3.14–13.2)
Nigeria	Female	76.7 (70.4–80.7)	0.0284 (0.0185–0.0349)	0.00883 (0.00651–0.0111)	9.77 (3.75–12.8)	9.8 (3.76–12.8)	9.78 (3.75–12.8)
Sao Tome and Principe	Male	75.3 (73.1–77.1)	0.637 (0.569–0.733)	0.349 (0.305–0.405)	4.61 (2.93–6.2)	5.24 (3.54–6.85)	4.95 (3.27–6.56)
Sao Tome and Principe	Female	78 (75.5–80.2)	0.0961 (0.0805–0.114)	0.0509 (0.042–0.0609)	4.36 (2.6–6.05)	4.45 (2.7–6.14)	4.41 (2.66–6.1)
Senegal	Male	75.2 (72–78.1)	0.362 (0.315–0.407)	0.121 (0.101–0.143)	7.64 (5.62–9.37)	8 (6–9.73)	7.76 (5.75–9.48)
Senegal	Female	78.5 (75.3–81.3)	0.0217 (0.018–0.0261)	0.00179 (0.00144–0.00224)	7.72 (5.71–9.41)	7.74 (5.73–9.43)	7.72 (5.71–9.41)
Sierra Leone	Male	72.6 (65.2–77.1)	0.531 (0.33–0.63)	0.258 (0.166–0.318)	10.7 (3.92–15.1)	11.3 (4.24–15.7)	11 (4.06–15.4)
Sierra Leone	Female	73.8 (64.2–78.5)	0.0877 (0.0455–0.117)	0.0169 (0.00935–0.0237)	9.84 (1.37–13.8)	9.93 (1.43–13.8)	9.86 (1.38–13.8)
Togo	Male	70.9 (64.9–75.2)	0.601 (0.348–0.678)	0.354 (0.201–0.402)	8.39 (1.93–11.4)	8.99 (2.23–12)	8.74 (2.09–11.8)
Togo	Female	76 (69–80.4)	0.123 (0.092–0.145)	0.0659 (0.0486–0.0802)	8.11 (2.25–11.1)	8.23 (2.38–11.2)	8.17 (2.32–11.1)

Supplementary Results Figure S1. Years of Life Lost (YLLs) by scenario, all causes

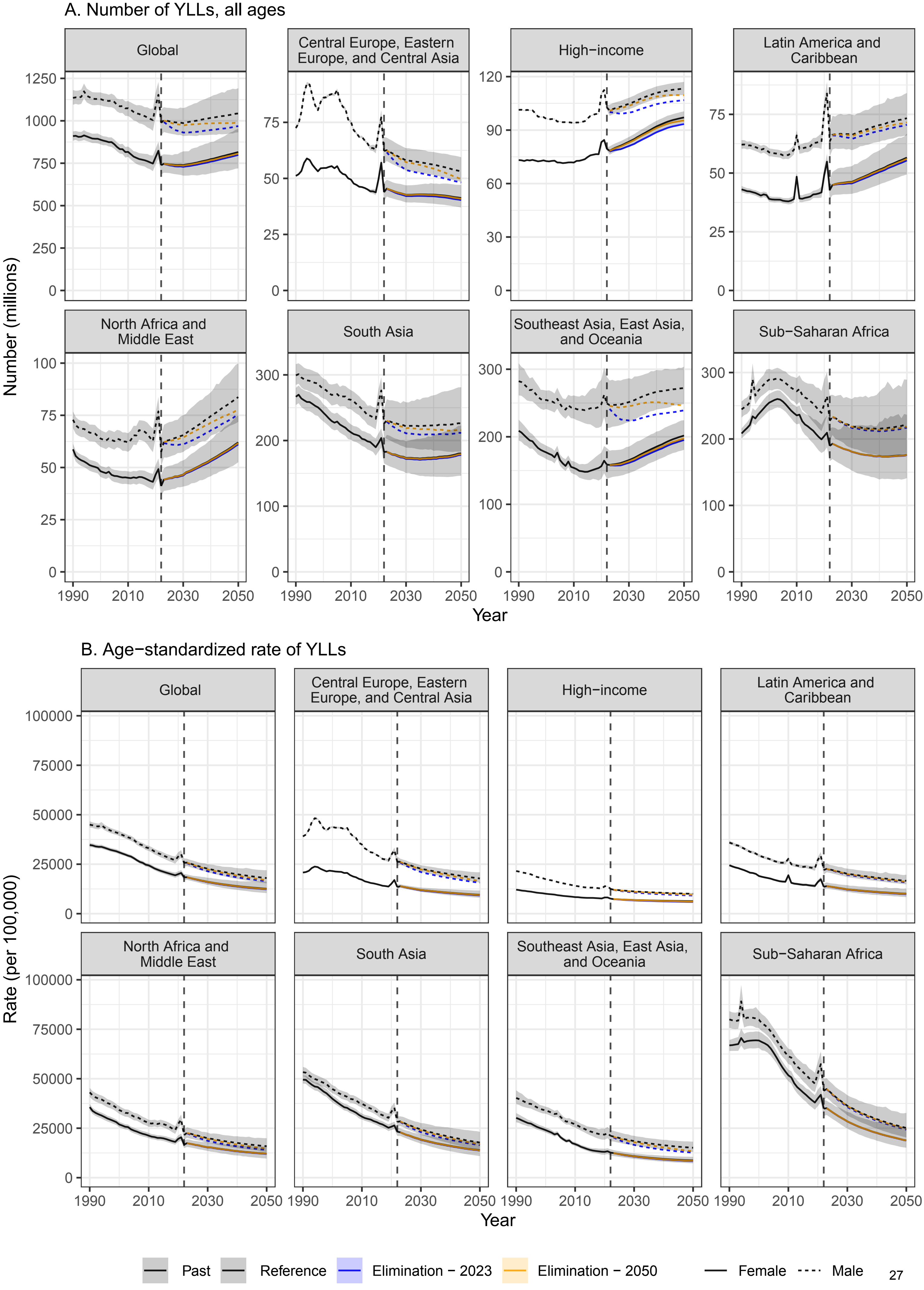

Supplementary Results Figure S2. Years of Life Lost (YLLs) by scenario, IHD

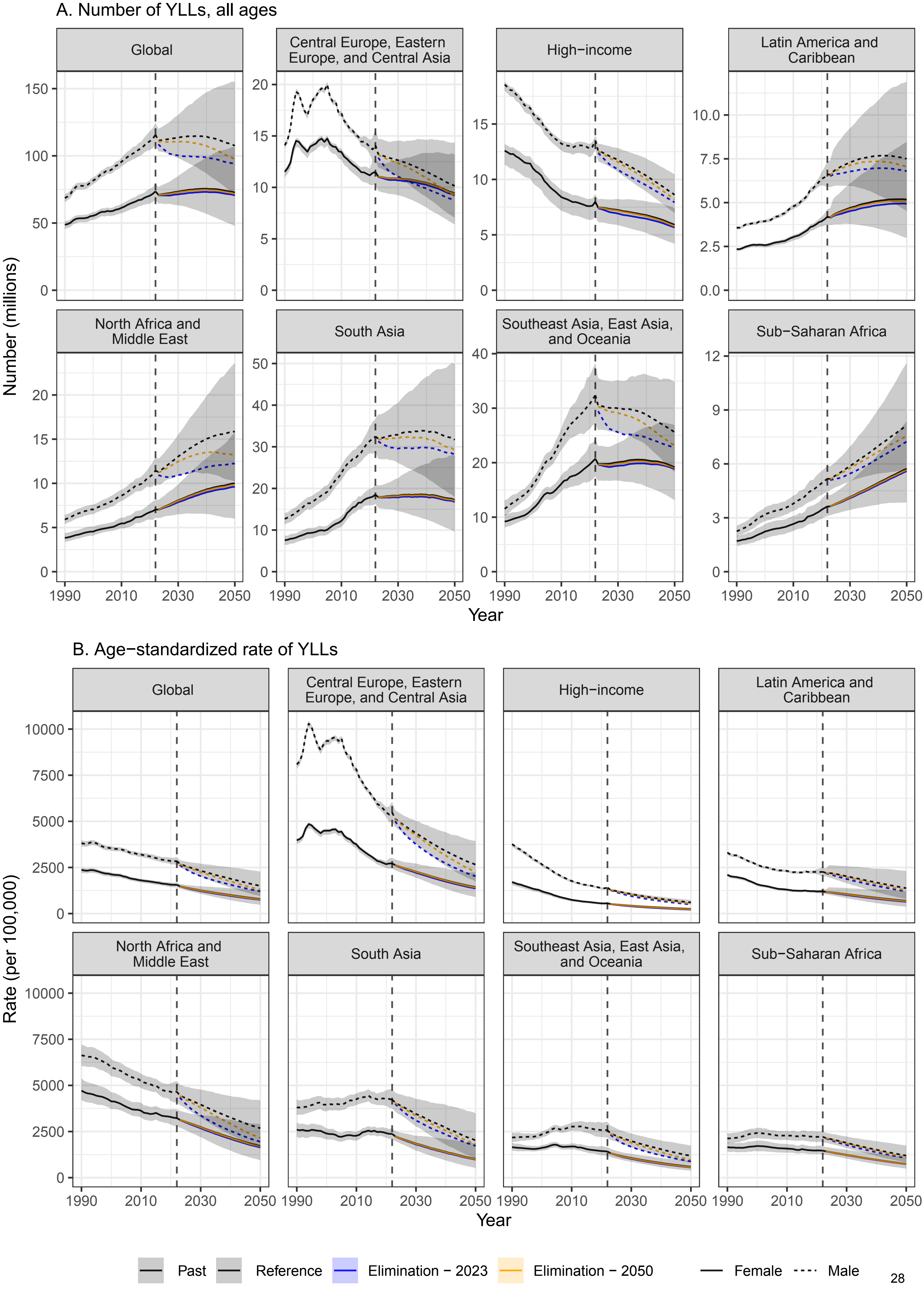

Supplementary Results Figure S3. Years of Life Lost (YLLs) by scenario, lung cancer

A. Number of YLLs, all ages

B. Age−standardized rate of YLLs

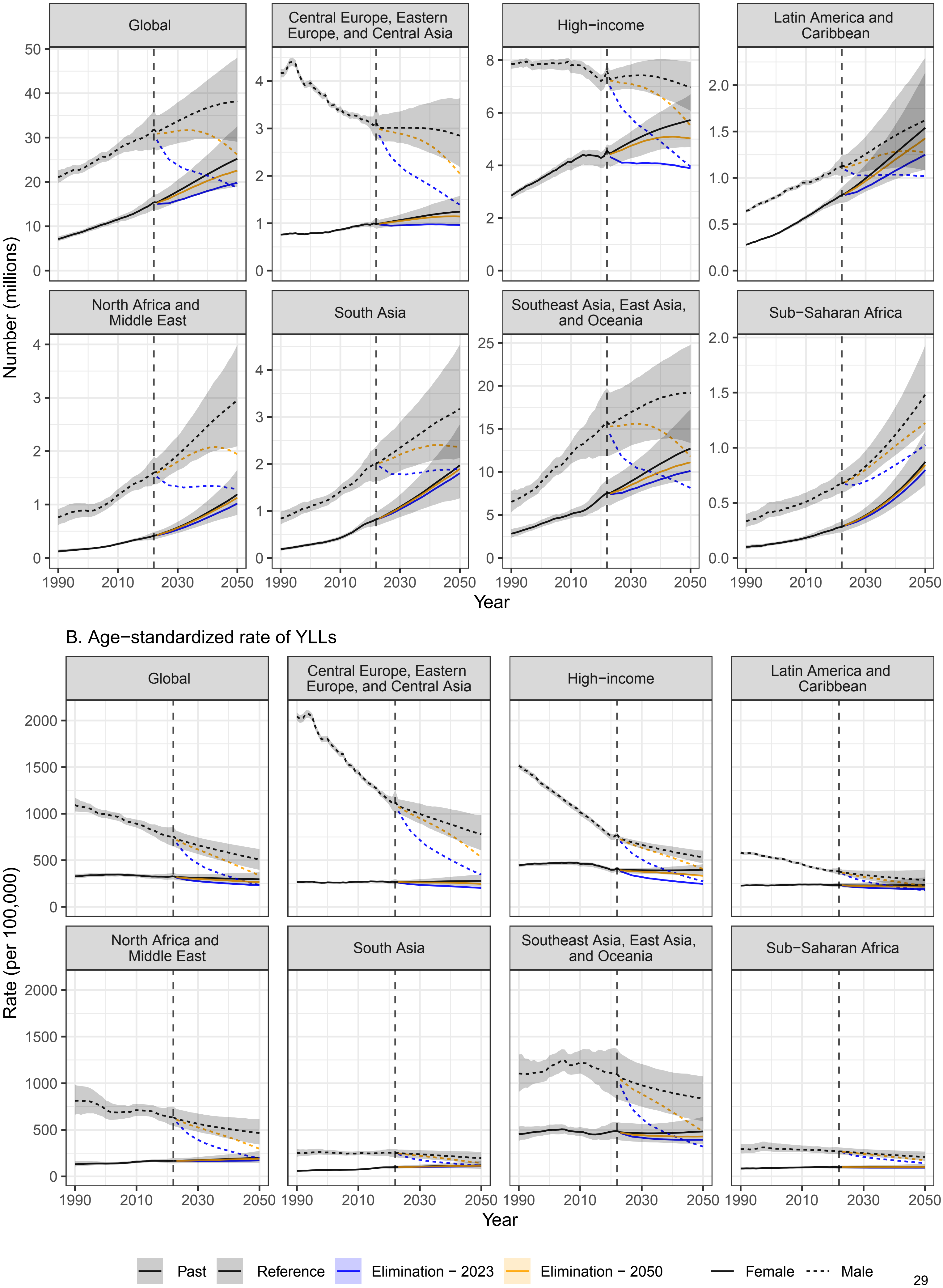

Supplementary Results Figure S4. Years of Life Lost (YLLs) by scenario, COPD

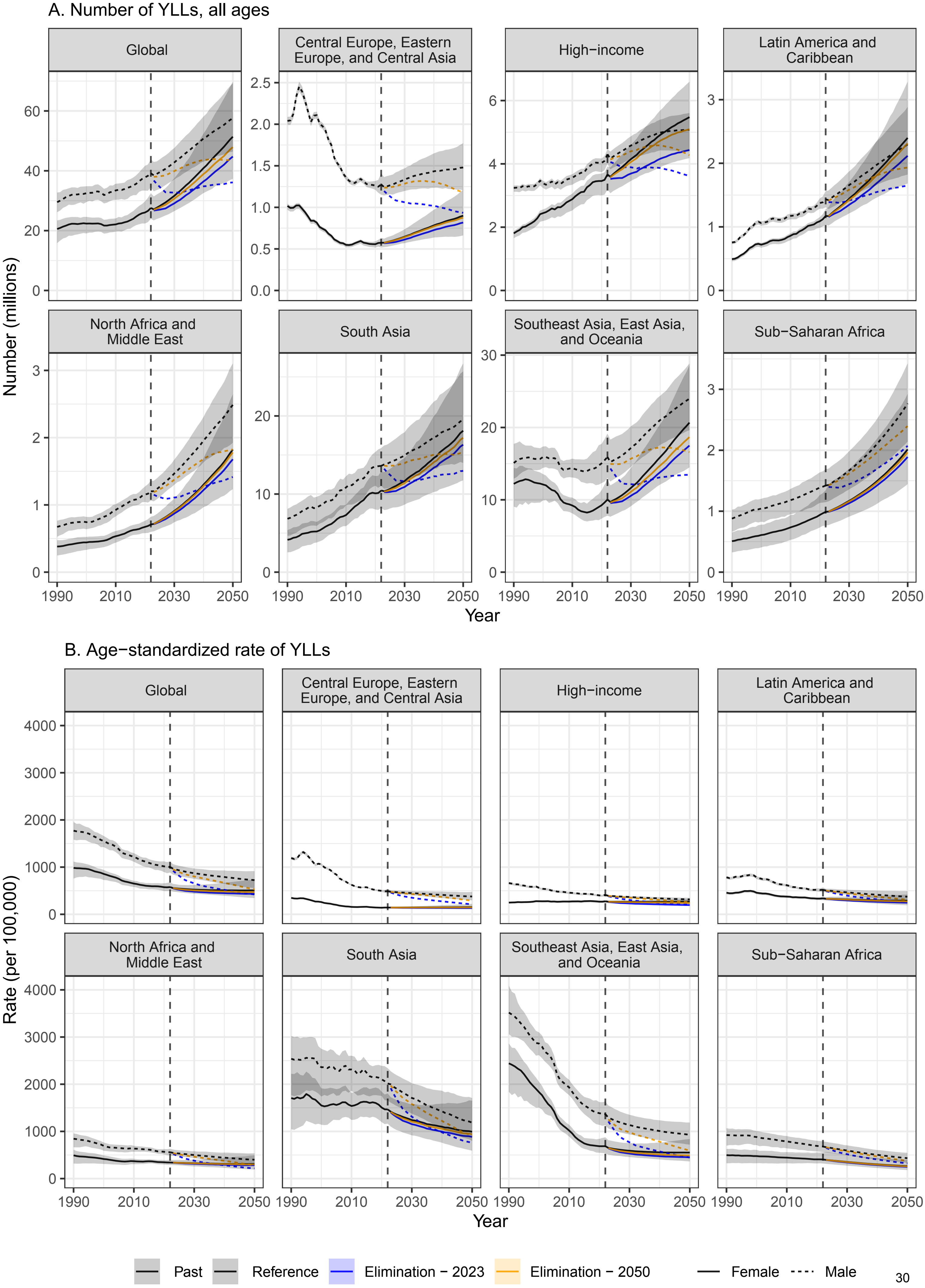

Supplementary Results Figure S5. Life expectancy at birth by super−region, sex, and scenario

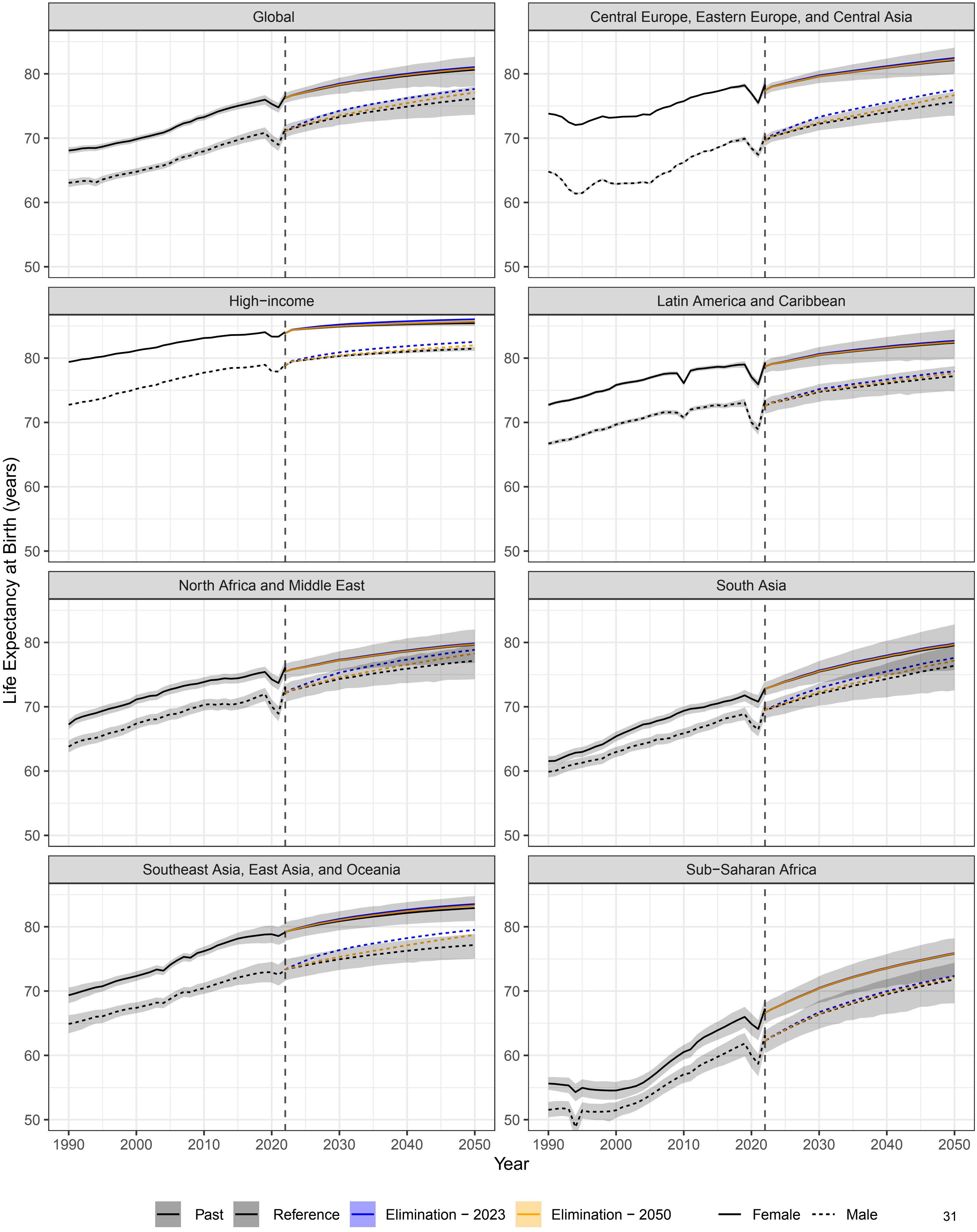

Supplementary Results Figure S6. Global YLLs under Reference scenario

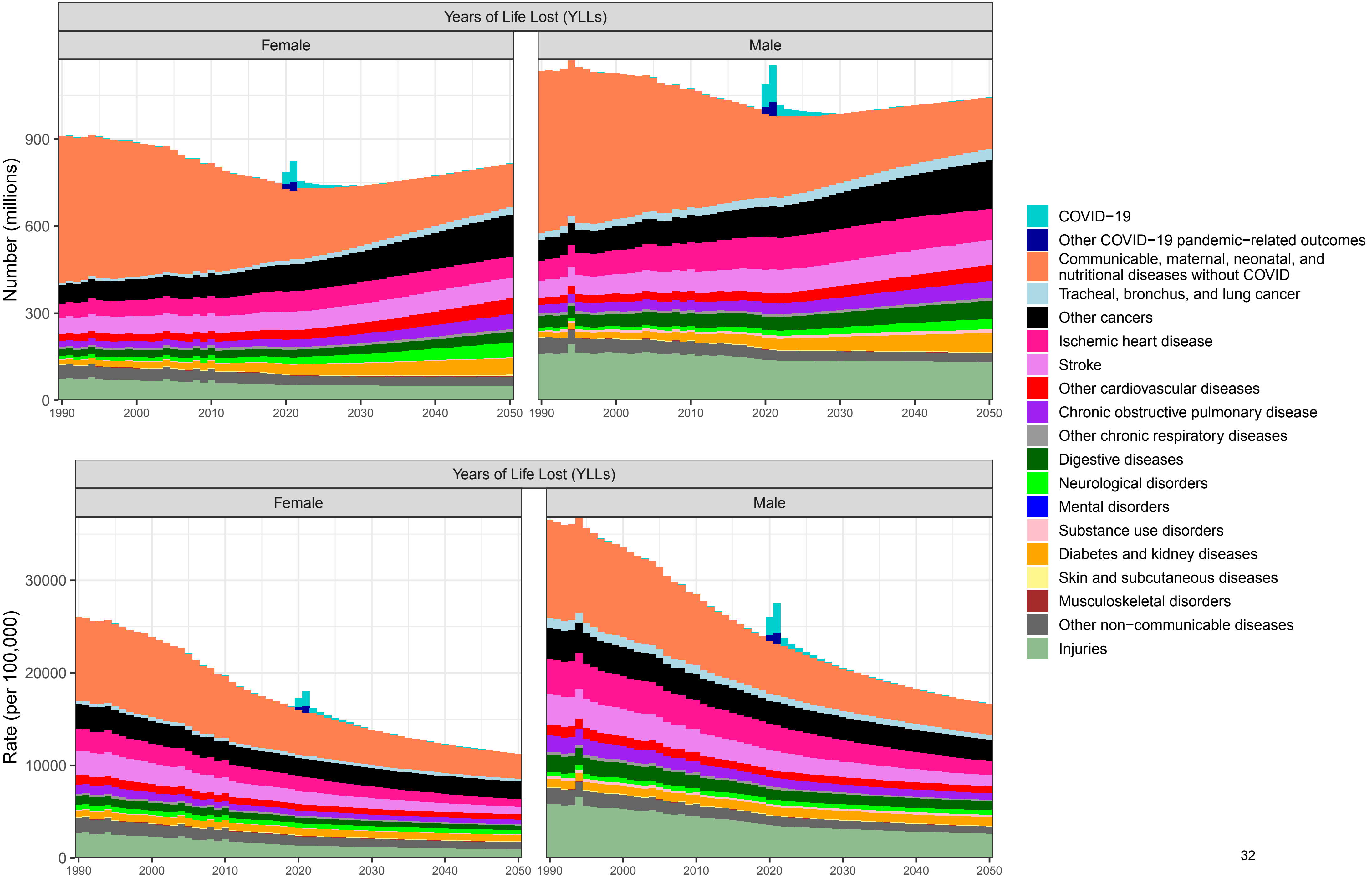

Supplementary Results Figure S7. Difference in cumulative age-standardised rate of YLLs under Elimination-2023 scenario compared to Reference scenario, 2022 to 2050 (per 100,000), Males

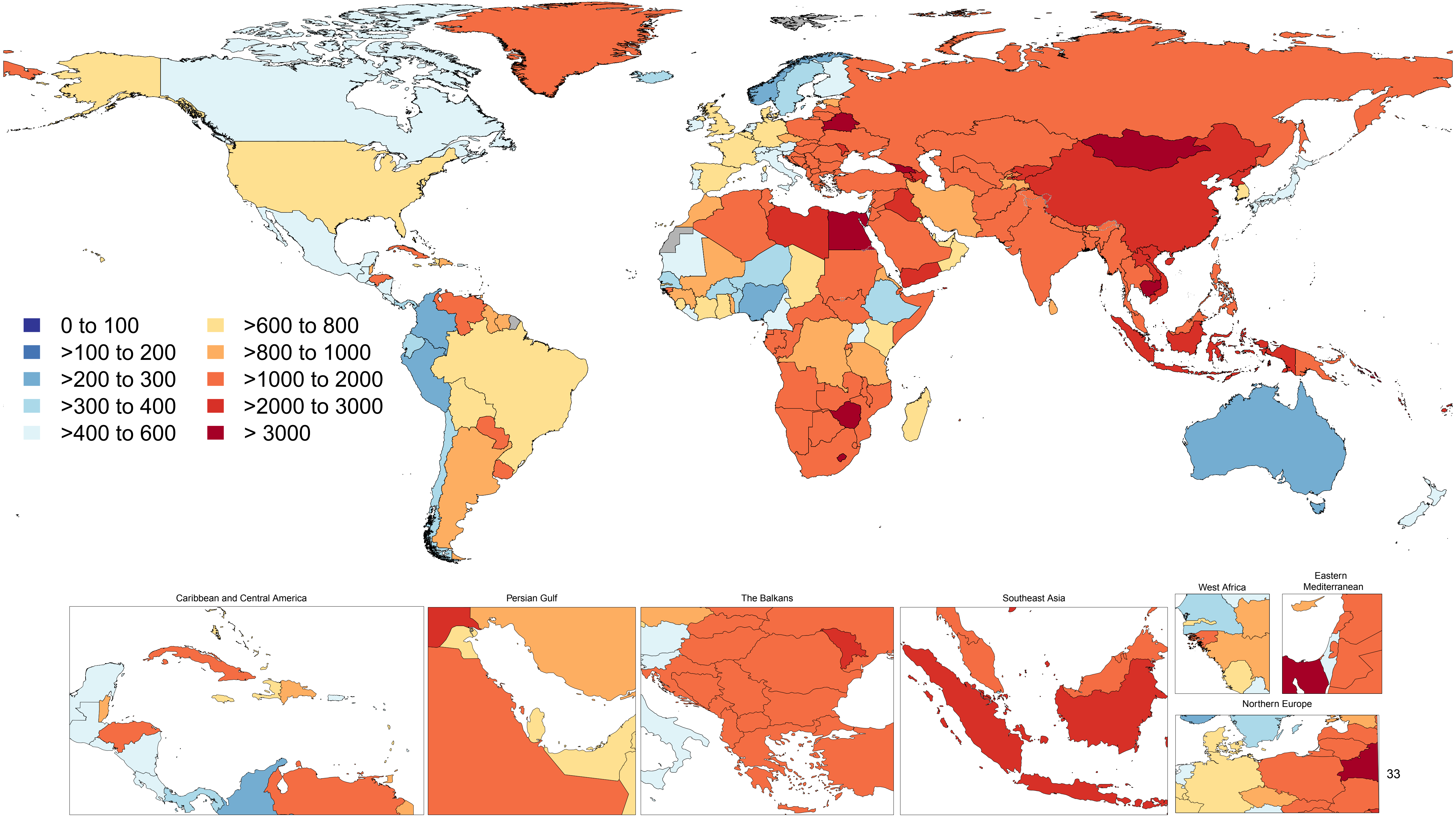

Supplementary Results Figure S8. Difference in cumulative age-standardised rate of YLLs under Elimination-2023 scenario compared to Reference scenario, 2022 to 2050 (per 100,000), Females

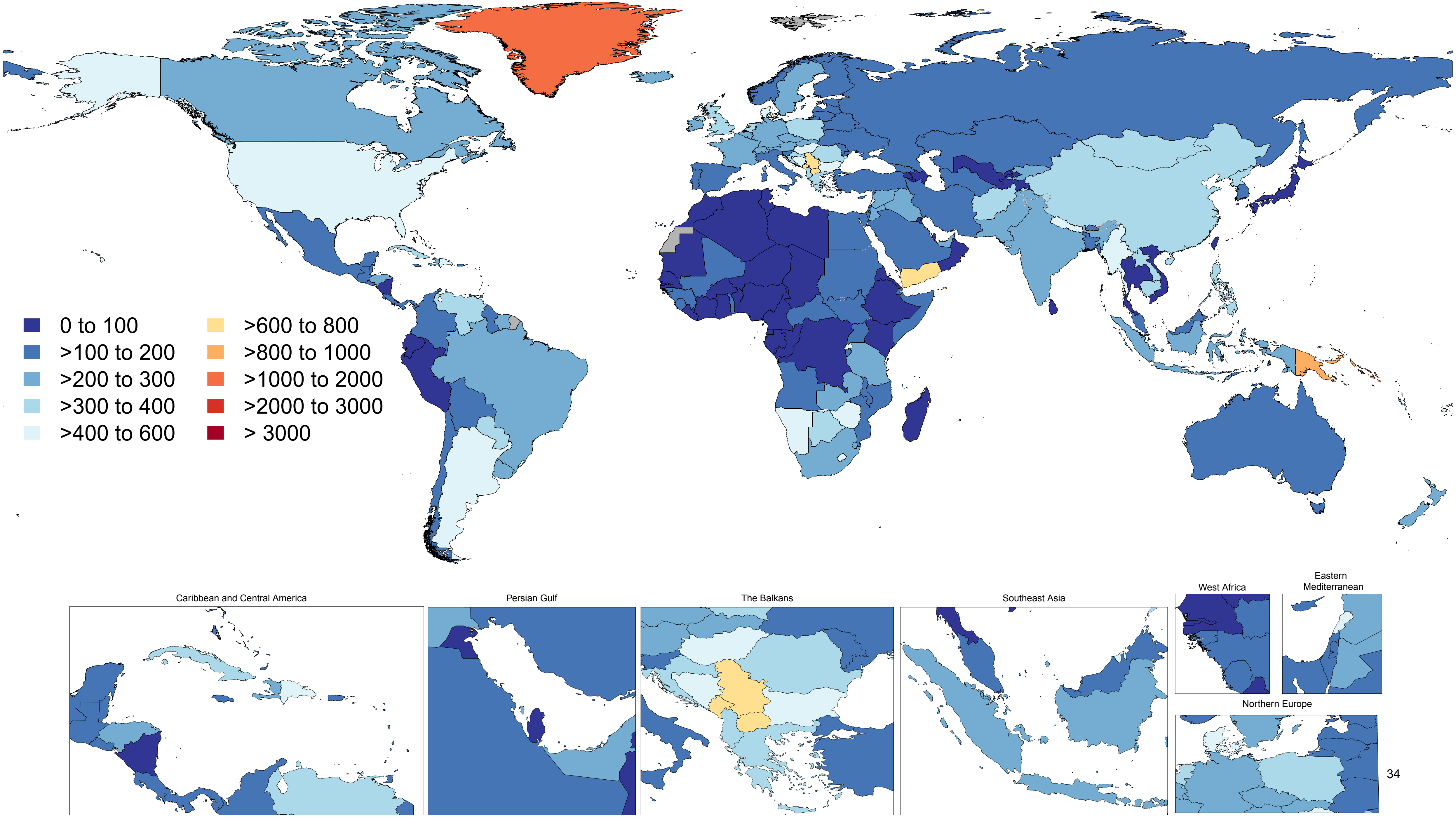

Supplement: Supplementary appendix 2 [file mmc2.pdf]
