# Supplementary material for: Forecasting the effects of smoking prevalence scenarios on years of life lost and life expectancy from 2022 to 2050: a systematic analysis for the Global Burden of Disease Study 2021
Source: Lancet Public Health. 2024 Oct 2;9(10):e729–44. doi: 10.1016/S2468-2667(24)00166-X (PMC11447278; doi:10.1016/S2468-2667(24)00166-X)
Supplement: Supplementary appendix 3 [file mmc3.pdf]

THE LANCET
Public Health

Supplementary appendix 3

This appendix formed part of the original submission and has been peer reviewed. We post it as supplied by the authors.

Supplement to: GBD 2021 Tobacco Forecasting Collaborators. Forecasting the effects of smoking prevalence scenarios on years of life lost and life expectancy from 2022 to 2050: a systematic analysis for the Global Burden of Disease Study 2021. *Lancet Public Health* 2024; **9:** e729–44.

Appendix 3: Authorship appendix to "Forecasting the effects of smoking prevalence scenarios on years of life lost and life expectancy from 2022 to 2050: a systematic analysis for the Global Burden of Disease Study 2021."

This appendix provides further authorship detail for "Forecasting the effects of smoking prevalence scenarios on years of life lost and life expectancy from 2022 to 2050: a systematic analysis for the Global Burden of Disease Study 2021."

Table of Contents

GBD 2021 Smoking Forecasting Collaborators ... 2

Affiliations ... 6

Authors' Contributions ... 31

Managing the overall research enterprise ... 31

Writing the first draft of the manuscript ... 31

Primary responsibility for applying analytical methods to produce estimates ... 32

Primary responsibility for seeking, cataloguing, extracting, or cleaning data; designing or coding figures and tables ... 32

Providing data or critical feedback on data sources ... 32

Developing methods or computational machinery ... 34

Providing critical feedback on methods or results ... 34

Drafting the work or revising it critically for important intellectual content ... 38

Managing the estimation or publications process ... 41

GBD 2021 Smoking Forecasting Collaborators

Dana Bryazka,* Marissa B Reitsma,* Yohannes Habtegiorgis Abate, Abdallah H A Abd Al Magied, Atef Abdelkader, Arash Abdollahi, Meriem Abdoun, Rizwan Suliankatchi Abdulkader, Roberto Ariel Abeldaño Zuñiga, E S Abhilash, Olugbenga Olusola Abiodun, Olumide Abiodun, Richard Gyan Aboagye, Lucas Guimarães Abreu, Dariush Abtahi, Hasan Abualruz, Bilyaminu Abubakar, Niveen ME Abu-Rmeileh, Salahdein Aburuz, Ahmed Abu-Zaid, Mesafint Molla Adane, Akindele Olupelumi Adebiyi, Oyelola A Adegboye, Victor Adekanmbi, Habeeb Omoponle Adewuyi, Qorinah Estiningtyas Sakilah Adnani, Leticia Akua Adzigbli, Siamak Afaghi, Aanuoluwapo Adeyimika Afolabi, Muhammad Sohail Afzal, Saira Afzal, Antonella Agodi, Williams Agyemang-Duah, Bright Opoku Ahinkorah, Austin J Ahlstrom, Aqeel Ahmad, Danish Ahmad, Muayyad M Ahmad, Sajjad Ahmad, Shahzaib Ahmad, Ali Ahmadi, Anisuddin Ahmed, Ayman Ahmed, Haroon Ahmed, Muktar Beshir Ahmed, Safoora Ahmed, Marjan Ajami, Mohammed Ahmed Akkaif, Ema Akter, Salah Al Awaidy, Syed Mahfuz Al Hasan, Yazan Al-Ajlouni, Ziyad Al-Aly, Khurshid Alam, Zufishan Alam, Wafa A Aldhaleei, Abdelazeem M Algammal, Adel Ali Saeed Al-Gheethi, Khalid F Alhabib, Fadwa Naji Alhalaiqa, Mohammed Khaled Al-Hanawi, Abid Ali, Mohammed Usman Ali, Rafat Ali, Syed Shujait Ali, Waad Ali, Sheikh Mohammad Alif, Syed Mohamed Aljunid, François Alla, Peter Allebeck, Wael Almahmeed, Sabah Al-Marwani, Sadeq Al-Maweri, Mahmoud A Alomari, Jaber S Alqahtani, Ahmed Yaseen Alqutaibi, Rajaa M Mohammad Al-Raddadi, Sahel Majed Alrousan, Saqr Alsakarneh, Najim Z Alshahrani, Zaid Altaany, Awais Altaf, Nelson Alvis-Guzman, Mohammad Al-Wardat, Yaser Mohammed Al-Worafi, Hany Aly, Safwat Aly, Mohammad Sharif Ibrahim Alyahya, Karem H Alzoubi, Walid Adnan Al-Zyoud, Reza Amani, Tarek Tawfik Amin, Sohrab Amiri, Hubert Amu, Gianna Gayle Herrera Amul, Ganiyu Adeniyi Amusa, Tanu Anand, Deanna Anderlini, David B Anderson, Jason A Anderson, Catalina Liliana Andrei, Tudorel Andrei, Mohammed Tahir Ansari, Iyadunni Adesola Anuoluwa, Saeid Anvari, Sumadi Lukman Anwar, Anayochukwu Edward Anyasodor, Jalal Arabloo, Elshaimaa A Arafa, Aleksandr Y Aravkin, Demelash Areda, Brhane Berhe Aregawi, Olatunde Aremu, Anton A Artamonov, Akeza Awealom Asgedom, Mohammad Asghari-Jafarabadi, Mubarek Yesse Ashemo, Tahira Ashraf, Thomas Astell-Burt, Seyyed Shamsadin Athari, Prince Atorkey, Alok Atreya, Avinash Aujayeb, Adedapo Wasiu Awotidebe, Getinet Ayano, Setognal Birara Aychiluhm, Sina Azadnajafabad, Ahmed Y Azzam, Giridhara Rathnaiah Babu, Pegah Bahrami Taghanaki, Saeed Bahramian, Ruhai Bai, Shankar M Bakkannavar, Senthilkumar Balakrishnan, Kiran Bam, Maciej Banach, Soham Bandyopadhyay, Mehmet Firat Baran, Martina Barchitta, Mainak Bardhan, Suzanne Lyn Barker-Collo, Amadou Barrow, Hameed Akande Bashiru, Afisu Basiru, Mohammad-Mahdi Bastan, Sanjay Basu, Saurav Basu, Kavita Batra, Mohsen Bayati, Amir Hossein Behnoush, Shelly L Bell, Luis Belo, Alice A Beneke, Derrick A Bennett, Isabela M Bensenor, Azizullah Beran, Amiel Nazer C Bermudez, Habtamu B Beyene, Devidas S Bhagat, Akshaya Srikanth Bhagavathula, Neeraj Bhala, Nikha Bhardwaj, Pankaj Bhardwaj, Sonu Bhaskar, Ajay Nagesh Bhat, Natalia V Bhattacharjee, Priyadarshini Bhattacharjee, Jasvinder Singh Bhatti, Cem Bilgin, Atanu Biswas, Bijit Biswas, Micheal Kofi Boachie, Eyob Ketema Bogale, Berrak Bora Basara, Hamed Borhany, Samuel Adolf Bosoka, Souad Bouaoud, Edward J Boyko, Hermann Brenner, Andre R Brunoni, Raffaele Bugiardini, Norma B Bulamu, Yasser Bustanji, Zahid A Butt, Florentino Luciano Caetano dos Santos, Daniela Calina, Chao Cao, Fan Cao, Angelo Capodici, Rosario Cárdenas, Giulia Carreras, Joao Mauricio Castaldelli-Maia, Maria Sofia Cattaruzza, Arthur Caye, Luca Cegolon, Edina Cenko, Sonia Cerrai, Sandip Chakraborty, Rama Mohan Chandika, Eeshwar K Chandrasekar, Vijay Kumar Chattu, Anis Ahmad Chaudhary, Akhilanand Chaurasia, An-Tian Chen, Guangjin Chen, Haowei Chen, Meng Xuan Chen, Simiao Chen, Kent Jason Go Cheng, Gerald Chi, Fatemeh

Chichagi, Ritesh Chimoriya, Jesus Lorenzo Chirinos-Caceres, Abdulaal Chitheer, Bryan Chong, Chean Lin Chong, Yuen Yu Chong, Hitesh Chopra, Sonali Gajanan Choudhari, Dinh-Toi Chu, Isaac Sunday Chukwu, Sheng-Chia Chung, Muhammad Chutiyami, Joao Conde, Alexandru Corlateanu, Michael H Criqui, Natalia Cruz-Martins, Alanna Gomes da Silva, Omid Dadras, Siyu Dai, Xiaochen Dai, Giovanni Damiani, Lalit Dandona, Rakhi Dandona, Samuel D Darcho, Reza Darvishi Cheshmeh Soltani, Saswati Das, Nihar Ranjan Dash, Kairat Davletov, Aklilu Tamire Debele, Shayom Debopadhaya, Daniel Demant, Hardik Dineshbhai Desai, Devananda Devegowda, Syed Masudur Rahman Dewan, Arkadeep Dhali, Amol S Dhane, Vishal R Dhulipala, Thanh Chi Do, Milad Dodangeh, Phidelia Theresa Doegah, Sushil Dohare, Deepa Dongarwar, Mario D'Oria, Ojas Prakashbhai Doshi, Rajkumar Prakashbhai Doshi, Robert Kokou Dowou, Ashel Chelsea Dsouza, Haneil Larson Dsouza, Viola Savy Dsouza, Bruce B Duncan, Andre Rodrigues Duraes, Arkadiusz Marian Dziedzic, Abdel Rahman E'mar, Alireza Ebrahimi, Negar Ebrahimi, Mohammad Ebrahimi Kalan, David Edvardsson, Kristina Edvardsson, Ferry Efendi, Diyan Ermawan Effendi, Foolad Eghbali, Michael Ekholuenetale, Rabie Adel El Arab, Ibrahim Farahat El Bayoumy, Iman El Sayed, Iffat Elbarazi, Muhammed Elhadi, Waseem El-Huneidi, Mohamed A Elmonem, Gihan ELNahas, Ibrahim Elsohaby, Chadi Eltaha, Mohd Elmagzoub Eltahir, Mehdi Emamverdi, Theophilus I Emeto, Daniel Asfaw Erku, Farshid Etaee, Elochukwu Fortune Ezenwankwo, Natalia Fabin, Adeniyi Francis Fagbamigbe, Omotayo Francis Fagbule, Shahriar Faghani, Ayesha Fahim, Ildar Ravisovich Fakhradiyev, Luca Falzone, Umar Farooque, Ali Fatehizadeh, Zareen Fatima, Nelsensius Klau Fauk, Timur Fazylov, Alireza Feizkhah, Ginenus Fekadu, Xiaoqi Feng, Pietro Ferrara, Nuno Ferreira, Bikila Regassa Feyisa, Filippos T Filippidis, Florian Fischer, Luisa S Flor, Nataliya A Foigt, Celia Fortuna Rodrigues, Matteo Foschi, Sridevi G, Peter Andras Gaal, Muktar A Gadanya, Abhay Motiramji Gaidhane, Márió Gajdács, Silvano Gallus, Aravind P Gandhi, Balasankar Ganesan, Prem Gautam, Rupesh K Gautam, Miglas Welay Gebregergis, Mesfin Gebrehiwot, Teferi Gebru Gebremeskel, Lemma Getacher, Fataneh Ghadirian, Ramy Mohamed Ghazy, Ali Gholamrezanezhad, Mahsa Ghorbani, Sherief Ghozy, Artyom Urievich Gil, Gabriela Fernanda Gil, Elena V Gnedovskaya, Sonu Goel, Salime Goharinezhad, Mohamad Goldust, Mahaveer Golechha, Pouya Goleij, Davide Golinelli, Giuseppe Gorini, Mahdi Gouravani, Ayman Grada, Michal Grivna, Shekhar Grover, Shi-Yang Guan, Mohammed Ibrahim Mohialdeen Gubari, Avirup Guha, Stefano Guicciardi, Snigdha Gulati, Damitha Asanga Gunawardane, Sasidhar Gunturu, Zhifeng Guo, Anish Kumar Gupta, Bhawna Gupta, Ishita Gupta, Mohak Gupta, Rajeev Gupta, Sapna Gupta, Veer Bala Gupta, Vipin Gupta, Vivek Kumar Gupta, Mostafa Hadei, Najah R Hadi, Ali Hajj Ali, Esam S Halboub, Nadia M Hamdy, Samer Hamidi, Ahmad Hammoud, Graeme J Hankey, Arief Hargono, Josep Maria Haro, Ahmed I Hasaballah, Faizul Hasan, Md Kamrul Hasan, Md Saquib Hasnain, Amr Hassan, Ikrama Ibrahim Hassan, Shoaib Hassan, Simon I Hay, Behzad Heibati, Mohammad Heidari, Mehdi Hemmati, Delia Hendrie, Claudiu Herteliu, Demisu Zenbaba Heyi, Kamal Hezam, Yuta Hiraike, Nguyen Quoc Hoan, Ramesh Holla, Nobuyuki Horita, Md Mahbub Hossain, Sahadat Hossain, Hassan Hosseinzadeh, Mihaela Hostiuc, Sorin Hostiuc, Junjie Huang, Ayesha Humayun, Javid Hussain, Bing-Fang Hwang, Segun Emmanuel Ibitoye, Nayu Ikeda, Adalia Ikiroma, Olayinka Stephen Ilesanmi, Irena M Ilic, Milena D Ilic, Mustapha Immurana, Leeberk Raja Inbaraj, Muhammad Iqhrammullah, Lalu Muhammad Irham, Md Rabiul Islam, Sheikh Mohammed Shariful Islam, Farhad Islami, Gaetano Isola, Ramaiah Itumalla, Masao Iwagami, Mahalaxmi Iyer, Vinothini J, Jalil Jaafari, Louis Jacob, Abdollah Jafarzadeh, Khushleen Jaggi, Nader Jahanmehr, Akhil Jain, Nityanand Jain, Ammar Abdulrahman Jairoun, Sanobar Jaka, Mihajlo Jakovljevic, Reza Jalilzadeh Yengejeh, Elham Jamshidi, Manthan Dilipkumar Janodia, Talha Jawaid, Sathish Kumar Jayapal, Shubha Jayaram, Ruwan Duminda Jayasinghe, Rime Jebai, Sun Ha Jee, Bijay Mukesh Jeswani, Heng Jiang, Mohammad Jokar, Jost B Jonas, Tamas Joo, Nitin Joseph, Charity Ehimwenma Joshua, Jacek Jerzy

Jozwiak, Mikk Jürisson, Vaishali K, Ali Kabir, Zubair Kabir, Vidya Kadashetti, Sivesh Kathir Kamarajah, Mona Kanaan, Kehinde Kazeem Kanmodi, Surya Kant, Rami S Kantar, Paschalis Karakasis, Ibraheem M Karaye, Salah Eddin Karimi, Yeganeh Karimi, Arman Karimi Behnagh, Samad Karkhah, Prabin Karki, Faizan Zaffar Kashoo, Srinivasa Vittal Katikireddi, Harkiran Kaur, Navjot Kaur, Sina Kazemian, Tahseen Haider Kazmi, Peter Njenga Keiyoro, Emmanuelle Kesse-Guyot, Yousef Saleh Khader, Himanshu Khajuria, Amirmohammad Khalaji, Alireza Khalilian, Ajmal Khan, Maseer Khan, Mohammad Jobair Khan, Moien AB Khan, Shaghayegh Khanmohammadi, Khaled Khatab, Haitham Khatatbeh, Moawiah Mohammad Khatatbeh, Amir M Khater, Khalid A Kheirallah, Manoj Khokhar, Moein Khormali, Atulya Aman Khosla, Sepehr Khosravi, Kwanghyun Kim, Min Seo Kim, Yun Jin Kim, Adnan Kisa, Ali-Asghar Kolahi, Somayeh Komaki, Shivakumar KM Marulasiddaiah Kondlahalli, Miikka Korja, Oleksii Korzh, Soewarta Kosen, Karel Kostev, Kewal Krishan, Barthelemy Kuate Defo, Mohammed Kuddus, Omar Kujan, Mukhtar Kulimbet, Ashish Kumar, G Anil Kumar, Nithin Kumar, Rakesh Kumar, Vijay Kumar, Amartya Kundu, Satyajit Kundu, Setor K Kunutsor, Om P Kurmi, Dian Kusuma, Frank Kyei-Arthur, Ville Kytö, Carlo La Vecchia, Chandrakant Lahariya, Daphne Teck Ching Lai, Hanpeng Lai, Ratilal Lalloo, Tea Lallukka, Bagher Larijani, Savita Lasrado, Jerrald Lau, Paolo Lauriola, Thao Thi Thu Le, Janet L Leasher, Munjae Lee, Seung Won Lee, Wei-Chen Lee, Yo Han Lee, Elvynna Leong, Temesgen Leka Lerango, An Li, Wei Li, Virendra S Ligade, Stephen S Lim, Jialing Lin, Paulina A Lindstedt, Gang Liu, Erand Llanaj, José Francisco López-Gil, Paulo A Lotufo, Giancarlo Lucchetti, Alessandra Lugo, Jay B Lusk, Hawraz Ibrahim M Amin, Zheng Feei Ma, Monika Machoy, Farzan Madadizadeh, Elham Mahmoudi, Abdelrahman M Makram, Omar M Makram, Kashish Malhotra, Ahmad Azam Malik, Deborah Carvalho Malta, Abdullah A Mamun, Pejman Mansouri, Mohammad Ali Mansournia, Emmanuel Manu, Hamid Reza Marateb, Jose Martinez-Raga, Miquel Martorell, Roy Rillera Marzo, Yasith Mathangasinghe, Elezebeth Mathews, Medha Mathur, Navgeet Mathur, Rita Mattiello, Andrea Maugeri, Martin McKee, Enkeleint A Mechili, Ravi Mehrotra, Tesfahun Mekene Meto, Birye Dessalegn Mekonnen, Hadush Negash Meles, Walter Mendoza, Ritesh G Menezes, Sultan Ayoub Meo, Atte Meretoja, Tuomo J Meretoja, Tomislav Mestrovic, Caine C A Meyers, Irmina Maria Michalek, Ted R Miller, Giuseppe Minervini, Mojgan Mirghafourvand, Erkin M Mirrakhimov, Vinaytosh Mishra, Sanjeev Misra, Prasanna Mithra, Ahmed Ismail Mohamed, Jama Mohamed, Mouhand F H Mohamed, Nouh Saad Mohamed, Ameen Mosa Mohammad, Sakineh Mohammad-Alizadeh-Charandabi, Ibrahim Mohammadzadeh, Hussen Mohammed, Shafiu Mohammed, Syam Mohan, Ali H Mokdad, Hossein Molavi Vardanjani, Sabrina Molinaro, Shaher Momani, Himel Mondal, Ute Mons, AmirAli Moodi Ghalibaf, Maryam Moradi, Rafael Silveira Moreira, Negar Morovatdar, Shane Douglas Morrison, Vincent Mougin, George Duke Mukoro, Francesk Mulita, Erin C Mullany, Malaisamy Muniyandi, Yanjinlkham Munkhsaikhan, Efren Murillo-Zamora, Christopher J L Murray, Woojae Myung, Pirouz Naghavi, Ganesh R Naik, Soroush Najdaghi, Hastyar Hama Rashid Najmuldeen, Luigi Naldi, Gopal Nambi, Vinay Nangia, Jobert Richie Nansseu, Shumaila Nargus, Gustavo G Nascimento, Abdulqadir J Nashwan, Zuhair S Natto, Javaid Nauman, Muhammad Naveed, Nawsherwan, Biswa Prakash Nayak, Vinod C Nayak, Athare Nazri-Panjaki, Sabina Onyinye Nduaguba, Ruxandra Irina Negoi, Reza Nejad Shahrokh Abadi, Seyed Aria Nejadghaderi, Chakib Nejjari, Subas Neupane, Marie Ng, Josephine W Ngunjiri, Duc Hoang Nguyen, Hau Thi Hien Nguyen, Hien Quang Nguyen, Phat Tuan Nguyen, Phuong The Nguyen, Van Thanh Nguyen, Yeshambel T Nigatu, Taxiarchis Konstantinos Nikolouzakis, Ali Nikoobar, Nasrin Nikravangolsefid, Vikram Niranjan, Chukwudi A Nnaji, Lawrence Achilles Nnyanzi, Efaq Ali Noman, Shuhei Nomura, Syed Toukir Ahmed Noor, Mamoona Noreen, Majid Nozari, Fred Nugen, Chimezie Igwegbe Nzoputam, Ogochukwu Janet Nzoputam, Bogdan Oancea, Kehinde O Obamiro, Ismail A Odetokun, Daniel Bogale Odo, Oluwakemi Ololade Odukoya, Michael Safo Oduro, James Odhiambo

Oguta, In-Hwan Oh, Hassan Okati-Aliabad, Sylvester Reuben Okeke, Akinkunmi Paul Okekunle, Osaretin Christabel Okonji, Andrew T Olagunju, Omotola O Olasupo, Matthew Idowu Olatubi, Gláucia Maria Moraes Oliveira, Abdulhakeem Abayomi Olorukooba, Goran Latif Omer, Sok King Ong, Abdulahi Opejin Opejin, Michal Ordak, Verner N Orish, Esteban Ortiz-Prado, Uchechukwu Levi Osuagwu, Stanislav S Otstavnov, Amel Ouyahia, Mahesh Padukudru P A, Alicia Padron-Monedero, Jagadish Rao Padubidri, Anton Pak, Raul Felipe Palma-Alvarez, Hai-Feng Pan, Demosthenes Panagiotakos, Songhomitra Panda-Jonas, Anamika Pandey, Leonidas D Panos, Ioannis Pantazopoulos, Anca Pantea Stoian, Paraskevi Papadopoulou, Shahina Pardhan, Pragyan Paramita Parija, Romil R Parikh, Eun-Kee Park, Seoyeon Park, Nicholas Parsons, Roberto Passera, Jay Patel, Sangram Kishor Patel, Shankargouda Patil, Hridoy Patwary, Shrikant Pawar, Prince Peprah, Gavin Pereira, Arokiasamy Perianayagam, Richard G Pestell, Fanny Emily Petermann-Rocha, Tom Pham, Anil K Philip, Michael R Phillips, Dimitri Poddighe, Roman V Polibin, Ramesh Poluru, Fabio Porru, Akram Pourshams, Jalandhar Pradhan, Pranil Man Singh Pradhan, Manya Prasad, Akila Prashant, Elton Junio Sady Prates, Dimas Ria Angga Pribadi, Bharathi M Purohit, Jagadeesh Puvvula, Ibrahim Qattea, Venkatraman Radhakrishnan, Catalina Raggi, Pankaja Raghav, Fakher Rahim, Afarin Rahimi-Movaghar, Md Mosfequr Rahman, Mosiur Rahman, Muhammad Aziz Rahman, Shayan Rahmani, Mohammad Rahmanian, Nazanin Rahmanian, Vinoth Rajendran, Pushp Lata Rajpoot, Prashant Rajput, Pradhum Ram, Mahmoud Mohammed Ramadan, Majed Ramadan, Kritika Rana, Rishabh Kumar Rana, Chhabi Lal Ranabhat, Sowmya J Rao, Sina Rashedi, Ahmed Mustafa Rashid, Mohammad-Mahdi Rashidi, Ashkan Rasouli-Saravani, Devarajan Rathish, Santosh Kumar Rauniyar, Ilari Rautalin, Nakul Ravikumar, Salman Rawaf, Murali Mohan Rama Krishna Reddy, Elrashdy Moustafa Mohamed Redwan, Negar Rezaei, Mohsen Rezaeian, Abanoub Riad, Monica Rodrigues, Thales Philipe R Rodrigues da Silva, Jefferson Antonio Buendia Rodriguez, Leonardo Roever, Kevin T Root, Gholamreza Roshandel, Allen Guy Ross, Himanshu Sekhar Rout, Bedanta Roy, Nitai Roy, Simanta Roy, Guilherme de Andrade Ruela, Chandan S N, Cameron John Sabet, Siamak Sabour, Kabir P Sadarangani, Basema Ahmad Saddik, Masoumeh Sadeghi, Mohammad Reza Saeb, Umar Saeed, Pooya Saeedi, Sher Zaman Safi, Dominic Sagoe, Fatemeh Saheb Sharif-Askari, Amirhossein Sahebkar, Soumya Swaroop Sahoo, Md Refat Uz Zaman Sajib, Mirza Rizwan Sajid, Luciane B Salaroli, Mohamed A Saleh, Mohammed Z Y Salem, Dauda Salihu, Yoseph Leonardo Samodra, Abdallah M Samy, Juan Sanabria, Milena M Santric-Milicevic, Bruno Piassi Sao Jose, Muhammad Arif Nadeem Saqib, Made Ary Sarasmita, Aswini Saravanan, Babak Saravi, Yaser Sarikhani, Tanmay Sarkar, Gargi Sachin Sarode, Sachin C Sarode, Benn Sartorius, Brijesh Sathian, Anudeep Sathyanarayan, Maheswar Satpathy, Monika Sawhney, Mete Saylan, Benedikt Michael Schaarschmidt, Michael P Schaub, Markus P Schlaich, Maria Inês Schmidt, Art Schuermans, Austin E Schumacher, Siddharthan Selvaraj, Mohammad H Semreen, Subramanian Senthilkumaran, Sadaf G Sepanlou, Yashendra Sethi, Seyed Arsalan Seyedi, Allen Seylani, Mahan Shafie, Arman Shafiee, Ataollah Shahbandi, Samiah Shahid, Hamid R Shahsavari, Moyad Jamal Shahwan, Ahmed Shaikh, Masood Ali Shaikh, Ali S Shalash, Muhammad Aaqib Shamim, Anas Shamsi, Alfiya Shamsutdinova, Mohd Shanawaz, Abhishek Shankar, Mohammed Shannawaz, Medha Sharath, Amin Sharifan, Manoj Sharma, Ujjawal Sharma, Vishal Sharma, Aziz Sheikh, Ali Sheikhy, Mahabalesh Shetty, Pavanchand H Shetty, Premalatha K Shetty, Desalegn Shiferaw, Tariku Shimels, Rahman Shiri, Aminu Shittu, Ivy Shiue, Velizar Shivarov, Seyed Afshin Shorofi, Sunil Shrestha, Emmanuel Edwar Siddig, João Pedro Silva, Abhinav Singh, Baljinder Singh, Harmanjit Singh, Jasvinder A Singh, Paramdeep Singh, Puneetpal Singh, Surjit Singh, Virendra Singh, Freddy Sitas, Amanda E Smith, Matiwos Soboka, Ranjan Solanki, Marco Solmi, Soroush Soraneh, Joan B Soriano, Ireneous N Soyiri, Michael Spartalis, Chandrashekhar T Sreeramareddy, Panagiotis Stachteas, Dan J Stein, Paschalis Steiropoulos, Aleksandar Stevanović, Kurt Straif, Muhammad Suleman, Gerhard

Sulo, Zhong Sun, Vinay Suresh, Chandan Kumar Swain, Lukasz Szarpak, Sree Sudha T Y, Payam Tabaee Damavandi, Ozra Tabatabaei Malazy, Seyed-Amir Tabatabaeizadeh, Celine Tabche, Jyothi Tadakamadla, Santosh Kumar Tadakamadla, Jabeen Taiba, Iman M Talaat, Ashis Talukder, Mircea Tampa, Jacques Lukenze JL Tamuzi, Ker-Kan Tan, Minale Tareke, Ingan Ukur Tarigan, Mojtaba Teimoori, Mohamad-Hani Temsah, Reem Mohamad Hani Temsah, Masayuki Teramoto, Dufera Rikitu Terefa, Pugazhenthan Thangaraju, Kavumpurathu Raman Thankappan, Rekha Thapar, Rasiah Thayakaran, Nikhil Kenny Thomas, Jansje Henny Vera Ticoalu, Krishna Tiwari, Roman Topor-Madry, Marcos Roberto Tovani-Palone, Khaled Trabelsi, An Thien Tran, Ngoc Ha Tran, Thang Huu Tran, Nguyen Tran Minh Duc, Indang Trihandini, Jaya Prasad Tripathy, Thien Tan Tri Tai Truyen, Evangelia Eirini Tsermpini, Abdul Rohim Tualeka, Aniefiok John Udoakang, Arit Udoh, Atta Ullah, Saeed Ullah, Muhammad Umair, Brigid Unim, Bhaskaran Unnikrishnan, Jibrin Sammani Usman, Sanaz Vahdati, Asokan Govindaraj Vaithinathan, Jef Van den Eynde, Constantine Vardavas, Tommi Juhani Vasankari, Siavash Vaziri, Balachandar Vellingiri, Narayanaswamy Venketasubramanian, Madhur Verma, Paul J Villeneuve, Manish Vinayak, Francesco S Violante, Sergey Konstantinovitch Vladimirov, Simona Ruxandra Volovat, Abdul Wadood, Yasir Waheed, Mandaras Tariku Walde, Shu Wang, Yanzhong Wang, Muhammad Waqas, Nuwan Darshana Wickramasinghe, Peter Willeit, Marcin W Wojewodzic, Asrat Arja Wolde, Tewodros Eshete Wonde, Hong Xiao, Suowen Xu, Mukesh Kumar Yadav, Kazumasa Yamagishi, Danting Yang, Lin Yang, Yuichiro Yano, Amir Yarahmadi, Renjulal Yesodharan, Saber Yezli, Xinglin Yi, Arzu Yiğit, Dehui Yin, Dong Keon Yon, Naohiro Yonemoto, Seok-Jun Yoon, Chuanhua Yu, Chun-Wei Yuan, Fathiah Zakham, Mohammed G M Zeariya, Haijun Zhang, Jianrong Zhang, Liqun Zhang, Claire Chenwen Zhong, Shang Cheng Zhou, Bin Zhu, Magdalena Zielińska, Ghazal Zoghi, Sa'ed H Zyoud, Stein Emil Vollset,† and Emmanuela Gakidou.†

* co-lead authors

† co-senior authors

Affiliations

Institute for Health Metrics and Evaluation (D Bryazka BA, M B Reitsma BS, A J Ahlstrom MSc, J A Anderson BS, A Y Aravkin PhD, N V Bhattacharjee PhD, X Dai PhD, Prof L Dandona MD, Prof R Dandona PhD, L S Flor MPH, G F Gil MPH, Prof S I Hay FMedSci, Prof S S Lim PhD, P A Lindstedt MPH, T Mestrovic PhD, Prof A H Mokdad PhD, V Mougin BA, E C Mullany BA, Prof C J L Murray DPhil, M Ng PhD, T Pham BS, C Raggi MS, A E Schumacher PhD, A E Smith MPA, A A Wolde MPH, C Yuan PhD, Prof S E Vollset DrPH, Prof E Gakidou PhD), Department of Applied Mathematics (A J Ahlstrom MSc, A Y Aravkin PhD), Department of Health Metrics Sciences, School of Medicine (A Y Aravkin PhD, X Dai PhD, Prof R Dandona PhD, L S Flor MPH, Prof S I Hay FMedSci, Prof S S Lim PhD, Prof A H Mokdad PhD, Prof C J L Murray DPhil, B Sartorius PhD, Prof S E Vollset DrPH, Prof E Gakidou PhD), School of Medicine (E J Boyko MD), University of Washington, Seattle, WA, USA; Department of Clinical Governance and Quality Improvement (Y H Abate MSc), Aleta Wondo General Hospital, Aleta Wondo, Ethiopia; College of Pharmacy (A H A Abd Al Magied BPharm), Department of Mathematics and Sciences (A Abdelkader PhD), Department of Clinical Sciences (Prof E A Arafa PhD), Department of Education (M Eltahir PhD), Nonlinear Dynamics Research Center (NDRC) (Prof S Momani PhD), Center for Medical and Bio-Allied Health Sciences Research (Prof M J Shahwan PhD, A Shamsi PhD), Ajman University, Ajman, United Arab Emirates; Minimally Invasive Surgery Research Center (A Abdollahi MD, A Kabir MD), Health

Management and Economics Research Center (J Arabloo PhD), School of Medicine (M Bastan MD, M Dodangeh MD), Iran University of Medical Sciences (F Eghbali MD), Preventive Medicine and Public Health Research Center (S Goharinezhad PhD), Endocrine Research Center (A Karimi Behnagh MD), Department of Echocardiography (A Karimi Behnagh MD), Iran University of Medical Sciences, Tehran, Iran (M Moradi MD); Department of Medicine (Prof M Abdoun PhD), University of Setif Algeria, Sétif, Algeria; Department of Health, Sétif, Algeria (Prof M Abdoun PhD); National Institute of Epidemiology (R Abdulkader PhD), Indian Council of Medical Research, Chennai, India; Postgraduate Department (Prof R Abeldaño Zuñiga PhD), University of Sierra Sur, Miahuatlan de Porfirio Diaz, Mexico; Yhteiskuntadatatieteen keskus (Centre for Social Data Science) (Prof R Abeldaño Zuñiga PhD), Department of Public Health (Prof T Lallukka PhD), Department of Virology (F Zakham PhD), University of Helsinki, Helsinki, Finland (T J Meretoja MD); Department of Botany (E S Abhilash PhD), Sree Narayana Guru College Chelannur, Kozhikode, India; Department of Internal Medicine (O O Abiodun FWACP), Federal Medical Centre, Abuja, Nigeria; Department of Community Medicine (Prof O Abiodun MPH), Babcock University, Ilishan-Remo, Nigeria; Department of Family and Community Health (R G Aboagye MPH), Department of Epidemiology and Biostatistics (L A Adzigbli BSc, S A Bosoka MPhil, R K Dowou MPhil), Department of Population and Behavioural Sciences (H Amu PhD, E Manu PhD), Institute of Health Research (P T Doegah PhD, M Immurana PhD), Department of Microbiology and Immunology (Prof V N Orish PhD), University of Health and Allied Sciences, Ho, Ghana; Department of Pediatric Dentistry of the School of Dentistry (Prof L Abreu PhD), School of Nursing (A da Silva PhD), Department of Maternal-Child Nursing and Public Health (Prof D C Malta PhD, E J S Prates BS), Vaccination Research Observatory (T R Rodrigues da Silva PhD), Department of Infectious Diseases and Tropical Medicine (B P Sao Jose PhD), Federal University of Minas Gerais, Belo Horizonte, Brazil; Department of Anesthesiology (D Abtahi MD), Department of Internal Medicine (S Afaghi MD), Department of Epidemiology (A Ahmadi PhD, Prof S Sabour PhD), National Nutrition and Food Technology Research Institute (M Ajami PhD), Internal Medicine Department (H Borhany MD), Psychiatric Nursing and Management Department (F Ghadirian PhD), Department of Health Policy and Management (N Jahanmehr PhD), Safety Promotion and Injury Prevention Research Center (N Jahanmehr PhD), Social Determinants of Health Research Center (A Kolahi MD, A Nikoobar BSc, M Rashidi MD), Skull Base Research Center (I Mohammadzadeh MD), School of Medicine (S Rahmani MD), Student Research Committee (M Rahmanian MD), Department of Immunology (A Rasouli-Saravani PhD), Shahid Beheshti University of Medical Sciences, Tehran, Iran; Department of Nursing (H Abualruz PhD), Al Zaytoonah University of Jordan, Amman, Jordan; Department of Pharmacology and Toxicology (B Abubakar PhD), Department of Veterinary Public Health and Preventive Medicine (A Shittu MSc), Usmanu Danfodiyo University, Sokoto, Sokoto, Nigeria; Nigerian Institute of Medical Research, Lagos, Nigeria (B Abubakar PhD); Institute of Community and Public Health (Prof N M Abu-Rmeileh PhD), Birzeit University, Ramallah, Palestine; Department of Therapeutics (Prof S Aburuz PhD), Institute of Public Health (I Elbarazi DrPH, Prof M Grivna PhD), Family Medicine Department (M A Khan MSc), College of Medicine and Health Sciences (J Nauman PhD), United Arab Emirates University, Al Ain, United Arab Emirates; College of Pharmacy (Prof S Aburuz PhD), Clinical Department (Prof M M Ahmad PhD), University of Jordan, Amman, Jordan; Department of Biochemistry and Molecular Medicine (A Abu-Zaid PhD), College of Pharmacy (R M H Temsah PharmD), Alfaisal University, Riyadh, Saudi Arabia; College of Graduate Health Sciences (A Abu-Zaid PhD), University of Tennessee, Memphis, TN, USA; College of Medicine and Health Sciences (M M Adane PhD), Department of Health Promotion and Behavioural Science (E K Bogale MPH), Department of Psychiatry (M Tareke MSc), Bahir Dar University, Bahir Dar, Ethiopia; Department of Community Medicine (A O Adebiyi MD),

Department of Educational Counselling and Developmental Psychology (H O Adewuyi PhD), Department of Epidemiology and Medical Statistics (A F Fagbamigbe PhD), Department of Periodontology and Community Dentistry (O F Fagbule FWACS), Department of Health Promotion and Education (S Ibitoye PhD), College of Medicine (A P Okekunle PhD), University of Ibadan, Ibadan, Nigeria; Department of Community Medicine (A O Adebiyi MD, O S Ilesanmi PhD), Department of Periodontology and Community Dentistry (O F Fagbule FWACS), University College Hospital, Ibadan, Ibadan, Nigeria; Menzies School of Health Research (Prof O A Adegboye PhD), Charles Darwin University, Darwin, NT, Australia; Department of Obstetrics and Gynecology (V Adekanmbi PhD), Department of Family Medicine (W Lee PhD), University of Texas Medical Branch, Galveston, TX, USA; Department of Educational Psychology (H O Adewuyi PhD), University of Johannesburg, Johannesburg, South Africa; Department of Public Health (Q Adnani PhD), Universitas Padjadjaran (Padjadjaran University), Bandung, Indonesia; Technical Services Directorate (A A Afolabi MPH), MSI Nigeria Reproductive Choices, Abuja, Nigeria; Department of Life Sciences (M S Afzal PhD, Prof M Umair PhD), School of Sciences (M N Saqib PhD), University of Management and Technology, Lahore, Pakistan; Department of Community Medicine (Prof S Afzal PhD), King Edward Memorial Hospital, Lahore, Pakistan; Department of Public Health (Prof S Afzal PhD), Public Health Institute, Lahore, Pakistan; Department of Medical and Surgical Sciences and Advanced Technologies "GF Ingrassia" (Prof A Agodi PhD, M Barchitta PhD, A Maugeri PhD), Department of Biomedical and Biotechnological Sciences (L Falzone PhD), Department of General Surgery and Medical-Surgical Specialties (Prof G Isola PhD), University of Catania, Catania, Italy; Department of Geography and Planning (W Agyemang-Duah PhD), Queen's University, Kingston, ON, Canada; School of Public Health (B O Ahinkorah MPhil, D Demant PhD), School of Nursing and Midwifery (M Chutiyami PhD), School of Life Sciences (G Liu PhD), School of Biomedical Engineering (N Tran MD), University of Technology Sydney, Sydney, NSW, Australia; Department of Medical Biochemistry (A Ahmad PhD), Shaqra University, Shaqra, Saudi Arabia; School of Medicine and Psychology (D Ahmad PhD), Australian National University, Canberra, ACT, Australia; Public Health Foundation of India, Gandhinagar, India (D Ahmad PhD); Department of Health and Biological Sciences (S Ahmad PhD), Abasyn University, Peshawar, Pakistan; Department of Natural Sciences (S Ahmad PhD), Gilbert and Rose-Marie Chagoury School of Medicine (Prof L Roever PhD), Lebanese American University, Beirut, Lebanon; Department of Medical Oncology (S Ahmad MD, A A Khosla MD), Miami Cancer Institute, Miami, FL, USA; Department of Community Medicine and Preventive Health (S Ahmad MD), King Edward Medical University Lahore, Lahore, Pakistan; Department of Epidemiology and Biostatistics (A Ahmadi PhD), Community-Oriented Nursing Midwifery Research Center (M Heidari PhD), Shahrekord University of Medical Sciences, Shahrekord, Iran; Maternal and Child Health Division (A Ahmed MS, E Akter MSc, H Patwary MSc), Department of Maternal and Child Health (S Noor MS), International Centre for Diarrhoeal Disease Research, Bangladesh, Dhaka, Bangladesh; Department of Women's and Children's Health (A Ahmed MS), Uppsala University, Uppsala, Sweden; Institute of Endemic Diseases (A Ahmed MSc), Unit of Basic Medical Sciences (E E Siddig MD), University of Khartoum, Khartoum, Sudan; Swiss Tropical and Public Health Institute (A Ahmed MSc), University of Basel, Basel, Switzerland; Department of Biosciences (H Ahmed PhD), COMSATS Institute of Information Technology, Islamabad, Pakistan; Department of Epidemiology (M B Ahmed PhD, D Shiferaw MPH), Department of Public Health (M Y Ashemo MPH), Institute of Health Science (A I Mohamed MSc), Department of Psychiatry (M Soboka MSc), Jimma University, Jimma, Ethiopia (B Feyisa MPH); College of Medicine and Public Health (M B Ahmed PhD, T G Gebremeskel PhD, G R Naik PhD), Flinders Health and Medical Research Institute (N B Bulamu PhD), Department of Nursing and Health Sciences (S Shorofi PhD), Flinders University, Adelaide,

SA, Australia; Department of Biochemistry (S Ahmed BSc), Jamia Hamdard, Delhi, India; Department of Cardiology (M Akkaif PhD), Fudan University, Shanghai, China; Department of Communicable Diseases (S Al Awaidy MSc), Centre of Studies and Research (S Jayapal PhD), Ministry of Health, Muscat, Oman; Middle East, Eurasia, and Africa Influenza Stakeholders Network, Muscat, Oman (S Al Awaidy MSc); Division of Public Health Sciences (S Al Hasan PhD), Department of Research and Development (Z Al-Aly MD), Department of Surgery (S Azadnajafabad MD), Washington University in St. Louis, St. Louis, MO, USA; School of Medicine (Y Al-Ajlouni MD), New York Medical College, Valhalla, NY, USA; Department of Epidemiology (Y Al-Ajlouni MD), Departments of Psychiatry and Epidemiology (Prof M R Phillips MD), Columbia University, New York, NY, USA; Clinical Epidemiology Center (Z Al-Aly MD), US Department of Veterans Affairs (VA), St Louis, MO, USA; Murdoch Business School (K Alam PhD), Murdoch University, Perth, WA, Australia; School of Health and Environmental Studies (Z Alam PhD, Prof S Hamidi DrPH), Hamdan Bin Mohammed Smart University, Dubai, United Arab Emirates; Division of Gastroenterology and Hepatology (W A Aldhaleei MD, A S Bhagavathula PhD), Mayo Clinic, Jacksonville, FL, USA; Department of Bacteriology, Immunology, and Mycology (Prof A M Algammal PhD), Suez Canal University, Ismailia, Egypt; Global Centre for Environmental Remediation (A A S Al-Gheethi PhD), School of Medicine and Public Health (P Atorkey PhD), University of Newcastle, Newcastle, NSW, Australia; Cooperative Research Centre for Contamination Assessment and Remediation of the Environment, Newcastle, NSW, Australia (A A S Al-Gheethi PhD); Department of Cardiac Sciences (Prof K F Alhabib MD), Department of Physiology (Prof S A Meo PhD), Pediatric Intensive Care Unit (Prof M Temsah MD), King Saud University, Riyadh, Saudi Arabia; College of Nursing (Prof F N Alhalaiqa PhD), College of Dental Medicine (S Al-Maweri PhD), Department of Population Medicine (Prof G Babu PhD), Social and Economic Survey Research Institute (Prof A Perianayagam PhD), Qatar University, Doha, Qatar; Department of Health Services and Hospital Administration (M K Al-Hanawi PhD), Health Economics Research Group (M K Al-Hanawi PhD), Department of Community Medicine (R M M Al-Raddadi MD), Rabigh Faculty of Medicine (Prof A Malik PhD), Department of Dental Public Health (Z S Natto DrPH), King Abdulaziz University, Jeddah, Saudi Arabia; Department of Zoology (A Ali PhD), Department of Biochemistry (Prof A Wadood PhD), Abdul Wali Khan University Mardan, Mardan, Pakistan; Department of Medical Rehabilitation (Physiotherapy) (M U Ali MSc), University of Maiduguri, Maiduguri, Nigeria; Department of Rehabilitation Sciences (M U Ali MSc, M Khan MPH, J S Usman PhD), Hong Kong Polytechnic University, Hong Kong, China; Department of Biosciences (R Ali PhD), Centre For Interdisciplinary Research In Basic Sciences (CIRBSc) (A Shamsi PhD), Jamia Millia Islamia, New Delhi, India; Center for Biotechnology and Microbiology (S S Ali PhD, M Suleman PhD), University of Swat, Swat, Pakistan; Department of Geography (W Ali PhD), Sultan Qaboos University, Muscat, Oman; Institute of Health and Wellbeing (S M Alif PhD), Federation University Australia, Melbourne, VIC, Australia; School of Public Health and Preventive Medicine (S M Alif PhD), School of Public Health and Preventative Medicine (Prof M Asghari-Jafarabadi PhD), School of Psychological Sciences (N Parsons PhD), Monash University, Melbourne, VIC, Australia; Department of Public Health and Community Medicine (Prof S M Aljunid PhD), Division of Community Medicine and Public Health (Prof C T Sreeramareddy MD), International Medical University, Kuala Lumpur, Malaysia; International Centre for Casemix and Clinical Coding (Prof S M Aljunid PhD), National University of Malaysia, Bandar Tun Razak, Malaysia; Bordeaux School of Public Health (Prof F Alla PhD), University of Bordeaux, Bordeaux, France; Department of Global Public Health (Prof P Allebeck MD), Karolinska Institutet, Stockholm, Sweden; Department of Cardiology, Heart, Vascular, and Thoracic Institute (Prof W Almahmeed MD), Cleveland Clinic Abu Dhabi, Abu Dhabi, United Arab Emirates; College of Medicine and Health Sciences Academic

Programs (Prof W Almahmeed MD), Khalifa University, Abu Dhabi, United Arab Emirates; Department of Dentistry (S Al-Marwani MSc), Sana'a University, Sana'a, Yemen; Independent Consultant, Irbid, Jordan (S Al-Marwani MSc); Department of Physical Therapy and Rehabilitation Sciences (Prof M A Alomari PhD), Department of Rehabilitation Sciences and Physical Therapy (Prof M A Alomari PhD), Department of Rehabilitation Sciences (M Al-Wardat PhD), Faculty of Medicine (Prof M S I Alyahya PhD), Department of Clinical Pharmacy (Prof K H Alzoubi PhD), Department of Public Health (Prof Y S Khader PhD, Prof K A Kheirallah PhD), Jordan University of Science and Technology, Irbid, Jordan; Department of Respiratory Care (J S Alqahtani PhD), Prince Sultan Military College of Health Sciences, Dammam, Saudi Arabia; Department of Prosthodontics and Implant Dentistry (A Alqutaibi PhD), Taibah University, Medinah, Saudi Arabia; Department of Prosthodontics and Implant Dentistry (A Alqutaibi PhD), Ibb University, Ibb, Yemen; Macro-Fiscal Policy Department (S M Alrousan PhD), Ministry of Finance, Dubai, United Arab Emirates; Internal Medicine Department (S Alsakarneh MD), University of Missouri, Kansas, MO, USA; Department of Internal Medicine (S Alsakarneh MD), Saint Luke's Mid America Heart Institute, Kansas, MO, USA; Department of Family and Community Medicine (N Z Alshahrani MD), University of Jeddah, Jeddah, Saudi Arabia; Department of Basic Sciences (Z Altaany PhD), Faculty of Nursing (H Khatatbeh PhD), Department of Basic Medical Sciences (Prof M M Khatatbeh PhD), Yarmouk University, Irbid, Jordan; Institute of Molecular Biology and Biotechnology (A Altaf PhD, S Shahid PhD), University Institute of Radiological Sciences and Medical Imaging Technology (T Ashraf MS, Prof Z Fatima PhD), University Institute of Public Health (S Nargus PhD), Research Centre for Health Sciences (RCHS) (S Shahid PhD), The University of Lahore, Lahore, Pakistan; Research Group in Health Economics (Prof N Alvis-Guzman PhD), Universidad de Cartagena (University of Cartagena), Cartagena, Colombia; Research Group in Hospital Management and Health Policies (Prof N Alvis-Guzman PhD), Universidad de la Costa (University of the Coast), Barranquilla, Colombia; Department of Medical Sciences (Prof Y M Al-Worafi PhD), Azal University for Human Development, Sana'a, Yemen; Department of Clinical Sciences (Prof Y M Al-Worafi PhD), University of Science and Technology of Fujairah, Fujairah, United Arab Emirates; Department of Pediatrics (Prof H Aly MD, A E’mar MD), Department of Thoracic Surgery (S Gulati MD), Cleveland Clinic, Cleveland, OH, USA; Department of Pediatric Cardiology (S Aly MD), Boston Children's Hospital, Boston, MA, USA; Department of Pediatrics (S Aly MD), Center for Primary Care (S Basu PhD), Harvard Business School (F Caetano dos Santos PhD), Dana-Farber Cancer Institute (C Cao PhD), Division of Cardiovascular Medicine (G Chi MD), Department of Health Policy and Oral Epidemiology (Z S Natto DrPH), T.H. Chan School of Public Health (P M S Pradhan MD), Division of General Internal Medicine (Prof A Sheikh MD), Harvard University, Boston, MA, USA; Department of Pharmacy Practice and Pharmacotherapeutics (Prof K H Alzoubi PhD), Department of Basic Biomedical Sciences (Prof Y Bustanji PhD), Clinical Sciences Department (N R Dash MD, Prof I M Talaat PhD), Department of Basic Medical Sciences (W El-Huneidi PhD), Department of Clinical Science (Prof M M Ramadan PhD), College of Medicine (Prof B A Saddik PhD, Prof M A Saleh PhD), Sharjah Institute of Medical Sciences (F Saheb Sharif-Askari PhD), College of Pharmacy (Prof M H Semreen PhD), Research Institute of Medical & Health Sciences (Prof M H Semreen PhD), University of Sharjah, Sharjah, United Arab Emirates; Department of Biomedical Engineering (W A Al-Zyoud PhD), German Jordanian University, Amman, Jordan; Interdisciplinary Graduate Program in Human Toxicology (R Amani DVM), University of Iowa, Iowa City, IA, USA; Health Policy Research Center (R Amani DVM, Y Sarikhani PhD), Health Human Resources Research Center (M Bayati PhD), Department of Biostatistics (H Molavi Vardanjani PhD), Non-communicable Disease Research Center (S G Sepanlou MD), Department of Medicine (A Yarahmadi PhD), Shiraz University of Medical Sciences, Shiraz, Iran; Public Health and Community Medicine

Department (Prof T T Amin MD), Department of Neurology (Prof A Hassan MD), National Hepatology and Tropical Medicine Research Institute (A M Khater MD), Cairo University, Cairo, Egypt; Quran and Hadith Research Center (S Amiri PhD), Baqiyatallah University of Medical Sciences, Tehran, Iran; School of Government (G H Amul PhD), Ateneo De Manila University, Quezon City, Philippines; Research for Impact, Singapore, Singapore (G H Amul PhD); Department of Medicine (G A Amusa MD), University of Jos, Jos, Nigeria; Department of Internal Medicine (G A Amusa MD), Jos University Teaching Hospital, Jos, Nigeria; Clinical Studies & Trials Unit (T Anand MD), Indian Council of Medical Research, Delhi, India; Centre for Sensorimotor Performance (D Anderlini MD), School of Dentistry (R Lalloo PhD), Poche Centre for Indigenous Health (Prof A A Mamun PhD), Centre for the Business and Economics of Health (A Pak PhD), Faculty of Medicine (B Sartorius PhD), The University of Queensland, Brisbane, QLD, Australia; Neurology Department (D Anderlini MD), Royal Brisbane and Women's Hospital, Brisbane, QLD, Australia; Faculty of Medicine and Health (D B Anderson PhD), School of Architecture, Design, and Planning (Prof T Astell-Burt PhD), Concord Clinical School (R Chimoriya PhD), Sydney Medical School (S Islam PhD), School of Chemical & Biomolecular Engineering (E A Noman PhD), Menzies Centre for Health Policy (F Sitas PhD), University of Sydney, Sydney, NSW, Australia (S R Okeke PhD); Department of Cardiology (Prof C Andrei PhD), Department of Internal Medicine (M Hostiuc PhD), Department of Legal Medicine and Bioethics (Prof S Hostiuc PhD), Department of Anatomy and Embryology (R I Negoi PhD), Department of Diabetes, Nutrition and Metabolic Diseases (Prof A Pantea Stoian PhD), Department of Dermatology (M Tampa PhD), Carol Davila University of Medicine and Pharmacy, Bucharest, Romania; Department of Statistics and Econometrics (Prof T Andrei PhD, Prof C Herteliu PhD), Bucharest University of Economic Studies, Bucharest, Romania; School of Pharmacy (M Ansari PhD), University of Nottingham Malaysia, Semenyih, Malaysia; Department of Microbiology (I A Anuoluwa PhD), Department of Biosciences and Biotechnology (A J Udoakang PhD), University of Medical Sciences, Ondo, Ondo, Nigeria; Regenerative Medicine, Organ Procurement and Transplantation Multi-disciplinary Center (S Anvari MD), Department of Social Medicine and Epidemiology (A Feizkhah MD), Department of Environmental Health Engineering (J Jaafari PhD), Department of Medical-Surgical Nursing (S Karkhah MSc), Guilan Road Trauma Research Center (N Rahmanian PhD), Guilan University of Medical Sciences, Rasht, Iran; Department of Surgery (S Anwar PhD), Gadjah Mada University, Yogyakarta, Indonesia; Rural Health Research Institute (A E Anyasodor PhD, S B Aychiluhm MPH, Prof A G Ross MD), Charles Sturt University, Orange, NSW, Australia; Department of Pharmacology and Toxicology (Prof E A Arafa PhD), Beni-Suef University, Beni-Suef, Egypt; College of Art and Science (D Areda PhD), Ottawa University, Surprise, AZ, USA; School of Life Sciences (D Areda PhD), Arizona State University, Tempe, AZ, USA; College of Medicine and Health Sciences (B B Aregawi PhD), Department of Midwifery (M W Gebregergis MSc), Department of Medical Laboratory Sciences (H N Meles MSc), Adigrat University, Adigrat, Ethiopia; Department of Public Health (O Aremu PhD), Birmingham City University, Birmingham, UK; Institute for Biomedical Problems (A A Artamonov PhD), Russian Academy of Sciences, Moscow, Russia; Department of Environmental Health (A A Asgedom PhD), Mekelle University, Mekelle, Ethiopia; Cabrini Research (Prof M Asghari-Jafarabadi PhD), Cabrini Health, Malvern, VIC, Australia; Department of Public Health (M Y Ashemo MPH), Wachemo University, Hossana, Ethiopia; Department of Immunology (S Athari PhD), Zanjan University of Medical Sciences, Zanjan, Iran; Australian College of Applied Professions (P Atorkey PhD), Australian College of Applied Professions, Sydney, NSW, Australia; Department of Forensic Medicine (A Atreya MD), Lumbini Medical College, Palpa, Nepal; Northumbria HealthCare NHS Foundation Trust, Newcastle upon Tyne, UK (A Aujayeb MBBS); Department of Physiotherapy (A W Awotidebe PhD, J S Usman PhD), Department of Community Medicine (Prof M A

Gadanya MD), Bayero University Kano, Kano, Nigeria; School of Nursing and Public Health (A W Awotidebe PhD), University of KwaZulu-Natal, Durban, South Africa; School of Indigenous Studies (G Ayano MSc), Centre for Neuromuscular and Neurological Disorders (Prof G J Hankey MD), Dental School (O Kujan PhD), Dobney Hypertension Centre (Prof M P Schlaich MD), The University of Western Australia, Perth, WA, Australia; School of Public Health (G Ayano MSc, D Hendrie PhD, T R Miller PhD), Curtin University, Perth, WA, Australia; Institute of Public Health (S B Aychiluhm MPH), University of Gondar, Gondar, Ethiopia; Leeds Institute of Rheumatic and Musculoskeletal Medicine (S Azadnajafabad MD), School of Dentistry (J Patel BSc), University of Leeds, Leeds, UK; Montefiore-Einstein Cerebrovascular Research Lab (A Azzam MBBCh), Albert Einstein College of Medicine, Bronx, NY, USA; Faculty of Medicine (A Azzam MBBCh), Department of Orthopaedics (A M Makram MD), Department of Cardiology (O M Makram MD), October 6 University, 6th of October City, Egypt; Department of Biostatistics (P Bahrami Taghanaki MD), Orthodontics Department (M Ghorbani DDS), Clinical Research Development Unit (N Morovatdar MD), Research Center (R Nejad Shahrokh Abadi MD), Endodontics Department (P Saeedi DDS), Biotechnology Research Center (Prof A Sahebkar PhD), Department of Medicine (A Yarahmadi PhD), Mashhad University of Medical Sciences, Mashhad, Iran; School of Medicine (S Bahramian MD), Heart Failure Research Center (S Najdaghi MD), Neuroscience Research Center (S Najdaghi MD), Cardiac Rehabilitation Research Center (Prof M Sadeghi MD), Isfahan University of Medical Sciences, Isfahan, Iran; School of Public Affairs (R Bai MD), Nanjing University of Information Science and Technology, Nanjing, China; Department of Forensic Medicine and Toxicology (S M Bakkannavar MD), Kasturba Medical College, Mangalore (R Holla MD, Prof B Unnikrishnan MD), Department of Physiotherapy (Prof V K PhD), Department of Pharmaceutical Regulatory Affairs and Management (V S Ligade PhD), Department of Forensic Medicine (Prof V C Nayak MD), Manipal Academy of Higher Education, Manipal, India (H L Dsouza MD); Division of Biological Sciences (S Balakrishnan PhD), Tamil Nadu State Council for Science and Technology, Chennai, India; Department of Medicine (K Bam MPH), Department of Anatomy and Developmental Biology (Y Mathangasinghe PhD), Monash University, Clayton, VIC, Australia; Department of Hypertension (Prof M Banach PhD), Medical University of Lodz, Lodz, Poland; Polish Mothers' Memorial Hospital Research Institute, Lodz, Poland (Prof M Banach PhD); Nuffield Department of Surgical Sciences (S Bandyopadhyay MPH), Nuffield Department of Population Health (D A Bennett PhD), Nuffield Department of Medicine (B Sartorius PhD), University of Oxford, Oxford, UK; Department of Neurosurgery (S Bandyopadhyay MPH), Faculty of Medicine (R Thayakaran PhD), University of Southampton, Southampton, UK; Vocational School of Technical Sciences (M Baran PhD), Batman University, Batman, Turkiye; Miller School of Medicine (M Bardhan MD), University of Miami, Miami, FL, USA; School of Psychology (Prof S L Barker-Collo PhD), University of Auckland, Auckland, New Zealand; Department of Public and Environmental Health (A Barrow MPH), University of The Gambia, Banjul, The Gambia; Department of Epidemiology (A Barrow MPH, D Yang MPH), College of Medicine (A A Beneke MS, K T Root BS), University of Florida, Gainesville, FL, USA; Department of Animal Sciences (H A Bashiru PhD), Obafemi Awolowo University, Ile-Ife, Nigeria; Department of Veterinary Physiology and Biochemistry (A Basiru PhD), Department of Veterinary Public Health and Preventive Medicine (I A Odetokun PhD), University of Ilorin, Ilorin, Nigeria; Non-communicable Diseases Research Center (M Bastan MD, S Rahmani MD, M Rashidi MD, N Rezaei PhD), School of Medicine (A Behnoush BS, M Gouravani MD, A Khalaji MD, S Khanmohammadi MD, A Shahbandi MD), Department of Scientific Research (F Chichagi MD), Iranian Research Center for HIV/AIDS (IRCHA) (O Dadras PhD), Cardiovascular Department (N Ebrahimi MD), Interdisciplinary Neuroscience Research Program (S Faghani MD), Department of Health in Emergencies and Disasters (M

Hadei PhD), Cardiovascular Diseases Research Institute (Y Karimi MD), Cardiac Primary Prevention Research Center (S Kazemian MD), Department of Cardiac Electrophysiology (S Kazemian MD), Sina Trauma and Surgery Research Center (M Khormali MD), Endocrinology and Metabolism Research Institute (Prof B Larijani MD, N Rezaei PhD, S Seyedi MD, O Tabatabaei Malazy PhD), Department of Cardiology (E Mahmoudi MD, P Mansouri MD), Department of Epidemiology and Biostatistics (M Mansournia PhD), Digestive Diseases Research Institute (Prof A Pourshams MD, S G Sepanlou MD), Iranian National Center for Addiction Studies (Prof A Rahimi-Movaghar MD), Department of Neurology (M Shafie MD), Research Center for Rational Use of Drugs (A Sharifan PharmD), Department of Endocrinology and Metabolism Population Sciences (A Sheikhy MD), Tehran University of Medical Sciences, Tehran, Iran; School of Public Health (S Basu PhD, F T Filippidis PhD, A M Makram MD), Department of Primary Care and Public Health (Prof S Rawaf MD, C Tabche MSc), Imperial College London, London, UK; Department of Academics (S Basu MD), Indian Institute of Public Health, Gurgaon, India; Department of Medical Education (K Batra PhD), Department of Social and Behavioral Health (Prof M Sharma PhD), University of Nevada Las Vegas, Las Vegas, NV, USA; Endocrinology and Metabolism Research Institute (A Khalaji MD), Department of Epidemiology (S Khanmohammadi MD, S Nejadghaderi MD, S Rashedi MD), Department of Epidemiology and Biostatistics (S Khosravi MD), School of Medicine (A Sheikhy MD), Non-Communicable Diseases Research Center (NCDRC), Tehran, Iran (A Behnoush BS); School of the Environment (Prof S L Bell PhD), Department of Internal Medicine (F Etaee MD), Department of Dermatology (M Goldust MD), Department of Psychiatry (W Li PhD), Department of Genetics (S Pawar PhD), Yale University, New Haven, CT, USA; School of Health Policy and Management (Prof S L Bell PhD), Department of Preventive Medicine (Prof Y Lee PhD, Prof S Yoon PhD), Korea University, Seoul, South Korea; Faculty of Pharmacy, Department of Biological Sciences (Prof L Belo PhD), Research Unit on Applied Molecular Biosciences (UCIBIO) (Prof L Belo PhD, J Silva PhD), Institute for Research and Innovation in Health (i3S) (Prof N Cruz-Martins PhD), Associate Laboratory Institute for Health and Bioeconomy (i4HB) (Prof C Fortuna Rodrigues PhD), Faculty of Engineering (Prof C Fortuna Rodrigues PhD), University of Porto, Porto, Portugal; Department of Internal Medicine (I M Bensenor PhD, Prof A R Brunoni PhD), Department of Psychiatry (Prof A R Brunoni PhD, Prof J Castaldelli-Maia PhD, A Caye PhD), Department of Medicine (Prof P A Lotufo DrPH), University of São Paulo, São Paulo, Brazil; School of Medicine (A Beran MD), Indiana University, Indianapolis, IN, USA; Department of Epidemiology and Biostatistics (A C Bermudez MD), University of the Philippines Manila, Manila, Philippines; Department of Epidemiology (A C Bermudez MD), Department of Internal Medicine (M F H Mohamed MSc), Brown University, Providence, RI, USA; Metabolomics Laboratory (H B Beyene PhD), Hypertension and Kidney Disease Laboratory (Prof M P Schlaich MD), Baker Heart and Diabetes Institute, Melbourne, VIC, Australia; Department of Microbiology (H B Beyene PhD), Addis Ababa University, Addis Ababa, Ethiopia; Department of Forensic Chemistry (D S Bhagat PhD), Government Institute of Forensic Science, Aurangabad, Aurangabad, India; Department of Public Health (A S Bhagavathula PhD), North Dakota State University, Fargo, ND, USA; Institute of Applied Health Research (N Bhala PhD), University of Nottingham, Nottingham, UK; Institute of Applied Health Research (N Bhala PhD, K Malhotra MBBS, R Thayakaran PhD), Academic Department of Surgery (S K Kamarajah MD), University of Birmingham, Birmingham, UK; Department of Anatomy (N Bhardwaj MD), Department of Community Medicine and Family Medicine (Prof P Bhardwaj MD, Prof P Raghav MD), School of Public Health (Prof P Bhardwaj MD), Department of Biochemistry (M Khokhar PhD), Department of Surgical Oncology (Prof S Misra MCh), Department of Pharmacology and Research (A Saravanan MD), Department of Pharmacology (M Shamim MBBS, S Singh MD, K Tiwari MBBS), All India Institute of Medical Sciences, Jodhpur, India;

Global Health Neurology Lab (S Bhaskar MD), NSW Brain Clot Bank, Sydney, NSW, Australia; Division of Cerebrovascular Medicine and Neurology (S Bhaskar MD), National Cerebral and Cardiovascular Center, Suita, Japan; Department of General Medicine (A N Bhat MD), Department of Community Medicine (N Joseph MD, N Kumar MD, P Mithra MD, R Thapar MD), Department of Forensic Medicine and Toxicology (Prof J Padubidri MD, P H Shetty MD), Department of Internal Medicine (M M R Reddy MD), Manipal College of Dental Sciences Mangalore (Prof P K Shetty MDS), Manipal Academy of Higher Education, Mangalore, India; Newcastle University, Newcastle upon Tyne, UK (P Bhattacharjee MD); Department of Clinical Medicine (P Bhattacharjee MD), Cambridge University Hospitals NHS Foundation Trust, Cambridge, UK; Department of Human Genetics and Molecular Medicine (Prof J Bhatti PhD, U Sharma PhD), Department of Microbiology (M Iyer PhD, M Yadav PhD), Department of Biochemistry (B Singh PhD), Human Genetics and OMICS, Department of Zoology (B Vellingiri PhD), Central University of Punjab, Bathinda, India; Department of Radiology (C Bilgin MD), Neurovascular Research Laboratory (C Bilgin MD), Mayo Clinic College of Medicine, Rochester, MN, USA; Department of Neurology (Prof A Biswas DM), Institute of Post-Graduate Medical Education and Research and Seth Sukhlal Karnani Memorial Hospital, Kolkata, India; Department of Community Medicine and Family Medicine (B Biswas MD), Department of Physiology (H Mondal MD), Department of Pharmacology (S T Y MD), All India Institute of Medical Sciences, Deoghar, India; SAMRC/Wits Centre for Health Economics and Decision Science - PRICELESS SA (M K Boachie PhD), SAMRC Centre for Health Economics and Decision Science (M K Boachie PhD), University of the Witwatersrand, Johannesburg, South Africa; General Directorate of Health Information Systems (B Bora Basara PhD), Ministry of Health, Ankara, Turkiye; Disease Surveillance Department (S A Bosoka MPhil), Ghana Health Service, Ho, Ghana; Department of Medicine (Prof S Bouaoud DrPH), Faculty of Medicine (Prof A Ouyahia PhD), University Ferhat Abbas of Setif, Setif, Algeria; Department of Epidemiology and Preventive Medicine (Prof S Bouaoud DrPH), University Hospital Saadna Abdenour, Setif, Algeria; General Medicine Service (E J Boyko MD), Department of Veterans Affairs, Seattle, WA, USA; Division of Clinical Epidemiology and Aging Research (Prof H Brenner MD), Faculty of Medicine and University Hospital Cologne (Prof U Mons PhD), German Cancer Research Center, Heidelberg, Germany; Department of Medical and Surgical Sciences (Prof R Bugiardini MD, E Cenko MD, Prof F S Violante MD), Department of Biomedical and Neuromotor Sciences (S Guicciardi MD), University of Bologna, Bologna, Italy; School of Pharmacy (Prof Y Bustanji PhD), Department of Mathematics (Prof S Momani PhD), The University of Jordan, Amman, Jordan; School of Public Health Sciences (Z A Butt PhD), University of Waterloo, Waterloo, ON, Canada; Al Shifa School of Public Health (Z A Butt PhD), Al Shifa Trust Eye Hospital, Rawalpindi, Pakistan; Department of Clinical Pharmacy (Prof D Calina PhD), University of Medicine and Pharmacy of Craiova, Romania, Craiova, Romania; Department of Ophthalmology (F Cao MD), Beijing Institute of Ophthalmology, Beijing, China; Department of Health Management (Direzione Sanitaria) (A Capodici MD), IRCCS Istituto Ortopedico Rizzoli, Bologna, Italy; Interdisciplinary Research Center for Health Science (A Capodici MD), Sant'Anna School of Advanced Studies, Pisa, Italy; Department of Health Care (Prof R Cárdenas DSc), Metropolitan Autonomous University, Mexico City, Mexico; Oncological Network, Prevention and Research Institute (G Gorini MD), Institute for Cancer Research, Prevention and Clinical Network, Florence, Italy (G Carreras PhD); Department of Public Health and Infectious Diseases (M S Cattaruzza PhD), La Sapienza University, Rome, Italy; Department of Psychiatry (A Caye PhD), Postgraduate Program in Epidemiology (Prof B B Duncan MD, Prof M I Schmidt MD), Department of Social Medicine (R Mattiello PhD), Federal University of Rio Grande do Sul, Porto Alegre, Brazil; Department of Medical, Surgical, and Health Sciences (L Cegolon PhD, Prof M D'Oria MD), University of Trieste, Trieste, Italy; Public Health Unit (L Cegolon PhD),

University Health Agency Giuliano-Isontina (ASUGI), Trieste, Italy; Institute of Clinical Physiology (S Cerrai MSc), Italian National Council of Research, Pisa, Italy; State Disease Investigation Laboratory (S Chakraborty MVSc), Animal Resources Development Department, Agartala, India; Department of Clinical Nutrition (R M Chandika PhD), Department of Epidemiology (S Dohare MD, M Khan MD), Department of Maxillofacial Surgery and Diagnostic Sciences (E S Halboub PhD), Substance Abuse and Toxicology Research Center (S Mohan PhD), Department of Public Health (P Rajpoot PhD), College of Nursing and Health Sciences (M Shanawaz MD), Jazan University, Jazan, Saudi Arabia; Department of Anesthesiology and Perioperative Medicine (E K Chandrasekar MD), School of Medicine (Prof S Xu PhD), University of Rochester, Rochester, NY, USA; Temerty Faculty of Medicine (V Chattu MD), University of Toronto, Toronto, ON, Canada; Department of Community Medicine (V Chattu MD), Datta Meghe Institute of Medical Sciences, Sawangi, India; Department of Biology (A A Chaudhary PhD), Al-Imam Mohammad Ibn Saud Islamic University, Riyadh, Saudi Arabia; Oral Medicine and Radiology (A Chaurasia MD), Department of Respiratory Medicine (Prof S Kant MD), Department of Neurology (V Suresh MBBS), King George's Medical University, Lucknow, India; Fuwai Hospital (A Chen PhD), Chinese Academy of Medical Sciences & Peking Union Medical College, Beijing, China; Department of Computer Science (A Chen PhD), University of Texas at Austin, Austin, TX, USA; Department of Stomatology (G Chen DMD), Huazhong University of Science and Technology, Wuhan, China; Hubei Province Key Laboratory of Oral and Maxillofacial Development and Regeneration, Wuhan, China (G Chen DMD); Clinical Research Center (H Chen PhD), Zhujiang Hospital of Southern Medical University, Guangzhou, China; University of Michigan, Ann Arbor, MI, USA (M Chen DDS); Heidelberg Institute of Global Health (HIGH) (S Chen DSc, Prof S Mohammed PhD), Department of Ophthalmology (S Panda-Jonas MD), Heidelberg University, Heidelberg, Germany; Center for Healthy Aging (K G Cheng PhD), Pennsylvania State University, University Park, PA, USA; Social Sciences Division (K G Cheng PhD), Syracuse University, Syracuse, NY, USA; Concord Institute of Academic Surgery (R Chimoriya PhD), Sydney Local Health District, Sydney, NSW, Australia; Department of Public Health, Administration, and Social Sciences (J L Chirinos-Caceres DrPH), Cayetano Heredia University, Lima, Peru; Iraq Field Epidemiology Training Program (I-FETP) (A Chitheer MD), Ministry of Health, Baghdad, Iraq; Department of Medicine (B Chong MBBS), Department of Surgery (J Lau MPH, K Tan PhD), Yong Loo Lin School of Medicine (M Ng PhD, Prof N Venketasubramanian MSc), National University of Singapore, Singapore, Singapore; RIPAS Hospital (C Chong MD), University of Brunei Darussalam, BSB, Brunei; The Nethersole School of Nursing (Y Chong PhD), Department of Paediatrics (S Dai PhD), Faculty of Medicine (J Huang MD), Jockey Club School of Public Health and Primary Care (C Zhong PhD), The Chinese University of Hong Kong, Hong Kong, China; Centre for Research Impact & Outcome (H Chopra PhD), Chitkara University, Rajpura, India; Department of Community Medicine (Prof S G Choudhari MD), Jawaharlal Nehru Medical College, Wardha, India; Center for Biomedicine and Community Health (D Chu PhD), International School, Vietnam National University Hanoi (VNUIS), Hanoi, Vietnam; Department of Paediatric Surgery (I S Chukwu BMedSc), Federal Medical Centre, Umuahia, Nigeria; Department of Health Informatics (S Chung PhD), Department of Behavioural Science and Health (S Hossain MS), University College London, London, UK; Health Data Research UK, London, UK (S Chung PhD); Nova Medical School (Prof J Conde PhD), Nova University of Lisbon, Lisbon, Portugal; Department of Respiratory Medicine and Allergology (Prof A Corlateanu PhD), Nicolae Testemitanu State University of Medicine and Pharmacy, Chisinau, Moldova; Department of Family Medicine and Public Health (Prof M H Criqui MD), University of California San Diego, La Jolla, CA, USA; Department of Diagnostic and Therapeutic Technologies (Prof N Cruz-Martins PhD), Cooperativa de Ensino Superior Politécnico e Universitário (Polytechnic and University Higher

Education Cooperative), Vila Nova de Famalicão, Portugal; Department of Global Public Health and Primary Care (O Dadras PhD), Center for International Health (CIH) (S Hassan MPhil), Bergen Center for Ethics and Priority Setting (BCEPS) (S Hassan MPhil), Department of Psychosocial Science (Prof D Sagoe PhD), University of Bergen, Bergen, Norway; School of Clinical Medicine (S Dai PhD), Hangzhou Normal University, Hangzhou, China; IRCCS Istituto Ortopedico Galeazzi (Galeazzi Orthopedic Institute IRCCS) (G Damiani MD), Department of Clinical Sciences and Community Health (Prof C La Vecchia MD), University of Milan, Milan, Italy; Department of Dermatology (G Damiani MD, A Grada MD), Harrington Heart and Vascular Institute (A Guha MD), Department of Neonatology (I Qattea MD), Department of Nutrition and Preventive Medicine (Prof J Sanabria MD), Case Western Reserve University, Cleveland, OH, USA; Public Health Foundation of India, Gurugram, India (Prof L Dandona MD, Prof R Dandona PhD, G Kumar PhD, A Pandey PhD); Non-communicable Diseases Division (NCD) (A K Gupta PharmD), Indian Council of Medical Research, New Delhi, India (Prof L Dandona MD); Department of Public Health (S D Darcho MPH), Department of Health Policy and Management (A T Debele MSc), Department of Psychiatry (M T Walde MSc), Haramaya University, Harar, Ethiopia; Environmental Health (R Darvishi Cheshmeh Soltani PhD), Arak University of Medical Sciences, Arak, Iran; Department of Biochemistry (S Das MD), Ministry of Health and Welfare, New Delhi, India; Health Research Institute (Prof K Davletov PhD), Director of the Scientific and Technological Park (I R Fakhradiyev PhD), Laboratory of Experimental Medicine (T Fazylov MD), Atchabarov Scientific-Research Institute of Fundamental and Applied Medicine (M Kulimbet MSc), Science Department (A Shamsutdinova MD), Kazakh National Medical University, Almaty, Kazakhstan; Medical College (S Debopadhaya BS), Albany Medical College, Albany, NY, USA; School of Public Health and Social Work (D Demant PhD), Queensland University of Technology, Brisbane, QLD, Australia; Department of Research (H D Desai MD), Gujarat Adani Institute of Medical Sciences and G.K. General Hospital, Bhuj, India; JSS Medical College Department of Biochemistry (D Devegowda PhD), Department of Biochemistry (Prof A Prashant PhD), Jagadguru Sri Shivarathreeswara University, Mysuru, India; Department of Pharmacy (S Dewan PhD), United International University, Dhaka, Bangladesh; Pharmacology Division (S Dewan PhD), Center for Life Sciences Research Bangladesh, Dhaka, Bangladesh; Sheffield Teaching Hospitals NHS Foundation Trust, Sheffield, UK (A Dhali MBBS); Research and Development Cell (A S Dhane MBA), Dr. D. Y. Patil University, Pune, India; The Zena and Michael A. Wiener Cardiovascular Institute (V R Dhulipala MD), Department of Psychiatry (S Gunturu MD), Department of Cardiology (M Vinayak MD), Icahn School of Medicine at Mount Sinai, New York, NY, USA (A Shaikh MD); Department of Medicine (T C Do MD, A T Tran MD), Pham Ngoc Thach University of Medicine, Ho Chi Minh City, Vietnam; Health Science Center (D Dongarwar MS), University of Texas, Houston, TX, USA; Cardio-Thoraco-Vascular Department (Prof M D'Oria MD), Azienda Sanitaria Universitaria Giuliano Isontina, Trieste, Italy; Independent Consultant, South Plainfield, NJ, USA (O P Doshi MSc); Department of Cardiology (R P Doshi MD), Hackettstown Medical Center, Hackettstown, NJ, USA; Newton Medical Center, Sparta, NJ, USA (R P Doshi MD); Department of Medicine (A C Dsouza MBBS, A Sathyanarayan MD, M Sharath MBBS), Bangalore Medical College and Research Institute, Bangalore, India; Department of Forensic Medicine and Toxicology (H L Dsouza MD), Kasturba Medical College Mangalore, Mangalore, India; Faculty of Health, Medicine and Life Sciences (FHML) (V S Dsouza MSc), Maastricht University, Maastricht, Netherlands; School of Medicine (Prof A R Duraes PhD), Federal University of Bahia, Salvador, Brazil; Department of Internal Medicine (Prof A R Duraes PhD), Escola Bahiana de Medicina e Saúde Pública (Bahiana School of Medicine and Public Health), Salvador, Brazil; Department of Conservative Dentistry with Endodontics (A M Dziedzic DSc), Medical University of Silesia, Katowice, Poland; Department of Orthopaedic Surgery (A Ebrahimi MD), Cardiovascular Research

Center (A Schuermans BSc), Massachusetts General Hospital, Boston, MA, USA (M Kim MD); Department of Behavioral and Community Health (M Ebrahimi Kalan PhD), University of Maryland, College Park, MD, USA; School of Nursing and Midwifery (Prof D Edvardsson PhD, Prof M Rahman PhD), Department of Public Health (H Jiang PhD), La Trobe University, Melbourne, VIC, Australia; School of Nursing and Midwifery (K Edvardsson PhD), La Trobe University, Bundoora, VIC, Australia; Advanced Nursing Department (F Efendi PhD), Department of Epidemiology Population Biostatistics and Health Promotion (A Hargono PhD), Universitas Airlangga (Airlangga University), Surabaya, Indonesia; Research Center for Public Health and Nutrition (D E Effendi MA), National Research and Innovation Agency Republic of Indonesia (BRIN), Jakarta, Indonesia; Faculty of Science and Health (M Ekholuenetale PhD), University of Portsmouth, Hampshire, UK; Almoosa College of Health Sciences, Al Ahsa, Saudi Arabia (R A El Arab PhD); Department of Public Health and Community Medicine (Prof I F El Bayoumy DrPH), Tanta University, Tanta city, Egypt; School of Public Health (Prof I F El Bayoumy DrPH), Texila American University, Guyana, Guyana; Biomedical Informatics and Medical Statistics Department (I El Sayed PhD), Tropical Health Department (R M Ghazy PhD), Department of Pathology (Prof I M Talaat PhD), Alexandria University, Alexandria, Egypt; Faculty of Medicine (M Elhadi MD), University of Tripoli, Tripoli, Libya; DeBakey Heart and Vascular Center (M Gupta MD), Houston Methodist Hospital, Houston, TX, USA (M Elhadi MD); Egypt Center for Research and Regenerative Medicine (ECRRM), Cairo, Egypt (M A Elmonem PhD); Department of Neuropsychiatry (Prof G ELNahas MD), Biochemistry Department (Prof N M Hamdy PhD), Department of Entomology (A M Samy PhD), Medical Ain Shams Research Institute (MASRI) (A M Samy PhD), Neurology Department (Prof A S Shalash PhD), Ain Shams University, Cairo, Egypt; Executive Committee (Prof G ELNahas MD), International Association for Women Mental Health, Potomac, MD, USA; Department of Infectious Diseases and Public Health (I Elsohaby PhD), Department of Public Health and Infectious Diseases (G Fekadu PhD), City University of Hong Kong, Hong Kong, China; Department of Animal Medicine (I Elsohaby PhD), Zagazig University, Zagazig, Egypt; Department of Pediatrics (C Eltaha MD), Texas A&M University, Dallas, TX, USA; National Eye Institute (M Emamverdi MD), National Human Genome Research Institute (NHGRI) (N Horita PhD), National Institutes of Health, Bethesda, MD, USA; Department of Public Health and Tropical Medicine (T I Emeto PhD), Australian Institute of Tropical Health and Medicine (A Pak PhD), James Cook University, Townsville, QLD, Australia (K O Obamiro PhD); Health Economics and Financing Practice Area (D A Erku PhD), Management Sciences for Health, Arlington, VA, USA; Drexel University Dornsife School of Public Health (E F Ezenwankwo MPH), Drexel University, Philadelphia, PA, USA; Independent Consultant, Bologna, Italy (N Fabin MD); Research Centre for Healthcare and Community (A F Fagbamigbe PhD), Faculty of Health and Life Sciences (O P Kurmi PhD), Coventry University, Coventry, UK; Department of Oral Biology (A Fahim PhD), Riphah International University, Islamabad, Pakistan; Epidemiology and Biostatistics Unit (L Falzone PhD), IRCCS Pascale, Naples, Italy; Department of Family Medicine (U Farooque MD), Luton & Dunstable University Hospital, Luton, UK; School of Engineering (A Fatehizadeh PhD), Edith Cowan University, Joondalup, Western Australia (WA), Australia; Centre for Public Health, Equity and Human Flourishing (N K Fauk PhD), Torrens University Australia, Adelaide, SA, Australia; Institute of Resource Governance and Social Change, Kupang, Indonesia (N K Fauk PhD); Department of Pharmacy (G Fekadu PhD), Institute of Health Sciences (B Feyisa MPH), Department of Public Health (D R Terefa MSc), Wollega University, Nekemte, Ethiopia; School of Population Health (X Feng PhD, Prof B A Saddik PhD), International Centre for Future Health Systems (J Lin PhD), Centre for Social Research in Health (S R Okeke PhD), Centre for Primary Health Care and Equity (CPHCE) (F Sitas PhD), University of New South Wales, Sydney, NSW, Australia; National Institute of Environmental Health (X Feng PhD), Chinese Center

for Disease Control and Prevention, Beijing, China; Center for Public Health Research (P Ferrara PhD), University of Milan Bicocca, Monza, Italy; Laboratory of Public Health (P Ferrara PhD), IRCCS Istituto Auxologico Italiano, Milan, Italy; Department of Social Sciences (Prof N Ferreira PhD), University of Nicosia, Nicosia, Cyprus; Institute of Public Health (F Fischer PhD), Charité Universitätsmedizin Berlin (Charité Medical University Berlin), Berlin, Germany; Institute of Gerontology (N A Foigt PhD), National Academy of Medical Sciences of Ukraine, Kyiv, Ukraine; Department of Neuroscience (M Foschi MD), Multiple Sclerosis Research Center, Ravenna, Italy; Department of Biotechnological and Applied Clinical Sciences (M Foschi MD), University of L'Aquila, L'Aquila, Italy; Department of Community Medicine and Family Medicine (S G MD, V J MD, V Rajendran MD), All India Institute of Medical Sciences, Gorakhpur, India; Health Services Management Training Centre (Prof P A Gaal PhD, T Joo PhD), Semmelweis University, Budapest, Hungary; Department of Applied Social Sciences (Prof P A Gaal PhD), Sapientia Hungarian University of Transylvania, Târgu-Mureş, Romania; Department of Community Medicine (Prof M A Gadanya MD), Aminu Kano Teaching Hospital, Kano, Nigeria; Department of Community Medicine (Prof A M Gaidhane MD), Datta Meghe Institute of Medical Sciences, Wardha, India; Department of Oral Biology and Experimental Dental Research (M Gajdács PhD), University of Szeged, Szeged, Hungary; Department of Medical Epidemiology (S Gallus PhD), Department of Environmental Health Sciences (A Lugo PhD), Mario Negri Institute for Pharmacological Research, Milan, Italy; Department of Community Medicine and Family Medicine (A P Gandhi MD), Department of Community and Family Medicine (J P Tripathy MD), All India Institute of Medical Sciences, Nagpur, India; Institute of Health and Wellbeing (B Ganesan PhD), Federation University Australia, Churchill, VIC, Australia; Professional Services Division (P Gautam PhD), Texas State Board of Pharmacy, Austin, TX, USA; Department of Pharmacology (Prof R K Gautam PhD), Indore Institute of Pharmacy, Indore, India; Department of Environmental Health (M Gebrehiwot DSc), Wollo University, Dessie, Ethiopia; Department of Reproductive and Family Health (T G Gebremeskel PhD), Axum College of Health Science, Axum, Ethiopia; Department of Public Health (L Getacher PhD), Debre Berhan University, Debre Berhan, Ethiopia; Family and Community Medicine Department (R M Ghazy PhD), King Khalid University, Abha, Saudi Arabia; Department of Radiology (A Gholamrezanezhad MD), University of Southern California, Los Angeles, CA, USA; Departments of Radiology and Neurosurgery (S Ghozy MD), Department of Nephrology and Hypertension (N Nikravangolsefid MD), Department of Radiology (F Nugen PhD), Department of Informatics and Radiology (S Vahdati MD), Mayo Clinic, Rochester, MN, USA; Country Office (A U Gil PhD), World Health Organization (WHO), Astana, Kazakhstan; Third Department of Neurology (E V Gnedovskaya PhD), Research Center of Neurology, Moscow, Russia; Department of Community Medicine & School of Public Health (Prof S Goel MD), Post Graduate Institute of Medical Education and Research, Chandigarh, India; College of Human and Health Sciences (Prof S Goel MD), Swansea University, Swansea, UK; Department of Health Systems and Policy Research (Prof M Golechha PhD), Indian Institute of Public Health, Gandhinagar, India; Department of Genetics (P Goleij MSc), Sana Institute of Higher Education, Sari, Iran; Universal Scientific Education and Research Network (USERN) (P Goleij MSc), Department of Infectious Disease (Prof S Vaziri MD), Kermanshah University of Medical Sciences, Kermanshah, Iran; Department of Life Sciences, Health and Healthcare Professions (Prof D Golinelli MD), Link Campus University, Rome, Italy; Health Services Research, Evaluation and Policy Unit (Prof D Golinelli MD), AUSL della Romagna, Ravenna, Italy; Department of Public Health and Preventive Medicine (Prof M Grivna PhD), Charles University, Prague, Czech Republic; Department of Preventive Oncology (S Grover MD), Centre for Health Innovation and Policy (CHIP) Foundation, Noida, India; School of Health System Studies (S Grover MD), Tata Institute of Social Sciences, Mumbai, India; Department of Epidemiology and Biostatistics (S

Guan MD, Prof H Pan PhD), Anhui Medical University, Hefei, China; Department of Clinical Science (M I M Gubari PhD), University Of Sulaimani, Sulaimani, Iraq; Division of Cardiovascular Medicine (A Guha MD), Ohio State University, Columbus, OH, USA; Health Directorate (S Guicciardi MD), Local Health Authority of Bologna, Bologna, Italy; Department of Community Medicine (D A Gunawardane MD), University of Peradeniya, Kandy, Sri Lanka; Department of Psychiatry (S Gunturu MD), Bronxcare Health System, Bronx, NY, USA; Group Health Department (Z Guo MPH), Nanyang Central Hospital, Nanyang, China; Department of Nephrology (A K Gupta PharmD), Max Super Specialty Hospital, New Delhi, India; Department of Public Health (B Gupta PhD), Torrens University Australia, Melbourne, VIC, Australia; Independent Consultant, Bharatpur, India (I Gupta MD); Independent Consultant, Delhi, India (I Gupta MD); Department of Preventive Cardiology & Medicine (Prof R Gupta MD), Eternal Heart Care Centre & Research Institute, Jaipur, India; Department of Medicine (Prof R Gupta MD), Mahatma Gandhi University Medical Sciences, Jaipur, India; Department of Toxicology (S Gupta MSc), Shriram Institute for Industrial Research, Delhi, India; School of Medicine (V Gupta PhD), Deakin University, Geelong, VIC, Australia; Department of Anthropology (V Gupta PhD), University of Delhi, Delhi, India; Faculty of Medicine Health and Human Sciences (Prof V K Gupta PhD), Australian Institute of Health Innovation (P Peprah MSc), Macquarie University, Sydney, NSW, Australia; Department of Clinical Pharmacology and Medicine (Prof N R Hadi PhD), University of Kufa, Najaf, Iraq; American University of Beirut, Faculty of Medicine (A Hajj Ali BS), American University of Beirut, Beirut, Lebanon; Department of Medical and Technical Information Technology (A Hammoud PhD), Bauman Moscow State Technical University, Moscow, Russia; Stroke Research Centre (Prof G J Hankey MD), Perron Institute for Neurological and Translational Science, Perth, WA, Australia; Research Unit (J M Haro MD), Parc Sanitari Sant Joan de Deu, Barcelona, Spain; Department of Mental Health (J M Haro MD), Biomedical Research Networking Center for Mental Health Network (CiberSAM), Madrid, Spain; Department of Zoology and Entomology (A I Hasaballah PhD, M G M Zeariya PhD), Al-Azhar University, Cairo, Egypt; Department of Nursing (F Hasan MSc), Department of Clinical Pharmacy (M A Sarasmita PharmD), Taipei Medical University, Taipei, Taiwan; Department of Health Research Methods, Evidence and Impact (M Hasan MPH), Department of Medicine (O P Kurmi PhD), Department of Psychiatry and Behavioural Neurosciences (A T Olagunju MD), Department of Health Research Methods, Evidence, and Impact (O O Olasupo PhD), McMaster University, Hamilton, ON, Canada; Department of Biochemistry and Molecular Biology (M Hasan MPH), Tejgaon College, Dhaka, Bangladesh; Department of Pharmacy (Prof M S Hasnain PhD), Palamau Institute of Pharmacy, Daltonganj, India; Public Health Department (I I Hassan PhD), Dalhatu Araf Specialist Hospital, Lafia, Nigeria; Department of Public Health (I I Hassan PhD), Federal University of Lafia, Lafia, Nigeria; Alberta Respiratory Centre (ARC) (B Heibati PhD), University of Alberta, Edmonton, AB, Canada; Department of Medicine (M Hemmati MD), MedStar Health, Washington, DC, USA; Department of Medicine (M Hemmati MD, C J Sabet MA), Georgetown University, Washington, DC, USA; Babes-Bolyai University, Cluj-Napoca, Romania (Prof C Herteliu PhD); Department of Public Health (D Z Heyi MPH), Madda Walabu University, Robe, Ethiopia; Department of Microbiology (K Hezam PhD), Faculty of Applied Sciences, Department of Microbiology (E A Noman PhD), Taiz University, Taiz, Yemen; School of Medicine (K Hezam PhD), Nankai University, Tianjin, China; Graduate School of Medicine (Y Hiraike PhD), Department of Global Health Policy (Prof S Nomura PhD, S K Rauniyar PhD), University of Tokyo, Tokyo, Japan; School of Dentistry (N Hoan DDS), Department of Allergy, Immunology and Dermatology (D H Nguyen MD), Hanoi Medical University, Hanoi, Vietnam; Department of Pulmonology (N Horita PhD), Yokohama City University, Yokohama, Japan; Department of Decision and Information Sciences (M Hossain DrPH), University of Houston, Houston, TX, USA; Public Health Research Group (M

Hossain DrPH), Nature Study Society of Bangladesh, Khulna, Bangladesh; Department of Public Health and Informatics (S Hossain MS), Jahangirnagar University, Dhaka, Bangladesh; School of Health and Society (H Hosseinzadeh PhD), University of Wollongong, Wollongong, NSW, Australia; Department of Clinical Legal Medicine (Prof S Hostiuc PhD), National Institute of Legal Medicine Mina Minovici, Bucharest, Romania; Department of Public Health and Community Medicine (Prof A Humayun PhD), Shaikh Zayed Postgraduate Medical Institute, Lahore, Pakistan; Department of Biological Sciences and Chemistry (Prof J Hussain PhD), Natural and Medical Sciences Research Center (A Khan PhD, A Ullah MS), School of Pharmacy (A K Philip PhD), University of Nizwa, Nizwa, Oman; Department of Occupational Safety and Health (Prof B Hwang PhD), China Medical University, Taiwan, Taichung, Taiwan; Department of Occupational Therapy (Prof B Hwang PhD), Asia University, Taiwan, Taichung, Taiwan; International Center for Nutrition and Information (N Ikeda PhD), National Institutes of Biomedical Innovation, Health and Nutrition, Tokyo, Japan; The National Centre for Remote and Rural Health and Care (A Ikiroma PhD), NHS National Services Scotland, Edinburgh, Scotland; West Africa RCC (O S Ilesanmi PhD), Africa Centre for Disease Control and Prevention, Abuja, Nigeria; Faculty of Medicine (I M Ilic PhD, Prof M M Santric-Milicevic PhD, A Stevanović MD), School of Public Health and Health Management (Prof M M Santric-Milicevic PhD), University of Belgrade, Belgrade, Serbia; Faculty of Medical Sciences (Prof M D Ilic PhD), University of Kragujevac, Kragujevac, Serbia; Department of Health Research (L R Inbaraj MD), ICMR National Institute for Research in Tuberculosis, Chennai, India; Faculty of Public Health (M Iqhrammullah PhD), Universitas Muhammadiyah Aceh, Banda Aceh, Indonesia; Faculty of Pharmacy (L M Irham PhD), Universitas Ahmad Dahlan, Yogyakarta, Indonesia; School of Pharmacy (M Islam PhD), BRAC University, Dhaka, Bangladesh; Institute for Physical Activity and Nutrition (S Islam PhD), Deakin University, Burwood, VIC, Australia; Department of Surveillance and Health Equity Science (F Islami PhD), American Cancer Society, Atlanta, GA, USA; School of Management (R Itumalla PhD), The Apollo University, Chittoor, India; Department of Health Services Research (M Iwagami PhD), Department of Public Health Medicine (Prof K Yamagishi MD), University of Tsukuba, Tsukuba, Japan; Department of Non-Communicable Disease Epidemiology (M Iwagami PhD), Department of Health Services Research and Policy (Prof M McKee DSc), London School of Hygiene & Tropical Medicine, London, UK; Department of Physical and Medicine (L Jacob MD), Université Paris Cité, Paris, France; Research and Development Unit (L Jacob MD), Biomedical Research Networking Center for Mental Health Network (CiberSAM), Barcelona, Spain; Department of Immunology (Prof A Jafarzadeh PhD), HIV/STI Surveillance Research Center (S Nejadghaderi MD), Kerman University of Medical Sciences, Kerman, Iran; Department of Immunology (Prof A Jafarzadeh PhD), Department of Epidemiology and Biostatistics (Prof M Rezaeian PhD), Rafsanjan University of Medical Sciences, Rafsanjan, Iran; Department of Nephrology (K Jaggi MD), San Mateo Medical Center, San Mateo, CA, USA; Department of Nephrology (K Jaggi MD), Mills Peninsula Medical Center, Burlingame, CA, USA; Department of Leukemia (A Jain MD), The University of MD Anderson Cancer Center, Houston, TX, USA; Statistics Unit (N Jain MD), Riga Stradins University, Riga, Latvia; Department of Health and Safety (A A Jairoun PhD), Dubai Municipality, Dubai, United Arab Emirates; Department of Population Health (S Jaka MD), New York University, New York, NY, USA; The World Academy of Sciences UNESCO, Trieste, Italy (Prof M Jakovljevic PhD); Shaanxi University of Technology, Hanzhong, China (Prof M Jakovljevic PhD); Department of Environmental Engineering (Prof R Jalilzadeh Yengejeh PhD), Islamic Azad University, Ahvaz, Iran; Department of International Health (H Zhang MS), Johns Hopkins University, Baltimore, MD, USA (E Jamshidi PharmD); School of Pharmaceutical Management (Prof M D Janodia PhD), IIHMR University, Jaipur, India; Department of Pharmacology (T Jawaid PhD), Imam Mohammad Ibn Saud Islamic University, Riyadh, Saudi Arabia;

Department of Biochemistry (Prof S Jayaram MD), Government Medical College, Mysuru, India; Department of Oral Medicine and Periodontology (Prof R D Jayasinghe MS), University of Peradeniya, Peradeniya, Sri Lanka; Department of Oral Medicine and Periodontology (Prof R D Jayasinghe MS), Saveetha Dental College and Hospitals (G Minervini PhD, M Tovani-Palone PhD), Centre of Molecular Medicine and Diagnostics (COMManD) (Prof S Patil PhD), Center for Global Health Research (Prof A Sahebkar PhD), Saveetha University, Chennai, India; Department of Epidemiology (R Jebai MPH, S Roy MPH), Florida International University, Miami, FL, USA; Department of Epidemiology and Health Promotion (Prof S Jee PhD), Yonsei University, Seoul, South Korea; Department of Internal Medicine (B M Jeswani MBBS), GCS Medical College, Hospital & Research Centre, Ahmedabad, India; Melbourne School of Population and Global Health (H Jiang PhD), School of Health Sciences (A Meretoja MD), Melbourne Medical School (J Zhang MD), University of Melbourne, Melbourne, VIC, Australia; Faculty of Veterinary Medicine (M Jokar DVM), Department of Oncology (L Yang PhD), University of Calgary, Calgary, AB, Canada; Young Researchers and Elite Club (M Jokar DVM), Islamic Azad University, Karaj, Iran; Rothschild Foundation Hospital (Prof J B Jonas MD), Institute of Molecular and Clinical Ophthalmology Basel, Paris, France; Singapore Eye Research Institute, Singapore, Singapore (Prof J B Jonas MD); Hungarian Health Management Association, Budapest, Hungary (T Joo PhD); Department of Economics (C E Joshua BSc), National Open University, Benin City, Nigeria; Department of Family Medicine and Public Health (J J Jozwiak PhD), University of Opole, Opole, Poland; Institute of Family Medicine and Public Health (M Jürisson PhD), University of Tartu, Tartu, Estonia; School of Public Health (Z Kabir PhD), University College Cork, Cork, Ireland; Department of Oral and Maxillofacial Pathology (V Kadashetti MDS), Department of Public Health Dentistry (Prof S M Kondlahalli MD), Krishna Vishwa Vidyapeeth (Deemed to be University), Karad, India; Department of Health Sciences (Prof M Kanaan PhD), University of York, York, UK; Faculty of Dentistry (K K Kanmodi MPH), University of Puthisastra, Phnom Penh, Cambodia; Office of the Executive Director (K K Kanmodi MPH), Cephas Health Research Initiative Inc, Ibadan, Nigeria; The Hansjörg Wyss Department of Plastic and Reconstructive Surgery (R S Kantar MD), NYU Langone Health, New York, NY, USA; Cleft Lip and Palate Surgery Division (R S Kantar MD), Global Smile Foundation, Norwood, MA, USA; 2nd Cardiology Department (P Karakasis MSc), 2nd Department of Cardiology (P Stachteas MSc), Aristotle University of Thessaloniki, Thessaloniki, Greece; School of Health Professions and Human Services (I M Karaye MD), Hofstra University, Hempstead, NY, USA; Department of Anesthesiology (I M Karaye MD), Montefiore Medical Center, Bronx, NY, USA; Social Determinants of Health Research Center (S Karimi PhD, Prof S Mohammad-Alizadeh-Charandabi PhD), Faculty of Nursing and Midwifery (Prof M Mirghafourvand PhD), Department of Midwifery (Prof S Mohammad-Alizadeh-Charandabi PhD), Tabriz University of Medical Sciences, Tabriz, Iran; Central Department of Public Health (P Karki MPH), Department of Community Medicine (P M S Pradhan MD), Tribhuvan University, Kathmandu, Nepal; Department of Physical Therapy and Health Rehabilitation (F Z Kashoo MSc), Majmaah University, Majmaah, Saudi Arabia; MRC/CSO Social and Public Health Sciences Unit (Prof S V Katikireddi PhD), School of Cardiovascular and Metabolic Health (F E Petermann-Rocha PhD), University of Glasgow, Glasgow, UK; Public Health Foundation of India, New Delhi, India (H Kaur MPH); Department of ENT (N Kaur MS), Dr. B. R. Ambedkar State Institute of Medical Sciences (AIMS), Mohali, India; Community Medicine Department (Prof T H Kazmi FCPS), Central Park Medical College, Lahore, Pakistan; Community Medicine & Public Health (Prof T H Kazmi FCPS), University of Health Sciences, Lahore, Pakistan; Open, Distance and eLearning Campus (Prof P N Keiyoro PhD), University of Nairobi, Nairobi, Kenya; Department of Human Nutrition (E Kesse-Guyot PhD), National Research Institute for Agriculture, Food and Environment, Jouy-en-Josas, France; Sorbonne Paris Nord University,

Bobigny, France (E Kesse-Guyot PhD); Amity Institute of Forensic Sciences (H Khajuria PhD, B P Nayak PhD), Amity Institute of Public Health (M Shannawaz PhD), Amity University, Noida, India; Department of Biostatistics (Prof A Khalilian PhD), Department of Medical-Surgical Nursing (S Shorofi PhD), Mazandaran University of Medical Sciences, Sari, Iran; Department of Primary Care (M A Khan MSc), NHS North West London, London, UK; College of Health, Wellbeing and Life Sciences (Prof K Khatab PhD), Sheffield Hallam University, Sheffield, UK; College of Arts and Sciences (Prof K Khatab PhD), Ohio University, Zanesville, OH, USA; Department of Internal Medicine (A A Khosla MD), Corewell Health East William Beaumont University Hospital, Royal Oak, MI, USA; Department of Clinical Research (S Khosravi MD), Icahn School of Medicine at Mount Sinai, New York City, NY, USA; Graduate School of Public Health (K Kim PhD), Yonsei University, Busan, South Korea; Broad Institute of MIT and Harvard, Cambridge, MA, USA (M Kim MD); School of Traditional Chinese Medicine (Y Kim PhD), Xiamen University Malaysia, Sepang, Malaysia; School of Health Sciences (Prof A Kisa PhD), Kristiania University College, Oslo, Norway; Department of International Health and Sustainable Development (Prof A Kisa PhD), Tulane University, New Orleans, LA, USA; Department of Physiology (S Komaki MD), Hamedan University of Medical Sciences, Hamedan, Iran; Department of Neurosurgery (M Korja PhD, I Rautalin PhD), General Administration Department (A Meretoja MD), Comprehensive Cancer Center (T J Meretoja MD), Helsinki University Hospital, Helsinki, Finland; Department of General Practice and Family Medicine (Prof O Korzh DSc), Kharkiv National Medical University, Kharkiv, Ukraine; Independent Consultant, Jakarta, Indonesia (S Kosen MD); Department of Epidemiology (Prof K Kostev PhD), IQVIA, Frankfurt am Main, Germany; University Hospital Marburg, Marburg, Germany (Prof K Kostev PhD); Department of Anthropology (Prof K Krishan PhD), Institute of Forensic Science & Criminology (V Sharma PhD), Panjab University, Chandigarh, India; Department of Demography (Prof B Kuate Defo PhD), Department of Social and Preventive Medicine (Prof B Kuate Defo PhD), University of Montreal, Montreal, QC, Canada; Department of Biochemistry (Prof M Kuddus PhD), College of Public Health & Health Informatics (R Kumar PhD), Department of Public Health (M G M Zeariya PhD), University of Hail, Hail, Saudi Arabia; Center of Medicine and Public Health (M Kulimbet MSc), Asfendiyarov Kazakh National Medical University, Almaty, Kazakhstan; Department of Cardiovascular Medicine (A Kumar MD), Cabrini Institute, Rochester, MN, USA; Geospatial Information Science and Engineering Hub (V Kumar PhD), Indian Institute of Technology, Mumbai, India; Centre for Studies in Economics and Planning (V Kumar PhD), Central University of Gujarat, Gandhinagar, India; Division of Cardiovascular Medicine (A Kundu MD), University of Kentucky, Lexington, KY, USA; School of Medicine and Dentistry (S Kundu MPH), School of Dentistry and Oral Health (S K Tadakamadla PhD), Griffith University, Gold Coast, QLD, Australia; Department of Nutrition and Food Science (S Kundu MPH), Department of Biochemistry and Food Analysis (N Roy PhD), Patuakhali Science and Technology University, Patuakhali, Bangladesh; Section of Cardiology (Prof S K Kunutsor PhD), University of Manitoba, Winnipeg, MB, Canada; Translational Health Sciences, Bristol Medical School (Prof S K Kunutsor PhD), University of Bristol, Bristol, UK; Department of Health Services Research and Management (D Kusuma DSc), City University of London, London, UK; Faculty of Public Health (D Kusuma DSc, Prof I Trihandini PhD), University of Indonesia, Depok, Indonesia; Department of Environment and Public Health (F Kyei-Arthur PhD), University of Environment and Sustainable Development, Somanya, Ghana; Clinical Research Center (V Kytö MD), Turku University Hospital, Turku, Finland; Heart Center (V Kytö MD), University of Turku, Turku, Finland; Integrated Department of Epidemiology, Health Policy, Preventive Medicine and Pediatrics (Prof C Lahariya MD), Foundation for People-centric Health Systems, New Delhi, India; Centre for Health: The Specialty Practice, New Delhi, India (Prof C Lahariya MD); School of Digital Science (D Lai PhD), Institute of Applied

Data Analytics (D Lai PhD), Faculty of Science (E Leong PhD), Institute of Health Sciences (S Ong FAMS), Universiti Brunei Darussalam (University of Brunei Darussalam), Bandar Seri Begawan, Brunei; Department of Occupational and Environmental Health (H Lai PhD), Yangzhou University, Yangzhou, China; Department of Respiratory and Critical Care Medicine (H Lai PhD), Northern Jiangsu People's Hospital, Yangzhou, China; Department of Otorhinolaryngology (S Lasrado MS), Father Muller Medical College, Mangalore, India; International Society Doctors for the Environment, Arezzo, Italy (P Lauriola MD); Department of General Medicine (V T Nguyen MD), Department of Internal Medicine (T H Tran MD), University of Medicine and Pharmacy at Ho Chi Minh City, Ho Chi Minh City, Vietnam (T T Le MD); College of Optometry (J L Leasher OD), Nova Southeastern University, Fort Lauderdale, FL, USA; Department of Medical Science (M Lee PhD), Ajou University School of Medicine, Suwon, South Korea; Department of Precision Medicine (Prof S Lee MD), Sungkyunkwan University, Suwon-si, South Korea; Department of Public Health (T Lerango MPH), Dilla University, Dilla, Ethiopia; Center for Dentistry and Oral Hygiene (A Li PhD), University of Groningen, Groningen, Netherlands; Stomatological Hospital (A Li PhD), Southern Medical University, Guangzhou, China; Department of Molecular Epidemiology (E Llanaj PhD), German Institute of Human Nutrition Potsdam-Rehbrücke, Potsdam, Germany; German Center for Diabetes Research (DZD), München-Neuherberg, Germany (E Llanaj PhD); One Health Research Group (J López-Gil PhD), Universidad de Las Américas, Quito, Ecuador; School of Medicine (Prof G Lucchetti PhD), Federal University of Juiz de Fora, Juiz de Fora, Brazil; Department of Population Health Sciences (J B Lusk MD), Duke University, Durham, NC, USA; Department of Chemistry (H I M Amin PhD), Salahaddin University-Erbil, Erbil, Iraq; Department of Medical Biochemical Analysis (H I M Amin PhD), Cihan University-Erbil, Erbil, Iraq; Centre for Public Health and Wellbeing (Z Ma PhD), University of the West of England, Bristol, UK; Department of Periodontology (Prof M Machoy PhD), Pomeranian Medical University, Szczecin, Poland; Department of Biostatistics and Epidemiology (F Madadizadeh PhD), Yazd University of Medical Sciences, Yazd, Iran; Department of Medicine (O M Makram MD), Medical College of Georgia at Augusta University, Augusta, GA, USA; Rama Medical College Hospital and Research Centre, Uttar Pradesh, India (K Malhotra MBBS); Biomedical Engineering Research Center (CREB) (H Marateb PhD), Universitat Politècnica de Catalunya (Barcelona Tech - UPC), Barcelona, Spain; Department of Biomedical Engineering (H Marateb PhD), University of Isfahan, Isfahan, Iran; Psychiatry Department (J Martinez-Raga PhD), Hospital Universitario Doctor Peset, Valencia, Spain; Department of Medicine (J Martinez-Raga PhD), University of Valencia, Valencia, Spain; Department of Nutrition and Dietetics (M Martorell PhD), Centre for Healthy Living (M Martorell PhD), University of Concepción, Concepción, Chile; Faculty of Humanities and Health Sciences (Prof R R Marzo MD), Curtin University, Sarawak, Malaysia; Jeffrey Cheah School of Medicine and Health Sciences (Prof R R Marzo MD), Monash University, Subang Jaya, Malaysia; Department of Anatomy, Genetics and Biomedical Informatics (Y Mathangasinghe PhD), University of Colombo, Colombo, Sri Lanka; Department of Public Health and Community Medicine (E Mathews PhD), Central University of Kerala, Kasaragod, India; Community Medicine (M Mathur MD), Department of General Medicine (N Mathur MD), Geetanjali Medical College and Hospital, Udaipur, India; Department of Healthcare (Prof E A Mechili PhD), University of Vlora, Vlora City, Albania; Clinic of Social and Family Medicine (Prof E A Mechili PhD), Laboratory of Toxicology (T K Nikolouzakis PhD, C Vardavas PhD), University of Crete, Heraklion, Greece; Centre for Health Innovation and Policy, Noida, India (Prof R Mehrotra PhD); Department of Public Health (T Mekene Meto MPH), Arba Minch University, Arba Minch, Ethiopia; School of Nursing and Midwifery (B Mekonnen MPH), Deakin University, Melbourne, VIC, Australia; Universidad Nacional Mayor de San Marcos, Lima, Peru (W Mendoza MD); Division of Forensic Medicine (Prof R G Menezes MD), Imam Abdulrahman Bin Faisal

University, Dammam, Saudi Arabia; University Centre Varazdin (T Mestrovic PhD), University North, Varazdin, Croatia; Icelandic Centre for Social Research and Analysis, Reykjavik, Iceland (C C A Meyers BSc); Icelandic Centre for Social Research and Analysis (ICSRA) (C C A Meyers BSc), Reykjavik University, Reykjavik, Iceland; National Cancer Registry (I Michalek PhD), Department of Pathology (I Michalek PhD), Maria Sklodowska-Curie National Research Institute of Oncology, Warsaw, Poland; Pacific Institute for Research & Evaluation, Calverton, MD, USA (T R Miller PhD); Multidisciplinary Department of Medical-Surgical and Dental Specialties (G Minervini PhD), University of Campania "Luigi Vanvitelli", Naples, Italy; Internal Medicine Programme (Prof E M Mirrakhimov PhD), Kyrgyz State Medical Academy, Bishkek, Kyrgyzstan; Department of Atherosclerosis and Coronary Heart Disease (Prof E M Mirrakhimov PhD), National Center of Cardiology and Internal Disease, Bishkek, Kyrgyzstan; College of Healthcare Management and Economics (V Mishra PhD), Gulf Medical University, Ajman, United Arab Emirates; Research and Development Department (V Mishra PhD), Panacea Institute of Interdisciplinary Research and Education, Varanasi, India; College of Health Science (A I Mohamed MSc), College of Applied and Natural Science (J Mohamed MSc), University of Hargeisa, Hargeisa, Somalia; Molecular Biology Unit (N S Mohamed MSc), Bio-Statistical and Molecular Biology Department (N S Mohamed MSc), Sirius Training and Research Centre, Khartoum, Sudan; College of Medicine (Prof A M Mohammad MD), University of Duhok, Duhok, Iraq; Department of Public Health (H Mohammed PhD), Dire Dawa University, Dire Dawa, Ethiopia; Health Systems and Policy Research Unit (Prof S Mohammed PhD), Department of Community Medicine (A A Olorukooba MD), Ahmadu Bello University, Zaria, Nigeria; School of Health Sciences (S Mohan PhD), University of Petroleum and Energy Studies, Dehradun, India; Institute of Clinical Physiology (S Molinaro PhD), National Research Council, Pisa, Italy; Faculty of Medicine and University Hospital Cologne (Prof U Mons PhD), University of Cologne, Cologne, Germany; Faculty of Medicine (A Moodi Ghalibaf MD), Birjand University of Medical Sciences, Birjand, Iran; Department of Public Health (Prof R S Moreira PhD), Oswaldo Cruz Foundation, Recife, Brazil; Department of Public Health (Prof R S Moreira PhD), Federal University of Pernambuco, Recife, Brazil; Division of Plastic and Reconstructive Surgery (S D Morrison MD), University of Washington Medical Center, Seattle, WA, USA; Department of Surgery (G D Mukoro MD), Ahmadu Bello University Teaching Hospital, Zaria, Nigeria; Department of Surgery (F Mulita PhD), General University Hospital of Patras, Patras, Greece; Faculty of Medicine (F Mulita PhD), Department of Emergency Medicine (Prof I Pantazopoulos PhD), University of Thessaly, Larissa, Greece; Department of Health Economics (M Muniyandi PhD), National Institute for Research in Tuberculosis, Chennai, India; Department of Community and Global Health (Y Munkhsaikhan MD), The University of Tokyo, Tokyo, Japan; Clinical Epidemiology Research Unit (E Murillo-Zamora PhD), Mexican Institute of Social Security, Villa de Alvarez, Mexico; Postgraduate in Medical Sciences (E Murillo-Zamora PhD), Universidad de Colima, Colima, Mexico; Department of Psychiatry (W Myung PhD), Department of Food and Nutrition (A P Okekunle PhD), Seoul National University, Seoul, South Korea; Department of Neuropsychiatry (W Myung PhD), Seoul National University Bundang Hospital, Seongnam, South Korea; Department of Computer Science (P Naghavi MS), University of Illinois Urbana-Champaign, Urbana, IL, USA; Department of Engineering (G R Naik PhD), Translational Health Research Institute (K Rana PhD), Western Sydney University, Sydney, NSW, Australia; Department of Medical Laboratory Analysis (H H Najmuldeen PhD), Cihan University Sulaymaniya, Sulaymaniyah, Iraq; Department of Dermatology (Prof L Naldi MD), San Bortolo Hospital, Vicenza, Italy; GISED Study Center, Bergamo, Italy (Prof L Naldi MD); Department of Health and Rehabilitation Sciences (Prof G Nambi PhD), Prince Sattam bin Abdulaziz University, Al Kharj, Saudi Arabia; Suraj Eye Institute, Nagpur, India (V Nangia MD); Department for the Control of Disease, Epidemics, and Pandemics (J Nansseu MD), Ministry of Public Health, Yaoundé,

Cameroon; Department of Public Heath (J Nansseu MD), University of Yaoundé I, Yaoundé, Cameroon; National Dental Research Institute Singapore (G G Nascimento PhD), Duke-NUS Medical School, Singapore, Singapore; Nursing & Midwifery Research Department (NMRD) (A J Nashwan PhD), Department of Geriatric and Long Term Care (B Sathian PhD), Hamad Medical Corporation, Doha, Qatar; Department of Circulation and Medical Imaging (J Nauman PhD), Norwegian University of Science and Technology, Trondheim, Norway; Department of Biotechnology (M Naveed PhD), University of Central Punjab, Lahore, Pakistan; School of Medicine (N Nawsherwan PhD), School of Life Sciences (M Suleman PhD), Xiamen University, Xiamen, China; Department of Health Promotion (A Nazri-Panjaki MSc), Health Promotion Research Center (H Okati-Aliabad PhD), Zahedan University of Medical Sciences, Zahedan, Iran; School of Pharmacy (S O Nduaguba PhD), West Virginia University, Morgantown, WV, USA; Department of Cardiology (R I Negoi PhD), Cardio-Aid, Bucharest, Romania; Faculty of Medicine (Prof C Nejjari PhD), Euromed University of Fes, Fez, Morocco; Faculty of Medicine (Prof C Nejjari PhD), University Sidi Mohammed Ben Abdellah, Fez, Morocco; Department of Health Sciences (S Neupane PhD), University of Tampere, Tampere, Finland; Department of Biological Sciences (J W Ngunjiri PhD), University of Embu, Embu, Kenya; Cardiovascular laboratory (D H Nguyen MD), Methodist Hospital, Merrillville, Merrillville, IN, USA; Faculty of Medicine (H T H Nguyen MD), Institute for Research and Training in Medicine, Biology and Pharmacy (H T H Nguyen MD), Duy Tan University, Da Nang, Vietnam; Cardiovascular Research Department (H Q Nguyen MD), Methodist Hospital, Merrillville, IL, USA; Department of Surgery (P T Nguyen MD), Danang Family Hospital, Danang, Vietnam; Hitotsubashi Institute for Advanced Study (HIAS) (P T Nguyen DrPH), Hitotsubashi University, Tokyo, Japan; Institute for Cancer Control (P T Nguyen DrPH), National Cancer Center, Chuo-ku, Japan; Institute for Mental Health Policy Research (Y T Nigatu PhD), Centre for Addiction and Mental Health, Toronto, ON, Canada; Department of General Surgery (T K Nikolouzakis PhD), University Hospital of Heraklion, Heraklion, Crete, Greece; Department of Public Health (V Niranjan PhD), HSE Ireland, Dublin, Ireland; Department of Public Health (V Niranjan PhD), UNICAF, Larnaca, Cyprus; Technical Department (C A Nnaji PhD), School of Public Health and Family Medicine (C A Nnaji PhD), SAMRC Unit on Risk and Resilience in Mental Disorders (Prof D J Stein FRCPC), University of Cape Town, Cape Town, South Africa; Center for Public Health (L A Nnyanzi PhD), Teesside University, Middlesbrough, UK; Global Research Institute (Prof S Nomura PhD), Keio University, Tokyo, Japan; Department of Statistics (S Noor MS), Shahjalal University of Science and Technology, Sylhet, Bangladesh; Department of Microbiology and Molecular Genetics (M Noreen PhD), The Women University Multan, Multan, Pakistan; School of Health (M Nozari PhD), Bam University of Medical Sciences, Bam, Iran; School of Information (F Nugen PhD), University of California Berkeley, Berkeley, CA, USA; Center of Excellence in Reproductive Health Innovation (CERHI) (C I Nzoputam MPH), University of Benin, Benin City, Nigeria; Department of Physiology (O J Nzoputam PhD), University of Benin, Edo, Nigeria; Department of Physiology (O J Nzoputam PhD), Benson Idahosa University, Benin City, Nigeria; Department of Applied Economics and Quantitative Analysis (Prof B Oancea PhD), University of Bucharest, Bucharest, Romania; Department of Public Health (D B Odo MPH), Arsi University, Asella, Ethiopia; Department of Community Health and Primary Care (O O Odukoya MSc), University of Lagos, Idi Araba, Nigeria; Department of Family and Preventive Medicine (O O Odukoya MSc), University of Utah, Salt Lake City, UT, USA; PSSM Data Sciences (M Oduro PhD), Pfizer Inc., Groton, CT, USA; Sheffield Centre for Health and Related Research (J O Oguta MSc), University of Sheffield, Sheffield, UK; Department of Preventive Medicine (Prof I Oh MD), Department of Pediatrics (Prof D Yon MD), Kyung Hee University, Seoul, South Korea; School of Pharmacy (O C Okonji MSc), University of the Western Cape, Cape Town, South Africa; Department of Psychiatry (A T Olagunju MD),

University of Lagos, Lagos, Nigeria; Department of Nursing Science (M I Olatubi PhD), Bowen University, Iwo, Nigeria; Cardiology Department (G M M Oliveira PhD), Federal University of Rio de Janeiro, Rio de Janeiro, Brazil; Surgery Department (G L Omer MD), Sulaimani University, Sulaimani, Iraq; ENT Department (G L Omer MD), Tor Vergata University of Rome, Rome, Italy; Department of Public Health (S Ong FAMS), Ministry of Health, Bandar Seri Begawan, Brunei; Department of Geography (A Opejin MS), East Carolina University, Greenville, NC, USA; Department of Pharmacotherapy and Pharmaceutical Care (M Ordak PhD), Department of Biochemistry and Pharmacogenomics (M Zielińska MPharm), Medical University of Warsaw, Warsaw, Poland; Sickle Cell Unit (Prof V N Orish PhD), Ho Teaching Hospital, Ho, Ghana; One Health Global Research Group (Prof E Ortiz-Prado PhD), Universidad de las Americas (University of the Americas), Quito, Ecuador; School of Medicine (U L Osuagwu PhD), Western Sydney University, Bathurst, NSW, Australia; Department of Optometry and Vision Science (U L Osuagwu PhD), University of KwaZulu-Natal, KwaZulu-Natal, South Africa; Laboratory of Public Health Indicators Analysis and Health Digitalization (S S Otstavnov PhD), Department of Information Technologies and Management (S K Vladimirov PhD), Moscow Institute of Physics and Technology, Dolgoprudny, Russia; Department of Project Management (S S Otstavnov PhD), National Research University Higher School of Economics, Moscow, Russia; Division of Infectious Diseases (Prof A Ouyahia PhD), University Hospital of Setif, Setif, Algeria; Department of Respiratory Medicine (Prof M P P A DNB), Department of Oral and Maxillofacial Surgery (C S N PhD), Jagadguru Sri Shivarathreeswara University, Mysore, India; National School of Public Health (A Padron-Monedero PhD), Institute of Health Carlos III, Madrid, Spain; Department of Mental Health (R F Palma-Alvarez PhD), Hospital Universitari Vall d'Hebron (Vall d'Hebron University Hospital), Barcelona, Spain; Department of Psychiatry, Mental Health and Addictions (R F Palma-Alvarez PhD), Vall d'Hebron Institut de Recerca (Vall d'Hebron Research Institute), Barcelona, Spain; Department of Nutrition and Dietetics (Prof D Panagiotakos PhD), Harokopio University, Athens, Greece; Board of Directors (Prof D Panagiotakos PhD), National Public Health Organization, Athens, Greece; Department of Neurology (L D Panos MD), Department of Emergency Medicine (Prof I Pantazopoulos PhD), University of Bern, Bern, Switzerland; Department of Neurology (L D Panos MD), University of Cyprus, Nicosia, Cyprus; Department of Science and Mathematics (Prof P Papadopoulou PhD), Deree-The American College of Greece, Athens, Greece; Department of Biophysics (Prof P Papadopoulou PhD), 3rd Department of Cardiology (M Spartalis PhD), University of Athens, Athens, Greece; Vision and Eye Research Institute (Prof S Pardhan PhD), Anglia Ruskin University, Cambridge, UK; Department of Community Medicine (P P Parija MD), All India Institute of Medical Sciences, Jammu, India; Department of Epidemiology and Community Health (R R Parikh MD), University of Minnesota, Minneapolis, MN, USA; Department of Medical Humanities and Social Medicine (Prof E Park PhD), Kosin University, Busan, South Korea; Department of Biomedical Data Science (S Park MD), Stanford University, Stanford, CA, USA; Department of Medical Sciences (R Passera PhD), University of Torino, Torino, Italy; Department of Imaging (R Passera PhD), AOU Città della Salute e della Scienza di Torino, Torino, Italy; Global Health Governance Programme (J Patel BSc), Centre for Medical Informatics (Prof A Sheikh MD), University of Edinburgh, Edinburgh, UK; Department of Research and Training (S K Patel PhD), Population Council Institute, New Delhi, India; College of Dental Medicine (Prof S Patil PhD), Roseman University of Health Sciences, South Jordan, UT, USA; School of Population Health (Prof G Pereira PhD), Curtin University, Bentley, WA, Australia; Centre for Fertility and Health (Prof G Pereira PhD), Department of Chemical Toxicology (M W Wojewodzic PhD), Norwegian Institute of Public Health, Oslo, Norway; Pennsylvania Cancer and Regenerative Medicine Center (R G Pestell MD), Baruch S Blumberg Institute, Doylestown, PA, USA; Department of Medicine (R G Pestell MD), Xavier University

School of Medicine, Woodbury, NY, USA; Facultad de Medicina (F E Petermann-Rocha PhD), Universidad Diego Portales (Diego Portales University), Santiago, Chile; Shanghai Mental Health Center (Prof M R Phillips MD), Shanghai Jiao Tong University, Shanghai, China; Department of Medicine (Prof D Poddighe PhD), Nazarbayev University, Astana, Kazakhstan; Clinical Academic Department of Pediatrics (Prof D Poddighe PhD), University Medical Center (UMC), Astana, Kazakhstan; Department of Epidemiology and Evidence-Based Medicine (R V Polibin PhD), I.M. Sechenov First Moscow State Medical University, Moscow, Russia; Department of Data Management and Analysis (R Poluru PhD), The International Clinical Epidemiology Network (INCLEN) Trust International, New Delhi, India; Department of Public Health (F Porru MD), Erasmus University Medical Center, Rotterdam, Netherlands; Humanities and Social Sciences (Prof J Pradhan PhD), National Institute of Technology Rourkela, Rourkela, India; Department of Clinical Research and Epidemiology (M Prasad MD), Institute of Liver and Biliary Sciences, New Delhi, New Delhi, India; Health Sciences Department (D R A Pribadi MSc), Muhammadiyah University of Surakarta, Sukoharjo, Indonesia; Centre for Dental Education and Research (B M Purohit MDS), Department of Radiation Oncology (A Shankar MD), All India Institute of Medical Sciences, New Delhi, India; Department of Biostatistics, Epidemiology, and Informatics (J Puvvula PhD), University of Pennsylvania, Philadelphia, PA, USA; Department of Medical Oncology (Prof V Radhakrishnan MD), Cancer Institute (W.I.A), Chennai, India; Department of Medical Laboratory Technologies (Prof F Rahim PhD), Al-Noor Center of Research and Innovation (Prof F Rahim PhD), Alnoor University, Mousl, Iraq; Department of Population Science and Human Resource Development (Prof M Rahman PhD, Prof M Rahman DrPH), University of Rajshahi, Rajshahi, Bangladesh; Institute of Health and Wellbeing (Prof M Rahman PhD), Federation University Australia, Berwick, VIC, Australia; Centre for Chronic Disease Control, New Delhi, India (P Rajput PhD); Department of Cardiology (P Ram MD), Emory University, Atlanta, GA, USA; Department of Cardiology (Prof M M Ramadan PhD), Faculty of Pharmacy (Prof M A Saleh PhD), Mansoura University, Mansoura, Egypt; Department of Population Health (M Ramadan DrPH), King Saud bin Abdulaziz University for Health Sciences, Jeddah, Saudi Arabia; Department of Community Medicine (R K Rana MD), Shaheed Nirmal Mahto Medical College and Hospital, Dhanbad, India; Department of Research (C L Ranabhat PhD), Eastern Scientific LLC, Richmond, KY, USA; Department of Health Promotion and Administration (C L Ranabhat PhD), Eastern Kentucky University, Richmond, KY, USA; Department of Oral Pathology, Microbiology and Forensic Odontology (S Rao MDS), Sharavathi Dental College and Hospital, Shimogga, India; Thrombosis Research Group (S Rashedi MD), Brigham and Women's Hospital, Harvard Medical School, Boston, MA, USA; Department of Medicine (A M Rashid MD), Jinnah Sindh Medical University, Karachi, Pakistan; Baylor University, Dallas, TX, USA (A M Rashid MD); Department of Family Medicine (Prof D Rathish MPH), Department of Community Medicine (N D Wickramasinghe MD), Rajarata University of Sri Lanka, Anuradhapura, Sri Lanka; The National Institute for Stroke and Applied Neurosciences (I Rautalin PhD), Auckland University of Technology, Auckland, New Zealand; Section of Pulmonary and Critical Care Medicine (N Ravikumar MD), University of Chicago, Chicago, IL, USA; Academic Public Health England (Prof S Rawaf MD), Public Health England, London, UK; Department of Biological Sciences (Prof E M M Redwan PhD), King Abdulaziz University, Jeddah, Egypt; Department of Protein Research (Prof E M M Redwan PhD), Research and Academic Institution, Alexandria, Egypt; Department of Public Health (A Riad PhD), Czech National Centre for Evidence-based Healthcare and Knowledge Translation (A Riad PhD), Masaryk University, Brno, Czech Republic; Department of Geography and Demography (M Rodrigues PhD), University of Coimbra, Coimbra, Portugal; Department of Nursing in Women's Health (T R Rodrigues da Silva PhD), Federal University of São Paulo, São Paulo, Brazil; Department of Pharmacology and

Toxicology (Prof J A B Rodriguez PhD), University of Antioquia, Medellin, Colombia; Warwick Medical School (Prof J A B Rodriguez PhD), University of Warwick, Coventry, UK; Department of Clinical Research (Prof L Roever PhD), University of Sao Paulo, Ribeirão Preto, Brazil; Golestan Research Center of Gastroenterology and Hepatology (G Roshandel PhD), Golestan University of Medical Sciences, Gorgan, Iran; Department of Analytical and Applied Economics (Prof H Rout PhD, C Swain MPhil), RUSA Centre of Excellence in Public Policy and Governance (Prof H Rout PhD), UGC Centre of Advanced Study in Psychology (M Satpathy PhD), Utkal University, Bhubaneswar, India; Faculty of Medicine (B Roy PhD), Quest International University Perak, Ipoh, Malaysia; Advanced Campus Governador Valadares (Prof G d Ruela MSc), Juiz de For a Federal University, Governador Valadares, Brazil; Department of Nursing (Prof G d Ruela MSc), Universidade Presidente Antônio Carlos (President Antônio Carlos University), Governador Valadares, Brazil; Faculty of Health and Dentistry (K P Sadarangani PhD), Diego Portales University, Santiago de Chile, Chile; Subdirección de Desarrollo Académico e investigación (K P Sadarangani PhD), Instituto Teletón, Santiago de Chile, Chile; Department of Pharmaceutical Chemistry (Prof M Saeb PhD), International Medical University, Gdańsk, Poland; Clinical and Biomedical Research Center (Prof U Saeed PhD), Foundation University Islamabad, Islamabad, Pakistan; International Center of Medical Sciences Research (ICMSR), Islamabad, Pakistan (Prof U Saeed PhD); Faculty of Medicine, Bioscience and Nursing (S Safi PhD), MAHSA University, Selangor, Malaysia; Interdisciplinary Research Centre in Biomedical Materials (IRCBM) (S Safi PhD), COMSATS Institute of Information Technology, Lahore, Pakistan; Department of Community Medicine and Family Medicine (S S Sahoo MD, M Verma MD), Department of Radiodiagnosis (P Singh MD), All India Institute of Medical Sciences, Bathinda, India; Department of Health and Kinesiology (M Sajib BDS), University of Illinois, Urbana-Champaign, IL, USA; Department of Statistics (M R Sajid PhD), University of Gujrat, Gujrat, Pakistan; Department of Integrated Health Education (Prof L B Salaroli PhD), Federal University of Espirito Santo, Vitória, Brazil; Technology Management Department (Prof M Z Y Salem PhD), University College of Applied Sciences, Gaza, Palestine; School of Economics and Management (Prof M Z Y Salem PhD), University of Kassel, Kassel, Germany; College of Nursing (D Salihu PhD), Jouf University, Jouf, Saudi Arabia; Institute of Epidemiology and Preventive Medicine (Y L Samodra PhD), National Taiwan University, Taipei, Taiwan; Benang Merah Research Center (BMRC), Minahasa Utara, Indonesia (Y L Samodra PhD); Department of Surgery (Prof J Sanabria MD), Marshall University, Huntington, WV, USA; Research Development Coordination Section (M N Saqib PhD), Pakistan Health Research Council, Islamabad, Pakistan; Pharmacy Study Program (M A Sarasmita PharmD), Udayana University, Badung, Indonesia; Indira Gandhi Medical College and Research Institute, Puducherry, India (A Saravanan MD); Department of Orthopaedics and Trauma Surgery (B Saravi PhD), University of Freiburg, Freiburg, Germany; Department of Orthopaedics (B Saravi PhD), Loretto Hospital Freiburg, Freiburg, Germany; Department of Public Health (Y Sarikhani PhD), Jahrom University of Medical Sciences, Jahrom, Iran; Department of Food Processing Technology (T Sarkar PhD), West Bengal State Council of Technical Education, Malda, India; Department of Oral Pathology and Microbiology (Prof G S Sarode PhD, Prof S C Sarode PhD), Dr D Y Patil Vidyapeeth, Pune, Pune, India; Faculty of Health & Social Sciences (B Sathian PhD), Bournemouth University, Bournemouth, UK; Udyam-Global Association for Sustainable Development, Bhubaneswar, India (M Satpathy PhD); Department of Public Health Sciences (M Sawhney PhD), University of North Carolina at Charlotte, Charlotte, NC, USA; Psychiatry Clinic (M Saylan MD), Holy Savior Armenian Hospital, Istanbul, Turkiye; Department of Diagnostic and Interventional Radiology and Neuroradiology (Prof B M Schaarschmidt MD), University Hospital Essen, Essen, Germany; Swiss Research Institute for Public Health and Addiction (M P Schaub PhD), University of Zürich, Zurich, Switzerland; Department of Cardiovascular

Sciences (A Schuermans BSc, J Van den Eynde BSc), Katholieke Universiteit Leuven, Leuven, Belgium; Department of Community Oral Health and Clinical Prevention (S Selvaraj PhD), University of Malaya, Kuala Lumpur, Malaysia; Emergency Department (S Senthilkumaran PhD), Manian Medical Centre, Erode, India; Department of Medicine and Surgery (Y Sethi MBBS), Government Doon Medical College, Dehradun, India; National Heart, Lung, and Blood Institute (A Seylani BS), National Institutes of Health, Rockville, MD, USA; Non-communicable Diseases Research Center (A Shafiee MD), Alborz University of Medical Sciences, Karaj, Iran; Department of Chemistry (H Shahsavari PhD), Institute for Advanced Studies in Basic Sciences (IASBS), Zanjan, Iran; Institute for Critical Care Medicine (A Shaikh MD), Mount Sinai Health System, New York, NY, USA; Independent Consultant, Karachi, Pakistan (M A Shaikh MD); Department for Evidence-based Medicine and Evaluation (A Sharifan PharmD), University for Continuing Education Krems, Krems, Austria; K S Hegde Medical Academy (Prof M Shetty MD), Nitte University, Mangalore, India; Department of Public Health (D Shiferaw MPH), Dambi Dollo University, Dembi Dollo, Ethiopia; Department of Pharmacology (T Shimels MSc), Saint Paul's Hospital Millennium Medical College, Addis Ababa, Ethiopia; Finnish Institute of Occupational Health, Helsinki, Finland (R Shiri PhD); Oulu Business School (I Shiue PhD), Martti Ahtisaari Institute (I Shiue PhD), University of Oulu, Oulu, Finland; Department of Experimental Research (V Shivarov PhD), Medical University Pleven, Sofia, Bulgaria; Department of Genetics (V Shivarov PhD), Sofia University "St. Kliment Ohridiski", Sofia, Bulgaria; Department of Research and Academics (S Shrestha PharmD), Kathmandu Cancer Center, Bhaktapur, Nepal; Department of Medical Microbiology and Infectious Diseases (E E Siddig MD), Erasmus University, Rotterdam, Netherlands; Department of Dentistry (Prof A Singh MDS), All India Institute of Medical Sciences, Bhopal, India; Department of Pharmacology (H Singh DM), Government Medical College and Hospital, Chandigarh, India; School of Medicine (Prof J A Singh MD), Henry JN Taub Department of Emergency Medicine (Prof L Szarpak PhD), Baylor College of Medicine, Houston, TX, USA; Department of Medicine Service (Prof J A Singh MD), US Department of Veterans Affairs (VA), Houston, TX, USA; Department of Human Genetics (P Singh PhD), Punjabi University, Patiala, India; Department of Pulmonary Medicine (Prof V Singh MD), Mahaveer Jaipuria Rajasthan Hospital, Jaipur, India; Department of Systemic Pathology (R Solanki MD), Touro College of Osteopathic Medicine, Middletown, NY, USA; Department of Pathology (R Solanki MD), American University of the Caribbean School of Medicine, Cupecoy, Saint Martin; Department of Neuroscience (M Solmi MD), University of Ottawa, Ottawa, ON, Canada; Student Research Committee (S Soraneh MD), Urmia University of Medical Sciences, Urmia, Iran; School of Medicine (S Soraneh MD), Babol University of Medical Sciences, Babol, Iran; Hospital Universitario de La Princesa (Prof J B Soriano MD), Universidad Autónoma de Madrid (Autonomous University of Madrid), Madrid, Spain; Centro de Investigación Biomédica en Red Enfermedades Respiratorias (CIBERES) (Center for Biomedical Research in Respiratory Diseases Network), Madrid, Spain (Prof J B Soriano MD); Hull York Medical School (I N Soyiri PhD), University of Hull, Hull City, UK; Department of Medicine (P Steiropoulos MD), Democritus University of Thrace, Alexandroupolis, Greece; Global Observatory on Pollution and Health (Prof K Straif PhD), Boston College, Chestnut Hill, MA, USA; ISGlobal Instituto de Salud Global de Barcelona, Barcelona, Spain (Prof K Straif PhD); Department of Disease Burden (G Sulo PhD), GBD Collaborating Unit (Prof S E Vollset DrPH), Norwegian Institute of Public Health, Bergen, Norway; Department of Biomedical Sciences (Z Sun PhD), Universiti Putra Malaysia, Selangor, Malaysia; Department of Clinical Research and Development (Prof L Szarpak PhD), LUXMED Group, Warsaw, Poland; Department of Neurology (P Tabaee Damavandi MD), Neurocenter of Southern Switzerland (NSI), Lugano, Switzerland; Department of Basic Medical Sciences (S Tabatabaeizadeh PhD), Department of Internal Medicine (S Tabatabaeizadeh PhD), Islamic Azad

University, Mashhad, Iran; Dentistry and Oral Health, Rural Clinical Sciences (J Tadakamadla PhD), La Trobe University, Bendigo, VIC, Australia; Department of Environmental, Agricultural and Occupational Health (J Taiba PhD), University of Nebraska Medical Center, Omaha, NE, USA; Sri Ramachandra Medical College and Research Institute, Chennai, India (J Taiba PhD); National Centre for Epidemiology and Population Health (A Talukder MSc), Australian National University, Acton, ACT, Australia; Statistics Discipline (A Talukder MSc), Khulna University, Khulna, Bangladesh; Department of Dermato-Venereology (M Tampa PhD), Dr. Victor Babes Clinical Hospital of Infectious Diseases and Tropical Diseases, Bucharest, Romania; Department of Epidemiology (J L J Tamuzi MSc), Stellenbosch University, Cape Town, South Africa; Department of Medicine (J L J Tamuzi MSc), Northlands Medical Group, Omuthiya, Namibia; National Research and Innovation Agency, Jakarta, Indonesia (I U Tarigan PhD); Department of Urology (M Teimoori MD), Sabzevar University of Medical Sciences, Sabzevar, Iran; Department of Epidemiology and Biostatistics (M Teramoto MD), University of California San Francisco, San Francisco, CA, USA; Outpatient Department (D R Terefa MSc), Wollega University, Bedele town, Ethiopia; Department of Pharmacology (P Thangaraju MD), All India Institute of Medical Sciences, Raipur, India; Public Health Department (Prof K R Thankappan MD), Amrita Institute of Medical Sciences, Kochi, India; Department of Gastroenterology (N K Thomas MD), St. Luke's Hospital, Patanamthitta, India; Faculty of Public Health (J H V Ticoalu MPH), Universitas Sam Ratulangi (Sam Ratulangi University), Manado, Indonesia; Institute of Public Health (R Topor-Madry PhD), Jagiellonian University Medical College, Kraków, Poland; Agency for Health Technology Assessment and Tariff System, Warsaw, Poland (R Topor-Madry PhD); High Institute of Sport and Physical Education of Sfax (K Trabelsi PhD), University of Sfax, Sfax, Tunisia; Department of Business Analytics (T H Tran MD), University of Massachusetts Dartmouth, Dartmouth, MA, USA; Molecular Neuroscience Research Center (N Tran Minh Duc MD), Shiga University of Medical Science, Shiga, Japan; Faculty of Medicine (T T Truyen MD), Nam Can Tho University, Can Tho, Vietnam; Department of Psychiatry (E Tsermpini PhD), Dalhousie University, Halifax, NS, Canada; Department of Occupational Health and Safety (A R Tualeka PhD), University of Development, Surabaya, Indonesia; Faculty of Health and Life Sciences (A Udoh PhD), University of Exeter, Exeter, UK; International Center for Chemical and Biological Sciences (S Ullah MSc), University of Karachi, Karachi, Pakistan; Medical Genomics Research Department (Prof M Umair PhD), King Abdullah International Medical Research Center, Riyadh, Saudi Arabia; Department of Cardiovascular, Endocrine-metabolic Diseases and Aging (B Unim PhD), National Institute of Health, Rome, Italy; College of Health and Sport Sciences (A G Vaithinathan MSc), University of Bahrain, Zallaq, Bahrain; UKK Institute, Tampere, Finland (Prof T J Vasankari PhD); Faculty of Medicine and Health Technology (Prof T J Vasankari PhD), Tampere University, Tampere, Finland; Department of Human Genetics & Molecular Biology (B Vellingiri PhD), Bharathiar University, Coimbatore, India; Raffles Neuroscience Centre (Prof N Venketasubramanian MSc), Raffles Hospital, Singapore, Singapore; School of Mathematics and Statistics (Prof P J Villeneuve PhD), Carleton University, Ottawa, ON, Canada; Occupational Medicine Unit (Prof F S Violante MD), Sant'Orsola Malpighi Hospital, Bologna, Italy; Department of Molecular Epidemiology (S K Vladimirov PhD), Research Institute for Systems Biology and Medicine, Moscow, Russia; Department of Medical Oncology (S Volovat PhD), University of Medicine and Pharmacy "Grigore T Popa" Iasi, Iaşi, Romania; Department of Medical Oncology (S Volovat PhD), Regional Institute of Oncology, Iaşi, Romania; School of Health Sciences (Prof Y Waheed PhD), National University of Sciences and Technology (NUST), Islamabad, Pakistan; Lebanese American University, Byblos, Lebanon (Prof Y Waheed PhD); Department of Neurosurgery (S Wang MD), Capital Medical University, Beijing, China; Department of Neurosurgery (S Wang MD), Beijing Tiantan Hospital, Beijing, China; School of Life Course

and Population Sciences (Prof Y Wang PhD), King's College London, London, UK; Key Laboratory of Computer-Aided Drug Design (M Waqas PhD), Guangdong Medical University, Dongguan, China; Department of Biotechnology and Genetic Engineering (M Waqas PhD), Hazara University Mansehra, Mansehra, Pakistan; Institute of Clinical Epidemiology, Public Health, Health Economics, Medical Statistics and Informatics (Prof P Willeit PhD), Medical University Innsbruck, Innsbruck, Austria; Department of Public Health and Primary Care (Prof P Willeit PhD), University of Cambridge, Cambridge, UK; Department of Research (M W Wojewodzic PhD), Cancer Registry of Norway, Oslo, Norway; National Data Management Center for Health (NDMC) (A A Wolde MPH), Ethiopian Public Health Institute, Addis Ababa, Ethiopia; Department of Public Health (T E Wonde MPH), Debre Markos University, Debre Markos, Ethiopia; School of Public Health (H Xiao PhD), Zhejiang University, Zhejiang, China; Department of Public Health Science (H Xiao PhD), Fred Hutchinson Cancer Research Center, Seattle, WA, USA; Department of Endocrinology (Prof S Xu PhD), University of Science and Technology of China, Hefei, China; Department of Public Health (Prof K Yamagishi MD, Prof N Yonemoto PhD), Faculty of Medicine (Y Yano MD), Juntendo University, Tokyo, Japan; Department of Cancer Epidemiology and Prevention Research (L Yang PhD), Alberta Health Services, Calgary, AB, Canada; Manipal College of Nursing (R Yesodharan MSc), Manipal Academy of Higher Education, Udupi, India; Biostatics, Epidemiology, and Science Computing Department (S Yezli PhD), King Faisal Specialist Hospital & Research Center, Riyadh, Saudi Arabia; Department of Respiratory Medicine (X Yi MD), Military Medical University, Chongqing, China; Department of Health Management (A Yiğit PhD), Süleyman Demirel Üniversitesi (Süleyman Demirel University), Isparta, Turkiye; Department of Epidemiology (D Yin DrPH), Xuzhou Medical University, Xuzhou, China; Department of Biostatistics (Prof N Yonemoto PhD), University of Toyama, Toyama, Japan; Department of Epidemiology and Biostatistics (Prof C Yu PhD), Wuhan University, Wuhan, China; Faculty of Medicine and Health Sciences (F Zakham PhD), Hodeidah University, Hodeidah, Yemen; School of Public Health (H Zhang MS), Peking University, Beijing, China; Victorian Comprehensive Cancer Centre, Melbourne, VIC, Australia (J Zhang MD); Medical Oncology Department of Gastrointestinal Cancer (L Zhang MS), Cancer Hospital of Dalian University of Technology, Shenyang, China; School of Biomedical Engineering (L Zhang MS), Dalian University of Technology, Dalian, China; School of Humanities and Management (Prof S C Zhou PhD), Zhejiang Chinese Medical University, Hangzhou, China; School of Public Health and Emergency Management (B Zhu PhD), Southern University of Science and Technology, Shenzhen, China; Endocrinology and Metabolism Research Center (G Zoghi MD), Hormozgan University of Medical Sciences, Bandar Abbas, Iran; Department of Clinical and Community Pharmacy (Prof S Zyoud PhD), An-Najah National University Hospital, Clinical Research Centre (Prof S Zyoud PhD), An-Najah National University, Nablus, Palestine

Authors' Contributions

Managing the overall research enterprise

Natalia V Bhattacharjee, Emmanuela Gakidou, Paulina A Lindstedt, Erin C Mullany, Amanda E Smith, and Stein Emil Vollset.

Writing the first draft of the manuscript

Dana Bryazka, Xiaochen Dai, Emmanuela Gakidou, Paulina A Lindstedt, Erin C Mullany, Marissa B Reitsma, Amanda E Smith, and Stein Emil Vollset.

Primary responsibility for applying analytical methods to produce estimates

Dana Bryazka, Xiaochen Dai, and Gabriela Fernanda Gil.

Primary responsibility for seeking, cataloguing, extracting, or cleaning data; designing or coding figures and tables

Dana Bryazka, Xiaochen Dai, Gabriela Fernanda Gil, and Marissa B Reitsma.

Providing data or critical feedback on data sources

Yohannes Habtegiorgis Abate, Rizwan Suliankatchi Abdulkader, E S Abhilash, Olugbenga Olusola Abiodun, Richard Gyan Aboagye, Lucas Guimarães Abreu, Niveen ME Abu-Rmeileh, Salahdein Aburuz, Ahmed Abu-Zaid, Mesafint Molla Adane, Akindele Olupelumi Adebiyi, Oyelola A Adegboye, Victor Adekanmbi, Habeeb Omoponle Adewuyi, Qorinah Estiningtyas Sakilah Adnani, Leticia Akua Adzigbli, Muhammad Sohail Afzal, Saira Afzal, Antonella Agodi, Bright Opoku Ahinkorah, Danish Ahmad, Muayyad M Ahmad, Sajjad Ahmad, Ali Ahmadi, Ayman Ahmed, Haroon Ahmed, Muktar Beshir Ahmed, Safoora Ahmed, Salah Al Awaidy, Abdelazeem M Algammal, Khalid F Alhabib, Abid Ali, Syed Shujait Ali, Sheikh Mohammad Alif, Syed Mohamed Aljunid, François Alla, Peter Allebeck, Jaber S Alqahtani, Najim Z Alshahrani, Awais Altaf, Nelson Alvis-Guzman, Mohammad Al-Wardat, Hubert Amu, Ganiyu Adeniyi Amusa, Tanu Anand, Deanna Anderlini, Jason A Anderson, Saeid Anvari, Sumadi Lukman Anwar, Jalal Arabloo, Elshaimaa A Arafa, Olatunde Aremu, Anton A Artamonov, Akeza Awealom Asgedom, Tahira Ashraf, Seyyed Shamsadin Athari, Prince Atorkey, Alok Atreya, Getinet Ayano, Setognal Birara Aychiluhm, Ahmed Y Azzam, Senthilkumar Balakrishnan, Maciej Banach, Mehmet Firat Baran, Martina Barchitta, Mainak Bardhan, Amadou Barrow, Mohammad-Mahdi Bastan, Sanjay Basu, Shelly L Bell, Alice A Beneke, Derrick A Bennett, Akshaya Srikanth Bhagavathula, Neeraj Bhala, Sonu Bhaskar, Ajay Nagesh Bhat, Priyadarshini Bhattacharjee, Jasvinder Singh Bhatti, Bijit Biswas, Berrak Bora Basara, Hamed Borhany, Souad Bouaoud, Dana Bryazka, Florentino Luciano Caetano dos Santos, Chao Cao, Joao Mauricio Castaldelli-Maia, Rama Mohan Chandika, Vijay Kumar Chattu, Akhilanand Chaurasia, Meng Xuan Chen, Abdulaal Chitheer, Bryan Chong, Chean Lin Chong, Hitesh Chopra, Dinh-Toi Chu, Michael H Criqui, Natalia Cruz-Martins, Siyu Dai, Xiaochen Dai, Giovanni Damiani, Lalit Dandona, Rakhi Dandona, Samuel D Darcho, Saswati Das, Aklilu Tamire Debele, Hardik Dineshbhai Desai, Devananda Devegowda, Vishal R Dhulipala, Thanh Chi Do, Milad Dodangeh, Ojas Prakashbhai Doshi, Rajkumar Prakashbhai Doshi, Robert Kokou Dowou, Haneil Larson Dsouza, Bruce B Duncan, Andre Rodrigues Duraes, Abdel Rahman E'mar, Alireza Ebrahimi, Diyan Ermawan Effendi, Michael Ekholuenetale, Rabie Adel El Arab, Ibrahim Farahat El Bayoumy, Gihan ELNahas, Chadi Eltaha, Adeniyi Francis Fagbamigbe, Omotayo Francis Fagbule, Ildar Ravisovich Fakhradiyev, Luca Falzone, Ali Fatehizadeh, Alireza Feizkhah, Ginenus Fekadu, Luisa S Flor, Celia Fortuna Rodrigues, Sridevi G, Peter Andras Gaal, Muktar A Gadanya, Emmanuela Gakidou, Silvano Gallus, Balasankar Ganesan, Teferi Gebru Gebremeskel, Lemma Getacher, Sherief Ghozy, Gabriela Fernanda Gil, Mahaveer Golechha, Pouya Goleij, Ayman Grada, Shi-Yang Guan, Avirup Guha, Ishita Gupta, Rajeev Gupta, Sapna Gupta, Veer Bala Gupta, Vivek Kumar Gupta, Najah R Hadi, Nadia M Hamdy, Josep Maria Haro, Faizul Hasan, Simon I Hay, Mehdi Hemmati, Delia Hendrie, Claudiu Herteliu, Demisu Zenbaba Heyi, Nguyen Quoc Hoan, Nobuyuki Horita, Md Mahbub Hossain, Segun Emmanuel Ibitoye, Nayu Ikeda, Olayinka Stephen Ilesanmi, Lalu Muhammad Irham, Gaetano Isola, Mahalaxmi Iyer, Jalil Jaafari, Khushleen Jaggi, Nader Jahanmehr, Nityanand Jain, Ammar Abdulrahman Jairoun, Mihajlo Jakovljevic, Reza Jalilzadeh Yengejeh, Sathish Kumar Jayapal, Shubha Jayaram, Bijay Mukesh Jeswani, Jost B Jonas, Tamas Joo, Charity Ehimwenma Joshua, Jacek Jerzy Jozwiak, Mikk Jürisson, Zubair Kabir, Vidya Kadashetti, Rami S Kantar, Samad Karkhah, Srinivasa Vittal

Katikireddi, Harkiran Kaur, Peter Njenga Keiyoro, Yousef Saleh Khader, Himanshu Khajuria, Ajmal Khan, Maseer Khan, Mohammad Jobair Khan, Moien AB Khan, Khaled Khatab, Haitham Khatatbeh, Amir M Khater, Manoj Khokhar, Atulya Aman Khosla, Min Seo Kim, Yun Jin Kim, Adnan Kisa, Shivakumar KM Marulasiddaiah Kondlahalli, Oleksii Korzh, Soewarta Kosen, Kewal Krishan, Barthelemy Kuate Defo, G Anil Kumar, Amartya Kundu, Dian Kusuma, Chandrakant Lahariya, Savita Lasrado, Thao Thi Thu Le, Munjae Lee, Seung Won Lee, Yo Han Lee, An Li, Virendra S Ligade, Stephen S Lim, Gang Liu, Erand Llanaj, Paulo A Lotufo, Jay B Lusk, Monika Machoy, Farzan Madadizadeh, Kashish Malhotra, Deborah Carvalho Malta, Pejman Mansouri, Mohammad Ali Mansournia, Roy Rillera Marzo, Medha Mathur, Navgeet Mathur, Rita Mattiello, Andrea Maugeri, Enkeleint A Mechili, Tesfahun Mekene Meto, Walter Mendoza, Ritesh G Menezes, Atte Meretoja, Tuomo J Meretoja, Irmina Maria Michalek, Erkin M Mirrakhimov, Ameen Mosa Mohammad, Shafiu Mohammed, Syam Mohan, Ali H Mokdad, Maryam Moradi, Vincent Mougin, George Duke Mukoro, Francesk Mulita, Malaisamy Muniyandi, Christopher J L Murray, Ganesh R Naik, Luigi Naldi, Jobert Richie Nansseu, Shumaila Nargus, Zuhair S Natto, Biswa Prakash Nayak, Vinod C Nayak, Ruxandra Irina Negoi, Reza Nejad Shahrokh Abadi, Josephine W Ngunjiri, Duc Hoang Nguyen, Hien Quang Nguyen, Phat Tuan Nguyen, Van Thanh Nguyen, Taxiarchis Konstantinos Nikolouzakis, Chukwudi A Nnaji, Lawrence Achilles Nnyanzi, Shuhei Nomura, Syed Toukir Ahmed Noor, Fred Nugen, Chimezie Igwegbe Nzoputam, Ogochukwu Janet Nzoputam, Bogdan Oancea, Kehinde O Obamiro, Ismail A Odetokun, Michael Safo Oduro, James Odhiambo Oguta, Akinkunmi Paul Okekunle, Osaretin Christabel Okonji, Andrew T Olagunju, Matthew Idowu Olatubi, Sok King Ong, Uchechukwu Levi Osuagwu, Stanislav S Otstavnov, Amel Ouyahia, Mahesh Padukudru P A, Jagadish Rao Padubidri, Demosthenes Panagiotakos, Songhomitra Panda-Jonas, Anamika Pandey, Anca Pantea Stoian, Paraskevi Papadopoulou, Shahina Pardhan, Romil R Parikh, Jay Patel, Sangram Kishor Patel, Shankargouda Patil, Shrikant Pawar, Prince Peprah, Gavin Pereira, Arokiasamy Perianayagam, Richard G Pestell, Tom Pham, Anil K Philip, Ramesh Poluru, Jalandhar Pradhan, Manya Prasad, Elton Junio Sady Prates, Jagadeesh Puvvula, Ibrahim Qattea, Pankaja Raghav, Fakher Rahim, Mohammad Rahmanian, Pushp Lata Rajpoot, Pradhum Ram, Mahmoud Mohammed Ramadan, Rishabh Kumar Rana, Chhabi Lal Ranabhat, Sowmya J Rao, Sina Rashedi, Ahmed Mustafa Rashid, Santosh Kumar Rauniyar, Nakul Ravikumar, Salman Rawaf, Elrashdy Moustafa Mohamed Redwan, Marissa B Reitsma, Monica Rodrigues, Jefferson Antonio Buendia Rodriguez, Leonardo Roever, Kevin T Root, Gholamreza Roshandel, Cameron John Sabet, Siamak Sabour, Basema Ahmad Saddik, Umar Saeed, Sher Zaman Safi, Fatemeh Saheb Sharif-Askari, Mirza Rizwan Sajid, Abdallah M Samy, Juan Sanabria, Milena M Santric-Milicevic, Tanmay Sarkar, Brijesh Sathian, Anudeep Sathyanarayan, Maheswar Satpathy, Monika Sawhney, Mete Saylan, Maria Inês Schmidt, Subramanian Senthilkumaran, Yashendra Sethi, Seyed Arsalan Seyedi, Samiah Shahid, Ahmed Shaikh, Masood Ali Shaikh, Muhammad Aaqib Shamim, Anas Shamsi, Alfiya Shamsutdinova, Abhishek Shankar, Mohammed Shannawaz, Medha Sharath, Amin Sharifan, Vishal Sharma, Aminu Shittu, Sunil Shrestha, Abhinav Singh, Baljinder Singh, Jasvinder A Singh, Paramdeep Singh, Virendra Singh, Marco Solmi, Ireneous N Soyiri, Michael Spartalis, Chandrashekhar T Sreeramareddy, Aleksandar Stevanović, Muhammad Suleman, Chandan Kumar Swain, Sree Sudha T Y, Jabeen Taiba, Mircea Tampa, Ker-Kan Tan, Pugazhenthan Thangaraju, Nikhil Kenny Thomas, Krishna Tiwari, Roman Topor-Madry, Marcos Roberto Tovani-Palone, Khaled Trabelsi, An Thien Tran, Ngoc Ha Tran, Indang Trihandini, Jaya Prasad Tripathy, Abdul Rohim Tualeka, Muhammad Umair, Bhaskaran Unnikrishnan, Jibrin Sammani Usman, Jef Van den Eynde, Tommi Juhani Vasankari, Balachandar Vellingiri, Narayanaswamy Venketasubramanian, Simona Ruxandra Volovat, Abdul Wadood, Yasir Waheed, Shu Wang, Peter Willeit, Tewodros Eshete Wonde,

Hong Xiao, Suowen Xu, Kazumasa Yamagishi, Yuichiro Yano, Amir Yarahmadi, Dong Keon Yon, Naohiro Yonemoto, Chuanhua Yu, Jianrong Zhang, and Magdalena Zielińska.

Developing methods or computational machinery

Qorinah Estiningtyas Sakilah Adnani, Saira Afzal, Austin J Ahlstrom, Ali Ahmadi, Muktar Beshir Ahmed, Safoora Ahmed, Abdelazeem M Algammal, Jaber S Alqahtani, Walid Adnan Al-Zyoud, Hubert Amu, Aleksandr Y Aravkin, Ahmed Y Azzam, Mainak Bardhan, Amadou Barrow, Mohammad-Mahdi Bastan, Akshaya Srikanth Bhagavathula, Natalia V Bhattacharjee, Hamed Borhany, Dana Bryazka, Fan Cao, Hitesh Chopra, Dinh-Toi Chu, Siyu Dai, Xiaochen Dai, Hardik Dineshbhai Desai, Thanh Chi Do, Michael Ekholuenetale, Iman El Sayed, Mehdi Emamverdi, Adeniyi Francis Fagbamigbe, Ali Fatehizadeh, Emmanuela Gakidou, Sherief Ghozy, Shi-Yang Guan, Simon I Hay, Mohammad Heidari, Lalu Muhammad Irham, Gaetano Isola, Sathish Kumar Jayapal, Bijay Mukesh Jeswani, Charity Ehimwenma Joshua, Samad Karkhah, Faizan Zaffar Kashoo, Peter Njenga Keiyoro, Atulya Aman Khosla, Adnan Kisa, Chandrakant Lahariya, Thao Thi Thu Le, Erand Llanaj, Elham Mahmoudi, Ali H Mokdad, Vincent Mougin, Francesk Mulita, Christopher J L Murray, Shumaila Nargus, Josephine W Ngunjiri, Phat Tuan Nguyen, Van Thanh Nguyen, Michal Ordak, Amel Ouyahia, Catalina Raggi, Pradhum Ram, Rishabh Kumar Rana, Chhabi Lal Ranabhat, Marissa B Reitsma, Monica Rodrigues, Umar Saeed, Sher Zaman Safi, Abdallah M Samy, Maheswar Satpathy, Austin E Schumacher, Mohammad H Semreen, Yashendra Sethi, Mohammed Shannawaz, Seyed Afshin Shorofi, Amanda E Smith, Michael Spartalis, Muhammad Suleman, Chandan Kumar Swain, Sree Sudha T Y, Muhammad Umair, Stein Emil Vollset, Tewodros Eshete Wonde, Xinglin Yi, Chun-Wei Yuan, and Mohammed G M Zeariya.

Providing critical feedback on methods or results

Yohannes Habtegiorgis Abate, Abdallah H A Abd Al Magied, Atef Abdelkader, Arash Abdollahi, Meriem Abdoun, Rizwan Suliankatchi Abdulkader, Roberto Ariel Abeldaño Zuñiga, E S Abhilash, Olugbenga Olusola Abiodun, Richard Gyan Aboagye, Lucas Guimarães Abreu, Dariush Abtahi, Hasan Abualruz, Bilyaminu Abubakar, Salahdein Aburuz, Ahmed Abu-Zaid, Mesafint Molla Adane, Akindele Olupelumi Adebiyi, Oyelola A Adegboye, Victor Adekanmbi, Habeeb Omoponle Adewuyi, Qorinah Estiningtyas Sakilah Adnani, Leticia Akua Adzigbli, Siamak Afaghi, Muhammad Sohail Afzal, Saira Afzal, Antonella Agodi, Williams Agyemang-Duah, Bright Opoku Ahinkorah, Aqeel Ahmad, Danish Ahmad, Muayyad M Ahmad, Sajjad Ahmad, Shahzaib Ahmad, Ali Ahmadi, Anisuddin Ahmed, Ayman Ahmed, Haroon Ahmed, Muktar Beshir Ahmed, Mohammed Ahmed Akkaif, Ema Akter, Salah Al Awaidy, Syed Mahfuz Al Hasan, Yazan Al-Ajlouni, Ziyad Al-Aly, Khurshid Alam, Zufishan Alam, Abdelazeem M Algammal, Adel Ali Saeed Al-Gheethi, Khalid F Alhabib, Mohammed Khaled Al-Hanawi, Abid Ali, Mohammed Usman Ali, Rafat Ali, Syed Shujait Ali, Waad Ali, Sheikh Mohammad Alif, Syed Mohamed Aljunid, Peter Allebeck, Wael Almahmeed, Sabah Al-Marwani, Sadeq Al-Maweri, Mahmoud A Alomari, Jaber S Alqahtani, Ahmed Yaseen Alqutaibi, Rajaa M Mohammad Al-Raddadi, Saqr Alsakarneh, Najim Z Alshahrani, Zaid Altaany, Awais Altaf, Nelson Alvis-Guzman, Mohammad Al-Wardat, Yaser Mohammed Al-Worafi, Hany Aly, Safwat Aly, Mohammad Sharif Ibrahim Alyahya, Karem H Alzoubi, Walid Adnan Al-Zyoud, Reza Amani, Tarek Tawfik Amin, Sohrab Amiri, Hubert Amu, Gianna Gayle Herrera Amul, Tanu Anand, Deanna Anderlini, David B Anderson, Catalina Liliana Andrei, Tudorel Andrei, Mohammed Tahir Ansari, Iyadunni Adesola Anuoluwa, Saeid Anvari, Sumadi Lukman Anwar, Anayochukwu Edward Anyasodor, Jalal Arabloo, Elshaimaa A Arafa, Demelash Areda, Olatunde Aremu, Anton A Artamonov, Akeza Awealom Asgedom, Mohammad Asghari-Jafarabadi, Mubarek Yesse Ashemo, Tahira Ashraf, Thomas Astell-Burt, Seyyed Shamsadin Athari, Prince Atorkey, Alok Atreya, Adedapo Wasiu Awotidebe, Getinet Ayano,

Setognal Birara Aychiluhm, Sina Azadnajafabad, Ahmed Y Azzam, Giridhara Rathnaiah Babu, Pegah Bahrami Taghanaki, Saeed Bahramian, Ruhai Bai, Senthilkumar Balakrishnan, Maciej Banach, Soham Bandyopadhyay, Mehmet Firat Baran, Martina Barchitta, Amadou Barrow, Hameed Akande Bashiru, Afisu Basiru, Mohammad-Mahdi Bastan, Sanjay Basu, Saurav Basu, Kavita Batra, Mohsen Bayati, Amir Hossein Behnoush, Shelly L Bell, Alice A Beneke, Derrick A Bennett, Isabela M Bensenor, Amiel Nazer C Bermudez, Habtamu B Beyene, Devidas S Bhagat, Akshaya Srikanth Bhagavathula, Neeraj Bhala, Nikha Bhardwaj, Pankaj Bhardwaj, Sonu Bhaskar, Ajay Nagesh Bhat, Natalia V Bhattacharjee, Priyadarshini Bhattacharjee, Jasvinder Singh Bhatti, Cem Bilgin, Bijit Biswas, Micheal Kofi Boachie, Eyob Ketema Bogale, Hamed Borhany, Samuel Adolf Bosoka, Souad Bouaoud, Edward J Boyko, Hermann Brenner, Andre R Brunoni, Dana Bryazka, Raffaele Bugiardini, Norma B Bulamu, Yasser Bustanji, Zahid A Butt, Florentino Luciano Caetano dos Santos, Chao Cao, Fan Cao, Angelo Capodici, Rosario Cárdenas, Joao Mauricio Castaldelli-Maia, Arthur Caye, Luca Cegolon, Edina Cenko, Sonia Cerrai, Rama Mohan Chandika, Eeshwar K Chandrasekar, Vijay Kumar Chattu, Akhilanand Chaurasia, An-Tian Chen, Guangjin Chen, Haowei Chen, Meng Xuan Chen, Kent Jason Go Cheng, Gerald Chi, Ritesh Chimoriya, Jesus Lorenzo Chirinos-Caceres, Bryan Chong, Chean Lin Chong, Yuen Yu Chong, Hitesh Chopra, Sonali Gajanan Choudhari, Dinh-Toi Chu, Isaac Sunday Chukwu, Sheng-Chia Chung, Muhammad Chutiyami, Joao Conde, Alexandru Corlateanu, Michael H Criqui, Natalia Cruz-Martins, Alanna Gomes da Silva, Omid Dadras, Siyu Dai, Xiaochen Dai, Giovanni Damiani, Lalit Dandona, Rakhi Dandona, Samuel D Darcho, Reza Darvishi Cheshmeh Soltani, Saswati Das, Nihar Ranjan Dash, Kairat Davletov, Aklilu Tamire Debele, Daniel Demant, Hardik Dineshbhai Desai, Devananda Devegowda, Syed Masudur Rahman Dewan, Arkadeep Dhali, Amol S Dhane, Vishal R Dhulipala, Thanh Chi Do, Milad Dodangeh, Deepa Dongarwar, Mario D'Oria, Ojas Prakashbhai Doshi, Rajkumar Prakashbhai Doshi, Robert Kokou Dowou, Haneil Larson Dsouza, Viola Savy Dsouza, Arkadiusz Marian Dziedzic, Abdel Rahman E'mar, Alireza Ebrahimi, Negar Ebrahimi, Mohammad Ebrahimi Kalan, David Edvardsson, Kristina Edvardsson, Ferry Efendi, Diyan Ermawan Effendi, Foolad Eghbali, Michael Ekholuenetale, Rabie Adel El Arab, Ibrahim Farahat El Bayoumy, Iman El Sayed, Muhammed Elhadi, Waseem El-Huneidi, Mohamed A Elmonem, Gihan ELNahas, Ibrahim Elsohaby, Chadi Eltaha, Mohd Elmagzoub Eltahir, Mehdi Emamverdi, Theophilus I Emeto, Daniel Asfaw Erku, Farshid Etaee, Natalia Fabin, Adeniyi Francis Fagbamigbe, Omotayo Francis Fagbule, Shahriar Faghani, Ayesha Fahim, Ildar Ravisovich Fakhradiyev, Luca Falzone, Umar Farooque, Ali Fatehizadeh, Zareen Fatima, Timur Fazylov, Alireza Feizkhah, Ginenus Fekadu, Xiaoqi Feng, Pietro Ferrara, Bikila Regassa Feyisa, Filippos T Filippidis, Florian Fischer, Luisa S Flor, Nataliya A Foigt, Celia Fortuna Rodrigues, Matteo Foschi, Sridevi G, Peter Andras Gaal, Muktar A Gadanya, Abhay Motiramji Gaidhane, Márió Gajdács, Emmanuela Gakidou, Aravind P Gandhi, Balasankar Ganesan, Rupesh K Gautam, Miglas Welay Gebregergis, Mesfin Gebrehiwot, Teferi Gebru Gebremeskel, Lemma Getacher, Ramy Mohamed Ghazy, Ali Gholamrezanezhad, Sherief Ghozy, Artyom Urievich Gil, Gabriela Fernanda Gil, Elena V Gnedovskaya, Sonu Goel, Salime Goharinezhad, Mohamad Goldust, Mahaveer Golechha, Davide Golinelli, Ayman Grada, Michal Grivna, Shekhar Grover, Shi-Yang Guan, Mohammed Ibrahim Mohialdeen Gubari, Avirup Guha, Stefano Guicciardi, Snigdha Gulati, Damitha Asanga Gunawardane, Sasidhar Gunturu, Zhifeng Guo, Anish Kumar Gupta, Bhawna Gupta, Ishita Gupta, Mohak Gupta, Sapna Gupta, Veer Bala Gupta, Vipin Gupta, Vivek Kumar Gupta, Mostafa Hadei, Najah R Hadi, Ali Hajj Ali, Nadia M Hamdy, Samer Hamidi, Ahmad Hammoud, Graeme J Hankey, Arief Hargono, Ahmed I Hasaballah, Faizul Hasan, Md Kamrul Hasan, Md Saquib Hasnain, Ikrama Ibrahim Hassan, Shoaib Hassan, Simon I Hay, Behzad Heibati, Mohammad Heidari, Mehdi Hemmati, Delia Hendrie, Claudiu Herteliu, Demisu Zenbaba Heyi, Kamal Hezam, Yuta Hiraike, Nguyen Quoc Hoan, Ramesh Holla, Nobuyuki Horita,

Md Mahbub Hossain, Sahadat Hossain, Hassan Hosseinzadeh, Mihaela Hostiuc, Ayesha Humayun, Javid Hussain, Bing-Fang Hwang, Segun Emmanuel Ibitoye, Olayinka Stephen Ilesanmi, Irena M Ilic, Milena D Ilic, Mustapha Immurana, Muhammad Iqhrammullah, Lalu Muhammad Irham, Md Rabiul Islam, Sheikh Mohammed Shariful Islam, Farhad Islami, Gaetano Isola, Ramaiah Itumalla, Masao Iwagami, Mahalaxmi Iyer, Vinothini J, Jalil Jaafari, Louis Jacob, Khushleen Jaggi, Nader Jahanmehr, Akhil Jain, Nityanand Jain, Ammar Abdulrahman Jairoun, Sanobar Jaka, Mihajlo Jakovljevic, Reza Jalilzadeh Yengejeh, Elham Jamshidi, Sathish Kumar Jayapal, Shubha Jayaram, Ruwan Duminda Jayasinghe, Rime Jebai, Sun Ha Jee, Bijay Mukesh Jeswani, Heng Jiang, Mohammad Jokar, Jost B Jonas, Tamas Joo, Nitin Joseph, Charity Ehimwenma Joshua, Mikk Jürisson, Vaishali K, Ali Kabir, Zubair Kabir, Vidya Kadashetti, Sivesh Kathir Kamarajah, Mona Kanaan, Kehinde Kazeem Kanmodi, Rami S Kantar, Paschalis Karakasis, Ibraheem M Karaye, Salah Eddin Karimi, Yeganeh Karimi, Arman Karimi Behnagh, Samad Karkhah, Prabin Karki, Faizan Zaffar Kashoo, Srinivasa Vittal Katikireddi, Harkiran Kaur, Navjot Kaur, Sina Kazemian, Tahseen Haider Kazmi, Peter Njenga Keiyoro, Emmanuelle Kesse-Guyot, Yousef Saleh Khader, Himanshu Khajuria, Amirmohammad Khalaji, Alireza Khalilian, Ajmal Khan, Maseer Khan, Mohammad Jobair Khan, Moien AB Khan, Shaghayegh Khanmohammadi, Khaled Khatab, Haitham Khatatbeh, Moawiah Mohammad Khatatbeh, Amir M Khater, Khalid A Kheirallah, Manoj Khokhar, Moein Khormali, Atulya Aman Khosla, Sepehr Khosravi, Kwanghyun Kim, Min Seo Kim, Yun Jin Kim, Adnan Kisa, Ali-Asghar Kolahi, Somayeh Komaki, Shivakumar KM Marulasiddaiah Kondlahalli, Miikka Korja, Oleksii Korzh, Kewal Krishan, Barthelemy Kuate Defo, Mohammed Kuddus, Omar Kujan, Ashish Kumar, G Anil Kumar, Nithin Kumar, Vijay Kumar, Amartya Kundu, Satyajit Kundu, Setor K Kunutsor, Om P Kurmi, Dian Kusuma, Frank Kyei-Arthur, Ville Kytö, Carlo La Vecchia, Chandrakant Lahariya, Daphne Teck Ching Lai, Hanpeng Lai, Ratilal Lalloo, Tea Lallukka, Bagher Larijani, Savita Lasrado, Jerrald Lau, Paolo Lauriola, Thao Thi Thu Le, Janet L Leasher, Munjae Lee, Seung Won Lee, Wei-Chen Lee, Yo Han Lee, Elvynna Leong, Temesgen Leka Lerango, An Li, Wei Li, Virendra S Ligade, Stephen S Lim, Jialing Lin, Gang Liu, Erand Llanaj, José Francisco López-Gil, Paulo A Lotufo, Giancarlo Lucchetti, Jay B Lusk, Hawraz Ibrahim M Amin, Zheng Feei Ma, Monika Machoy, Farzan Madadizadeh, Elham Mahmoudi, Abdelrahman M Makram, Omar M Makram, Kashish Malhotra, Ahmad Azam Malik, Deborah Carvalho Malta, Abdullah A Mamun, Mohammad Ali Mansournia, Emmanuel Manu, Hamid Reza Marateb, Jose Martinez-Raga, Miquel Martorell, Roy Rillera Marzo, Yasith Mathangasinghe, Elezebeth Mathews, Medha Mathur, Navgeet Mathur, Rita Mattiello, Andrea Maugeri, Martin McKee, Enkeleint A Mechili, Ravi Mehrotra, Tesfahun Mekene Meto, Birye Dessalegn Mekonnen, Hadush Negash Meles, Walter Mendoza, Ritesh G Menezes, Sultan Ayoub Meo, Atte Meretoja, Tomislav Mestrovic, Caine C A Meyers, Irmina Maria Michalek, Ted R Miller, Erkin M Mirrakhimov, Vinaytosh Mishra, Sanjeev Misra, Prasanna Mithra, Ahmed Ismail Mohamed, Jama Mohamed, Mouhand F H Mohamed, Nouh Saad Mohamed, Ameen Mosa Mohammad, Sakineh Mohammad-Alizadeh-Charandabi, Ibrahim Mohammadzadeh, Shafiu Mohammed, Syam Mohan, Ali H Mokdad, Hossein Molavi Vardanjani, Sabrina Molinaro, Shaher Momani, Himel Mondal, Ute Mons, AmirAli Moodi Ghalibaf, Maryam Moradi, Rafael Silveira Moreira, Negar Morovatdar, Shane Douglas Morrison, George Duke Mukoro, Francesk Mulita, Malaisamy Muniyandi, Yanjinlkham Munkhsaikhan, Efren Murillo-Zamora, Christopher J L Murray, Woojae Myung, Pirouz Naghavi, Ganesh R Naik, Soroush Najdaghi, Hastyar Hama Rashid Najmuldeen, Vinay Nangia, Jobert Richie Nansseu, Shumaila Nargus, Abdulqadir J Nashwan, Zuhair S Natto, Javaid Nauman, Muhammad Naveed, Nawsherwan, Biswa Prakash Nayak, Athare Nazri-Panjaki, Sabina Onyinye Nduaguba, Ruxandra Irina Negoi, Reza Nejad Shahrokh Abadi, Seyed Aria Nejadghaderi, Chakib Nejjari, Subas Neupane, Marie Ng, Josephine W Ngunjiri, Duc Hoang Nguyen, Hau Thi Hien Nguyen, Hien Quang Nguyen, Phat Tuan Nguyen, Phuong The

Nguyen, Van Thanh Nguyen, Taxiarchis Konstantinos Nikolouzakis, Ali Nikoobar, Nasrin Nikravangolsefid, Vikram Niranjan, Chukwudi A Nnaji, Lawrence Achilles Nnyanzi, Efaq Ali Noman, Syed Toukir Ahmed Noor, Mamoona Noreen, Majid Nozari, Fred Nugen, Chimezie Igwegbe Nzoputam, Ogochukwu Janet Nzoputam, Bogdan Oancea, Kehinde O Obamiro, Ismail A Odetokun, Oluwakemi Ololade Odukoya, Michael Safo Oduro, James Odhiambo Oguta, Hassan Okati-Aliabad, Akinkunmi Paul Okekunle, Osaretin Christabel Okonji, Andrew T Olagunju, Omotola O Olasupo, Matthew Idowu Olatubi, Gláucia Maria Moraes Oliveira, Abdulhakeem Abayomi Olorukooba, Goran Latif Omer, Sok King Ong, Abdulahi Opejin Opejin, Michal Ordak, Uchechukwu Levi Osuagwu, Stanislav S Otstavnov, Amel Ouyahia, Mahesh Padukudru P A, Alicia Padron-Monedero, Jagadish Rao Padubidri, Anton Pak, Hai-Feng Pan, Demosthenes Panagiotakos, Songhomitra Panda-Jonas, Anamika Pandey, Leonidas D Panos, Ioannis Pantazopoulos, Anca Pantea Stoian, Paraskevi Papadopoulou, Shahina Pardhan, Pragyan Paramita Parija, Romil R Parikh, Eun-Kee Park, Seoyeon Park, Roberto Passera, Jay Patel, Sangram Kishor Patel, Shankargouda Patil, Hridoy Patwary, Shrikant Pawar, Prince Peprah, Gavin Pereira, Arokiasamy Perianayagam, Richard G Pestell, Fanny Emily Petermann-Rocha, Anil K Philip, Michael R Phillips, Roman V Polibin, Ramesh Poluru, Fabio Porru, Akram Pourshams, Jalandhar Pradhan, Pranil Man Singh Pradhan, Manya Prasad, Akila Prashant, Elton Junio Sady Prates, Dimas Ria Angga Pribadi, Bharathi M Purohit, Jagadeesh Puvvula, Ibrahim Qattea, Venkatraman Radhakrishnan, Pankaja Raghav, Fakher Rahim, Afarin Rahimi-Movaghar, Md Mosfequr Rahman, Mosiur Rahman, Muhammad Aziz Rahman, Shayan Rahmani, Mohammad Rahmanian, Nazanin Rahmanian, Vinoth Rajendran, Pushp Lata Rajpoot, Prashant Rajput, Pradhum Ram, Mahmoud Mohammed Ramadan, Majed Ramadan, Kritika Rana, Rishabh Kumar Rana, Chhabi Lal Ranabhat, Sowmya J Rao, Sina Rashedi, Ahmed Mustafa Rashid, Mohammad-Mahdi Rashidi, Ashkan Rasouli-Saravani, Devarajan Rathish, Santosh Kumar Rauniyar, Ilari Rautalin, Nakul Ravikumar, Salman Rawaf, Murali Mohan Rama Krishna Reddy, Elrashdy Moustafa Mohamed Redwan, Marissa B Reitsma, Negar Rezaei, Mohsen Rezaeian, Abanoub Riad, Monica Rodrigues, Thales Philipe R Rodrigues da Silva, Jefferson Antonio Buendia Rodriguez, Leonardo Roever, Kevin T Root, Gholamreza Roshandel, Allen Guy Ross, Himanshu Sekhar Rout, Simanta Roy, Chandan S N, Cameron John Sabet, Siamak Sabour, Kabir P Sadarangani, Basema Ahmad Saddik, Mohammad Reza Saeb, Umar Saeed, Sher Zaman Safi, Fatemeh Saheb Sharif-Askari, Soumya Swaroop Sahoo, Md Refat Uz Zaman Sajib, Mirza Rizwan Sajid, Mohamed A Saleh, Yoseph Leonardo Samodra, Abdallah M Samy, Juan Sanabria, Milena M Santric-Milicevic, Bruno Piassi Sao Jose, Muhammad Arif Nadeem Saqib, Babak Saravi, Yaser Sarikhani, Tanmay Sarkar, Gargi Sachin Sarode, Sachin C Sarode, Benn Sartorius, Brijesh Sathian, Anudeep Sathyanarayan, Maheswar Satpathy, Monika Sawhney, Mete Saylan, Markus P Schlaich, Art Schuermans, Mohammad H Semreen, Subramanian Senthilkumaran, Sadaf G Sepanlou, Yashendra Sethi, Seyed Arsalan Seyedi, Mahan Shafie, Arman Shafiee, Ataollah Shahbandi, Samiah Shahid, Hamid R Shahsavari, Ahmed Shaikh, Masood Ali Shaikh, Ali S Shalash, Muhammad Aaqib Shamim, Anas Shamsi, Mohd Shanawaz, Abhishek Shankar, Mohammed Shannawaz, Medha Sharath, Amin Sharifan, Ujjawal Sharma, Vishal Sharma, Aziz Sheikh, Ali Sheikhy, Desalegn Shiferaw, Tariku Shimels, Rahman Shiri, Aminu Shittu, Ivy Shiue, Seyed Afshin Shorofi, Sunil Shrestha, Emmanuel Edwar Siddig, João Pedro Silva, Abhinav Singh, Baljinder Singh, Harmanjit Singh, Jasvinder A Singh, Paramdeep Singh, Puneetpal Singh, Virendra Singh, Freddy Sitas, Amanda E Smith, Matiwos Soboka, Marco Solmi, Soroush Soraneh, Joan B Soriano, Ireneous N Soyiri, Michael Spartalis, Chandrashekhar T Sreeramareddy, Paschalis Steiropoulos, Aleksandar Stevanović, Kurt Straif, Muhammad Suleman, Gerhard Sulo, Vinay Suresh, Chandan Kumar Swain, Lukasz Szarpak, Sree Sudha T Y, Payam Tabaee Damavandi, Ozra Tabatabaei Malazy, Seyed-Amir Tabatabaeizadeh, Celine Tabche, Jyothi Tadakamadla, Santosh Kumar Tadakamadla, Jabeen Taiba, Iman M Talaat, Ashis Talukder,

Mircea Tampa, Jacques Lukenze JL Tamuzi, Ker-Kan Tan, Minale Tareke, Ingan Ukur Tarigan, Mojtaba Teimoori, Mohamad-Hani Temsah, Reem Mohamad Hani Temsah, Masayuki Teramoto, Dufera Rikitu Terefa, Pugazhenthan Thangaraju, Kavumpurathu Raman Thankappan, Rekha Thapar, Rasiah Thayakaran, Nikhil Kenny Thomas, Jansje Henny Vera Ticoalu, Roman Topor-Madry, Marcos Roberto Tovani-Palone, Khaled Trabelsi, An Thien Tran, Ngoc Ha Tran, Thang Huu Tran, Nguyen Tran Minh Duc, Indang Trihandini, Jaya Prasad Tripathy, Aniefiok John Udoakang, Arit Udoh, Atta Ullah, Saeed Ullah, Muhammad Umair, Bhaskaran Unnikrishnan, Jibrin Sammani Usman, Sanaz Vahdati, Jef Van den Eynde, Constantine Vardavas, Siavash Vaziri, Balachandar Vellingiri, Narayanaswamy Venketasubramanian, Madhur Verma, Paul J Villeneuve, Manish Vinayak, Francesco S Violante, Stein Emil Vollset, Simona Ruxandra Volovat, Abdul Wadood, Yasir Waheed, Mandaras Tariku Walde, Shu Wang, Yanzhong Wang, Muhammad Waqas, Nuwan Darshana Wickramasinghe, Peter Willeit, Marcin W Wojewodzic, Asrat Arja Wolde, Tewodros Eshete Wonde, Hong Xiao, Suowen Xu, Mukesh Kumar Yadav, Danting Yang, Lin Yang, Amir Yarahmadi, Renjulal Yesodharan, Saber Yezli, Xinglin Yi, Arzu Yiğit, Dehui Yin, Dong Keon Yon, Naohiro Yonemoto, Seok-Jun Yoon, Chuanhua Yu, Fathiah Zakham, Mohammed G M Zeariya, Haijun Zhang, Jianrong Zhang, Liqun Zhang, Claire Chenwen Zhong, Shang Cheng Zhou, Bin Zhu, Magdalena Zielińska, and Sa'ed H Zyoud.

Drafting the work or revising it critically for important intellectual content

Yohannes Habtegiorgis Abate, Abdallah H A Abd Al Magied, Atef Abdelkader, Arash Abdollahi, Roberto Ariel Abeldaño Zuñiga, Olugbenga Olusola Abiodun, Olumide Abiodun, Lucas Guimarães Abreu, Hasan Abualruz, Niveen ME Abu-Rmeileh, Salahdein Aburuz, Ahmed Abu-Zaid, Mesafint Molla Adane, Oyelola A Adegboye, Victor Adekanmbi, Habeeb Omoponle Adewuyi, Qorinah Estiningtyas Sakilah Adnani, Aanuoluwapo Adeyimika Afolabi, Muhammad Sohail Afzal, Saira Afzal, Antonella Agodi, Bright Opoku Ahinkorah, Austin J Ahlstrom, Danish Ahmad, Muayyad M Ahmad, Ali Ahmadi, Anisuddin Ahmed, Ayman Ahmed, Haroon Ahmed, Muktar Beshir Ahmed, Safoora Ahmed, Marjan Ajami, Mohammed Ahmed Akkaif, Ema Akter, Salah Al Awaidy, Yazan Al-Ajlouni, Khurshid Alam, Zufishan Alam, Wafa A Aldhaleei, Abdelazeem M Algammal, Fadwa Naji Alhalaiqa, Mohammed Khaled Al-Hanawi, Abid Ali, Mohammed Usman Ali, Rafat Ali, Syed Shujait Ali, Waad Ali, Peter Allebeck, Wael Almahmeed, Sadeq Al-Maweri, Mahmoud A Alomari, Jaber S Alqahtani, Sahel Majed Alrousan, Saqr Alsakarneh, Najim Z Alshahrani, Zaid Altaany, Awais Altaf, Nelson Alvis-Guzman, Mohammad Al-Wardat, Yaser Mohammed Al-Worafi, Hany Aly, Safwat Aly, Mohammad Sharif Ibrahim Alyahya, Karem H Alzoubi, Walid Adnan Al-Zyoud, Reza Amani, Tarek Tawfik Amin, Sohrab Amiri, Hubert Amu, Ganiyu Adeniyi Amusa, Tanu Anand, Deanna Anderlini, David B Anderson, Iyadunni Adesola Anuoluwa, Saeid Anvari, Anayochukwu Edward Anyasodor, Jalal Arabloo, Elshaimaa A Arafa, Brhane Berhe Aregawi, Olatunde Aremu, Seyyed Shamsadin Athari, Prince Atorkey, Alok Atreya, Avinash Aujayeb, Adedapo Wasiu Awotidebe, Sina Azadnajafabad, Ahmed Y Azzam, Giridhara Rathnaiah Babu, Ruhai Bai, Shankar M Bakkannavar, Senthilkumar Balakrishnan, Kiran Bam, Maciej Banach, Soham Bandyopadhyay, Mehmet Firat Baran, Martina Barchitta, Mainak Bardhan, Suzanne Lyn Barker-Collo, Amadou Barrow, Hameed Akande Bashiru, Mohammad-Mahdi Bastan, Sanjay Basu, Amir Hossein Behnoush, Shelly L Bell, Luis Belo, Alice A Beneke, Isabela M Bensenor, Azizullah Beran, Habtamu B Beyene, Akshaya Srikanth Bhagavathula, Neeraj Bhala, Sonu Bhaskar, Ajay Nagesh Bhat, Natalia V Bhattacharjee, Priyadarshini Bhattacharjee, Jasvinder Singh Bhatti, Cem Bilgin, Atanu Biswas, Bijit Biswas, Hamed Borhany, Samuel Adolf Bosoka, Souad Bouaoud, Edward J Boyko, Hermann Brenner, Andre R Brunoni, Dana Bryazka, Raffaele Bugiardini, Norma B Bulamu, Yasser Bustanji, Florentino Luciano Caetano dos Santos, Daniela Calina, Angelo Capodici, Giulia Carreras, Joao Mauricio Castaldelli-Maia, Maria Sofia Cattaruzza, Arthur Caye, Luca

Cegolon, Edina Cenko, Sandip Chakraborty, Rama Mohan Chandika, Eeshwar K Chandrasekar, Vijay Kumar Chattu, Anis Ahmad Chaudhary, Akhilanand Chaurasia, An-Tian Chen, Guangjin Chen, Haowei Chen, Meng Xuan Chen, Simiao Chen, Fatemeh Chichagi, Ritesh Chimoriya, Jesus Lorenzo Chirinos-Caceres, Bryan Chong, Yuen Yu Chong, Hitesh Chopra, Joao Conde, Michael H Criqui, Natalia Cruz-Martins, Alanna Gomes da Silva, Siyu Dai, Xiaochen Dai, Giovanni Damiani, Samuel D Darcho, Nihar Ranjan Dash, Aklilu Tamire Debele, Shayom Debopadhaya, Hardik Dineshbhai Desai, Devananda Devegowda, Syed Masudur Rahman Dewan, Arkadeep Dhali, Amol S Dhane, Vishal R Dhulipala, Thanh Chi Do, Milad Dodangeh, Phidelia Theresa Doegah, Sushil Dohare, Deepa Dongarwar, Mario D'Oria, Rajkumar Prakashbhai Doshi, Robert Kokou Dowou, Ashel Chelsea Dsouza, Haneil Larson Dsouza, Viola Savy Dsouza, Bruce B Duncan, Arkadiusz Marian Dziedzic, Abdel Rahman E'mar, Negar Ebrahimi, David Edvardsson, Diyan Ermawan Effendi, Foolad Eghbali, Michael Ekholuenetale, Rabie Adel El Arab, Ibrahim Farahat El Bayoumy, Iman El Sayed, Iffat Elbarazi, Muhammed Elhadi, Mohamed A Elmonem, Gihan ELNahas, Chadi Eltaha, Mohd Elmagzoub Eltahir, Theophilus I Emeto, Farshid Etaee, Elochukwu Fortune Ezenwankwo, Natalia Fabin, Adeniyi Francis Fagbamigbe, Omotayo Francis Fagbule, Shahriar Faghani, Ayesha Fahim, Luca Falzone, Ali Fatehizadeh, Zareen Fatima, Nelsensius Klau Fauk, Pietro Ferrara, Nuno Ferreira, Filippos T Filippidis, Florian Fischer, Matteo Foschi, Peter Andras Gaal, Muktar A Gadanya, Márió Gajdács, Emmanuela Gakidou, Silvano Gallus, Balasankar Ganesan, Prem Gautam, Rupesh K Gautam, Miglas Welay Gebregergis, Teferi Gebru Gebremeskel, Lemma Getacher, Fataneh Ghadirian, Ramy Mohamed Ghazy, Mahsa Ghorbani, Sherief Ghozy, Elena V Gnedovskaya, Mohamad Goldust, Davide Golinelli, Giuseppe Gorini, Mahdi Gouravani, Ayman Grada, Michal Grivna, Shekhar Grover, Shi-Yang Guan, Avirup Guha, Stefano Guicciardi, Snigdha Gulati, Damitha Asanga Gunawardane, Sasidhar Gunturu, Bhawna Gupta, Ishita Gupta, Mohak Gupta, Rajeev Gupta, Sapna Gupta, Veer Bala Gupta, Vivek Kumar Gupta, Mostafa Hadei, Najah R Hadi, Ali Hajj Ali, Esam S Halboub, Nadia M Hamdy, Ahmad Hammoud, Graeme J Hankey, Josep Maria Haro, Ahmed I Hasaballah, Md Kamrul Hasan, Md Saquib Hasnain, Amr Hassan, Shoaib Hassan, Simon I Hay, Mehdi Hemmati, Claudiu Herteliu, Demisu Zenbaba Heyi, Kamal Hezam, Yuta Hiraike, Nguyen Quoc Hoan, Ramesh Holla, Nobuyuki Horita, Md Mahbub Hossain, Sahadat Hossain, Sorin Hostiuc, Junjie Huang, Javid Hussain, Segun Emmanuel Ibitoye, Adalia Ikiroma, Olayinka Stephen Ilesanmi, Irena M Ilic, Milena D Ilic, Mustapha Immurana, Leeberk Raja Inbaraj, Muhammad Iqhrammullah, Lalu Muhammad Irham, Md Rabiul Islam, Sheikh Mohammed Shariful Islam, Farhad Islami, Gaetano Isola, Ramaiah Itumalla, Mahalaxmi Iyer, Vinothini J, Louis Jacob, Abdollah Jafarzadeh, Khushleen Jaggi, Nader Jahanmehr, Akhil Jain, Sanobar Jaka, Mihajlo Jakovljevic, Manthan Dilipkumar Janodia, Talha Jawaid, Sathish Kumar Jayapal, Shubha Jayaram, Ruwan Duminda Jayasinghe, Rime Jebai, Bijay Mukesh Jeswani, Jost B Jonas, Tamas Joo, Nitin Joseph, Charity Ehimwenma Joshua, Mikk Jürisson, Vaishali K, Ali Kabir, Vidya Kadashetti, Sivesh Kathir Kamarajah, Kehinde Kazeem Kanmodi, Surya Kant, Rami S Kantar, Paschalis Karakasis, Yeganeh Karimi, Prabin Karki, Faizan Zaffar Kashoo, Srinivasa Vittal Katikireddi, Navjot Kaur, Peter Njenga Keiyoro, Emmanuelle Kesse-Guyot, Yousef Saleh Khader, Himanshu Khajuria, Amirmohammad Khalaji, Alireza Khalilian, Ajmal Khan, Maseer Khan, Moien AB Khan, Shaghayegh Khanmohammadi, Khaled Khatab, Haitham Khatatbeh, Moawiah Mohammad Khatatbeh, Khalid A Kheirallah, Atulya Aman Khosla, Kwanghyun Kim, Min Seo Kim, Yun Jin Kim, Adnan Kisa, Shivakumar KM Marulasiddaiah Kondlahalli, Oleksii Korzh, Karel Kostev, Kewal Krishan, Barthelemy Kuate Defo, Mohammed Kuddus, Omar Kujan, Mukhtar Kulimbet, Rakesh Kumar, Amartya Kundu, Satyajit Kundu, Setor K Kunutsor, Om P Kurmi, Dian Kusuma, Frank Kyei-Arthur, Ville Kytö, Carlo La Vecchia, Chandrakant Lahariya, Hanpeng Lai, Ratilal Lalloo, Tea Lallukka, Savita Lasrado, Jerrald Lau, Thao Thi Thu Le, Elvynna Leong, Wei Li, Paulina A Lindstedt, Erand Llanaj, José

Francisco López-Gil, Paulo A Lotufo, Giancarlo Lucchetti, Alessandra Lugo, Jay B Lusk, Zheng Feei Ma, Monika Machoy, Farzan Madadizadeh, Elham Mahmoudi, Abdelrahman M Makram, Omar M Makram, Kashish Malhotra, Ahmad Azam Malik, Deborah Carvalho Malta, Abdullah A Mamun, Emmanuel Manu, Hamid Reza Marateb, Jose Martinez-Raga, Miquel Martorell, Roy Rillera Marzo, Yasith Mathangasinghe, Medha Mathur, Navgeet Mathur, Andrea Maugeri, Enkeleint A Mechili, Tesfahun Mekene Meto, Hadush Negash Meles, Walter Mendoza, Ritesh G Menezes, Sultan Ayoub Meo, Atte Meretoja, Tuomo J Meretoja, Tomislav Mestrovic, Caine C A Meyers, Irmina Maria Michalek, Ted R Miller, Giuseppe Minervini, Mojgan Mirghafourvand, Prasanna Mithra, Mouhand F H Mohamed, Nouh Saad Mohamed, Ameen Mosa Mohammad, Sakineh Mohammad-Alizadeh-Charandabi, Hussen Mohammed, Shafiu Mohammed, Ali H Mokdad, Sabrina Molinaro, Himel Mondal, Ute Mons, AmirAli Moodi Ghalibaf, Maryam Moradi, Rafael Silveira Moreira, Shane Douglas Morrison, George Duke Mukoro, Francesk Mulita, Malaisamy Muniyandi, Yanjinlkham Munkhsaikhan, Efren Murillo-Zamora, Christopher J L Murray, Soroush Najdaghi, Gopal Nambi, Jobert Richie Nansseu, Shumaila Nargus, Gustavo G Nascimento, Zuhair S Natto, Javaid Nauman, Nawsherwan, Biswa Prakash Nayak, Vinod C Nayak, Sabina Onyinye Nduaguba, Ruxandra Irina Negoi, Seyed Aria Nejadghaderi, Subas Neupane, Josephine W Ngunjiri, Duc Hoang Nguyen, Hau Thi Hien Nguyen, Hien Quang Nguyen, Phat Tuan Nguyen, Van Thanh Nguyen, Yeshambel T Nigatu, Taxiarchis Konstantinos Nikolouzakis, Nasrin Nikravangolsefid, Vikram Niranjan, Lawrence Achilles Nnyanzi, Mamoona Noreen, Fred Nugen, Chimezie Igwegbe Nzoputam, Ogochukwu Janet Nzoputam, Bogdan Oancea, Kehinde O Obamiro, Ismail A Odetokun, Daniel Bogale Odo, In-Hwan Oh, Sylvester Reuben Okeke, Osaretin Christabel Okonji, Andrew T Olagunju, Omotola O Olasupo, Matthew Idowu Olatubi, Abdulhakeem Abayomi Olorukooba, Michal Ordak, Verner N Orish, Esteban Ortiz-Prado, Uchechukwu Levi Osuagwu, Stanislav S Otstavnov, Amel Ouyahia, Mahesh Padukudru P A, Alicia Padron-Monedero, Jagadish Rao Padubidri, Raul Felipe Palma-Alvarez, Songhomitra Panda-Jonas, Ioannis Pantazopoulos, Paraskevi Papadopoulou, Shahina Pardhan, Nicholas Parsons, Roberto Passera, Jay Patel, Shankargouda Patil, Shrikant Pawar, Gavin Pereira, Arokiasamy Perianayagam, Richard G Pestell, Fanny Emily Petermann-Rocha, Michael R Phillips, Dimitri Poddighe, Ramesh Poluru, Fabio Porru, Jalandhar Pradhan, Pranil Man Singh Pradhan, Manya Prasad, Akila Prashant, Elton Junio Sady Prates, Jagadeesh Puvvula, Venkatraman Radhakrishnan, Pankaja Raghav, Fakher Rahim, Afarin Rahimi-Movaghar, Md Mosfequr Rahman, Shayan Rahmani, Mohammad Rahmanian, Vinoth Rajendran, Pushp Lata Rajpoot, Prashant Rajput, Pradhum Ram, Mahmoud Mohammed Ramadan, Kritika Rana, Rishabh Kumar Rana, Chhabi Lal Ranabhat, Sowmya J Rao, Ahmed Mustafa Rashid, Ashkan Rasouli-Saravani, Devarajan Rathish, Ilari Rautalin, Nakul Ravikumar, Salman Rawaf, Elrashdy Moustafa Mohamed Redwan, Marissa B Reitsma, Abanoub Riad, Monica Rodrigues, Thales Philipe R Rodrigues da Silva, Jefferson Antonio Buendia Rodriguez, Leonardo Roever, Kevin T Root, Gholamreza Roshandel, Allen Guy Ross, Bedanta Roy, Nitai Roy, Simanta Roy, Guilherme de Andrade Ruela, Chandan S N, Cameron John Sabet, Siamak Sabour, Kabir P Sadarangani, Basema Ahmad Saddik, Masoumeh Sadeghi, Umar Saeed, Pooya Saeedi, Dominic Sagoe, Fatemeh Saheb Sharif-Askari, Amirhossein Sahebkar, Soumya Swaroop Sahoo, Md Refat Uz Zaman Sajib, Mirza Rizwan Sajid, Luciane B Salaroli, Mohammed Z Y Salem, Dauda Salihu, Abdallah M Samy, Juan Sanabria, Milena M Santric-Milicevic, Bruno Piassi Sao Jose, Made Ary Sarasmita, Aswini Saravanan, Babak Saravi, Yaser Sarikhani, Tanmay Sarkar, Gargi Sachin Sarode, Sachin C Sarode, Anudeep Sathyanarayan, Maheswar Satpathy, Mete Saylan, Benedikt Michael Schaarschmidt, Michael P Schaub, Markus P Schlaich, Maria Inês Schmidt, Art Schuermans, Siddharthan Selvaraj, Mohammad H Semreen, Sadaf G Sepanlou, Yashendra Sethi, Allen Seylani, Mahan Shafie, Arman Shafiee, Samiah Shahid, Moyad Jamal Shahwan, Ali S Shalash,

Muhammad Aaqib Shamim, Anas Shamsi, Alfiya Shamsutdinova, Mohd Shanawaz, Abhishek Shankar, Mohammed Shannawaz, Medha Sharath, Amin Sharifan, Manoj Sharma, Ujjawal Sharma, Vishal Sharma, Ali Sheikhy, Mahabalesh Shetty, Pavanchand H Shetty, Premalatha K Shetty, Tariku Shimels, Aminu Shittu, Velizar Shivarov, Seyed Afshin Shorofi, Sunil Shrestha, Emmanuel Edwar Siddig, João Pedro Silva, Abhinav Singh, Harmanjit Singh, Jasvinder A Singh, Paramdeep Singh, Puneetpal Singh, Surjit Singh, Ranjan Solanki, Marco Solmi, Joan B Soriano, Ireneous N Soyiri, Michael Spartalis, Chandrashekhar T Sreeramareddy, Panagiotis Stachteas, Dan J Stein, Paschalis Steiropoulos, Aleksandar Stevanović, Muhammad Suleman, Zhong Sun, Chandan Kumar Swain, Lukasz Szarpak, Payam Tabaee Damavandi, Seyed-Amir Tabatabaeizadeh, Celine Tabche, Jyothi Tadakamadla, Santosh Kumar Tadakamadla, Iman M Talaat, Mircea Tampa, Jacques Lukenze JL Tamuzi, Ker-Kan Tan, Minale Tareke, Mohamad-Hani Temsah, Reem Mohamad Hani Temsah, Masayuki Teramoto, Dufera Rikitu Terefa, Pugazhenthan Thangaraju, Roman Topor-Madry, Marcos Roberto Tovani-Palone, Khaled Trabelsi, Thang Huu Tran, Nguyen Tran Minh Duc, Jaya Prasad Tripathy, Thien Tan Tri Tai Truyen, Evangelia Eirini Tsermpini, Aniefiok John Udoakang, Arit Udoh, Muhammad Umair, Brigid Unim, Bhaskaran Unnikrishnan, Jibrin Sammani Usman, Sanaz Vahdati, Asokan Govindaraj Vaithinathan, Jef Van den Eynde, Constantine Vardavas, Tommi Juhani Vasankari, Balachandar Vellingiri, Narayanaswamy Venketasubramanian, Madhur Verma, Manish Vinayak, Stein Emil Vollset, Simona Ruxandra Volovat, Shu Wang, Yanzhong Wang, Nuwan Darshana Wickramasinghe, Peter Willeit, Marcin W Wojewodzic, Tewodros Eshete Wonde, Mukesh Kumar Yadav, Kazumasa Yamagishi, Lin Yang, Amir Yarahmadi, Renjulal Yesodharan, Saber Yezli, Xinglin Yi, Arzu Yiğit, Naohiro Yonemoto, Haijun Zhang, Jianrong Zhang, Claire Chenwen Zhong, Bin Zhu, Magdalena Zielińska, Ghazal Zoghi, and Sa'ed H Zyoud.

Managing the estimation or publications process

Dana Bryazka, Emmanuela Gakidou, Simon I Hay, Paulina A Lindstedt, Ali H Mokdad, Erin C Mullany, Amanda E Smith, and Stein Emil Vollset.
